# Supplementary material for: Global burden of major chronic respiratory diseases among older adults aged 55 and above from 1990 to 2021: Changes, challenges, and predictions amid the pandemic
Source: PLoS One. 2025 Aug 1;20(8):e0329283. doi: 10.1371/journal.pone.0329283 (PMC12316243; doi:10.1371/journal.pone.0329283)
Supplement: S1 File — (PDF) [file pone.0329283.s001.pdf]

Supplementary Material-1

Additional Methodological Explanations

1. Global Burden of Disease Database 1

2. Global Burden of Disease Study 3

3. Disease Definition 5

4. R Packages Used in This Study 5

5. Age-Standardized Rate for Population Aged 55 and Above (TASR55+) 6

6. Estimated Annual Percentage Change(EAPC) 6

7. Joinpoint Regression Analysis Model 7

8. Age-Period-Cohort (APC) Model 8

9. Cross-National Inequality 10

10. Attributable Risk Factors 12

11. Predictive Model 12

12. Decomposition Analysis 14

References 16

1. Global Burden of Disease Database

1.1 Relevant Institutions and Objectives of GBD

The Global Burden of Disease (GBD) study is a large-scale collaborative project conducted by the World Health Organization, the World Bank, the Bill & Melinda Gates Foundation, and numerous academic and research institutions. Launched in 1990, GBD aims to systematically assess the disease burden at the global, regional, and national levels, as well as its trends over time, providing essential scientific evidence for health policy formulation and resource allocation[1].

1.2 Data Sources of GBD

The data in GBD 2021 is sourced from a variety of comprehensive methods, including systematic reviews, epidemiological surveys, administrative health records, verbal autopsies, censuses, household surveys, and disease-specific registries. This database is renowned for its rigorous maintenance and regular updates, aggregating scientific data from over 90,000 different sources, including published epidemiological studies and government publications[1].

1.3 Main Contents of GBD

GBD 2021 is the most extensive and scientifically rigorous global epidemiological burden assessment to date, covering 371 diseases and injuries, as well as 88 risk factors. The disease burden statistics encompass the global level, 7 super-regions, 21 regions, and 204 countries and territories[2]. Additionally, GBD 2021 includes COVID-19 for the first time as a comprehensive infectious disease burden estimate and evaluates its impact on the burden of specific diseases. The database, led by the Institute for Health Metrics and Evaluation (IHME), systematically provides global disease burden estimates for various age groups, genders, and geographical regions. It includes data on incidence, prevalence, mortality, Disability-Adjusted Life Years (DALY), Years Lived with Disability (YLD), and Years of Life Lost (YLL), with 95% uncertainty intervals (UI) to reflect the uncertainty of the estimates[3]. These indicators facilitate comparisons between countries, regions, and periods, aiding researchers and policymakers in

monitoring health trends, evaluating intervention effects, and more effectively allocating health resources.

1.4 GBD Modeling

GBD employs advanced modeling techniques[1, 4], including Meta-Regression-Bayesian, Regularized, Trimmed(Mr-BRT), Cause of Death Ensemble modeling (CODEm), Spatiotemporal Gaussian Process Regression (ST-GPR), and the Bayesian meta-regression tool DisMod-MR 2.1. These models estimate disease incidence, prevalence, mortality, YLL, YLD, DALY, and Healthy Life Expectancy (HALE), with detailed analyses across dimensions such as etiology, age, sex, year, and geographic location.

MR-BRT is another key tool used by the Institute for Health Metrics and Evaluation (IHME) at the University of Washington. It is a meta-regression model that incorporates uncertainty in the dependent variable. The purpose of MR-BRT is to standardize diagnostic criteria, dimensions, or exposure definitions across different studies, providing correction coefficients and purified effect values, while also displaying explicit transfer of standard errors/covariances. This approach significantly improves analytical accuracy, especially when dealing with complex datasets.[2, 5]。

DisMod-MR 2.1, a Bayesian meta-regression model, is used to estimate disease incidence and prevalence. This model fully accounts for uncertainty in the data and assigns standard errors to each observation, generating more accurate and reliable estimates. DisMod-MR 2.1 performs stratified modeling by age, sex, year, and country to correct for data biases, while performing 1,000 random samplings for each parameter to simulate its possible range of values. It retains and propagates the uncertainty introduced by measurement errors, model assumptions, and regional heterogeneity, ultimately constructing the uncertainty interval (UI) using the 2.5th and 97.5th percentiles from the 1,000 samplings[6]. The width of the interval directly reflects the robustness of the estimates: wider intervals suggest data scarcity or higher model uncertainty (common in low-income countries), while narrower intervals indicate sufficient data and stable models. Although the UI cannot fully capture structural biases

in the model or systematic data biases, it provides a transparent quantification of statistical uncertainty, serving as an important tool for evaluating the credibility of results in global health decision-making. Therefore, when interpreting results, caution must be exercised in conjunction with the regional context and model assumptions.

CODEm is a specialized analytical tool developed by the Institute for Health Metrics and Evaluation (IHME), designed to systematically estimate global trends and distributions of causes of death by integrating multi-source data (such as cause-of-death registries and survey data) with complex modeling methods. The model's principle is based on a covariate selection algorithm that generates hundreds to thousands of candidate models (including mixed-effects linear models, spatiotemporal Gaussian process regression models, etc.). It then filters these models based on out-of-sample predictive validity and integrates them using Bayesian model averaging, enabling high-precision predictions of mortality rates and cause-of-death composition ratios. This tool can quantify changes in causes of death across different regions, time periods, and age dimensions.

2. Global Burden of Disease Study

2.1 Disease and Risk Data

We downloaded data related to Chronic Respiratory Diseases (CRDs), including COPD, asthma, and ILD & PS, for populations aged 55 and older at the global, regional, and national levels from the GBD 2021 database. GBD 2021 includes new analyses on disease-related risk factors[7]. Therefore, we extracted relevant risk factors related to CRDs, which encompass three categories of risk factors: environmental and occupational risks, behavioral risks, and metabolic risks, with a total of 19 subcategories across four levels (Table S1).

2.2 Population Data

We extracted population data from GBD for the years 1990-2021, following the evaluation process and content outlined in the latest GBD population studies[8, 9]. Additionally, we obtained the corresponding ratios for the World Standard Population

and population projection data until the end of this century from the World Health Organization (WHO). Using age-specific standardized rates, we forecasted the burden trends of CRDs.

2.3 Sociodemographic Index (SDI)

The Sociodemographic Index (SDI) was introduced in 2015 by the Institute for Health Metrics and Evaluation (IHME) to assess the level of development of countries or regions. It emphasizes the relationship between social development and population health outcomes and is influenced by factors such as fertility rate, education level, and per capita income. A higher SDI typically indicates better health outcomes, reflecting improvements in socioeconomic conditions. As social demographics progress, the strengthening of healthcare systems, increased educational opportunities, and economic growth generally contribute to better health levels, but may also present new challenges. Based on the SDI scores provided by GBD 2021[10], 204 countries and regions are classified into five levels: low SDI ($SDI < 0.45$), low-middle SDI ($0.45 \leq SDI < 0.61$), middle SDI ($0.61 \leq SDI < 0.69$), high-middle SDI ($0.69 \leq SDI < 0.80$), and high SDI ($SDI \geq 0.80$). The SDI range is from 0 to 1, with 0 representing the lowest level of development and 1 representing the highest level of development.

2.4 Additional Explanation of Disease Burden

Years Lived with Disability (YLD): Refers to the years of life lost due to disability caused by diseases. It is calculated by multiplying the number of cases by the disease duration (until remission or death) and the disability weight (DW), which quantifies non-fatal health losses.

Years of Life Lost (YLL): Refers to the years of life lost due to premature death associated with diseases. It is calculated by multiplying the number of deaths by the expected life expectancy for the corresponding age, sex, region, and year.

Disability-Adjusted Life Years (DALY): DALY is a composite indicator used to measure the health life years lost due to diseases. DALY = YLL + YLD.

Patient age groups are divided into 9 subgroups: 55-59 years, 60-64 years, 65-69 years, 70-74 years, 75-79 years, 80-84 years, 85-89 years, 90-94 years, and >95 years.

3. Disease Definition

International Classification of Diseases ICD-9/10 related codes

Cause	ICD-10	ICD-9
Chronic respiratory diseases	D86-D86.2, D86.9, G47.3, J30-J35.9, J37-J39.9, J41-J46.9, J60-J63.8, J65-J68.9, J70, J70.8-J70.9,J82, J84-J84.9, J91, J91.8-J92.9	135-135.9, 327.2-327.8, 470, 470.9-474.9, 476-476.1, 477-479, 491-493.9, 495-504.9, 506-506.9, 508-509, 515, 516-517.8, 518.6, 518.9,519.1-519.4
Chronic obstructive pulmonary disease	J41-J44.9	491-492.9, 496-499
Asthma	J45-J46.9	493-493.9
Interstitial lung disease and pulmonary sarcoidosis	D86-D86.2, D86.9, J84-J84.9	135-135.9, 515, 516-516.9

4. R Packages Used in This Study

Data Organization & Manipulation

dplyr, data.table, tidyr, purrr, stringr, reshape, plyr, tidyverse, foreach, car

Visualization & Plotting

ggplot2, ggsci, fanplot, RColorBrewer, ggbrace, patchwork, grid, ggmap

Spatial/GIS Analysis

sp, sf, maps

Statistical Modeling & Inference

MASS, mgcv, splines, broom, INLA, nordpred, BAPC

Epidemiological Analysis

Epi, epitools

5. Age-Standardized Rate for Population Aged 55 and Above (TASR55+)

When comparing outcomes across multiple groups, age standardization is necessary to eliminate the impact of age differences, especially for conservative predictions in future disease forecasting models. The data is sourced from the GBD database for the age group 55 and above, as well as the age-specific ratios from the World Standard Population provided by the WHO. The WHO data is re-normalized, and then the Truncated Age Standardized Rate ($TASR_{55+}$) is calculated using the following theoretical formula[1, 11]:

$$TASR_{55+} = \frac{\sum_{g=55-59}^{k=95+} r_g w_g}{\sum_{g=55-59}^{k=95+} w_g}$$

where r_g is the rate for the $g-th$ age group, w_g is the number of people in the reference standard population corresponding to that age group, and k is the upper limit of the highest age group.

6. Estimated Annual Percentage Change(EAPC)

EAPC is used to measure the annual percentage change in disease indicators by applying a linear regression to the natural logarithm of age-specific rates over time. The linear regression model is as follows[1]:

$$ln(R_y) = \alpha + \beta x + \varepsilon$$

Where α is the intercept, β is the annual change rate, ε is the error term, and x represents the years.

The formula for calculating EAPC is as follows[12]:

$$EAPC = 100 \times (exp(\beta) - 1)$$

Where β is the regression coefficient or the annual change rate.

EAPC can reveal changes in a time series and is widely used. If EAPC and its 95% confidence interval (CI) lower bound are both >0, the indicator is on an upward trend; if the EAPC and its 95% CI upper bound are both <0, the trend is downward; if the 95% CI contains 0, it is considered stable.

However, EAPC has some limitations[13]. EAPC implicitly assumes a "single slope linear" trend. Therefore, if the true time trend includes inflection points or nonlinear curvature, EAPC will forcefully average multiple stages of change, potentially masking significant phase transitions. Additionally, its estimates are highly sensitive to the start and end years of the study period, with slight changes in the observation window potentially leading to significant differences. Moreover, EAPC only models the overall time series and cannot distinguish structural factors such as age, period, or cohort, nor can it simultaneously account for covariates or handle excessive dispersion.

7. Joinpoint Regression Analysis Model

The data analysis portion of the Joinpoint regression model was performed in R version 4.4.1, while the trend analysis was conducted using Joinpoint V5.2.0. The model fits a segmented regression model using a grid search algorithm (GSM) and enumerates all candidate inflection points. The optimal number of inflection points is determined by minimizing the Mean Squared Error (MSE), and the Monte Carlo method is used to estimate permutation tests, allowing for 0-5 inflection points to identify the best-fit model[14, 15]. The model testing starts from $k = 0$（with $k_{max} = 5$）and incrementally increases k until the model corresponding to $k = k_{max}$ is reached, which represents the final optimal model.

The Annual Percent Change (APC) within each segment is calculated using the geometric mean, while the Average Annual Percent Change (AAPC) is the weighted average of all segment APCs, reflecting the overall trend. In this study, AAPC and APC values for the period 1990–2021 were calculated, and their 95% confidence intervals (CI) were used to describe disease trend changes.

The logarithmic linear regression fitted for each time segment[16] is as follows：

$$\log(R_y) = b_0 + b_i y$$

The formula for calculating APC is:

$$APC_i = (\exp(b_i) - 1) * 100$$

The formula for calculating AAPC is:[17]

$$AAPC = \left(\exp\left(\frac{\sum w_i b_i}{\sum w_i}\right) - 1\right) * 100$$

Where b_i is the regression slope coefficient for the $i-th$ segment, w_i is the weight corresponding to the time span of the segment, and R_y is the rate for the year y.

Interpretation:

If AAPC/APC and their 95% confidence intervals (CI) are both > 0, it indicates a significant upward trend.

If AAPC/APC and their 95% CI are both < 0, it indicates a significant downward trend.

If AAPC/APC and their 95% CI contain 0, it indicates no statistically significant change, and the trend is stable.

Joinpoint regression models are particularly suitable for analyzing long-term time series data as they can objectively identify statistically significant inflection points and estimate the AAPC for each segment. Compared to traditional models, this method is more sensitive in capturing phase changes in trends, such as those before and after the COVID-19 pandemic, making it an ideal tool for assessing changes in disease burden over decades. Compared to EAPC, AAPC provides a more accurate depiction of segmented trends, resulting in more robust overall change rates.

8. Age-Period-Cohort (APC) Model

The APC model is used to evaluate the effects of age, period, and birth cohort on outcomes. This study used the NIH APC Web Tool to examine trends in the burden of disease and employed the Intrinsic Estimator (IE) and parametric hypothesis testing to calculate the Wald statistic[16]. The APC analysis utilized global data from 1992 to 2021, with age groups defined in 5-year intervals. Time periods were divided into six intervals, from 1992–1996 to 2017–2021, with the reference period set as 2012–2016 (RR=1). A linear dependency exists between the age, period, and birth cohort parameters $(cohort = period - age)$. The APC model addresses this interrelationship by separating these time components, allowing for the simultaneous evaluation of their impact on disease burden. The primary focus of the APC model was to analyze the

changes in prevalence and incidence globally, by region, and in 11 example countries.

Significance of the APC Model：

In trend studies, while we may primarily focus on one temporal dimension, such as the change in the prevalence of a certain disease over time, in actual research, it is inevitable to simultaneously involve age, period, and cohort dimensions. When age and cohort are neglected and only period changes are discussed, the implicit assumption is that for each observed period, the composition of age and cohort is essentially similar, or that different age and cohort groups do not exert differential effects on the observed dependent variable. However, in reality, this assumption is often not valid. Therefore, the observed changes in prevalence over time may also be influenced by cohort effects due to differences in cohort composition. To truly understand the changes in prevalence across the temporal dimension, it is necessary to separate the potential cohort and age effects, which is the essence of Age-Period-Cohort (APC) analysis.

The APC model is applied to estimate the independent effects of age, period, and cohort factors on the burden of chronic non-communicable diseases. The model first uses stepwise likelihood ratio tests or AIC/BIC to assess whether period and birth cohort are significant. Then, it calculates net and local drift. A drift of ≥1% per year with a $Wald\chi^2P < 0.05$ is considered a significant trend. The significance lies in assessing whether age-standardized rates and EAPC adequately capture the temporal trends for each age group.

Age Effect: Represents the magnitude of the impact of increasing age on disease burden outcomes.

Period Effect： Reflects the temporal changes in the risk of lead-related diseases over the years.

Cohort Effect： Reflects differences in outcomes for groups born in different eras, due to changes in birth methods or different exposures.

APC Model Variables:

Net drift： Similar to EAPC (Estimated Annual Percentage Change), it simultaneously reflects the effects of both period and birth cohort.

Local drift： The trend in disease rates over time for each age group, reflecting the trend in cohort effects.

Longitudinal age curve： Used to present the age effect, i.e., the impact that increases with age.

Period rate ratios： Used to display the period effect, i.e., the changes in disease burden over time.

Cohort rate ratios： Used to show the cohort effect, i.e., the differences in performance of the same group of individuals over different periods.

9. Cross-National Inequality

Following the recommendations of the World Health Organization (WHO), we use the Slope Index of Inequality (SII) and Concentration Index (CI) to assess the distributional inequality of disease burden.

SII is used to measure the average health level difference between the highest and lowest socio-economic groups, and it is an absolute inequality indicator. This method was first proposed by Preston, Haines, and Pamuk and can be estimated using weighted least squares in the following linear regression framework[18]：

$$y_i = \beta_0 + \beta_1 R_i$$

y_i: Health outcome for the i-th socio-economic group.

R_i: The relative rank of the socio-economic group in the overall population's socio-economic distribution.

β_0: The health level of individuals at the bottom rank($R = 0$).

β_1: The linear slope, or SII, representing the health difference between individuals at the bottom rank and the top rank($R = 1$).

Calculation of Relative Rank R_i:

Let p_j be the population proportion of the $\boldsymbol{j-th}$ socio-economic group in the total population. The rank of the $\boldsymbol{j-th}$ group is given by:

$$R_j = \sum_{k=1}^{j-1} p_k + 0.5\, p_j$$

This represents the midpoint of the cumulative population proportion for each group.

Weighted Least Squares Estimate for SII:

$$\widehat{\mathrm{SII}} = \widehat{\beta_1} = \frac{\sum_{j=1}^{J} p_j R_j (y_j - \mu)}{\sum_{j=1}^{J} p_j R_j^2 - \left(\sum_{j=1}^{J} p_j R_j\right)^2}$$

Where:

$$\mu = \sum_{j=1}^{J} p_j y_j$$

represents the overall weighted average health level. The weight p_j ensures that the sample structure aligns with the overall population structure.

An SII value of 0 indicates no inequality; the larger the absolute value, the higher the level of inequality.

CI is used to measure the degree of health inequity between countries with different socio-economic statuses, based on the concentration curve [1, 17, 19].

Calculation Method: Epidemiological burden and population in different countries are arranged in ascending order based on SDI scores. The cumulative population proportion (X-axis) and the cumulative age-standardized prevalence ratio (Y-axis) are calculated, and the concentration curve is plotted. The CI is then calculated as the ratio of the area between the Lorenz curve and the diagonal line to the area under the diagonal line.

A positive CI value (Lorenz curve below the diagonal) indicates that the epidemiological burden is concentrated in countries with higher socio-economic development.

A negative CI value (Lorenz curve above the diagonal) indicates that the epidemiological burden is concentrated in countries with lower socio-economic development.

The closer the absolute value of the CI is to 0, the lower the level of inequity.

The absolute value of the CI ranges from 0 to 1. The closer it is to 0, the more equal the health distribution; the closer it is to 1, the more unequal the health distribution.

10. Attributable Risk Factors

We downloaded risk factor data related to CRDs from the GBD study. Detailed information and definitions of the risk factors can be found in the supplementary file (Table S2).

GBD 2021 uses the Comparative Risk Assessment (CRA) framework to quantify the contribution of various risk factors to the disease burden through the "exposure-risk-attribution" chain. First, methods like spatiotemporal Gaussian process regression (ST-GPR) are used to integrate survey, monitoring, and literature data to estimate the exposure distributions for different countries, age groups, and genders. Then, dose-response meta-analyses of prospective cohort studies are conducted to obtain relative risk curves for continuous exposure levels, with the theoretical minimum risk exposure level (TMREL) set as the optimal health baseline. The comparison between the population's current exposure distribution and TMREL can be made using the following formula (to calculate population attributable fraction, PAF)[20]:

$$PAF = \left(\sum_{i=1}^{n} P_i(RR_i - 1)\right) / \left(\sum_{i=1}^{n} P_i(RR_i - 1) + 1\right)$$

Where:

P_i is the proportion of the population at exposure level i.

RR_i is the relative risk corresponding to exposure level i.

n is the total number of exposure levels.

Next, PAF is multiplied by the total number of deaths adjusted by CODEm and the YLL and YLD estimates obtained from DisMod-MR 2.1 to calculate attributable deaths and attributable DALYs. The related calculation formulas are as follows:

$$Deaths_{risk} = PAF \times Deaths_{total}$$

$$DALY_{risk} = [YLL \times PAF] + [YLD \times PAF]$$

11. Predictive Model

Bayesian Age-Period-Cohort Model (BAPC)

To assess the future disease burden, we employed the BAPC model. The BAPC model

is built on the Bayesian Generalized Linear Model (GLM) framework and applies second-order random walk (RW2) priors to age, period, and cohort effects, enabling smoothness along the temporal dimension. The model uses Integrated Nested Laplace Approximation (INLA) to directly approximate the marginal posterior distribution, thus avoiding the challenges of mixing and convergence typically associated with traditional MCMC. This approach has shown excellent computational efficiency and interval coverage in high-dimensional sparse data, such as that in GBD 2021[16, 21]。

The basic formula for the BAPC model is as follows:

$$\log\left(Rate_{ijk}\right) = \alpha + \mu_i + \beta_j + \gamma_k + \varepsilon_{ijk}$$

Where:

$Rate_{ijt}$ represents the disease burden rate at time i, for age group j, and birth cohort k;

α is the intercept term;

μ_i represents the period effect that varies by time i;

β_j represents the age effect that varies by age group j;

γ_k represents the cohort effect that varies by birth cohort k;

$\varepsilon_{ijt}$is the error term, capturing unobserved variation or random fluctuations.

We used the BAPC model as the primary predictive method to estimate the changes in age-standardized prevalence and incidence rates of CRDs over the next 10 years.

Classical APC Model

The APC model was first proposed by Mason in 1973 and can independently evaluate the age effect (Age), period effect (Period), and cohort effect (Cohort). The formula is as follows[22, 23]：

$$Y = \log(M) = \mu + \alpha X_1 + \beta X_2 + \gamma X_3 + \varepsilon$$

Where:

X_1,X_2,X_3 represent age, period, and cohort, respectively;

M is the related disease burden rate;

α、β、γ are the coefficients for the corresponding effects;

μ is the intercept term;

ε is the random error.

To validate the stability of the prediction results, we applied the Norpred APC model to re-predict the overall burden of CRDs and assess the trend for the next 20 years.

12. Decomposition Analysis

Decomposition analysis allows for the quantitative dissection of the factors contributing to changes in an indicator over a given period at global, regional, and national levels. In this study, we adopted the method proposed by Das Gupta et al. to further explore the impact of underlying factors on the disease burden. The changes in disease burden are divided into three aspects: population aging, population growth, and epidemiological changes. The consensus formula for calculation is as follows[14, 24]:

$$A_{a,g,e,t} = \sum_{k=1}^{n} a_{k,t} \cdot p_t \cdot e_{k,t}$$

Where:

A represents the disease burden;

$A_{a,g,e,t}$ represents the disease burden accumulated due to population aging, population growth, and epidemiological changes in year t;

$a_{k,t}$ represents the proportion of the population in age group k in year t;

p_t represents the total population in year t;

$e_{k,t}$ represents the disease burden rate for age group k in year t.

Estimation of Contribution

Unlike traditional linear regression, which only establishes variable relationships, decomposition analysis allows for a detailed evaluation of the independent contributions of each factor to changes in disease burden. By keeping other factors constant, we sequentially calculate the impact of each factor on changes in incidence or DALYs. For example, the contribution of population aging to the change in disease burden in 2021 is calculated using the following formula:

$$Effect_{2021} = \left[\frac{A_{a2021} \cdot p_{2021} \cdot e_{2021} + A_{a2021} \cdot p_{1990} \cdot e_{1990}}{3} + \frac{A_{a2021} \cdot p_{1990} \cdot e_{2021} + A_{a2021} \cdot p_{2021} \cdot e_{1990}}{6}\right] - \left[\frac{A_{a1990} \cdot p_{2021} \cdot e_{2021} + A_{a1990} \cdot p_{1990} \cdot e_{1990}}{3} + \frac{A_{a1990} \cdot p_{1990} \cdot e_{2021} + A_{a1990} \cdot p_{2021} \cdot e_{1990}}{6}\right]$$

This method quantifies the net effect of population aging (or population growth, epidemiological changes) on the overall change in disease burden while keeping other factors constant, thereby providing actionable insights for public health decision-making.

References

[1] Chen Q, Zhang C, Zhong F, et al. Global burden of disease related to tobacco products and trends projected: 1990-2021 [J]. Addictive behaviors, 2025, 169: 108391.

[2] Liu J, Qin Y, Liu H, et al. Global, regional, and national burden of female infertility and trends from 1990 to 2021 with projections to 2050 based on the GBD 2021 analysis [J]. Scientific reports, 2025, 15(1): 17559.

[3] GBD 2021 Diseases and Injuries Collaborators. Global incidence, prevalence, years lived with disability (YLDs), disability-adjusted life-years (DALYs), and healthy life expectancy (HALE) for 371 diseases and injuries in 204 countries and territories and 811 subnational locations, 1990-2021: a systematic analysis for the Global Burden of Disease Study 2021 [J]. Lancet (London, England), 2024, 403(10440): 2133-61.

[4] GBD 2021 Stroke Risk Factor Collaborators. Global, regional, and national burden of stroke and its risk factors, 1990-2021: a systematic analysis for the Global Burden of Disease Study 2021 [J]. The Lancet Neurology, 2024, 23(10): 973-1003.

[5] Shams-Beyranvand M, Farzadfar F, Naderimagham S, et al. Estimation of burden of ischemic heart diseases in Isfahan, Iran, 2014: using incompleteness and misclassification adjustment models [J]. Journal of diabetes and metabolic disorders, 2017, 16: 12.

[6] Liu C, Wang Y, Liu M, et al. Global, regional, and national burden and trends of tension-type headache among adolescents and young adults (15-39 years) from 1990 to 2021: findings from the Global Burden of Disease study 2021 [J]. Scientific reports, 2025, 15(1): 18254.

[7] GBD 2021 Risk Factors Collaborators. Global burden and strength of evidence for 88 risk factors in 204 countries and 811 subnational locations, 1990-2021: a systematic analysis for the Global Burden of Disease Study 2021 [J]. Lancet (London, England), 2024, 403(10440): 2162-203.

[8] GBD 2021 Demographics Collaborators. Global age-sex-specific mortality, life expectancy, and population estimates in 204 countries and territories and 811 subnational locations, 1950-2021, and the impact of the COVID-19 pandemic: a comprehensive demographic analysis for the Global Burden of Disease Study 2021 [J]. Lancet (London, England), 2024, 403(10440): 1989-2056.

[9] GBD 2021 -Fertility and Forecasting Collaborators. Global fertility in 204 countries and territories, 1950-2021, with forecasts to 2100: a comprehensive demographic analysis for the Global Burden of Disease Study 2021 [J]. Lancet (London, England), 2024, 403(10440): 2057-99.

[10] Liu Y, Wen H, Bai J, et al. Disease Burden and Prediction Analysis of Tracheal, Bronchus, and Lung Cancer Attributable to Residential Radon, Solid Fuels, and Particulate Matter Pollution Under Different Sociodemographic Transitions From 1990 to 2030 [J]. Chest, 2024, 165(2): 446-60.

[11] Lu Y, Li H, Dong R, et al. Global and regional disease burden of HIV/AIDS from 1990 to 2021 and projections to 2030 [J]. BMC public health, 2025, 25(1): 1928.

[12] Cen J, Wang Q, Cheng L, et al. Global, regional, and national burden and trends of migraine among women of childbearing age from 1990 to 2021: insights from the Global Burden of Disease Study 2021 [J]. The journal of headache and pain, 2024, 25(1): 96.

[13] Rosenberg P S, Anderson W F. Age-period-cohort models in cancer surveillance research: ready for prime time? [J]. Cancer epidemiology, biomarkers & prevention : a publication of the American Association for Cancer Research, cosponsored by the American Society of Preventive Oncology, 2011, 20(7): 1263-8.

[14] Shi L, Kuang Z, Tu J, et al. Global, regional, and national burden of laryngeal cancer attributable

to smoking, 1990-2021, and projections to 2036: a systematic analysis of the Global Burden of Disease study 2021 [J]. Frontiers in public health, 2025, 13: 1583045.

[15] Lerman P M. Fitting Segmented Regression Models by Grid Search [J]. Journal of the Royal Statistical Society Series C (Applied Statistics), 1980, 29(1): 77-84.

[16] Song C, Deng F, Qiao L, et al. Burdens of idiopathic developmental intellectual disability attributable to lead exposure from 1990 to 2021 and projection to 2035 in China: findings from the 2021 global burden of disease study [J]. Frontiers in public health, 2025, 13: 1562794.

[17] Wang Z, You Q, Wang Y, et al. Global, regional, and national burden of chronic kidney disease among adolescents and emerging adults from 1990 to 2021 [J]. Renal failure, 2025, 47(1): 2508296.

[18] Moreno-Betancur M, Latouche A, Menvielle G, et al. Relative index of inequality and slope index of inequality: a structured regression framework for estimation [J]. Epidemiology (Cambridge, Mass), 2015, 26(4): 518-27.

[19] Wang R, Chen Y, Shao X, et al. Burden of Skin Cancer in Older Adults From 1990 to 2021 and Modelled Projection to 2050 [J]. JAMA dermatology, 2025.

[20] Xu C, Jiang C, Liu X, et al. Epidemiological and sociodemographic transitions in the global burden and risk factors for Alzheimer's disease and other dementias: a secondary analysis of GBD 2021 [J]. International journal for equity in health, 2025, 24(1): 149.

[21] Knoll M, Furkel J, Debus J, et al. An R package for an integrated evaluation of statistical approaches to cancer incidence projection [J]. BMC medical research methodology, 2020, 20(1): 257.

[22] Jiang C Y, Han K, Yang F, et al. Global, regional, and national prevalence of hearing loss from 1990 to 2019: A trend and health inequality analyses based on the Global Burden of Disease Study 2019 [J]. Ageing research reviews, 2023, 92: 102124.

[23] Møller B, Fekjaer H, Hakulinen T, et al. Prediction of cancer incidence in the Nordic countries: empirical comparison of different approaches [J]. Statistics in medicine, 2003, 22(17): 2751-66.

[24] Chevan A, Sutherland M. Revisiting Das Gupta: refinement and extension of standardization and decomposition [J]. Demography, 2009, 46(3): 429-49.
