# Supplementary material for: Global burden of major chronic respiratory diseases among older adults aged 55 and above from 1990 to 2021: Changes, challenges, and predictions amid the pandemic
Source: PLoS One. 2025 Aug 1;20(8):e0329283. doi: 10.1371/journal.pone.0329283 (PMC12316243; doi:10.1371/journal.pone.0329283)
Supplement: S2 File — (PDF) [file pone.0329283.s002.pdf]

Supplementary Material-2

Contents

Table S1 Global Burden of Disease Study risk hierarchy with levels 1

Table S2 Definition of all risk factors 2

Table S3 Comprehensive Overview of the Burden of CRDs, COPD, Asthma, and ILD & PS in the Global, Five SDI Regions, and 21 GBD Regions (EAPC, Time Points: 1990, 2019, 2021) 2

Table S4 Temporal Joinpoint Analysis of CRDs, COPD, Asthma, and ILD & PS in Adults Aged 55 and Above at the Global Level, 1990-2021 16

Table S5 Overview of the Prevalence of CRDs, COPD, Asthma, and ILD & PS Among Adults Aged 55 and Above in 204 Countries Globally (EAPC, Time Points: 1990, 2019, 2021) 22

Table S6 *Net Drift* in the Prevalence of CRDs, COPD, Asthma, and ILD & PS in the Global, 5 SDI Regions, and 21 GBD Regions (1990-2021) 48

Table S7 APC Model Analysis Results of CRDs Prevalence Among Adults Aged 55 and Above in the Global, 5 SDI Regions, and 21 GBD Regions 50

Table S8 APC Model Analysis Results of COPD Prevalence Among Adults Aged 55 and Above in the Global, 5 SDI Regions, and 21 GBD Regions 82

Table S9 APC Model Analysis Results of Asthma Prevalence Among Adults Aged 55 and Above in the Global, 5 SDI Regions, and 21 GBD Regions 114

Table S10 APC Model Analysis Results of ILD&PS Prevalence Among Adults Aged 55 and Above in the Global, 5 SDI Regions, and 21 GBD Regions 146

Table S11 *Net Drift* in the Prevalence of CRDs, COPD, Asthma, and ILD & PS in 11 Example Countries (1990-2021) 178

Table S12 APC Model Analysis Results of CRDs Prevalence Among Adults Aged 55 and Above in 11 Example Countries 179

Table S13 APC Model Analysis Results of COPD Prevalence Among Adults Aged 55 and Above in 11 Example Countries 192

Table S14 APC Model Analysis Results of Asthma Prevalence Among Adults Aged 55 and Above in 11 Example Countries 205

Table S15 APC Model Analysis Results of ILD&PS Prevalence Among Adults Aged 55 and Above in 11 Example Countries 218

Table S16 *Net Drift* in the Incidence of CRDs, COPD, Asthma, and ILD & PS in 11 Example Countries (1990-2021) 231

Table S17 APC Model Analysis Results of CRDs Incidence Among Adults Aged 55 and Above in 11 Example Countries 232

Table S18 APC Model Analysis Results of COPD Incidence Among Adults Aged 55 and Above in 11 Example Countries 245

Table S19 APC Model Analysis Results of Asthma Incidence Among Adults Aged 55 and Above in 11 Example Countries 258

Table S20 APC Model Analysis Results of ILD&PS Incidence Among Adults Aged 55 and Above in 11 Example Countries 271

Table S21 Global Burden Projection of Chronic Respiratory Diseases in Adults Aged 55 and Above, 2022-2031 284

Table S22 Burden of DALYs Attributed to Risk Factors for CRDs, COPD, and Asthma 285

Table S23 Burden of Mortality Attributed to Risk Factors for CRDs, COPD, and Asthma 287

Table S24 Decomposition Analysis of CRDs-Related Burden Globally and by SDI Region, 1990–2021 289

Table S25 Decomposition Analysis of COPD-Related Burden Globally and by SDI Region, 1990–2021 291

Table S26 Decomposition Analysis of Asthma-Related Burden Globally and by SDI Region, 1990–2021 292

Table S27 Decomposition Analysis of ILD & PS-Related Burden Globally and by SDI Region, 1990–2021 293

Fig.S1 Trends in Disease Burden with Changes in SDI at the Global Level, 5 SDI Regions, and 21 GBD Regions 296

Fig.S2 APC Model Analysis Figures for the Prevalence of CRDs, COPD, Asthma, and ILD & PS at the Global Level 297

Fig.S3 APC Model Analysis Figures for the Mortality of CRDs, COPD, Asthma, and ILD & PS at the Global Level 298

Fig.S4 Age and Sex Structure Figures for the Prevalence, Incidence, and DALYs of CRDs, COPD, Asthma, and ILD & PS at the Global Level 299

Fig.S5 Risk Factor Figures for the DALYs Rate of CRDs at the Global Level, 5 SDI Regions, and 21 GBD Regions 300

Fig.S6 Risk Factor Figures for the Mortality Rate of CRDs at the Global Level, 5 SDI Regions, and 21 GBD Regions 301

Fig.S7 Risk Factor Figures for the DALYs Rate of COPD at the Global Level, 5 SDI Regions, and 21 GBD Regions 302

Fig.S8 Risk Factor Figures for the Mortality Rate of COPD at the Global Level, 5 SDI Regions, and 21 GBD Regions 303

Fig.S9 Risk Factor Figures for the DALYs Rate of Asthma at the Global Level, 5 SDI Regions, and 21 GBD Regions 304

Fig.S10 Risk Factor Figures for the Mortality Rate of Asthma at the Global Level, 5 SDI Regions, and 21 GBD Regions 305

Fig.S11 Risk Factor Figures for the DALYs Rate of CRDs in 204 Countries Globally 306

Fig.S12 Risk Factor Figures for the Mortality Rate of CRDs in 204 Countries Globally 307

Fig.S13 Risk Factor Figures for the DALYs Rate of COPD in 204 Countries Globally 308

Fig.S14 Risk Factor Figures for the Mortality Rate of COPD in 204 Countries Globally 309

Fig.S15 Risk Factor Figures for the DALYs Rate of Asthma in 204 Countries Globally 310

Fig.S16 Risk Factor Figures for the Mortality Rate of Asthma in 204 Countries Globally 311

Fig.S17 Decomposition Analysis of CRD-Related Burden Globally and by SDI Region, 1990–2021 312

Table S1 Global Burden of Disease Study risk hierarchy with levels

Risk factor	Level
Environmental/occupational risks	1
Air pollution	2
Particulate matter pollution	3
Ambient particulate matter pollution	4
Household air pollution from solid fuels	4
Ambient ozone pollution	3
Non-optimal temperature	2
High temperature	3
Low temperature	3
Occupational risks	2
Occupational carcinogens	3
Occupational asthmagens	3
Occupational particulate matter, gases, and fumes	3
Behavioral risks	1
Tobacco	2
Smoking	3
Secondhand smoke	3
Metabolic risks	1
High body-mass index	2

Table S2 Definition of all risk factors

All risk factor	Definition
Environmental/occupational risks	
Air pollution	Air pollution includes ambient particulate matter pollution (PM2.5), household air pollution from the use of solid fuels for cooking (HAP), ambient ozone pollution, and nitrogen dioxide pollution.
Particulate matter pollution	Particulate matter pollution includes two sources of PM2.5: ambient particulate matter pollution and household air pollution from the use of solid fuels for cooking, including coal, charcoal, wood, agricultural residue, and animal dung.
Ambient particulate matter pollution	Ambient particulate matter pollution is the population-weighted annual average mass concentration of particles with an aerodynamic diameter less than 2·5 micrometres in a cubic metre of air. The TMREL is a uniform distribution between 2·4 and 5·9 μg/m^3.
Household air pollution from solid fuels	Household air pollution includes exposure to particulate matter less than 2·5 micrometres in diameter (PM2.5) due to the use of solid fuels for cooking, including coal, charcoal, wood, agricultural residue, and animal dung.
Ambient ozone pollution	Ambient ozone pollution is defined as the highest seasonal (six-month) average of eight-hour daily maximum ozone concentrations. The TMREL is a uniform distribution between 29·1 and 35·7 parts per billion (ppb).
Non-optimal temperature	Non-optimal temperature is an aggregate of the burden attributable to low and high temperatures. Heat and cold effects relate to effects above and below the TMREL. The population-weighted mean TMREL is 25·6°C.
High temperature	High temperature is defined as a daily mean temperature warmer than the TMREL, the temperature with the minimum mortality for all included causes. The population-weighted mean TMREL is 25·6°C, with a range of 21·3–26·6°C.
Low temperature	Low temperature is defined as a daily mean temperature colder than the TMREL, defined as the temperature with the minimum mortality for all included causes. The population-weighted mean TMREL is 25·6°C.
Occupational risks	Occupational risks constitute an aggregation of all individual occupational risks modelled as Level 3 risk factors. These include occupational injuries; ergonomic factors; and occupational exposure to particulate matter, fumes and gases, carcinogens, noise, and asthmagens.
Occupational carcinogens	Occupational carcinogens are an aggregate risk, comprising exposure to asbestos, arsenic, benzene, beryllium, cadmium, chromium, diesel engine exhaust, formaldehyde, nickel, polycyclic aromatic hydrocarbons, silica, sulfuric acid, and trichloroethlyene.
Occupational asthmagens	This risk is defined as the proportion of the population occupationally exposed to asthmagens, based on employed population distributions across nine occupational categories. The TMREL category is working in an administrative or managerial occupation.
Occupational particulate matter, gases, and fumes	This risk is defined as the proportion of the population occupationally exposed to particulate matter, gases, and fumes based on population distributions across 17 economic activities. The TMREL is no exposure to particulate matter, gases, and fumes.
Behavioral risks	
Tobacco	Tobacco includes tobacco smoking, chewing tobacco use, and secondhand smoke exposure.
Smoking	Smoking is defined as current daily or occasional use of any smoked tobacco product.
Secondhand smoke	This risk factor refers to any current exposure to secondhand tobacco smoke at home or at work. Only non-daily smokers are considered to be exposed to secondhand smoke.
Metabolic risks	
High body-mass index	High BMI for adults (ages 20 and older) is defined as BMI greater than 20–23 kg/m2 . High BMI for children (ages 2–19) is defined as being overweight or obese based on International Obesity Task Force standards.

Table S3 Comprehensive Overview of the Burden of CRDs, COPD, Asthma, and ILD & PS in the Global, Five SDI Regions, and 21 GBD Regions (EAPC, Time Points: 1990, 2019,

2021)

CRDs		Cases			EAPC	
Measure	Location	2019_millions(95% UI)	2021_millions(95% UI)	% change	2019-2021(95% CI)	1990-2021(95% CI)
Prevalence	Global	211.38 (195.67-228.54)	223.17 (206.5-241.48)	0.06 (0.06-0.06)	-0.05 (-0.19-0.08)	-0.71 (-0.76--0.65)
	Low SDI	10.36 (9.57-11.17)	11.04 (10.16-11.94)	0.07 (0.06-0.07)	0.45 (0.44-0.46)	-0.2 (-0.22--0.18)
	Low-middle SDI	34.66 (31.88-37.38)	36.72 (33.81-39.61)	0.06 (0.06-0.06)	0.23 (0.16-0.31)	-0.1 (-0.12--0.07)
	Middle SDI	56.61 (51.25-62.25)	61 (55.26-67.17)	0.08 (0.08-0.08)	-0.01 (-0.14-0.13)	-0.4 (-0.43--0.37)
	High-middle SDI	44.27 (40.36-48.46)	46.71 (42.61-51.12)	0.06 (0.06-0.05)	0.01 (-0.02-0.05)	-1.05 (-1.09--1)
	High SDI	65.29 (61.7-69.16)	67.51 (63.87-71.52)	0.03 (0.04-0.03)	0.02 (-0.23-0.27)	-0.82 (-0.97--0.67)
	Andean Latin America	0.92 (0.83-1.02)	0.93 (0.82-1.03)	0.01 (-0.01-0.01)	-1.82 (-3.45--0.16)	0.33 (0.27-0.39)
	Australasia	1.25 (1.16-1.34)	1.29 (1.19-1.41)	0.03 (0.03-0.05)	-1.03 (-2.25-0.2)	-1.55 (-1.63--1.47)
	Caribbean	1.05 (0.97-1.14)	1.09 (1.01-1.18)	0.04 (0.04-0.04)	-0.5 (-0.96--0.04)	0.03 (-0.03-0.08)
	Central Asia	1.7 (1.57-1.83)	1.81 (1.66-1.96)	0.06 (0.06-0.07)	-0.11 (-0.3-0.09)	-0.67 (-0.73--0.6)
	Central Europe	6.38 (5.91-6.9)	6.46 (5.96-7.01)	0.01 (0.01-0.02)	0.58 (0.51-0.66)	-1.12 (-1.28--0.96)
	Central Latin America	4.73 (4.27-5.21)	4.98 (4.5-5.47)	0.05 (0.05-0.05)	-0.3 (-0.67-0.07)	-0.38 (-0.41--0.34)
	Central Sub-Saharan Africa	0.86 (0.78-0.96)	0.93 (0.84-1.03)	0.08 (0.08-0.07)	0.62 (0.6-0.64)	-0.22 (-0.28--0.16)
	East Asia	45.9 (41.11-51.04)	49.62 (44.32-55.1)	0.08 (0.08-0.08)	-0.26 (-0.38--0.14)	-0.63 (-0.69--0.58)
	Eastern Europe	6.69 (5.95-7.46)	6.88 (6.1-7.69)	0.03 (0.03-0.03)	1.31 (0.84-1.78)	-1.85 (-1.95--1.75)
	Eastern Sub-Saharan Africa	2.51 (2.28-2.77)	2.69 (2.44-2.96)	0.07 (0.07-0.07)	0.72 (0.72-0.73)	-0.45 (-0.51--0.4)
	High-income Asia Pacific	9.2 (8.31-10.12)	9.5 (8.59-10.45)	0.03 (0.03-0.03)	0.4 (-0.16-0.97)	-1.95 (-2.17--1.74)
	High-income North America	28.41 (27.07-29.79)	29.45 (27.91-31.02)	0.04 (0.03-0.04)	0.13 (-0.04-0.31)	0.31 (0.16-0.47)
	North Africa and Middle East	10.12 (9.33-11.04)	10.94 (10.04-11.93)	0.08 (0.08-0.08)	0.31 (-0.19-0.81)	-0.26 (-0.3--0.23)
	Oceania	0.15 (0.14-0.16)	0.16 (0.15-0.18)	0.07 (0.07-0.12)	-0.14 (-0.15--0.14)	-0.87 (-0.93--0.81)
	South Asia	39.03 (35.77-42.1)	41.4 (38.01-44.65)	0.06 (0.06-0.06)	0.19 (0.12-0.27)	-0.01 (-0.06-0.03)
	Southeast Asia	12.61 (11.48-13.84)	13.66 (12.42-15)	0.08 (0.08-0.08)	0.39 (0.02-0.76)	-0.65 (-0.71--0.6)
	Southern Latin America	2.29 (2.11-2.47)	2.41 (2.22-2.59)	0.05 (0.05-0.05)	0.55 (0.04-1.07)	-0.82 (-0.91--0.72)
	Southern Sub-Saharan Africa	1 (0.89-1.11)	1.03 (0.93-1.15)	0.03 (0.04-0.04)	0.03 (-0.22-0.29)	-0.37 (-0.46--0.27)
	Tropical Latin America	5.51 (4.91-6.13)	5.96 (5.28-6.62)	0.08 (0.08-0.08)	0.89 (-0.35-2.13)	-0.5 (-0.6--0.4)
	Western Europe	27.84 (25.94-30.05)	28.46 (26.52-30.59)	0.02 (0.02-0.02)	-0.4 (-0.55--0.25)	-1.07 (-1.19--0.95)
	Western Sub-Saharan Africa	3.23 (2.93-3.55)	3.52 (3.2-3.88)	0.09 (0.09-0.09)	1.2 (1.15-1.26)	-0.25 (-0.33--0.18)
Measure						
Incidence	Global	17.39 (15.95-18.93)	18.47 (16.97-20.11)	0.06 (0.06-0.06)	0.26 (0.02-0.49)	-0.59 (-0.64--0.54)
	Low SDI	0.93 (0.85-1.03)	0.99 (0.9-1.09)	0.06 (0.06-0.06)	0.33 (0.16-0.5)	-0.21 (-0.25--0.18)
	Low-middle SDI	3.21 (2.95-3.49)	3.4 (3.12-3.69)	0.06 (0.06-0.06)	0.15 (0.14-0.15)	-0.27 (-0.31--0.24)

	Middle SDI	5.19 (4.72-5.7)	5.6 (5.11-6.15)	0.08 (0.08-0.08)	0.13 (0.12-0.13)	-0.67 (-0.72--0.63)
	High-middle SDI	3.54 (3.23-3.86)	3.76 (3.43-4.11)	0.06 (0.06-0.06)	0.29 (0.19-0.39)	-1 (-1.06--0.93)
	High SDI	4.5 (4.12-4.88)	4.7 (4.31-5.12)	0.04 (0.05-0.05)	0.59 (-0.33-1.5)	-0.43 (-0.58--0.28)
	Andean Latin America	0.08 (0.07-0.09)	0.08 (0.07-0.09)	0 (0-0)	-1.07 (-2--0.13)	0.47 (0.42-0.52)
	Australasia	0.07 (0.07-0.08)	0.08 (0.07-0.09)	0.14 (0-0.12)	-0.38 (-1.05-0.3)	-0.6 (-0.65--0.55)
	Caribbean	0.08 (0.07-0.08)	0.08 (0.07-0.09)	0 (0-0.12)	-0.02 (-0.41-0.37)	0.27 (0.21-0.32)
	Central Asia	0.14 (0.13-0.16)	0.15 (0.14-0.17)	0.07 (0.08-0.06)	0.35 (0.16-0.53)	-0.4 (-0.44--0.36)
	Central Europe	0.46 (0.41-0.51)	0.47 (0.42-0.52)	0.02 (0.02-0.02)	0.7 (0.3-1.11)	-1.07 (-1.23--0.91)
	Central Latin America	0.4 (0.37-0.44)	0.43 (0.39-0.47)	0.07 (0.05-0.07)	-0.01 (-0.36-0.35)	-0.52 (-0.55--0.48)
	Central Sub-Saharan Africa	0.08 (0.07-0.09)	0.08 (0.07-0.09)	0 (0-0)	0.39 (0.23-0.55)	-0.22 (-0.3--0.15)
	East Asia	4.33 (3.93-4.77)	4.69 (4.25-5.14)	0.08 (0.08-0.08)	-0.17 (-0.26--0.08)	-1.1 (-1.19--1)
	Eastern Europe	0.45 (0.39-0.51)	0.46 (0.41-0.53)	0.02 (0.05-0.04)	1.73 (1.07-2.4)	-2.14 (-2.27--2)
	Eastern Sub-Saharan Africa	0.2 (0.18-0.23)	0.22 (0.19-0.24)	0.1 (0.06-0.04)	0.39 (-0.35-1.13)	-0.43 (-0.47--0.39)
	High-income Asia Pacific	0.79 (0.7-0.87)	0.81 (0.73-0.9)	0.03 (0.04-0.03)	0.4 (-0.14-0.94)	-1.61 (-1.87--1.35)
	High-income North America	1.89 (1.72-2.09)	2 (1.81-2.22)	0.06 (0.05-0.06)	1.19 (-1.45-3.91)	0.41 (0.23-0.6)
	North Africa and Middle East	0.83 (0.74-0.93)	0.9 (0.8-1)	0.08 (0.08-0.08)	0.3 (0.3-0.31)	-0.11 (-0.14--0.09)
	Oceania	0.02 (0.02-0.02)	0.02 (0.02-0.02)	0 (0-0)	-0.23 (-0.32--0.14)	-0.75 (-0.8--0.69)
	South Asia	3.71 (3.42-4.03)	3.94 (3.62-4.28)	0.06 (0.06-0.06)	0.14 (0.12-0.16)	-0.28 (-0.33--0.24)
	Southeast Asia	1.16 (1.04-1.29)	1.26 (1.12-1.4)	0.09 (0.08-0.09)	0.53 (0.33-0.74)	-0.66 (-0.71--0.61)
	Southern Latin America	0.15 (0.14-0.17)	0.16 (0.14-0.17)	0.07 (0-0)	0.26 (-0.41-0.93)	-0.16 (-0.26--0.06)
	Southern Sub-Saharan Africa	0.08 (0.07-0.09)	0.09 (0.08-0.1)	0.12 (0.14-0.11)	0.09 (-0.09-0.27)	-0.49 (-0.59--0.39)
	Tropical Latin America	0.44 (0.39-0.49)	0.48 (0.42-0.53)	0.09 (0.08-0.08)	0.73 (0.2-1.26)	-0.7 (-0.8--0.59)
	Western Europe	1.75 (1.6-1.9)	1.79 (1.65-1.95)	0.02 (0.03-0.03)	-0.32 (-0.54--0.11)	-0.34 (-0.43--0.26)
	Western Sub-Saharan Africa	0.27 (0.23-0.31)	0.3 (0.26-0.34)	0.11 (0.13-0.1)	1.41 (1.13-1.69)	-0.17 (-0.24--0.1)
Measure						
Deaths	Global	4.03 (3.68-4.35)	4.15 (3.76-4.58)	0.03 (0.02-0.05)	-1.29 (-1.57--1.01)	-1.4 (-1.5--1.3)
	Low SDI	0.32 (0.28-0.36)	0.32 (0.28-0.37)	0 (0-0.03)	-2.96 (-3.32--2.59)	-0.29 (-0.39--0.18)
	Low-middle SDI	1.16 (1.03-1.29)	1.16 (1.03-1.3)	0 (0-0.01)	-2.68 (-2.85--2.51)	-0.11 (-0.19--0.02)
	Middle SDI	1.31 (1.17-1.46)	1.39 (1.21-1.57)	0.06 (0.03-0.08)	-0.93 (-1.35--0.51)	-2.43 (-2.57--2.29)
	High-middle SDI	0.69 (0.6-0.77)	0.72 (0.62-0.82)	0.04 (0.03-0.06)	-0.36 (-0.45--0.28)	-2.65 (-2.9--2.4)
	High SDI	0.55 (0.48-0.59)	0.56 (0.49-0.6)	0.02 (0.02-0.02)	-0.32 (-1.33-0.7)	-0.55 (-0.64--0.46)
	Andean Latin America	0.01 (0.01-0.02)	0.01 (0.01-0.02)	0 (0-0)	-6.5 (-7.4--5.59)	0.44 (0.28-0.6)
	Australasia	0.01 (0.01-0.01)	0.01 (0.01-0.02)	0 (0-1)	-0.94 (-6.43-4.88)	-1.03 (-1.24--0.81)

	Caribbean	0.01 (0.01-0.01)	0.01 (0.01-0.01)	0 (0-0)	-1.08 (-1.73--0.42)	0.15 (-0.01-0.3)
	Central Asia	0.02 (0.02-0.02)	0.02 (0.02-0.02)	0 (0-0)	-2.3 (-3.04--1.56)	-2.59 (-2.87--2.32)
	Central Europe	0.04 (0.04-0.04)	0.04 (0.04-0.04)	0 (0-0)	0.04 (-0.99-1.08)	-1.54 (-1.74--1.34)
	Central Latin America	0.07 (0.06-0.07)	0.07 (0.06-0.08)	0 (0-0.14)	-0.88 (-1.88-0.13)	-0.78 (-0.89--0.67)
	Central Sub-Saharan Africa	0.02 (0.01-0.03)	0.02 (0.01-0.03)	0 (0-0)	-1.32 (-1.47--1.18)	-0.85 (-1--0.7)
	East Asia	1.25 (1.07-1.47)	1.34 (1.1-1.59)	0.07 (0.03-0.08)	-0.72 (-1.08--0.37)	-3.46 (-3.71--3.19)
	Eastern Europe	0.04 (0.04-0.04)	0.04 (0.04-0.05)	0 (0-0.25)	-0.83 (-1.12--0.55)	-4.35 (-4.68--4.01)
	Eastern Sub-Saharan Africa	0.04 (0.04-0.06)	0.05 (0.04-0.06)	0.25 (0-0)	-1.47 (-1.83--1.11)	-1.35 (-1.42--1.29)
	High-income Asia Pacific	0.07 (0.06-0.08)	0.08 (0.06-0.08)	0.14 (0-0)	1.62 (-1.41-4.75)	-0.55 (-0.82--0.29)
	High-income North America	0.23 (0.21-0.25)	0.24 (0.21-0.26)	0.04 (0-0.04)	0.16 (-0.78-1.11)	0.16 (-0.08-0.41)
	North Africa and Middle East	0.11 (0.1-0.12)	0.11 (0.1-0.12)	0 (0-0)	-2.61 (-3.26--1.95)	-1.25 (-1.31--1.19)
	Oceania	0.01 (0.01-0.01)	0.01 (0.01-0.01)	0 (0-0)	-1.46 (-2.19--0.73)	-0.78 (-0.87--0.69)
	South Asia	1.43 (1.27-1.62)	1.43 (1.27-1.63)	0 (0-0.01)	-2.7 (-2.82--2.58)	-0.09 (-0.18-0)
	Southeast Asia	0.25 (0.23-0.28)	0.27 (0.24-0.3)	0.08 (0.04-0.07)	-0.95 (-2.13-0.23)	-1.39 (-1.6--1.18)
	Southern Latin America	0.03 (0.02-0.03)	0.03 (0.02-0.03)	0 (0-0)	-4.85 (-5.04--4.65)	0.29 (-0.07-0.65)
	Southern Sub-Saharan Africa	0.02 (0.02-0.02)	0.02 (0.02-0.02)	0 (0-0)	-1.68 (-2.71--0.65)	-0.7 (-1.14--0.25)
	Tropical Latin America	0.07 (0.06-0.07)	0.07 (0.06-0.07)	0 (0-0)	-2.03 (-3.5--0.54)	-1.5 (-1.78--1.22)
	Western Europe	0.25 (0.21-0.26)	0.24 (0.21-0.26)	-0.04 (0-0)	-2.03 (-2.95--1.09)	-0.29 (-0.4--0.17)
	Western Sub-Saharan Africa	0.04 (0.04-0.05)	0.04 (0.04-0.05)	0 (0-0)	-2.42 (-2.52--2.31)	-1.1 (-1.18--1.03)
Measure						
DALY	Global	81.19 (76.08-86.79)	83.67 (77.49-90.36)	0.03 (0.02-0.04)	-1.25 (-1.59--0.92)	-1.59 (-1.67--1.5)
	Low SDI	6.93 (6.19-7.8)	6.97 (6.19-7.95)	0.01 (0-0.02)	-2.41 (-2.46--2.36)	-0.58 (-0.65--0.51)
	Low-middle SDI	24.19 (21.82-26.54)	24.24 (21.68-26.79)	0 (-0.01-0.01)	-2.52 (-2.83--2.21)	-0.43 (-0.49--0.37)
	Middle SDI	25.33 (23.04-28.04)	26.79 (24.05-29.87)	0.06 (0.04-0.07)	-0.95 (-1.34--0.55)	-2.6 (-2.73--2.48)
	High-middle SDI	12.9 (11.59-14.2)	13.52 (12.15-15.03)	0.05 (0.05-0.06)	-0.34 (-0.47--0.21)	-2.87 (-3.08--2.65)
	High SDI	11.79 (10.86-12.57)	12.11 (11.15-12.93)	0.03 (0.03-0.03)	-0.32 (-1.06-0.43)	-0.84 (-0.92--0.76)
	Andean Latin America	0.26 (0.22-0.29)	0.24 (0.21-0.29)	-0.08 (-0.05-0)	-4.42 (-4.87--3.98)	0.06 (-0.06-0.18)
	Australasia	0.26 (0.23-0.27)	0.26 (0.24-0.28)	0 (0.04-0.04)	-1.05 (-4.71-2.75)	-1.6 (-1.8--1.41)
	Caribbean	0.25 (0.22-0.28)	0.25 (0.22-0.29)	0 (0-0.04)	-0.99 (-1.36--0.61)	0 (-0.12-0.12)
	Central Asia	0.39 (0.37-0.42)	0.41 (0.37-0.45)	0.05 (0-0.07)	-1.4 (-1.73--1.07)	-2.47 (-2.76--2.19)
	Central Europe	0.98 (0.92-1.04)	0.99 (0.91-1.06)	0.01 (-0.01-0.02)	0.12 (-0.26-0.5)	-1.65 (-1.84--1.47)
	Central Latin America	1.21 (1.13-1.27)	1.28 (1.16-1.39)	0.06 (0.03-0.09)	-0.08 (-0.23-0.08)	-1.04 (-1.13--0.94)
	Central Sub-Saharan Africa	0.48 (0.35-0.76)	0.51 (0.37-0.79)	0.06 (0.06-0.04)	-0.66 (-0.68--0.64)	-0.87 (-0.97--0.77)

East Asia	22.22 (19.17-25.87)	23.68 (20.18-27.81)	0.07 (0.05-0.07)	-0.96 (-1.31--0.61)	-3.72 (-3.95--3.49)
Eastern Europe	1.07 (1.01-1.14)	1.07 (0.99-1.15)	0 (-0.02-0.01)	-0.24 (-0.72-0.24)	-4.14 (-4.47--3.81)
Eastern Sub-Saharan Africa	1.08 (0.94-1.29)	1.13 (0.97-1.34)	0.05 (0.03-0.04)	-0.8 (-0.9--0.69)	-1.33 (-1.39--1.27)
High-income Asia Pacific	1.45 (1.29-1.59)	1.51 (1.34-1.65)	0.04 (0.04-0.04)	0.63 (-0.93-2.22)	-1.35 (-1.56--1.14)
High-income North America	5.46 (5.04-5.81)	5.67 (5.25-6.02)	0.04 (0.04-0.04)	0.19 (-0.42-0.8)	-0.01 (-0.12-0.1)
North Africa and Middle East	2.44 (2.27-2.64)	2.54 (2.32-2.79)	0.04 (0.02-0.06)	-1.53 (-1.84--1.21)	-1.38 (-1.42--1.34)
Oceania	0.15 (0.12-0.17)	0.15 (0.13-0.19)	0 (0.08-0.12)	-1.01 (-1.5--0.52)	-0.91 (-0.95--0.86)
South Asia	29.98 (27.19-33.39)	30.07 (27.03-33.73)	0 (-0.01-0.01)	-2.57 (-2.88--2.26)	-0.44 (-0.5--0.38)
Southeast Asia	5.47 (4.95-6.01)	5.81 (5.24-6.41)	0.06 (0.06-0.07)	-0.63 (-1.58-0.33)	-1.43 (-1.59--1.27)
Southern Latin America	0.51 (0.47-0.54)	0.49 (0.45-0.53)	-0.04 (-0.04--0.02)	-3.51 (-3.97--3.05)	-0.3 (-0.53--0.06)
Southern Sub-Saharan Africa	0.5 (0.47-0.53)	0.51 (0.48-0.55)	0.02 (0.02-0.04)	-0.7 (-1.39-0)	-0.46 (-0.87--0.06)
Tropical Latin America	1.32 (1.23-1.4)	1.38 (1.27-1.46)	0.05 (0.03-0.04)	-1.05 (-2.06--0.03)	-1.68 (-1.93--1.42)
Western Europe	4.64 (4.25-4.94)	4.61 (4.21-4.93)	-0.01 (-0.01-0)	-1.75 (-1.78--1.72)	-0.86 (-0.97--0.74)
Western Sub-Saharan Africa	1.08 (0.95-1.23)	1.12 (0.99-1.29)	0.04 (0.04-0.05)	-1.29 (-1.42--1.17)	-0.97 (-1.03--0.91)

COPD		Cases			EAPC	
Measure	Location	2019_millions(95% UI)	2021_millions(95% UI)	% change	2019-2021(95% CI)	1990-2021(95% CI)
Prevalence	Global	158.41 (141.7-175.05)	166.94 (148.84-184.74)	0.05 (0.05-0.06)	-0.14 (-0.33-0.05)	0.18 (0.13-0.23)
	Low SDI	7.06 (6.31-7.86)	7.49 (6.67-8.35)	0.06 (0.06-0.06)	0.19 (0.15-0.23)	0.38 (0.34-0.42)
	Low-middle SDI	25.62 (22.82-28.48)	26.98 (24.07-29.86)	0.05 (0.05-0.05)	-0.07 (-0.14--0.01)	0.35 (0.3-0.4)
	Middle SDI	45.52 (39.82-51.32)	48.89 (42.61-55.28)	0.07 (0.07-0.08)	-0.17 (-0.38-0.03)	0.24 (0.19-0.28)
	High-middle SDI	35.45 (31.37-39.73)	37.36 (32.91-42.05)	0.05 (0.05-0.06)	-0.04 (-0.08-0)	0.06 (-0.01-0.12)
	High SDI	44.62 (41.15-48.16)	46.08 (42.41-49.65)	0.03 (0.03-0.03)	-0.04 (-0.44-0.36)	0.23 (0.17-0.28)
	Andean Latin America	0.75 (0.65-0.85)	0.75 (0.64-0.85)	0 (-0.02-0)	-2.25 (-4.22--0.23)	0.68 (0.59-0.77)
	Australasia	0.75 (0.66-0.84)	0.76 (0.67-0.88)	0.01 (0.02-0.05)	-1.84 (-3.99-0.37)	-0.73 (-0.84--0.62)
	Caribbean	0.77 (0.69-0.85)	0.79 (0.71-0.88)	0.03 (0.03-0.04)	-0.74 (-1.3--0.17)	0.78 (0.69-0.87)
	Central Asia	1.19 (1.06-1.34)	1.25 (1.11-1.41)	0.05 (0.05-0.05)	-0.58 (-0.76--0.4)	-0.1 (-0.2--0.01)
	Central Europe	4.4 (3.96-4.85)	4.47 (3.99-4.95)	0.02 (0.01-0.02)	0.7 (0.45-0.95)	0.57 (0.55-0.6)
	Central Latin America	4.08 (3.61-4.58)	4.29 (3.79-4.78)	0.05 (0.05-0.04)	-0.38 (-0.79-0.04)	0.52 (0.43-0.62)
	Central Sub-Saharan Africa	0.58 (0.5-0.67)	0.63 (0.54-0.73)	0.09 (0.08-0.09)	0.72 (0.54-0.91)	0.26 (0.21-0.32)
	East Asia	39.51 (34.69-44.83)	42.58 (37.16-48.21)	0.08 (0.07-0.08)	-0.4 (-0.59--0.21)	-0.07 (-0.14--0.01)
	Eastern Europe	5.59 (4.8-6.36)	5.77 (4.94-6.59)	0.03 (0.03-0.04)	1.53 (0.97-2.09)	-0.36 (-0.44--0.28)
	Eastern Sub-Saharan Africa	1.54 (1.33-1.76)	1.66 (1.43-1.9)	0.08 (0.08-0.08)	0.99 (0.95-1.03)	0.12 (0.05-0.18)

	High-income Asia Pacific	6.46 (5.55-7.42)	6.66 (5.74-7.68)	0.03 (0.03-0.04)	0.28 (-0.57-1.14)	0.77 (0.71-0.83)
	High-income North America	18.49 (17.51-19.31)	19.24 (18.19-20.17)	0.04 (0.04-0.04)	0.29 (0.04-0.54)	0.2 (0.06-0.34)
	North Africa and Middle East	6.98 (6.16-7.89)	7.5 (6.63-8.51)	0.07 (0.08-0.08)	0.03 (-0.59-0.64)	0.75 (0.68-0.83)
	Oceania	0.1 (0.09-0.11)	0.11 (0.1-0.12)	0.1 (0.11-0.09)	-0.09 (-0.19-0.02)	-0.31 (-0.36--0.26)
	South Asia	29.65 (26.58-32.79)	31.24 (28.03-34.62)	0.05 (0.05-0.06)	-0.14 (-0.27--0.01)	0.42 (0.36-0.47)
	Southeast Asia	8.69 (7.53-9.86)	9.43 (8.15-10.72)	0.09 (0.08-0.09)	0.44 (-0.14-1.03)	0 (-0.07-0.07)
	Southern Latin America	1.03 (0.9-1.17)	1.06 (0.93-1.21)	0.03 (0.03-0.03)	-0.61 (-1.3-0.08)	0.16 (0.04-0.29)
	Southern Sub-Saharan Africa	0.78 (0.68-0.89)	0.82 (0.7-0.93)	0.05 (0.03-0.04)	0.13 (-0.15-0.4)	-0.06 (-0.1--0.01)
	Tropical Latin America	4.66 (4.04-5.3)	5.04 (4.37-5.73)	0.08 (0.08-0.08)	0.89 (-0.61-2.42)	0.14 (0.07-0.21)
	Western Europe	20.51 (18.44-22.64)	20.85 (18.75-22.93)	0.02 (0.02-0.01)	-0.67 (-0.9--0.44)	0.34 (0.31-0.36)
	Western Sub-Saharan Africa	1.89 (1.62-2.18)	2.05 (1.76-2.36)	0.08 (0.09-0.08)	0.82 (0.61-1.02)	0.41 (0.38-0.45)
Measure						
Incidence	Global	12.35 (11.15-13.47)	13.07 (11.76-14.25)	0.06 (0.05-0.06)	0.09 (-0.04-0.21)	0.07 (0.03-0.11)
	Low SDI	0.57 (0.52-0.63)	0.61 (0.55-0.66)	0.07 (0.06-0.05)	0.13 (0.12-0.14)	0.23 (0.2-0.26)
	Low-middle SDI	2.16 (1.96-2.34)	2.28 (2.06-2.47)	0.06 (0.05-0.06)	-0.04 (-0.15-0.08)	0.14 (0.09-0.19)
	Middle SDI	3.81 (3.39-4.2)	4.11 (3.65-4.53)	0.08 (0.08-0.08)	-0.01 (-0.14-0.11)	-0.05 (-0.1--0.01)
	High-middle SDI	2.74 (2.43-3.03)	2.9 (2.56-3.21)	0.06 (0.05-0.06)	0.23 (0.22-0.24)	-0.15 (-0.21--0.09)
	High SDI	3.06 (2.8-3.32)	3.17 (2.9-3.47)	0.04 (0.04-0.05)	0.21 (-0.07-0.5)	0.32 (0.28-0.35)
	Andean Latin America	0.06 (0.05-0.07)	0.06 (0.05-0.07)	0 (0-0)	-1.34 (-2.49--0.18)	0.83 (0.75-0.92)
	Australasia	0.05 (0.05-0.06)	0.06 (0.05-0.06)	0.2 (0-0)	-0.95 (-2.06-0.18)	-0.64 (-0.7--0.59)
	Caribbean	0.05 (0.05-0.06)	0.06 (0.05-0.06)	0.2 (0-0)	-0.38 (-0.56--0.2)	0.83 (0.75-0.91)
	Central Asia	0.09 (0.08-0.1)	0.1 (0.08-0.11)	0.11 (0-0.1)	-0.12 (-0.23--0.01)	0.09 (0-0.17)
	Central Europe	0.29 (0.26-0.33)	0.3 (0.26-0.33)	0.03 (0-0)	0.79 (0.58-1)	0.57 (0.54-0.6)
	Central Latin America	0.33 (0.3-0.37)	0.35 (0.32-0.39)	0.06 (0.07-0.05)	-0.04 (-0.47-0.39)	0.58 (0.5-0.66)
	Central Sub-Saharan Africa	0.04 (0.04-0.05)	0.05 (0.04-0.06)	0.25 (0-0.2)	0.47 (0.04-0.9)	0.16 (0.1-0.22)
	East Asia	3.48 (3.12-3.85)	3.76 (3.36-4.15)	0.08 (0.08-0.08)	-0.32 (-0.33--0.3)	-0.51 (-0.6--0.42)
	Eastern Europe	0.35 (0.3-0.41)	0.37 (0.31-0.42)	0.06 (0.03-0.02)	2.09 (1.31-2.89)	-0.75 (-0.84--0.67)
	Eastern Sub-Saharan Africa	0.11 (0.09-0.13)	0.12 (0.1-0.13)	0.09 (0.11-0)	0.76 (0.74-0.78)	-0.07 (-0.13-0)
	High-income Asia Pacific	0.52 (0.45-0.6)	0.53 (0.46-0.61)	0.02 (0.02-0.02)	0.06 (-1.15-1.28)	0.73 (0.69-0.77)
	High-income North America	1.16 (1.08-1.23)	1.22 (1.13-1.29)	0.05 (0.05-0.05)	0.65 (0.61-0.69)	0.43 (0.33-0.53)
	North Africa and Middle East	0.49 (0.42-0.55)	0.52 (0.46-0.59)	0.06 (0.1-0.07)	0.22 (-0.38-0.82)	0.9 (0.85-0.96)
	Oceania	0.01 (0.01-0.01)	0.01 (0.01-0.01)	0 (0-0)	-0.14 (-0.14--0.14)	-0.3 (-0.35--0.25)
	South Asia	2.57 (2.34-2.78)	2.71 (2.47-2.93)	0.05 (0.06-0.05)	-0.05 (-0.21-0.12)	0.08 (0.04-0.13)

	Southeast Asia	0.69 (0.59-0.78)	0.75 (0.65-0.85)	0.09 (0.1-0.09)	0.72 (0.29-1.14)	-0.06 (-0.13-0)
	Southern Latin America	0.08 (0.07-0.09)	0.09 (0.08-0.1)	0.12 (0.14-0.11)	-0.81 (-1.57--0.04)	0.32 (0.19-0.45)
	Southern Sub-Saharan Africa	0.06 (0.05-0.07)	0.06 (0.05-0.07)	0 (0-0)	0.26 (0.25-0.27)	-0.12 (-0.17--0.07)
	Tropical Latin America	0.35 (0.3-0.4)	0.38 (0.33-0.43)	0.09 (0.1-0.07)	0.67 (0.04-1.32)	-0.06 (-0.12-0.01)
	Western Europe	1.44 (1.3-1.58)	1.47 (1.34-1.62)	0.02 (0.03-0.03)	-0.43 (-0.65--0.22)	0.36 (0.34-0.38)
	Western Sub-Saharan Africa	0.12 (0.1-0.14)	0.13 (0.11-0.16)	0.08 (0.1-0.14)	0.86 (0.55-1.17)	0.38 (0.35-0.4)
Measure						
Deaths	Global	3.46 (3.15-3.73)	3.59 (3.22-3.94)	0.04 (0.02-0.06)	-1.04 (-1.32--0.77)	-1.46 (-1.57--1.35)
	Low SDI	0.25 (0.22-0.28)	0.25 (0.22-0.28)	0 (0-0)	-2.76 (-3.21--2.32)	-0.08 (-0.19-0.03)
	Low-middle SDI	0.95 (0.85-1.04)	0.95 (0.85-1.05)	0 (0-0.01)	-2.43 (-2.58--2.27)	0.05 (-0.04-0.13)
	Middle SDI	1.18 (1.05-1.33)	1.25 (1.08-1.43)	0.06 (0.03-0.08)	-0.75 (-1.19--0.32)	-2.51 (-2.65--2.36)
	High-middle SDI	0.64 (0.56-0.72)	0.67 (0.58-0.76)	0.05 (0.04-0.06)	-0.23 (-0.28--0.18)	-2.68 (-2.94--2.41)
	High SDI	0.45 (0.39-0.48)	0.46 (0.4-0.49)	0.02 (0.03-0.02)	-0.23 (-1.22-0.77)	-0.58 (-0.67--0.48)
	Andean Latin America	0.01 (0.01-0.01)	0.01 (0.01-0.01)	0 (0-0)	-6.41 (-7.49--5.31)	-0.3 (-0.44--0.16)
	Australasia	0.01 (0.01-0.01)	0.01 (0.01-0.01)	0 (0-0)	-0.89 (-6.5-5.06)	-1.27 (-1.49--1.05)
	Caribbean	0.01 (0.01-0.01)	0.01 (0.01-0.01)	0 (0-0)	-1.06 (-1.69--0.42)	0.42 (0.24-0.6)
	Central Asia	0.01 (0.01-0.01)	0.01 (0.01-0.01)	0 (0-0)	-2.32 (-2.37--2.26)	-2.27 (-2.52--2.03)
	Central Europe	0.04 (0.03-0.04)	0.04 (0.03-0.04)	0 (0-0)	0.03 (-1.02-1.09)	-1.23 (-1.4--1.06)
	Central Latin America	0.06 (0.05-0.06)	0.06 (0.05-0.07)	0 (0-0.17)	-0.74 (-1.76-0.29)	-0.6 (-0.75--0.46)
	Central Sub-Saharan Africa	0.01 (0.01-0.02)	0.01 (0.01-0.02)	0 (0-0)	-1.01 (-1.14--0.87)	-0.76 (-0.91--0.61)
	East Asia	1.21 (1.04-1.42)	1.3 (1.06-1.54)	0.07 (0.02-0.08)	-0.68 (-1.03--0.32)	-3.47 (-3.74--3.21)
	Eastern Europe	0.04 (0.04-0.04)	0.04 (0.04-0.04)	0 (0-0)	-0.87 (-1.11--0.64)	-4.05 (-4.37--3.72)
	Eastern Sub-Saharan Africa	0.03 (0.03-0.04)	0.03 (0.03-0.04)	0 (0-0)	-1.29 (-1.69--0.88)	-1.27 (-1.34--1.21)
	High-income Asia Pacific	0.04 (0.03-0.05)	0.04 (0.04-0.05)	0 (0.33-0)	2.12 (-0.93-5.26)	-0.45 (-0.7--0.2)
	High-income North America	0.2 (0.18-0.21)	0.21 (0.18-0.22)	0.05 (0-0.05)	0.26 (-0.8-1.32)	0.07 (-0.18-0.32)
	North Africa and Middle East	0.08 (0.07-0.09)	0.08 (0.07-0.09)	0 (0-0)	-2.45 (-3.26--1.63)	-0.72 (-0.81--0.63)
	Oceania	0.01 (0-0.01)	0.01 (0-0.01)	0 (NaN-0)	-1.36 (-2.19--0.53)	-0.75 (-0.83--0.67)
	South Asia	1.17 (1.06-1.3)	1.18 (1.06-1.32)	0.01 (0-0.02)	-2.44 (-2.53--2.35)	0.05 (-0.04-0.14)
	Southeast Asia	0.2 (0.18-0.22)	0.21 (0.19-0.24)	0.05 (0.06-0.09)	-0.73 (-2-0.55)	-1.19 (-1.4--0.98)
	Southern Latin America	0.02 (0.02-0.02)	0.02 (0.02-0.02)	0 (0-0)	-4.99 (-5--4.98)	0.11 (-0.26-0.48)
	Southern Sub-Saharan Africa	0.02 (0.01-0.02)	0.02 (0.01-0.02)	0 (0-0)	-1.49 (-2.44--0.52)	-0.52 (-0.93--0.11)
	Tropical Latin America	0.06 (0.05-0.06)	0.06 (0.05-0.07)	0 (0-0.17)	-2.01 (-3.34--0.65)	-1.61 (-1.89--1.32)
	Western Europe	0.2 (0.18-0.22)	0.2 (0.18-0.22)	0 (0-0)	-1.95 (-2.87--1.01)	-0.28 (-0.36--0.2)

	Western Sub-Saharan Africa	0.03 (0.03-0.03)	0.03 (0.03-0.03)	0 (0-0)	-2.28 (-2.43--2.13)	-0.84 (-0.94--0.75)
Measure						
DALY	Global	67.93 (63.15-72.58)	70.28 (64.85-76)	0.03 (0.03-0.05)	-1.06 (-1.41--0.71)	-1.6 (-1.7--1.5)
	Low SDI	5.34 (4.81-5.89)	5.39 (4.87-6)	0.01 (0.01-0.02)	-2.25 (-2.35--2.15)	-0.36 (-0.43--0.29)
	Low-middle SDI	19.38 (17.75-21.18)	19.51 (17.77-21.32)	0.01 (0-0.01)	-2.31 (-2.61--2)	-0.26 (-0.33--0.2)
	Middle SDI	22.31 (20.13-24.79)	23.64 (21.06-26.47)	0.06 (0.05-0.07)	-0.83 (-1.24--0.42)	-2.68 (-2.81--2.55)
	High-middle SDI	11.64 (10.43-12.9)	12.22 (10.94-13.7)	0.05 (0.05-0.06)	-0.24 (-0.41--0.07)	-2.85 (-3.07--2.62)
	High SDI	9.22 (8.53-9.78)	9.48 (8.71-10.05)	0.03 (0.02-0.03)	-0.25 (-1.04-0.54)	-0.65 (-0.72--0.58)
	Andean Latin America	0.13 (0.12-0.16)	0.13 (0.11-0.15)	0 (-0.08--0.06)	-4.37 (-4.79--3.96)	-0.53 (-0.63--0.43)
	Australasia	0.19 (0.18-0.21)	0.2 (0.18-0.21)	0.05 (0-0)	-1.12 (-5.06-2.98)	-1.69 (-1.9--1.47)
	Caribbean	0.2 (0.18-0.22)	0.21 (0.18-0.23)	0.05 (0-0.05)	-1.03 (-1.37--0.7)	0.37 (0.22-0.53)
	Central Asia	0.3 (0.28-0.32)	0.31 (0.28-0.34)	0.03 (0-0.06)	-1.49 (-1.99--0.98)	-2.15 (-2.41--1.9)
	Central Europe	0.82 (0.78-0.87)	0.83 (0.77-0.89)	0.01 (-0.01-0.02)	0.12 (-0.32-0.56)	-1.15 (-1.29--1.01)
	Central Latin America	1.02 (0.96-1.08)	1.08 (0.98-1.19)	0.06 (0.02-0.1)	0.06 (-0.07-0.19)	-0.81 (-0.94--0.67)
	Central Sub-Saharan Africa	0.34 (0.25-0.47)	0.36 (0.26-0.5)	0.06 (0.04-0.06)	-0.32 (-0.34--0.31)	-0.74 (-0.84--0.64)
	East Asia	21.14 (18.28-24.67)	22.54 (19.22-26.51)	0.07 (0.05-0.07)	-0.94 (-1.29--0.58)	-3.75 (-3.99--3.52)
	Eastern Europe	0.97 (0.91-1.02)	0.96 (0.89-1.04)	-0.01 (-0.02-0.02)	-0.26 (-0.73-0.22)	-3.73 (-4.06--3.4)
	Eastern Sub-Saharan Africa	0.8 (0.66-0.92)	0.83 (0.69-0.96)	0.04 (0.05-0.04)	-0.59 (-0.72--0.46)	-1.21 (-1.28--1.15)
	High-income Asia Pacific	0.88 (0.77-0.97)	0.91 (0.8-1.01)	0.03 (0.04-0.04)	0.82 (-0.48-2.14)	-0.49 (-0.63--0.34)
	High-income North America	4.43 (4.1-4.68)	4.61 (4.24-4.87)	0.04 (0.03-0.04)	0.32 (-0.45-1.09)	-0.11 (-0.28-0.05)
	North Africa and Middle East	1.84 (1.7-1.99)	1.92 (1.73-2.11)	0.04 (0.02-0.06)	-1.45 (-1.87--1.02)	-0.84 (-0.89--0.79)
	Oceania	0.11 (0.09-0.13)	0.12 (0.1-0.14)	0.09 (0.11-0.08)	-0.9 (-1.48--0.31)	-0.88 (-0.92--0.83)
	South Asia	24.19 (21.93-26.49)	24.37 (22.12-27.05)	0.01 (0.01-0.02)	-2.34 (-2.63--2.05)	-0.29 (-0.35--0.23)
	Southeast Asia	4.28 (3.88-4.73)	4.56 (4.11-5.08)	0.07 (0.06-0.07)	-0.43 (-1.45-0.6)	-1.22 (-1.38--1.05)
	Southern Latin America	0.36 (0.34-0.39)	0.35 (0.32-0.37)	-0.03 (-0.06--0.05)	-4.15 (-4.81--3.48)	-0.29 (-0.56--0.02)
	Southern Sub-Saharan Africa	0.36 (0.34-0.39)	0.37 (0.34-0.4)	0.03 (0-0.03)	-0.5 (-1.09-0.1)	-0.26 (-0.62-0.1)
	Tropical Latin America	1.16 (1.07-1.22)	1.21 (1.11-1.28)	0.04 (0.04-0.05)	-1.05 (-1.9--0.19)	-1.71 (-1.97--1.46)
	Western Europe	3.69 (3.38-3.92)	3.67 (3.34-3.9)	-0.01 (-0.01--0.01)	-1.82 (-1.84--1.79)	-0.53 (-0.61--0.45)
	Western Sub-Saharan Africa	0.72 (0.64-0.8)	0.75 (0.67-0.84)	0.04 (0.05-0.05)	-1.14 (-1.28--0.99)	-0.66 (-0.73--0.59)

Asthma		Cases			EAPC	
Measure	Location	2019_TenThousands(95% UI)	2021_TenThousands(95% UI)	% change	2019-2021(95% CI)	1990-2021(95% CI)
Prevalence	Global	5814.97 (5126.28-6527.97)	6168.93 (5423.24-6941.08)	0.06 (0.06-0.06)	0.19 (0.15-0.22)	-2.57 (-2.76--2.37)
	Low SDI	358.45 (313.22-410.92)	386.65 (336.99-443.64)	0.08 (0.08-0.08)	1.05 (0.92-1.18)	-1.17 (-1.23--1.12)

	Low-middle SDI	1000.94 (864.74-1149.19)	1079.29 (934.55-1237.93)	0.08 (0.08-0.08)	1.11 (0.95-1.28)	-1.11 (-1.2--1.03)
	Middle SDI	1193.69 (1042.65-1358.52)	1305.26 (1137.28-1488.12)	0.09 (0.09-0.1)	0.73 (0.63-0.83)	-2.34 (-2.44--2.23)
	High-middle SDI	953.57 (848.12-1072.82)	1010.22 (898.99-1132.03)	0.06 (0.06-0.06)	0.22 (0.2-0.24)	-3.79 (-3.96--3.63)
	High SDI	2302.1 (2057.06-2562.85)	2381.1 (2114.65-2670.11)	0.03 (0.03-0.04)	0.03 (0-0.07)	-2.4 (-2.8--1.99)
	Andean Latin America	13.16 (11.35-15.12)	13.8 (11.99-16.09)	0.05 (0.06-0.06)	0.11 (-0.39-0.61)	-1.69 (-1.74--1.64)
	Australasia	52.81 (48.02-57.7)	55.58 (50.2-61.08)	0.05 (0.05-0.06)	-0.05 (-0.06--0.04)	-2.7 (-2.87--2.53)
	Caribbean	30.27 (27.39-33.68)	31.83 (28.79-35.45)	0.05 (0.05-0.05)	0.1 (-0.14-0.33)	-1.46 (-1.54--1.37)
	Central Asia	53.96 (47.7-61.83)	58.52 (51.28-67)	0.08 (0.08-0.08)	0.96 (0.69-1.23)	-1.81 (-1.96--1.66)
	Central Europe	218.91 (189.6-249.61)	220.89 (192.28-252.58)	0.01 (0.01-0.01)	0.45 (0.14-0.75)	-3.39 (-3.68--3.1)
	Central Latin America	64.52 (56.59-73.79)	68.62 (60.32-78.05)	0.06 (0.07-0.06)	0.2 (0.05-0.34)	-3.83 (-3.98--3.67)
	Central Sub-Saharan Africa	29.42 (25.29-34.41)	31.77 (27.36-37.26)	0.08 (0.08-0.08)	0.43 (-0.01-0.87)	-1.04 (-1.13--0.96)
	East Asia	682.37 (594.42-776.14)	750.62 (654.69-855.43)	0.1 (0.1-0.1)	0.62 (0.38-0.86)	-3.16 (-3.33--2.99)
	Eastern Europe	117.14 (101.25-136.56)	118.13 (102.1-137.84)	0.01 (0.01-0.01)	0.32 (0.23-0.42)	-5.56 (-5.79--5.33)
	Eastern Sub-Saharan Africa	102.58 (89.47-119.17)	109.28 (94.78-126.03)	0.07 (0.06-0.06)	0.37 (0.3-0.44)	-1.2 (-1.26--1.14)
	High-income Asia Pacific	263.71 (229.32-298.74)	274.81 (240.11-309.66)	0.04 (0.05-0.04)	0.87 (0.73-1.02)	-5.42 (-5.74--5.1)
	High-income North America	1156.46 (1016.48-1297.21)	1187.61 (1031.57-1351.78)	0.03 (0.01-0.04)	-0.34 (-0.46--0.23)	0.77 (0-1.54)
	North Africa and Middle East	349.27 (310.56-394.06)	381.91 (339.3-433.97)	0.09 (0.09-0.1)	0.89 (0.6-1.19)	-1.77 (-1.86--1.68)
	Oceania	5.35 (4.82-6.03)	5.74 (5.19-6.49)	0.07 (0.08-0.08)	-0.31 (-0.5--0.13)	-1.88 (-1.97--1.78)
	South Asia	1048.65 (898.55-1216.89)	1136.62 (972.71-1317.09)	0.08 (0.08-0.08)	1.29 (1.28-1.29)	-1.07 (-1.18--0.96)
	Southeast Asia	424.02 (368.98-484.02)	458.6 (399.6-526.52)	0.08 (0.08-0.09)	0.31 (0.28-0.35)	-1.8 (-1.88--1.72)
	Southern Latin America	130.83 (116.37-148.15)	139.96 (124.58-156.56)	0.07 (0.07-0.06)	1.49 (0.94-2.04)	-1.55 (-1.69--1.41)
	Southern Sub-Saharan Africa	21.51 (18.73-24.79)	22.19 (19.41-25.57)	0.03 (0.04-0.03)	-0.33 (-0.48--0.17)	-1.39 (-1.66--1.12)
	Tropical Latin America	92.84 (80.53-106.84)	100.82 (87.83-115.66)	0.09 (0.09-0.08)	1.05 (0.97-1.13)	-2.87 (-3.15--2.59)
	Western Europe	815.58 (728.74-907.16)	845.19 (756.5-939.25)	0.04 (0.04-0.04)	0.28 (0.24-0.32)	-3.46 (-3.68--3.23)
	Western Sub-Saharan Africa	141.62 (123.43-163.73)	156.43 (136.02-180.39)	0.1 (0.1-0.1)	1.81 (1.4-2.23)	-1.01 (-1.13--0.89)
Measure						
Incidence	Global	472.19 (388.12-571.36)	506.33 (415.33-612.42)	0.07 (0.07-0.07)	0.73 (-0.49-1.96)	-1.96 (-2.11--1.81)
	Low SDI	34.96 (28.25-43)	37.42 (30.13-46.16)	0.07 (0.07-0.07)	0.67 (0.22-1.11)	-0.85 (-0.9--0.8)
	Low-middle SDI	100.44 (81.57-122.03)	107.12 (86.93-131.58)	0.07 (0.07-0.08)	0.56 (0.34-0.78)	-1.04 (-1.11--0.98)
	Middle SDI	130.32 (107.03-156.21)	142.01 (117.21-169.58)	0.09 (0.1-0.09)	0.56 (0.21-0.9)	-2.09 (-2.19--2)
	High-middle SDI	75.47 (62.68-90.13)	80.41 (66.76-95.62)	0.07 (0.07-0.06)	0.51 (0.08-0.94)	-3.13 (-3.29--2.96)
	High SDI	130.52 (105.02-162.98)	138.88 (111.62-173.3)	0.06 (0.06-0.06)	1.46 (-2.37-5.44)	-1.84 (-2.27--1.4)
	Andean Latin America	1.33 (1.11-1.58)	1.4 (1.16-1.68)	0.05 (0.05-0.06)	0.16 (-0.17-0.5)	-1.54 (-1.6--1.48)

	Australasia	1.58<br>(1.25-1.93)	1.71<br>(1.39-2.08)	0.08<br>(0.11-0.08)	1.31<br>(0.59-2.03)	-0.85<br>(-1.13--0.57)
	Caribbean	2.29<br>(1.87-2.78)	2.44<br>(2.01-2.96)	0.07<br>(0.07-0.06)	0.81<br>(-0.05-1.68)	-0.79<br>(-0.86--0.73)
	Central Asia	5.19<br>(4.18-6.33)	5.65<br>(4.64-6.95)	0.09<br>(0.11-0.1)	1.16<br>(0.84-1.48)	-1.15<br>(-1.28--1.02)
	Central Europe	16.43<br>(13.21-20.52)	16.61<br>(13.36-20.47)	0.01<br>(0.01-0)	0.56<br>(-0.17-1.3)	-2.92<br>(-3.18--2.66)
	Central Latin America	6.06<br>(5.01-7.21)	6.45<br>(5.37-7.66)	0.06<br>(0.07-0.06)	0.18<br>(0.18-0.19)	-3.88<br>(-4.05--3.71)
	Central Sub-Saharan Africa	3<br>(2.37-3.78)	3.23<br>(2.58-4.05)	0.08<br>(0.09-0.07)	0.28<br>(-0.74-1.32)	-0.72<br>(-0.82--0.62)
	East Asia	79.12<br>(64.28-95.5)	86.8<br>(70.73-103.71)	0.1<br>(0.1-0.09)	0.48<br>(0.07-0.91)	-2.98<br>(-3.12--2.84)
	Eastern Europe	9.31<br>(7.26-11.7)	9.4<br>(7.31-11.76)	0.01<br>(0.01-0.01)	0.38<br>(0.18-0.58)	-5.01<br>(-5.26--4.77)
	Eastern Sub-Saharan Africa	9.16<br>(7.22-11.5)	9.68<br>(7.6-12.21)	0.06<br>(0.05-0.06)	-0.04<br>(-1.68-1.62)	-0.81<br>(-0.85--0.77)
	High-income Asia Pacific	22.85<br>(18.99-27.11)	23.98<br>(19.91-28.5)	0.05<br>(0.05-0.05)	1.23<br>(0.36-2.11)	-4.56<br>(-5.04--4.08)
	High-income North America	67.61<br>(52.61-85.96)	72.93<br>(55.97-93.82)	0.08<br>(0.06-0.09)	2.13<br>(-5.27-10.11)	0.44<br>(-0.22-1.11)
	North Africa and Middle East	33.92<br>(28.22-41.24)	36.74<br>(30-45.27)	0.08<br>(0.06-0.1)	0.43<br>(-0.45-1.32)	-1.23<br>(-1.28--1.17)
	Oceania	0.65<br>(0.55-0.8)	0.7<br>(0.58-0.84)	0.08<br>(0.05-0.05)	-0.39<br>(-0.63--0.15)	-1.37<br>(-1.44--1.31)
	South Asia	108.25<br>(87.1-131.55)	115.72<br>(92.99-142.22)	0.07<br>(0.07-0.08)	0.59<br>(0.13-1.04)	-1.05<br>(-1.15--0.94)
	Southeast Asia	46.09<br>(37.7-55.24)	49.79<br>(41.06-60.07)	0.08<br>(0.09-0.09)	0.26<br>(0.13-0.38)	-1.41<br>(-1.47--1.36)
	Southern Latin America	6.14<br>(5.02-7.7)	6.59<br>(5.37-8.17)	0.07<br>(0.07-0.06)	1.66<br>(1-2.33)	-0.89<br>(-1--0.77)
	Southern Sub-Saharan Africa	2.29<br>(1.87-2.77)	2.36<br>(1.94-2.83)	0.03<br>(0.04-0.02)	-0.31<br>(-0.92-0.31)	-1.25<br>(-1.51--0.98)
	Tropical Latin America	8.65<br>(6.92-10.6)	9.39<br>(7.57-11.46)	0.09<br>(0.09-0.08)	0.99<br>(0.85-1.13)	-2.6<br>(-2.85--2.35)
	Western Europe	27.77<br>(21.08-36.12)	28.73<br>(21.8-37.77)	0.03<br>(0.03-0.05)	0.18<br>(-0.07-0.44)	-2.84<br>(-3.15--2.54)
	Western Sub-Saharan Africa	14.49<br>(11.61-17.96)	16.03<br>(12.77-19.87)	0.11<br>(0.1-0.11)	1.89<br>(1.12-2.67)	-0.57<br>(-0.69--0.46)
Measure						
Deaths	Global	35.55<br>(28.58-47.23)	35.1<br>(28.35-45.22)	-0.01<br>(-0.01--0.04)	-3.33<br>(-3.58--3.08)	-1.8<br>(-1.88--1.72)
	Low SDI	5.62<br>(3.89-8.69)	5.47<br>(3.85-8.32)	-0.03<br>(-0.01--0.04)	-3.98<br>(-4.01--3.96)	-1.2<br>(-1.31--1.09)
	Low-middle SDI	17.32<br>(12.47-25.75)	16.76<br>(12.14-24.33)	-0.03<br>(-0.03--0.06)	-4.21<br>(-4.47--3.95)	-1.03<br>(-1.13--0.93)
	Middle SDI	9.07<br>(8.18-10.3)	9.26<br>(8.25-10.39)	0.02<br>(0.01-0.01)	-2.65<br>(-2.96--2.34)	-2.43<br>(-2.6--2.25)
	High-middle SDI	2.06<br>(1.85-2.26)	2.1<br>(1.87-2.32)	0.02<br>(0.01-0.03)	-1.79<br>(-1.85--1.73)	-3.99<br>(-4.22--3.76)
	High SDI	1.46<br>(1.24-1.62)	1.5<br>(1.27-1.68)	0.03<br>(0.02-0.04)	-0.44<br>(-1.92-1.07)	-5.51<br>(-5.96--5.05)
	Andean Latin America	0.04<br>(0.03-0.05)	0.03<br>(0.03-0.04)	-0.25<br>(0--0.2)	-6.46<br>(-7.09--5.84)	-3.89<br>(-4--3.77)
	Australasia	0.05<br>(0.04-0.05)	0.05<br>(0.04-0.05)	0<br>(0-0)	-1.84<br>(-7.43-4.09)	-4.16<br>(-5.01--3.3)
	Caribbean	0.11<br>(0.09-0.16)	0.12<br>(0.09-0.16)	0.09<br>(0-0)	-1.44<br>(-2.63--0.22)	-2.07<br>(-2.26--1.89)
	Central Asia	0.27<br>(0.24-0.3)	0.27<br>(0.23-0.32)	0<br>(-0.04-0.07)	-2.28<br>(-2.87--1.67)	-3.99<br>(-4.46--3.51)
	Central Europe	0.17<br>(0.16-0.19)	0.17<br>(0.16-0.19)	0<br>(0-0)	-0.47<br>(-0.93--0.01)	-5.72<br>(-6.26--5.18)
	Central Latin America	0.19<br>(0.17-0.2)	0.19<br>(0.17-0.21)	0<br>(0-0.05)	-1.98<br>(-2.95--1)	-6.02<br>(-6.22--5.83)

	Central Sub-Saharan Africa	0.49 (0.27-1.16)	0.5 (0.29-1.17)	0.02 (0.07-0.01)	-2.34 (-2.5--2.18)	-1.2 (-1.39--1.01)
	East Asia	2.52 (2.1-3.01)	2.6 (2.08-3.11)	0.03 (-0.01-0.03)	-2.72 (-3.1--2.34)	-3.98 (-4.26--3.7)
	Eastern Europe	0.13 (0.12-0.14)	0.13 (0.12-0.14)	0 (0-0)	-0.17 (-1.39-1.07)	-8.35 (-8.85--7.84)
	Eastern Sub-Saharan Africa	0.94 (0.66-1.57)	0.95 (0.66-1.59)	0.01 (0-0.01)	-2.3 (-2.52--2.07)	-1.76 (-1.83--1.7)
	High-income Asia Pacific	0.37 (0.28-0.46)	0.4 (0.31-0.5)	0.08 (0.11-0.09)	3.38 (-0.07-6.95)	-6.9 (-7.43--6.37)
	High-income North America	0.25 (0.22-0.27)	0.26 (0.23-0.28)	0.04 (0.05-0.04)	-0.68 (-1.7-0.35)	-4.09 (-4.44--3.74)
	North Africa and Middle East	2.02 (1.77-2.27)	2.02 (1.76-2.29)	0 (-0.01-0.01)	-3.49 (-3.61--3.37)	-3.04 (-3.16--2.92)
	Oceania	0.14 (0.1-0.22)	0.15 (0.1-0.22)	0.07 (0-0)	-1.92 (-2.3--1.53)	-1.02 (-1.17--0.88)
	South Asia	20.39 (14.1-31.82)	19.66 (13.86-29.57)	-0.04 (-0.02--0.07)	-4.46 (-4.71--4.21)	-0.98 (-1.09--0.87)
	Southeast Asia	4.88 (4.31-5.48)	5.05 (4.44-5.74)	0.03 (0.03-0.05)	-1.89 (-2.56--1.21)	-2.15 (-2.38--1.93)
	Southern Latin America	0.07 (0.06-0.07)	0.06 (0.06-0.07)	-0.14 (0-0)	-4.97 (-6.22--3.69)	-3.15 (-3.51--2.78)
	Southern Sub-Saharan Africa	0.53 (0.47-0.58)	0.52 (0.46-0.58)	-0.02 (-0.02-0)	-2.57 (-3.78--1.36)	-1.18 (-1.73--0.62)
	Tropical Latin America	0.2 (0.18-0.21)	0.2 (0.18-0.21)	0 (0-0)	-2.99 (-3.69--2.28)	-3.26 (-3.6--2.92)
	Western Europe	0.63 (0.53-0.68)	0.61 (0.51-0.67)	-0.03 (-0.04--0.01)	-2.91 (-4.07--1.74)	-4.81 (-5.4--4.22)
	Western Sub-Saharan Africa	1.15 (0.94-1.42)	1.15 (0.93-1.43)	0 (-0.01-0.01)	-3.01 (-3.03--2.98)	-1.72 (-1.78--1.66)
Measure						
DALY	Global	904.35 (733.91-1159.83)	908.29 (745.46-1127.68)	0 (0.02--0.03)	-2.52 (-2.83--2.2)	-2.19 (-2.26--2.13)
	Low SDI	129.51 (93.34-191.11)	128.17 (93.51-185.68)	-0.01 (0--0.03)	-3.21 (-3.36--3.05)	-1.49 (-1.58--1.41)
	Low-middle SDI	382.51 (282.58-559.82)	373.77 (281.24-528.83)	-0.02 (0--0.06)	-3.74 (-4.13--3.36)	-1.35 (-1.41--1.28)
	Middle SDI	214.83 (190.45-244.19)	223 (197.01-255.57)	0.04 (0.03-0.05)	-1.86 (-2.2--1.51)	-2.59 (-2.73--2.45)
	High-middle SDI	70.16 (57.69-86.56)	72.89 (59.63-88.74)	0.04 (0.03-0.03)	-0.76 (-0.96--0.55)	-4.23 (-4.44--4.03)
	High SDI	106.66 (77.93-141.03)	109.77 (79.27-144.97)	0.03 (0.02-0.03)	-0.22 (-0.56-0.13)	-3.51 (-3.9--3.12)
	Andean Latin America	1.1 (0.88-1.36)	1.09 (0.86-1.38)	-0.01 (-0.02-0.01)	-2.65 (-2.77--2.53)	-3.29 (-3.39--3.19)
	Australasia	2.63 (1.99-3.41)	2.74 (2.07-3.55)	0.04 (0.04-0.04)	-0.61 (-1.9-0.7)	-3.49 (-3.77--3.21)
	Caribbean	3.45 (2.82-4.69)	3.56 (2.88-4.88)	0.03 (0.02-0.04)	-0.87 (-1.54--0.19)	-1.91 (-2.04--1.77)
	Central Asia	7.58 (6.59-8.63)	7.91 (6.73-9.26)	0.04 (0.02-0.07)	-0.97 (-1.8--0.14)	-3.65 (-4.1--3.21)
	Central Europe	10.97 (8.13-14.48)	10.99 (8.18-14.51)	0 (0.01-0)	0.04 (-0.18-0.27)	-4.33 (-4.7--3.96)
	Central Latin America	5.68 (4.84-6.72)	5.95 (5.04-7.12)	0.05 (0.04-0.06)	-0.6 (-0.66--0.54)	-5.32 (-5.51--5.13)
	Central Sub-Saharan Africa	12.03 (7.24-25.74)	12.45 (7.67-26.04)	0.03 (0.06-0.01)	-1.7 (-1.81--1.6)	-1.33 (-1.47--1.2)
	East Asia	65.45 (53.67-80.05)	68.89 (55.16-83.25)	0.05 (0.03-0.04)	-1.58 (-1.96--1.19)	-4.08 (-4.29--3.87)
	Eastern Europe	6.89 (5.37-8.75)	6.92 (5.41-8.7)	0 (0.01--0.01)	0.06 (-0.5-0.62)	-7.23 (-7.61--6.85)
	Eastern Sub-Saharan Africa	24.17 (17.86-36.73)	24.76 (18.3-37.56)	0.02 (0.02-0.02)	-1.59 (-1.62--1.56)	-1.81 (-1.87--1.75)
	High-income Asia Pacific	14.11 (10.58-18.32)	14.83 (11.27-19.14)	0.05 (0.07-0.04)	1.29 (0.32-2.27)	-6.42 (-6.81--6.04)

	High-income North America	46.98 (32.5-64.38)	48.13 (33-66.78)	0.02 (0.02-0.04)	-0.46 (-0.51--0.42)	-0.18 (-0.83-0.47)
	North Africa and Middle East	50.16 (44.04-57.75)	51.79 (45.02-60.14)	0.03 (0.02-0.04)	-1.96 (-1.99--1.93)	-3.05 (-3.16--2.93)
	Oceania	3.08 (2.11-4.69)	3.23 (2.23-4.93)	0.05 (0.06-0.05)	-1.48 (-1.62--1.34)	-1.19 (-1.28--1.1)
	South Asia	446.54 (319.72-685.4)	434.32 (318.53-640.63)	-0.03 (0--0.07)	-4.05 (-4.46--3.64)	-1.36 (-1.43--1.29)
	Southeast Asia	110.53 (97.33-126.32)	115.46 (101.49-130.78)	0.04 (0.04-0.04)	-1.41 (-2.04--0.78)	-2.23 (-2.4--2.06)
	Southern Latin America	5.93 (4.3-8.14)	6.2 (4.5-8.51)	0.05 (0.05-0.05)	0.31 (-0.08-0.7)	-2.13 (-2.31--1.96)
	Southern Sub-Saharan Africa	11.55 (10.51-12.67)	11.61 (10.49-12.92)	0.01 (0-0.02)	-1.61 (-2.57--0.65)	-1.04 (-1.58--0.49)
	Tropical Latin America	6.94 (5.7-8.39)	7.28 (5.98-8.91)	0.05 (0.05-0.06)	-0.68 (-0.97--0.39)	-3.38 (-3.69--3.07)
	Western Europe	38.97 (29.1-51.59)	39.77 (29.6-52.49)	0.02 (0.02-0.02)	-0.49 (-0.54--0.44)	-4.08 (-4.37--3.79)
	Western Sub-Saharan Africa	29.57 (24.63-35.62)	30.41 (24.89-36.95)	0.03 (0.01-0.04)	-1.76 (-1.84--1.67)	-1.66 (-1.72--1.6)

ILD&PS		Cases			EAPC	
Measure	Location	2019_TenThousands(95% UI)	2021_TenThousands(95% UI)	% change	2019-2021(95% CI)	1990-2021(95% CI)
Prevalence	Global	314.42 (275.96-358.12)	331.47 (289.75-378.73)	0.05 (0.05-0.06)	-0.13 (-0.13--0.12)	0.68 (0.58-0.79)
	Low SDI	8.33 (7.12-9.7)	8.8 (7.54-10.22)	0.06 (0.06-0.05)	0.04 (-0.28-0.35)	0.2 (0.16-0.24)
	Low-middle SDI	38.22 (32.8-44.29)	40.12 (34.39-46.47)	0.05 (0.05-0.05)	-0.23 (-0.57-0.1)	0.49 (0.46-0.53)
	Middle SDI	59.81 (51.62-68.83)	64.08 (55.34-73.81)	0.07 (0.07-0.07)	-0.29 (-0.51--0.08)	0.94 (0.83-1.05)
	High-middle SDI	51.04 (45.19-57.78)	53.85 (47.48-60.98)	0.06 (0.05-0.06)	0.02 (-0.22-0.25)	0.92 (0.76-1.09)
	High SDI	156.87 (137.95-177.31)	164.46 (143.56-186.79)	0.05 (0.04-0.05)	0.71 (0.47-0.96)	0.95 (0.84-1.06)
	Andean Latin America	6.3 (5.76-6.81)	6.46 (5.93-6.95)	0.03 (0.03-0.02)	-0.98 (-1.11--0.86)	2.7 (2.53-2.87)
	Australasia	2.76 (2.49-3.09)	2.95 (2.65-3.3)	0.07 (0.06-0.07)	0.66 (0.49-0.84)	2.08 (1.87-2.28)
	Caribbean	0.69 (0.62-0.77)	0.72 (0.64-0.8)	0.04 (0.03-0.04)	-0.26 (-0.36--0.16)	1.32 (1.2-1.44)
	Central Asia	1.69 (1.5-1.88)	1.81 (1.61-2.03)	0.07 (0.07-0.08)	0.5 (0.47-0.53)	0.09 (-0.11-0.29)
	Central Europe	4.56 (4.05-5.13)	4.57 (4.07-5.16)	0 (0-0.01)	0.11 (-0.15-0.38)	0.26 (0.17-0.35)
	Central Latin America	8.05 (7.06-9.11)	8.52 (7.48-9.65)	0.06 (0.06-0.06)	-0.09 (-0.1--0.07)	0.58 (0.5-0.66)
	Central Sub-Saharan Africa	0.56 (0.48-0.65)	0.61 (0.52-0.7)	0.09 (0.08-0.08)	0.63 (0.31-0.95)	0.34 (0.22-0.46)
	East Asia	46.86 (39.81-55.01)	50.73 (43.28-59.59)	0.08 (0.09-0.08)	-0.18 (-0.65-0.29)	1.27 (1-1.54)
	Eastern Europe	2.82 (2.37-3.32)	2.82 (2.36-3.31)	0 (0-0)	-0.09 (-0.35-0.17)	-2.97 (-3.11--2.83)
	Eastern Sub-Saharan Africa	1.36 (1.16-1.59)	1.45 (1.23-1.7)	0.07 (0.06-0.07)	0.42 (0.27-0.57)	0.37 (0.34-0.4)
	High-income Asia Pacific	55.27 (48.01-63.3)	56.95 (49.6-65.24)	0.03 (0.03-0.03)	0.31 (-0.06-0.68)	1.1 (0.96-1.23)
	High-income North America	61.37 (54.04-69.11)	65.55 (56.55-74.93)	0.07 (0.05-0.08)	1.63 (0.82-2.45)	0.32 (0.17-0.47)
	North Africa and Middle East	9.32 (8.17-10.57)	10.15 (8.87-11.5)	0.09 (0.09-0.09)	0.69 (0.64-0.75)	1.51 (1.43-1.59)
	Oceania	0.17 (0.15-0.19)	0.18 (0.16-0.2)	0.06 (0.07-0.05)	0.59 (0.18-1)	0.33 (0.28-0.38)

	South Asia	50.99 (43.19-59.63)	53.75 (45.53-62.71)	0.05 (0.05-0.05)	-0.12 (-0.6-0.36)	0.37 (0.34-0.4)
	Southeast Asia	7.64 (6.48-8.95)	8.33 (7.05-9.71)	0.09 (0.09-0.08)	0.72 (0.71-0.72)	0.92 (0.89-0.96)
	Southern Latin America	6.75 (6.16-7.33)	7.07 (6.46-7.68)	0.05 (0.05-0.05)	0.43 (0.29-0.57)	1.97 (1.81-2.14)
	Southern Sub-Saharan Africa	1.55 (1.32-1.81)	1.61 (1.36-1.88)	0.04 (0.03-0.04)	0.01 (-0.37-0.39)	-0.43 (-0.6--0.27)
	Tropical Latin America	3.6 (3.08-4.17)	3.81 (3.26-4.39)	0.06 (0.06-0.05)	-0.26 (-0.67-0.16)	-0.56 (-0.68--0.43)
	Western Europe	40.76 (36.62-45.34)	41.98 (37.62-46.6)	0.03 (0.03-0.03)	-0.03 (-0.19-0.14)	1.44 (1.24-1.64)
	Western Sub-Saharan Africa	1.35 (1.16-1.58)	1.44 (1.23-1.69)	0.07 (0.06-0.07)	0.11 (-0.16-0.37)	-0.51 (-0.59--0.43)
Measure						
Incidence	Global	27.1 (23.44-31.21)	28.49 (24.48-32.88)	0.05 (0.04-0.05)	-0.27 (-0.5--0.04)	1.21 (1.07-1.35)
	Low SDI	1 (0.84-1.18)	1.06 (0.89-1.24)	0.06 (0.06-0.05)	-0.08 (-0.09--0.07)	0.32 (0.28-0.35)
	Low-middle SDI	4.48 (3.77-5.25)	4.69 (3.94-5.51)	0.05 (0.05-0.05)	-0.36 (-0.48--0.23)	0.54 (0.51-0.57)
	Middle SDI	5.69 (4.83-6.62)	6.08 (5.16-7.07)	0.07 (0.07-0.07)	-0.4 (-0.44--0.37)	1.46 (1.33-1.59)
	High-middle SDI	3.76 (3.26-4.31)	3.97 (3.43-4.55)	0.06 (0.05-0.06)	0.05 (-0.18-0.29)	1.72 (1.51-1.93)
	High SDI	12.16 (10.58-13.91)	12.68 (10.96-14.58)	0.04 (0.04-0.05)	0.44 (-0.07-0.94)	1.56 (1.38-1.74)
	Andean Latin America	0.98 (0.91-1.05)	1 (0.93-1.07)	0.02 (0.02-0.02)	-1.14 (-1.58--0.7)	2.55 (2.38-2.72)
	Australasia	0.28 (0.25-0.31)	0.3 (0.27-0.33)	0.07 (0.08-0.06)	0.77 (0.73-0.8)	2.58 (2.36-2.8)
	Caribbean	0.06 (0.05-0.07)	0.06 (0.06-0.07)	0 (0.2-0)	-0.29 (-0.51--0.07)	1.33 (1.23-1.43)
	Central Asia	0.16 (0.14-0.18)	0.17 (0.15-0.19)	0.06 (0.07-0.06)	0.37 (0.2-0.54)	0.06 (-0.1-0.22)
	Central Europe	0.24 (0.2-0.28)	0.24 (0.2-0.28)	0 (0-0)	-0.01 (-0.35-0.33)	0.04 (-0.17-0.26)
	Central Latin America	0.8 (0.7-0.92)	0.85 (0.74-0.97)	0.06 (0.06-0.05)	0 (-0.05-0.05)	1.37 (1.22-1.52)
	Central Sub-Saharan Africa	0.05 (0.04-0.06)	0.05 (0.04-0.07)	0 (0-0.17)	0.01 (-0.06-0.07)	0.14 (0.13-0.15)
	East Asia	3.17 (2.58-3.79)	3.44 (2.8-4.08)	0.09 (0.09-0.08)	-0.1 (-0.51-0.32)	2.34 (1.89-2.8)
	Eastern Europe	0.11 (0.09-0.14)	0.11 (0.09-0.14)	0 (0-0)	0.12 (0.01-0.23)	-3.2 (-3.47--2.92)
	Eastern Sub-Saharan Africa	0.13 (0.11-0.16)	0.14 (0.12-0.17)	0.08 (0.09-0.06)	-0.1 (-0.29-0.09)	0.02 (0.01-0.04)
	High-income Asia Pacific	3.62 (3.1-4.2)	3.7 (3.18-4.27)	0.02 (0.03-0.02)	-0.06 (-0.26-0.15)	1.31 (1.08-1.54)
	High-income North America	5.16 (4.46-5.92)	5.45 (4.64-6.36)	0.06 (0.04-0.07)	1.06 (-0.17-2.31)	1.2 (0.97-1.42)
	North Africa and Middle East	0.64 (0.54-0.75)	0.69 (0.58-0.82)	0.08 (0.07-0.09)	0.18 (0.09-0.27)	1.07 (1.02-1.12)
	Oceania	0.01 (0.01-0.02)	0.02 (0.01-0.02)	1 (0-0)	0.54 (0.52-0.55)	0.47 (0.46-0.48)
	South Asia	6.16 (5.13-7.32)	6.51 (5.41-7.73)	0.06 (0.05-0.06)	-0.04 (-0.13-0.06)	0.41 (0.39-0.43)
	Southeast Asia	0.63 (0.52-0.75)	0.68 (0.56-0.8)	0.08 (0.08-0.07)	0.31 (0.21-0.4)	0.58 (0.55-0.61)
	Southern Latin America	0.64 (0.59-0.7)	0.67 (0.62-0.74)	0.05 (0.05-0.06)	0.42 (0.3-0.54)	2.08 (1.86-2.29)
	Southern Sub-Saharan Africa	0.15 (0.12-0.17)	0.15 (0.12-0.18)	0 (0-0.06)	-0.22 (-0.38--0.06)	-0.54 (-0.71--0.37)
	Tropical Latin America	0.47 (0.4-0.54)	0.5 (0.43-0.58)	0.06 (0.07-0.07)	0.14 (0.04-0.25)	1.11 (0.92-1.31)

	Western Europe	3.52 (3.14-3.93)	3.63 (3.24-4.05)	0.03 (0.03-0.03)	0.07 (0.03-0.12)	2.19 (2.02-2.36)
	Western Sub-Saharan Africa	0.1 (0.09-0.12)	0.11 (0.09-0.13)	0.1 (0-0.08)	-0.43 (-0.53--0.33)	-1.06 (-1.13--0.98)
Measure						
Deaths	Global	17.13 (14.93-19.34)	17.44 (14.99-19.63)	0.02 (0-0.01)	-1.87 (-2.52--1.22)	1.89 (1.72-2.05)
	Low SDI	0.93 (0.56-1.32)	0.94 (0.56-1.35)	0.01 (0-0.02)	-2.19 (-2.59--1.79)	0.74 (0.62-0.86)
	Low-middle SDI	3.42 (2.27-4.78)	3.46 (2.35-4.72)	0.01 (0.04--0.01)	-2.03 (-2.12--1.94)	1.11 (1.01-1.21)
	Middle SDI	2.88 (2.4-3.48)	2.97 (2.47-3.57)	0.03 (0.03-0.03)	-2.27 (-2.63--1.91)	1.59 (1.47-1.71)
	High-middle SDI	2.15 (1.88-2.36)	2.15 (1.86-2.36)	0 (-0.01-0)	-2.72 (-3.27--2.17)	1.77 (1.6-1.95)
	High SDI	7.74 (6.73-8.35)	7.91 (6.86-8.54)	0.02 (0.02-0.02)	-0.56 (-2.06-0.95)	2.88 (2.62-3.14)
	Andean Latin America	0.65 (0.51-0.78)	0.6 (0.45-0.76)	-0.08 (-0.12--0.03)	-6.65 (-7.46--5.83)	2.38 (2.17-2.6)
	Australasia	0.18 (0.15-0.2)	0.18 (0.15-0.2)	0 (0-0)	-1.17 (-6.16-4.08)	3.9 (3.31-4.5)
	Caribbean	0.05 (0.05-0.06)	0.05 (0.05-0.07)	0 (0-0.17)	-0.64 (-0.98--0.3)	2.18 (1.95-2.41)
	Central Asia	0.05 (0.04-0.06)	0.05 (0.04-0.06)	0 (0-0)	-0.89 (-0.94--0.83)	-2.14 (-2.54--1.74)
	Central Europe	0.19 (0.18-0.21)	0.2 (0.18-0.22)	0.05 (0-0.05)	0.9 (0.54-1.26)	0.72 (0.36-1.08)
	Central Latin America	0.53 (0.49-0.55)	0.56 (0.5-0.61)	0.06 (0.02-0.11)	-0.61 (-1.54-0.33)	2.48 (2.23-2.74)
	Central Sub-Saharan Africa	0.05 (0.02-0.13)	0.05 (0.02-0.13)	0 (0-0)	-0.33 (-0.56--0.09)	0.1 (0.05-0.15)
	East Asia	0.71 (0.46-0.94)	0.75 (0.47-1)	0.06 (0.02-0.06)	-0.75 (-1.06--0.45)	0.89 (0.75-1.03)
	Eastern Europe	0.1 (0.09-0.1)	0.1 (0.09-0.11)	0 (0-0.1)	0.02 (-1.22-1.27)	-5.39 (-6.3--4.47)
	Eastern Sub-Saharan Africa	0.1 (0.04-0.22)	0.11 (0.04-0.24)	0.1 (0-0.09)	-0.33 (-0.6--0.06)	-0.23 (-0.27--0.19)
	High-income Asia Pacific	2.5 (2.07-2.75)	2.59 (2.15-2.85)	0.04 (0.04-0.04)	0.61 (-2.41-3.74)	3.05 (2.86-3.24)
	High-income North America	2.76 (2.43-2.94)	2.86 (2.49-3.04)	0.04 (0.02-0.03)	-0.01 (-1.02-1.01)	2.18 (1.77-2.59)
	North Africa and Middle East	0.23 (0.17-0.34)	0.24 (0.18-0.37)	0.04 (0.06-0.09)	-0.94 (-1.23--0.66)	1 (0.88-1.13)
	Oceania	0.01 (0.01-0.02)	0.01 (0.01-0.02)	0 (0-0)	-0.63 (-0.7--0.56)	0.17 (0.14-0.21)
	South Asia	4.78 (3.13-6.69)	4.89 (3.21-6.64)	0.02 (0.03--0.01)	-1.69 (-1.78--1.61)	0.93 (0.84-1.02)
	Southeast Asia	0.14 (0.08-0.29)	0.15 (0.08-0.31)	0.07 (0-0.07)	-0.08 (-3.85-3.83)	0.37 (0.22-0.53)
	Southern Latin America	0.43 (0.39-0.46)	0.41 (0.37-0.45)	-0.05 (-0.05--0.02)	-4.11 (-5.11--3.1)	2.79 (2.33-3.25)
	Southern Sub-Saharan Africa	0.08 (0.06-0.11)	0.08 (0.06-0.11)	0 (0-0)	0.04 (-1.03-1.12)	-0.45 (-0.76--0.15)
	Tropical Latin America	0.38 (0.34-0.41)	0.4 (0.35-0.43)	0.05 (0.03-0.05)	-1.31 (-2.58--0.02)	2.77 (2.39-3.15)
	Western Europe	3.02 (2.67-3.23)	2.97 (2.6-3.19)	-0.02 (-0.03--0.01)	-2.31 (-2.87--1.74)	4.49 (4.14-4.84)
	Western Sub-Saharan Africa	0.18 (0.07-0.31)	0.19 (0.07-0.33)	0.06 (0-0.06)	-1.23 (-1.34--1.11)	-0.78 (-0.87--0.7)
Measure						
DALY	Global	321.02 (278.09-361.64)	328.43 (284.8-366.47)	0.02 (0.02-0.01)	-1.61 (-2.26--0.96)	1.26 (1.13-1.39)
	Low SDI	19.68 (12.2-27.55)	20.02 (12.3-28.4)	0.02 (0.01-0.03)	-1.88 (-2.02--1.73)	0.44 (0.36-0.52)
	Low-middle SDI	70.9 (48.16-97.32)	71.89 (49.39-97.51)	0.01 (0.03-0)	-1.95 (-2.19--1.72)	0.77 (0.7-0.84)

Middle SDI	58.29 (49.95-70.21)	61.04 (52-73.64)	0.05 (0.04-0.05)	-1.43 (-1.67--1.18)	1.15 (1.07-1.23)
High-middle SDI	39.91 (35.54-43.96)	40.32 (35.52-44.78)	0.01 (0-0.02)	-2.12 (-2.32--1.93)	0.97 (0.85-1.09)
High SDI	132.07 (118.72-141.48)	135 (121.14-145.22)	0.02 (0.02-0.03)	-0.56 (-1.91-0.81)	2.06 (1.84-2.28)
Andean Latin America	10.55 (8.46-12.47)	10.03 (7.87-12.45)	-0.05 (-0.07-0)	-4.7 (-5.4--3.99)	1.91 (1.73-2.09)
Australasia	2.92 (2.57-3.19)	3.02 (2.61-3.29)	0.03 (0.02-0.03)	-1.07 (-5.13-3.17)	3.23 (2.68-3.77)
Caribbean	0.99 (0.86-1.17)	1.03 (0.87-1.23)	0.04 (0.01-0.05)	-0.54 (-0.65--0.43)	1.92 (1.71-2.13)
Central Asia	1.11 (0.95-1.31)	1.18 (0.97-1.43)	0.06 (0.02-0.09)	-0.19 (-0.6-0.23)	-1.92 (-2.29--1.56)
Central Europe	4.09 (3.82-4.38)	4.15 (3.78-4.54)	0.01 (-0.01-0.04)	0.68 (0.33-1.04)	0.3 (-0.01-0.61)
Central Latin America	10.39 (9.81-10.91)	11.05 (10.02-12.16)	0.06 (0.02-0.11)	0.2 (0.09-0.31)	2.1 (1.88-2.31)
Central Sub-Saharan Africa	1.1 (0.43-2.71)	1.18 (0.46-2.83)	0.07 (0.07-0.04)	0.18 (-0.01-0.38)	0.03 (-0.02-0.07)
East Asia	17.65 (13.38-23.1)	18.91 (13.46-24.2)	0.07 (0.01-0.05)	-0.71 (-1.03--0.38)	0.61 (0.45-0.77)
Eastern Europe	2.35 (2.2-2.52)	2.35 (2.15-2.59)	0 (-0.02-0.03)	0.03 (-1.08-1.15)	-4.98 (-5.78--4.17)
Eastern Sub-Saharan Africa	2.33 (0.92-4.62)	2.47 (0.97-5.01)	0.06 (0.05-0.08)	0.09 (0.02-0.16)	-0.28 (-0.32--0.24)
High-income Asia Pacific	40.2 (35.23-44.11)	41.2 (36.15-45.36)	0.02 (0.03-0.03)	0.04 (-2.33-2.48)	1.9 (1.74-2.06)
High-income North America	49.52 (44.58-52.83)	51.51 (46.65-54.95)	0.04 (0.05-0.04)	0.3 (-0.58-1.19)	1.58 (1.25-1.92)
North Africa and Middle East	5.4 (4.26-7.63)	5.78 (4.44-8.3)	0.07 (0.04-0.09)	-0.24 (-0.25--0.23)	0.91 (0.82-1.01)
Oceania	0.21 (0.13-0.37)	0.23 (0.14-0.4)	0.1 (0.08-0.08)	-0.28 (-0.3--0.25)	0.17 (0.08-0.26)
South Asia	99.7 (66.93-138.16)	101.79 (67.97-137.19)	0.02 (0.02--0.01)	-1.7 (-1.96--1.44)	0.57 (0.52-0.62)
Southeast Asia	3.57 (2.13-6.45)	3.84 (2.25-6.97)	0.08 (0.06-0.08)	0.02 (-2.67-2.78)	0.3 (0.2-0.4)
Southern Latin America	7.66 (7.08-8.13)	7.42 (6.79-7.95)	-0.03 (-0.04--0.02)	-3.42 (-3.9--2.94)	2.25 (1.89-2.6)
Southern Sub-Saharan Africa	1.7 (1.21-2.19)	1.79 (1.26-2.32)	0.05 (0.04-0.06)	0.89 (0.03-1.76)	-0.28 (-0.58-0.03)
Tropical Latin America	7.11 (6.54-7.53)	7.46 (6.81-7.94)	0.05 (0.04-0.05)	-0.7 (-1.79-0.4)	2.06 (1.75-2.37)
Western Europe	48.61 (44.08-51.61)	47.99 (43.42-51.1)	-0.01 (-0.01--0.01)	-2.12 (-2.5--1.74)	3.53 (3.22-3.85)
Western Sub-Saharan Africa	3.86 (1.6-6.49)	4.05 (1.65-6.94)	0.05 (0.03-0.07)	-0.71 (-0.74--0.68)	-0.78 (-0.87--0.69)

Table S4 Temporal Joinpoint Analysis of CRDs, COPD, Asthma, and ILD & PS in Adults Aged 55 and Above at the Global Level, 1990-2021

Temporal Joinpoint Analysis of CRDs in Adults Aged 55 and Above, 1990-2021							
Prevalence							
Sex	Start	End	Measure	Val	lower	upper	P.Val
Both	1990	2021	AAPC	-0.728	-0.757	-0.699	0.000
Female	1990	2021	AAPC	-0.743	-0.784	-0.702	0.000
Male	1990	2021	AAPC	-0.708	-0.755	-0.661	0.000
Both	1990	1993	APC	-1.602	-1.740	-1.464	0.000
Both	1993	1997	APC	-1.131	-1.265	-0.997	0.000
Both	1997	2006	APC	-0.756	-0.784	-0.728	0.000
Both	2006	2013	APC	-0.627	-0.670	-0.584	0.000
Both	2013	2018	APC	-0.358	-0.443	-0.273	0.000
Both	2018	2021	APC	-0.079	-0.215	0.057	0.236
Female	1990	1993	APC	-1.838	-2.015	-1.661	0.000
Female	1993	1997	APC	-1.294	-1.467	-1.122	0.000
Female	1997	2008	APC	-0.783	-0.809	-0.757	0.000
Female	2008	2011	APC	-0.625	-0.946	-0.303	0.001
Female	2011	2021	APC	-0.180	-0.206	-0.154	0.000
Male	1990	1995	APC	-1.191	-1.281	-1.101	0.000
Male	1995	2006	APC	-0.738	-0.768	-0.708	0.000
Male	2006	2010	APC	-0.467	-0.659	-0.275	0.000
Male	2010	2014	APC	-0.990	-1.186	-0.795	0.000
Male	2014	2018	APC	-0.454	-0.655	-0.253	0.000
Male	2018	2021	APC	-0.069	-0.271	0.135	0.483
Incidence							
Sex	Start	End	Measure	Val	lower	upper	P.Val
Both	1990	2021	AAPC	-0.556	-0.579	-0.534	0.000
Female	1990	2021	AAPC	-0.474	-0.508	-0.439	0.000
Male	1990	2021	AAPC	-0.657	-0.675	-0.639	0.000
Both	1990	1994	APC	-1.074	-1.153	-0.994	0.000
Both	1994	2001	APC	-0.629	-0.668	-0.590	0.000
Both	2001	2007	APC	-0.814	-0.862	-0.766	0.000
Both	2007	2012	APC	-0.570	-0.635	-0.505	0.000
Both	2012	2019	APC	-0.183	-0.217	-0.150	0.000
Both	2019	2021	APC	0.245	0.050	0.441	0.017
Female	1990	1995	APC	-1.080	-1.214	-0.946	0.000
Female	1995	2002	APC	-0.543	-0.635	-0.450	0.000
Female	2002	2010	APC	-0.704	-0.772	-0.637	0.000
Female	2010	2021	APC	0.016	-0.017	0.048	0.335
Male	1990	1993	APC	-1.071	-1.162	-0.980	0.000
Male	1993	2001	APC	-0.688	-0.710	-0.665	0.000
Male	2001	2005	APC	-1.039	-1.117	-0.960	0.000
Male	2005	2014	APC	-0.699	-0.715	-0.683	0.000
Male	2014	2019	APC	-0.308	-0.352	-0.263	0.000
Male	2019	2021	APC	0.170	0.028	0.312	0.022
Deaths							
Sex	Start	End	Measure	Val	lower	upper	P.Val
Both	1990	2021	AAPC	-1.148	-1.244	-1.053	0.000
Female	1990	2021	AAPC	-1.038	-1.193	-0.882	0.000
Male	1990	2021	AAPC	-1.250	-1.373	-1.127	0.000
Both	1990	1995	APC	0.170	-0.058	0.400	0.135
Both	1995	2003	APC	-0.995	-1.113	-0.877	0.000
Both	2003	2006	APC	-3.156	-3.941	-2.364	0.000
Both	2006	2012	APC	-1.745	-1.932	-1.557	0.000
Both	2012	2021	APC	-0.938	-1.036	-0.840	0.000
Female	1990	1995	APC	0.493	0.092	0.896	0.019
Female	1995	2004	APC	-1.242	-1.398	-1.086	0.000
Female	2004	2007	APC	-3.178	-4.439	-1.901	0.000
Female	2007	2012	APC	-1.593	-1.996	-1.188	0.000
Female	2012	2021	APC	-0.646	-0.785	-0.507	0.000
Male	1990	1995	APC	-0.046	-0.371	0.281	0.771
Male	1995	2003	APC	-0.905	-1.060	-0.749	0.000
Male	2003	2006	APC	-3.359	-4.364	-2.344	0.000
Male	2006	2012	APC	-1.717	-1.939	-1.494	0.000
Male	2012	2021	APC	-1.199	-1.326	-1.071	0.000
DALYs							
Sex	Start	End	Measure	Val	lower	upper	P.Val

Both	1990	2021	AAPC	-1.338	-1.427	-1.248	0.000
Female	1990	2021	AAPC	-1.214	-1.341	-1.088	0.000
Male	1990	2021	AAPC	-1.470	-1.590	-1.350	0.000
Both	1990	1995	APC	-0.313	-0.538	-0.087	0.009
Both	1995	2003	APC	-1.352	-1.468	-1.237	0.000
Both	2003	2006	APC	-3.209	-3.953	-2.459	0.000
Both	2006	2014	APC	-1.743	-1.848	-1.637	0.000
Both	2014	2021	APC	-0.775	-0.904	-0.646	0.000
Female	1990	1995	APC	-0.174	-0.504	0.157	0.279
Female	1995	2003	APC	-1.354	-1.519	-1.189	0.000
Female	2003	2007	APC	-2.781	-3.303	-2.256	0.000
Female	2007	2014	APC	-1.545	-1.715	-1.376	0.000
Female	2014	2018	APC	-0.144	-0.648	0.362	0.553
Female	2018	2021	APC	-1.107	-1.759	-0.452	0.003
Male	1990	1995	APC	-0.436	-0.747	-0.124	0.009
Male	1995	2003	APC	-1.384	-1.533	-1.234	0.000
Male	2003	2006	APC	-3.388	-4.377	-2.389	0.000
Male	2006	2015	APC	-1.821	-1.927	-1.715	0.000
Male	2015	2021	APC	-0.945	-1.184	-0.706	0.000

CRDs = Chronic Respiratory Diseases; DALYs = Disability-Adjusted Life Years.

Temporal Joinpoint Analysis of COPD in Adults Aged 55 and Above, 1990-2021

Sex	Start	End	Measure	Val	lower	upper	P.Val
Prevalence							
Both	1990	2021	AAPC	0.180	0.158	0.202	0.000
Female	1990	2021	AAPC	0.154	0.129	0.180	0.000
Male	1990	2021	AAPC	0.207	0.187	0.228	0.000
Both	1990	1996	APC	0.395	0.360	0.431	0.000
Both	1996	2001	APC	0.755	0.690	0.820	0.000
Both	2001	2005	APC	0.212	0.115	0.310	0.000
Both	2005	2011	APC	-0.178	-0.220	-0.135	0.000
Both	2011	2015	APC	0.153	0.058	0.249	0.004
Both	2015	2021	APC	-0.156	-0.189	-0.123	0.000
Female	1990	1995	APC	-0.013	-0.062	0.037	0.593
Female	1995	2002	APC	0.628	0.591	0.664	0.000
Female	2002	2005	APC	0.065	-0.140	0.269	0.512
Female	2005	2010	APC	-0.394	-0.457	-0.331	0.000
Female	2010	2015	APC	0.565	0.501	0.628	0.000
Female	2015	2021	APC	-0.097	-0.131	-0.062	0.000
Male	1990	2002	APC	0.761	0.742	0.780	0.000
Male	2002	2009	APC	0.113	0.060	0.166	0.000
Male	2009	2016	APC	-0.361	-0.414	-0.309	0.000
Male	2016	2021	APC	-0.188	-0.259	-0.117	0.000
Incidence							
Sex	Start	End	Measure	Val	lower	upper	P.Val
Both	1990	2021	AAPC	0.114	0.088	0.140	0.000
Female	1990	2021	AAPC	0.158	0.124	0.192	0.000
Male	1990	2021	AAPC	0.063	0.039	0.088	0.000
Both	1990	1996	APC	0.374	0.331	0.417	0.000
Both	1996	2001	APC	0.505	0.425	0.585	0.000
Both	2001	2005	APC	-0.090	-0.211	0.032	0.137
Both	2005	2010	APC	-0.393	-0.467	-0.318	0.000
Both	2010	2015	APC	0.236	0.163	0.308	0.000
Both	2015	2021	APC	-0.012	-0.052	0.027	0.513
Female	1990	1996	APC	0.179	0.124	0.233	0.000
Female	1996	2001	APC	0.565	0.462	0.669	0.000
Female	2001	2005	APC	-0.030	-0.186	0.126	0.688
Female	2005	2010	APC	-0.436	-0.533	-0.339	0.000
Female	2010	2015	APC	0.616	0.522	0.711	0.000
Female	2015	2021	APC	0.039	-0.011	0.089	0.117
Male	1990	1995	APC	0.582	0.519	0.644	0.000
Male	1995	2001	APC	0.452	0.391	0.512	0.000
Male	2001	2005	APC	-0.166	-0.298	-0.034	0.017
Male	2005	2011	APC	-0.344	-0.400	-0.288	0.000

Sex	Start	End	Measure	Val	lower	upper	P.Val
Male	2011	2021	APC	-0.091	-0.110	-0.071	0.000
Deaths							
Sex	Start	End	Measure	Val	lower	upper	P.Val
Both	1990	2021	AAPC	-1.195	-1.300	-1.089	0.000
Female	1990	2021	AAPC	-1.158	-1.330	-0.986	0.000
Male	1990	2021	AAPC	-1.237	-1.401	-1.073	0.000
Both	1990	1995	APC	0.170	-0.086	0.426	0.180
Both	1995	2003	APC	-0.991	-1.117	-0.864	0.000
Both	2003	2006	APC	-3.389	-4.264	-2.507	0.000
Both	2006	2012	APC	-1.859	-2.068	-1.650	0.000
Both	2012	2021	APC	-0.944	-1.048	-0.840	0.000
Female	1990	1995	APC	0.314	-0.033	0.663	0.073
Female	1995	2001	APC	-1.168	-1.445	-0.891	0.000
Female	2001	2004	APC	-1.771	-2.906	-0.624	0.005
Female	2004	2007	APC	-3.316	-4.407	-2.214	0.000
Female	2007	2012	APC	-1.816	-2.181	-1.449	0.000
Female	2012	2021	APC	-0.663	-0.789	-0.537	0.000
Male	1990	1995	APC	0.010	-0.397	0.418	0.961
Male	1995	2003	APC	-0.828	-1.026	-0.630	0.000
Male	2003	2006	APC	-3.531	-4.888	-2.155	0.000
Male	2006	2013	APC	-1.720	-1.954	-1.485	0.000
Male	2013	2021	APC	-1.127	-1.338	-0.916	0.000
DALYs							
Sex	Start	End	Measure	Val	lower	upper	P.Val
Both	1990	2021	AAPC	-1.323	-1.417	-1.229	0.000
Female	1990	2021	AAPC	-1.217	-1.389	-1.045	0.000
Male	1990	2021	AAPC	-1.422	-1.544	-1.300	0.000
Both	1990	1995	APC	-0.183	-0.424	0.060	0.131
Both	1995	2003	APC	-1.269	-1.383	-1.155	0.000
Both	2003	2006	APC	-3.424	-4.203	-2.639	0.000
Both	2006	2014	APC	-1.804	-1.915	-1.693	0.000
Both	2014	2021	APC	-0.732	-0.866	-0.598	0.000
Female	1990	1995	APC	-0.139	-0.510	0.233	0.439
Female	1995	2001	APC	-1.235	-1.527	-0.943	0.000
Female	2001	2004	APC	-1.965	-3.113	-0.803	0.003
Female	2004	2007	APC	-3.150	-4.253	-2.036	0.000
Female	2007	2013	APC	-1.698	-1.943	-1.453	0.000
Female	2013	2021	APC	-0.496	-0.637	-0.355	0.000
Male	1990	1995	APC	-0.284	-0.586	0.019	0.064
Male	1995	2003	APC	-1.244	-1.391	-1.097	0.000
Male	2003	2006	APC	-3.534	-4.550	-2.507	0.000
Male	2006	2015	APC	-1.841	-1.959	-1.722	0.000
Male	2015	2021	APC	-0.905	-1.138	-0.672	0.000

COPD = Chronic Obstructive Pulmonary Disease; DALYs = Disability-Adjusted Life Years.

Temporal Joinpoint Analysis of Asthma in Adults Aged 55 and Above, 1990-2021

Sex	Start	End	Measure	Val	lower	upper	P.Val
Prevalence							
Sex	Start	End	Measure	Val	lower	upper	P.Val
Both	1990	2021	AAPC	-2.489	-2.568	-2.410	0.000
Female	1990	2021	AAPC	-2.365	-2.434	-2.297	0.000
Male	1990	2021	AAPC	-2.666	-2.730	-2.601	0.000
Both	1990	1999	APC	-3.934	-4.005	-3.864	0.000
Both	1999	2005	APC	-3.178	-3.348	-3.008	0.000
Both	2005	2010	APC	-1.709	-1.939	-1.479	0.000
Both	2010	2014	APC	-2.515	-2.851	-2.177	0.000
Both	2014	2018	APC	-0.979	-1.300	-0.658	0.000
Both	2018	2021	APC	0.038	-0.288	0.365	0.808
Female	1990	1999	APC	-3.886	-3.947	-3.824	0.000
Female	1999	2005	APC	-2.934	-3.082	-2.786	0.000
Female	2005	2010	APC	-1.504	-1.701	-1.306	0.000
Female	2010	2014	APC	-2.296	-2.588	-2.004	0.000
Female	2014	2018	APC	-0.888	-1.166	-0.610	0.000
Female	2018	2021	APC	-0.078	-0.362	0.208	0.570
Male	1990	1994	APC	-4.389	-4.589	-4.189	0.000

Male	1994	2005	APC	-3.657	-3.705	-3.608	0.000
Male	2005	2010	APC	-1.886	-2.076	-1.696	0.000
Male	2010	2014	APC	-2.826	-3.105	-2.546	0.000
Male	2014	2018	APC	-1.091	-1.357	-0.825	0.000
Male	2018	2021	APC	0.186	-0.080	0.452	0.157
Incidence							
Sex	Start	End	Measure	Val	lower	upper	P.Val
Both	1990	2021	AAPC	-1.876	-1.936	-1.817	0.000
Female	1990	2021	AAPC	-1.643	-1.686	-1.600	0.000
Male	1990	2021	AAPC	-2.140	-2.242	-2.038	0.000
Both	1990	1993	APC	-3.335	-3.662	-3.007	0.000
Both	1993	2004	APC	-2.683	-2.732	-2.633	0.000
Both	2004	2015	APC	-1.628	-1.675	-1.582	0.000
Both	2015	2019	APC	-0.453	-0.730	-0.175	0.003
Both	2019	2021	APC	0.626	0.073	1.181	0.029
Female	1990	1993	APC	-3.134	-3.348	-2.919	0.000
Female	1993	1999	APC	-2.563	-2.657	-2.469	0.000
Female	1999	2004	APC	-2.231	-2.359	-2.103	0.000
Female	2004	2014	APC	-1.443	-1.478	-1.407	0.000
Female	2014	2019	APC	-0.401	-0.515	-0.287	0.000
Female	2019	2021	APC	0.806	0.448	1.165	0.000
Male	1990	1992	APC	-3.697	-4.492	-2.896	0.000
Male	1992	2005	APC	-2.954	-3.001	-2.908	0.000
Male	2005	2011	APC	-1.720	-1.892	-1.547	0.000
Male	2011	2014	APC	-2.319	-3.046	-1.586	0.000
Male	2014	2019	APC	-0.868	-1.087	-0.649	0.000
Male	2019	2021	APC	0.635	-0.059	1.334	0.070
Deaths							
Sex	Start	End	Measure	Val	lower	upper	P.Val
Both	1990	2021	AAPC	-1.703	-1.960	-1.446	0.000
Female	1990	2021	AAPC	-1.162	-1.439	-0.884	0.000
Male	1990	2021	AAPC	-2.233	-2.342	-2.124	0.000
Both	1990	1996	APC	-0.389	-0.732	-0.044	0.029
Both	1996	2001	APC	-2.195	-2.834	-1.551	0.000
Both	2001	2004	APC	-1.408	-3.462	0.690	0.172
Both	2004	2010	APC	-2.507	-2.965	-2.046	0.000
Both	2010	2019	APC	-1.457	-1.664	-1.250	0.000
Both	2019	2021	APC	-3.497	-5.094	-1.873	0.000
Female	1990	1996	APC	0.299	-0.083	0.683	0.116
Female	1996	2001	APC	-1.759	-2.449	-1.064	0.000
Female	2001	2004	APC	-0.429	-2.680	1.875	0.694
Female	2004	2010	APC	-2.074	-2.606	-1.539	0.000
Female	2010	2018	APC	-0.696	-0.989	-0.402	0.000
Female	2018	2021	APC	-3.183	-4.127	-2.230	0.000
Male	1990	1997	APC	-1.189	-1.539	-0.839	0.000
Male	1997	2011	APC	-2.747	-2.877	-2.616	0.000
Male	2011	2021	APC	-2.240	-2.424	-2.056	0.000
DALYs							
Sex	Start	End	Measure	Val	lower	upper	P.Val
Both	1990	2021	AAPC	-2.072	-2.195	-1.950	0.000
Female	1990	2021	AAPC	-1.687	-1.866	-1.507	0.000
Male	1990	2021	AAPC	-2.487	-2.583	-2.391	0.000
Both	1990	1996	APC	-1.567	-1.802	-1.332	0.000
Both	1996	2006	APC	-2.606	-2.738	-2.474	0.000
Both	2006	2014	APC	-2.227	-2.424	-2.030	0.000
Both	2014	2019	APC	-1.160	-1.584	-0.734	0.000
Both	2019	2021	APC	-2.560	-3.754	-1.352	0.000
Female	1990	1992	APC	-1.975	-3.111	-0.826	0.002
Female	1992	1995	APC	-0.750	-1.930	0.444	0.200
Female	1995	2010	APC	-2.042	-2.100	-1.984	0.000
Female	2010	2015	APC	-1.464	-1.864	-1.062	0.000
Female	2015	2018	APC	-0.393	-1.532	0.759	0.477
Female	2018	2021	APC	-2.299	-2.846	-1.750	0.000
Male	1990	1997	APC	-2.037	-2.249	-1.825	0.000
Male	1997	2006	APC	-3.209	-3.387	-3.031	0.000
Male	2006	2014	APC	-2.650	-2.876	-2.423	0.000

Male	2014	2021	APC	-1.815	-2.013	-1.617	0.000

DALYs = Disability-Adjusted Life Years.

Temporal Joinpoint Analysis of ILD & PS in Adults Aged 55 and Above, 1990-2021

Sex	Start	End	Measure	Val	lower	upper	P.Val
**Prevalence**							
Both	1990	2021	AAPC	0.571	0.528	0.615	0.000
Female	1990	2021	AAPC	0.504	0.464	0.544	0.000
Male	1990	2021	AAPC	0.639	0.591	0.687	0.000
Both	1990	1995	APC	0.600	0.525	0.675	0.000
Both	1995	2000	APC	1.585	1.480	1.690	0.000
Both	2000	2006	APC	0.694	0.623	0.764	0.000
Both	2006	2009	APC	1.614	1.314	1.915	0.000
Both	2009	2012	APC	0.029	-0.257	0.316	0.832
Both	2012	2021	APC	-0.247	-0.272	-0.222	0.000
Female	1990	1995	APC	0.457	0.376	0.538	0.000
Female	1995	2000	APC	1.449	1.333	1.564	0.000
Female	2000	2006	APC	0.691	0.613	0.769	0.000
Female	2006	2009	APC	1.595	1.260	1.932	0.000
Female	2009	2021	APC	-0.231	-0.249	-0.212	0.000
Male	1990	1995	APC	0.739	0.657	0.822	0.000
Male	1995	2000	APC	1.724	1.608	1.840	0.000
Male	2000	2006	APC	0.679	0.601	0.757	0.000
Male	2006	2009	APC	1.720	1.392	2.050	0.000
Male	2009	2012	APC	0.140	-0.171	0.453	0.353
Male	2012	2021	APC	-0.231	-0.259	-0.204	0.000
**Incidence**							
Sex	Start	End	Measure	Val	lower	upper	P.Val
Both	1990	2021	AAPC	1.084	1.048	1.121	0.000
Female	1990	2021	AAPC	1.171	1.133	1.210	0.000
Male	1990	2021	AAPC	1.006	0.972	1.040	0.000
Both	1990	1995	APC	1.420	1.359	1.482	0.000
Both	1995	2000	APC	2.312	2.228	2.395	0.000
Both	2000	2006	APC	1.174	1.119	1.229	0.000
Both	2006	2009	APC	2.315	2.079	2.552	0.000
Both	2009	2012	APC	0.472	0.245	0.700	0.001
Both	2012	2019	APC	0.038	0.002	0.075	0.040
Both	2019	2021	APC	-0.300	-0.512	-0.088	0.010
Female	1990	1995	APC	1.469	1.405	1.533	0.000
Female	1995	2000	APC	2.375	2.288	2.462	0.000
Female	2000	2006	APC	1.275	1.217	1.333	0.000
Female	2006	2009	APC	2.373	2.125	2.622	0.000
Female	2009	2012	APC	0.507	0.269	0.744	0.001
Female	2012	2019	APC	0.200	0.161	0.238	0.000
Female	2019	2021	APC	-0.240	-0.468	-0.011	0.041
Male	1990	1995	APC	1.350	1.280	1.420	0.000
Male	1995	2000	APC	2.255	2.159	2.350	0.000
Male	2000	2006	APC	1.067	1.004	1.130	0.000
Male	2006	2009	APC	2.305	2.038	2.573	0.000
Male	2009	2014	APC	0.261	0.182	0.341	0.000
Male	2014	2021	APC	-0.190	-0.222	-0.158	0.000
**Deaths**							
Sex	Start	End	Measure	Val	lower	upper	P.Val
Both	1990	2021	AAPC	1.651	1.466	1.835	0.000
Female	1990	2021	AAPC	1.837	1.622	2.053	0.000
Male	1990	2021	AAPC	1.489	1.309	1.670	0.000
Both	1990	1993	APC	1.652	0.428	2.890	0.011
Both	1993	2003	APC	2.972	2.781	3.164	0.000
Both	2003	2014	APC	1.542	1.409	1.675	0.000
Both	2014	2019	APC	0.763	0.247	1.281	0.006
Both	2019	2021	APC	-2.021	-3.640	-0.375	0.019
Female	1990	1993	APC	2.131	0.730	3.551	0.005
Female	1993	2004	APC	3.277	3.083	3.472	0.000
Female	2004	2014	APC	1.569	1.380	1.758	0.000

Female	2014	2019	APC	0.801	0.176	1.430	0.015
Female	2019	2021	APC	-2.430	-4.288	-0.536	0.015
Male	1990	1993	APC	1.394	0.195	2.606	0.025
Male	1993	2003	APC	2.602	2.407	2.797	0.000
Male	2003	2014	APC	1.423	1.284	1.562	0.000
Male	2014	2019	APC	0.784	0.281	1.289	0.004
Male	2019	2021	APC	-1.712	-3.259	-0.141	0.034
DALYs							
Sex	Start	End	Measure	Val	lower	upper	P.Val
Both	1990	2021	AAPC	1.100	0.968	1.233	0.000
Female	1990	2021	AAPC	1.285	1.135	1.435	0.000
Male	1990	2021	AAPC	0.978	0.854	1.103	0.000
Both	1990	1993	APC	1.271	0.380	2.170	0.008
Both	1993	2003	APC	2.115	1.971	2.260	0.000
Both	2003	2012	APC	0.981	0.835	1.126	0.000
Both	2012	2019	APC	0.563	0.348	0.779	0.000
Both	2019	2021	APC	-1.744	-2.946	-0.528	0.008
Female	1990	1993	APC	1.369	0.410	2.337	0.008
Female	1993	2003	APC	2.506	2.342	2.671	0.000
Female	2003	2012	APC	1.099	0.932	1.266	0.000
Female	2012	2019	APC	0.659	0.410	0.908	0.000
Female	2019	2021	APC	-1.829	-3.230	-0.407	0.015
Male	1990	2003	APC	1.728	1.622	1.834	0.000
Male	2003	2012	APC	0.889	0.709	1.068	0.000
Male	2012	2019	APC	0.473	0.216	0.730	0.001
Male	2019	2021	APC	-1.668	-3.123	-0.191	0.029

ILD & PS = Interstitial lung disease and pulmonary sarcoidosis; DALYs = Disability-Adjusted Life Years.

Table S5 Overview of the Prevalence of CRDs, COPD, Asthma, and ILD & PS Among Adults Aged 55 and Above in 204 Countries Globally (EAPC, Time Points: 1990, 2019, 2021)

Cause	CRDs				
Location	Prevalence number	Prevalence rate	EAPC 1990-2021(95% CI)	EAPC 2019-2021(95% CI)	2021SDI
American Samoa	733.31 (647.24 to 827.48)	8682.16 (7663.06 to 9797.06)	-0.98 (-1.06 to -0.9)	-0.1 (-0.2 to -0.01)	0.724
Antigua and Barbuda	1535.7 (1369.83 to 1706.7)	8139.34 (7260.22 to 9045.64)	-0.24 (-0.28 to -0.19)	0.49 (0.32 to 0.65)	0.750
Egypt	1478048.9 (1315902.03 to 1658341.96)	13356.85 (11891.56 to 14986.13)	0.02 (-0.01 to 0.05)	1.34 (0.17 to 2.52)	0.607
Argentina	1494659.2 (1370905.45 to 1617886.27)	16014.25 (14688.31 to 17334.54)	-0.84 (-0.99 to -0.69)	0.33 (0.06 to 0.6)	0.723
Australia	1087962.47 (1004015.38 to 1193286.21)	14686.66 (13553.44 to 16108.45)	-1.4 (-1.51 to -1.29)	-0.92 (-2.05 to 0.22)	0.844
Barbados	10815.87 (9842.18 to 11916.4)	11870.07 (10801.47 to 13077.86)	-0.46 (-0.52 to -0.41)	0.26 (-0.07 to 0.59)	0.747
Belize	4853.27 (4374.8 to 5366.39)	9689.8 (8734.52 to 10714.26)	-0.05 (-0.12 to 0.03)	-0.4 (-0.43 to -0.36)	0.610
Bermuda	2235.87 (1967.04 to 2503.35)	9599.78 (8445.53 to 10748.23)	-0.21 (-0.27 to -0.15)	0.49 (-0.1 to 1.08)	0.821
Venezuela	590047.72 (535374.92 to 651821.38)	11277.26 (10232.33 to 12457.91)	0.21 (0.13 to 0.29)	0.14 (-0.77 to 1.07)	0.597
Bosnia and Herzegovina	192393.51 (174826.21 to 210417.79)	17565.88 (15961.95 to 19211.52)	0.05 (-0.1 to 0.2)	0.12 (-0.59 to 0.83)	0.723
Brunei	9470.66 (8758.07 to 10168.71)	15817.49 (14627.35 to 16983.34)	-1.38 (-1.5 to -1.26)	1.17 (1.15 to 1.19)	0.810
Burkina Faso	138071.3 (125127.88 to 153020.17)	9183.85 (8322.91 to 10178.18)	-0.14 (-0.19 to -0.09)	0.96 (0.62 to 1.29)	0.285
Canada	1994062.15 (1838500.25 to 2150708.5)	16249.96 (14982.25 to 17526.49)	-0.88 (-0.97 to -0.78)	0.4 (-0.12 to 0.93)	0.873
Central African Republic	43498.97 (39285.48 to 48187.76)	12160.79 (10982.85 to 13471.61)	-0.33 (-0.36 to -0.3)	0.09 (-0.43 to 0.62)	0.309
Dominica	1541.59 (1391.1 to 1694.31)	10220.82 (9223.12 to 11233.42)	-0.32 (-0.35 to -0.28)	0.17 (0.05 to 0.29)	0.747
Bahamas	5383.83 (4892.61 to 5908.79)	7481.84 (6799.2 to 8211.37)	-0.34 (-0.4 to -0.29)	-0.66 (-1.33 to 0.01)	0.805
Cook Islands	424.55 (362.6 to 484.18)	9008.22 (7693.73 to 10273.49)	-0.64 (-0.79 to -0.49)	0.61 (0.06 to 1.16)	0.779
Czech Republic	519987.89 (468894.69 to 566534.02)	14884.59 (13422.06 to 16216.97)	0.46 (0.38 to 0.54)	0.33 (0.15 to 0.51)	0.828
North Korea	995301.74 (911936.63 to 1073327.55)	17660.44 (16181.23 to 19044.92)	-0.23 (-0.32 to -0.15)	-0.48 (-0.56 to -0.41)	0.570
Sao Tome and Principe	2577.09 (2371.22 to 2799.46)	14094.87 (12968.92 to 15311.08)	-0.05 (-0.14 to 0.04)	0.63 (0.62 to 0.65)	0.505
DR Congo	649671.5 (584151.94 to 722409.12)	10756.48 (9671.69 to 11960.78)	0.08 (-0.01 to 0.16)	0.61 (0.47 to 0.76)	0.383
Timor-Leste	19081.46 (17373.7 to 21070.26)	13434.71 (12232.32 to 14834.97)	-0.23 (-0.32 to -0.14)	1.09 (0.03 to 2.17)	0.445
Sri Lanka	762448.18 (693657.18 to 835337.47)	15830.56 (14402.27 to 17343.95)	-0.39 (-0.46 to -0.33)	1.72 (1.36 to 2.09)	0.702
Dominican Republic	155283.57 (136523.61 to 174788.83)	9292.72 (8170.06 to 10459.98)	0.54 (0.47 to 0.6)	0.15 (-0.07 to 0.37)	0.619
Uruguay	137777.89 (126552.14 to 149587.69)	15235.99 (13994.61 to 16541.96)	-0.79 (-0.83 to -0.75)	-0.23 (-1.17 to 0.72)	0.719
Ethiopia	602371.27 (535203.39 to 669998.6)	8799.42 (7818.24 to 9787.32)	-0.81 (-0.87 to -0.75)	0.8 (0.4 to 1.21)	0.359
Nepal	655392.93 (617667.51 to 691119.47)	16559.29 (15606.11 to 17461.97)	0.03 (-0.01 to 0.06)	-0.08 (-0.21 to 0.05)	0.433
Germany	5781643.86 (5330052.67 to 6292328.53)	18346.33 (16913.34 to 19966.83)	-0.88 (-1.14 to -0.62)	-0.33 (-0.81 to 0.16)	0.903
Nigeria	1969912.56 (1770894.18 to 2179474.2)	13131.43 (11804.77 to 14528.36)	-0.26 (-0.34 to -0.19)	1.3 (1.07 to 1.54)	0.503
Somalia	125148.28 (112986.45 to 137618.41)	13270.44 (11980.82 to 14592.74)	-0.13 (-0.22 to -0.04)	0.55 (0.49 to 0.61)	0.078
Micronesia	1339.91 (1210.19 to 1493.16)	10182.37 (9196.58 to 11346.94)	-1.59 (-1.65 to -1.52)	0.27 (-0.07 to 0.61)	0.588

Brazil	5838860.69 (5173617.64 to 6484284.57)	13482.25 (11946.17 to 14972.57)	-0.51 (-0.62 to -0.41)	0.89 (-0.35 to 2.15)	0.653
France	3899590.1 (3585374.22 to 4253828.83)	17637.13 (16215.99 to 19239.28)	-1.41 (-1.51 to -1.32)	-0.16 (-0.27 to -0.04)	0.838
Gabon	15426.54 (13696.56 to 17427.4)	8630.14 (7662.33 to 9749.49)	-0.58 (-0.62 to -0.54)	0.59 (0.32 to 0.86)	0.635
Georgia	116530.92 (101453.47 to 130649.94)	11099.57 (9663.44 to 12444.41)	0.09 (0.04 to 0.14)	0.02 (-0.23 to 0.27)	0.732
Luxembourg	35076.07 (32045.12 to 37817.28)	19738.7 (18033.07 to 21281.29)	-0.96 (-1 to -0.91)	-0.26 (-0.66 to 0.14)	0.884
Greenland	2708.37 (2528.6 to 2885.58)	19949.7 (18625.51 to 21255)	-1.84 (-1.97 to -1.71)	0.31 (0.22 to 0.4)	0.826
Grenada	2072.93 (1832.77 to 2302.2)	10319.67 (9124.09 to 11461.04)	-0.66 (-0.75 to -0.58)	0.11 (-1.33 to 1.58)	0.669
Guam	3062.7 (2584.24 to 3475.87)	8127.02 (6857.4 to 9223.37)	-0.62 (-0.78 to -0.47)	0.41 (0.23 to 0.59)	0.804
Jordan	134413.02 (119655.24 to 150450.1)	10691.1 (9517.28 to 11966.68)	-0.56 (-0.61 to -0.51)	-0.4 (-0.94 to 0.14)	0.725
Greece	694876.76 (630522.41 to 761445.66)	18905.9 (17154.97 to 20717.08)	-0.36 (-0.45 to -0.27)	0.56 (0.53 to 0.59)	0.792
Hungary	545429.03 (491596.62 to 592757.69)	17151.19 (15458.41 to 18639.45)	0.02 (-0.03 to 0.07)	0.12 (0.08 to 0.17)	0.791
Papua New Guinea	118088.78 (109014.32 to 127352.51)	14378.22 (13273.34 to 15506.16)	-0.8 (-0.88 to -0.73)	-0.35 (-0.52 to -0.19)	0.418
Samoa	2931.91 (2591.12 to 3288.36)	11907.91 (10523.79 to 13355.63)	-0.69 (-0.77 to -0.62)	-0.41 (-0.41 to -0.4)	0.593
Ireland	238253.01 (219511.26 to 258654.17)	18154.43 (16726.35 to 19708.96)	-1.49 (-1.61 to -1.37)	0.71 (0.71 to 0.72)	0.874
Afghanistan	226336.05 (208332.25 to 246308.54)	18420.01 (16954.79 to 20045.43)	-0.35 (-0.38 to -0.32)	-0.79 (-1.3 to -0.29)	0.337
Iran	1509042.22 (1345784.1 to 1674586.23)	11616.11 (10359.4 to 12890.42)	-0.67 (-0.75 to -0.59)	-0.2 (-0.36 to -0.04)	0.697
Mauritania	43517.19 (39503.52 to 48018.17)	12129.97 (11011.2 to 13384.57)	-0.63 (-0.74 to -0.52)	1.42 (1.28 to 1.55)	0.499
Pakistan	2448929.33 (2178255.84 to 2728080.23)	12352.11 (10986.87 to 13760.12)	-0.8 (-0.87 to -0.73)	-0.76 (-1.18 to -0.33)	0.504
Jamaica	54460.09 (49570.53 to 59553.67)	10297.02 (9372.52 to 11260.08)	-0.3 (-0.42 to -0.18)	-0.01 (-0.37 to 0.36)	0.683
Japan	6948738.78 (6158717.58 to 7788775.58)	13311.53 (11798.1 to 14920.77)	-2.2 (-2.47 to -1.92)	0.74 (0.24 to 1.24)	0.871
Bahrain	17654.55 (15756.06 to 19770.84)	10872.88 (9703.66 to 12176.23)	-1.68 (-1.84 to -1.51)	1.16 (-0.24 to 2.58)	0.753
Belgium	637185.35 (581588.3 to 697240.75)	16853.31 (15382.79 to 18441.75)	-1.22 (-1.32 to -1.12)	-0.72 (-0.79 to -0.65)	0.854
Bhutan	15083.48 (13708.75 to 16647.54)	15145.56 (13765.17 to 16716.06)	0.01 (-0.01 to 0.04)	-0.04 (-0.5 to 0.43)	0.473
Cambodia	245550.23 (222007.06 to 272668.19)	11295.84 (10212.8 to 12543.33)	-0.32 (-0.34 to -0.3)	0.57 (0.44 to 0.7)	0.474
Denmark	342521.5 (310626.48 to 375163.03)	17791.27 (16134.57 to 19486.73)	-1.4 (-1.51 to -1.29)	0.27 (-0.06 to 0.6)	0.896
Eswatini	13305.74 (12011.29 to 14506.49)	14696.08 (13266.37 to 16022.29)	-0.67 (-0.75 to -0.58)	0.67 (0.65 to 0.69)	0.585
Lesotho	19301.5 (17127.49 to 21722.44)	10690.13 (9486.05 to 12030.96)	-0.09 (-0.16 to -0.01)	0.77 (0.73 to 0.82)	0.510
Morocco	754059.39 (672748.24 to 839435.27)	12550.95 (11197.57 to 13971.99)	0.45 (0.42 to 0.47)	0.91 (0.21 to 1.62)	0.563
Norway	323645.54 (294540.06 to 354870.63)	19956.74 (18162.03 to 21882.16)	-2.37 (-2.56 to -2.18)	-0.38 (-0.43 to -0.32)	0.916
Saudi Arabia	299899.78 (268054.24 to 332351.43)	9961.12 (8903.38 to 11039)	0.04 (-0.01 to 0.08)	1.25 (-0.27 to 2.79)	0.815
Spain	2918170.99 (2675490.08 to 3169660.32)	18857.23 (17289.02 to 20482.35)	-0.31 (-0.35 to -0.28)	-0.63 (-1.38 to 0.12)	0.769
Sweden	816652.17 (749744.98 to 888548.7)	24258.4 (22270.95 to 26394.07)	-1.62 (-1.84 to -1.4)	0.09 (0.03 to 0.16)	0.887
Thailand	2063618.7 (1850343.56 to 2278753.24)	10616.42 (9519.21 to 11723.2)	-1.35 (-1.42 to -1.28)	-0.22 (-1.95 to 1.54)	0.683

Netherlands	1045443.56 (964262.55 to 1125438.74)	17868.16 (16480.66 to 19235.4)	-0.69 (-0.79 to -0.58)	-1.94 (-2.29 to -1.59)	0.888
Tonga	1785.4 (1584.32 to 2005.49)	13293.6 (11796.45 to 14932.35)	-0.62 (-0.66 to -0.57)	-0.36 (-0.51 to -0.21)	0.626
Kyrgyzstan	107452.71 (95735.82 to 119498.63)	12705.01 (11319.63 to 14129.3)	-1.41 (-1.56 to -1.25)	-0.27 (-0.69 to 0.15)	0.604
Laos	89072.71 (79818.59 to 98847.25)	11520.77 (10323.83 to 12785.02)	-0.6 (-0.64 to -0.57)	0.83 (0.36 to 1.31)	0.489
Lebanon	169175.56 (151029.57 to 189194.85)	17262.31 (15410.73 to 19305.04)	0.54 (0.51 to 0.57)	-0.65 (-2.21 to 0.93)	0.745
Malaysia	474102.82 (418366.19 to 528456.47)	9670.06 (8533.23 to 10778.69)	-1.49 (-1.59 to -1.39)	1.31 (1.03 to 1.6)	0.743
Mongolia	44332.7 (39868.92 to 49636.8)	11228.98 (10098.36 to 12572.45)	-1.19 (-1.26 to -1.12)	0.08 (-0.65 to 0.82)	0.618
Montenegro	19848.99 (17467.89 to 22196.68)	11383.05 (10017.53 to 12729.41)	0.37 (0.27 to 0.48)	-0.29 (-0.75 to 0.17)	0.796
New Zealand	201754.68 (179521.28 to 226394.26)	14144.1 (12585.42 to 15871.47)	-2.25 (-2.45 to -2.05)	-1.61 (-3.27 to 0.07)	0.849
North Macedonia	120861.45 (110984.14 to 131259.04)	20384.6 (18718.68 to 22138.26)	-1.45 (-1.65 to -1.25)	0.3 (-0.05 to 0.65)	0.751
Northern Mariana Islands	731.97 (632.95 to 832.72)	7530.32 (6511.58 to 8566.79)	-1.05 (-1.24 to -0.85)	1.32 (1.04 to 1.61)	0.772
Palestine	54932.67 (49844.07 to 60747.45)	12767.31 (11584.63 to 14118.76)	-0.52 (-0.54 to -0.5)	0.68 (-0.36 to 1.73)	0.631
Algeria	852890.49 (763663.22 to 953288.28)	14043.12 (12573.96 to 15696.2)	0.18 (0.12 to 0.23)	0.56 (0.39 to 0.73)	0.660
Bangladesh	3558233.64 (3236190.22 to 3929454.1)	15228.16 (13849.91 to 16816.87)	-0.38 (-0.43 to -0.33)	0.57 (0.4 to 0.73)	0.492
China	47673490.77 (42477046.77 to 53050486.13)	12580.25 (11208.99 to 13999.15)	-0.61 (-0.67 to -0.56)	-0.26 (-0.38 to -0.14)	0.722
Bolivia	154480.29 (136242.49 to 175003.44)	9995.67 (8815.59 to 11323.63)	-0.02 (-0.06 to 0.01)	-0.47 (-0.57 to -0.36)	0.599
Portugal	892033.74 (820859.31 to 970357.74)	22927.66 (21098.29 to 24940.8)	-1 (-1.13 to -0.87)	0.19 (0.09 to 0.29)	0.744
Andorra	4892.44 (4461.4 to 5354.26)	18488.73 (16859.8 to 20233.95)	-0.78 (-0.82 to -0.74)	-0.46 (-0.51 to -0.42)	0.869
Monaco	2840.02 (2582.2 to 3138.75)	18152.51 (16504.57 to 20061.86)	-0.34 (-0.4 to -0.28)	-0.38 (-0.46 to -0.29)	0.908
Puerto Rico	163583.14 (147744.89 to 178525.06)	14013.03 (12656.28 to 15293)	-0.79 (-0.9 to -0.69)	0.83 (-0.09 to 1.77)	0.826
Albania	108670.96 (96750.84 to 121951.34)	13820.65 (12304.66 to 15509.64)	-0.15 (-0.26 to -0.04)	0.32 (0.32 to 0.33)	0.707
Angola	170310.74 (152313.67 to 189521.25)	8807.82 (7877.08 to 9801.32)	-1.04 (-1.09 to -0.98)	0.94 (0.3 to 1.57)	0.454
Armenia	91751.66 (81308.09 to 103004.38)	11664.05 (10336.4 to 13094.57)	0.18 (0 to 0.37)	-0.37 (-1.77 to 1.05)	0.702
Austria	565232.88 (518750.58 to 614547.85)	19126.9 (17553.98 to 20795.66)	-0.95 (-0.99 to -0.91)	-0.72 (-1.55 to 0.13)	0.854
Azerbaijan	214612.24 (191581.5 to 238836.51)	11202.23 (10000.08 to 12466.68)	-0.81 (-0.91 to -0.71)	-0.25 (-0.56 to 0.07)	0.695
Belarus	396563.32 (354153.59 to 439544.43)	13785.82 (12311.52 to 15279.98)	-1.83 (-1.91 to -1.75)	0.17 (-0.12 to 0.47)	0.784
Benin	76590.79 (68950.11 to 85027.12)	9260.59 (8336.75 to 10280.62)	-0.51 (-0.59 to -0.43)	0.83 (0.7 to 0.97)	0.373
Botswana	28213.06 (25420.88 to 31239.75)	11637.93 (10486.15 to 12886.44)	-0.62 (-0.69 to -0.55)	0.76 (0.49 to 1.04)	0.643
Bulgaria	352186.95 (311274.35 to 395064.98)	14817.02 (13095.77 to 16620.96)	-0.91 (-0.98 to -0.83)	0.82 (-0.03 to 1.67)	0.768
Burundi	90635.08 (82220.89 to 99981.31)	11321.91 (10270.83 to 12489.42)	-0.74 (-0.83 to -0.65)	0.36 (0.27 to 0.46)	0.289
Cabo Verde	4397.69 (3958.29 to 4885.4)	5737.65 (5164.37 to 6373.96)	-0.75 (-0.86 to -0.64)	0.14 (0.05 to 0.22)	0.534
Cameroon	148177.42 (130185.74 to 167849.7)	7279.74 (6395.83 to 8246.21)	-0.28 (-0.34 to -0.22)	1.02 (0.34 to 1.7)	0.480
Chad	85025.72 (75414 to 95521.95)	9157.1 (8121.94 to 10287.52)	-0.1 (-0.17 to -0.03)	0.75 (0.73 to 0.78)	0.240

Chile	774943.44 (706813.66 to 847070.24)	17306.8 (15785.26 to 18917.6)	-0.83 (-0.95 to -0.72)	1.05 (0.12 to 1.99)	0.772
Colombia	1211249.75 (1090503.77 to 1330213.17)	12665.34 (11402.76 to 13909.27)	-0.65 (-0.7 to -0.6)	0.47 (0.06 to 0.87)	0.655
Costa Rica	130835.11 (117214.04 to 144773.06)	13664.77 (12242.15 to 15120.49)	-0.48 (-0.54 to -0.43)	0.46 (0.1 to 0.83)	0.700
Côte d'Ivoire	171578.8 (154337.35 to 191863.8)	9568.4 (8606.9 to 10699.62)	-0.14 (-0.22 to -0.06)	1.12 (0.63 to 1.6)	0.426
Croatia	262025.3 (239637.03 to 282838.73)	17569.95 (16068.72 to 18965.58)	-0.69 (-0.74 to -0.64)	0.44 (0.16 to 0.73)	0.798
Cuba	438801.7 (400080.7 to 481969.8)	12841.55 (11708.37 to 14104.86)	0.69 (0.57 to 0.8)	-1.53 (-2.16 to -0.9)	0.669
Cyprus	72600.89 (64666.52 to 80746.64)	20772.69 (18502.49 to 23103.36)	-0.59 (-0.6 to -0.57)	-0.04 (-1.36 to 1.31)	0.836
Djibouti	8378.6 (7530.98 to 9277.55)	8079.35 (7262 to 8946.19)	-0.68 (-0.79 to -0.58)	1.07 (0.82 to 1.32)	0.488
Ecuador	253881.95 (222005.78 to 288365.24)	9173.63 (8021.83 to 10419.63)	0.18 (0.15 to 0.21)	-0.19 (-0.28 to -0.09)	0.661
El Salvador	149944.1 (134357.26 to 166616.61)	14663.26 (13139 to 16293.69)	-0.12 (-0.17 to -0.07)	0.09 (-0.65 to 0.84)	0.564
Equatorial Guinea	7349.7 (6587.19 to 8172.69)	9292.02 (8327.99 to 10332.5)	-0.93 (-1.03 to -0.83)	0.25 (-0.11 to 0.61)	0.658
Estonia	47093.88 (42682.81 to 52188.81)	10764.92 (9756.62 to 11929.54)	-0.18 (-0.25 to -0.11)	-1.76 (-3.46 to -0.03)	0.845
Fiji	13077.28 (11713.11 to 14655.9)	9563.57 (8565.93 to 10718.03)	-1.61 (-1.68 to -1.54)	0.08 (-1.24 to 1.41)	0.675
Finland	369999.98 (337620.08 to 404276.98)	18383.57 (16774.76 to 20086.63)	-0.37 (-0.41 to -0.33)	0.92 (0.21 to 1.63)	0.860
Ghana	221544.24 (197259.84 to 246907.38)	8123.59 (7233.13 to 9053.61)	0.2 (0.16 to 0.24)	1.12 (1.07 to 1.17)	0.565
Guatemala	181610.91 (161568.65 to 204598.11)	9919.9 (8825.16 to 11175.5)	0.01 (-0.04 to 0.05)	0.84 (0.79 to 0.88)	0.540
Guinea	93738.23 (84044.91 to 104036.56)	10260.78 (9199.73 to 11388.06)	-0.07 (-0.12 to -0.02)	0.59 (0.16 to 1.02)	0.336
Guinea-Bissau	11490.89 (10390.68 to 12862.77)	10003.36 (9045.57 to 11197.64)	-0.49 (-0.6 to -0.37)	0.71 (0.28 to 1.13)	0.353
Guyana	9773.11 (8808.58 to 10789.07)	8678.15 (7821.68 to 9580.28)	-0.69 (-0.75 to -0.64)	0.87 (0.19 to 1.56)	0.651
Haiti	157137.71 (142888.53 to 173299.47)	13259.58 (12057.2 to 14623.34)	-0.45 (-0.49 to -0.42)	-0.11 (-0.27 to 0.05)	0.448
Honduras	136278.53 (121801.6 to 154006.2)	12958.31 (11581.74 to 14643.97)	0.33 (0.28 to 0.39)	0.03 (-0.14 to 0.2)	0.513
Iceland	18862.15 (17261.61 to 20431.57)	19368.4 (17724.91 to 20979.95)	-1.46 (-1.55 to -1.38)	0.32 (0.13 to 0.5)	0.876
India	34725057.6 (31867270.84 to 37363934.28)	17272.25 (15850.79 to 18584.83)	0.08 (0.03 to 0.13)	0.25 (0.17 to 0.32)	0.575
Indonesia	4681338.73 (4161367.16 to 5232045.54)	11178.1 (9936.51 to 12493.08)	-0.3 (-0.4 to -0.2)	0.13 (0.09 to 0.17)	0.657
Iraq	421480.59 (379065.22 to 471866.7)	10848.39 (9756.67 to 12145.26)	-0.81 (-0.86 to -0.75)	0.55 (-0.47 to 1.59)	0.663
Italy	3661627.27 (3249096.96 to 4072415.5)	16118.59 (14302.62 to 17926.89)	-1.68 (-1.85 to -1.51)	-0.78 (-1.4 to -0.15)	0.806
Kazakhstan	420933.11 (382233.26 to 468339.99)	13264.23 (12044.74 to 14758.09)	0.28 (0.17 to 0.4)	-0.21 (-0.51 to 0.1)	0.725
Kenya	307155.52 (273301.06 to 341580.86)	8211.02 (7306.01 to 9131.29)	-0.33 (-0.36 to -0.3)	0.59 (0.2 to 0.99)	0.524
Kiribati	1713.11 (1570.31 to 1879.88)	13621.12 (12485.69 to 14947.12)	-1.04 (-1.09 to -0.98)	-0.13 (-0.24 to -0.02)	0.527
South Korea	2421131.63 (2251208.61 to 2599440.96)	14477.2 (13461.14 to 15543.4)	-0.56 (-0.7 to -0.42)	-0.67 (-1.39 to 0.05)	0.887
Latvia	76180.87 (68243.65 to 84257.57)	11577.3 (10371.07 to 12804.72)	-1.05 (-1.26 to -0.84)	0.89 (0.51 to 1.27)	0.831
Liberia	28277.85 (25112.32 to 31789.5)	8565.89 (7606.99 to 9629.63)	-0.04 (-0.13 to 0.06)	0.37 (0.11 to 0.65)	0.352
Lithuania	110340.04 (98880.3 to 122507.1)	11401.35 (10217.23 to 12658.57)	-0.76 (-0.86 to -0.67)	0.74 (0.27 to 1.21)	0.856

Madagascar	213330.71 (194896.73 to 235685)	11552.12 (10553.9 to 12762.63)	-0.34 (-0.45 to -0.23)	0.43 (0.09 to 0.77)	0.400
Malawi	111666.81 (100619.21 to 124582.03)	9551.29 (8606.35 to 10655.98)	-0.01 (-0.07 to 0.05)	1.22 (1.06 to 1.38)	0.385
Maldives	5852.55 (5217.86 to 6602.54)	10759.08 (9592.28 to 12137.83)	-0.56 (-0.65 to -0.48)	0.08 (-0.55 to 0.71)	0.651
Mali	132657.3 (117950.93 to 149199.79)	9165.38 (8149.31 to 10308.32)	0.08 (0 to 0.16)	1.06 (0.96 to 1.16)	0.269
Malta	28858.36 (26149.71 to 31596.48)	18287.56 (16571.09 to 20022.71)	-1.47 (-1.59 to -1.35)	0.92 (0.61 to 1.24)	0.802
Mauritius	34890.8 (31831.54 to 38179.46)	10312.6 (9408.38 to 11284.62)	-1.46 (-1.57 to -1.34)	1.1 (1.05 to 1.15)	0.718
Moldova	113930.28 (100405.81 to 129259.56)	10790.56 (9509.63 to 12242.42)	-1.74 (-1.84 to -1.65)	0.67 (0.64 to 0.7)	0.732
Mozambique	163146.97 (147490.49 to 180670.33)	9118.77 (8243.68 to 10098.2)	-0.13 (-0.21 to -0.05)	0.76 (0.48 to 1.05)	0.326
Namibia	25127.55 (22669.82 to 27904.26)	11121 (10033.25 to 12349.92)	-0.45 (-0.5 to -0.4)	0.19 (-0.09 to 0.47)	0.618
Nauru	92.96 (82.9 to 104.42)	10057.71 (8969.39 to 11297.3)	-0.97 (-1.05 to -0.88)	0.5 (0.29 to 0.71)	0.625
Nicaragua	102677.61 (92628.48 to 112802.74)	12613.44 (11378.95 to 13857.26)	-0.18 (-0.28 to -0.09)	0.01 (-1.13 to 1.17)	0.524
Niue	37.16 (32.51 to 42.59)	9496.64 (8308.58 to 10885.03)	-1.23 (-1.28 to -1.18)	-0.12 (-0.12 to -0.12)	0.726
Palau	349.43 (307.84 to 392.51)	8344.88 (7351.63 to 9373.79)	-1.7 (-1.79 to -1.61)	0.12 (-0.47 to 0.71)	0.754
Panama	82579.23 (74251.63 to 91197.87)	11105.86 (9985.9 to 12264.95)	-0.02 (-0.09 to 0.05)	1.31 (1.28 to 1.33)	0.709
Paraguay	120588.55 (108023.3 to 135104.32)	12179.37 (10910.29 to 13645.45)	0.18 (0.17 to 0.19)	0.65 (0.01 to 1.3)	0.636
Peru	520775.77 (457029.82 to 582448.57)	9310.47 (8170.81 to 10413.06)	0.5 (0.39 to 0.61)	-2.95 (-5.78 to -0.03)	0.662
Poland	2502376.08 (2282212.99 to 2739618.23)	20654.59 (18837.36 to 22612.78)	-2.31 (-2.62 to -1.99)	0.85 (0.54 to 1.17)	0.812
Rwanda	162224.71 (144877 to 180995.52)	15340.89 (13700.39 to 17115.96)	-0.96 (-1.1 to -0.81)	1.35 (1.27 to 1.43)	0.436
San Marino	2050.78 (1850.46 to 2274.78)	17339.8 (15646.03 to 19233.78)	-0.18 (-0.22 to -0.14)	-0.49 (-1.11 to 0.13)	0.888
Senegal	113742.26 (101065.12 to 128504.9)	8809.27 (7827.43 to 9952.63)	0 (-0.06 to 0.06)	0.88 (0.83 to 0.94)	0.408
Serbia	463415.92 (419335.99 to 508140.84)	16519.77 (14948.42 to 18114.12)	0.26 (0.23 to 0.29)	0.5 (0.4 to 0.59)	0.792
Seychelles	1905.06 (1692.85 to 2134.32)	9184.14 (8161.11 to 10289.37)	-0.55 (-0.68 to -0.41)	0.48 (-0.06 to 1.02)	0.730
Sierra Leone	58318.55 (52496.14 to 64901.72)	9654.05 (8690.21 to 10743.83)	-0.27 (-0.33 to -0.2)	0.99 (0.65 to 1.32)	0.359
Singapore	121431.1 (109903.14 to 133695.88)	7995.06 (7236.05 to 8802.57)	-3.09 (-3.33 to -2.85)	0.46 (-0.02 to 0.94)	0.856
Slovenia	124895.29 (112539.93 to 137598.15)	17057.8 (15370.34 to 18792.72)	-1.04 (-1.11 to -0.97)	-0.13 (-0.97 to 0.72)	0.842
South Africa	835981.89 (742925.08 to 933305.86)	10597.41 (9417.76 to 11831.14)	-0.44 (-0.56 to -0.32)	-0.12 (-0.36 to 0.13)	0.680
South Sudan	59004.14 (53482.84 to 65656.57)	9475.19 (8588.55 to 10543.47)	-0.64 (-0.69 to -0.59)	-0.18 (-0.34 to -0.02)	0.278
Sudan	390841.84 (347630.24 to 440833.02)	12551.78 (11164.05 to 14157.24)	-0.68 (-0.75 to -0.61)	-0.42 (-0.46 to -0.39)	0.542
Suriname	9734.85 (8690.5 to 10943.61)	8714.96 (7780.02 to 9797.08)	-0.22 (-0.3 to -0.14)	0.2 (-0.18 to 0.57)	0.634
Tajikistan	121586.56 (109624.26 to 135454.81)	11837.33 (10672.72 to 13187.51)	-0.96 (-1.08 to -0.83)	-0.35 (-0.56 to -0.14)	0.542
Republic of the Congo	44609.6 (40124.02 to 49313.59)	10266.21 (9233.92 to 11348.76)	-0.69 (-0.74 to -0.64)	0.49 (0.18 to 0.8)	0.583
Gambia	15801.79 (14240.05 to 17572.57)	10218.42 (9208.5 to 11363.52)	0.08 (0.01 to 0.14)	0.71 (0.66 to 0.76)	0.410
Marshall Islands	544.98 (488.05 to 607.68)	9301.6 (8329.85 to 10371.69)	-1.6 (-1.69 to -1.5)	0.79 (0.71 to 0.87)	0.574

Niger	144793.34<br>(129316.31 to 162888.45)	10400.22<br>(9288.53 to 11699.95)	0.02<br>(-0.1 to 0.13)	0.88<br>(0.73 to 1.04)	0.168
Philippines	1820290.97<br>(1641688.94 to 2009557.25)	13053.29<br>(11772.54 to 14410.52)	-1.24<br>(-1.32 to -1.17)	0.31<br>(-0.14 to 0.75)	0.651
Myanmar	1109363.51<br>(1005433.98 to 1229952.94)	13140.81<br>(11909.73 to 14569.24)	-0.48<br>(-0.53 to -0.43)	0.23<br>(0.08 to 0.37)	0.534
Trinidad and Tobago	25925.49<br>(22708.59 to 29303.15)	7442.61<br>(6519.11 to 8412.26)	-0.31<br>(-0.38 to -0.24)	1.13<br>(0.72 to 1.53)	0.769
Tunisia	360438.95<br>(327206.22 to 396584.28)	15422.26<br>(14000.32 to 16968.83)	0.16<br>(0.09 to 0.24)	0.49<br>(-0.26 to 1.25)	0.682
Turkey	3259794.08<br>(3031127.83 to 3513448.92)	19730.91<br>(18346.84 to 21266.24)	-0.24<br>(-0.28 to -0.2)	0.13<br>(-0.09 to 0.35)	0.713
Uganda	257306.86<br>(231440.95 to 284573.03)	10850<br>(9759.3 to 11999.74)	-0.63<br>(-0.69 to -0.58)	0.73<br>(0.71 to 0.76)	0.423
Uzbekistan	636616.17<br>(573532.34 to 704793.14)	13694.64<br>(12337.61 to 15161.24)	-1.45<br>(-1.59 to -1.31)	-0.04<br>(-0.2 to 0.13)	0.663
Vanuatu	3209.74<br>(2884.53 to 3547.98)	11193.64<br>(10059.47 to 12373.19)	-1.09<br>(-1.14 to -1.04)	0.17<br>(0.11 to 0.23)	0.473
Yemen	353249.88<br>(320657.3 to 388527.21)	15707.49<br>(14258.24 to 17276.12)	-0.9<br>(-0.96 to -0.83)	0.29<br>(-0.33 to 0.91)	0.450
Zambia	79893.5<br>(70252.03 to 90508.81)	7396.46<br>(6503.86 to 8379.22)	-0.11<br>(-0.16 to -0.07)	1.02<br>(0.26 to 1.79)	0.506
Zimbabwe	112337.53<br>(100276.01 to 125856.71)	10145.44<br>(9056.14 to 11366.38)	0<br>(-0.07 to 0.07)	0.68<br>(0.05 to 1.32)	0.474
Romania	947751.35<br>(857502.72 to 1050376.81)	15781.78<br>(14278.98 to 17490.68)	-0.78<br>(-0.83 to -0.72)	0.52<br>(-0.46 to 1.52)	0.768
Russia	4763881.06<br>(4216598.03 to 5338570.24)	11207.75<br>(9920.19 to 12559.8)	-1.93<br>(-2.05 to -1.81)	1.69<br>(1.02 to 2.37)	0.809
Saint Kitts and Nevis	1012.7<br>(900.18 to 1137.46)	7862.98<br>(6989.36 to 8831.66)	-0.71<br>(-0.87 to -0.56)	-0.47<br>(-1.19 to 0.25)	0.755
Saint Lucia	4726.73<br>(4248.97 to 5152.16)	11232.22<br>(10096.92 to 12243.19)	-0.59<br>(-0.63 to -0.55)	-0.11<br>(-0.4 to 0.19)	0.673
Saint Vincent and the Grenadines	2118.32<br>(1919.57 to 2343.02)	8331.43<br>(7549.72 to 9215.19)	-0.18<br>(-0.22 to -0.15)	-0.14<br>(-0.62 to 0.34)	0.637
Slovakia	201487.39<br>(180810.4 to 224057.98)	12283.74<br>(11023.16 to 13659.77)	-0.04<br>(-0.09 to 0.01)	0.07<br>(-0.86 to 1.01)	0.811
Vietnam	2331929.48<br>(2119593.55 to 2566677.87)	13349.27<br>(12133.74 to 14693.1)	-0.28<br>(-0.35 to -0.2)	1.03<br>(0.88 to 1.17)	0.628
Solomon Islands	6888.33<br>(6264.43 to 7600.55)	12698.39<br>(11548.25 to 14011.34)	-0.59<br>(-0.63 to -0.54)	-0.33<br>(-0.45 to -0.2)	0.429
Eritrea	43436.89<br>(39182.93 to 48260.19)	9858.37<br>(8892.9 to 10953.06)	-0.7<br>(-0.75 to -0.64)	0.6<br>(0.29 to 0.91)	0.404
Israel	300608.05<br>(269315.48 to 330530.38)	15041.35<br>(13475.58 to 16538.55)	-1.14<br>(-1.23 to -1.05)	0.09<br>(-0.33 to 0.51)	0.809
Kuwait	47040.11<br>(41842 to 52634.37)	10089.6<br>(8974.66 to 11289.51)	-0.25<br>(-0.42 to -0.07)	-0.48<br>(-1.05 to 0.09)	0.847
Libya	106291<br>(96528.31 to 117836.81)	12709.42<br>(11542.08 to 14089.98)	-0.16<br>(-0.2 to -0.12)	-1.71<br>(-2.55 to -0.87)	0.726
Qatar	14258.71<br>(12694.68 to 15991.61)	9308.22<br>(8287.2 to 10439.47)	-0.63<br>(-0.71 to -0.54)	1.64<br>(0.27 to 3.03)	0.847
Oman	35900.04<br>(32002.35 to 39979.64)	11493.36<br>(10245.52 to 12799.44)	-0.19<br>(-0.22 to -0.17)	0.12<br>(-0.78 to 1.03)	0.773
Switzerland	541062.08<br>(492993.49 to 592038.58)	18461.02<br>(16820.92 to 20200.34)	-1.16<br>(-1.2 to -1.12)	-0.88<br>(-0.93 to -0.84)	0.933
Syria	314163.58<br>(282904.79 to 349494.8)	13296.79<br>(11973.78 to 14792.16)	0.24<br>(0.18 to 0.3)	0.54<br>(-0.12 to 1.2)	0.623
Taiwan	947702.57<br>(867016.84 to 1027929.04)	12585.8<br>(11514.27 to 13651.23)	-1.56<br>(-1.64 to -1.47)	0.43<br>(0.22 to 0.63)	0.875
Togo	62404.46<br>(55250.17 to 70619.49)	9989.71<br>(8844.45 to 11304.77)	-0.02<br>(-0.1 to 0.05)	0.8<br>(0.73 to 0.88)	0.409
Tokelau	26.22<br>(23.04 to 29.56)	10373.31<br>(9117.92 to 11696.4)	-1<br>(-1.09 to -0.91)	-0.12<br>(-0.37 to 0.14)	0.686
Turkmenistan	52036.1<br>(45802.78 to 59048.7)	7352.84<br>(6472.05 to 8343.73)	-2.29<br>(-2.38 to -2.2)	0.19<br>(-0.86 to 1.25)	0.682
Tuvalu	187<br>(165.33 to 210.5)	10261.46<br>(9072.28 to 11551.4)	-1.34<br>(-1.43 to -1.25)	0.64<br>(0.29 to 0.99)	0.577

Ukraine	1367643.98 (1187927.18 to 1560380.25)	10072.45 (8748.87 to 11491.91)	-1.81 (-1.89 to -1.73)	0.53 (0.49 to 0.57)	0.761
Comoros	7971.59 (7143.28 to 8900.55)	9862.11 (8837.36 to 11011.36)	-0.47 (-0.56 to -0.39)	0.44 (-0.23 to 1.12)	0.476
UAE	128843.47 (116142.26 to 143421.57)	17942.8 (16174.03 to 19972.96)	-1.65 (-1.79 to -1.52)	0.49 (-0.49 to 1.48)	0.849
UK	5241045.13 (4880834.22 to 5612767.37)	24921.53 (23208.71 to 26689.1)	-0.83 (-0.94 to -0.73)	-0.38 (-0.83 to 0.07)	0.859
Mexico	2398613.12 (2113374.16 to 2698792.81)	11130.23 (9806.64 to 12523.14)	-0.44 (-0.46 to -0.42)	-1.02 (-1.73 to -0.3)	0.665
Tanzania	458213.18 (402057.76 to 524013.61)	11115.79 (9753.52 to 12712.05)	-0.11 (-0.15 to -0.06)	0.68 (0.41 to 0.94)	0.447
USA	27457355.03 (25987761.5 to 28905522.88)	27389.5 (25923.53 to 28834.08)	0.43 (0.26 to 0.6)	0.13 (-0.01 to 0.28)	0.862
US Virgin Islands	3391.38 (3018.78 to 3750.83)	10535.75 (9378.22 to 11652.42)	0.3 (0.16 to 0.44)	0.97 (0.91 to 1.03)	0.822

**Cause**	**COPD**				
**Location**	**Prevalence number**	**Prevalence rate**	**EAPC 1990-2021(95% CI)**	**EAPC 2019-2021(95% CI)**	**2021SDI**
American Samoa	614.41 (528.18 to 706.44)	7274.41 (6253.49 to 8364.03)	-0.45 (-0.51 to -0.38)	-0.01 (-0.28 to 0.25)	0.724
Antigua and Barbuda	1030.84 (866.67 to 1197.72)	5463.54 (4593.42 to 6348.01)	0.25 (0.19 to 0.3)	0.5 (0.48 to 0.51)	0.750
Egypt	962115.06 (820055.36 to 1109951.47)	8694.45 (7410.69 to 10030.42)	0.82 (0.78 to 0.86)	0.82 (0.15 to 1.48)	0.607
Argentina	723379.87 (632775.44 to 828791.22)	7750.52 (6779.75 to 8879.93)	0.37 (0.23 to 0.51)	-0.45 (-0.8 to -0.09)	0.723
Australia	635660.08 (558394.56 to 738713.11)	8580.93 (7537.9 to 9972.06)	-0.77 (-0.88 to -0.67)	-1.86 (-3.99 to 0.32)	0.844
Barbados	5417.14 (4633.13 to 6225.57)	5945.14 (5084.71 to 6832.36)	0.3 (0.22 to 0.38)	0.43 (-0.6 to 1.48)	0.747
Belize	3548.14 (3039.09 to 4058.75)	7084.04 (6067.71 to 8103.49)	0.36 (0.32 to 0.4)	-0.65 (-0.97 to -0.33)	0.610
Bermuda	1745.44 (1475.87 to 2028.19)	7494.1 (6336.68 to 8708.1)	0.9 (0.83 to 0.97)	0.61 (-0.32 to 1.54)	0.821
Venezuela	487865.18 (434625.55 to 550187.48)	9324.3 (8306.76 to 10515.44)	0.77 (0.65 to 0.9)	0.24 (-0.87 to 1.36)	0.597
Bosnia and Herzegovina	134515.13 (117838.04 to 152522.9)	12281.47 (10758.83 to 13925.62)	0.89 (0.77 to 1.01)	0.05 (-0.75 to 0.85)	0.723
Brunei	3834.83 (3298.9 to 4384.44)	6404.77 (5509.68 to 7322.71)	-1.77 (-1.88 to -1.66)	0.56 (0.39 to 0.74)	0.810
Burkina Faso	88468.3 (75931.98 to 102565.64)	5884.49 (5050.64 to 6822.18)	0.33 (0.29 to 0.38)	0.82 (0.44 to 1.2)	0.285
Canada	1401495.06 (1252107.3 to 1569679.62)	11421.02 (10203.64 to 12791.59)	-0.12 (-0.44 to 0.2)	0.2 (-1.02 to 1.43)	0.873
Central African Republic	26552.15 (22791.11 to 30710.04)	7423.05 (6371.6 to 8585.45)	0.21 (0.19 to 0.23)	0.55 (0.23 to 0.88)	0.309
Dominica	927.72 (778.58 to 1082.59)	6150.82 (5162.05 to 7177.62)	0.14 (0.09 to 0.19)	0.01 (-0.7 to 0.73)	0.747
Bahamas	3764.43 (3261.04 to 4335.95)	5231.38 (4531.83 to 6025.62)	0.47 (0.4 to 0.54)	-1 (-1.65 to -0.34)	0.805
Cook Islands	363.6 (299.81 to 425.03)	7714.83 (6361.4 to 9018.37)	-0.11 (-0.17 to -0.05)	0.69 (-0.01 to 1.38)	0.779
Czech Republic	450350.9 (398941.95 to 495024.64)	12891.24 (11419.67 to 14170.02)	0.98 (0.9 to 1.06)	0.5 (0.28 to 0.72)	0.828
North Korea	662670.93 (585993.51 to 743685.65)	11758.3 (10397.76 to 13195.81)	0.06 (-0.04 to 0.16)	-0.83 (-1.06 to -0.59)	0.570
Sao Tome and Principe	1422.23 (1232.13 to 1641.19)	7778.61 (6738.88 to 8976.16)	0.47 (0.35 to 0.59)	0.28 (-0.21 to 0.77)	0.505
DR Congo	445996.42 (384203.1 to 516776.75)	7384.27 (6361.17 to 8556.17)	0.44 (0.36 to 0.53)	0.63 (0.21 to 1.05)	0.383
Timor-Leste	13363.91 (11600.27 to 15416.58)	9409.14 (8167.42 to 10854.37)	0.25 (0.17 to 0.33)	1.6 (0.07 to 3.16)	0.445

Sri Lanka	383879.1<br>(323618.71 to 446704.67)	7970.41<br>(6719.23 to 9274.84)	0.25<br>(0.18 to 0.32)	1.96<br>(1.78 to 2.14)	0.702
Dominican Republic	122341.5<br>(103749.55 to 142213.12)	7321.35<br>(6208.74 to 8510.54)	1.04<br>(0.97 to 1.1)	-0.06<br>(-0.37 to 0.24)	0.619
Uruguay	71367.96<br>(61056.41 to 81672.96)	7892.13<br>(6751.84 to 9031.7)	0.38<br>(0.25 to 0.51)	-1.36<br>(-3.12 to 0.43)	0.719
Ethiopia	453815.97<br>(387413.21 to 522956.49)	6629.33<br>(5659.32 to 7639.33)	-0.05<br>(-0.14 to 0.04)	1.05<br>(0.28 to 1.83)	0.359
Nepal	512188.32<br>(473195.98 to 546948.79)	12941.06<br>(11955.87 to 13819.32)	0.38<br>(0.33 to 0.43)	-0.25<br>(-0.54 to 0.04)	0.433
Germany	4643490.32<br>(4175299.61 to 5180086.66)	14734.74<br>(13249.07 to 16437.47)	0.41<br>(0.28 to 0.54)	-0.46<br>(-1.15 to 0.25)	0.903
Nigeria	988718.63<br>(837298.07 to 1140327.73)	6590.79<br>(5581.42 to 7601.42)	0.44<br>(0.39 to 0.5)	0.73<br>(0.59 to 0.87)	0.503
Somalia	71295.35<br>(60206.24 to 82839.96)	7560<br>(6384.13 to 8784.16)	0.49<br>(0.37 to 0.61)	1.65<br>(1.62 to 1.68)	0.078
Micronesia	964.97<br>(828.59 to 1126.82)	7333.05<br>(6296.7 to 8562.99)	-1.14<br>(-1.21 to -1.06)	0.36<br>(0.17 to 0.55)	0.588
Brazil	4951601.61<br>(4292708.43 to 5626859.31)	11433.52<br>(9912.1 to 12992.73)	0.14<br>(0.06 to 0.21)	0.9<br>(-0.62 to 2.44)	0.653
France	2595191.6<br>(2291248.83 to 2941712.84)	11737.57<br>(10362.9 to 13304.82)	0.29<br>(0.26 to 0.32)	-0.26<br>(-0.39 to -0.12)	0.838
Gabon	10269.36<br>(8701.29 to 12169.01)	5745.03<br>(4867.8 to 6807.76)	0.15<br>(0.09 to 0.22)	1.05<br>(0.87 to 1.23)	0.635
Georgia	101571.27<br>(86108.19 to 115895.28)	9674.67<br>(8201.81 to 11039.03)	1.11<br>(1.02 to 1.2)	-0.01<br>(-0.35 to 0.34)	0.732
Luxembourg	22326.87<br>(19673.93 to 25065.96)	12564.22<br>(11071.3 to 14105.61)	0.01<br>(-0.09 to 0.11)	-0.66<br>(-0.91 to -0.4)	0.884
Greenland	1401.9<br>(1262.41 to 1557.42)	10326.3<br>(9298.8 to 11471.85)	-0.19<br>(-0.23 to -0.15)	-0.42<br>(-0.6 to -0.24)	0.826
Grenada	1219.9<br>(1042.27 to 1395.92)	6073.02<br>(5188.72 to 6949.3)	-0.12<br>(-0.26 to 0.02)	-0.1<br>(-0.14 to -0.06)	0.669
Guam	2680.54<br>(2202.33 to 3097.18)	7112.92<br>(5843.97 to 8218.51)	0.36<br>(0.3 to 0.42)	0.52<br>(0.37 to 0.67)	0.804
Jordan	99613.58<br>(84523.61 to 115782.24)	7923.19<br>(6722.94 to 9209.23)	0.31<br>(0.26 to 0.36)	-0.12<br>(-0.47 to 0.23)	0.725
Greece	539163.02<br>(475700.78 to 611126.83)	14669.31<br>(12942.66 to 16627.27)	0.88<br>(0.84 to 0.92)	-0.03<br>(-0.91 to 0.87)	0.792
Hungary	468212.83<br>(412885.77 to 514039.54)	14723.1<br>(12983.32 to 16164.14)	0.87<br>(0.84 to 0.9)	0.17<br>(0.08 to 0.25)	0.791
Papua New Guinea	78874.57<br>(70524.59 to 88098.55)	9603.59<br>(8586.92 to 10726.68)	-0.27<br>(-0.35 to -0.19)	-0.32<br>(-0.33 to -0.31)	0.418
Samoa	2150.43<br>(1840.09 to 2499.53)	8733.92<br>(7473.5 to 10151.82)	-0.43<br>(-0.52 to -0.33)	0.13<br>(-0.36 to 0.63)	0.593
Ireland	163321.47<br>(146117.83 to 183433.55)	12444.79<br>(11133.9 to 13977.29)	-0.36<br>(-0.4 to -0.31)	1.08<br>(0.78 to 1.38)	0.874
Afghanistan	128456.48<br>(111176.26 to 148962.48)	10454.23<br>(9047.91 to 12123.08)	0.66<br>(0.59 to 0.72)	-1.76<br>(-2.61 to -0.9)	0.337
Iran	1073078.33<br>(916124.38 to 1239504.12)	8260.2<br>(7052.02 to 9541.3)	1.44<br>(1.34 to 1.54)	-0.37<br>(-0.97 to 0.22)	0.697
Mauritania	20162.85<br>(17082.28 to 23947.91)	5620.19<br>(4761.51 to 6675.23)	0.33<br>(0.26 to 0.4)	1.26<br>(0.72 to 1.79)	0.499
Pakistan	2011521.6<br>(1740667.84 to 2295781.69)	10145.88<br>(8779.72 to 11579.65)	-0.27<br>(-0.31 to -0.23)	-0.82<br>(-1.32 to -0.33)	0.504
Jamaica	40316.7<br>(35558.63 to 45401.87)	7622.86<br>(6723.23 to 8584.34)	0.38<br>(0.26 to 0.5)	-0.11<br>(-0.77 to 0.55)	0.683
Japan	4796380.88<br>(3984854.1 to 5698963.36)	9188.31<br>(7633.69 to 10917.36)	0.69<br>(0.63 to 0.75)	0.81<br>(-0.04 to 1.66)	0.871
Bahrain	13137.99<br>(11159.55 to 15320.56)	8091.27<br>(6872.82 to 9435.45)	-0.61<br>(-0.78 to -0.44)	1.28<br>(-0.32 to 2.9)	0.753
Belgium	490834.24<br>(436211.29 to 550268.2)	12982.38<br>(11537.62 to 14554.38)	0.35<br>(0.27 to 0.44)	-1.02<br>(-1.13 to -0.92)	0.854
Bhutan	12353.5<br>(10808.6 to 13889.8)	12404.34<br>(10853.08 to 13946.97)	0.42<br>(0.38 to 0.47)	-0.22<br>(-0.83 to 0.39)	0.473
Cambodia	179966.51<br>(155806.83 to 207862.84)	8278.85<br>(7167.45 to 9562.14)	0.06<br>(0.05 to 0.07)	0.49<br>(0.4 to 0.58)	0.474

Denmark	271919.09<br>(239423.44 to 303563.51)	14124.03<br>(12436.14 to 15767.71)	-0.32<br>(-0.44 to -0.21)	0.3<br>(-0.16 to 0.76)	0.896
Eswatini	6513.14<br>(5589.23 to 7540.38)	7193.71<br>(6173.26 to 8328.29)	-0.16<br>(-0.28 to -0.04)	0.5<br>(0.21 to 0.79)	0.585
Lesotho	16208.18<br>(14016.7 to 18673.55)	8976.9<br>(7763.15 to 10342.34)	0.01<br>(-0.1 to 0.11)	0.82<br>(0.56 to 1.08)	0.510
Morocco	561181.18<br>(480128.9 to 642541.32)	9340.59<br>(7991.51 to 10694.79)	1.04<br>(0.99 to 1.09)	0.68<br>(0.14 to 1.22)	0.563
Norway	236331.59<br>(206372.07 to 267590.08)	14572.76<br>(12725.39 to 16500.23)	0.27<br>(0.13 to 0.41)	-0.4<br>(-0.55 to -0.25)	0.916
Saudi Arabia	206699.77<br>(176177.41 to 239705.48)	6865.5<br>(5851.7 to 7961.78)	0.6<br>(0.56 to 0.64)	0.75<br>(-0.71 to 2.22)	0.815
Spain	2239541.11<br>(2000744.67 to 2510844.81)	14471.92<br>(12928.82 to 16225.09)	0.39<br>(0.27 to 0.52)	-0.82<br>(-1.82 to 0.19)	0.769
Sweden	587049.44<br>(514171.41 to 658256.36)	17438.12<br>(15273.3 to 19553.3)	0.09<br>(0.03 to 0.15)	0.06<br>(-0.06 to 0.17)	0.887
Thailand	1398721.91<br>(1177380.88 to 1619546.17)	7195.82<br>(6057.11 to 8331.86)	-0.3<br>(-0.34 to -0.27)	-0.61<br>(-3.18 to 2.03)	0.683
Netherlands	662332.13<br>(591079.45 to 743292.63)	11320.23<br>(10102.41 to 12703.96)	0.32<br>(0.19 to 0.45)	-3.15<br>(-3.54 to -2.76)	0.888
Tonga	1102.44<br>(925.16 to 1292.63)	8208.48<br>(6888.51 to 9624.55)	-0.3<br>(-0.34 to -0.26)	-0.04<br>(-0.5 to 0.43)	0.626
Kyrgyzstan	84966.74<br>(73309 to 96986.55)	10046.31<br>(8667.92 to 11467.51)	-0.72<br>(-0.9 to -0.55)	-0.59<br>(-1.24 to 0.06)	0.604
Laos	69866.76<br>(60590.85 to 80118.92)	9036.65<br>(7836.89 to 10362.68)	-0.19<br>(-0.22 to -0.16)	0.78<br>(0.11 to 1.47)	0.489
Lebanon	116821.48<br>(98552.07 to 135611)	11920.21<br>(10056.04 to 13837.45)	2<br>(1.92 to 2.08)	-1.06<br>(-2.75 to 0.66)	0.745
Malaysia	350442.88<br>(293060.31 to 405205.22)	7147.83<br>(5977.42 to 8264.79)	-0.1<br>(-0.18 to -0.02)	1.61<br>(1.2 to 2.03)	0.743
Mongolia	30917.14<br>(26593.66 to 35838.1)	7830.97<br>(6735.88 to 9077.39)	-0.33<br>(-0.38 to -0.28)	-0.15<br>(-1.24 to 0.94)	0.618
Montenegro	15783.86<br>(13345.13 to 18118.92)	9051.77<br>(7653.2 to 10390.89)	0.77<br>(0.67 to 0.87)	-0.42<br>(-0.9 to 0.06)	0.796
New Zealand	125359.36<br>(104447.26 to 148423.06)	8788.37<br>(7322.32 to 10405.26)	-0.49<br>(-0.6 to -0.38)	-1.72<br>(-4 to 0.62)	0.849
North Macedonia	66776.19<br>(57285.25 to 78501.34)	11262.53<br>(9661.78 to 13240.1)	0.48<br>(0.39 to 0.57)	0.27<br>(-0.23 to 0.77)	0.751
Northern Mariana Islands	615.2<br>(514.1 to 719.57)	6329.03<br>(5288.94 to 7402.71)	-0.56<br>(-0.72 to -0.4)	1.31<br>(0.87 to 1.75)	0.772
Palestine	34265.16<br>(29723.19 to 39549.14)	7963.82<br>(6908.18 to 9191.91)	0.17<br>(0.15 to 0.19)	0.56<br>(-0.57 to 1.71)	0.631
Algeria	638934.7<br>(549545.14 to 727015.11)	10520.27<br>(9048.44 to 11970.54)	0.88<br>(0.79 to 0.98)	0.64<br>(0.62 to 0.65)	0.660
Bangladesh	2948575.31<br>(2622094.86 to 3318716.39)	12619.01<br>(11221.77 to 14203.1)	0.29<br>(0.23 to 0.34)	0.38<br>(0.16 to 0.6)	0.492
China	41391128.46<br>(36108228.63 to 46866337.15)	10922.44<br>(9528.37 to 12367.25)	-0.07<br>(-0.13 to 0)	-0.4<br>(-0.58 to -0.23)	0.722
Bolivia	128104.6<br>(110071.48 to 148880.01)	8289.03<br>(7122.19 to 9633.31)	0.28<br>(0.26 to 0.3)	-0.52<br>(-0.53 to -0.51)	0.599
Portugal	492248.58<br>(435699.91 to 556996.86)	12652.11<br>(11198.66 to 14316.32)	0.18<br>(0.12 to 0.24)	0.31<br>(-0.14 to 0.77)	0.744
Andorra	3315.38<br>(2902.78 to 3734.66)	12528.93<br>(10969.71 to 14113.43)	-0.01<br>(-0.15 to 0.13)	-0.74<br>(-0.78 to -0.71)	0.869
Monaco	1991.54<br>(1706.43 to 2271.95)	12729.26<br>(10906.96 to 14521.55)	-0.19<br>(-0.27 to -0.12)	-0.51<br>(-0.64 to -0.37)	0.908
Puerto Rico	114100.57<br>(98266.59 to 128555.69)	9774.2<br>(8417.82 to 11012.47)	0.94<br>(0.88 to 1)	1.05<br>(-0.28 to 2.4)	0.826
Albania	87447.98<br>(75185.01 to 101035.23)	11121.54<br>(9561.95 to 12849.55)	0.6<br>(0.48 to 0.71)	0.37<br>(0.28 to 0.45)	0.707
Angola	115900.89<br>(98035.22 to 134818.08)	5993.95<br>(5070.01 to 6972.28)	-0.28<br>(-0.33 to -0.23)	1.23<br>(0.59 to 1.87)	0.454
Armenia	84605.55<br>(74056.66 to 96255.99)	10755.6<br>(9414.55 to 12236.67)	0.63<br>(0.49 to 0.76)	-0.41<br>(-1.97 to 1.16)	0.702
Austria	441343.4<br>(394290.77 to 484974.26)	14934.6<br>(13342.39 to 16411.03)	0.26<br>(0.15 to 0.38)	-0.97<br>(-2.06 to 0.13)	0.854

Azerbaijan	155117.98<br>(131284.26 to 178969.47)	8096.78<br>(6852.72 to 9341.77)	-0.05<br>(-0.22 to 0.13)	-0.56<br>(-0.76 to -0.37)	0.695
Belarus	258448.79<br>(224663.6 to 295576.74)	8984.51<br>(7810.03 to 10275.2)	-0.52<br>(-0.56 to -0.48)	0.17<br>(-0.09 to 0.42)	0.784
Benin	51866.6<br>(44461.48 to 60741.38)	6271.19<br>(5375.83 to 7344.24)	0.1<br>(0.05 to 0.16)	0.64<br>(0.62 to 0.66)	0.373
Botswana	18670.33<br>(15975.72 to 21569.22)	7701.54<br>(6590.01 to 8897.33)	0.05<br>(0 to 0.11)	0.71<br>(0.67 to 0.74)	0.643
Bulgaria	289147.53<br>(246977.31 to 331452.72)	12164.86<br>(10390.7 to 13944.7)	0.47<br>(0.44 to 0.51)	0.94<br>(-0.52 to 2.42)	0.768
Burundi	54516.59<br>(46599.85 to 63120.91)	6810.08<br>(5821.14 to 7884.91)	-0.03<br>(-0.11 to 0.06)	1.11<br>(1.02 to 1.2)	0.289
Cabo Verde	2875.2<br>(2438.18 to 3322.71)	3751.27<br>(3181.08 to 4335.13)	0.21<br>(0.15 to 0.27)	-0.19<br>(-0.37 to -0.01)	0.534
Cameroon	113244.3<br>(96580.2 to 133406.66)	5563.52<br>(4744.84 to 6554.07)	0.14<br>(0.08 to 0.21)	1.17<br>(0.55 to 1.79)	0.480
Chad	62670.63<br>(53260.47 to 73216.76)	6749.5<br>(5736.05 to 7885.3)	0.19<br>(0.12 to 0.26)	0.77<br>(0.24 to 1.3)	0.240
Chile	264824.86<br>(225617.23 to 304785.98)	5914.33<br>(5038.71 to 6806.78)	-0.32<br>(-0.38 to -0.26)	-0.49<br>(-1.8 to 0.85)	0.772
Colombia	1073325.85<br>(949403.82 to 1197883.75)	11223.15<br>(9927.36 to 12525.57)	0.36<br>(0.27 to 0.44)	0.43<br>(0.04 to 0.83)	0.655
Costa Rica	96085.39<br>(82056.29 to 109232.42)	10035.42<br>(8570.18 to 11408.53)	0.32<br>(0.25 to 0.38)	0.95<br>(0.5 to 1.41)	0.700
Côte d'Ivoire	106264.58<br>(90915.13 to 125442.71)	5926.03<br>(5070.04 to 6995.53)	0.47<br>(0.41 to 0.54)	0.98<br>(0.49 to 1.47)	0.426
Croatia	199089.87<br>(177280 to 220343.72)	13349.85<br>(11887.41 to 14775.02)	1.36<br>(1.28 to 1.44)	0.49<br>(0.11 to 0.87)	0.798
Cuba	342861.86<br>(302723.06 to 385264.86)	10033.86<br>(8859.2 to 11274.79)	1.05<br>(0.93 to 1.18)	-1.92<br>(-2.72 to -1.11)	0.669
Cyprus	41310.07<br>(35994.48 to 46743.56)	11819.71<br>(10298.8 to 13374.35)	-0.03<br>(-0.06 to 0)	0.56<br>(0.55 to 0.56)	0.836
Djibouti	5099.47<br>(4297.31 to 5941.12)	4917.34<br>(4143.83 to 5728.93)	0.21<br>(0.04 to 0.38)	1.42<br>(1.12 to 1.71)	0.488
Ecuador	219277.89<br>(187389.84 to 254463.76)	7923.26<br>(6771.04 to 9194.65)	0.49<br>(0.46 to 0.53)	-0.23<br>(-0.34 to -0.12)	0.661
El Salvador	97579.64<br>(82897.83 to 113014.54)	9542.46<br>(8106.71 to 11051.87)	0.86<br>(0.82 to 0.91)	-0.02<br>(-1.05 to 1.02)	0.564
Equatorial Guinea	5221.11<br>(4428.85 to 6059.19)	6600.91<br>(5599.27 to 7660.46)	0.02<br>(-0.12 to 0.16)	0.51<br>(0.24 to 0.77)	0.658
Estonia	34454.8<br>(30186.99 to 39619.22)	7875.83<br>(6900.27 to 9056.33)	1.51<br>(1.4 to 1.62)	-2.62<br>(-5.08 to -0.1)	0.845
Fiji	7929.32<br>(6762.39 to 9300.28)	5798.8<br>(4945.41 to 6801.41)	-0.87<br>(-0.95 to -0.79)	0.04<br>(-0.76 to 0.86)	0.675
Finland	229167.13<br>(197945.98 to 263056.64)	11386.24<br>(9835.01 to 13070.05)	0.29<br>(0.2 to 0.38)	1.28<br>(0.25 to 2.33)	0.860
Ghana	163177.18<br>(137960.73 to 188492.01)	5983.39<br>(5058.75 to 6911.63)	0.73<br>(0.66 to 0.81)	0.91<br>(0.91 to 0.92)	0.565
Guatemala	151318.08<br>(130594.68 to 174882.58)	8265.26<br>(7133.31 to 9552.39)	0.66<br>(0.61 to 0.71)	0.68<br>(0.64 to 0.71)	0.540
Guinea	63110.23<br>(53760.24 to 72365.6)	6908.18<br>(5884.71 to 7921.29)	0.49<br>(0.47 to 0.52)	0.81<br>(0.66 to 0.97)	0.336
Guinea-Bissau	6981.2<br>(5864.55 to 8310.81)	6077.46<br>(5105.36 to 7234.95)	0.14<br>(0.03 to 0.25)	0.58<br>(-0.15 to 1.31)	0.353
Guyana	6148.25<br>(5189.14 to 7182.49)	5459.41<br>(4607.76 to 6377.78)	0.4<br>(0.33 to 0.46)	0.27<br>(-0.66 to 1.2)	0.651
Haiti	86092.85<br>(73786.62 to 100350.38)	7264.68<br>(6226.25 to 8467.76)	0.14<br>(0.04 to 0.23)	-0.33<br>(-0.36 to -0.31)	0.448
Honduras	111278.53<br>(97000.4 to 128563.73)	10581.13<br>(9223.47 to 12224.73)	0.6<br>(0.53 to 0.66)	-0.08<br>(-0.33 to 0.16)	0.513
Iceland	13383.84<br>(11836.49 to 14818.51)	13743.06<br>(12154.17 to 15216.23)	-0.51<br>(-0.68 to -0.34)	0.45<br>(0.22 to 0.67)	0.876
India	25754244.13<br>(23105088.44 to 28450001.88)	12810.17<br>(11492.47 to 14151.04)	0.49<br>(0.43 to 0.55)	-0.14<br>(-0.27 to 0)	0.575
Indonesia	3333818.54<br>(2828709.47 to 3839088.05)	7960.49<br>(6754.39 to 9166.98)	0.29<br>(0.21 to 0.38)	0.53<br>(0.36 to 0.7)	0.657

Iraq	289090.46 (247018.06 to 338731.36)	7440.83 (6357.94 to 8718.52)	0.41 (0.33 to 0.49)	0.32 (-1.02 to 1.67)	0.663
Italy	2865320.38 (2456953.51 to 3272520.89)	12613.23 (10815.58 to 14405.73)	0.59 (0.52 to 0.67)	-1.02 (-1.85 to -0.18)	0.806
Kazakhstan	344480.75 (305494.14 to 392062.08)	10855.1 (9626.58 to 12354.46)	0.51 (0.41 to 0.61)	-0.45 (-0.45 to -0.45)	0.725
Kenya	209708.39 (176708.68 to 243787.28)	5606.02 (4723.86 to 6517.03)	0.06 (-0.01 to 0.13)	0.98 (0.53 to 1.44)	0.524
Kiribati	965.73 (818.35 to 1124.29)	7678.65 (6506.8 to 8939.4)	-0.38 (-0.44 to -0.32)	0.02 (-0.5 to 0.54)	0.527
South Korea	1790479.87 (1619666.51 to 1978860.89)	10706.21 (9684.82 to 11832.63)	1.01 (0.73 to 1.29)	-1.3 (-2.14 to -0.46)	0.887
Latvia	58603.51 (50706.89 to 66778.39)	8906.04 (7705.99 to 10148.39)	0.62 (0.47 to 0.76)	1.12 (1.09 to 1.15)	0.831
Liberia	20487.77 (17257.03 to 23877.86)	6206.13 (5227.48 to 7233.05)	0.41 (0.3 to 0.51)	0.19 (-0.26 to 0.63)	0.352
Lithuania	88922.06 (77590.9 to 101573.7)	9188.25 (8017.41 to 10495.53)	0.04 (-0.03 to 0.11)	0.9 (0.59 to 1.21)	0.856
Madagascar	116819.38 (100462.95 to 136921.42)	6325.91 (5440.19 to 7414.46)	0.13 (0.06 to 0.2)	0.6 (-0.15 to 1.36)	0.400
Malawi	66089.05 (56618.87 to 77018.32)	5652.85 (4842.83 to 6587.67)	0.55 (0.49 to 0.6)	1.09 (1.03 to 1.14)	0.385
Maldives	4914.39 (4265.27 to 5645.25)	9034.41 (7841.09 to 10377.99)	0.23 (0.12 to 0.33)	0.07 (-0.71 to 0.86)	0.651
Mali	96301.75 (82253.28 to 112283.54)	6653.56 (5682.94 to 7757.75)	0.49 (0.42 to 0.56)	0.92 (0.87 to 0.96)	0.269
Malta	17298.87 (14759.32 to 19738.17)	10962.31 (9352.99 to 12508.09)	-0.09 (-0.19 to 0.02)	1.46 (1.37 to 1.55)	0.802
Mauritius	22561.61 (19599.2 to 25825.46)	6668.48 (5792.89 to 7633.17)	-0.06 (-0.17 to 0.05)	1.19 (1.11 to 1.27)	0.718
Moldova	95588.74 (81891.37 to 111359.87)	9053.39 (7756.09 to 10547.11)	-0.43 (-0.51 to -0.36)	0.74 (0.74 to 0.75)	0.732
Mozambique	100755.73 (85189.87 to 117596.48)	5631.54 (4761.51 to 6572.81)	0.21 (0.14 to 0.28)	1.18 (0.91 to 1.45)	0.326
Namibia	18042.2 (15505.6 to 20869.04)	7985.15 (6862.5 to 9236.26)	0.14 (0.09 to 0.19)	0.06 (-0.25 to 0.36)	0.618
Nauru	69.68 (58.86 to 82.2)	7538.45 (6367.83 to 8893.61)	-0.38 (-0.44 to -0.32)	0.53 (0.11 to 0.95)	0.625
Nicaragua	80824.8 (70672.23 to 91495.97)	9928.93 (8681.73 to 11239.83)	0.75 (0.64 to 0.86)	-0.15 (-1.6 to 1.33)	0.524
Niue	30.17 (25.33 to 35.63)	7711.17 (6473.04 to 9106.7)	-0.77 (-0.83 to -0.7)	-0.05 (-0.22 to 0.12)	0.726
Palau	275.99 (233.9 to 319.93)	6591.09 (5585.86 to 7640.38)	-1.11 (-1.2 to -1.01)	0.28 (-0.45 to 1.01)	0.754
Panama	67816.24 (59790.74 to 76001.09)	9120.42 (8041.1 to 10221.18)	0.56 (0.47 to 0.64)	1.43 (1.17 to 1.7)	0.709
Paraguay	92484.66 (79624.08 to 107172.28)	9340.89 (8041.98 to 10824.33)	0.3 (0.27 to 0.32)	0.6 (-0.37 to 1.57)	0.636
Peru	398747.58 (336949.99 to 463641.68)	7128.84 (6024.02 to 8289.02)	0.89 (0.73 to 1.05)	-3.82 (-7.42 to -0.08)	0.662
Poland	1345775.92 (1162153.28 to 1528454.06)	11108.02 (9592.4 to 12615.84)	0.24 (0.17 to 0.32)	1.36 (1.09 to 1.63)	0.812
Rwanda	70779 (60614.19 to 81011.6)	6693.26 (5732.02 to 7660.92)	0.05 (-0.05 to 0.16)	1.35 (1.22 to 1.48)	0.436
San Marino	1410.8 (1202.97 to 1619.32)	11928.68 (10171.4 to 13691.75)	0.1 (0 to 0.2)	-0.77 (-1.66 to 0.13)	0.888
Senegal	80099.24 (68244.05 to 93495.75)	6203.64 (5285.46 to 7241.19)	0.39 (0.33 to 0.46)	0.62 (0.51 to 0.72)	0.408
Serbia	372635.06 (329561.25 to 417632.2)	13283.63 (11748.14 to 14887.68)	1.23 (1.16 to 1.31)	0.46 (0.3 to 0.62)	0.792
Seychelles	1480.97 (1269.65 to 1710.02)	7139.62 (6120.89 to 8243.86)	-0.02 (-0.15 to 0.12)	0.55 (-0.07 to 1.17)	0.730
Sierra Leone	39528.42 (33820.85 to 45553.53)	6543.53 (5598.7 to 7540.93)	0.19 (0.14 to 0.24)	1.18 (0.54 to 1.82)	0.359
Singapore	66004.51 (56671.09 to 77205.58)	4345.75 (3731.24 to 5083.24)	-1.6 (-1.75 to -1.45)	0.54 (0.01 to 1.06)	0.856

Slovenia	84481.48<br>(73388.92 to 96044)	11538.21<br>(10023.22 to 13117.38)	0.61<br>(0.58 to 0.64)	-0.2<br>(-1.3 to 0.91)	0.842
South Africa	684192.4<br>(589872.9 to 782358.58)	8673.23<br>(7477.58 to 9917.64)	-0.12<br>(-0.18 to -0.06)	-0.02<br>(-0.24 to 0.2)	0.680
South Sudan	35534.07<br>(30225.96 to 41248.45)	5706.25<br>(4853.84 to 6623.89)	-0.09<br>(-0.16 to -0.03)	0.47<br>(0.35 to 0.6)	0.278
Sudan	290911.78<br>(247655.52 to 335358.84)	9342.56<br>(7953.39 to 10769.96)	-0.03<br>(-0.09 to 0.04)	-0.24<br>(-1.1 to 0.62)	0.542
Suriname	7341.56<br>(6308.18 to 8597.94)	6572.41<br>(5647.29 to 7697.17)	0.49<br>(0.39 to 0.58)	0.21<br>(-0.08 to 0.5)	0.634
Tajikistan	91676.47<br>(79394.82 to 105335.09)	8925.37<br>(7729.66 to 10255.13)	-0.35<br>(-0.52 to -0.19)	-0.45<br>(-0.71 to -0.19)	0.542
Republic of the Congo	27318.74<br>(23218.76 to 31973.02)	6286.99<br>(5343.44 to 7358.1)	0.08<br>(0.05 to 0.11)	0.63<br>(0.13 to 1.12)	0.583
Gambia	10375.17<br>(8786.12 to 12119.35)	6709.23<br>(5681.65 to 7837.12)	0.56<br>(0.5 to 0.61)	0.5<br>(0.37 to 0.64)	0.410
Marshall Islands	384.27<br>(329.28 to 447.09)	6558.56<br>(5620.03 to 7630.82)	-1.17<br>(-1.29 to -1.04)	1<br>(0.68 to 1.32)	0.574
Niger	96651.37<br>(82293.22 to 113500.28)	6942.27<br>(5910.96 to 8152.5)	0.69<br>(0.59 to 0.79)	1.02<br>(0.75 to 1.29)	0.168
Philippines	1170329.02<br>(1002862.77 to 1333531.54)	8392.42<br>(7191.52 to 9562.74)	-0.56<br>(-0.63 to -0.48)	0.58<br>(0.22 to 0.93)	0.651
Myanmar	905054.4<br>(803823.64 to 1026547.15)	10720.7<br>(9521.58 to 12159.82)	-0.02<br>(-0.09 to 0.06)	0.06<br>(0.04 to 0.07)	0.534
Trinidad and Tobago	21068.11<br>(17837.5 to 24505.02)	6048.17<br>(5120.73 to 7034.82)	0.24<br>(0.19 to 0.29)	1.33<br>(1.28 to 1.38)	0.769
Tunisia	271230.76<br>(235709.05 to 307569.68)	11605.27<br>(10085.39 to 13160.12)	0.95<br>(0.86 to 1.04)	0.29<br>(-0.6 to 1.19)	0.682
Turkey	2187279.42<br>(1957151.14 to 2444553.68)	13239.18<br>(11846.26 to 14796.42)	0.65<br>(0.52 to 0.78)	-0.2<br>(-0.61 to 0.2)	0.713
Uganda	145616.04<br>(123433.82 to 168537.22)	6140.27<br>(5204.9 to 7106.8)	-0.08<br>(-0.14 to -0.03)	0.7<br>(0.42 to 0.97)	0.423
Uzbekistan	317495.57<br>(271181.44 to 364822.12)	6829.84<br>(5833.55 to 7847.91)	-0.87<br>(-0.99 to -0.76)	-0.35<br>(-0.81 to 0.12)	0.663
Vanuatu	2358.72<br>(2018.19 to 2730.38)	8225.77<br>(7038.22 to 9521.92)	-0.51<br>(-0.55 to -0.48)	0.31<br>(0.26 to 0.36)	0.473
Yemen	212647.18<br>(180694.86 to 246083.65)	9455.5<br>(8034.72 to 10942.28)	0.07<br>(0.01 to 0.14)	0.35<br>(-0.31 to 1.02)	0.450
Zambia	60859.12<br>(51527.92 to 70804.26)	5634.28<br>(4770.4 to 6554.99)	0.26<br>(0.21 to 0.31)	1.05<br>(0.45 to 1.66)	0.506
Zimbabwe	73155.22<br>(61391.63 to 85926.01)	6606.8<br>(5544.41 to 7760.16)	-0.03<br>(-0.13 to 0.07)	0.99<br>(0.02 to 1.96)	0.474
Romania	723062.17<br>(634510.59 to 816456.63)	12040.3<br>(10565.76 to 13595.49)	0.38<br>(0.34 to 0.42)	0.61<br>(-0.7 to 1.94)	0.768
Russia	4031571.79<br>(3439770.88 to 4619650.15)	9484.89<br>(8092.58 to 10868.43)	-0.17<br>(-0.27 to -0.07)	1.95<br>(1.18 to 2.72)	0.809
Saint Kitts and Nevis	718.07<br>(602.87 to 842.31)	5575.34<br>(4680.93 to 6540.01)	-0.11<br>(-0.36 to 0.14)	-0.85<br>(-1.72 to 0.03)	0.755
Saint Lucia	3190.82<br>(2734.93 to 3625.31)	7582.4<br>(6499.07 to 8614.89)	0.46<br>(0.37 to 0.55)	-0.3<br>(-1.11 to 0.51)	0.673
Saint Vincent and the Grenadines	1414.54<br>(1211.78 to 1633.42)	5563.44<br>(4765.97 to 6424.31)	0.53<br>(0.47 to 0.6)	-0.52<br>(-1.72 to 0.7)	0.637
Slovakia	162828.78<br>(142105.37 to 185678.21)	9926.91<br>(8663.5 to 11319.93)	0.49<br>(0.45 to 0.52)	0.19<br>(-0.82 to 1.2)	0.811
Vietnam	1578849.28<br>(1371250.78 to 1811558.18)	9038.22<br>(7849.81 to 10370.37)	0.24<br>(0.09 to 0.38)	0.71<br>(0.4 to 1.02)	0.628
Solomon Islands	4898.18<br>(4304.75 to 5622.51)	9029.61<br>(7935.65 to 10364.9)	-0.07<br>(-0.11 to -0.02)	-0.2<br>(-0.58 to 0.19)	0.429
Eritrea	23798.12<br>(20127.42 to 28085.44)	5401.19<br>(4568.09 to 6374.23)	0.14<br>(0.08 to 0.21)	1.15<br>(0.46 to 1.86)	0.404
Israel	232063.63<br>(197742.35 to 261603.85)	11611.63<br>(9894.32 to 13089.72)	-0.21<br>(-0.27 to -0.15)	0.13<br>(-0.32 to 0.59)	0.809
Kuwait	34156.02<br>(29308.35 to 39352.77)	7326.1<br>(6286.33 to 8440.75)	0.76<br>(0.58 to 0.94)	-0.76<br>(-1.38 to -0.14)	0.847
Libya	71876.48<br>(62218.2 to 82818.27)	8594.41<br>(7439.55 to 9902.74)	0.85<br>(0.75 to 0.94)	-1.39<br>(-2.42 to -0.34)	0.726

Qatar	10438.54 (8984.14 to 12180.12)	6814.37 (5864.93 to 7951.3)	-0.19 (-0.3 to -0.07)	1.24 (-0.47 to 2.99)	0.847
Oman	26431.81 (22967.25 to 30775.84)	8462.12 (7352.94 to 9852.86)	0.1 (0.07 to 0.14)	-0.21 (-1.13 to 0.73)	0.773
Switzerland	392372.94 (348034.26 to 439857.71)	13387.75 (11874.92 to 15007.93)	-0.17 (-0.27 to -0.07)	-0.94 (-1.07 to -0.81)	0.933
Syria	212811 (185501.11 to 244859.69)	9007.1 (7851.22 to 10363.54)	0.72 (0.67 to 0.78)	0.33 (-0.33 to 1)	0.623
Taiwan	529659.43 (461093.83 to 621988.07)	7034.05 (6123.48 to 8260.2)	-0.47 (-0.65 to -0.28)	-0.1 (-1.06 to 0.87)	0.875
Togo	37515.97 (31488.42 to 43587.79)	6005.56 (5040.67 to 6977.53)	0.24 (0.16 to 0.33)	0.97 (0.89 to 1.05)	0.409
Tokelau	20.78 (17.44 to 24.24)	8223.28 (6898.91 to 9591.19)	-0.26 (-0.36 to -0.17)	-0.13 (-0.32 to 0.07)	0.686
Turkmenistan	43444.12 (37184.64 to 50203.65)	6138.77 (5254.29 to 7093.91)	-1.05 (-1.13 to -0.97)	0.15 (-1.12 to 1.42)	0.682
Tuvalu	141.21 (119.64 to 165.77)	7748.9 (6565.3 to 9096.47)	-0.61 (-0.7 to -0.51)	0.84 (0.22 to 1.47)	0.577
Ukraine	1200842.59 (1014169.08 to 1397580.91)	8843.99 (7469.17 to 10292.93)	-0.89 (-0.95 to -0.83)	0.7 (0.59 to 0.81)	0.761
Comoros	4580.79 (3864.37 to 5324.52)	5667.15 (4780.83 to 6587.26)	0.12 (0.05 to 0.19)	0.8 (0.21 to 1.4)	0.476
UAE	54014.32 (46687.57 to 62074.15)	7522.06 (6501.73 to 8644.48)	-0.5 (-0.59 to -0.4)	0.71 (-0.31 to 1.73)	0.849
UK	3648421.19 (3254319.47 to 4010988.71)	17348.5 (15474.52 to 19072.53)	0.32 (0.27 to 0.38)	-1.01 (-1.77 to -0.25)	0.859
Mexico	2126432.02 (1825950.6 to 2428163.9)	9867.23 (8472.91 to 11267.35)	0.52 (0.41 to 0.62)	-1.17 (-1.98 to -0.34)	0.665
Tanzania	235784.89 (199979.85 to 276891.89)	5719.91 (4851.31 to 6717.12)	0.41 (0.34 to 0.47)	0.99 (0.92 to 1.06)	0.447
USA	17832187.07 (16837655.82 to 18705001.72)	17788.12 (16796.04 to 18658.77)	0.24 (0.11 to 0.37)	0.31 (0.14 to 0.49)	0.862
US Virgin Islands	2247.3 (1903.19 to 2597.38)	6981.52 (5912.51 to 8069.09)	1.22 (1.11 to 1.32)	1.47 (1.34 to 1.59)	0.822

**Cause**	**Asthma**				
**Location**	**Prevalence number**	**Prevalence rate**	**EAPC 1990-2021(95% CI)**	**EAPC 2019-2021(95% CI)**	**2021SDI**
American Samoa	123.36 (111.73 to 136.26)	1460.5 (1322.9 to 1613.24)	-3 (-3.24 to -2.75)	-0.59 (-1.31 to 0.14)	0.724
Antigua and Barbuda	520.31 (479.25 to 566.84)	2757.68 (2540.07 to 3004.29)	-1.09 (-1.15 to -1.04)	0.49 (-0.01 to 1)	0.750
Egypt	554916.95 (446017.46 to 676098.13)	5014.68 (4030.58 to 6109.77)	-1.08 (-1.14 to -1.03)	2.43 (0.18 to 4.73)	0.607
Argentina	807213.38 (710680.94 to 902911.45)	8648.74 (7614.46 to 9674.08)	-1.78 (-2 to -1.56)	1.05 (0.71 to 1.39)	0.723
Australia	475133.79 (430398.9 to 522162.09)	6413.94 (5810.06 to 7048.79)	-2.35 (-2.55 to -2.14)	0.22 (0.07 to 0.37)	0.844
Barbados	5655.92 (4945.46 to 6507.15)	6207.19 (5427.48 to 7141.39)	-1.1 (-1.15 to -1.05)	0.13 (-0.2 to 0.45)	0.747
Belize	1275.29 (1162.07 to 1383.31)	2546.18 (2320.13 to 2761.85)	-1.15 (-1.32 to -0.97)	0.33 (-0.46 to 1.14)	0.610
Bermuda	465.89 (418.59 to 517.93)	2000.32 (1797.23 to 2223.74)	-2.84 (-2.97 to -2.72)	0.1 (-0.47 to 0.67)	0.821
Venezuela	105764.4 (91769.86 to 122967.29)	2021.42 (1753.95 to 2350.21)	-1.77 (-1.92 to -1.62)	-0.24 (-0.37 to -0.1)	0.597
Bosnia and Herzegovina	66439.11 (56972.52 to 77503.92)	6066.01 (5201.69 to 7076.25)	-1.25 (-1.49 to -1)	0.27 (-0.39 to 0.92)	0.723
Brunei	6098.92 (5532.74 to 6701.38)	10186.16 (9240.55 to 11192.36)	-1.18 (-1.38 to -0.98)	1.62 (1.44 to 1.79)	0.810
Burkina Faso	52235.77 (46516.05 to 59754.6)	3474.48 (3094.03 to 3974.59)	-0.85 (-0.92 to -0.77)	1.26 (1 to 1.52)	0.285

Canada	616645.68 (530510.68 to 709038.83)	5025.15 (4323.22 to 5778.08)	-2.45 (-2.77 to -2.12)	0.88 (-0.13 to 1.91)	0.873
Central African Republic	18148.22 (15868.74 to 20737.28)	5073.61 (4436.35 to 5797.42)	-1.01 (-1.07 to -0.94)	-0.57 (-1.43 to 0.3)	0.309
Dominica	644.58 (586.79 to 714.9)	4273.62 (3890.43 to 4739.81)	-0.94 (-0.99 to -0.89)	0.42 (-0.34 to 1.18)	0.747
Bahamas	1619.01 (1469.91 to 1774.45)	2249.92 (2042.71 to 2465.93)	-1.83 (-1.92 to -1.75)	0.09 (-0.7 to 0.9)	0.805
Cook Islands	61.24 (56.05 to 66.84)	1299.42 (1189.31 to 1418.27)	-2.88 (-3.68 to -2.08)	0.18 (-0.03 to 0.39)	0.779
Czech Republic	73305.92 (63432.09 to 85399.55)	2098.37 (1815.74 to 2444.55)	-1.8 (-1.94 to -1.66)	-0.78 (-0.82 to -0.74)	0.828
North Korea	378384.06 (338228.42 to 422351.15)	6713.97 (6001.46 to 7494.12)	-0.79 (-0.87 to -0.71)	0.09 (-0.1 to 0.28)	0.570
Sao Tome and Principe	1236.32 (1122.81 to 1376.47)	6761.83 (6141 to 7528.36)	-0.59 (-0.69 to -0.49)	1.1 (0.5 to 1.71)	0.505
DR Congo	216957.19 (182649.76 to 257634.4)	3592.12 (3024.1 to 4265.6)	-0.58 (-0.68 to -0.49)	0.6 (0.17 to 1.02)	0.383
Timor-Leste	6265.64 (5565.91 to 7032.81)	4411.46 (3918.8 to 4951.6)	-1.11 (-1.23 to -0.99)	0.14 (-0.01 to 0.29)	0.445
Sri Lanka	417738.83 (366246.19 to 478596.89)	8673.43 (7604.3 to 9937.02)	-0.87 (-0.96 to -0.79)	1.73 (1.11 to 2.34)	0.702
Dominican Republic	34723.07 (31070.07 to 39043.31)	2077.95 (1859.34 to 2336.49)	-0.89 (-1.03 to -0.76)	0.97 (0.89 to 1.05)	0.619
Uruguay	70242.31 (64261.85 to 76824.58)	7767.66 (7106.31 to 8495.55)	-1.82 (-1.94 to -1.69)	0.92 (0.66 to 1.18)	0.719
Ethiopia	156772.54 (133365.14 to 182188.81)	2290.13 (1948.19 to 2661.41)	-2.44 (-2.6 to -2.29)	0.12 (-0.54 to 0.79)	0.359
Nepal	153557.22 (135303.05 to 172029.7)	3879.81 (3418.59 to 4346.54)	-1.11 (-1.25 to -0.97)	0.44 (0.07 to 0.82)	0.433
Germany	1242148.53 (1105842.12 to 1389787.57)	3941.59 (3509.06 to 4410.08)	-3.79 (-4.2 to -3.38)	0.14 (-0.15 to 0.43)	0.903
Nigeria	1047262.48 (890032.7 to 1222467.32)	6981.05 (5932.95 to 8148.96)	-0.85 (-0.98 to -0.72)	1.95 (1.35 to 2.56)	0.503
Somalia	57874.41 (50043.04 to 66754.4)	6136.87 (5306.45 to 7078.48)	-0.74 (-0.79 to -0.69)	-0.73 (-0.86 to -0.6)	0.078
Micronesia	391.54 (350.12 to 442.35)	2975.45 (2660.66 to 3361.54)	-2.71 (-2.78 to -2.64)	0.03 (-0.71 to 0.78)	0.588
Brazil	977634.94 (850578.15 to 1123307.15)	2257.41 (1964.03 to 2593.78)	-2.94 (-3.22 to -2.65)	1.06 (0.98 to 1.13)	0.653
France	1423279.58 (1282543.09 to 1578011.25)	6437.23 (5800.71 to 7137.05)	-3.49 (-3.63 to -3.35)	0.03 (-0.01 to 0.07)	0.838
Gabon	5351.74 (4419.06 to 6538.83)	2993.95 (2472.17 to 3658.05)	-1.68 (-1.77 to -1.59)	-0.28 (-1.4 to 0.85)	0.635
Georgia	15413.39 (13642.49 to 17572.32)	1468.13 (1299.45 to 1673.76)	-3.71 (-4.02 to -3.4)	0.19 (-0.19 to 0.58)	0.732
Luxembourg	14138.94 (12456.23 to 15858.95)	7956.55 (7009.62 to 8924.46)	-2.3 (-2.39 to -2.2)	0.36 (-0.33 to 1.06)	0.884
Greenland	1420.08 (1283.25 to 1567.03)	10460.22 (9452.32 to 11542.62)	-3.24 (-3.47 to -3.01)	1.06 (1.06 to 1.06)	0.826
Grenada	888.08 (728.73 to 1054.47)	4421.14 (3627.85 to 5249.46)	-1.36 (-1.42 to -1.29)	0.4 (-3.13 to 4.07)	0.669
Guam	318.41 (289.27 to 353.03)	844.91 (767.59 to 936.77)	-4.22 (-5.03 to -3.4)	-0.38 (-0.71 to -0.05)	0.804
Jordan	34652.18 (29088.05 to 41065.22)	2756.21 (2313.64 to 3266.29)	-2.43 (-2.58 to -2.28)	-1.27 (-4.53 to 2.1)	0.725
Greece	173756.91 (150872.43 to 199458.13)	4727.5 (4104.87 to 5426.77)	-2.99 (-3.14 to -2.83)	2.62 (-0.2 to 5.52)	0.792
Hungary	85181.21 (74538.75 to 97547.45)	2678.55 (2343.89 to 3067.41)	-2.93 (-3.12 to -2.74)	-0.15 (-0.36 to 0.06)	0.791

Papua New Guinea	42682.71 (38612.17 to 48185.47)	5196.95 (4701.33 to 5866.95)	-1.76 (-1.86 to -1.66)	-0.49 (-0.94 to -0.04)	0.418
Samoa	834.68 (658.82 to 1064.35)	3390.06 (2675.81 to 4322.86)	-1.43 (-1.5 to -1.36)	-1.94 (-3.26 to -0.61)	0.593
Ireland	78721.49 (69784.3 to 89153.76)	5998.43 (5317.43 to 6793.35)	-3.41 (-3.6 to -3.23)	-0.02 (-0.64 to 0.61)	0.874
Afghanistan	108944.53 (97776.56 to 121151.69)	8866.28 (7957.39 to 9859.74)	-1.32 (-1.38 to -1.25)	0.36 (0.26 to 0.47)	0.337
Iran	476663.68 (410176.43 to 550340.69)	3669.2 (3157.4 to 4236.34)	-3.26 (-3.47 to -3.06)	0.18 (-0.73 to 1.09)	0.697
Mauritania	24668.59 (21821.62 to 28385.76)	6876.11 (6082.55 to 7912.24)	-1.28 (-1.41 to -1.14)	1.63 (0.96 to 2.31)	0.499
Pakistan	469212.71 (399758.77 to 544381.97)	2366.65 (2016.34 to 2745.8)	-2.63 (-2.87 to -2.39)	-0.52 (-0.69 to -0.35)	0.504
Jamaica	14983.8 (13716.29 to 16444.97)	2833.05 (2593.4 to 3109.33)	-1.77 (-1.96 to -1.58)	0.31 (-0.12 to 0.74)	0.683
Japan	2012230.22 (1714898.22 to 2294289.76)	3854.78 (3285.19 to 4395.11)	-5.75 (-6.12 to -5.38)	0.8 (0.72 to 0.88)	0.871
Bahrain	4688.37 (4263.58 to 5192.23)	2887.42 (2625.8 to 3197.73)	-3.82 (-4.08 to -3.57)	0.89 (-0.15 to 1.94)	0.753
Belgium	157621.94 (140992.59 to 176770.03)	4169.04 (3729.2 to 4675.5)	-4.22 (-4.48 to -3.96)	0.18 (0.16 to 0.2)	0.854
Bhutan	2932.07 (2624.76 to 3241.96)	2944.14 (2635.56 to 3255.3)	-1.53 (-1.61 to -1.46)	0.74 (0.73 to 0.75)	0.473
Cambodia	71124.07 (64396.28 to 79149.77)	3271.86 (2962.37 to 3641.06)	-1.2 (-1.25 to -1.16)	0.83 (0.57 to 1.08)	0.474
Denmark	76386.02 (67644.7 to 85532.81)	3967.65 (3513.6 to 4442.75)	-3.99 (-4.18 to -3.8)	0.2 (0.09 to 0.31)	0.896
Eswatini	7246.15 (6351.59 to 8268.32)	8003.31 (7015.28 to 9132.29)	-1.1 (-1.29 to -0.91)	0.87 (0.57 to 1.18)	0.585
Lesotho	3185.76 (2715.74 to 3688.96)	1764.43 (1504.11 to 2043.13)	-0.55 (-0.63 to -0.47)	0.63 (-0.48 to 1.77)	0.510
Morocco	208407.48 (179087.81 to 244070.62)	3468.84 (2980.83 to 4062.44)	-0.82 (-0.9 to -0.74)	1.69 (0.46 to 2.94)	0.563
Norway	98178.7 (85052.9 to 111930.06)	6053.93 (5244.56 to 6901.87)	-5.69 (-6.05 to -5.34)	-0.4 (-0.96 to 0.16)	0.916
Saudi Arabia	88664.2 (77898.78 to 102176.92)	2944.97 (2587.4 to 3393.79)	-1.2 (-1.29 to -1.11)	2.8 (0.78 to 4.87)	0.815
Spain	747770.27 (661149.71 to 843046.77)	4832.09 (4272.35 to 5447.77)	-2.05 (-2.19 to -1.92)	-0.08 (-0.23 to 0.06)	0.769
Sweden	273049.28 (234691.92 to 313131.33)	8110.85 (6971.45 to 9301.47)	-4.15 (-4.65 to -3.64)	0.31 (-0.2 to 0.83)	0.887
Thailand	717139.24 (634767.43 to 818193.41)	3689.37 (3265.6 to 4209.25)	-2.94 (-3.09 to -2.79)	0.56 (0.39 to 0.73)	0.683
Netherlands	417867.48 (361530.26 to 474756.31)	7141.97 (6179.08 to 8114.28)	-2.1 (-2.46 to -1.73)	-0.13 (-0.53 to 0.28)	0.888
Tonga	745.72 (594.08 to 933.96)	5552.41 (4423.34 to 6953.99)	-1.11 (-1.18 to -1.04)	-0.86 (-1.15 to -0.57)	0.626
Kyrgyzstan	24502.91 (20552.29 to 29202.93)	2897.18 (2430.07 to 3452.9)	-3.4 (-3.63 to -3.17)	0.91 (0.5 to 1.31)	0.604
Laos	20782.39 (17725.06 to 24446.5)	2688.02 (2292.58 to 3161.94)	-1.85 (-1.93 to -1.77)	1.07 (0.88 to 1.25)	0.489
Lebanon	59290.33 (48040.05 to 72183.93)	6049.86 (4901.9 to 7365.49)	-1.28 (-1.33 to -1.22)	0.13 (-1.32 to 1.61)	0.745
Malaysia	127608.21 (112814.95 to 145109.69)	2602.77 (2301.04 to 2959.74)	-3.91 (-4.13 to -3.69)	0.59 (0.53 to 0.66)	0.743
Mongolia	14204.34 (12777.08 to 15911.94)	3597.8 (3236.29 to 4030.32)	-2.76 (-2.95 to -2.57)	0.61 (0.6 to 0.62)	0.618

Montenegro	4331.72<br>(3644.38 to 5084.42)	2484.16<br>(2089.99 to 2915.83)	-0.82<br>(-0.98 to -0.66)	0.18<br>(-0.25 to 0.62)	0.796
New Zealand	80699.27<br>(69801.4 to 91915.79)	5657.46<br>(4893.46 to 6443.8)	-4.23<br>(-4.66 to -3.8)	-1.59<br>(-2.4 to -0.78)	0.849
North Macedonia	62714.19<br>(56809.23 to 69456.27)	10577.43<br>(9581.49 to 11714.55)	-2.9<br>(-3.31 to -2.5)	0.36<br>(0.14 to 0.59)	0.751
Northern Mariana Islands	106.79<br>(96.45 to 118.57)	1098.67<br>(992.24 to 1219.83)	-3.14<br>(-3.89 to -2.38)	1.34<br>(0.81 to 1.87)	0.772
Palestine	20896.01<br>(17638.88 to 25340.26)	4856.59<br>(4099.58 to 5889.52)	-1.56<br>(-1.62 to -1.5)	0.96<br>(-0.12 to 2.05)	0.631
Algeria	236605.34<br>(193344.43 to 292129.1)	3895.78<br>(3183.48 to 4810)	-1.31<br>(-1.37 to -1.24)	0.47<br>(-0.2 to 1.15)	0.660
Bangladesh	655287.88<br>(585034.46 to 735477.3)	2804.43<br>(2503.77 to 3147.62)	-2.7<br>(-2.9 to -2.5)	1.54<br>(1.41 to 1.67)	0.492
China	6679616.01<br>(5797760.68 to 7643806.4)	1762.64<br>(1529.93 to 2017.07)	-3.28<br>(-3.47 to -3.1)	0.72<br>(0.51 to 0.93)	0.722
Bolivia	21301.52<br>(18262.83 to 24680.93)	1378.32<br>(1181.7 to 1596.98)	-1.87<br>(-1.96 to -1.78)	-0.14<br>(-0.85 to 0.57)	0.599
Portugal	449739.54<br>(396323.55 to 502408.38)	11559.51<br>(10186.58 to 12913.25)	-2.08<br>(-2.33 to -1.83)	0.09<br>(-0.14 to 0.32)	0.744
Andorra	1726.39<br>(1529.51 to 1941.12)	6524.09<br>(5780.08 to 7335.57)	-2.15<br>(-2.31 to -1.99)	0.05<br>(-0.18 to 0.27)	0.869
Monaco	925.66<br>(826.83 to 1037.4)	5916.49<br>(5284.81 to 6630.74)	-0.75<br>(-0.8 to -0.71)	-0.15<br>(-0.16 to -0.13)	0.908
Puerto Rico	52524.47<br>(47111.58 to 59467.43)	4499.41<br>(4035.72 to 5094.16)	-3.12<br>(-3.31 to -2.92)	0.48<br>(0.29 to 0.67)	0.826
Albania	23720.8<br>(20484.09 to 27035.82)	3016.78<br>(2605.14 to 3438.39)	-2.18<br>(-2.39 to -1.96)	0.13<br>(-0.2 to 0.47)	0.707
Angola	56808.47<br>(50812.98 to 64007.88)	2937.92<br>(2627.85 to 3310.24)	-2.28<br>(-2.41 to -2.15)	0.4<br>(-0.28 to 1.09)	0.454
Armenia	6567.87<br>(5677.21 to 7633.45)	834.95<br>(721.72 to 970.41)	-2.9<br>(-3.68 to -2.12)	0.02<br>(-0.35 to 0.38)	0.702
Austria	138150.01<br>(118515.46 to 159090.61)	4674.85<br>(4010.44 to 5383.46)	-3.66<br>(-3.77 to -3.56)	0.04<br>(-0.05 to 0.12)	0.854
Azerbaijan	63524.06<br>(56987.25 to 71707.59)	3315.8<br>(2974.59 to 3742.96)	-2.36<br>(-2.51 to -2.21)	0.48<br>(-0.17 to 1.15)	0.695
Belarus	148873.66<br>(119939.84 to 186970.19)	5175.33<br>(4169.5 to 6499.69)	-3.57<br>(-3.72 to -3.41)	0.22<br>(-1.08 to 1.54)	0.784
Benin	26093.16<br>(23594.68 to 28897.93)	3154.92<br>(2852.83 to 3494.05)	-1.53<br>(-1.62 to -1.44)	1.28<br>(0.81 to 1.75)	0.373
Botswana	10026.93<br>(8745.21 to 11628.05)	4136.12<br>(3607.41 to 4796.59)	-1.71<br>(-1.82 to -1.6)	0.94<br>(0.14 to 1.74)	0.643
Bulgaria	69600.98<br>(56738.29 to 84166.5)	2928.22<br>(2387.06 to 3541.01)	-4.24<br>(-4.44 to -4.03)	0.37<br>(-1.51 to 2.27)	0.768
Burundi	38388.14<br>(33789.3 to 43760.1)	4795.35<br>(4220.88 to 5466.4)	-1.59<br>(-1.68 to -1.49)	-0.67<br>(-0.79 to -0.54)	0.289
Cabo Verde	1559.4<br>(1423.83 to 1732.85)	2034.54<br>(1857.67 to 2260.85)	-2.14<br>(-2.39 to -1.88)	0.75<br>(0.66 to 0.84)	0.534
Cameroon	36172.12<br>(29782.4 to 44746.18)	1777.08<br>(1463.16 to 2198.31)	-1.37<br>(-1.42 to -1.32)	0.6<br>(-0.32 to 1.52)	0.480
Chad	23636.42<br>(20352.23 to 27796.32)	2545.6<br>(2191.89 to 2993.61)	-0.8<br>(-0.87 to -0.73)	0.78<br>(-0.54 to 2.12)	0.240
Chile	522111.15<br>(455911.33 to 593491.01)	11660.3<br>(10181.86 to 13254.42)	-1.21<br>(-1.41 to -1.02)	1.94<br>(1.01 to 2.88)	0.772
Colombia	141297.31<br>(121647.11 to 165170.3)	1477.46<br>(1271.99 to 1727.09)	-4.76<br>(-4.92 to -4.61)	0.86<br>(0.28 to 1.43)	0.655
Costa Rica	35152.64<br>(29343.69 to 42083.96)	3671.44<br>(3064.73 to 4395.36)	-2.21<br>(-2.34 to -2.09)	-0.85<br>(-1.02 to -0.69)	0.700
Côte d'Ivoire	68724.89<br>(59606.42 to 80725.35)	3832.57<br>(3324.06 to 4501.79)	-0.93<br>(-1.04 to -0.83)	1.41<br>(0.89 to 1.93)	0.426
Croatia	72962.94<br>(63204.39 to 83888.63)	4892.49<br>(4238.13 to 5625.1)	-3.7<br>(-3.84 to -3.56)	0.33<br>(0.3 to 0.37)	0.798

Cuba	103883.83<br>(91360.13 to 117360.34)	3040.16<br>(2673.66 to 3434.55)	-0.32<br>(-0.47 to -0.18)	-0.33<br>(-0.43 to -0.23)	0.669
Cyprus	34398.64<br>(27588.76 to 41725.09)	9842.2<br>(7893.74 to 11938.45)	-1.21<br>(-1.25 to -1.17)	-0.82<br>(-3.88 to 2.34)	0.836
Djibouti	3393.28<br>(3015.29 to 3843.18)	3272.09<br>(2907.59 to 3705.92)	-1.72<br>(-1.79 to -1.65)	0.59<br>(0.38 to 0.79)	0.488
Ecuador	28033.43<br>(24011.25 to 32715.64)	1012.94<br>(867.61 to 1182.13)	-2.14<br>(-2.2 to -2.08)	0.26<br>(0.23 to 0.28)	0.661
El Salvador	56634.3<br>(48114.89 to 66476.42)	5538.36<br>(4705.23 to 6500.83)	-1.44<br>(-1.55 to -1.34)	0.33<br>(0.03 to 0.63)	0.564
Equatorial Guinea	2227.52<br>(1969.13 to 2537.47)	2816.18<br>(2489.51 to 3208.05)	-2.56<br>(-2.61 to -2.5)	-0.34<br>(-0.97 to 0.31)	0.658
Estonia	13240.7<br>(11544.05 to 15166.31)	3026.62<br>(2638.79 to 3466.78)	-2.83<br>(-2.95 to -2.71)	0.49<br>(0.14 to 0.85)	0.845
Fiji	5417.43<br>(4550.86 to 6437.17)	3961.83<br>(3328.09 to 4707.58)	-2.61<br>(-2.72 to -2.5)	0.14<br>(-2.09 to 2.42)	0.675
Finland	154501.75<br>(137300.16 to 172460.16)	7676.47<br>(6821.8 to 8568.74)	-1.22<br>(-1.26 to -1.17)	0.48<br>(0.14 to 0.82)	0.860
Ghana	60440.52<br>(54020.1 to 68784.16)	2216.24<br>(1980.81 to 2522.18)	-1<br>(-1.12 to -0.89)	1.79<br>(1.59 to 1.99)	0.565
Guatemala	29727.04<br>(25976.54 to 34012.19)	1623.74<br>(1418.88 to 1857.8)	-2.39<br>(-2.5 to -2.28)	1.89<br>(1.72 to 2.07)	0.540
Guinea	32557.27<br>(27840.5 to 38158.81)	3563.79<br>(3047.48 to 4176.94)	-0.96<br>(-1.03 to -0.9)	0.18<br>(-1.43 to 1.81)	0.336
Guinea-Bissau	4758.25<br>(4301.19 to 5309.18)	4142.28<br>(3744.39 to 4621.89)	-1.26<br>(-1.38 to -1.14)	0.95<br>(0.95 to 0.95)	0.353
Guyana	3733.31<br>(3386.69 to 4114.66)	3315.04<br>(3007.25 to 3653.66)	-2.08<br>(-2.17 to -1.98)	1.94<br>(1.6 to 2.28)	0.651
Haiti	75623.99<br>(66631.83 to 86471.33)	6381.3<br>(5622.52 to 7296.62)	-1.08<br>(-1.14 to -1.02)	0.16<br>(-0.18 to 0.51)	0.448
Honduras	25531.85<br>(21866.95 to 30491.73)	2427.74<br>(2079.26 to 2899.36)	-0.76<br>(-0.81 to -0.71)	0.55<br>(0.39 to 0.7)	0.513
Iceland	6082.78<br>(5219.68 to 7040.98)	6246.04<br>(5359.77 to 7229.96)	-3.31<br>(-3.42 to -3.2)	0.09<br>(-0.02 to 0.19)	0.876
India	10085239.95<br>(8582926.02 to<br>11777049.89)	5016.4<br>(4269.15 to 5857.91)	-0.85<br>(-0.99 to -0.7)	1.42<br>(1.42 to 1.43)	0.575
Indonesia	1447320.06<br>(1228832.52 to 1693338.7)	3455.91<br>(2934.21 to 4043.35)	-1.41<br>(-1.59 to -1.23)	-0.81<br>(-1.02 to -0.6)	0.657
Iraq	139808.99<br>(128318.64 to 154354.68)	3598.51<br>(3302.76 to 3972.9)	-2.58<br>(-2.71 to -2.45)	1.1<br>(0.66 to 1.54)	0.663
Italy	855011.82<br>(717981.66 to 992394.01)	3763.79<br>(3160.58 to 4368.55)	-5.62<br>(-5.97 to -5.27)	-0.01<br>(-0.04 to 0.02)	0.806
Kazakhstan	82953.65<br>(70829.22 to 98462.14)	2613.99<br>(2231.93 to 3102.69)	-0.62<br>(-0.87 to -0.36)	0.86<br>(-0.85 to 2.6)	0.725
Kenya	101266.02<br>(85696.98 to 119044.78)	2707.09<br>(2290.89 to 3182.36)	-1.02<br>(-1.1 to -0.93)	-0.18<br>(-0.49 to 0.12)	0.524
Kiribati	807.94<br>(728.31 to 898.46)	6424.02<br>(5790.89 to 7143.78)	-1.78<br>(-1.86 to -1.69)	-0.34<br>(-1.24 to 0.56)	0.527
South Korea	675237.33<br>(601997.11 to 755404.93)	4037.59<br>(3599.65 to 4516.96)	-3.31<br>(-3.46 to -3.15)	1.05<br>(0.23 to 1.89)	0.887
Latvia	18645.44<br>(15785.71 to 21518.33)	2833.57<br>(2398.97 to 3270.17)	-3.96<br>(-4.17 to -3.74)	0.24<br>(-1.49 to 2)	0.831
Liberia	8210.27<br>(7332.12 to 9194.68)	2487.04<br>(2221.04 to 2785.24)	-0.97<br>(-1.09 to -0.85)	0.9<br>(0.71 to 1.09)	0.352
Lithuania	22761.26<br>(18673.46 to 27504.97)	2351.9<br>(1929.51 to 2842.07)	-3<br>(-3.17 to -2.84)	0.16<br>(-1 to 1.34)	0.856
Madagascar	102018.01<br>(91643.39 to 113886.94)	5524.4<br>(4962.6 to 6167.12)	-0.83<br>(-0.99 to -0.67)	0.25<br>(0.14 to 0.35)	0.400
Malawi	47734.56<br>(40689.15 to 56257.92)	4082.92<br>(3480.3 to 4811.96)	-0.67<br>(-0.73 to -0.6)	1.48<br>(1.02 to 1.95)	0.385
Maldives	766.49<br>(700.66 to 845.53)	1409.07<br>(1288.06 to 1554.39)	-3.9<br>(-4.12 to -3.67)	-0.05<br>(-0.21 to 0.12)	0.651

Mali	38116.93<br>(32630.15 to 45085.25)	2633.53<br>(2254.44 to 3114.97)	-0.8<br>(-0.89 to -0.72)	1.53<br>(1.28 to 1.78)	0.269
Malta	12254.04<br>(10560.14 to 14023.31)	7765.39<br>(6691.97 to 8886.58)	-2.99<br>(-3.11 to -2.86)	0.22<br>(-0.48 to 0.92)	0.802
Mauritius	11665.27<br>(10184.01 to 13482.53)	3447.88<br>(3010.06 to 3985)	-3.47<br>(-3.63 to -3.32)	0.99<br>(0.72 to 1.26)	0.718
Moldova	19374.44<br>(17186.05 to 21898.71)	1834.99<br>(1627.72 to 2074.07)	-5.22<br>(-5.5 to -4.95)	0.35<br>(0.09 to 0.62)	0.732
Mozambique	65603.41<br>(58451.04 to 73451.58)	3666.77<br>(3267 to 4105.43)	-0.59<br>(-0.73 to -0.45)	0.17<br>(-0.17 to 0.52)	0.326
Namibia	7418.57<br>(6547.68 to 8597.98)	3283.33<br>(2897.88 to 3805.31)	-1.67<br>(-1.78 to -1.55)	0.58<br>(0.31 to 0.85)	0.618
Nauru	23.96<br>(21.46 to 26.94)	2592.23<br>(2321.5 to 2914.76)	-2.39<br>(-2.61 to -2.16)	0.41<br>(0.04 to 0.78)	0.625
Nicaragua	23488.97<br>(20306.49 to 27671.67)	2885.5<br>(2494.55 to 3399.33)	-2.46<br>(-2.65 to -2.27)	0.59<br>(0.44 to 0.74)	0.524
Niue	7.06<br>(6.32 to 7.85)	1804.57<br>(1614.76 to 2006.42)	-2.97<br>(-3.16 to -2.78)	-0.48<br>(-1.2 to 0.24)	0.726
Palau	72.82<br>(66.15 to 80.35)	1738.95<br>(1579.69 to 1918.92)	-3.48<br>(-3.75 to -3.2)	-0.54<br>(-0.67 to -0.41)	0.754
Panama	14478.26<br>(12851.46 to 16409.4)	1947.14<br>(1728.36 to 2206.86)	-2.09<br>(-2.23 to -1.96)	0.92<br>(-0.21 to 2.08)	0.709
Paraguay	30515.59<br>(27711.6 to 33687.92)	3082.06<br>(2798.85 to 3402.46)	-0.19<br>(-0.3 to -0.08)	0.93<br>(0.63 to 1.22)	0.636
Peru	88698.71<br>(76674.14 to 103692.51)	1585.76<br>(1370.79 to 1853.82)	-1.46<br>(-1.52 to -1.41)	0.17<br>(-0.42 to 0.77)	0.662
Poland	1283977.64<br>(1112193.59 to 1474037.12)	10597.94<br>(9180.04 to 12166.69)	-4.08<br>(-4.57 to -3.58)	0.44<br>(0.04 to 0.85)	0.812
Rwanda	97700.24<br>(83630.68 to 116584.16)	9239.09<br>(7908.59 to 11024.86)	-1.58<br>(-1.74 to -1.41)	1.45<br>(1.19 to 1.7)	0.436
San Marino	706.62<br>(627.24 to 793.28)	5974.6<br>(5303.49 to 6707.4)	-0.73<br>(-0.83 to -0.64)	0.02<br>(-0.1 to 0.14)	0.888
Senegal	35335.1<br>(30034.79 to 40870.86)	2736.68<br>(2326.18 to 3165.42)	-0.79<br>(-0.86 to -0.72)	1.59<br>(1.12 to 2.06)	0.408
Serbia	104702.11<br>(94515.07 to 116617.55)	3732.4<br>(3369.26 to 4157.16)	-2.1<br>(-2.24 to -1.96)	0.7<br>(0.59 to 0.81)	0.792
Seychelles	446.87<br>(405.18 to 498.72)	2154.3<br>(1953.34 to 2404.27)	-1.99<br>(-2.18 to -1.8)	0.28<br>(-0.03 to 0.59)	0.730
Sierra Leone	19911.36<br>(17974.77 to 22215.03)	3296.13<br>(2975.54 to 3677.48)	-1.05<br>(-1.14 to -0.95)	0.67<br>(0.42 to 0.93)	0.359
Singapore	54534.43<br>(47430.07 to 63492.83)	3590.56<br>(3122.81 to 4180.39)	-4.6<br>(-4.9 to -4.29)	0.35<br>(-0.15 to 0.85)	0.856
Slovenia	44723.5<br>(37113.95 to 53628.83)	6108.19<br>(5068.9 to 7324.45)	-3.12<br>(-3.24 to -2.99)	-0.05<br>(-0.51 to 0.41)	0.842
South Africa	152804.81<br>(131770.87 to 178654.18)	1937.05<br>(1670.41 to 2264.73)	-1.67<br>(-2.04 to -1.31)	-0.59<br>(-0.93 to -0.25)	0.680
South Sudan	24734.49<br>(21908.3 to 28291.3)	3971.99<br>(3518.15 to 4543.16)	-1.32<br>(-1.36 to -1.28)	-1.13<br>(-1.33 to -0.93)	0.278
Sudan	108362.95<br>(89491.79 to 130373.1)	3480.05<br>(2874.01 to 4186.9)	-2.13<br>(-2.26 to -1.99)	-1<br>(-3.29 to 1.34)	0.542
Suriname	2456.72<br>(2246.35 to 2711.98)	2199.34<br>(2011 to 2427.85)	-1.86<br>(-1.98 to -1.75)	0.21<br>(-0.48 to 0.9)	0.634
Tajikistan	31384.9<br>(28542.79 to 34442.11)	3055.55<br>(2778.85 to 3353.19)	-2.46<br>(-2.58 to -2.35)	-0.24<br>(-0.34 to -0.14)	0.542
Republic of the Congo	18223.47<br>(15674.82 to 21031.48)	4193.85<br>(3607.32 to 4840.07)	-1.64<br>(-1.71 to -1.57)	0.31<br>(0.28 to 0.35)	0.583
Gambia	5763.8<br>(5194.29 to 6431.33)	3727.23<br>(3358.95 to 4158.89)	-0.67<br>(-0.76 to -0.58)	1.14<br>(1.03 to 1.25)	0.410
Marshall Islands	165.55<br>(147.9 to 185.9)	2825.64<br>(2524.38 to 3172.87)	-2.61<br>(-2.69 to -2.53)	0.35<br>(-0.13 to 0.83)	0.574
Niger	51325.17<br>(46029.99 to 58161.04)	3686.58<br>(3306.24 to 4177.59)	-0.98<br>(-1.11 to -0.85)	0.7<br>(0.62 to 0.78)	0.168
Philippines	709844.85<br>(611135.92 to 826088.56)	5090.29<br>(4382.45 to 5923.87)	-2.27<br>(-2.35 to -2.19)	-0.13<br>(-0.78 to 0.52)	0.651

Myanmar	221589.71 (193424.96 to 258896.94)	2624.81 (2291.19 to 3066.73)	-2.13 (-2.23 to -2.03)	0.98 (0.09 to 1.87)	0.534
Trinidad and Tobago	4733.43 (4138.36 to 5530.69)	1358.86 (1188.03 to 1587.73)	-2.14 (-2.54 to -1.74)	0.35 (-1.73 to 2.48)	0.769
Tunisia	100407.64 (87726.39 to 115381.89)	4296.19 (3753.59 to 4936.9)	-1.47 (-1.61 to -1.34)	1.11 (0.66 to 1.57)	0.682
Turkey	1264291.81 (1147610.75 to 1391817.94)	7652.52 (6946.27 to 8424.41)	-1.49 (-1.62 to -1.35)	0.73 (0.61 to 0.86)	0.713
Uganda	118283.74 (101554.12 to 138664.89)	4987.73 (4282.29 to 5847.16)	-1.26 (-1.34 to -1.17)	0.82 (0.56 to 1.09)	0.423
Uzbekistan	337920.45 (284884.74 to 396635.94)	7269.22 (6128.33 to 8532.28)	-2.03 (-2.25 to -1.81)	0.25 (0.16 to 0.35)	0.663
Vanuatu	905.37 (807.02 to 1021.22)	3157.38 (2814.38 to 3561.39)	-2.42 (-2.52 to -2.32)	-0.2 (-0.56 to 0.17)	0.473
Yemen	154622.24 (140242.17 to 172512.36)	6875.38 (6235.96 to 7670.88)	-1.99 (-2.06 to -1.93)	0.24 (-0.36 to 0.85)	0.450
Zambia	19542.46 (16488.15 to 22968.85)	1809.22 (1526.46 to 2126.43)	-1.12 (-1.27 to -0.97)	1.01 (-0.33 to 2.37)	0.506
Zimbabwe	41173.07 (36187.98 to 47014.88)	3718.43 (3268.21 to 4246.01)	0.05 (0.01 to 0.1)	0.22 (0.13 to 0.32)	0.474
Romania	244258.75 (201561.16 to 297520.5)	4067.35 (3356.36 to 4954.26)	-3.06 (-3.19 to -2.92)	0.32 (0.13 to 0.5)	0.768
Russia	784186.43 (672275.14 to 914384.59)	1844.92 (1581.63 to 2151.23)	-6.05 (-6.29 to -5.8)	0.53 (0.27 to 0.8)	0.809
Saint Kitts and Nevis	294.63 (267.04 to 327.11)	2287.64 (2073.39 to 2539.79)	-1.99 (-2.07 to -1.91)	0.39 (-0.02 to 0.8)	0.755
Saint Lucia	1617.52 (1492.36 to 1766.19)	3843.74 (3546.33 to 4197.04)	-2.21 (-2.36 to -2.07)	0.31 (-0.41 to 1.03)	0.673
Saint Vincent and the Grenadines	728.37 (673.63 to 796.27)	2864.72 (2649.42 to 3131.74)	-1.35 (-1.49 to -1.21)	0.62 (-0.35 to 1.61)	0.637
Slovakia	40842.46 (34798.04 to 47909.66)	2489.97 (2121.47 to 2920.83)	-1.69 (-1.82 to -1.56)	-0.41 (-1.17 to 0.35)	0.811
Vietnam	827305.33 (712791.11 to 966108.35)	4735.96 (4080.42 to 5530.55)	-1.16 (-1.24 to -1.09)	1.74 (0.63 to 2.85)	0.628
Solomon Islands	2150.31 (1924.19 to 2424.56)	3964.02 (3547.17 to 4469.59)	-1.65 (-1.74 to -1.55)	-0.68 (-1.16 to -0.2)	0.429
Eritrea	20549.15 (18389.97 to 23275.88)	4663.81 (4173.76 to 5282.66)	-1.5 (-1.58 to -1.42)	0 (-0.1 to 0.09)	0.404
Israel	74009.98 (65408.09 to 83249.08)	3703.19 (3272.79 to 4165.49)	-3.38 (-3.61 to -3.15)	-0.08 (-0.47 to 0.3)	0.809
Kuwait	13146.68 (10835.83 to 15825.24)	2819.82 (2324.17 to 3394.34)	-2.11 (-2.38 to -1.84)	0.28 (-0.22 to 0.77)	0.847
Libya	37237.46 (33418.41 to 41325.33)	4452.56 (3995.91 to 4941.35)	-1.58 (-1.63 to -1.54)	-2.56 (-3.12 to -2)	0.726
Qatar	4018.72 (3284.42 to 4905.64)	2623.46 (2144.1 to 3202.45)	-1.64 (-1.81 to -1.47)	2.93 (2.36 to 3.51)	0.847
Oman	9995.28 (8039.64 to 12405.77)	3199.98 (2573.88 to 3971.69)	-0.98 (-1.05 to -0.91)	1.11 (0.13 to 2.1)	0.773
Switzerland	165734.94 (140495.09 to 192525.25)	5654.87 (4793.69 to 6568.96)	-3.07 (-3.2 to -2.95)	-0.91 (-1.43 to -0.38)	0.933
Syria	109777.06 (94257.99 to 127792.84)	4646.25 (3989.41 to 5408.76)	-0.54 (-0.64 to -0.43)	1.04 (0.33 to 1.75)	0.623
Taiwan	448199.18 (405062.33 to 495477.53)	5952.23 (5379.36 to 6580.1)	-2.71 (-2.83 to -2.58)	1.07 (0.4 to 1.75)	0.875
Togo	26282.16 (21559.51 to 33416.28)	4207.25 (3451.25 to 5349.28)	-0.36 (-0.43 to -0.3)	0.62 (0.34 to 0.89)	0.409
Tokelau	5.67 (5.16 to 6.21)	2241.55 (2040.98 to 2457.8)	-3.06 (-3.22 to -2.9)	-0.1 (-0.6 to 0.42)	0.686
Turkmenistan	8727.92 (7620.36 to 10036.14)	1233.28 (1076.78 to 1418.13)	-5.73 (-5.99 to -5.48)	0.44 (0.38 to 0.51)	0.682

Tuvalu	47.57<br>(42.9 to 53.31)	2610.6<br>(2354.01 to 2925.46)	-3.09<br>(-3.2 to -2.99)	0.06<br>(-0.38 to 0.5)	0.577
Ukraine	174245<br>(146070.27 to 207190.27)	1283.28<br>(1075.78 to 1525.92)	-5.43<br>(-5.73 to -5.13)	-0.57<br>(-1.71 to 0.58)	0.761
Comoros	3561.32<br>(3028.52 to 4180.1)	4405.91<br>(3746.75 to 5171.43)	-1.13<br>(-1.23 to -1.03)	0.02<br>(-0.83 to 0.87)	0.476
UAE	80097.92<br>(69153.3 to 94232.65)	11154.47<br>(9630.32 to 13122.88)	-2.38<br>(-2.55 to -2.21)	0.4<br>(-0.65 to 1.45)	0.849
UK	1848283.81<br>(1631912.67 to 2074654.03)	8788.72<br>(7759.86 to 9865.12)	-2.62<br>(-2.88 to -2.37)	0.95<br>(0.84 to 1.06)	0.859
Mexico	254116.29<br>(220579.55 to 291805.5)	1179.17<br>(1023.55 to 1354.06)	-4.78<br>(-4.99 to -4.58)	0.06<br>(0.05 to 0.06)	0.665
Tanzania	234395.44<br>(186081.16 to 289150.49)	5686.2<br>(4514.14 to 7014.5)	-0.55<br>(-0.6 to -0.51)	0.4<br>(-0.08 to 0.87)	0.447
USA	11257841.44<br>(9777863.07 to 12819234.64)	11230.02<br>(9753.7 to 12787.55)	1.04<br>(0.24 to 1.86)	-0.39<br>(-0.55 to -0.22)	0.862
US Virgin Islands	1169.24<br>(1024.1 to 1354.19)	3632.38<br>(3181.49 to 4206.95)	-1<br>(-1.17 to -0.83)	0.14<br>(-0.3 to 0.57)	0.822

**Cause**	**ILD & PS**				
**Location**	**Prevalence number**	**Prevalence rate**	**EAPC 1990-2021(95% CI)**	**EAPC 2019-2021(95% CI)**	**2021SDI**
American Samoa	6.28<br>(5.53 to 7.16)	74.36<br>(65.42 to 84.72)	-0.61<br>(-0.65 to -0.56)	-0.05<br>(-0.39 to 0.3)	0.724
Antigua and Barbuda	13.73<br>(12.05 to 15.56)	72.75<br>(63.86 to 82.44)	1.55<br>(1.47 to 1.63)	0.32<br>(0.13 to 0.51)	0.750
Egypt	12842.33<br>(11002.91 to 15098.97)	116.05<br>(99.43 to 136.45)	1.42<br>(1.4 to 1.45)	0.96<br>(0.92 to 0.99)	0.607
Argentina	34476.88<br>(31591.58 to 37566.73)	369.4<br>(338.48 to 402.5)	1.33<br>(1.12 to 1.54)	-0.06<br>(-0.23 to 0.12)	0.723
Australia	25615.63<br>(22903.88 to 28597.28)	345.79<br>(309.18 to 386.04)	2.42<br>(2.18 to 2.65)	0.71<br>(0.53 to 0.9)	0.844
Barbados	95.62<br>(85.99 to 105.22)	104.94<br>(94.37 to 115.47)	1.2<br>(1.07 to 1.33)	-0.14<br>(-0.25 to -0.03)	0.747
Belize	126.72<br>(114.67 to 138.89)	253.01<br>(228.95 to 277.3)	1.94<br>(1.75 to 2.14)	-0.4<br>(-0.48 to -0.31)	0.610
Bermuda	69.12<br>(63.08 to 75.27)	296.75<br>(270.83 to 323.17)	1.51<br>(1.39 to 1.63)	0.52<br>(0.28 to 0.76)	0.821
Venezuela	6632.16<br>(5980.02 to 7346.59)	126.76<br>(114.29 to 140.41)	0.82<br>(0.69 to 0.95)	-1.2<br>(-1.22 to -1.18)	0.597
Bosnia and Herzegovina	967.11<br>(845.43 to 1112.13)	88.3<br>(77.19 to 101.54)	0.14<br>(-0.17 to 0.44)	0.48<br>(0.38 to 0.59)	0.723
Brunei	324.17<br>(292.11 to 358.64)	541.42<br>(487.87 to 598.98)	-1.17<br>(-1.28 to -1.07)	1.05<br>(0.52 to 1.59)	0.810
Burkina Faso	463.45<br>(389.23 to 548.25)	30.83<br>(25.89 to 36.47)	-0.6<br>(-0.64 to -0.56)	0.63<br>(0.35 to 0.92)	0.285
Canada	67566.1<br>(61057.48 to 73983.72)	550.61<br>(497.57 to 602.91)	1.69<br>(1.52 to 1.87)	0.71<br>(0.51 to 0.9)	0.873
Central African Republic	183.29<br>(153.64 to 216.32)	51.24<br>(42.95 to 60.48)	-0.31<br>(-0.36 to -0.27)	-0.16<br>(-0.33 to 0.01)	0.309
Dominica	12.46<br>(11.03 to 13.92)	82.59<br>(73.1 to 92.32)	1.1<br>(0.99 to 1.22)	-0.3<br>(-0.35 to -0.25)	0.747
Bahamas	87.84<br>(78.22 to 97.06)	122.07<br>(108.7 to 134.88)	1.02<br>(0.88 to 1.15)	-0.32<br>(-0.41 to -0.22)	0.805
Cook Islands	6.18<br>(5.47 to 6.92)	131.19<br>(116.09 to 146.86)	0.19<br>(0.14 to 0.24)	0.97<br>(0.54 to 1.4)	0.779
Czech Republic	5390.11<br>(4823.81 to 6026.9)	154.29<br>(138.08 to 172.52)	3<br>(2.71 to 3.29)	-0.08<br>(-0.15 to -0.01)	0.828
North Korea	4383.84<br>(3781.36 to 5069.72)	77.79<br>(67.1 to 89.96)	0.26<br>(0.15 to 0.38)	0.17<br>(-0.22 to 0.56)	0.570

Sao Tome and Principe	20.46<br>(17.95 to 23.09)	111.92<br>(98.15 to 126.3)	0.62<br>(0.49 to 0.76)	0.67<br>(0.17 to 1.18)	0.505
DR Congo	4071.67<br>(3462.55 to 4745.54)	67.41<br>(57.33 to 78.57)	0.2<br>(0.05 to 0.35)	0.8<br>(0.47 to 1.12)	0.383
Timor-Leste	95.54<br>(81.35 to 110.95)	67.27<br>(57.27 to 78.12)	1.32<br>(1.2 to 1.44)	-0.3<br>(-0.61 to 0.01)	0.445
Sri Lanka	5007.19<br>(4361.89 to 5731.17)	103.96<br>(90.57 to 119)	1.87<br>(1.81 to 1.92)	1.28<br>(1.15 to 1.4)	0.702
Dominican Republic	903.59<br>(782.96 to 1036.55)	54.07<br>(46.86 to 62.03)	1.7<br>(1.61 to 1.78)	0.69<br>(0.46 to 0.92)	0.619
Uruguay	2613.5<br>(2373.18 to 2850.58)	289.01<br>(262.44 to 315.23)	1.73<br>(1.6 to 1.85)	0.46<br>(0.25 to 0.68)	0.719
Ethiopia	3032.18<br>(2545.99 to 3587.32)	44.29<br>(37.19 to 52.4)	0.61<br>(0.55 to 0.67)	-0.14<br>(-0.41 to 0.12)	0.359
Nepal	13503.23<br>(11721.77 to 15230.72)	341.18<br>(296.16 to 384.82)	1.38<br>(1.11 to 1.65)	1.22<br>(0.9 to 1.54)	0.433
Germany	67728.4<br>(61098 to 74613.97)	214.92<br>(193.88 to 236.77)	1.3<br>(1.14 to 1.47)	-0.27<br>(-0.35 to -0.19)	0.903
Nigeria	6812.3<br>(5721.17 to 8113.64)	45.41<br>(38.14 to 54.09)	-0.59<br>(-0.63 to -0.56)	-0.55<br>(-0.71 to -0.38)	0.503
Somalia	431.56<br>(361.06 to 511.8)	45.76<br>(38.29 to 54.27)	-0.34<br>(-0.42 to -0.27)	0.11<br>(-0.13 to 0.35)	0.078
Micronesia	15.9<br>(14.02 to 17.96)	120.86<br>(106.54 to 136.46)	0.18<br>(0.14 to 0.22)	0.79<br>(0.29 to 1.3)	0.588
Brazil	37388.97<br>(31916.83 to 43117.61)	86.33<br>(73.7 to 99.56)	-0.59<br>(-0.72 to -0.46)	-0.27<br>(-0.7 to 0.15)	0.653
France	48367.41<br>(44029.59 to 53208.09)	218.76<br>(199.14 to 240.65)	1.22<br>(1.09 to 1.35)	0.36<br>(0.17 to 0.54)	0.838
Gabon	144.86<br>(124.96 to 166.46)	81.04<br>(69.91 to 93.12)	0.46<br>(0.42 to 0.5)	0.6<br>(0.41 to 0.79)	0.635
Georgia	1047.62<br>(938.13 to 1163.13)	99.79<br>(89.36 to 110.79)	0.67<br>(0.43 to 0.9)	0.39<br>(0.3 to 0.48)	0.732
Luxembourg	325.54<br>(293.12 to 361.01)	183.19<br>(164.95 to 203.15)	1.29<br>(0.97 to 1.61)	-0.09<br>(-0.23 to 0.04)	0.884
Greenland	59.76<br>(53.3 to 66.49)	440.2<br>(392.62 to 489.73)	0.42<br>(0.34 to 0.49)	0.4<br>(-0.01 to 0.82)	0.826
Grenada	18.31<br>(16.28 to 20.61)	91.13<br>(81.05 to 102.62)	1.47<br>(1.42 to 1.52)	0.42<br>(0.1 to 0.74)	0.669
Guam	96.71<br>(87.29 to 106.54)	256.61<br>(231.64 to 282.71)	-0.71<br>(-0.78 to -0.65)	0.08<br>(-0.37 to 0.53)	0.804
Jordan	3618.93<br>(3239.16 to 4018.76)	287.85<br>(257.64 to 319.65)	0.94<br>(0.86 to 1.03)	-0.47<br>(-0.53 to -0.41)	0.725
Greece	6049.95<br>(5366.21 to 6729.49)	164.6<br>(146 to 183.09)	2.91<br>(2.67 to 3.15)	-0.17<br>(-0.58 to 0.25)	0.792
Hungary	4165.19<br>(3772.79 to 4592.32)	130.98<br>(118.64 to 144.41)	0.91<br>(0.83 to 1)	0.42<br>(0.08 to 0.76)	0.791
Papua New Guinea	1356.56<br>(1197.05 to 1521.76)	165.17<br>(145.75 to 185.29)	0.41<br>(0.36 to 0.46)	0.4<br>(-0.03 to 0.83)	0.418
Samoa	33.11<br>(29.62 to 36.74)	134.47<br>(120.29 to 149.23)	0.31<br>(0.28 to 0.34)	0.39<br>(0.07 to 0.71)	0.593
Ireland	6397.04<br>(5739.2 to 7077.05)	487.44<br>(437.32 to 539.26)	2.58<br>(2.51 to 2.65)	1.05<br>(0.64 to 1.46)	0.874
Afghanistan	960.15<br>(824.81 to 1113.57)	78.14<br>(67.13 to 90.63)	1.04<br>(0.88 to 1.19)	-0.89<br>(-1.02 to -0.75)	0.337
Iran	8734.89<br>(7429.36 to 10387.52)	67.24<br>(57.19 to 79.96)	0.39<br>(0.29 to 0.5)	-0.05<br>(-0.05 to -0.05)	0.697
Mauritania	158.4<br>(136.87 to 184.38)	44.15<br>(38.15 to 51.39)	-0.3<br>(-0.45 to -0.16)	0.82<br>(0.59 to 1.06)	0.499
Pakistan	24930.54<br>(20963.91 to 29452.38)	125.75<br>(105.74 to 148.55)	-1.23<br>(-1.29 to -1.17)	-1.12<br>(-1.35 to -0.89)	0.504
Jamaica	320.33<br>(283.01 to 361.53)	60.57<br>(53.51 to 68.36)	1.12<br>(0.96 to 1.28)	-0.05<br>(-0.17 to 0.08)	0.683
Japan	473976.17<br>(406413.6 to 548926.89)	907.98<br>(778.56 to 1051.57)	1.11<br>(1 to 1.22)	0.44<br>(0.03 to 0.86)	0.871
Bahrain	341.33<br>(300.97 to 386.56)	210.22<br>(185.36 to 238.07)	1.61<br>(1.41 to 1.82)	0.96<br>(0.74 to 1.18)	0.753

Belgium	7567.94 (6817.97 to 8400.46)	200.17 (180.33 to 222.19)	1.68 (1.39 to 1.98)	0.21 (-0.1 to 0.52)	0.854
Bhutan	288.15 (253.66 to 321.59)	289.33 (254.7 to 322.92)	2.31 (2.26 to 2.37)	0.99 (0.44 to 1.53)	0.473
Cambodia	1122.03 (936.28 to 1327.41)	51.62 (43.07 to 61.06)	1.23 (1.18 to 1.29)	1.12 (1.05 to 1.19)	0.474
Denmark	4830.6 (4368.69 to 5320.93)	250.91 (226.92 to 276.38)	0.76 (0.65 to 0.88)	0.18 (-0.16 to 0.53)	0.896
Eswatini	104.48 (90.68 to 118.37)	115.39 (100.16 to 130.73)	-0.32 (-0.41 to -0.24)	0.22 (0.08 to 0.37)	0.585
Lesotho	205.92 (179.76 to 234.22)	114.05 (99.56 to 129.72)	-0.06 (-0.18 to 0.06)	0.5 (0.06 to 0.94)	0.510
Morocco	6884.06 (5935.52 to 8012.27)	114.58 (98.79 to 133.36)	1.24 (1.21 to 1.26)	0.99 (0.99 to 0.99)	0.563
Norway	4672.53 (3989.22 to 5447.48)	288.12 (245.98 to 335.9)	0.4 (0.21 to 0.58)	0.37 (0.08 to 0.65)	0.916
Saudi Arabia	12484.89 (11037.94 to 13924.3)	414.68 (366.62 to 462.49)	1.96 (1.87 to 2.06)	-0.13 (-0.34 to 0.08)	0.815
Spain	61033.18 (55484.09 to 67145.76)	394.4 (358.54 to 433.9)	1.63 (1.39 to 1.88)	-0.29 (-0.33 to -0.25)	0.769
Sweden	10518.02 (8975.49 to 12205.13)	312.43 (266.61 to 362.55)	0.6 (0.56 to 0.65)	0.15 (-0.25 to 0.55)	0.887
Thailand	9617.75 (8150.48 to 11324.03)	49.48 (41.93 to 58.26)	0.11 (-0.02 to 0.23)	0.87 (0.66 to 1.08)	0.683
Netherlands	14105.21 (12638.23 to 15693.15)	241.08 (216.01 to 268.22)	2.45 (2.23 to 2.66)	0.15 (-0.21 to 0.52)	0.888
Tonga	13.29 (11.8 to 14.92)	98.93 (87.83 to 111.09)	0.31 (0.26 to 0.35)	0.51 (0.1 to 0.92)	0.626
Kyrgyzstan	372.64 (311.16 to 443.39)	44.06 (36.79 to 52.43)	-1.29 (-1.38 to -1.21)	0.52 (0.41 to 0.63)	0.604
Laos	502.47 (426.62 to 581.13)	64.99 (55.18 to 75.16)	1.08 (1.03 to 1.14)	1.27 (1.14 to 1.39)	0.489
Lebanon	1298.59 (1129.07 to 1509.62)	132.51 (115.21 to 154.04)	1.32 (1.19 to 1.44)	-0.24 (-0.29 to -0.19)	0.745
Malaysia	5823.6 (5148.91 to 6627.76)	118.78 (105.02 to 135.18)	1.53 (1.5 to 1.55)	0.84 (0.75 to 0.92)	0.743
Mongolia	421.44 (370.3 to 480.07)	106.75 (93.79 to 121.6)	0.36 (0.29 to 0.44)	0.64 (0.48 to 0.8)	0.618
Montenegro	95.65 (81.57 to 111.24)	54.85 (46.78 to 63.79)	-0.06 (-0.14 to 0.03)	0.24 (0.23 to 0.25)	0.796
New Zealand	3888.27 (3341.79 to 4461.34)	272.59 (234.28 to 312.76)	0.42 (0.31 to 0.53)	0.32 (0.18 to 0.45)	0.849
North Macedonia	322.11 (275.2 to 376.28)	54.33 (46.41 to 63.46)	0.02 (-0.09 to 0.13)	0.19 (0.05 to 0.33)	0.751
Northern Mariana Islands	18.78 (16.39 to 21.35)	193.17 (168.6 to 219.66)	-0.49 (-0.65 to -0.33)	2.11 (1.91 to 2.3)	0.772
Palestine	1895.4 (1689.08 to 2110.21)	440.52 (392.57 to 490.45)	1.12 (1.03 to 1.21)	0.57 (0.28 to 0.86)	0.631
Algeria	7205.1 (6237.32 to 8363.51)	118.63 (102.7 to 137.71)	1.02 (0.96 to 1.09)	0.09 (-0.02 to 0.19)	0.660
Bangladesh	52460.99 (45869.31 to 58711.92)	224.52 (196.31 to 251.27)	0.93 (0.88 to 0.98)	1.39 (0.83 to 1.96)	0.492
China	491497.84 (418646.71 to 578308.73)	129.7 (110.47 to 152.61)	1.24 (0.97 to 1.52)	-0.21 (-0.68 to 0.26)	0.722
Bolivia	8100.22 (7284.75 to 8924.9)	524.13 (471.36 to 577.49)	1.91 (1.84 to 1.97)	-0.62 (-0.87 to -0.37)	0.599
Portugal	10205.01 (9241.72 to 11128.94)	262.3 (237.54 to 286.04)	2.44 (2.16 to 2.72)	0.3 (0.02 to 0.58)	0.744
Andorra	73.52 (65.14 to 82.8)	277.82 (246.16 to 312.91)	0.33 (0.25 to 0.41)	0.09 (-0.3 to 0.48)	0.869
Monaco	37.33 (33.56 to 41.43)	238.59 (214.53 to 264.83)	0.36 (0.3 to 0.43)	0.17 (0.09 to 0.25)	0.908
Puerto Rico	2313.21 (2077.94 to 2550.58)	198.16 (178 to 218.49)	2.06 (1.84 to 2.27)	0.11 (-0.16 to 0.38)	0.826
Albania	949.14 (830.22 to 1076.35)	120.71 (105.59 to 136.89)	0.28 (0.19 to 0.37)	0.64 (0.53 to 0.76)	0.707

Angola	1287.22 (1101.43 to 1493.8)	66.57 (56.96 to 77.25)	0.9 (0.82 to 0.98)	0.38 (-0.07 to 0.82)	0.454
Armenia	1308.26 (1174.69 to 1454.79)	166.31 (149.33 to 184.94)	0.89 (0.69 to 1.08)	0.53 (0.49 to 0.57)	0.702
Austria	4823.35 (4333.5 to 5332.92)	163.22 (146.64 to 180.46)	0.31 (0.02 to 0.59)	-0.02 (-0.28 to 0.24)	0.854
Azerbaijan	2097.11 (1819.53 to 2392.23)	109.46 (94.97 to 124.87)	0.36 (0.12 to 0.61)	0.1 (0 to 0.19)	0.695
Belarus	1924.35 (1669.7 to 2191.1)	66.9 (58.04 to 76.17)	-3.49 (-3.67 to -3.31)	0.29 (0.25 to 0.33)	0.784
Benin	323.52 (276.65 to 377.53)	39.12 (33.45 to 45.65)	-0.6 (-0.71 to -0.49)	0.88 (0.39 to 1.37)	0.373
Botswana	366.82 (320.82 to 416.58)	151.31 (132.34 to 171.84)	0.42 (0.34 to 0.5)	0.44 (0.2 to 0.69)	0.643
Bulgaria	1330.16 (1153.5 to 1538.13)	55.96 (48.53 to 64.71)	-0.99 (-1.04 to -0.93)	0.27 (0.12 to 0.42)	0.768
Burundi	438.12 (368.67 to 511.84)	54.73 (46.05 to 63.94)	-0.06 (-0.1 to -0.03)	0.13 (-0.26 to 0.52)	0.289
Cabo Verde	28 (23.46 to 33.14)	36.53 (30.6 to 43.24)	1.01 (0.96 to 1.05)	1.23 (0.93 to 1.54)	0.534
Cameroon	824.42 (708.72 to 970.62)	40.5 (34.82 to 47.68)	-0.78 (-0.96 to -0.6)	0.62 (0.3 to 0.94)	0.480
Chad	351.75 (299.08 to 412.9)	37.88 (32.21 to 44.47)	-0.27 (-0.4 to -0.13)	0 (-0.2 to 0.2)	0.240
Chile	33613.47 (30682.26 to 36847.28)	750.69 (685.23 to 822.91)	2.43 (2.28 to 2.57)	0.31 (0.19 to 0.44)	0.772
Colombia	12069.42 (10746.52 to 13482.47)	126.2 (112.37 to 140.98)	1.91 (1.82 to 2)	0.4 (0.4 to 0.4)	0.655
Costa Rica	3207.25 (2920.51 to 3506.76)	334.97 (305.03 to 366.26)	1.04 (0.9 to 1.18)	0.6 (0.53 to 0.66)	0.700
Côte d'Ivoire	748.71 (644.24 to 868.91)	41.75 (35.93 to 48.46)	-0.66 (-0.87 to -0.45)	1.04 (0.46 to 1.61)	0.426
Croatia	1107.34 (981.01 to 1245.83)	74.25 (65.78 to 83.54)	0.41 (-0.07 to 0.9)	0.18 (0.15 to 0.22)	0.798
Cuba	1415.35 (1207.74 to 1653.25)	41.42 (35.34 to 48.38)	0.41 (0.31 to 0.5)	-0.11 (-0.16 to -0.07)	0.669
Cyprus	1261.16 (1128.49 to 1407.51)	360.85 (322.89 to 402.72)	0.03 (-0.19 to 0.25)	0.7 (0.45 to 0.94)	0.836
Djibouti	63.84 (54.11 to 74.25)	61.56 (52.17 to 71.6)	0.26 (0.14 to 0.38)	1.21 (0.67 to 1.74)	0.488
Ecuador	10507.99 (9530.54 to 11404.38)	379.69 (344.37 to 412.08)	3.19 (2.94 to 3.44)	-0.61 (-0.62 to -0.59)	0.661
El Salvador	2253.58 (2024.93 to 2480.7)	220.38 (198.02 to 242.59)	2.68 (2.54 to 2.83)	0.18 (0.14 to 0.22)	0.564
Equatorial Guinea	69.6 (59.72 to 80.66)	87.99 (75.5 to 101.98)	2.96 (2.65 to 3.27)	-0.7 (-0.73 to -0.66)	0.658
Estonia	406.18 (366.74 to 455.2)	92.85 (83.83 to 104.05)	1.15 (1.06 to 1.23)	0.23 (0.07 to 0.38)	0.845
Fiji	83.42 (72.31 to 96.5)	61.01 (52.88 to 70.57)	0.07 (0.04 to 0.09)	0.56 (0.09 to 1.02)	0.675
Finland	6083.07 (5464.65 to 6725.92)	302.24 (271.51 to 334.18)	1.58 (1.32 to 1.84)	0.84 (0.54 to 1.13)	0.860
Ghana	1796.47 (1563.44 to 2036.38)	65.87 (57.33 to 74.67)	-0.1 (-0.33 to 0.13)	0.86 (0.51 to 1.21)	0.565
Guatemala	3376.53 (3075.02 to 3693.89)	184.43 (167.96 to 201.77)	1.24 (1.11 to 1.36)	0.51 (0.5 to 0.52)	0.540
Guinea	358.24 (308.95 to 413.33)	39.21 (33.82 to 45.24)	-0.72 (-0.88 to -0.55)	0.96 (0.74 to 1.17)	0.336
Guinea-Bissau	41.85 (35.65 to 49.3)	36.43 (31.04 to 42.92)	-0.78 (-0.93 to -0.64)	0.36 (-0.06 to 0.78)	0.353
Guyana	93.6 (82.98 to 104.84)	83.11 (73.69 to 93.09)	1.11 (1.07 to 1.15)	2.47 (2 to 2.95)	0.651
Haiti	805.3 (692.32 to 921.21)	67.95 (58.42 to 77.73)	0.8 (0.72 to 0.88)	-0.55 (-0.74 to -0.35)	0.448
Honduras	2395.74 (2111.39 to 2727.02)	227.8 (200.77 to 259.3)	1.81 (1.75 to 1.87)	0.36 (0.36 to 0.37)	0.513

Iceland	192.97<br>(174.85 to 213.95)	198.15<br>(179.54 to 219.69)	1.29<br>(1.1 to 1.48)	0.43<br>(0.26 to 0.6)	0.876
India	446277.01<br>(374669.13 to 524069.86)	221.98<br>(186.36 to 260.67)	0.4<br>(0.36 to 0.45)	-0.26<br>(-0.75 to 0.22)	0.575
Indonesia	29047.95<br>(24314.13 to 34544.79)	69.36<br>(58.06 to 82.49)	0.63<br>(0.54 to 0.73)	-0.09<br>(-0.25 to 0.07)	0.657
Iraq	3958.3<br>(3423.33 to 4571.81)	101.88<br>(88.11 to 117.67)	1.52<br>(1.38 to 1.67)	0.75<br>(0.74 to 0.75)	0.663
Italy	59070.25<br>(50821.03 to 68148.83)	260.03<br>(223.72 to 299.99)	2.05<br>(1.41 to 2.7)	-0.33<br>(-0.4 to -0.26)	0.806
Kazakhstan	3659.13<br>(3236.65 to 4117.17)	115.3<br>(101.99 to 129.74)	1.95<br>(1.49 to 2.4)	0.24<br>(0.19 to 0.29)	0.725
Kenya	2303.73<br>(1951.13 to 2744.68)	61.58<br>(52.16 to 73.37)	-0.31<br>(-0.41 to -0.21)	0<br>(-0.08 to 0.08)	0.524
Kiribati	9.42<br>(8.27 to 10.74)	74.88<br>(65.72 to 85.39)	-0.05<br>(-0.08 to -0.02)	0.49<br>(0.22 to 0.77)	0.527
South Korea	91425.88<br>(82274.56 to 101502.05)	546.68<br>(491.96 to 606.93)	2.94<br>(2.64 to 3.24)	1.24<br>(0.96 to 1.52)	0.887
Latvia	476.01<br>(419.62 to 538.87)	72.34<br>(63.77 to 81.89)	-1<br>(-1.22 to -0.78)	0.43<br>(0.33 to 0.53)	0.831
Liberia	128.16<br>(107.87 to 153.14)	38.82<br>(32.68 to 46.39)	-0.24<br>(-0.35 to -0.13)	0.15<br>(0.13 to 0.17)	0.352
Lithuania	523.24<br>(455.29 to 594.05)	54.07<br>(47.05 to 61.38)	-0.12<br>(-0.38 to 0.14)	0.57<br>(0.4 to 0.73)	0.856
Madagascar	1219.73<br>(1039.42 to 1412.96)	66.05<br>(56.29 to 76.51)	0.21<br>(0.15 to 0.26)	0.6<br>(0.56 to 0.65)	0.400
Malawi	593.25<br>(500.81 to 693.17)	50.74<br>(42.84 to 59.29)	0.39<br>(0.35 to 0.43)	0.56<br>(0.23 to 0.89)	0.385
Maldives	294.95<br>(262.87 to 329.18)	542.22<br>(483.25 to 605.14)	1.54<br>(1.36 to 1.73)	0.59<br>(0.47 to 0.71)	0.651
Mali	773.68<br>(671.32 to 881.27)	53.45<br>(46.38 to 60.89)	0.09<br>(0.03 to 0.14)	0.52<br>(0.18 to 0.85)	0.269
Malta	662.76<br>(601.02 to 725.69)	419.99<br>(380.87 to 459.87)	1.79<br>(1.65 to 1.93)	1.92<br>(1.67 to 2.16)	0.802
Mauritius	1734.36<br>(1556.97 to 1921.22)	512.62<br>(460.19 to 567.85)	2.19<br>(2.07 to 2.31)	1.43<br>(1.05 to 1.8)	0.718
Moldova	486.73<br>(409.28 to 568.7)	46.1<br>(38.76 to 53.86)	-0.25<br>(-0.31 to -0.18)	0.44<br>(0.43 to 0.44)	0.732
Mozambique	781.56<br>(663.68 to 920.22)	43.68<br>(37.1 to 51.43)	0.4<br>(0.32 to 0.48)	0.58<br>(0.25 to 0.91)	0.326
Namibia	315.82<br>(277.8 to 356.35)	139.77<br>(122.95 to 157.71)	0.28<br>(0.18 to 0.38)	-0.03<br>(-0.44 to 0.37)	0.618
Nauru	1.38<br>(1.22 to 1.55)	149.81<br>(132.23 to 168.03)	-0.03<br>(-0.32 to 0.25)	1.41<br>(1.07 to 1.74)	0.625
Nicaragua	1216.69<br>(1084 to 1358.09)	149.46<br>(133.16 to 166.83)	2.2<br>(2.03 to 2.36)	-0.11<br>(-0.43 to 0.2)	0.524
Niue	0.61<br>(0.54 to 0.68)	154.94<br>(138.45 to 173.75)	0.26<br>(0.18 to 0.34)	0.34<br>(-0.04 to 0.71)	0.726
Palau	6.53<br>(5.78 to 7.37)	155.93<br>(138.14 to 175.91)	-0.23<br>(-0.29 to -0.16)	0.41<br>(0.28 to 0.55)	0.754
Panama	1744.89<br>(1568.79 to 1914.26)	234.67<br>(210.98 to 257.44)	2.65<br>(2.55 to 2.75)	0.75<br>(0.48 to 1.03)	0.709
Paraguay	740.13<br>(651.67 to 834.67)	74.75<br>(65.82 to 84.3)	1.48<br>(1.42 to 1.54)	0.46<br>(0.37 to 0.55)	0.636
Peru	45990.19<br>(42300.1 to 49650.03)	822.22<br>(756.24 to 887.65)	2.83<br>(2.65 to 3.01)	-1.1<br>(-1.21 to -0.99)	0.662
Poland	15370.08<br>(13442.71 to 17735.92)	126.86<br>(110.96 to 146.39)	0.22<br>(0.09 to 0.36)	-0.33<br>(-0.92 to 0.27)	0.812
Rwanda	675.21<br>(569.53 to 790.96)	63.85<br>(53.86 to 74.8)	0.7<br>(0.54 to 0.86)	1.42<br>(1.06 to 1.79)	0.436
San Marino	9.83<br>(8.58 to 11.34)	83.12<br>(72.57 to 95.87)	-0.24<br>(-0.42 to -0.05)	-0.35<br>(-0.53 to -0.17)	0.888
Senegal	565.42<br>(486.19 to 650.45)	43.79<br>(37.66 to 50.38)	-0.44<br>(-0.57 to -0.32)	0.86<br>(0.36 to 1.36)	0.408
Serbia	2072.33<br>(1833.33 to 2343.41)	73.87<br>(65.35 to 83.54)	0.81<br>(0.6 to 1.03)	0.39<br>(0.15 to 0.63)	0.792

Seychelles	14.95 (12.66 to 17.57)	72.07 (61.01 to 84.69)	0.45 (0.3 to 0.6)	0.69 (0.68 to 0.71)	0.730
Sierra Leone	220.06 (186.62 to 257.88)	36.43 (30.89 to 42.69)	-0.72 (-0.95 to -0.49)	0.38 (0.01 to 0.75)	0.359
Singapore	3816.02 (3412.55 to 4278.14)	251.25 (224.68 to 281.67)	1.56 (1.45 to 1.66)	1.14 (1.07 to 1.21)	0.856
Slovenia	1035.97 (914.37 to 1178.81)	141.49 (124.88 to 161)	2.48 (1.97 to 3)	0.14 (-0.07 to 0.35)	0.842
South Africa	14372.26 (12137.95 to 16903.42)	182.19 (153.87 to 214.28)	-0.58 (-0.76 to -0.4)	-0.12 (-0.48 to 0.25)	0.680
South Sudan	276.49 (237.24 to 322.32)	44.4 (38.1 to 51.76)	-0.78 (-0.85 to -0.71)	-1.1 (-1.28 to -0.93)	0.278
Sudan	3032.12 (2621.75 to 3504.88)	97.38 (84.2 to 112.56)	1.07 (1.01 to 1.13)	0.47 (0.14 to 0.8)	0.542
Suriname	99.05 (87.85 to 110.33)	88.67 (78.64 to 98.77)	1.35 (1.23 to 1.46)	-0.45 (-0.54 to -0.36)	0.634
Tajikistan	2606.47 (2316.54 to 2917.74)	253.76 (225.53 to 284.06)	0.27 (0.07 to 0.46)	0.34 (0.29 to 0.4)	0.542
Republic of the Congo	314.57 (271.62 to 363.41)	72.39 (62.51 to 83.63)	0.58 (0.51 to 0.65)	0.2 (0.17 to 0.23)	0.583
Gambia	61.73 (53.12 to 71.13)	39.92 (34.35 to 46)	-0.67 (-0.76 to -0.58)	0.45 (0.15 to 0.75)	0.410
Marshall Islands	7.18 (6.35 to 8.11)	122.61 (108.3 to 138.5)	-0.09 (-0.2 to 0.01)	0.8 (0.56 to 1.04)	0.574
Niger	496.31 (423.47 to 582.29)	35.65 (30.42 to 41.82)	-0.79 (-0.94 to -0.65)	0.31 (0.02 to 0.6)	0.168
Philippines	4149.88 (3386.96 to 5041.1)	29.76 (24.29 to 36.15)	-1.3 (-1.37 to -1.23)	-0.01 (-0.22 to 0.19)	0.651
Myanmar	9988.53 (8595.47 to 11533.27)	118.32 (101.82 to 136.62)	2.1 (1.95 to 2.24)	1.48 (1.28 to 1.68)	0.534
Trinidad and Tobago	449.48 (403.64 to 499.42)	129.04 (115.88 to 143.37)	1.47 (1.23 to 1.72)	0.22 (0.12 to 0.31)	0.769
Tunisia	2932.07 (2527.67 to 3391.7)	125.46 (108.15 to 145.12)	1.09 (1.01 to 1.18)	0.64 (0.48 to 0.81)	0.682
Turkey	26547.6 (23349.62 to 29775.03)	160.69 (141.33 to 180.22)	2.03 (1.85 to 2.22)	0.67 (0.61 to 0.73)	0.713
Uganda	1513.99 (1289.12 to 1753.6)	63.84 (54.36 to 73.94)	0.54 (0.46 to 0.62)	0.77 (0.6 to 0.94)	0.423
Uzbekistan	6263.57 (5597.12 to 6951.47)	134.74 (120.4 to 149.54)	-1.56 (-1.78 to -1.35)	0.47 (0.35 to 0.6)	0.663
Vanuatu	29.36 (26.12 to 33.3)	102.4 (91.07 to 116.13)	-0.18 (-0.21 to -0.15)	0.37 (0.07 to 0.68)	0.473
Yemen	1925.12 (1651.02 to 2228.39)	85.6 (73.41 to 99.09)	0.85 (0.7 to 1.01)	-0.74 (-0.87 to -0.61)	0.450
Zambia	656.07 (559.62 to 765.36)	60.74 (51.81 to 70.86)	0.79 (0.66 to 0.91)	0.36 (0.11 to 0.61)	0.506
Zimbabwe	731.22 (625.26 to 854.96)	66.04 (56.47 to 77.21)	-0.82 (-0.93 to -0.7)	0.33 (0 to 0.65)	0.474
Romania	10428.78 (9238.48 to 11696.4)	173.66 (153.84 to 194.77)	-0.83 (-1 to -0.65)	0.68 (0.59 to 0.78)	0.768
Russia	18069.32 (14972.71 to 21412.23)	42.51 (35.23 to 50.38)	-1.74 (-1.83 to -1.65)	0 (-0.2 to 0.2)	0.809
Saint Kitts and Nevis	17.6 (15.58 to 19.69)	136.69 (120.93 to 152.9)	1.41 (1.26 to 1.56)	0.61 (0.6 to 0.63)	0.755
Saint Lucia	52.57 (47.51 to 57.66)	124.93 (112.9 to 137.02)	1.47 (1.38 to 1.57)	-0.41 (-0.41 to -0.41)	0.673
Saint Vincent and the Grenadines	17.02 (15 to 19.14)	66.93 (58.98 to 75.28)	1.65 (1.54 to 1.76)	0.09 (-0.14 to 0.31)	0.637
Slovakia	1805.2 (1608.37 to 2023.6)	110.05 (98.05 to 123.37)	1.14 (1.06 to 1.23)	0.12 (-0.02 to 0.27)	0.811
Vietnam	15782.94 (13367.94 to 18453.83)	90.35 (76.53 to 105.64)	1.92 (1.89 to 1.96)	1.57 (1.51 to 1.64)	0.628
Solomon Islands	49.88 (43.87 to 56.91)	91.94 (80.88 to 104.9)	-0.03 (-0.08 to 0.02)	0.29 (0.13 to 0.46)	0.429
Eritrea	222.97 (190.61 to 258.92)	50.61 (43.26 to 58.76)	0.27 (0.17 to 0.37)	0.79 (0.77 to 0.81)	0.404

Israel	3285.24 (2954.28 to 3650.09)	164.38 (147.82 to 182.64)	0.74 (0.71 to 0.77)	0.62 (0.2 to 1.03)	0.809
Kuwait	975.33 (874.55 to 1089.63)	209.2 (187.58 to 233.71)	0.89 (0.58 to 1.2)	-1.15 (-1.48 to -0.81)	0.847
Libya	864.71 (744.53 to 1004.62)	103.39 (89.02 to 120.12)	0.35 (0.15 to 0.55)	-1.8 (-2.44 to -1.17)	0.726
Qatar	181.2 (155.76 to 210.08)	118.29 (101.68 to 137.14)	0.13 (-0.07 to 0.34)	1.48 (1.2 to 1.76)	0.847
Oman	448.25 (386.49 to 516.31)	143.51 (123.73 to 165.29)	2.03 (1.89 to 2.17)	-0.44 (-0.7 to -0.17)	0.773
Switzerland	6160.86 (5573.24 to 6784.69)	210.21 (190.16 to 231.49)	0.61 (0.33 to 0.9)	0.08 (-0.19 to 0.34)	0.933
Syria	3283.57 (2858.9 to 3777.39)	138.98 (121 to 159.88)	1.21 (1.06 to 1.36)	0.14 (0.08 to 0.21)	0.623
Taiwan	11396.22 (10032.93 to 12876.68)	151.35 (133.24 to 171.01)	2.85 (2.67 to 3.03)	1.03 (0.66 to 1.41)	0.875
Togo	237.26 (201.58 to 279.45)	37.98 (32.27 to 44.73)	-0.8 (-0.99 to -0.61)	0.92 (0.65 to 1.19)	0.409
Tokelau	0.34 (0.3 to 0.38)	134.84 (119.98 to 149.95)	0.42 (0.4 to 0.44)	0.28 (-0.14 to 0.71)	0.686
Turkmenistan	359.63 (302.88 to 425.44)	50.82 (42.8 to 60.12)	-1.58 (-1.83 to -1.32)	0.71 (0.63 to 0.79)	0.682
Tuvalu	2.48 (2.2 to 2.79)	136.25 (120.9 to 152.86)	0.37 (0.31 to 0.44)	0.75 (0.57 to 0.93)	0.577
Ukraine	6347.09 (5298.26 to 7487.67)	46.75 (39.02 to 55.15)	-5.03 (-5.28 to -4.77)	-0.59 (-1.16 to -0.02)	0.761
Comoros	45.83 (39.43 to 53.45)	56.7 (48.78 to 66.12)	-0.03 (-0.08 to 0.01)	0.28 (0.15 to 0.41)	0.476
UAE	997.94 (850.35 to 1169.51)	138.97 (118.42 to 162.87)	0.25 (0.2 to 0.31)	-0.06 (-0.23 to 0.11)	0.849
UK	96015.63 (82706.13 to 109671.33)	456.56 (393.27 to 521.49)	1.2 (1.14 to 1.27)	-0.11 (-0.28 to 0.07)	0.859
Mexico	52283.06 (44540.7 to 60617.71)	242.61 (206.68 to 281.28)	0.1 (-0.02 to 0.22)	-0.24 (-0.26 to -0.22)	0.665
Tanzania	2213.46 (1906.16 to 2555.81)	53.7 (46.24 to 62)	0.72 (0.65 to 0.79)	0.76 (0.66 to 0.85)	0.447
USA	587825.18 (504169.27 to 678530.05)	586.37 (502.92 to 676.85)	0.19 (0.03 to 0.36)	1.74 (0.82 to 2.67)	0.862
US Virgin Islands	61.84 (55.68 to 68.3)	192.1 (172.99 to 212.19)	1.92 (1.78 to 2.06)	-0.12 (-0.18 to -0.05)	0.822

Table S6 *Net Drift* in the Prevalence of CRDs, COPD, Asthma, and ILD & PS in the Global, 5 SDI Regions, and 21 GBD Regions (1990-2021)

Cause<br>Location	CRDs<br>Net Drift (%/year)	COPD<br>Net Drift (%/year)	Asthma<br>Net Drift (%/year)	ILD&PS<br>Net Drift (%/year)
Global	-0.47<br>(-0.51 to -0.43)	0.12<br>(0.1 to 0.13)	-2.2<br>(-2.29 to -2.11)	0.98<br>(0.89 to 1.07)
High SDI	-0.59<br>(-0.66 to -0.52)	0.07<br>(0.05 to 0.1)	-1.98<br>(-2.08 to -1.88)	1.16<br>(1.06 to 1.26)
High-middle SDI	-0.74<br>(-0.79 to -0.68)	0<br>(-0.05 to 0.04)	-3.14<br>(-3.29 to -2.98)	1.56<br>(1.36 to 1.77)
Middle SDI	-0.2<br>(-0.26 to -0.14)	0.28<br>(0.23 to 0.32)	-2.19<br>(-2.32 to -2.06)	1.47<br>(1.36 to 1.57)
Low-middle SDI	0.05<br>(0.02 to 0.07)	0.32<br>(0.31 to 0.33)	-0.88<br>(-0.98 to -0.77)	0.77<br>(0.7 to 0.83)
Low SDI	0.05<br>(-0.03 to 0.12)	0.44<br>(0.37 to 0.51)	-0.93<br>(-0.99 to -0.87)	0.36<br>(0.18 to 0.54)
Andean Latin America	0.35<br>(0.33 to 0.38)	0.57<br>(0.55 to 0.59)	-1.58<br>(-1.67 to -1.5)	3.03<br>(2.93 to 3.12)
Australasia	-1.25<br>(-1.32 to -1.18)	-0.77<br>(-0.8 to -0.74)	-2.17<br>(-2.38 to -1.97)	2.26<br>(2.11 to 2.42)
Caribbean	0.2<br>(0.17 to 0.23)	0.72<br>(0.7 to 0.74)	-1.41<br>(-1.48 to -1.35)	1.67<br>(1.34 to 2)
Central Asia	-0.12<br>(-0.18 to -0.07)	0.31<br>(0.27 to 0.34)	-1.53<br>(-1.6 to -1.46)	0.76<br>(0.57 to 0.95)
Central Europe	-0.79<br>(-0.86 to -0.72)	0.37<br>(0.34 to 0.39)	-3.35<br>(-3.47 to -3.24)	0.72<br>(0.52 to 0.91)
Central Latin America	-0.26<br>(-0.32 to -0.21)	0.37<br>(0.35 to 0.4)	-3.96<br>(-4.13 to -3.8)	0.97<br>(0.8 to 1.15)
Central Sub-Saharan Africa	0.19<br>(0.15 to 0.24)	0.57<br>(0.53 to 0.61)	-0.72<br>(-0.8 to -0.65)	0.4<br>(-0.38 to 1.18)
East Asia	-0.42<br>(-0.56 to -0.28)	0.04<br>(-0.1 to 0.17)	-2.95<br>(-3.19 to -2.71)	2.1<br>(1.52 to 2.69)
Eastern Europe	-1.52<br>(-1.58 to -1.46)	-0.55<br>(-0.64 to -0.45)	-5.37<br>(-5.55 to -5.2)	-3.01<br>(-3.17 to -2.86)
Eastern Sub-Saharan Africa	-0.18<br>(-0.23 to -0.13)	0.27<br>(0.24 to 0.3)	-1.02<br>(-1.07 to -0.96)	0.27<br>(-0.21 to 0.75)
High-income Asia Pacific	-2.38<br>(-2.51 to -2.25)	-0.3<br>(-0.35 to -0.24)	-5.73<br>(-6.04 to -5.42)	0.78<br>(0.65 to 0.91)
High-income North America	0.73<br>(0.61 to 0.85)	0.48<br>(0.41 to 0.55)	1.61<br>(1.45 to 1.78)	0.85<br>(0.76 to 0.95)
North Africa and Middle East	0.17<br>(0.12 to 0.22)	0.85<br>(0.84 to 0.87)	-1.04<br>(-1.17 to -0.92)	1.64<br>(1.5 to 1.77)
Oceania	-0.66<br>(-0.74 to -0.59)	-0.23<br>(-0.31 to -0.14)	-1.82<br>(-1.96 to -1.67)	0.52<br>(-1.51 to 2.6)
South Asia	0.03<br>(0 to 0.05)	0.27<br>(0.26 to 0.29)	-0.88<br>(-1.04 to -0.71)	0.53<br>(0.45 to 0.6)
Southeast Asia	-0.33<br>(-0.36 to -0.29)	0.13<br>(0.11 to 0.15)	-1.38<br>(-1.45 to -1.31)	1.25<br>(1.1 to 1.39)
Southern Latin America	-0.61<br>(-0.65 to -0.58)	0.03<br>(-0.01 to 0.06)	-1.22<br>(-1.26 to -1.17)	2.13<br>(2.03 to 2.23)

Southern Sub-Saharan Africa	-0.18<br>(-0.26 to -0.11)	0.1<br>(0.05 to 0.16)	-1.42<br>(-1.51 to -1.34)	-0.52<br>(-0.74 to -0.29)
Tropical Latin America	-0.51<br>(-0.55 to -0.47)	-0.07<br>(-0.1 to -0.05)	-2.58<br>(-2.8 to -2.37)	-0.13<br>(-0.28 to 0.02)
Western Europe	-0.88<br>(-0.96 to -0.8)	0.02<br>(0 to 0.04)	-3.07<br>(-3.27 to -2.86)	1.68<br>(1.63 to 1.72)
Western Sub-Saharan Africa	0.16<br>(0.13 to 0.19)	0.75<br>(0.72 to 0.78)	-0.74<br>(-0.8 to -0.69)	-0.15<br>(-0.55 to 0.26)

Table S7 APC Model Analysis Results of CRDs Prevalence Among Adults Aged 55 and Above in the Global, 5 SDI Regions, and 21 GBD Regions

	CRDs

Location	Age	Local drift (%/year)
Global	55 to 59	-1.2<br>(-1.26 to -1.15)
Global	60 to 64	-1.08<br>(-1.12 to -1.04)
Global	65 to 69	-0.93<br>(-0.97 to -0.9)
Global	70 to 74	-0.75<br>(-0.79 to -0.72)
Global	75 to 79	-0.56<br>(-0.6 to -0.52)
Global	80 to 84	-0.33<br>(-0.38 to -0.27)
Global	85 to 89	-0.04<br>(-0.11 to 0.04)
Global	90 to 94	0.26<br>(0.13 to 0.38)
Global	95+	0.55<br>(0.27 to 0.84)
High SDI	55 to 59	-1<br>(-1.11 to -0.88)
High SDI	60 to 64	-0.96<br>(-1.05 to -0.88)
High SDI	65 to 69	-0.96<br>(-1.04 to -0.89)
High SDI	70 to 74	-0.93<br>(-1.01 to -0.85)
High SDI	75 to 79	-0.85<br>(-0.93 to -0.76)
High SDI	80 to 84	-0.6<br>(-0.7 to -0.51)
High SDI	85 to 89	-0.25<br>(-0.38 to -0.12)
High SDI	90 to 94	0.12<br>(-0.09 to 0.34)
High SDI	95+	0.51<br>(0.06 to 0.96)
High-middle SDI	55 to 59	-2<br>(-2.07 to -1.93)
High-middle SDI	60 to 64	-1.81<br>(-1.87 to -1.76)
High-middle SDI	65 to 69	-1.5<br>(-1.55 to -1.45)
High-middle SDI	70 to 74	-1.14<br>(-1.19 to -1.09)
High-middle SDI	75 to 79	-0.76<br>(-0.82 to -0.71)
High-middle SDI	80 to 84	-0.38<br>(-0.45 to -0.31)
High-middle SDI	85 to 89	0.02<br>(-0.08 to 0.12)
High-middle SDI	90 to 94	0.36<br>(0.17 to 0.54)
High-middle SDI	95+	0.68<br>(0.23 to 1.12)
Middle SDI	55 to 59	-1.05<br>(-1.11 to -0.99)
Middle SDI	60 to 64	-0.93<br>(-0.98 to -0.88)
Middle SDI	65 to 69	-0.76<br>(-0.8 to -0.72)
Middle SDI	70 to 74	-0.53<br>(-0.57 to -0.48)
Middle SDI	75 to 79	-0.26<br>(-0.32 to -0.21)

Middle SDI	80 to 84	0.02<br>(-0.05 to 0.09)
Middle SDI	85 to 89	0.32<br>(0.22 to 0.43)
Middle SDI	90 to 94	0.63<br>(0.43 to 0.83)
Middle SDI	95+	0.93<br>(0.46 to 1.4)
Low-middle SDI	55 to 59	-0.55<br>(-0.57 to -0.52)
Low-middle SDI	60 to 64	-0.46<br>(-0.48 to -0.45)
Low-middle SDI	65 to 69	-0.31<br>(-0.32 to -0.29)
Low-middle SDI	70 to 74	-0.11<br>(-0.13 to -0.1)
Low-middle SDI	75 to 79	0.08<br>(0.06 to 0.1)
Low-middle SDI	80 to 84	0.24<br>(0.22 to 0.27)
Low-middle SDI	85 to 89	0.37<br>(0.33 to 0.41)
Low-middle SDI	90 to 94	0.5<br>(0.42 to 0.57)
Low-middle SDI	95+	0.63<br>(0.46 to 0.79)
Low SDI	55 to 59	-0.62<br>(-0.68 to -0.57)
Low SDI	60 to 64	-0.47<br>(-0.52 to -0.43)
Low SDI	65 to 69	-0.26<br>(-0.3 to -0.22)
Low SDI	70 to 74	-0.05<br>(-0.1 to -0.01)
Low SDI	75 to 79	0.11<br>(0.06 to 0.17)
Low SDI	80 to 84	0.22<br>(0.14 to 0.3)
Low SDI	85 to 89	0.27<br>(0.15 to 0.4)
Low SDI	90 to 94	0.44<br>(0.19 to 0.69)
Low SDI	95+	0.69<br>(0.1 to 1.29)
Andean Latin America	55 to 59	-0.46<br>(-0.5 to -0.42)
Andean Latin America	60 to 64	-0.33<br>(-0.36 to -0.29)
Andean Latin America	65 to 69	-0.15<br>(-0.18 to -0.12)
Andean Latin America	70 to 74	0.06<br>(0.03 to 0.09)
Andean Latin America	75 to 79	0.29<br>(0.26 to 0.32)
Andean Latin America	80 to 84	0.55<br>(0.51 to 0.58)
Andean Latin America	85 to 89	0.82<br>(0.78 to 0.87)
Andean Latin America	90 to 94	1.11<br>(1.03 to 1.19)
Andean Latin America	95+	1.39<br>(1.24 to 1.55)
Australasia	55 to 59	-2.29<br>(-2.39 to -2.18)
Australasia	60 to 64	-2.1<br>(-2.18 to -2.02)

Australasia	65 to 69	-1.85<br>(-1.93 to -1.78)
Australasia	70 to 74	-1.58<br>(-1.65 to -1.51)
Australasia	75 to 79	-1.3<br>(-1.37 to -1.22)
Australasia	80 to 84	-0.97<br>(-1.06 to -0.88)
Australasia	85 to 89	-0.63<br>(-0.76 to -0.5)
Australasia	90 to 94	-0.34<br>(-0.56 to -0.12)
Australasia	95+	-0.1<br>(-0.58 to 0.39)
Caribbean	55 to 59	-0.62<br>(-0.66 to -0.58)
Caribbean	60 to 64	-0.46<br>(-0.49 to -0.42)
Caribbean	65 to 69	-0.23<br>(-0.26 to -0.2)
Caribbean	70 to 74	0<br>(-0.03 to 0.03)
Caribbean	75 to 79	0.23<br>(0.2 to 0.27)
Caribbean	80 to 84	0.45<br>(0.4 to 0.49)
Caribbean	85 to 89	0.63<br>(0.57 to 0.69)
Caribbean	90 to 94	0.79<br>(0.69 to 0.89)
Caribbean	95+	0.96<br>(0.74 to 1.19)
Central Asia	55 to 59	-1.2<br>(-1.27 to -1.14)
Central Asia	60 to 64	-0.97<br>(-1.02 to -0.92)
Central Asia	65 to 69	-0.67<br>(-0.72 to -0.62)
Central Asia	70 to 74	-0.36<br>(-0.42 to -0.31)
Central Asia	75 to 79	-0.03<br>(-0.09 to 0.03)
Central Asia	80 to 84	0.24<br>(0.17 to 0.32)
Central Asia	85 to 89	0.48<br>(0.38 to 0.58)
Central Asia	90 to 94	0.61<br>(0.45 to 0.77)
Central Asia	95+	0.67<br>(0.32 to 1.03)
Central Europe	55 to 59	-1.68<br>(-1.77 to -1.59)
Central Europe	60 to 64	-1.64<br>(-1.7 to -1.58)
Central Europe	65 to 69	-1.54<br>(-1.6 to -1.49)
Central Europe	70 to 74	-1.32<br>(-1.38 to -1.26)
Central Europe	75 to 79	-1<br>(-1.06 to -0.93)
Central Europe	80 to 84	-0.55<br>(-0.63 to -0.46)
Central Europe	85 to 89	-0.1<br>(-0.22 to 0.02)
Central Europe	90 to 94	0.33<br>(0.1 to 0.55)

Central Europe	95+	0.74<br>(0.18 to 1.3)
Central Latin America	55 to 59	-1.25<br>(-1.33 to -1.17)
Central Latin America	60 to 64	-1.07<br>(-1.14 to -1.01)
Central Latin America	65 to 69	-0.83<br>(-0.89 to -0.78)
Central Latin America	70 to 74	-0.55<br>(-0.61 to -0.49)
Central Latin America	75 to 79	-0.26<br>(-0.32 to -0.2)
Central Latin America	80 to 84	0.02<br>(-0.05 to 0.1)
Central Latin America	85 to 89	0.3<br>(0.2 to 0.39)
Central Latin America	90 to 94	0.54<br>(0.38 to 0.7)
Central Latin America	95+	0.74<br>(0.4 to 1.09)
Central Sub-Saharan Africa	55 to 59	-0.54<br>(-0.56 to -0.52)
Central Sub-Saharan Africa	60 to 64	-0.38<br>(-0.4 to -0.37)
Central Sub-Saharan Africa	65 to 69	-0.22<br>(-0.24 to -0.2)
Central Sub-Saharan Africa	70 to 74	-0.03<br>(-0.05 to -0.01)
Central Sub-Saharan Africa	75 to 79	0.17<br>(0.14 to 0.2)
Central Sub-Saharan Africa	80 to 84	0.39<br>(0.35 to 0.43)
Central Sub-Saharan Africa	85 to 89	0.63<br>(0.55 to 0.7)
Central Sub-Saharan Africa	90 to 94	0.83<br>(0.68 to 0.98)
Central Sub-Saharan Africa	95+	0.95<br>(0.62 to 1.29)
East Asia	55 to 59	-1.42<br>(-1.53 to -1.32)
East Asia	60 to 64	-1.31<br>(-1.39 to -1.23)
East Asia	65 to 69	-1.12<br>(-1.19 to -1.05)
East Asia	70 to 74	-0.85<br>(-0.93 to -0.78)
East Asia	75 to 79	-0.53<br>(-0.61 to -0.45)
East Asia	80 to 84	-0.18<br>(-0.29 to -0.06)
East Asia	85 to 89	0.23<br>(0.03 to 0.42)
East Asia	90 to 94	0.65<br>(0.21 to 1.08)
East Asia	95+	1<br>(-0.29 to 2.3)
Eastern Europe	55 to 59	-2.77<br>(-2.84 to -2.69)
Eastern Europe	60 to 64	-2.51<br>(-2.57 to -2.46)
Eastern Europe	65 to 69	-2.2<br>(-2.25 to -2.15)
Eastern Europe	70 to 74	-1.85<br>(-1.9 to -1.79)
Eastern Europe	75 to 79	-1.47<br>(-1.53 to -1.41)

Eastern Europe	80 to 84	-1.13<br>(-1.2 to -1.06)
Eastern Europe	85 to 89	-0.83<br>(-0.93 to -0.73)
Eastern Europe	90 to 94	-0.59<br>(-0.77 to -0.41)
Eastern Europe	95+	-0.37<br>(-0.81 to 0.08)
Eastern Sub-Saharan Africa	55 to 59	-0.76<br>(-0.79 to -0.73)
Eastern Sub-Saharan Africa	60 to 64	-0.61<br>(-0.63 to -0.58)
Eastern Sub-Saharan Africa	65 to 69	-0.45<br>(-0.47 to -0.43)
Eastern Sub-Saharan Africa	70 to 74	-0.3<br>(-0.33 to -0.27)
Eastern Sub-Saharan Africa	75 to 79	-0.16<br>(-0.19 to -0.12)
Eastern Sub-Saharan Africa	80 to 84	-0.03<br>(-0.08 to 0.02)
Eastern Sub-Saharan Africa	85 to 89	0.09<br>(0.01 to 0.17)
Eastern Sub-Saharan Africa	90 to 94	0.2<br>(0.04 to 0.37)
Eastern Sub-Saharan Africa	95+	0.34<br>(-0.07 to 0.75)
High-income Asia Pacific	55 to 59	-3.05<br>(-3.34 to -2.76)
High-income Asia Pacific	60 to 64	-2.99<br>(-3.19 to -2.79)
High-income Asia Pacific	65 to 69	-2.98<br>(-3.15 to -2.82)
High-income Asia Pacific	70 to 74	-2.92<br>(-3.08 to -2.77)
High-income Asia Pacific	75 to 79	-2.74<br>(-2.89 to -2.58)
High-income Asia Pacific	80 to 84	-2.39<br>(-2.57 to -2.21)
High-income Asia Pacific	85 to 89	-1.86<br>(-2.09 to -1.62)
High-income Asia Pacific	90 to 94	-1.23<br>(-1.63 to -0.84)
High-income Asia Pacific	95+	-0.66<br>(-1.52 to 0.21)
High-income North America	55 to 59	0.8<br>(0.61 to 0.99)
High-income North America	60 to 64	0.76<br>(0.61 to 0.91)
High-income North America	65 to 69	0.69<br>(0.56 to 0.82)
High-income North America	70 to 74	0.62<br>(0.49 to 0.75)
High-income North America	75 to 79	0.6<br>(0.46 to 0.74)
High-income North America	80 to 84	0.65<br>(0.48 to 0.82)
High-income North America	85 to 89	0.75<br>(0.52 to 0.98)
High-income North America	90 to 94	0.86<br>(0.49 to 1.24)
High-income North America	95+	0.99<br>(0.23 to 1.76)
North Africa and Middle East	55 to 59	-0.99<br>(-1.05 to -0.94)
North Africa and Middle East	60 to 64	-0.79<br>(-0.83 to -0.74)

North Africa and Middle East	65 to 69	-0.48<br>(-0.52 to -0.44)
North Africa and Middle East	70 to 74	-0.13<br>(-0.18 to -0.09)
North Africa and Middle East	75 to 79	0.22<br>(0.17 to 0.28)
North Africa and Middle East	80 to 84	0.53<br>(0.45 to 0.6)
North Africa and Middle East	85 to 89	0.81<br>(0.7 to 0.91)
North Africa and Middle East	90 to 94	1.04<br>(0.87 to 1.22)
North Africa and Middle East	95+	1.31<br>(0.94 to 1.68)
Oceania	55 to 59	-0.94<br>(-0.99 to -0.89)
Oceania	60 to 64	-0.91<br>(-0.95 to -0.87)
Oceania	65 to 69	-0.89<br>(-0.93 to -0.85)
Oceania	70 to 74	-0.83<br>(-0.88 to -0.79)
Oceania	75 to 79	-0.74<br>(-0.8 to -0.69)
Oceania	80 to 84	-0.59<br>(-0.67 to -0.51)
Oceania	85 to 89	-0.41<br>(-0.54 to -0.28)
Oceania	90 to 94	-0.29<br>(-0.56 to -0.02)
Oceania	95+	-0.26<br>(-0.89 to 0.36)
South Asia	55 to 59	-0.47<br>(-0.49 to -0.44)
South Asia	60 to 64	-0.4<br>(-0.42 to -0.38)
South Asia	65 to 69	-0.28<br>(-0.3 to -0.26)
South Asia	70 to 74	-0.13<br>(-0.15 to -0.11)
South Asia	75 to 79	0.03<br>(0 to 0.05)
South Asia	80 to 84	0.18<br>(0.15 to 0.22)
South Asia	85 to 89	0.32<br>(0.27 to 0.37)
South Asia	90 to 94	0.44<br>(0.34 to 0.53)
South Asia	95+	0.53<br>(0.31 to 0.75)
Southeast Asia	55 to 59	-1.07<br>(-1.1 to -1.04)
Southeast Asia	60 to 64	-0.92<br>(-0.94 to -0.89)
Southeast Asia	65 to 69	-0.75<br>(-0.77 to -0.72)
Southeast Asia	70 to 74	-0.58<br>(-0.61 to -0.55)
Southeast Asia	75 to 79	-0.39<br>(-0.42 to -0.35)
Southeast Asia	80 to 84	-0.16<br>(-0.2 to -0.12)
Southeast Asia	85 to 89	0.09<br>(0.03 to 0.15)
Southeast Asia	90 to 94	0.34<br>(0.23 to 0.45)

Southeast Asia	95+	0.62<br>(0.38 to 0.85)
Southern Latin America	55 to 59	-1.46<br>(-1.5 to -1.41)
Southern Latin America	60 to 64	-1.39<br>(-1.43 to -1.35)
Southern Latin America	65 to 69	-1.22<br>(-1.26 to -1.19)
Southern Latin America	70 to 74	-0.97<br>(-1 to -0.94)
Southern Latin America	75 to 79	-0.65<br>(-0.69 to -0.61)
Southern Latin America	80 to 84	-0.32<br>(-0.37 to -0.28)
Southern Latin America	85 to 89	-0.03<br>(-0.09 to 0.04)
Southern Latin America	90 to 94	0.2<br>(0.09 to 0.31)
Southern Latin America	95+	0.41<br>(0.16 to 0.66)
Southern Sub-Saharan Africa	55 to 59	-0.79<br>(-0.88 to -0.71)
Southern Sub-Saharan Africa	60 to 64	-0.53<br>(-0.59 to -0.46)
Southern Sub-Saharan Africa	65 to 69	-0.3<br>(-0.36 to -0.23)
Southern Sub-Saharan Africa	70 to 74	-0.15<br>(-0.22 to -0.08)
Southern Sub-Saharan Africa	75 to 79	-0.05<br>(-0.13 to 0.03)
Southern Sub-Saharan Africa	80 to 84	0<br>(-0.1 to 0.1)
Southern Sub-Saharan Africa	85 to 89	-0.01<br>(-0.15 to 0.13)
Southern Sub-Saharan Africa	90 to 94	-0.01<br>(-0.24 to 0.22)
Southern Sub-Saharan Africa	95+	-0.02<br>(-0.53 to 0.49)
Tropical Latin America	55 to 59	-1.29<br>(-1.35 to -1.24)
Tropical Latin America	60 to 64	-1.11<br>(-1.15 to -1.06)
Tropical Latin America	65 to 69	-0.9<br>(-0.94 to -0.86)
Tropical Latin America	70 to 74	-0.67<br>(-0.71 to -0.63)
Tropical Latin America	75 to 79	-0.45<br>(-0.5 to -0.41)
Tropical Latin America	80 to 84	-0.25<br>(-0.31 to -0.2)
Tropical Latin America	85 to 89	-0.09<br>(-0.17 to -0.01)
Tropical Latin America	90 to 94	0.01<br>(-0.12 to 0.14)
Tropical Latin America	95+	0.07<br>(-0.22 to 0.36)
Western Europe	55 to 59	-1.89<br>(-2.03 to -1.74)
Western Europe	60 to 64	-1.77<br>(-1.88 to -1.66)
Western Europe	65 to 69	-1.58<br>(-1.68 to -1.49)
Western Europe	70 to 74	-1.34<br>(-1.43 to -1.24)
Western Europe	75 to 79	-1.06<br>(-1.16 to -0.96)

Western Europe	80 to 84	-0.63<br>(-0.75 to -0.52)
Western Europe	85 to 89	-0.16<br>(-0.31 to -0.02)
Western Europe	90 to 94	0.22<br>(-0.02 to 0.46)
Western Europe	95+	0.53<br>(0.01 to 1.04)
Western Sub-Saharan Africa	55 to 59	-0.34<br>(-0.36 to -0.31)
Western Sub-Saharan Africa	60 to 64	-0.28<br>(-0.3 to -0.26)
Western Sub-Saharan Africa	65 to 69	-0.23<br>(-0.24 to -0.21)
Western Sub-Saharan Africa	70 to 74	-0.12<br>(-0.14 to -0.1)
Western Sub-Saharan Africa	75 to 79	0.06<br>(0.03 to 0.08)
Western Sub-Saharan Africa	80 to 84	0.27<br>(0.23 to 0.3)
Western Sub-Saharan Africa	85 to 89	0.52<br>(0.47 to 0.58)
Western Sub-Saharan Africa	90 to 94	0.77<br>(0.67 to 0.87)
Western Sub-Saharan Africa	95+	0.97<br>(0.76 to 1.18)

CRDs<br>Location	Age	Prevalence rate (per 100,000 population)
Global	55 to 59	8193.59<br>(8113.21 to 8274.77)
Global	60 to 64	10026.05<br>(9929.99 to 10123.04)
Global	65 to 69	12740.85<br>(12596.45 to 12886.91)
Global	70 to 74	15823.35<br>(15639.2 to 16009.66)
Global	75 to 79	19005.03<br>(18775.49 to 19237.38)
Global	80 to 84	22317.02<br>(22030.05 to 22607.73)
Global	85 to 89	26283.7<br>(25903.21 to 26669.78)
Global	90 to 94	30485.43<br>(29927.6 to 31053.66)
Global	95+	35236.13<br>(34266.8 to 36232.89)
High SDI	55 to 59	11110.59<br>(10867.32 to 11359.31)
High SDI	60 to 64	12999.51<br>(12719.87 to 13285.3)
High SDI	65 to 69	15795.17<br>(15397.29 to 16203.33)
High SDI	70 to 74	18721.98<br>(18240.12 to 19216.56)
High SDI	75 to 79	21656.72<br>(21083.62 to 22245.4)
High SDI	80 to 84	24773.04<br>(24089.23 to 25476.27)
High SDI	85 to 89	28569.97<br>(27717.41 to 29448.76)
High SDI	90 to 94	32488.6<br>(31350.07 to 33668.48)
High SDI	95+	36624.71<br>(34873.7 to 38463.65)

High-middle SDI	55 to 59	6992.53 (6893.21 to 7093.28)
High-middle SDI	60 to 64	8369.96 (8253.67 to 8487.89)
High-middle SDI	65 to 69	10335.93 (10169 to 10505.6)
High-middle SDI	70 to 74	12611 (12403.54 to 12821.93)
High-middle SDI	75 to 79	14986.37 (14732.55 to 15244.57)
High-middle SDI	80 to 84	17575.09 (17260.84 to 17895.06)
High-middle SDI	85 to 89	20777.82 (20361.44 to 21202.71)
High-middle SDI	90 to 94	24258.09 (23637.27 to 24895.23)
High-middle SDI	95+	28640.59 (27469.9 to 29861.19)
Middle SDI	55 to 59	6810.57 (6734.35 to 6887.65)
Middle SDI	60 to 64	8647.38 (8553.34 to 8742.45)
Middle SDI	65 to 69	11523.84 (11373.82 to 11675.83)
Middle SDI	70 to 74	15086.88 (14884.51 to 15292)
Middle SDI	75 to 79	18817.77 (18554.46 to 19084.82)
Middle SDI	80 to 84	22479.97 (22140.65 to 22824.5)
Middle SDI	85 to 89	26458.52 (25993.26 to 26932.12)
Middle SDI	90 to 94	30362.54 (29637.98 to 31104.81)
Middle SDI	95+	35059.22 (33700.91 to 36472.27)
Low-middle SDI	55 to 59	8688.01 (8651.6 to 8724.58)
Low-middle SDI	60 to 64	11031.69 (10986.7 to 11076.86)
Low-middle SDI	65 to 69	14561.25 (14489.97 to 14632.88)
Low-middle SDI	70 to 74	18594.03 (18499.52 to 18689.03)
Low-middle SDI	75 to 79	22764.75 (22642.43 to 22887.73)
Low-middle SDI	80 to 84	26861.98 (26702.99 to 27021.91)
Low-middle SDI	85 to 89	31835.53 (31610.29 to 32062.37)
Low-middle SDI	90 to 94	37461.27 (37097.41 to 37828.69)
Low-middle SDI	95+	44258.96 (43585.73 to 44942.59)
Low SDI	55 to 59	8614.49 (8529.02 to 8700.83)
Low SDI	60 to 64	10736.4 (10631.81 to 10842.02)
Low SDI	65 to 69	13362.8 (13205.99 to 13521.47)
Low SDI	70 to 74	16019.53 (15822.25 to 16219.26)
Low SDI	75 to 79	18786.29 (18538.08 to 19037.83)
Low SDI	80 to 84	21520.57 (21197.52 to 21848.54)

Low SDI	85 to 89	24418.12 (23949.67 to 24895.74)
Low SDI	90 to 94	27324.97 (26515.49 to 28159.17)
Low SDI	95+	30449.03 (28759.3 to 32238.05)
Andean Latin America	55 to 59	4349.32 (4318.33 to 4380.53)
Andean Latin America	60 to 64	5826.57 (5786.66 to 5866.75)
Andean Latin America	65 to 69	7760.58 (7696.03 to 7825.68)
Andean Latin America	70 to 74	10483.16 (10393.32 to 10573.78)
Andean Latin America	75 to 79	14265.11 (14138.66 to 14392.7)
Andean Latin America	80 to 84	19262.13 (19082.81 to 19443.13)
Andean Latin America	85 to 89	25455.33 (25197.68 to 25715.61)
Andean Latin America	90 to 94	32116.46 (31735.06 to 32502.45)
Andean Latin America	95+	38539.63 (37932.72 to 39156.25)
Australasia	55 to 59	9646.63 (9451.71 to 9845.56)
Australasia	60 to 64	10132.65 (9930.15 to 10339.28)
Australasia	65 to 69	11106.46 (10846.93 to 11372.2)
Australasia	70 to 74	12400.54 (12103.93 to 12704.42)
Australasia	75 to 79	13758.37 (13418.9 to 14106.42)
Australasia	80 to 84	15063.7 (14674.59 to 15463.13)
Australasia	85 to 89	16441.88 (15979.78 to 16917.34)
Australasia	90 to 94	17590.86 (16999.62 to 18202.67)
Australasia	95+	18572.74 (17651.91 to 19541.6)
Caribbean	55 to 59	7524.28 (7463.1 to 7585.95)
Caribbean	60 to 64	8771.33 (8700.81 to 8842.42)
Caribbean	65 to 69	10243.71 (10145.19 to 10343.18)
Caribbean	70 to 74	12143.07 (12021.8 to 12265.56)
Caribbean	75 to 79	14548.74 (14396.43 to 14702.67)
Caribbean	80 to 84	17740.33 (17542.15 to 17940.75)
Caribbean	85 to 89	22035.48 (21762.58 to 22311.81)
Caribbean	90 to 94	27244.98 (26841.83 to 27654.19)
Caribbean	95+	33360.87 (32730.89 to 34002.98)
Central Asia	55 to 59	7559.56 (7468.7 to 7651.52)
Central Asia	60 to 64	9357.29 (9247.96 to 9467.92)
Central Asia	65 to 69	11524.14 (11361.23 to 11689.38)

Central Asia	70 to 74	14087.44<br>(13881.6 to 14296.34)
Central Asia	75 to 79	16765.73<br>(16509.11 to 17026.35)
Central Asia	80 to 84	19762.7<br>(19432.78 to 20098.23)
Central Asia	85 to 89	23067.79<br>(22610.02 to 23534.84)
Central Asia	90 to 94	26330<br>(25628.32 to 27050.89)
Central Asia	95+	29269.92<br>(28031.16 to 30563.41)
Central Europe	55 to 59	9988.16<br>(9824.24 to 10154.82)
Central Europe	60 to 64	11535.99<br>(11349.07 to 11725.98)
Central Europe	65 to 69	13548.61<br>(13296.64 to 13805.36)
Central Europe	70 to 74	15769.91<br>(15471.5 to 16074.09)
Central Europe	75 to 79	17537.91<br>(17195.78 to 17886.85)
Central Europe	80 to 84	19036.67<br>(18642.55 to 19439.13)
Central Europe	85 to 89	20652.76<br>(20166.21 to 21151.05)
Central Europe	90 to 94	22418.24<br>(21713.82 to 23145.5)
Central Europe	95+	24615.14<br>(23329.24 to 25971.91)
Central Latin America	55 to 59	5534.41<br>(5452.71 to 5617.33)
Central Latin America	60 to 64	6942.42<br>(6843.06 to 7043.22)
Central Latin America	65 to 69	8836.88<br>(8683.71 to 8992.76)
Central Latin America	70 to 74	11552.87<br>(11346.79 to 11762.68)
Central Latin America	75 to 79	15338.24<br>(15055.26 to 15626.53)
Central Latin America	80 to 84	20482.59<br>(20085.37 to 20887.68)
Central Latin America	85 to 89	27094.39<br>(26523.04 to 27678.05)
Central Latin America	90 to 94	33874.52<br>(33040.07 to 34730.04)
Central Latin America	95+	40458.4<br>(39209.26 to 41747.32)
Central Sub-Saharan Africa	55 to 59	7218.13<br>(7187.99 to 7248.4)
Central Sub-Saharan Africa	60 to 64	8983.7<br>(8946.42 to 9021.14)
Central Sub-Saharan Africa	65 to 69	10767.33<br>(10711.94 to 10823)
Central Sub-Saharan Africa	70 to 74	12703.22<br>(12633.64 to 12773.18)
Central Sub-Saharan Africa	75 to 79	14912.44<br>(14823.22 to 15002.2)
Central Sub-Saharan Africa	80 to 84	17684.69<br>(17561.58 to 17808.65)
Central Sub-Saharan Africa	85 to 89	20800.57<br>(20608.61 to 20994.31)
Central Sub-Saharan Africa	90 to 94	23452.64<br>(23094 to 23816.86)
Central Sub-Saharan Africa	95+	25664.65<br>(24849.24 to 26506.81)

East Asia	55 to 59	5956.88<br>(5837.12 to 6079.1)
East Asia	60 to 64	7686.08<br>(7536.19 to 7838.96)
East Asia	65 to 69	10762.01<br>(10515.92 to 11013.87)
East Asia	70 to 74	14546.68<br>(14206.73 to 14894.77)
East Asia	75 to 79	18273.81<br>(17832.53 to 18726.02)
East Asia	80 to 84	21545.63<br>(20989.63 to 22116.36)
East Asia	85 to 89	24483.19<br>(23746.48 to 25242.74)
East Asia	90 to 94	26350.82<br>(25223.76 to 27528.24)
East Asia	95+	28979.15<br>(26629.33 to 31536.31)
Eastern Europe	55 to 59	6917.18<br>(6815.78 to 7020.08)
Eastern Europe	60 to 64	7544.91<br>(7435.27 to 7656.17)
Eastern Europe	65 to 69	8126.2<br>(7991.58 to 8263.08)
Eastern Europe	70 to 74	8749.41<br>(8601.35 to 8900.01)
Eastern Europe	75 to 79	9428.16<br>(9262.66 to 9596.62)
Eastern Europe	80 to 84	10342.91<br>(10149.95 to 10539.55)
Eastern Europe	85 to 89	11515.68<br>(11267.6 to 11769.22)
Eastern Europe	90 to 94	12801.57<br>(12429.85 to 13184.41)
Eastern Europe	95+	14168.65<br>(13408.63 to 14971.76)
Eastern Sub-Saharan Africa	55 to 59	7106.12<br>(7067.63 to 7144.82)
Eastern Sub-Saharan Africa	60 to 64	8585.07<br>(8539.21 to 8631.19)
Eastern Sub-Saharan Africa	65 to 69	9775.04<br>(9710.97 to 9839.54)
Eastern Sub-Saharan Africa	70 to 74	10858.52<br>(10782.8 to 10934.77)
Eastern Sub-Saharan Africa	75 to 79	12056.18<br>(11964.58 to 12148.48)
Eastern Sub-Saharan Africa	80 to 84	13300.71<br>(13183.15 to 13419.33)
Eastern Sub-Saharan Africa	85 to 89	14183.09<br>(14016.15 to 14352.03)
Eastern Sub-Saharan Africa	90 to 94	14512.67<br>(14232.11 to 14798.76)
Eastern Sub-Saharan Africa	95+	14559.74<br>(14001.07 to 15140.7)
High-income Asia Pacific	55 to 59	5835.66<br>(5505.93 to 6185.13)
High-income Asia Pacific	60 to 64	6640.89<br>(6271.02 to 7032.58)
High-income Asia Pacific	65 to 69	7709.69<br>(7235.02 to 8215.49)
High-income Asia Pacific	70 to 74	8838.93<br>(8290.89 to 9423.2)
High-income Asia Pacific	75 to 79	9948.81<br>(9323.44 to 10616.12)
High-income Asia Pacific	80 to 84	11186.19<br>(10466.18 to 11955.73)

High-income Asia Pacific	85 to 89	12800.84<br>(11940.01 to 13723.74)
High-income Asia Pacific	90 to 94	14466.83<br>(13401.38 to 15616.99)
High-income Asia Pacific	95+	16114.31<br>(14706.82 to 17656.5)
High-income North America	55 to 59	14719.07<br>(14236.89 to 15217.58)
High-income North America	60 to 64	18925.43<br>(18319.36 to 19551.55)
High-income North America	65 to 69	26474.39<br>(25443.15 to 27547.43)
High-income North America	70 to 74	35011.56<br>(33598.77 to 36483.76)
High-income North America	75 to 79	43614.45<br>(41788.03 to 45520.7)
High-income North America	80 to 84	50756.87<br>(48520.22 to 53096.63)
High-income North America	85 to 89	56252.13<br>(53536.84 to 59105.13)
High-income North America	90 to 94	59879.42<br>(56388.14 to 63586.86)
High-income North America	95+	62896.45<br>(57517.98 to 68777.87)
North Africa and Middle East	55 to 59	8655.87<br>(8570.44 to 8742.16)
North Africa and Middle East	60 to 64	10517.6<br>(10415.57 to 10620.63)
North Africa and Middle East	65 to 69	12824.72<br>(12672.53 to 12978.74)
North Africa and Middle East	70 to 74	15724.44<br>(15529.61 to 15921.71)
North Africa and Middle East	75 to 79	19213.39<br>(18961.74 to 19468.38)
North Africa and Middle East	80 to 84	23694.82<br>(23356.19 to 24038.36)
North Africa and Middle East	85 to 89	29671.81<br>(29171.59 to 30180.62)
North Africa and Middle East	90 to 94	36816.2<br>(35966.5 to 37685.96)
North Africa and Middle East	95+	45992.85<br>(44444.93 to 47594.68)
Oceania	55 to 59	8828.12<br>(8746.94 to 8910.05)
Oceania	60 to 64	10699.74<br>(10601.84 to 10798.55)
Oceania	65 to 69	13847.88<br>(13693.09 to 14004.42)
Oceania	70 to 74	17304.17<br>(17101.28 to 17509.47)
Oceania	75 to 79	19905.86<br>(19654.47 to 20160.46)
Oceania	80 to 84	21634.69<br>(21322.22 to 21951.74)
Oceania	85 to 89	23797.78<br>(23358.75 to 24245.07)
Oceania	90 to 94	25326.94<br>(24595.43 to 26080.21)
Oceania	95+	26562.72<br>(25144.75 to 28060.64)
South Asia	55 to 59	9236.12<br>(9190.45 to 9282.02)
South Asia	60 to 64	11801.43<br>(11744.91 to 11858.23)
South Asia	65 to 69	16108.51<br>(16015.93 to 16201.63)

South Asia	70 to 74	21072.07 (20946.36 to 21198.53)
South Asia	75 to 79	26116.09 (25951.36 to 26281.86)
South Asia	80 to 84	30651.4 (30437.99 to 30866.3)
South Asia	85 to 89	35848.4 (35547.6 to 36151.74)
South Asia	90 to 94	41467.66 (40977.77 to 41963.4)
South Asia	95+	48262.34 (47274.18 to 49271.15)
Southeast Asia	55 to 59	7310.75 (7265.82 to 7355.95)
Southeast Asia	60 to 64	9083.12 (9028.58 to 9138)
Southeast Asia	65 to 69	11185.41 (11103.18 to 11268.24)
Southeast Asia	70 to 74	13624.96 (13520.4 to 13730.34)
Southeast Asia	75 to 79	16140.69 (16009.38 to 16273.09)
Southeast Asia	80 to 84	19022.75 (18852.51 to 19194.53)
Southeast Asia	85 to 89	22452.81 (22213.49 to 22694.71)
Southeast Asia	90 to 94	25769.7 (25390.59 to 26154.46)
Southeast Asia	95+	28813.6 (28129.4 to 29514.45)
Southern Latin America	55 to 59	9915.09 (9824.47 to 10006.55)
Southern Latin America	60 to 64	12074.5 (11967 to 12182.97)
Southern Latin America	65 to 69	14043 (13896.34 to 14191.2)
Southern Latin America	70 to 74	15817.1 (15647.47 to 15988.57)
Southern Latin America	75 to 79	17321.96 (17128.59 to 17517.51)
Southern Latin America	80 to 84	18943.89 (18718.82 to 19171.66)
Southern Latin America	85 to 89	21197.52 (20917.2 to 21481.6)
Southern Latin America	90 to 94	23593.3 (23203.34 to 23989.81)
Southern Latin America	95+	25682.09 (25058.64 to 26321.05)
Southern Sub-Saharan Africa	55 to 59	6825.91 (6722.58 to 6930.82)
Southern Sub-Saharan Africa	60 to 64	8301.67 (8177.09 to 8428.14)
Southern Sub-Saharan Africa	65 to 69	10144.94 (9960.99 to 10332.28)
Southern Sub-Saharan Africa	70 to 74	12348.47 (12114.34 to 12587.13)
Southern Sub-Saharan Africa	75 to 79	14951.27 (14650.41 to 15258.32)
Southern Sub-Saharan Africa	80 to 84	18261.78 (17864.26 to 18668.14)
Southern Sub-Saharan Africa	85 to 89	22140.43 (21574.54 to 22721.15)
Southern Sub-Saharan Africa	90 to 94	25928.36 (24982.51 to 26910.03)
Southern Sub-Saharan Africa	95+	28787.21 (26732.79 to 30999.51)

Tropical Latin America	55 to 59	7032.77<br>(6961.43 to 7104.85)
Tropical Latin America	60 to 64	8426.68<br>(8343.15 to 8511.04)
Tropical Latin America	65 to 69	10540.8<br>(10414.81 to 10668.32)
Tropical Latin America	70 to 74	13447.56<br>(13281.78 to 13615.4)
Tropical Latin America	75 to 79	16922.61<br>(16705.44 to 17142.6)
Tropical Latin America	80 to 84	21076.32<br>(20789.36 to 21367.24)
Tropical Latin America	85 to 89	26249.15<br>(25853.98 to 26650.36)
Tropical Latin America	90 to 94	31714.27<br>(31141.8 to 32297.26)
Tropical Latin America	95+	37068.43<br>(36187.48 to 37970.84)
Western Europe	55 to 59	11071.7<br>(10750.4 to 11402.59)
Western Europe	60 to 64	12094.09<br>(11747.07 to 12451.36)
Western Europe	65 to 69	13258.86<br>(12824.77 to 13707.64)
Western Europe	70 to 74	14637.89<br>(14151.19 to 15141.32)
Western Europe	75 to 79	16490.49<br>(15930.37 to 17070.3)
Western Europe	80 to 84	19360.42<br>(18682.13 to 20063.33)
Western Europe	85 to 89	23799.97<br>(22913.42 to 24720.82)
Western Europe	90 to 94	28921.62<br>(27687.89 to 30210.33)
Western Europe	95+	34544.78<br>(32568.95 to 36640.47)
Western Sub-Saharan Africa	55 to 59	7511.51<br>(7481.11 to 7542.03)
Western Sub-Saharan Africa	60 to 64	9672.36<br>(9633.96 to 9710.91)
Western Sub-Saharan Africa	65 to 69	10947.64<br>(10893.59 to 11001.97)
Western Sub-Saharan Africa	70 to 74	11886.47<br>(11823.84 to 11949.44)
Western Sub-Saharan Africa	75 to 79	13664.61<br>(13586.93 to 13742.72)
Western Sub-Saharan Africa	80 to 84	16255.58<br>(16150.88 to 16360.95)
Western Sub-Saharan Africa	85 to 89	18760.76<br>(18608.29 to 18914.49)
Western Sub-Saharan Africa	90 to 94	20799.03<br>(20543.17 to 21058.09)
Western Sub-Saharan Africa	95+	22454.45<br>(21953.37 to 22966.96)

CRDs<br>Location	Period	Prevalence rate ratio
Global	1992 to 1996	1.14<br>(1.13 to 1.15)
Global	1997 to 2001	1.07<br>(1.06 to 1.07)
Global	2002 to 2006	1.02<br>(1.01 to 1.03)
Global	2007 to 2011	1.01<br>(1 to 1.02)

Global	2012 to 2016	1<br>(1 to 1)
Global	2017 to 2021	1.01<br>(1 to 1.02)
High SDI	1992 to 1996	1.19<br>(1.17 to 1.21)
High SDI	1997 to 2001	1.07<br>(1.05 to 1.08)
High SDI	2002 to 2006	1.02<br>(1 to 1.03)
High SDI	2007 to 2011	1.01<br>(0.99 to 1.02)
High SDI	2012 to 2016	1<br>(1 to 1)
High SDI	2017 to 2021	1<br>(0.99 to 1.02)
High-middle SDI	1992 to 1996	1.22<br>(1.2 to 1.24)
High-middle SDI	1997 to 2001	1.12<br>(1.11 to 1.14)
High-middle SDI	2002 to 2006	1.05<br>(1.04 to 1.06)
High-middle SDI	2007 to 2011	1.01<br>(1 to 1.02)
High-middle SDI	2012 to 2016	1<br>(1 to 1)
High-middle SDI	2017 to 2021	1.02<br>(1.01 to 1.03)
Middle SDI	1992 to 1996	1.06<br>(1.05 to 1.08)
Middle SDI	1997 to 2001	1.03<br>(1.02 to 1.05)
Middle SDI	2002 to 2006	1.01<br>(1 to 1.02)
Middle SDI	2007 to 2011	1<br>(0.99 to 1.01)
Middle SDI	2012 to 2016	1<br>(1 to 1)
Middle SDI	2017 to 2021	1.02<br>(1.01 to 1.02)
Low-middle SDI	1992 to 1996	1.01<br>(1 to 1.01)
Low-middle SDI	1997 to 2001	0.99<br>(0.98 to 0.99)
Low-middle SDI	2002 to 2006	0.99<br>(0.99 to 0.99)
Low-middle SDI	2007 to 2011	0.99<br>(0.99 to 1)
Low-middle SDI	2012 to 2016	1<br>(1 to 1)
Low-middle SDI	2017 to 2021	1.02<br>(1.01 to 1.02)
Low SDI	1992 to 1996	1.01<br>(1 to 1.03)
Low SDI	1997 to 2001	1<br>(0.98 to 1.01)
Low SDI	2002 to 2006	0.99<br>(0.98 to 1.01)
Low SDI	2007 to 2011	1<br>(0.99 to 1.01)
Low SDI	2012 to 2016	1<br>(1 to 1)
Low SDI	2017 to 2021	1.03<br>(1.02 to 1.04)
Andean Latin America	1992 to 1996	0.94<br>(0.93 to 0.94)

Andean Latin America	1997 to 2001	0.96 (0.96 to 0.97)
Andean Latin America	2002 to 2006	0.97 (0.97 to 0.98)
Andean Latin America	2007 to 2011	0.98 (0.97 to 0.98)
Andean Latin America	2012 to 2016	1 (1 to 1)
Andean Latin America	2017 to 2021	1.04 (1.03 to 1.04)
Australasia	1992 to 1996	1.31 (1.29 to 1.34)
Australasia	1997 to 2001	1.24 (1.22 to 1.26)
Australasia	2002 to 2006	1.17 (1.15 to 1.18)
Australasia	2007 to 2011	1.05 (1.04 to 1.07)
Australasia	2012 to 2016	1 (1 to 1)
Australasia	2017 to 2021	0.98 (0.97 to 1)
Caribbean	1992 to 1996	0.97 (0.96 to 0.98)
Caribbean	1997 to 2001	0.98 (0.98 to 0.99)
Caribbean	2002 to 2006	0.99 (0.98 to 1)
Caribbean	2007 to 2011	1 (0.99 to 1.01)
Caribbean	2012 to 2016	1 (1 to 1)
Caribbean	2017 to 2021	1.03 (1.02 to 1.04)
Central Asia	1992 to 1996	1.08 (1.07 to 1.09)
Central Asia	1997 to 2001	1.03 (1.02 to 1.04)
Central Asia	2002 to 2006	0.99 (0.98 to 1)
Central Asia	2007 to 2011	0.98 (0.97 to 0.99)
Central Asia	2012 to 2016	1 (1 to 1)
Central Asia	2017 to 2021	1.05 (1.04 to 1.07)
Central Europe	1992 to 1996	1.25 (1.23 to 1.27)
Central Europe	1997 to 2001	1.13 (1.11 to 1.14)
Central Europe	2002 to 2006	1.03 (1.01 to 1.04)
Central Europe	2007 to 2011	0.99 (0.98 to 1)
Central Europe	2012 to 2016	1 (1 to 1)
Central Europe	2017 to 2021	1.03 (1.01 to 1.04)
Central Latin America	1992 to 1996	1.09 (1.07 to 1.1)
Central Latin America	1997 to 2001	1.06 (1.04 to 1.07)
Central Latin America	2002 to 2006	1.02 (1 to 1.03)
Central Latin America	2007 to 2011	1 (0.99 to 1.01)

Central Latin America	2012 to 2016	1<br>(1 to 1)
Central Latin America	2017 to 2021	1.03<br>(1.02 to 1.04)
Central Sub-Saharan Africa	1992 to 1996	0.98<br>(0.97 to 0.99)
Central Sub-Saharan Africa	1997 to 2001	0.97<br>(0.97 to 0.98)
Central Sub-Saharan Africa	2002 to 2006	0.98<br>(0.97 to 0.98)
Central Sub-Saharan Africa	2007 to 2011	0.99<br>(0.99 to 1)
Central Sub-Saharan Africa	2012 to 2016	1<br>(1 to 1)
Central Sub-Saharan Africa	2017 to 2021	1.03<br>(1.02 to 1.03)
East Asia	1992 to 1996	1.11<br>(1.07 to 1.14)
East Asia	1997 to 2001	1.06<br>(1.03 to 1.09)
East Asia	2002 to 2006	1.01<br>(0.99 to 1.03)
East Asia	2007 to 2011	1<br>(0.98 to 1.01)
East Asia	2012 to 2016	1<br>(1 to 1)
East Asia	2017 to 2021	0.99<br>(0.98 to 1.01)
Eastern Europe	1992 to 1996	1.46<br>(1.44 to 1.49)
Eastern Europe	1997 to 2001	1.3<br>(1.29 to 1.32)
Eastern Europe	2002 to 2006	1.17<br>(1.15 to 1.18)
Eastern Europe	2007 to 2011	1.07<br>(1.05 to 1.08)
Eastern Europe	2012 to 2016	1<br>(1 to 1)
Eastern Europe	2017 to 2021	1.02<br>(1.01 to 1.04)
Eastern Sub-Saharan Africa	1992 to 1996	1.08<br>(1.06 to 1.09)
Eastern Sub-Saharan Africa	1997 to 2001	1.05<br>(1.04 to 1.06)
Eastern Sub-Saharan Africa	2002 to 2006	1.02<br>(1.01 to 1.03)
Eastern Sub-Saharan Africa	2007 to 2011	1<br>(1 to 1.01)
Eastern Sub-Saharan Africa	2012 to 2016	1<br>(1 to 1)
Eastern Sub-Saharan Africa	2017 to 2021	1.04<br>(1.04 to 1.05)
High-income Asia Pacific	1992 to 1996	1.74<br>(1.68 to 1.8)
High-income Asia Pacific	1997 to 2001	1.44<br>(1.39 to 1.49)
High-income Asia Pacific	2002 to 2006	1.18<br>(1.14 to 1.22)
High-income Asia Pacific	2007 to 2011	1.07<br>(1.03 to 1.1)
High-income Asia Pacific	2012 to 2016	1<br>(1 to 1)
High-income Asia Pacific	2017 to 2021	0.95<br>(0.92 to 0.98)
High-income North America	1992 to 1996	0.87<br>(0.84 to 0.89)

High-income North America	1997 to 2001	0.84 (0.82 to 0.87)
High-income North America	2002 to 2006	0.89 (0.87 to 0.92)
High-income North America	2007 to 2011	0.96 (0.94 to 0.98)
High-income North America	2012 to 2016	1 (1 to 1)
High-income North America	2017 to 2021	1 (0.97 to 1.02)
North Africa and Middle East	1992 to 1996	1 (0.99 to 1.02)
North Africa and Middle East	1997 to 2001	0.98 (0.97 to 0.99)
North Africa and Middle East	2002 to 2006	0.98 (0.97 to 0.99)
North Africa and Middle East	2007 to 2011	0.98 (0.97 to 0.99)
North Africa and Middle East	2012 to 2016	1 (1 to 1)
North Africa and Middle East	2017 to 2021	1.05 (1.05 to 1.06)
Oceania	1992 to 1996	1.15 (1.13 to 1.17)
Oceania	1997 to 2001	1.12 (1.1 to 1.13)
Oceania	2002 to 2006	1.07 (1.06 to 1.09)
Oceania	2007 to 2011	1.03 (1.02 to 1.04)
Oceania	2012 to 2016	1 (1 to 1)
Oceania	2017 to 2021	0.98 (0.97 to 0.99)
South Asia	1992 to 1996	1.01 (1 to 1.01)
South Asia	1997 to 2001	0.99 (0.98 to 0.99)
South Asia	2002 to 2006	0.99 (0.99 to 1)
South Asia	2007 to 2011	1 (1 to 1.01)
South Asia	2012 to 2016	1 (1 to 1)
South Asia	2017 to 2021	1.01 (1 to 1.01)
Southeast Asia	1992 to 1996	1.1 (1.09 to 1.11)
Southeast Asia	1997 to 2001	1.06 (1.06 to 1.07)
Southeast Asia	2002 to 2006	1.04 (1.03 to 1.04)
Southeast Asia	2007 to 2011	1.02 (1.02 to 1.03)
Southeast Asia	2012 to 2016	1 (1 to 1)
Southeast Asia	2017 to 2021	1.02 (1.02 to 1.03)
Southern Latin America	1992 to 1996	1.16 (1.15 to 1.17)
Southern Latin America	1997 to 2001	1.13 (1.13 to 1.14)
Southern Latin America	2002 to 2006	1.09 (1.08 to 1.1)
Southern Latin America	2007 to 2011	1.04 (1.04 to 1.05)

Southern Latin America	2012 to 2016	1<br>(1 to 1)
Southern Latin America	2017 to 2021	1.01<br>(1.01 to 1.02)
Southern Sub-Saharan Africa	1992 to 1996	1.04<br>(1.02 to 1.06)
Southern Sub-Saharan Africa	1997 to 2001	1.05<br>(1.03 to 1.06)
Southern Sub-Saharan Africa	2002 to 2006	1.04<br>(1.03 to 1.06)
Southern Sub-Saharan Africa	2007 to 2011	1.02<br>(1.01 to 1.03)
Southern Sub-Saharan Africa	2012 to 2016	1<br>(1 to 1)
Southern Sub-Saharan Africa	2017 to 2021	1<br>(0.99 to 1.02)
Tropical Latin America	1992 to 1996	1.13<br>(1.12 to 1.14)
Tropical Latin America	1997 to 2001	1.12<br>(1.11 to 1.13)
Tropical Latin America	2002 to 2006	1.09<br>(1.08 to 1.1)
Tropical Latin America	2007 to 2011	1.02<br>(1.01 to 1.03)
Tropical Latin America	2012 to 2016	1<br>(1 to 1)
Tropical Latin America	2017 to 2021	1.03<br>(1.02 to 1.04)
Western Europe	1992 to 1996	1.29<br>(1.26 to 1.32)
Western Europe	1997 to 2001	1.13<br>(1.11 to 1.16)
Western Europe	2002 to 2006	1.07<br>(1.05 to 1.09)
Western Europe	2007 to 2011	1.04<br>(1.02 to 1.06)
Western Europe	2012 to 2016	1<br>(1 to 1)
Western Europe	2017 to 2021	1.02<br>(1 to 1.04)
Western Sub-Saharan Africa	1992 to 1996	1.01<br>(1.01 to 1.02)
Western Sub-Saharan Africa	1997 to 2001	0.98<br>(0.97 to 0.98)
Western Sub-Saharan Africa	2002 to 2006	0.97<br>(0.97 to 0.98)
Western Sub-Saharan Africa	2007 to 2011	0.99<br>(0.98 to 0.99)
Western Sub-Saharan Africa	2012 to 2016	1<br>(1 to 1)
Western Sub-Saharan Africa	2017 to 2021	1.05<br>(1.05 to 1.06)

CRDs<br>Location	Cohort	Prevalence rate ratio
Global	1897 to 1901	1.2<br>(1.09 to 1.32)
Global	1902 to 1906	1.27<br>(1.22 to 1.33)
Global	1907 to 1911	1.32<br>(1.29 to 1.36)
Global	1912 to 1916	1.36<br>(1.34 to 1.38)
Global	1917 to 1921	1.38<br>(1.36 to 1.4)

Global	1922 to 1926	1.38 (1.36 to 1.4)
Global	1927 to 1931	1.35 (1.33 to 1.37)
Global	1932 to 1936	1.31 (1.3 to 1.33)
Global	1937 to 1941	1.26 (1.24 to 1.27)
Global	1942 to 1946	1.21 (1.19 to 1.22)
Global	1947 to 1951	1.14 (1.13 to 1.16)
Global	1952 to 1956	1.06 (1.05 to 1.08)
Global	1957 to 1961	1 (1 to 1)
Global	1962 to 1966	0.93 (0.92 to 0.95)
High SDI	1897 to 1901	1.23 (1.06 to 1.43)
High SDI	1902 to 1906	1.31 (1.22 to 1.41)
High SDI	1907 to 1911	1.36 (1.3 to 1.42)
High SDI	1912 to 1916	1.4 (1.35 to 1.45)
High SDI	1917 to 1921	1.42 (1.38 to 1.47)
High SDI	1922 to 1926	1.39 (1.35 to 1.43)
High SDI	1927 to 1931	1.35 (1.31 to 1.38)
High SDI	1932 to 1936	1.28 (1.25 to 1.31)
High SDI	1937 to 1941	1.21 (1.18 to 1.24)
High SDI	1942 to 1946	1.16 (1.13 to 1.19)
High SDI	1947 to 1951	1.11 (1.08 to 1.14)
High SDI	1952 to 1956	1.05 (1.03 to 1.08)
High SDI	1957 to 1961	1 (1 to 1)
High SDI	1962 to 1966	0.94 (0.91 to 0.98)
High-middle SDI	1897 to 1901	1.41 (1.22 to 1.64)
High-middle SDI	1902 to 1906	1.49 (1.41 to 1.59)
High-middle SDI	1907 to 1911	1.56 (1.51 to 1.62)
High-middle SDI	1912 to 1916	1.62 (1.58 to 1.66)
High-middle SDI	1917 to 1921	1.66 (1.63 to 1.7)
High-middle SDI	1922 to 1926	1.66 (1.63 to 1.7)
High-middle SDI	1927 to 1931	1.62 (1.59 to 1.65)
High-middle SDI	1932 to 1936	1.57 (1.55 to 1.6)
High-middle SDI	1937 to 1941	1.47 (1.45 to 1.49)
High-middle SDI	1942 to 1946	1.38 (1.35 to 1.4)

High-middle SDI	1947 to 1951	1.25 (1.23 to 1.27)
High-middle SDI	1952 to 1956	1.11 (1.09 to 1.13)
High-middle SDI	1957 to 1961	1 (1 to 1)
High-middle SDI	1962 to 1966	0.9 (0.88 to 0.92)
Middle SDI	1897 to 1901	1.01 (0.86 to 1.19)
Middle SDI	1902 to 1906	1.09 (1.02 to 1.17)
Middle SDI	1907 to 1911	1.16 (1.12 to 1.2)
Middle SDI	1912 to 1916	1.21 (1.19 to 1.24)
Middle SDI	1917 to 1921	1.26 (1.23 to 1.28)
Middle SDI	1922 to 1926	1.27 (1.25 to 1.3)
Middle SDI	1927 to 1931	1.27 (1.26 to 1.29)
Middle SDI	1932 to 1936	1.26 (1.24 to 1.27)
Middle SDI	1937 to 1941	1.22 (1.21 to 1.24)
Middle SDI	1942 to 1946	1.18 (1.16 to 1.19)
Middle SDI	1947 to 1951	1.12 (1.1 to 1.13)
Middle SDI	1952 to 1956	1.05 (1.04 to 1.07)
Middle SDI	1957 to 1961	1 (1 to 1)
Middle SDI	1962 to 1966	0.94 (0.93 to 0.96)
Low-middle SDI	1897 to 1901	0.94 (0.89 to 0.99)
Low-middle SDI	1902 to 1906	0.98 (0.95 to 1)
Low-middle SDI	1907 to 1911	1.02 (1.01 to 1.03)
Low-middle SDI	1912 to 1916	1.05 (1.04 to 1.06)
Low-middle SDI	1917 to 1921	1.07 (1.07 to 1.08)
Low-middle SDI	1922 to 1926	1.09 (1.09 to 1.1)
Low-middle SDI	1927 to 1931	1.11 (1.1 to 1.12)
Low-middle SDI	1932 to 1936	1.12 (1.11 to 1.12)
Low-middle SDI	1937 to 1941	1.11 (1.11 to 1.12)
Low-middle SDI	1942 to 1946	1.09 (1.09 to 1.1)
Low-middle SDI	1947 to 1951	1.06 (1.06 to 1.07)
Low-middle SDI	1952 to 1956	1.03 (1.03 to 1.04)
Low-middle SDI	1957 to 1961	1 (1 to 1)
Low-middle SDI	1962 to 1966	0.97 (0.97 to 0.98)
Low SDI	1897 to 1901	0.92 (0.75 to 1.12)

Low SDI	1902 to 1906	0.98<br>(0.9 to 1.06)
Low SDI	1907 to 1911	1.05<br>(1.01 to 1.09)
Low SDI	1912 to 1916	1.07<br>(1.04 to 1.1)
Low SDI	1917 to 1921	1.08<br>(1.06 to 1.1)
Low SDI	1922 to 1926	1.1<br>(1.08 to 1.11)
Low SDI	1927 to 1931	1.11<br>(1.09 to 1.13)
Low SDI	1932 to 1936	1.12<br>(1.11 to 1.14)
Low SDI	1937 to 1941	1.12<br>(1.11 to 1.14)
Low SDI	1942 to 1946	1.11<br>(1.09 to 1.12)
Low SDI	1947 to 1951	1.08<br>(1.07 to 1.09)
Low SDI	1952 to 1956	1.04<br>(1.03 to 1.05)
Low SDI	1957 to 1961	1<br>(1 to 1)
Low SDI	1962 to 1966	0.96<br>(0.95 to 0.98)
Andean Latin America	1897 to 1901	0.74<br>(0.7 to 0.77)
Andean Latin America	1902 to 1906	0.81<br>(0.79 to 0.83)
Andean Latin America	1907 to 1911	0.88<br>(0.87 to 0.9)
Andean Latin America	1912 to 1916	0.95<br>(0.93 to 0.96)
Andean Latin America	1917 to 1921	1<br>(0.99 to 1.01)
Andean Latin America	1922 to 1926	1.04<br>(1.03 to 1.05)
Andean Latin America	1927 to 1931	1.07<br>(1.06 to 1.08)
Andean Latin America	1932 to 1936	1.08<br>(1.07 to 1.09)
Andean Latin America	1937 to 1941	1.08<br>(1.08 to 1.09)
Andean Latin America	1942 to 1946	1.08<br>(1.07 to 1.08)
Andean Latin America	1947 to 1951	1.06<br>(1.05 to 1.07)
Andean Latin America	1952 to 1956	1.03<br>(1.02 to 1.04)
Andean Latin America	1957 to 1961	1<br>(1 to 1)
Andean Latin America	1962 to 1966	0.97<br>(0.96 to 0.98)
Australasia	1897 to 1901	1.96<br>(1.66 to 2.31)
Australasia	1902 to 1906	1.99<br>(1.85 to 2.15)
Australasia	1907 to 1911	2<br>(1.91 to 2.09)
Australasia	1912 to 1916	2<br>(1.93 to 2.07)
Australasia	1917 to 1921	1.97<br>(1.91 to 2.03)
Australasia	1922 to 1926	1.91<br>(1.86 to 1.96)

Australasia	1927 to 1931	1.82<br>(1.78 to 1.87)
Australasia	1932 to 1936	1.7<br>(1.66 to 1.74)
Australasia	1937 to 1941	1.56<br>(1.53 to 1.6)
Australasia	1942 to 1946	1.43<br>(1.39 to 1.46)
Australasia	1947 to 1951	1.29<br>(1.26 to 1.32)
Australasia	1952 to 1956	1.14<br>(1.11 to 1.17)
Australasia	1957 to 1961	1<br>(1 to 1)
Australasia	1962 to 1966	0.88<br>(0.85 to 0.91)
Caribbean	1897 to 1901	0.85<br>(0.78 to 0.91)
Caribbean	1902 to 1906	0.91<br>(0.88 to 0.94)
Caribbean	1907 to 1911	0.96<br>(0.94 to 0.98)
Caribbean	1912 to 1916	1<br>(0.99 to 1.02)
Caribbean	1917 to 1921	1.04<br>(1.03 to 1.06)
Caribbean	1922 to 1926	1.08<br>(1.07 to 1.09)
Caribbean	1927 to 1931	1.11<br>(1.09 to 1.12)
Caribbean	1932 to 1936	1.12<br>(1.11 to 1.13)
Caribbean	1937 to 1941	1.12<br>(1.11 to 1.13)
Caribbean	1942 to 1946	1.11<br>(1.1 to 1.12)
Caribbean	1947 to 1951	1.08<br>(1.07 to 1.09)
Caribbean	1952 to 1956	1.04<br>(1.03 to 1.05)
Caribbean	1957 to 1961	1<br>(1 to 1)
Caribbean	1962 to 1966	0.97<br>(0.95 to 0.98)
Central Asia	1897 to 1901	1.06<br>(0.94 to 1.2)
Central Asia	1902 to 1906	1.1<br>(1.04 to 1.16)
Central Asia	1907 to 1911	1.14<br>(1.1 to 1.17)
Central Asia	1912 to 1916	1.18<br>(1.15 to 1.21)
Central Asia	1917 to 1921	1.22<br>(1.19 to 1.24)
Central Asia	1922 to 1926	1.26<br>(1.23 to 1.28)
Central Asia	1927 to 1931	1.27<br>(1.25 to 1.29)
Central Asia	1932 to 1936	1.27<br>(1.26 to 1.29)
Central Asia	1937 to 1941	1.24<br>(1.23 to 1.26)
Central Asia	1942 to 1946	1.21<br>(1.19 to 1.23)
Central Asia	1947 to 1951	1.14<br>(1.13 to 1.16)

Central Asia	1952 to 1956	1.08 (1.06 to 1.09)
Central Asia	1957 to 1961	1 (1 to 1)
Central Asia	1962 to 1966	0.92 (0.91 to 0.94)
Central Europe	1897 to 1901	1.35 (1.12 to 1.63)
Central Europe	1902 to 1906	1.46 (1.35 to 1.57)
Central Europe	1907 to 1911	1.55 (1.49 to 1.62)
Central Europe	1912 to 1916	1.6 (1.56 to 1.65)
Central Europe	1917 to 1921	1.64 (1.6 to 1.68)
Central Europe	1922 to 1926	1.62 (1.59 to 1.66)
Central Europe	1927 to 1931	1.59 (1.55 to 1.62)
Central Europe	1932 to 1936	1.51 (1.48 to 1.54)
Central Europe	1937 to 1941	1.4 (1.37 to 1.42)
Central Europe	1942 to 1946	1.28 (1.25 to 1.3)
Central Europe	1947 to 1951	1.18 (1.16 to 1.2)
Central Europe	1952 to 1956	1.09 (1.07 to 1.11)
Central Europe	1957 to 1961	1 (1 to 1)
Central Europe	1962 to 1966	0.91 (0.88 to 0.93)
Central Latin America	1897 to 1901	1.1 (0.98 to 1.23)
Central Latin America	1902 to 1906	1.15 (1.09 to 1.22)
Central Latin America	1907 to 1911	1.21 (1.17 to 1.25)
Central Latin America	1912 to 1916	1.26 (1.23 to 1.3)
Central Latin America	1917 to 1921	1.3 (1.27 to 1.33)
Central Latin America	1922 to 1926	1.32 (1.29 to 1.34)
Central Latin America	1927 to 1931	1.32 (1.3 to 1.35)
Central Latin America	1932 to 1936	1.31 (1.28 to 1.33)
Central Latin America	1937 to 1941	1.27 (1.25 to 1.29)
Central Latin America	1942 to 1946	1.21 (1.19 to 1.24)
Central Latin America	1947 to 1951	1.15 (1.13 to 1.17)
Central Latin America	1952 to 1956	1.07 (1.06 to 1.09)
Central Latin America	1957 to 1961	1 (1 to 1)
Central Latin America	1962 to 1966	0.93 (0.91 to 0.95)
Central Sub-Saharan Africa	1897 to 1901	0.85 (0.76 to 0.95)
Central Sub-Saharan Africa	1902 to 1906	0.89 (0.85 to 0.94)

Central Sub-Saharan Africa	1907 to 1911	0.94<br>(0.92 to 0.96)
Central Sub-Saharan Africa	1912 to 1916	0.99<br>(0.98 to 1.01)
Central Sub-Saharan Africa	1917 to 1921	1.04<br>(1.03 to 1.05)
Central Sub-Saharan Africa	1922 to 1926	1.07<br>(1.06 to 1.08)
Central Sub-Saharan Africa	1927 to 1931	1.09<br>(1.08 to 1.1)
Central Sub-Saharan Africa	1932 to 1936	1.1<br>(1.09 to 1.11)
Central Sub-Saharan Africa	1937 to 1941	1.1<br>(1.09 to 1.1)
Central Sub-Saharan Africa	1942 to 1946	1.09<br>(1.08 to 1.09)
Central Sub-Saharan Africa	1947 to 1951	1.07<br>(1.06 to 1.07)
Central Sub-Saharan Africa	1952 to 1956	1.03<br>(1.03 to 1.04)
Central Sub-Saharan Africa	1957 to 1961	1<br>(1 to 1)
Central Sub-Saharan Africa	1962 to 1966	0.96<br>(0.95 to 0.97)
East Asia	1897 to 1901	1.13<br>(0.73 to 1.76)
East Asia	1902 to 1906	1.22<br>(1.05 to 1.41)
East Asia	1907 to 1911	1.31<br>(1.22 to 1.4)
East Asia	1912 to 1916	1.38<br>(1.32 to 1.43)
East Asia	1917 to 1921	1.43<br>(1.38 to 1.47)
East Asia	1922 to 1926	1.44<br>(1.41 to 1.49)
East Asia	1927 to 1931	1.43<br>(1.39 to 1.46)
East Asia	1932 to 1936	1.38<br>(1.35 to 1.41)
East Asia	1937 to 1941	1.32<br>(1.29 to 1.36)
East Asia	1942 to 1946	1.26<br>(1.23 to 1.29)
East Asia	1947 to 1951	1.16<br>(1.14 to 1.19)
East Asia	1952 to 1956	1.08<br>(1.05 to 1.1)
East Asia	1957 to 1961	1<br>(1 to 1)
East Asia	1962 to 1966	0.93<br>(0.9 to 0.97)
Eastern Europe	1897 to 1901	2.33<br>(2.01 to 2.71)
Eastern Europe	1902 to 1906	2.34<br>(2.2 to 2.49)
Eastern Europe	1907 to 1911	2.33<br>(2.25 to 2.42)
Eastern Europe	1912 to 1916	2.28<br>(2.22 to 2.34)
Eastern Europe	1917 to 1921	2.22<br>(2.17 to 2.27)
Eastern Europe	1922 to 1926	2.13<br>(2.08 to 2.17)
Eastern Europe	1927 to 1931	2.02<br>(1.99 to 2.06)

Eastern Europe	1932 to 1936	1.89<br>(1.86 to 1.92)
Eastern Europe	1937 to 1941	1.7<br>(1.68 to 1.73)
Eastern Europe	1942 to 1946	1.53<br>(1.51 to 1.56)
Eastern Europe	1947 to 1951	1.33<br>(1.31 to 1.36)
Eastern Europe	1952 to 1956	1.17<br>(1.15 to 1.19)
Eastern Europe	1957 to 1961	1<br>(1 to 1)
Eastern Europe	1962 to 1966	0.85<br>(0.83 to 0.87)
Eastern Sub-Saharan Africa	1897 to 1901	1.07<br>(0.94 to 1.23)
Eastern Sub-Saharan Africa	1902 to 1906	1.11<br>(1.05 to 1.17)
Eastern Sub-Saharan Africa	1907 to 1911	1.14<br>(1.11 to 1.17)
Eastern Sub-Saharan Africa	1912 to 1916	1.16<br>(1.14 to 1.18)
Eastern Sub-Saharan Africa	1917 to 1921	1.17<br>(1.15 to 1.18)
Eastern Sub-Saharan Africa	1922 to 1926	1.17<br>(1.16 to 1.18)
Eastern Sub-Saharan Africa	1927 to 1931	1.17<br>(1.16 to 1.18)
Eastern Sub-Saharan Africa	1932 to 1936	1.16<br>(1.16 to 1.17)
Eastern Sub-Saharan Africa	1937 to 1941	1.15<br>(1.14 to 1.15)
Eastern Sub-Saharan Africa	1942 to 1946	1.12<br>(1.11 to 1.13)
Eastern Sub-Saharan Africa	1947 to 1951	1.09<br>(1.08 to 1.09)
Eastern Sub-Saharan Africa	1952 to 1956	1.05<br>(1.04 to 1.05)
Eastern Sub-Saharan Africa	1957 to 1961	1<br>(1 to 1)
Eastern Sub-Saharan Africa	1962 to 1966	0.95<br>(0.94 to 0.95)
High-income Asia Pacific	1897 to 1901	3.37<br>(2.5 to 4.54)
High-income Asia Pacific	1902 to 1906	3.4<br>(2.95 to 3.91)
High-income Asia Pacific	1907 to 1911	3.4<br>(3.09 to 3.74)
High-income Asia Pacific	1912 to 1916	3.32<br>(3.07 to 3.59)
High-income Asia Pacific	1917 to 1921	3.13<br>(2.91 to 3.36)
High-income Asia Pacific	1922 to 1926	2.82<br>(2.64 to 3.02)
High-income Asia Pacific	1927 to 1931	2.49<br>(2.33 to 2.66)
High-income Asia Pacific	1932 to 1936	2.14<br>(2.01 to 2.28)
High-income Asia Pacific	1937 to 1941	1.83<br>(1.72 to 1.95)
High-income Asia Pacific	1942 to 1946	1.58<br>(1.48 to 1.69)
High-income Asia Pacific	1947 to 1951	1.35<br>(1.27 to 1.44)
High-income Asia Pacific	1952 to 1956	1.17<br>(1.09 to 1.25)

High-income Asia Pacific	1957 to 1961	1<br>(1 to 1)
High-income Asia Pacific	1962 to 1966	0.84<br>(0.76 to 0.93)
High-income North America	1897 to 1901	0.61<br>(0.48 to 0.79)
High-income North America	1902 to 1906	0.65<br>(0.58 to 0.74)
High-income North America	1907 to 1911	0.69<br>(0.63 to 0.74)
High-income North America	1912 to 1916	0.72<br>(0.68 to 0.77)
High-income North America	1917 to 1921	0.76<br>(0.72 to 0.8)
High-income North America	1922 to 1926	0.79<br>(0.75 to 0.82)
High-income North America	1927 to 1931	0.81<br>(0.77 to 0.84)
High-income North America	1932 to 1936	0.83<br>(0.79 to 0.86)
High-income North America	1937 to 1941	0.85<br>(0.82 to 0.89)
High-income North America	1942 to 1946	0.88<br>(0.85 to 0.92)
High-income North America	1947 to 1951	0.92<br>(0.88 to 0.95)
High-income North America	1952 to 1956	0.96<br>(0.92 to 1)
High-income North America	1957 to 1961	1<br>(1 to 1)
High-income North America	1962 to 1966	1.04<br>(0.98 to 1.1)
North Africa and Middle East	1897 to 1901	0.83<br>(0.74 to 0.94)
North Africa and Middle East	1902 to 1906	0.92<br>(0.87 to 0.98)
North Africa and Middle East	1907 to 1911	1<br>(0.97 to 1.04)
North Africa and Middle East	1912 to 1916	1.07<br>(1.04 to 1.09)
North Africa and Middle East	1917 to 1921	1.11<br>(1.09 to 1.13)
North Africa and Middle East	1922 to 1926	1.16<br>(1.15 to 1.18)
North Africa and Middle East	1927 to 1931	1.21<br>(1.19 to 1.22)
North Africa and Middle East	1932 to 1936	1.22<br>(1.21 to 1.24)
North Africa and Middle East	1937 to 1941	1.2<br>(1.19 to 1.22)
North Africa and Middle East	1942 to 1946	1.18<br>(1.16 to 1.19)
North Africa and Middle East	1947 to 1951	1.13<br>(1.12 to 1.15)
North Africa and Middle East	1952 to 1956	1.07<br>(1.06 to 1.08)
North Africa and Middle East	1957 to 1961	1<br>(1 to 1)
North Africa and Middle East	1962 to 1966	0.94<br>(0.93 to 0.96)
Oceania	1897 to 1901	1.46<br>(1.18 to 1.8)
Oceania	1902 to 1906	1.43<br>(1.31 to 1.56)
Oceania	1907 to 1911	1.4<br>(1.34 to 1.46)

Oceania	1912 to 1916	1.39<br>(1.35 to 1.42)
Oceania	1917 to 1921	1.38<br>(1.35 to 1.4)
Oceania	1922 to 1926	1.36<br>(1.34 to 1.38)
Oceania	1927 to 1931	1.31<br>(1.3 to 1.33)
Oceania	1932 to 1936	1.25<br>(1.24 to 1.27)
Oceania	1937 to 1941	1.2<br>(1.19 to 1.22)
Oceania	1942 to 1946	1.15<br>(1.14 to 1.17)
Oceania	1947 to 1951	1.11<br>(1.09 to 1.12)
Oceania	1952 to 1956	1.05<br>(1.03 to 1.06)
Oceania	1957 to 1961	1<br>(1 to 1)
Oceania	1962 to 1966	0.95<br>(0.94 to 0.97)
South Asia	1897 to 1901	0.96<br>(0.89 to 1.03)
South Asia	1902 to 1906	0.99<br>(0.96 to 1.02)
South Asia	1907 to 1911	1.02<br>(1 to 1.03)
South Asia	1912 to 1916	1.05<br>(1.04 to 1.06)
South Asia	1917 to 1921	1.07<br>(1.06 to 1.08)
South Asia	1922 to 1926	1.09<br>(1.08 to 1.1)
South Asia	1927 to 1931	1.1<br>(1.09 to 1.11)
South Asia	1932 to 1936	1.1<br>(1.1 to 1.11)
South Asia	1937 to 1941	1.1<br>(1.09 to 1.1)
South Asia	1942 to 1946	1.08<br>(1.07 to 1.08)
South Asia	1947 to 1951	1.05<br>(1.05 to 1.06)
South Asia	1952 to 1956	1.03<br>(1.02 to 1.03)
South Asia	1957 to 1961	1<br>(1 to 1)
South Asia	1962 to 1966	0.98<br>(0.97 to 0.99)
Southeast Asia	1897 to 1901	1.11<br>(1.03 to 1.2)
Southeast Asia	1902 to 1906	1.18<br>(1.14 to 1.23)
Southeast Asia	1907 to 1911	1.24<br>(1.21 to 1.26)
Southeast Asia	1912 to 1916	1.27<br>(1.25 to 1.29)
Southeast Asia	1917 to 1921	1.29<br>(1.28 to 1.31)
Southeast Asia	1922 to 1926	1.3<br>(1.29 to 1.31)
Southeast Asia	1927 to 1931	1.29<br>(1.28 to 1.3)
Southeast Asia	1932 to 1936	1.26<br>(1.25 to 1.27)

Southeast Asia	1937 to 1941	1.22<br>(1.21 to 1.23)
Southeast Asia	1942 to 1946	1.18<br>(1.17 to 1.19)
Southeast Asia	1947 to 1951	1.13<br>(1.12 to 1.14)
Southeast Asia	1952 to 1956	1.07<br>(1.06 to 1.08)
Southeast Asia	1957 to 1961	1<br>(1 to 1)
Southeast Asia	1962 to 1966	0.94<br>(0.93 to 0.94)
Southern Latin America	1897 to 1901	1.33<br>(1.22 to 1.45)
Southern Latin America	1902 to 1906	1.39<br>(1.34 to 1.44)
Southern Latin America	1907 to 1911	1.43<br>(1.4 to 1.46)
Southern Latin America	1912 to 1916	1.46<br>(1.43 to 1.48)
Southern Latin America	1917 to 1921	1.48<br>(1.46 to 1.5)
Southern Latin America	1922 to 1926	1.48<br>(1.46 to 1.5)
Southern Latin America	1927 to 1931	1.46<br>(1.44 to 1.48)
Southern Latin America	1932 to 1936	1.41<br>(1.4 to 1.43)
Southern Latin America	1937 to 1941	1.34<br>(1.33 to 1.35)
Southern Latin America	1942 to 1946	1.25<br>(1.24 to 1.27)
Southern Latin America	1947 to 1951	1.16<br>(1.15 to 1.18)
Southern Latin America	1952 to 1956	1.08<br>(1.07 to 1.09)
Southern Latin America	1957 to 1961	1<br>(1 to 1)
Southern Latin America	1962 to 1966	0.93<br>(0.92 to 0.95)
Southern Sub-Saharan Africa	1897 to 1901	1.15<br>(0.97 to 1.37)
Southern Sub-Saharan Africa	1902 to 1906	1.15<br>(1.07 to 1.24)
Southern Sub-Saharan Africa	1907 to 1911	1.15<br>(1.1 to 1.21)
Southern Sub-Saharan Africa	1912 to 1916	1.14<br>(1.11 to 1.18)
Southern Sub-Saharan Africa	1917 to 1921	1.14<br>(1.11 to 1.17)
Southern Sub-Saharan Africa	1922 to 1926	1.15<br>(1.12 to 1.18)
Southern Sub-Saharan Africa	1927 to 1931	1.15<br>(1.12 to 1.17)
Southern Sub-Saharan Africa	1932 to 1936	1.15<br>(1.12 to 1.17)
Southern Sub-Saharan Africa	1937 to 1941	1.14<br>(1.12 to 1.16)
Southern Sub-Saharan Africa	1942 to 1946	1.13<br>(1.11 to 1.15)
Southern Sub-Saharan Africa	1947 to 1951	1.1<br>(1.08 to 1.12)
Southern Sub-Saharan Africa	1952 to 1956	1.06<br>(1.04 to 1.08)
Southern Sub-Saharan Africa	1957 to 1961	1<br>(1 to 1)

Southern Sub-Saharan Africa	1962 to 1966	0.93<br>(0.91 to 0.96)
Tropical Latin America	1897 to 1901	1.34<br>(1.21 to 1.48)
Tropical Latin America	1902 to 1906	1.35<br>(1.29 to 1.41)
Tropical Latin America	1907 to 1911	1.36<br>(1.32 to 1.39)
Tropical Latin America	1912 to 1916	1.36<br>(1.33 to 1.39)
Tropical Latin America	1917 to 1921	1.37<br>(1.34 to 1.39)
Tropical Latin America	1922 to 1926	1.36<br>(1.34 to 1.38)
Tropical Latin America	1927 to 1931	1.35<br>(1.33 to 1.37)
Tropical Latin America	1932 to 1936	1.32<br>(1.3 to 1.34)
Tropical Latin America	1937 to 1941	1.27<br>(1.26 to 1.29)
Tropical Latin America	1942 to 1946	1.22<br>(1.2 to 1.23)
Tropical Latin America	1947 to 1951	1.15<br>(1.14 to 1.17)
Tropical Latin America	1952 to 1956	1.08<br>(1.06 to 1.09)
Tropical Latin America	1957 to 1961	1<br>(1 to 1)
Tropical Latin America	1962 to 1966	0.92<br>(0.91 to 0.94)
Western Europe	1897 to 1901	1.51<br>(1.27 to 1.8)
Western Europe	1902 to 1906	1.59<br>(1.46 to 1.72)
Western Europe	1907 to 1911	1.64<br>(1.56 to 1.73)
Western Europe	1912 to 1916	1.69<br>(1.62 to 1.77)
Western Europe	1917 to 1921	1.74<br>(1.67 to 1.81)
Western Europe	1922 to 1926	1.71<br>(1.65 to 1.77)
Western Europe	1927 to 1931	1.66<br>(1.61 to 1.72)
Western Europe	1932 to 1936	1.57<br>(1.52 to 1.63)
Western Europe	1937 to 1941	1.45<br>(1.4 to 1.5)
Western Europe	1942 to 1946	1.34<br>(1.3 to 1.39)
Western Europe	1947 to 1951	1.24<br>(1.19 to 1.28)
Western Europe	1952 to 1956	1.12<br>(1.08 to 1.16)
Western Europe	1957 to 1961	1<br>(1 to 1)
Western Europe	1962 to 1966	0.9<br>(0.86 to 0.95)
Western Sub-Saharan Africa	1897 to 1901	0.84<br>(0.78 to 0.9)
Western Sub-Saharan Africa	1902 to 1906	0.89<br>(0.86 to 0.92)
Western Sub-Saharan Africa	1907 to 1911	0.94<br>(0.93 to 0.96)
Western Sub-Saharan Africa	1912 to 1916	0.99<br>(0.98 to 1)

Western Sub-Saharan Africa	1917 to 1921	1.03<br>(1.02 to 1.04)
Western Sub-Saharan Africa	1922 to 1926	1.06<br>(1.05 to 1.07)
Western Sub-Saharan Africa	1927 to 1931	1.07<br>(1.07 to 1.08)
Western Sub-Saharan Africa	1932 to 1936	1.07<br>(1.06 to 1.08)
Western Sub-Saharan Africa	1937 to 1941	1.06<br>(1.06 to 1.07)
Western Sub-Saharan Africa	1942 to 1946	1.05<br>(1.05 to 1.06)
Western Sub-Saharan Africa	1947 to 1951	1.03<br>(1.03 to 1.04)
Western Sub-Saharan Africa	1952 to 1956	1.02<br>(1.01 to 1.02)
Western Sub-Saharan Africa	1957 to 1961	1<br>(1 to 1)
Western Sub-Saharan Africa	1962 to 1966	0.98<br>(0.97 to 0.98)

Table S8 APC Model Analysis Results of COPD Prevalence Among Adults Aged 55 and Above in the Global, 5 SDI Regions, and 21 GBD Regions

COPD<br>Location	Age	Local drift (%/year)
Global	55 to 59	-0.32<br>(-0.35 to -0.29)
Global	60 to 64	-0.2<br>(-0.22 to -0.18)
Global	65 to 69	-0.08<br>(-0.09 to -0.06)
Global	70 to 74	0.02<br>(0 to 0.04)
Global	75 to 79	0.1<br>(0.08 to 0.12)
Global	80 to 84	0.19<br>(0.17 to 0.21)
Global	85 to 89	0.31<br>(0.27 to 0.34)
Global	90 to 94	0.44<br>(0.38 to 0.5)
Global	95+	0.61<br>(0.48 to 0.74)
High SDI	55 to 59	-0.03<br>(-0.08 to 0.03)
High SDI	60 to 64	0.05<br>(0.01 to 0.09)
High SDI	65 to 69	0.05<br>(0.01 to 0.08)
High SDI	70 to 74	0.02<br>(-0.01 to 0.05)
High SDI	75 to 79	-0.03<br>(-0.06 to 0)
High SDI	80 to 84	0.01<br>(-0.02 to 0.05)
High SDI	85 to 89	0.11<br>(0.06 to 0.15)
High SDI	90 to 94	0.23<br>(0.16 to 0.3)
High SDI	95+	0.38<br>(0.23 to 0.52)
High-middle SDI	55 to 59	-0.65<br>(-0.72 to -0.58)
High-middle SDI	60 to 64	-0.51<br>(-0.56 to -0.46)
High-middle SDI	65 to 69	-0.33<br>(-0.37 to -0.28)
High-middle SDI	70 to 74	-0.18<br>(-0.23 to -0.14)
High-middle SDI	75 to 79	-0.03<br>(-0.08 to 0.02)
High-middle SDI	80 to 84	0.14<br>(0.08 to 0.2)
High-middle SDI	85 to 89	0.34<br>(0.26 to 0.42)
High-middle SDI	90 to 94	0.52<br>(0.37 to 0.67)
High-middle SDI	95+	0.71<br>(0.36 to 1.07)
Middle SDI	55 to 59	-0.47<br>(-0.52 to -0.42)
Middle SDI	60 to 64	-0.31<br>(-0.35 to -0.27)
Middle SDI	65 to 69	-0.12<br>(-0.16 to -0.08)
Middle SDI	70 to 74	0.07<br>(0.03 to 0.1)

Middle SDI	75 to 79	0.24<br>(0.2 to 0.28)
Middle SDI	80 to 84	0.43<br>(0.38 to 0.49)
Middle SDI	85 to 89	0.64<br>(0.56 to 0.73)
Middle SDI	90 to 94	0.89<br>(0.74 to 1.05)
Middle SDI	95+	1.18<br>(0.82 to 1.54)
Low-middle SDI	55 to 59	-0.15<br>(-0.17 to -0.14)
Low-middle SDI	60 to 64	-0.06<br>(-0.07 to -0.05)
Low-middle SDI	65 to 69	0.08<br>(0.07 to 0.09)
Low-middle SDI	70 to 74	0.23<br>(0.22 to 0.24)
Low-middle SDI	75 to 79	0.36<br>(0.35 to 0.37)
Low-middle SDI	80 to 84	0.47<br>(0.45 to 0.48)
Low-middle SDI	85 to 89	0.54<br>(0.52 to 0.56)
Low-middle SDI	90 to 94	0.62<br>(0.59 to 0.66)
Low-middle SDI	95+	0.72<br>(0.64 to 0.8)
Low SDI	55 to 59	-0.05<br>(-0.11 to 0.02)
Low SDI	60 to 64	0.11<br>(0.06 to 0.16)
Low SDI	65 to 69	0.3<br>(0.25 to 0.34)
Low SDI	70 to 74	0.43<br>(0.38 to 0.48)
Low SDI	75 to 79	0.5<br>(0.44 to 0.56)
Low SDI	80 to 84	0.52<br>(0.44 to 0.61)
Low SDI	85 to 89	0.51<br>(0.38 to 0.64)
Low SDI	90 to 94	0.65<br>(0.4 to 0.9)
Low SDI	95+	0.89<br>(0.31 to 1.48)
Andean Latin America	55 to 59	-0.1<br>(-0.13 to -0.07)
Andean Latin America	60 to 64	0.02<br>(0 to 0.05)
Andean Latin America	65 to 69	0.16<br>(0.14 to 0.19)
Andean Latin America	70 to 74	0.33<br>(0.31 to 0.35)
Andean Latin America	75 to 79	0.51<br>(0.48 to 0.53)
Andean Latin America	80 to 84	0.71<br>(0.69 to 0.74)
Andean Latin America	85 to 89	0.95<br>(0.91 to 0.98)
Andean Latin America	90 to 94	1.2<br>(1.15 to 1.26)
Andean Latin America	95+	1.45<br>(1.34 to 1.56)
Australasia	55 to 59	-1.24<br>(-1.3 to -1.17)

Australasia	60 to 64	-1.16<br>(-1.21 to -1.11)
Australasia	65 to 69	-1.08<br>(-1.12 to -1.04)
Australasia	70 to 74	-0.97<br>(-1.01 to -0.93)
Australasia	75 to 79	-0.81<br>(-0.85 to -0.78)
Australasia	80 to 84	-0.63<br>(-0.67 to -0.58)
Australasia	85 to 89	-0.45<br>(-0.51 to -0.39)
Australasia	90 to 94	-0.32<br>(-0.42 to -0.22)
Australasia	95+	-0.23<br>(-0.45 to 0)
Caribbean	55 to 59	0.36<br>(0.33 to 0.39)
Caribbean	60 to 64	0.39<br>(0.37 to 0.41)
Caribbean	65 to 69	0.5<br>(0.48 to 0.52)
Caribbean	70 to 74	0.62<br>(0.6 to 0.64)
Caribbean	75 to 79	0.74<br>(0.72 to 0.76)
Caribbean	80 to 84	0.84<br>(0.82 to 0.87)
Caribbean	85 to 89	0.91<br>(0.88 to 0.94)
Caribbean	90 to 94	0.98<br>(0.93 to 1.03)
Caribbean	95+	1.1<br>(0.98 to 1.22)
Central Asia	55 to 59	-0.56<br>(-0.61 to -0.51)
Central Asia	60 to 64	-0.31<br>(-0.34 to -0.27)
Central Asia	65 to 69	-0.05<br>(-0.08 to -0.01)
Central Asia	70 to 74	0.19<br>(0.16 to 0.23)
Central Asia	75 to 79	0.4<br>(0.36 to 0.44)
Central Asia	80 to 84	0.58<br>(0.53 to 0.63)
Central Asia	85 to 89	0.71<br>(0.65 to 0.78)
Central Asia	90 to 94	0.81<br>(0.71 to 0.91)
Central Asia	95+	0.84<br>(0.61 to 1.06)
Central Europe	55 to 59	-0.29<br>(-0.33 to -0.25)
Central Europe	60 to 64	-0.15<br>(-0.18 to -0.12)
Central Europe	65 to 69	0.01<br>(-0.01 to 0.04)
Central Europe	70 to 74	0.19<br>(0.17 to 0.21)
Central Europe	75 to 79	0.35<br>(0.33 to 0.38)
Central Europe	80 to 84	0.51<br>(0.48 to 0.54)
Central Europe	85 to 89	0.69<br>(0.65 to 0.73)

Central Europe	90 to 94	0.9<br>(0.82 to 0.97)
Central Europe	95+	1.14<br>(0.96 to 1.33)
Central Latin America	55 to 59	-0.2<br>(-0.25 to -0.15)
Central Latin America	60 to 64	-0.07<br>(-0.11 to -0.03)
Central Latin America	65 to 69	0.09<br>(0.05 to 0.12)
Central Latin America	70 to 74	0.25<br>(0.22 to 0.28)
Central Latin America	75 to 79	0.4<br>(0.37 to 0.44)
Central Latin America	80 to 84	0.53<br>(0.49 to 0.57)
Central Latin America	85 to 89	0.66<br>(0.61 to 0.71)
Central Latin America	90 to 94	0.79<br>(0.7 to 0.88)
Central Latin America	95+	0.89<br>(0.71 to 1.08)
Central Sub-Saharan Africa	55 to 59	0.04<br>(0.01 to 0.06)
Central Sub-Saharan Africa	60 to 64	0.13<br>(0.11 to 0.15)
Central Sub-Saharan Africa	65 to 69	0.24<br>(0.22 to 0.26)
Central Sub-Saharan Africa	70 to 74	0.37<br>(0.35 to 0.39)
Central Sub-Saharan Africa	75 to 79	0.51<br>(0.48 to 0.54)
Central Sub-Saharan Africa	80 to 84	0.7<br>(0.65 to 0.74)
Central Sub-Saharan Africa	85 to 89	0.91<br>(0.84 to 0.99)
Central Sub-Saharan Africa	90 to 94	1.1<br>(0.95 to 1.25)
Central Sub-Saharan Africa	95+	1.22<br>(0.88 to 1.55)
East Asia	55 to 59	-0.98<br>(-1.09 to -0.88)
East Asia	60 to 64	-0.79<br>(-0.87 to -0.71)
East Asia	65 to 69	-0.57<br>(-0.64 to -0.5)
East Asia	70 to 74	-0.31<br>(-0.38 to -0.24)
East Asia	75 to 79	-0.04<br>(-0.12 to 0.04)
East Asia	80 to 84	0.25<br>(0.14 to 0.36)
East Asia	85 to 89	0.59<br>(0.41 to 0.78)
East Asia	90 to 94	1<br>(0.58 to 1.41)
East Asia	95+	1.34<br>(0.12 to 2.58)
Eastern Europe	55 to 59	-0.55<br>(-0.7 to -0.41)
Eastern Europe	60 to 64	-0.52<br>(-0.62 to -0.41)
Eastern Europe	65 to 69	-0.54<br>(-0.63 to -0.44)
Eastern Europe	70 to 74	-0.57<br>(-0.66 to -0.47)

Eastern Europe	75 to 79	-0.6<br>(-0.7 to -0.49)
Eastern Europe	80 to 84	-0.61<br>(-0.73 to -0.49)
Eastern Europe	85 to 89	-0.6<br>(-0.76 to -0.43)
Eastern Europe	90 to 94	-0.5<br>(-0.8 to -0.21)
Eastern Europe	95+	-0.36<br>(-1.06 to 0.34)
Eastern Sub-Saharan Africa	55 to 59	-0.11<br>(-0.13 to -0.09)
Eastern Sub-Saharan Africa	60 to 64	0.01<br>(-0.01 to 0.02)
Eastern Sub-Saharan Africa	65 to 69	0.11<br>(0.09 to 0.12)
Eastern Sub-Saharan Africa	70 to 74	0.2<br>(0.18 to 0.22)
Eastern Sub-Saharan Africa	75 to 79	0.27<br>(0.25 to 0.29)
Eastern Sub-Saharan Africa	80 to 84	0.34<br>(0.31 to 0.37)
Eastern Sub-Saharan Africa	85 to 89	0.42<br>(0.37 to 0.47)
Eastern Sub-Saharan Africa	90 to 94	0.52<br>(0.42 to 0.61)
Eastern Sub-Saharan Africa	95+	0.67<br>(0.43 to 0.91)
High-income Asia Pacific	55 to 59	-0.17<br>(-0.3 to -0.04)
High-income Asia Pacific	60 to 64	-0.28<br>(-0.37 to -0.19)
High-income Asia Pacific	65 to 69	-0.44<br>(-0.51 to -0.36)
High-income Asia Pacific	70 to 74	-0.42<br>(-0.48 to -0.35)
High-income Asia Pacific	75 to 79	-0.34<br>(-0.4 to -0.27)
High-income Asia Pacific	80 to 84	-0.31<br>(-0.38 to -0.23)
High-income Asia Pacific	85 to 89	-0.28<br>(-0.37 to -0.19)
High-income Asia Pacific	90 to 94	-0.22<br>(-0.36 to -0.07)
High-income Asia Pacific	95+	-0.12<br>(-0.44 to 0.2)
High-income North America	55 to 59	0.17<br>(0.03 to 0.32)
High-income North America	60 to 64	0.25<br>(0.15 to 0.36)
High-income North America	65 to 69	0.34<br>(0.25 to 0.43)
High-income North America	70 to 74	0.41<br>(0.33 to 0.49)
High-income North America	75 to 79	0.49<br>(0.41 to 0.57)
High-income North America	80 to 84	0.57<br>(0.47 to 0.67)
High-income North America	85 to 89	0.64<br>(0.51 to 0.77)
High-income North America	90 to 94	0.68<br>(0.47 to 0.89)
High-income North America	95+	0.71<br>(0.28 to 1.14)
North Africa and Middle East	55 to 59	0.15<br>(0.13 to 0.18)

North Africa and Middle East	60 to 64	0.32<br>(0.3 to 0.34)
North Africa and Middle East	65 to 69	0.52<br>(0.51 to 0.54)
North Africa and Middle East	70 to 74	0.74<br>(0.72 to 0.76)
North Africa and Middle East	75 to 79	0.92<br>(0.9 to 0.94)
North Africa and Middle East	80 to 84	1.03<br>(1 to 1.06)
North Africa and Middle East	85 to 89	1.15<br>(1.11 to 1.19)
North Africa and Middle East	90 to 94	1.29<br>(1.23 to 1.36)
North Africa and Middle East	95+	1.5<br>(1.36 to 1.63)
Oceania	55 to 59	-0.23<br>(-0.3 to -0.17)
Oceania	60 to 64	-0.26<br>(-0.31 to -0.2)
Oceania	65 to 69	-0.3<br>(-0.35 to -0.25)
Oceania	70 to 74	-0.32<br>(-0.38 to -0.27)
Oceania	75 to 79	-0.31<br>(-0.37 to -0.25)
Oceania	80 to 84	-0.24<br>(-0.33 to -0.15)
Oceania	85 to 89	-0.15<br>(-0.29 to 0)
Oceania	90 to 94	-0.08<br>(-0.37 to 0.22)
Oceania	95+	-0.01<br>(-0.7 to 0.69)
South Asia	55 to 59	-0.11<br>(-0.12 to -0.09)
South Asia	60 to 64	-0.02<br>(-0.04 to -0.01)
South Asia	65 to 69	0.07<br>(0.06 to 0.08)
South Asia	70 to 74	0.17<br>(0.15 to 0.18)
South Asia	75 to 79	0.27<br>(0.25 to 0.28)
South Asia	80 to 84	0.38<br>(0.36 to 0.4)
South Asia	85 to 89	0.49<br>(0.46 to 0.52)
South Asia	90 to 94	0.58<br>(0.53 to 0.63)
South Asia	95+	0.62<br>(0.5 to 0.74)
Southeast Asia	55 to 59	-0.26<br>(-0.28 to -0.23)
Southeast Asia	60 to 64	-0.17<br>(-0.19 to -0.15)
Southeast Asia	65 to 69	-0.1<br>(-0.12 to -0.08)
Southeast Asia	70 to 74	-0.03<br>(-0.05 to -0.01)
Southeast Asia	75 to 79	0.07<br>(0.05 to 0.09)
Southeast Asia	80 to 84	0.19<br>(0.16 to 0.22)
Southeast Asia	85 to 89	0.33<br>(0.3 to 0.37)

Southeast Asia	90 to 94	0.49 (0.43 to 0.56)
Southeast Asia	95+	0.72 (0.57 to 0.86)
Southern Latin America	55 to 59	-0.34 (-0.4 to -0.28)
Southern Latin America	60 to 64	-0.28 (-0.33 to -0.24)
Southern Latin America	65 to 69	-0.21 (-0.25 to -0.18)
Southern Latin America	70 to 74	-0.13 (-0.16 to -0.09)
Southern Latin America	75 to 79	0 (-0.04 to 0.03)
Southern Latin America	80 to 84	0.15 (0.1 to 0.19)
Southern Latin America	85 to 89	0.27 (0.21 to 0.33)
Southern Latin America	90 to 94	0.36 (0.26 to 0.45)
Southern Latin America	95+	0.47 (0.26 to 0.68)
Southern Sub-Saharan Africa	55 to 59	-0.28 (-0.36 to -0.21)
Southern Sub-Saharan Africa	60 to 64	-0.08 (-0.14 to -0.02)
Southern Sub-Saharan Africa	65 to 69	0.08 (0.02 to 0.14)
Southern Sub-Saharan Africa	70 to 74	0.17 (0.11 to 0.23)
Southern Sub-Saharan Africa	75 to 79	0.22 (0.16 to 0.29)
Southern Sub-Saharan Africa	80 to 84	0.22 (0.14 to 0.3)
Southern Sub-Saharan Africa	85 to 89	0.16 (0.05 to 0.28)
Southern Sub-Saharan Africa	90 to 94	0.14 (-0.05 to 0.32)
Southern Sub-Saharan Africa	95+	0.13 (-0.28 to 0.54)
Tropical Latin America	55 to 59	-0.37 (-0.41 to -0.32)
Tropical Latin America	60 to 64	-0.25 (-0.29 to -0.22)
Tropical Latin America	65 to 69	-0.17 (-0.2 to -0.14)
Tropical Latin America	70 to 74	-0.11 (-0.14 to -0.08)
Tropical Latin America	75 to 79	-0.06 (-0.09 to -0.03)
Tropical Latin America	80 to 84	0 (-0.04 to 0.03)
Tropical Latin America	85 to 89	0.04 (-0.01 to 0.1)
Tropical Latin America	90 to 94	0.09 (0 to 0.18)
Tropical Latin America	95+	0.14 (-0.06 to 0.34)
Western Europe	55 to 59	-0.32 (-0.37 to -0.27)
Western Europe	60 to 64	-0.24 (-0.28 to -0.2)
Western Europe	65 to 69	-0.16 (-0.19 to -0.13)
Western Europe	70 to 74	-0.07 (-0.1 to -0.04)

Western Europe	75 to 79	-0.02<br>(-0.05 to 0.01)
Western Europe	80 to 84	0.08<br>(0.05 to 0.11)
Western Europe	85 to 89	0.21<br>(0.17 to 0.24)
Western Europe	90 to 94	0.32<br>(0.26 to 0.38)
Western Europe	95+	0.41<br>(0.28 to 0.54)
Western Sub-Saharan Africa	55 to 59	0.27<br>(0.25 to 0.3)
Western Sub-Saharan Africa	60 to 64	0.35<br>(0.33 to 0.37)
Western Sub-Saharan Africa	65 to 69	0.44<br>(0.42 to 0.46)
Western Sub-Saharan Africa	70 to 74	0.58<br>(0.55 to 0.6)
Western Sub-Saharan Africa	75 to 79	0.73<br>(0.7 to 0.76)
Western Sub-Saharan Africa	80 to 84	0.89<br>(0.85 to 0.93)
Western Sub-Saharan Africa	85 to 89	1.05<br>(0.99 to 1.1)
Western Sub-Saharan Africa	90 to 94	1.17<br>(1.07 to 1.28)
Western Sub-Saharan Africa	95+	1.29<br>(1.06 to 1.51)

COPD<br>Location	Age	Prevalence rate (per 100,000 population)
Global	55 to 59	4881.21<br>(4855.26 to 4907.3)
Global	60 to 64	6692.43<br>(6658.26 to 6726.78)
Global	65 to 69	9754.44<br>(9695.3 to 9813.95)
Global	70 to 74	13715.13<br>(13630.06 to 13800.74)
Global	75 to 79	18412.73<br>(18294.97 to 18531.24)
Global	80 to 84	23626.8<br>(23467.99 to 23786.68)
Global	85 to 89	29619.98<br>(29401.43 to 29840.15)
Global	90 to 94	35582.87<br>(35264.45 to 35904.17)
Global	95+	41801.14<br>(41261.68 to 42347.66)
High SDI	55 to 59	5082.76<br>(5033.14 to 5132.87)
High SDI	60 to 64	7125.55<br>(7059.14 to 7192.6)
High SDI	65 to 69	10696.65<br>(10580.03 to 10814.56)
High SDI	70 to 74	15135.73<br>(14968.14 to 15305.19)
High SDI	75 to 79	20408.86<br>(20178.24 to 20642.11)
High SDI	80 to 84	26299.23<br>(25993.24 to 26608.82)
High SDI	85 to 89	32691.89<br>(32291.09 to 33097.66)
High SDI	90 to 94	38537.38<br>(38008.57 to 39073.54)

High SDI	95+	43936.41 (43165.55 to 44721.03)
High-middle SDI	55 to 59	4630.66 (4570.42 to 4691.68)
High-middle SDI	60 to 64	6265.1 (6186.57 to 6344.63)
High-middle SDI	65 to 69	8855.3 (8724.65 to 8987.91)
High-middle SDI	70 to 74	12295.75 (12111.16 to 12483.15)
High-middle SDI	75 to 79	16415.98 (16163.4 to 16672.51)
High-middle SDI	80 to 84	21101.87 (20762.38 to 21446.92)
High-middle SDI	85 to 89	26623.55 (26153.8 to 27101.74)
High-middle SDI	90 to 94	32314.6 (31614.6 to 33030.09)
High-middle SDI	95+	38689.74 (37404.36 to 40019.29)
Middle SDI	55 to 59	4678.28 (4633.8 to 4723.2)
Middle SDI	60 to 64	6413.18 (6354.6 to 6472.3)
Middle SDI	65 to 69	9310.65 (9208.49 to 9413.95)
Middle SDI	70 to 74	13217.95 (13068.78 to 13368.82)
Middle SDI	75 to 79	17845.82 (17636.72 to 18057.39)
Middle SDI	80 to 84	22766.9 (22482.17 to 23055.23)
Middle SDI	85 to 89	28229.3 (27827.04 to 28637.36)
Middle SDI	90 to 94	33614.28 (32986.72 to 34253.77)
Middle SDI	95+	40026.13 (38850.93 to 41236.88)
Low-middle SDI	55 to 59	5359.2 (5345.88 to 5372.55)
Low-middle SDI	60 to 64	7260.9 (7243.51 to 7278.32)
Low-middle SDI	65 to 69	10697.86 (10667.21 to 10728.6)
Low-middle SDI	70 to 74	15058.52 (15014.02 to 15103.15)
Low-middle SDI	75 to 79	20051.65 (19989.66 to 20113.84)
Low-middle SDI	80 to 84	25236.92 (25152.57 to 25321.55)
Low-middle SDI	85 to 89	31421.6 (31300.18 to 31543.49)
Low-middle SDI	90 to 94	38271.18 (38077.02 to 38466.34)
Low-middle SDI	95+	46586.14 (46229.67 to 46945.35)
Low SDI	55 to 59	4818.36 (4761.51 to 4875.9)
Low SDI	60 to 64	6509.61 (6435.11 to 6584.96)
Low SDI	65 to 69	9265.11 (9137.68 to 9394.32)
Low SDI	70 to 74	12536.23 (12357.04 to 12718.02)
Low SDI	75 to 79	16225.28 (15980.21 to 16474.11)

Low SDI	80 to 84	19974.15 (19640.69 to 20313.27)
Low SDI	85 to 89	23929.23 (23441.06 to 24427.57)
Low SDI	90 to 94	27838.86 (27005.17 to 28698.28)
Low SDI	95+	32008.11 (30272.34 to 33843.41)
Andean Latin America	55 to 59	2940.24 (2922.82 to 2957.77)
Andean Latin America	60 to 64	4300.24 (4276.2 to 4324.42)
Andean Latin America	65 to 69	6160.51 (6118.62 to 6202.69)
Andean Latin America	70 to 74	8885.9 (8823.83 to 8948.41)
Andean Latin America	75 to 79	12831.81 (12739.4 to 12924.89)
Andean Latin America	80 to 84	18234.01 (18096.94 to 18372.12)
Andean Latin America	85 to 89	24963.88 (24762.28 to 25167.12)
Andean Latin America	90 to 94	32212.6 (31914.26 to 32513.72)
Andean Latin America	95+	39298.75 (38830.9 to 39772.24)
Australasia	55 to 59	3347.44 (3305.15 to 3390.26)
Australasia	60 to 64	4834.53 (4776.87 to 4892.89)
Australasia	65 to 69	6883.52 (6787.85 to 6980.54)
Australasia	70 to 74	9324.87 (9193.4 to 9458.22)
Australasia	75 to 79	11843.2 (11672.89 to 12015.99)
Australasia	80 to 84	14181.98 (13972.28 to 14394.83)
Australasia	85 to 89	16043.86 (15793.33 to 16298.36)
Australasia	90 to 94	16919.83 (16618.73 to 17226.38)
Australasia	95+	16921.85 (16500.06 to 17354.42)
Caribbean	55 to 59	3805.44 (3784.81 to 3826.18)
Caribbean	60 to 64	5445.87 (5417.58 to 5474.32)
Caribbean	65 to 69	7553.18 (7506.19 to 7600.47)
Caribbean	70 to 74	10409.76 (10343.13 to 10476.82)
Caribbean	75 to 79	14230.78 (14136.45 to 14325.73)
Caribbean	80 to 84	19227.74 (19094.25 to 19362.16)
Caribbean	85 to 89	25377.16 (25187.37 to 25568.38)
Caribbean	90 to 94	32491.52 (32213.83 to 32771.61)
Caribbean	95+	40663.59 (40239.85 to 41091.79)
Central Asia	55 to 59	4411.3 (4371.31 to 4451.66)
Central Asia	60 to 64	6028.69 (5978.56 to 6079.25)

Central Asia	65 to 69	8370.43<br>(8302.4 to 8439.01)
Central Asia	70 to 74	11622.58<br>(11510.31 to 11735.96)
Central Asia	75 to 79	15756.5<br>(15598.75 to 15915.85)
Central Asia	80 to 84	20705.17<br>(20481.44 to 20931.34)
Central Asia	85 to 89	26041.2<br>(25711.69 to 26374.92)
Central Asia	90 to 94	31088.46<br>(30568.5 to 31617.26)
Central Asia	95+	35686.45<br>(34752.22 to 36645.81)
Central Europe	55 to 59	4883.83<br>(4849.61 to 4918.28)
Central Europe	60 to 64	6717.32<br>(6671.9 to 6763.06)
Central Europe	65 to 69	9400.25<br>(9326.55 to 9474.52)
Central Europe	70 to 74	13082.61<br>(12978.69 to 13187.36)
Central Europe	75 to 79	17574.02<br>(17431.64 to 17717.56)
Central Europe	80 to 84	22568.58<br>(22378.21 to 22760.58)
Central Europe	85 to 89	27790.23<br>(27533.94 to 28048.9)
Central Europe	90 to 94	32936.58<br>(32560.48 to 33317.03)
Central Europe	95+	38463.02<br>(37785.64 to 39152.55)
Central Latin America	55 to 59	3873.01<br>(3838.67 to 3907.66)
Central Latin America	60 to 64	5539.71<br>(5493.1 to 5586.71)
Central Latin America	65 to 69	7947.4<br>(7865.66 to 8029.99)
Central Latin America	70 to 74	11622.09<br>(11499.21 to 11746.29)
Central Latin America	75 to 79	17029.8<br>(16843.88 to 17217.78)
Central Latin America	80 to 84	24541.1<br>(24260.85 to 24824.59)
Central Latin America	85 to 89	34098.84<br>(33680.63 to 34522.25)
Central Latin America	90 to 94	43981.51<br>(43366.79 to 44604.95)
Central Latin America	95+	53722.82<br>(52808.25 to 54653.23)
Central Sub-Saharan Africa	55 to 59	4122.38<br>(4102.3 to 4142.55)
Central Sub-Saharan Africa	60 to 64	5630.74<br>(5603.95 to 5657.66)
Central Sub-Saharan Africa	65 to 69	7435<br>(7391 to 7479.26)
Central Sub-Saharan Africa	70 to 74	9707.4<br>(9646.86 to 9768.31)
Central Sub-Saharan Africa	75 to 79	12605.09<br>(12520.88 to 12689.87)
Central Sub-Saharan Africa	80 to 84	16284.43<br>(16161.85 to 16407.94)
Central Sub-Saharan Africa	85 to 89	20144.44<br>(19951.67 to 20339.08)
Central Sub-Saharan Africa	90 to 94	23280.15<br>(22925.26 to 23640.54)

Central Sub-Saharan Africa	95+	25762.79 (24961.09 to 26590.24)
East Asia	55 to 59	4718.76 (4626.14 to 4813.25)
East Asia	60 to 64	6401.4 (6280.22 to 6524.92)
East Asia	65 to 69	9410.04 (9200.26 to 9624.61)
East Asia	70 to 74	13338.98 (13034.85 to 13650.2)
East Asia	75 to 79	17667.43 (17251.65 to 18093.23)
East Asia	80 to 84	21804.89 (21259.04 to 22364.75)
East Asia	85 to 89	25785.62 (25042.27 to 26551.04)
East Asia	90 to 94	28531.67 (27386.51 to 29724.7)
East Asia	95+	32090.23 (29688.35 to 34686.43)
Eastern Europe	55 to 59	4530.66 (4408.39 to 4656.32)
Eastern Europe	60 to 64	5961.82 (5805.46 to 6122.39)
Eastern Europe	65 to 69	7820.63 (7581.89 to 8066.89)
Eastern Europe	70 to 74	10138.5 (9824.58 to 10462.45)
Eastern Europe	75 to 79	12747.38 (12342.65 to 13165.37)
Eastern Europe	80 to 84	15640.62 (15120.85 to 16178.26)
Eastern Europe	85 to 89	18607.28 (17915.81 to 19325.44)
Eastern Europe	90 to 94	21448.38 (20426.22 to 22521.7)
Eastern Europe	95+	24077.47 (22070.09 to 26267.43)
Eastern Sub-Saharan Africa	55 to 59	3573.24 (3559.89 to 3586.64)
Eastern Sub-Saharan Africa	60 to 64	4722.01 (4704.9 to 4739.19)
Eastern Sub-Saharan Africa	65 to 69	6036.34 (6009.41 to 6063.39)
Eastern Sub-Saharan Africa	70 to 74	7654.85 (7619 to 7690.87)
Eastern Sub-Saharan Africa	75 to 79	9566.06 (9518.29 to 9614.07)
Eastern Sub-Saharan Africa	80 to 84	11557.96 (11493.12 to 11623.17)
Eastern Sub-Saharan Africa	85 to 89	13032.57 (12939.72 to 13126.08)
Eastern Sub-Saharan Africa	90 to 94	13715.41 (13561.63 to 13870.94)
Eastern Sub-Saharan Africa	95+	13980.19 (13674.09 to 14293.13)
High-income Asia Pacific	55 to 59	2991.74 (2920.36 to 3064.86)
High-income Asia Pacific	60 to 64	4294.76 (4196.31 to 4395.51)
High-income Asia Pacific	65 to 69	6211.74 (6049.99 to 6377.82)
High-income Asia Pacific	70 to 74	8838.63 (8606.81 to 9076.7)
High-income Asia Pacific	75 to 79	12408.11 (12077.93 to 12747.31)

High-income Asia Pacific	80 to 84	17185.94 (16718.42 to 17666.54)
High-income Asia Pacific	85 to 89	23174.75 (22521.07 to 23847.41)
High-income Asia Pacific	90 to 94	29273.01 (28387.48 to 30186.15)
High-income Asia Pacific	95+	35228.46 (34010.28 to 36490.27)
High-income North America	55 to 59	6608.23 (6443.94 to 6776.71)
High-income North America	60 to 64	9544.85 (9319.24 to 9775.93)
High-income North America	65 to 69	15752.82 (15312.2 to 16206.11)
High-income North America	70 to 74	23024.94 (22366.37 to 23702.89)
High-income North America	75 to 79	30863.51 (29957.85 to 31796.54)
High-income North America	80 to 84	37498.61 (36359.66 to 38673.22)
High-income North America	85 to 89	42140.5 (40777.22 to 43549.35)
High-income North America	90 to 94	44507.62 (42846.47 to 46233.17)
High-income North America	95+	45646.13 (43272.97 to 48149.44)
North Africa and Middle East	55 to 59	4982.21 (4961.18 to 5003.33)
North Africa and Middle East	60 to 64	6744.78 (6717.19 to 6772.48)
North Africa and Middle East	65 to 69	9192.91 (9146.31 to 9239.74)
North Africa and Middle East	70 to 74	12709.53 (12642.45 to 12776.97)
North Africa and Middle East	75 to 79	17722.66 (17624.75 to 17821.12)
North Africa and Middle East	80 to 84	24633.5 (24487.71 to 24780.15)
North Africa and Middle East	85 to 89	33642.66 (33415.48 to 33871.39)
North Africa and Middle East	90 to 94	43980.27 (43593.86 to 44370.11)
North Africa and Middle East	95+	56257.77 (55561.41 to 56962.85)
Oceania	55 to 59	5096.44 (5035.34 to 5158.28)
Oceania	60 to 64	6634.37 (6556.09 to 6713.59)
Oceania	65 to 69	9470.88 (9333.78 to 9610.01)
Oceania	70 to 74	13042.38 (12845.49 to 13242.3)
Oceania	75 to 79	16879.38 (16609.48 to 17153.66)
Oceania	80 to 84	20276.74 (19917.06 to 20642.92)
Oceania	85 to 89	23889.86 (23371.68 to 24419.53)
Oceania	90 to 94	26306.44 (25454.23 to 27187.19)
Oceania	95+	28361.76 (26716.59 to 30108.23)
South Asia	55 to 59	5936.91 (5918.37 to 5955.51)
South Asia	60 to 64	8032.83 (8008.76 to 8056.97)

South Asia	65 to 69	12214.65<br>(12170.85 to 12258.6)
South Asia	70 to 74	17454.9<br>(17390.29 to 17519.75)
South Asia	75 to 79	23249.03<br>(23158.83 to 23339.58)
South Asia	80 to 84	28758.81<br>(28637.7 to 28880.44)
South Asia	85 to 89	35066.12<br>(34893.56 to 35239.53)
South Asia	90 to 94	41822.94<br>(41545.01 to 42102.74)
South Asia	95+	50019.76<br>(49463.21 to 50582.57)
Southeast Asia	55 to 59	4240.77<br>(4222.16 to 4259.46)
Southeast Asia	60 to 64	5697.14<br>(5673.04 to 5721.33)
Southeast Asia	65 to 69	7718.36<br>(7678.06 to 7758.87)
Southeast Asia	70 to 74	10499.84<br>(10442.93 to 10557.05)
Southeast Asia	75 to 79	14106.23<br>(14026.28 to 14186.63)
Southeast Asia	80 to 84	18564.97<br>(18451.83 to 18678.8)
Southeast Asia	85 to 89	23535.15<br>(23370.33 to 23701.13)
Southeast Asia	90 to 94	28114.7<br>(27856.1 to 28375.71)
Southeast Asia	95+	32206.31<br>(31746.85 to 32672.41)
Southern Latin America	55 to 59	2902.5<br>(2869.54 to 2935.84)
Southern Latin America	60 to 64	4236.82<br>(4191.1 to 4283.03)
Southern Latin America	65 to 69	5995.35<br>(5919.38 to 6072.3)
Southern Latin America	70 to 74	8297.47<br>(8190.63 to 8405.71)
Southern Latin America	75 to 79	11201.85<br>(11054.25 to 11351.41)
Southern Latin America	80 to 84	14746.57<br>(14545.61 to 14950.31)
Southern Latin America	85 to 89	18608.96<br>(18339.23 to 18882.66)
Southern Latin America	90 to 94	21930<br>(21563.42 to 22302.81)
Southern Latin America	95+	24333.7<br>(23788.81 to 24891.06)
Southern Sub-Saharan Africa	55 to 59	4536.84<br>(4473.91 to 4600.66)
Southern Sub-Saharan Africa	60 to 64	6055.29<br>(5973.32 to 6138.38)
Southern Sub-Saharan Africa	65 to 69	8072.69<br>(7940.19 to 8207.4)
Southern Sub-Saharan Africa	70 to 74	10666.27<br>(10484.32 to 10851.37)
Southern Sub-Saharan Africa	75 to 79	13915.28<br>(13665.96 to 14169.15)
Southern Sub-Saharan Africa	80 to 84	18026.19<br>(17681.84 to 18377.25)
Southern Sub-Saharan Africa	85 to 89	22597.16<br>(22101.75 to 23103.66)
Southern Sub-Saharan Africa	90 to 94	26989.54<br>(26173.03 to 27831.52)

Southern Sub-Saharan Africa	95+	30381.75 (28624.84 to 32246.49)
Tropical Latin America	55 to 59	4923.83 (4884.69 to 4963.27)
Tropical Latin America	60 to 64	6683.27 (6632.34 to 6734.59)
Tropical Latin America	65 to 69	9314.62 (9228.36 to 9401.69)
Tropical Latin America	70 to 74	13070.1 (12945.48 to 13195.93)
Tropical Latin America	75 to 79	17903.99 (17727.03 to 18082.72)
Tropical Latin America	80 to 84	23751.46 (23504.5 to 24001.01)
Tropical Latin America	85 to 89	30674.25 (30326.95 to 31025.52)
Tropical Latin America	90 to 94	37788.49 (37288.23 to 38295.47)
Tropical Latin America	95+	44657.35 (43899.99 to 45427.78)
Western Europe	55 to 59	5052.2 (5000.63 to 5104.29)
Western Europe	60 to 64	6833 (6766.16 to 6900.5)
Western Europe	65 to 69	9497.44 (9390.46 to 9605.64)
Western Europe	70 to 74	13173.01 (13023.51 to 13324.23)
Western Europe	75 to 79	18054.12 (17846.57 to 18264.1)
Western Europe	80 to 84	24696.58 (24406.81 to 24989.78)
Western Europe	85 to 89	33481.46 (33073.13 to 33894.82)
Western Europe	90 to 94	42818.97 (42249.47 to 43396.16)
Western Europe	95+	52486.96 (51623.4 to 53364.96)
Western Sub-Saharan Africa	55 to 59	3760.01 (3741.23 to 3778.88)
Western Sub-Saharan Africa	60 to 64	5040.01 (5015.4 to 5064.74)
Western Sub-Saharan Africa	65 to 69	6482.24 (6443.01 to 6521.71)
Western Sub-Saharan Africa	70 to 74	8241.23 (8188.84 to 8293.95)
Western Sub-Saharan Africa	75 to 79	10488.77 (10417.93 to 10560.09)
Western Sub-Saharan Africa	80 to 84	13366.72 (13266.82 to 13467.37)
Western Sub-Saharan Africa	85 to 89	16556.95 (16407.05 to 16708.21)
Western Sub-Saharan Africa	90 to 94	19509.79 (19255.67 to 19767.26)
Western Sub-Saharan Africa	95+	21988.43 (21486.79 to 22501.79)

COPD Location	Period	Prevalence rate ratio
Global	1992 to 1996	0.98 (0.97 to 0.98)
Global	1997 to 2001	0.98 (0.98 to 0.98)
Global	2002 to 2006	0.99 (0.99 to 0.99)

Global	2007 to 2011	0.99<br>(0.99 to 1)
Global	2012 to 2016	1<br>(1 to 1)
Global	2017 to 2021	1<br>(1 to 1.01)
High SDI	1992 to 1996	0.97<br>(0.97 to 0.98)
High SDI	1997 to 2001	0.99<br>(0.98 to 0.99)
High SDI	2002 to 2006	1.01<br>(1.01 to 1.02)
High SDI	2007 to 2011	1.01<br>(1 to 1.01)
High SDI	2012 to 2016	1<br>(1 to 1)
High SDI	2017 to 2021	0.99<br>(0.99 to 1)
High-middle SDI	1992 to 1996	1.01<br>(1 to 1.02)
High-middle SDI	1997 to 2001	1<br>(0.99 to 1.01)
High-middle SDI	2002 to 2006	0.99<br>(0.98 to 1)
High-middle SDI	2007 to 2011	0.99<br>(0.98 to 1)
High-middle SDI	2012 to 2016	1<br>(1 to 1)
High-middle SDI	2017 to 2021	1<br>(0.99 to 1.01)
Middle SDI	1992 to 1996	0.95<br>(0.94 to 0.96)
Middle SDI	1997 to 2001	0.95<br>(0.94 to 0.96)
Middle SDI	2002 to 2006	0.96<br>(0.95 to 0.97)
Middle SDI	2007 to 2011	0.97<br>(0.97 to 0.98)
Middle SDI	2012 to 2016	1<br>(1 to 1)
Middle SDI	2017 to 2021	1.02<br>(1.01 to 1.02)
Low-middle SDI	1992 to 1996	0.94<br>(0.94 to 0.94)
Low-middle SDI	1997 to 2001	0.95<br>(0.95 to 0.95)
Low-middle SDI	2002 to 2006	0.97<br>(0.96 to 0.97)
Low-middle SDI	2007 to 2011	0.98<br>(0.98 to 0.99)
Low-middle SDI	2012 to 2016	1<br>(1 to 1)
Low-middle SDI	2017 to 2021	1.02<br>(1.01 to 1.02)
Low SDI	1992 to 1996	0.92<br>(0.91 to 0.94)
Low SDI	1997 to 2001	0.94<br>(0.92 to 0.95)
Low SDI	2002 to 2006	0.95<br>(0.94 to 0.96)
Low SDI	2007 to 2011	0.98<br>(0.97 to 0.99)
Low SDI	2012 to 2016	1<br>(1 to 1)
Low SDI	2017 to 2021	1.03<br>(1.02 to 1.04)

Andean Latin America	1992 to 1996	0.89<br>(0.88 to 0.89)
Andean Latin America	1997 to 2001	0.94<br>(0.94 to 0.94)
Andean Latin America	2002 to 2006	0.96<br>(0.96 to 0.97)
Andean Latin America	2007 to 2011	0.97<br>(0.97 to 0.98)
Andean Latin America	2012 to 2016	1<br>(1 to 1)
Andean Latin America	2017 to 2021	1.04<br>(1.04 to 1.04)
Australasia	1992 to 1996	1.15<br>(1.14 to 1.16)
Australasia	1997 to 2001	1.14<br>(1.13 to 1.15)
Australasia	2002 to 2006	1.08<br>(1.08 to 1.09)
Australasia	2007 to 2011	1.04<br>(1.04 to 1.05)
Australasia	2012 to 2016	1<br>(1 to 1)
Australasia	2017 to 2021	0.96<br>(0.95 to 0.96)
Caribbean	1992 to 1996	0.85<br>(0.85 to 0.86)
Caribbean	1997 to 2001	0.9<br>(0.9 to 0.91)
Caribbean	2002 to 2006	0.94<br>(0.94 to 0.94)
Caribbean	2007 to 2011	0.98<br>(0.97 to 0.98)
Caribbean	2012 to 2016	1<br>(1 to 1)
Caribbean	2017 to 2021	1.02<br>(1.02 to 1.03)
Central Asia	1992 to 1996	0.98<br>(0.97 to 0.99)
Central Asia	1997 to 2001	0.94<br>(0.93 to 0.95)
Central Asia	2002 to 2006	0.94<br>(0.93 to 0.95)
Central Asia	2007 to 2011	0.96<br>(0.95 to 0.96)
Central Asia	2012 to 2016	1<br>(1 to 1)
Central Asia	2017 to 2021	1.05<br>(1.04 to 1.05)
Central Europe	1992 to 1996	0.94<br>(0.93 to 0.94)
Central Europe	1997 to 2001	0.94<br>(0.93 to 0.94)
Central Europe	2002 to 2006	0.95<br>(0.95 to 0.96)
Central Europe	2007 to 2011	0.97<br>(0.97 to 0.98)
Central Europe	2012 to 2016	1<br>(1 to 1)
Central Europe	2017 to 2021	1.02<br>(1.02 to 1.03)
Central Latin America	1992 to 1996	0.92<br>(0.91 to 0.93)
Central Latin America	1997 to 2001	0.96<br>(0.95 to 0.97)
Central Latin America	2002 to 2006	0.97<br>(0.96 to 0.97)

Central Latin America	2007 to 2011	0.98<br>(0.98 to 0.99)
Central Latin America	2012 to 2016	1<br>(1 to 1)
Central Latin America	2017 to 2021	1.02<br>(1.02 to 1.03)
Central Sub-Saharan Africa	1992 to 1996	0.91<br>(0.9 to 0.91)
Central Sub-Saharan Africa	1997 to 2001	0.92<br>(0.91 to 0.93)
Central Sub-Saharan Africa	2002 to 2006	0.94<br>(0.93 to 0.94)
Central Sub-Saharan Africa	2007 to 2011	0.97<br>(0.96 to 0.97)
Central Sub-Saharan Africa	2012 to 2016	1<br>(1 to 1)
Central Sub-Saharan Africa	2017 to 2021	1.04<br>(1.04 to 1.05)
East Asia	1992 to 1996	0.99<br>(0.97 to 1.02)
East Asia	1997 to 2001	0.98<br>(0.96 to 1)
East Asia	2002 to 2006	0.97<br>(0.95 to 0.99)
East Asia	2007 to 2011	0.98<br>(0.96 to 0.99)
East Asia	2012 to 2016	1<br>(1 to 1)
East Asia	2017 to 2021	0.99<br>(0.98 to 1.01)
Eastern Europe	1992 to 1996	1.14<br>(1.12 to 1.17)
Eastern Europe	1997 to 2001	1.1<br>(1.08 to 1.12)
Eastern Europe	2002 to 2006	1.08<br>(1.06 to 1.1)
Eastern Europe	2007 to 2011	1.04<br>(1.02 to 1.06)
Eastern Europe	2012 to 2016	1<br>(1 to 1)
Eastern Europe	2017 to 2021	1.01<br>(0.99 to 1.03)
Eastern Sub-Saharan Africa	1992 to 1996	0.98<br>(0.98 to 0.99)
Eastern Sub-Saharan Africa	1997 to 2001	0.98<br>(0.97 to 0.98)
Eastern Sub-Saharan Africa	2002 to 2006	0.97<br>(0.96 to 0.97)
Eastern Sub-Saharan Africa	2007 to 2011	0.97<br>(0.97 to 0.98)
Eastern Sub-Saharan Africa	2012 to 2016	1<br>(1 to 1)
Eastern Sub-Saharan Africa	2017 to 2021	1.06<br>(1.06 to 1.07)
High-income Asia Pacific	1992 to 1996	1.02<br>(1.01 to 1.04)
High-income Asia Pacific	1997 to 2001	1.02<br>(1.01 to 1.04)
High-income Asia Pacific	2002 to 2006	1.02<br>(1.01 to 1.03)
High-income Asia Pacific	2007 to 2011	1<br>(0.99 to 1.02)
High-income Asia Pacific	2012 to 2016	1<br>(1 to 1)
High-income Asia Pacific	2017 to 2021	0.94<br>(0.93 to 0.95)

High-income North America	1992 to 1996	0.88<br>(0.86 to 0.89)
High-income North America	1997 to 2001	0.93<br>(0.91 to 0.94)
High-income North America	2002 to 2006	1<br>(0.99 to 1.02)
High-income North America	2007 to 2011	1.01<br>(0.99 to 1.02)
High-income North America	2012 to 2016	1<br>(1 to 1)
High-income North America	2017 to 2021	0.99<br>(0.98 to 1)
North Africa and Middle East	1992 to 1996	0.85<br>(0.85 to 0.86)
North Africa and Middle East	1997 to 2001	0.88<br>(0.87 to 0.88)
North Africa and Middle East	2002 to 2006	0.91<br>(0.91 to 0.92)
North Africa and Middle East	2007 to 2011	0.95<br>(0.95 to 0.95)
North Africa and Middle East	2012 to 2016	1<br>(1 to 1)
North Africa and Middle East	2017 to 2021	1.05<br>(1.05 to 1.05)
Oceania	1992 to 1996	1.05<br>(1.03 to 1.07)
Oceania	1997 to 2001	1.03<br>(1.02 to 1.05)
Oceania	2002 to 2006	1.02<br>(1.01 to 1.03)
Oceania	2007 to 2011	1.01<br>(1 to 1.02)
Oceania	2012 to 2016	1<br>(1 to 1)
Oceania	2017 to 2021	0.99<br>(0.98 to 1)
South Asia	1992 to 1996	0.94<br>(0.94 to 0.95)
South Asia	1997 to 2001	0.96<br>(0.95 to 0.96)
South Asia	2002 to 2006	0.97<br>(0.97 to 0.97)
South Asia	2007 to 2011	0.99<br>(0.98 to 0.99)
South Asia	2012 to 2016	1<br>(1 to 1)
South Asia	2017 to 2021	1.01<br>(1 to 1.01)
Southeast Asia	1992 to 1996	0.99<br>(0.98 to 0.99)
Southeast Asia	1997 to 2001	0.99<br>(0.99 to 1)
Southeast Asia	2002 to 2006	0.99<br>(0.99 to 0.99)
Southeast Asia	2007 to 2011	0.99<br>(0.99 to 1)
Southeast Asia	2012 to 2016	1<br>(1 to 1)
Southeast Asia	2017 to 2021	1.03<br>(1.02 to 1.03)
Southern Latin America	1992 to 1996	0.98<br>(0.97 to 0.99)
Southern Latin America	1997 to 2001	0.99<br>(0.98 to 1)
Southern Latin America	2002 to 2006	1.02<br>(1.01 to 1.03)

Southern Latin America	2007 to 2011	1.02 (1.01 to 1.03)
Southern Latin America	2012 to 2016	1 (1 to 1)
Southern Latin America	2017 to 2021	0.98 (0.98 to 0.99)
Southern Sub-Saharan Africa	1992 to 1996	0.98 (0.97 to 1)
Southern Sub-Saharan Africa	1997 to 2001	0.99 (0.97 to 1)
Southern Sub-Saharan Africa	2002 to 2006	1 (0.98 to 1.01)
Southern Sub-Saharan Africa	2007 to 2011	0.99 (0.98 to 1)
Southern Sub-Saharan Africa	2012 to 2016	1 (1 to 1)
Southern Sub-Saharan Africa	2017 to 2021	1.01 (1 to 1.03)
Tropical Latin America	1992 to 1996	1.02 (1.01 to 1.03)
Tropical Latin America	1997 to 2001	1.03 (1.02 to 1.04)
Tropical Latin America	2002 to 2006	1.03 (1.02 to 1.03)
Tropical Latin America	2007 to 2011	1.01 (1 to 1.01)
Tropical Latin America	2012 to 2016	1 (1 to 1)
Tropical Latin America	2017 to 2021	1.02 (1.01 to 1.02)
Western Europe	1992 to 1996	1.01 (1 to 1.01)
Western Europe	1997 to 2001	1.01 (1 to 1.01)
Western Europe	2002 to 2006	1.01 (1 to 1.02)
Western Europe	2007 to 2011	1.01 (1 to 1.01)
Western Europe	2012 to 2016	1 (1 to 1)
Western Europe	2017 to 2021	1.02 (1.01 to 1.02)
Western Sub-Saharan Africa	1992 to 1996	0.89 (0.88 to 0.89)
Western Sub-Saharan Africa	1997 to 2001	0.89 (0.88 to 0.89)
Western Sub-Saharan Africa	2002 to 2006	0.92 (0.92 to 0.93)
Western Sub-Saharan Africa	2007 to 2011	0.96 (0.96 to 0.97)
Western Sub-Saharan Africa	2012 to 2016	1 (1 to 1)
Western Sub-Saharan Africa	2017 to 2021	1.06 (1.06 to 1.07)

COPD<br>Location	Cohort	Prevalence rate ratio
Global	1897 to 1901	0.89 (0.86 to 0.93)
Global	1902 to 1906	0.94 (0.92 to 0.96)
Global	1907 to 1911	0.97 (0.96 to 0.98)
Global	1912 to 1916	1 (0.99 to 1.01)

Global	1917 to 1921	1.03<br>(1.02 to 1.03)
Global	1922 to 1926	1.04<br>(1.03 to 1.05)
Global	1927 to 1931	1.05<br>(1.04 to 1.05)
Global	1932 to 1936	1.05<br>(1.04 to 1.06)
Global	1937 to 1941	1.05<br>(1.05 to 1.06)
Global	1942 to 1946	1.05<br>(1.05 to 1.06)
Global	1947 to 1951	1.04<br>(1.04 to 1.05)
Global	1952 to 1956	1.02<br>(1.02 to 1.03)
Global	1957 to 1961	1<br>(1 to 1)
Global	1962 to 1966	0.97<br>(0.97 to 0.98)
High SDI	1897 to 1901	0.91<br>(0.87 to 0.96)
High SDI	1902 to 1906	0.95<br>(0.92 to 0.97)
High SDI	1907 to 1911	0.97<br>(0.95 to 0.98)
High SDI	1912 to 1916	0.98<br>(0.96 to 0.99)
High SDI	1917 to 1921	1<br>(0.99 to 1.02)
High SDI	1922 to 1926	1<br>(0.99 to 1.02)
High SDI	1927 to 1931	1<br>(0.99 to 1.01)
High SDI	1932 to 1936	0.99<br>(0.98 to 1)
High SDI	1937 to 1941	0.99<br>(0.98 to 1)
High SDI	1942 to 1946	1<br>(0.99 to 1.01)
High SDI	1947 to 1951	1.01<br>(1 to 1.02)
High SDI	1952 to 1956	1<br>(0.99 to 1.01)
High SDI	1957 to 1961	1<br>(1 to 1)
High SDI	1962 to 1966	0.98<br>(0.97 to 1)
High-middle SDI	1897 to 1901	0.95<br>(0.85 to 1.07)
High-middle SDI	1902 to 1906	1<br>(0.95 to 1.05)
High-middle SDI	1907 to 1911	1.04<br>(1.01 to 1.07)
High-middle SDI	1912 to 1916	1.08<br>(1.06 to 1.1)
High-middle SDI	1917 to 1921	1.11<br>(1.09 to 1.13)
High-middle SDI	1922 to 1926	1.13<br>(1.11 to 1.15)
High-middle SDI	1927 to 1931	1.13<br>(1.11 to 1.15)
High-middle SDI	1932 to 1936	1.14<br>(1.12 to 1.16)
High-middle SDI	1937 to 1941	1.12<br>(1.1 to 1.14)

High-middle SDI	1942 to 1946	1.11 (1.09 to 1.12)
High-middle SDI	1947 to 1951	1.08 (1.06 to 1.1)
High-middle SDI	1952 to 1956	1.04 (1.03 to 1.06)
High-middle SDI	1957 to 1961	1 (1 to 1)
High-middle SDI	1962 to 1966	0.95 (0.93 to 0.97)
Middle SDI	1897 to 1901	0.78 (0.69 to 0.88)
Middle SDI	1902 to 1906	0.85 (0.81 to 0.9)
Middle SDI	1907 to 1911	0.92 (0.89 to 0.94)
Middle SDI	1912 to 1916	0.97 (0.95 to 0.99)
Middle SDI	1917 to 1921	1.02 (1 to 1.03)
Middle SDI	1922 to 1926	1.05 (1.03 to 1.06)
Middle SDI	1927 to 1931	1.07 (1.05 to 1.08)
Middle SDI	1932 to 1936	1.08 (1.07 to 1.09)
Middle SDI	1937 to 1941	1.08 (1.07 to 1.1)
Middle SDI	1942 to 1946	1.08 (1.07 to 1.09)
Middle SDI	1947 to 1951	1.06 (1.05 to 1.07)
Middle SDI	1952 to 1956	1.04 (1.02 to 1.05)
Middle SDI	1957 to 1961	1 (1 to 1)
Middle SDI	1962 to 1966	0.97 (0.95 to 0.98)
Low-middle SDI	1897 to 1901	0.81 (0.79 to 0.83)
Low-middle SDI	1902 to 1906	0.84 (0.83 to 0.85)
Low-middle SDI	1907 to 1911	0.88 (0.88 to 0.89)
Low-middle SDI	1912 to 1916	0.91 (0.91 to 0.92)
Low-middle SDI	1917 to 1921	0.94 (0.94 to 0.94)
Low-middle SDI	1922 to 1926	0.97 (0.96 to 0.97)
Low-middle SDI	1927 to 1931	0.99 (0.99 to 0.99)
Low-middle SDI	1932 to 1936	1.01 (1.01 to 1.02)
Low-middle SDI	1937 to 1941	1.02 (1.02 to 1.03)
Low-middle SDI	1942 to 1946	1.03 (1.02 to 1.03)
Low-middle SDI	1947 to 1951	1.02 (1.02 to 1.02)
Low-middle SDI	1952 to 1956	1.01 (1.01 to 1.01)
Low-middle SDI	1957 to 1961	1 (1 to 1)
Low-middle SDI	1962 to 1966	0.99 (0.98 to 0.99)

Low SDI	1897 to 1901	0.73<br>(0.6 to 0.89)
Low SDI	1902 to 1906	0.79<br>(0.73 to 0.86)
Low SDI	1907 to 1911	0.86<br>(0.82 to 0.89)
Low SDI	1912 to 1916	0.88<br>(0.85 to 0.9)
Low SDI	1917 to 1921	0.9<br>(0.88 to 0.92)
Low SDI	1922 to 1926	0.92<br>(0.9 to 0.94)
Low SDI	1927 to 1931	0.94<br>(0.93 to 0.96)
Low SDI	1932 to 1936	0.97<br>(0.96 to 0.99)
Low SDI	1937 to 1941	1<br>(0.98 to 1.01)
Low SDI	1942 to 1946	1.01<br>(1 to 1.02)
Low SDI	1947 to 1951	1.02<br>(1.01 to 1.04)
Low SDI	1952 to 1956	1.02<br>(1 to 1.03)
Low SDI	1957 to 1961	1<br>(1 to 1)
Low SDI	1962 to 1966	0.99<br>(0.97 to 1.01)
Andean Latin America	1897 to 1901	0.65<br>(0.63 to 0.68)
Andean Latin America	1902 to 1906	0.72<br>(0.71 to 0.73)
Andean Latin America	1907 to 1911	0.78<br>(0.77 to 0.79)
Andean Latin America	1912 to 1916	0.84<br>(0.84 to 0.85)
Andean Latin America	1917 to 1921	0.9<br>(0.89 to 0.9)
Andean Latin America	1922 to 1926	0.94<br>(0.93 to 0.95)
Andean Latin America	1927 to 1931	0.97<br>(0.96 to 0.98)
Andean Latin America	1932 to 1936	0.99<br>(0.99 to 1)
Andean Latin America	1937 to 1941	1.01<br>(1 to 1.02)
Andean Latin America	1942 to 1946	1.02<br>(1.01 to 1.02)
Andean Latin America	1947 to 1951	1.02<br>(1.01 to 1.03)
Andean Latin America	1952 to 1956	1.01<br>(1.01 to 1.02)
Andean Latin America	1957 to 1961	1<br>(1 to 1)
Andean Latin America	1962 to 1966	0.99<br>(0.98 to 1)
Australasia	1897 to 1901	1.53<br>(1.41 to 1.65)
Australasia	1902 to 1906	1.52<br>(1.47 to 1.57)
Australasia	1907 to 1911	1.5<br>(1.47 to 1.54)
Australasia	1912 to 1916	1.49<br>(1.46 to 1.51)
Australasia	1917 to 1921	1.47<br>(1.45 to 1.49)

Australasia	1922 to 1926	1.44<br>(1.42 to 1.46)
Australasia	1927 to 1931	1.4<br>(1.38 to 1.42)
Australasia	1932 to 1936	1.34<br>(1.32 to 1.36)
Australasia	1937 to 1941	1.27<br>(1.25 to 1.29)
Australasia	1942 to 1946	1.2<br>(1.18 to 1.22)
Australasia	1947 to 1951	1.13<br>(1.12 to 1.15)
Australasia	1952 to 1956	1.07<br>(1.05 to 1.08)
Australasia	1957 to 1961	1<br>(1 to 1)
Australasia	1962 to 1966	0.93<br>(0.91 to 0.95)
Caribbean	1897 to 1901	0.63<br>(0.61 to 0.66)
Caribbean	1902 to 1906	0.68<br>(0.67 to 0.7)
Caribbean	1907 to 1911	0.72<br>(0.72 to 0.73)
Caribbean	1912 to 1916	0.76<br>(0.75 to 0.76)
Caribbean	1917 to 1921	0.8<br>(0.79 to 0.8)
Caribbean	1922 to 1926	0.84<br>(0.83 to 0.84)
Caribbean	1927 to 1931	0.87<br>(0.87 to 0.88)
Caribbean	1932 to 1936	0.9<br>(0.9 to 0.91)
Caribbean	1937 to 1941	0.93<br>(0.93 to 0.94)
Caribbean	1942 to 1946	0.96<br>(0.95 to 0.97)
Caribbean	1947 to 1951	0.98<br>(0.97 to 0.98)
Caribbean	1952 to 1956	0.99<br>(0.98 to 0.99)
Caribbean	1957 to 1961	1<br>(1 to 1)
Caribbean	1962 to 1966	1.03<br>(1.02 to 1.04)
Central Asia	1897 to 1901	0.8<br>(0.74 to 0.86)
Central Asia	1902 to 1906	0.83<br>(0.81 to 0.86)
Central Asia	1907 to 1911	0.87<br>(0.85 to 0.89)
Central Asia	1912 to 1916	0.91<br>(0.89 to 0.92)
Central Asia	1917 to 1921	0.95<br>(0.93 to 0.96)
Central Asia	1922 to 1926	0.98<br>(0.97 to 1)
Central Asia	1927 to 1931	1.02<br>(1.01 to 1.03)
Central Asia	1932 to 1936	1.03<br>(1.02 to 1.04)
Central Asia	1937 to 1941	1.05<br>(1.04 to 1.06)
Central Asia	1942 to 1946	1.04<br>(1.03 to 1.05)

Central Asia	1947 to 1951	1.03<br>(1.02 to 1.04)
Central Asia	1952 to 1956	1<br>(1 to 1)
Central Asia	1957 to 1961	0.96<br>(0.95 to 0.97)
Central Asia	1962 to 1966	0.91<br>(0.9 to 0.93)
Central Europe	1897 to 1901	0.75<br>(0.7 to 0.79)
Central Europe	1902 to 1906	0.81<br>(0.79 to 0.83)
Central Europe	1907 to 1911	0.87<br>(0.86 to 0.88)
Central Europe	1912 to 1916	0.92<br>(0.91 to 0.93)
Central Europe	1917 to 1921	0.96<br>(0.95 to 0.97)
Central Europe	1922 to 1926	0.99<br>(0.98 to 1)
Central Europe	1927 to 1931	1.02<br>(1.01 to 1.03)
Central Europe	1932 to 1936	1.04<br>(1.03 to 1.05)
Central Europe	1937 to 1941	1.05<br>(1.04 to 1.05)
Central Europe	1942 to 1946	1.05<br>(1.04 to 1.06)
Central Europe	1947 to 1951	1.04<br>(1.03 to 1.05)
Central Europe	1952 to 1956	1.02<br>(1.01 to 1.03)
Central Europe	1957 to 1961	1<br>(1 to 1)
Central Europe	1962 to 1966	0.98<br>(0.96 to 0.99)
Central Latin America	1897 to 1901	0.78<br>(0.73 to 0.83)
Central Latin America	1902 to 1906	0.82<br>(0.79 to 0.84)
Central Latin America	1907 to 1911	0.86<br>(0.84 to 0.88)
Central Latin America	1912 to 1916	0.9<br>(0.89 to 0.92)
Central Latin America	1917 to 1921	0.94<br>(0.92 to 0.95)
Central Latin America	1922 to 1926	0.97<br>(0.96 to 0.98)
Central Latin America	1927 to 1931	0.99<br>(0.98 to 1.01)
Central Latin America	1932 to 1936	1.02<br>(1 to 1.03)
Central Latin America	1937 to 1941	1.03<br>(1.02 to 1.04)
Central Latin America	1942 to 1946	1.03<br>(1.02 to 1.04)
Central Latin America	1947 to 1951	1.03<br>(1.02 to 1.04)
Central Latin America	1952 to 1956	1.02<br>(1.01 to 1.03)
Central Latin America	1957 to 1961	1<br>(1 to 1)
Central Latin America	1962 to 1966	0.98<br>(0.97 to 0.99)
Central Sub-Saharan Africa	1897 to 1901	0.68<br>(0.61 to 0.76)

Central Sub-Saharan Africa	1902 to 1906	0.72<br>(0.69 to 0.76)
Central Sub-Saharan Africa	1907 to 1911	0.77<br>(0.75 to 0.79)
Central Sub-Saharan Africa	1912 to 1916	0.83<br>(0.81 to 0.84)
Central Sub-Saharan Africa	1917 to 1921	0.87<br>(0.87 to 0.88)
Central Sub-Saharan Africa	1922 to 1926	0.91<br>(0.91 to 0.92)
Central Sub-Saharan Africa	1927 to 1931	0.94<br>(0.94 to 0.95)
Central Sub-Saharan Africa	1932 to 1936	0.97<br>(0.96 to 0.97)
Central Sub-Saharan Africa	1937 to 1941	0.98<br>(0.98 to 0.99)
Central Sub-Saharan Africa	1942 to 1946	0.99<br>(0.99 to 1)
Central Sub-Saharan Africa	1947 to 1951	1<br>(1 to 1.01)
Central Sub-Saharan Africa	1952 to 1956	1<br>(1 to 1.01)
Central Sub-Saharan Africa	1957 to 1961	1<br>(1 to 1)
Central Sub-Saharan Africa	1962 to 1966	0.99<br>(0.99 to 1)
East Asia	1897 to 1901	0.87<br>(0.58 to 1.32)
East Asia	1902 to 1906	0.95<br>(0.83 to 1.09)
East Asia	1907 to 1911	1.05<br>(0.98 to 1.12)
East Asia	1912 to 1916	1.12<br>(1.08 to 1.17)
East Asia	1917 to 1921	1.18<br>(1.14 to 1.21)
East Asia	1922 to 1926	1.21<br>(1.18 to 1.24)
East Asia	1927 to 1931	1.22<br>(1.19 to 1.26)
East Asia	1932 to 1936	1.22<br>(1.19 to 1.25)
East Asia	1937 to 1941	1.2<br>(1.17 to 1.23)
East Asia	1942 to 1946	1.17<br>(1.14 to 1.2)
East Asia	1947 to 1951	1.12<br>(1.09 to 1.14)
East Asia	1952 to 1956	1.06<br>(1.04 to 1.08)
East Asia	1957 to 1961	1<br>(1 to 1)
East Asia	1962 to 1966	0.94<br>(0.91 to 0.97)
Eastern Europe	1897 to 1901	1.32<br>(1.04 to 1.68)
Eastern Europe	1902 to 1906	1.32<br>(1.2 to 1.46)
Eastern Europe	1907 to 1911	1.32<br>(1.25 to 1.4)
Eastern Europe	1912 to 1916	1.29<br>(1.24 to 1.35)
Eastern Europe	1917 to 1921	1.25<br>(1.2 to 1.3)
Eastern Europe	1922 to 1926	1.21<br>(1.17 to 1.26)

Eastern Europe	1927 to 1931	1.18<br>(1.14 to 1.22)
Eastern Europe	1932 to 1936	1.14<br>(1.11 to 1.18)
Eastern Europe	1937 to 1941	1.11<br>(1.07 to 1.14)
Eastern Europe	1942 to 1946	1.08<br>(1.04 to 1.11)
Eastern Europe	1947 to 1951	1.05<br>(1.02 to 1.09)
Eastern Europe	1952 to 1956	1.03<br>(1 to 1.06)
Eastern Europe	1957 to 1961	1<br>(1 to 1)
Eastern Europe	1962 to 1966	0.96<br>(0.91 to 1)
Eastern Sub-Saharan Africa	1897 to 1901	0.81<br>(0.75 to 0.88)
Eastern Sub-Saharan Africa	1902 to 1906	0.86<br>(0.84 to 0.89)
Eastern Sub-Saharan Africa	1907 to 1911	0.9<br>(0.88 to 0.91)
Eastern Sub-Saharan Africa	1912 to 1916	0.93<br>(0.92 to 0.94)
Eastern Sub-Saharan Africa	1917 to 1921	0.95<br>(0.94 to 0.96)
Eastern Sub-Saharan Africa	1922 to 1926	0.97<br>(0.96 to 0.97)
Eastern Sub-Saharan Africa	1927 to 1931	0.98<br>(0.98 to 0.99)
Eastern Sub-Saharan Africa	1932 to 1936	1<br>(1 to 1)
Eastern Sub-Saharan Africa	1937 to 1941	1.01<br>(1 to 1.01)
Eastern Sub-Saharan Africa	1942 to 1946	1.01<br>(1.01 to 1.02)
Eastern Sub-Saharan Africa	1947 to 1951	1.02<br>(1.01 to 1.02)
Eastern Sub-Saharan Africa	1952 to 1956	1.01<br>(1.01 to 1.02)
Eastern Sub-Saharan Africa	1957 to 1961	1<br>(1 to 1)
Eastern Sub-Saharan Africa	1962 to 1966	0.98<br>(0.97 to 0.99)
High-income Asia Pacific	1897 to 1901	1.11<br>(1 to 1.24)
High-income Asia Pacific	1902 to 1906	1.12<br>(1.06 to 1.18)
High-income Asia Pacific	1907 to 1911	1.12<br>(1.08 to 1.17)
High-income Asia Pacific	1912 to 1916	1.12<br>(1.08 to 1.15)
High-income Asia Pacific	1917 to 1921	1.1<br>(1.07 to 1.13)
High-income Asia Pacific	1922 to 1926	1.08<br>(1.05 to 1.11)
High-income Asia Pacific	1927 to 1931	1.07<br>(1.04 to 1.1)
High-income Asia Pacific	1932 to 1936	1.05<br>(1.02 to 1.08)
High-income Asia Pacific	1937 to 1941	1.04<br>(1.01 to 1.06)
High-income Asia Pacific	1942 to 1946	1.01<br>(0.98 to 1.03)
High-income Asia Pacific	1947 to 1951	0.97<br>(0.94 to 1)

High-income Asia Pacific	1952 to 1956	0.97<br>(0.94 to 0.99)
High-income Asia Pacific	1957 to 1961	1<br>(1 to 1)
High-income Asia Pacific	1962 to 1966	0.98<br>(0.94 to 1.02)
High-income North America	1897 to 1901	0.75<br>(0.65 to 0.86)
High-income North America	1902 to 1906	0.78<br>(0.72 to 0.83)
High-income North America	1907 to 1911	0.8<br>(0.76 to 0.84)
High-income North America	1912 to 1916	0.83<br>(0.8 to 0.86)
High-income North America	1917 to 1921	0.86<br>(0.83 to 0.89)
High-income North America	1922 to 1926	0.89<br>(0.86 to 0.92)
High-income North America	1927 to 1931	0.91<br>(0.89 to 0.94)
High-income North America	1932 to 1936	0.94<br>(0.91 to 0.97)
High-income North America	1937 to 1941	0.96<br>(0.93 to 0.99)
High-income North America	1942 to 1946	0.98<br>(0.95 to 1)
High-income North America	1947 to 1951	0.99<br>(0.96 to 1.01)
High-income North America	1952 to 1956	1<br>(0.97 to 1.02)
High-income North America	1957 to 1961	1<br>(1 to 1)
High-income North America	1962 to 1966	1<br>(0.96 to 1.05)
North Africa and Middle East	1897 to 1901	0.57<br>(0.54 to 0.59)
North Africa and Middle East	1902 to 1906	0.63<br>(0.62 to 0.65)
North Africa and Middle East	1907 to 1911	0.69<br>(0.69 to 0.7)
North Africa and Middle East	1912 to 1916	0.75<br>(0.74 to 0.75)
North Africa and Middle East	1917 to 1921	0.78<br>(0.78 to 0.79)
North Africa and Middle East	1922 to 1926	0.83<br>(0.82 to 0.83)
North Africa and Middle East	1927 to 1931	0.88<br>(0.88 to 0.89)
North Africa and Middle East	1932 to 1936	0.93<br>(0.92 to 0.93)
North Africa and Middle East	1937 to 1941	0.95<br>(0.95 to 0.96)
North Africa and Middle East	1942 to 1946	0.98<br>(0.98 to 0.99)
North Africa and Middle East	1947 to 1951	1<br>(1 to 1.01)
North Africa and Middle East	1952 to 1956	1.01<br>(1 to 1.01)
North Africa and Middle East	1957 to 1961	1<br>(1 to 1)
North Africa and Middle East	1962 to 1966	1<br>(0.99 to 1)
Oceania	1897 to 1901	1.11<br>(0.88 to 1.4)
Oceania	1902 to 1906	1.12<br>(1.02 to 1.24)

Oceania	1907 to 1911	1.11<br>(1.06 to 1.17)
Oceania	1912 to 1916	1.11<br>(1.08 to 1.14)
Oceania	1917 to 1921	1.11<br>(1.09 to 1.14)
Oceania	1922 to 1926	1.11<br>(1.09 to 1.13)
Oceania	1927 to 1931	1.09<br>(1.08 to 1.11)
Oceania	1932 to 1936	1.07<br>(1.05 to 1.08)
Oceania	1937 to 1941	1.05<br>(1.03 to 1.07)
Oceania	1942 to 1946	1.04<br>(1.02 to 1.05)
Oceania	1947 to 1951	1.03<br>(1.01 to 1.04)
Oceania	1952 to 1956	1.01<br>(0.99 to 1.02)
Oceania	1957 to 1961	1<br>(1 to 1)
Oceania	1962 to 1966	0.99<br>(0.97 to 1.01)
South Asia	1897 to 1901	0.84<br>(0.81 to 0.87)
South Asia	1902 to 1906	0.86<br>(0.85 to 0.88)
South Asia	1907 to 1911	0.89<br>(0.88 to 0.9)
South Asia	1912 to 1916	0.92<br>(0.91 to 0.93)
South Asia	1917 to 1921	0.95<br>(0.95 to 0.96)
South Asia	1922 to 1926	0.97<br>(0.97 to 0.98)
South Asia	1927 to 1931	0.99<br>(0.99 to 0.99)
South Asia	1932 to 1936	1.01<br>(1 to 1.01)
South Asia	1937 to 1941	1.01<br>(1.01 to 1.02)
South Asia	1942 to 1946	1.02<br>(1.01 to 1.02)
South Asia	1947 to 1951	1.01<br>(1.01 to 1.02)
South Asia	1952 to 1956	1.01<br>(1.01 to 1.01)
South Asia	1957 to 1961	1<br>(1 to 1)
South Asia	1962 to 1966	0.99<br>(0.98 to 0.99)
Southeast Asia	1897 to 1901	0.86<br>(0.82 to 0.9)
Southeast Asia	1902 to 1906	0.92<br>(0.9 to 0.94)
Southeast Asia	1907 to 1911	0.96<br>(0.95 to 0.98)
Southeast Asia	1912 to 1916	0.99<br>(0.98 to 1)
Southeast Asia	1917 to 1921	1.02<br>(1.01 to 1.03)
Southeast Asia	1922 to 1926	1.04<br>(1.03 to 1.04)
Southeast Asia	1927 to 1931	1.05<br>(1.04 to 1.05)

Southeast Asia	1932 to 1936	1.05<br>(1.04 to 1.05)
Southeast Asia	1937 to 1941	1.04<br>(1.04 to 1.05)
Southeast Asia	1942 to 1946	1.04<br>(1.03 to 1.04)
Southeast Asia	1947 to 1951	1.03<br>(1.03 to 1.04)
Southeast Asia	1952 to 1956	1.02<br>(1.01 to 1.03)
Southeast Asia	1957 to 1961	1<br>(1 to 1)
Southeast Asia	1962 to 1966	0.98<br>(0.97 to 0.98)
Southern Latin America	1897 to 1901	0.94<br>(0.88 to 1.01)
Southern Latin America	1902 to 1906	0.99<br>(0.96 to 1.02)
Southern Latin America	1907 to 1911	1.01<br>(0.99 to 1.03)
Southern Latin America	1912 to 1916	1.03<br>(1.01 to 1.04)
Southern Latin America	1917 to 1921	1.05<br>(1.03 to 1.07)
Southern Latin America	1922 to 1926	1.07<br>(1.05 to 1.08)
Southern Latin America	1927 to 1931	1.07<br>(1.06 to 1.09)
Southern Latin America	1932 to 1936	1.07<br>(1.06 to 1.09)
Southern Latin America	1937 to 1941	1.07<br>(1.05 to 1.08)
Southern Latin America	1942 to 1946	1.05<br>(1.04 to 1.06)
Southern Latin America	1947 to 1951	1.04<br>(1.02 to 1.05)
Southern Latin America	1952 to 1956	1.02<br>(1.01 to 1.03)
Southern Latin America	1957 to 1961	1<br>(1 to 1)
Southern Latin America	1962 to 1966	0.98<br>(0.96 to 1)
Southern Sub-Saharan Africa	1897 to 1901	0.96<br>(0.84 to 1.1)
Southern Sub-Saharan Africa	1902 to 1906	0.97<br>(0.91 to 1.03)
Southern Sub-Saharan Africa	1907 to 1911	0.98<br>(0.95 to 1.02)
Southern Sub-Saharan Africa	1912 to 1916	0.98<br>(0.95 to 1.01)
Southern Sub-Saharan Africa	1917 to 1921	0.98<br>(0.96 to 1.01)
Southern Sub-Saharan Africa	1922 to 1926	1<br>(0.98 to 1.02)
Southern Sub-Saharan Africa	1927 to 1931	1.01<br>(0.99 to 1.03)
Southern Sub-Saharan Africa	1932 to 1936	1.02<br>(1 to 1.04)
Southern Sub-Saharan Africa	1937 to 1941	1.03<br>(1.02 to 1.05)
Southern Sub-Saharan Africa	1942 to 1946	1.04<br>(1.02 to 1.06)
Southern Sub-Saharan Africa	1947 to 1951	1.04<br>(1.02 to 1.05)
Southern Sub-Saharan Africa	1952 to 1956	1.02<br>(1.01 to 1.04)

Southern Sub-Saharan Africa	1957 to 1961	1<br>(1 to 1)
Southern Sub-Saharan Africa	1962 to 1966	0.96<br>(0.94 to 0.98)
Tropical Latin America	1897 to 1901	1.03<br>(0.97 to 1.11)
Tropical Latin America	1902 to 1906	1.05<br>(1.02 to 1.08)
Tropical Latin America	1907 to 1911	1.06<br>(1.04 to 1.08)
Tropical Latin America	1912 to 1916	1.06<br>(1.05 to 1.08)
Tropical Latin America	1917 to 1921	1.07<br>(1.06 to 1.08)
Tropical Latin America	1922 to 1926	1.07<br>(1.06 to 1.08)
Tropical Latin America	1927 to 1931	1.07<br>(1.06 to 1.08)
Tropical Latin America	1932 to 1936	1.07<br>(1.06 to 1.08)
Tropical Latin America	1937 to 1941	1.06<br>(1.05 to 1.07)
Tropical Latin America	1942 to 1946	1.05<br>(1.04 to 1.06)
Tropical Latin America	1947 to 1951	1.04<br>(1.03 to 1.05)
Tropical Latin America	1952 to 1956	1.03<br>(1.02 to 1.04)
Tropical Latin America	1957 to 1961	1<br>(1 to 1)
Tropical Latin America	1962 to 1966	0.97<br>(0.95 to 0.98)
Western Europe	1897 to 1901	0.96<br>(0.92 to 1.01)
Western Europe	1902 to 1906	0.99<br>(0.96 to 1.01)
Western Europe	1907 to 1911	1.01<br>(0.99 to 1.02)
Western Europe	1912 to 1916	1.03<br>(1.01 to 1.04)
Western Europe	1917 to 1921	1.06<br>(1.05 to 1.07)
Western Europe	1922 to 1926	1.06<br>(1.05 to 1.07)
Western Europe	1927 to 1931	1.06<br>(1.05 to 1.08)
Western Europe	1932 to 1936	1.06<br>(1.05 to 1.07)
Western Europe	1937 to 1941	1.06<br>(1.05 to 1.07)
Western Europe	1942 to 1946	1.05<br>(1.04 to 1.07)
Western Europe	1947 to 1951	1.04<br>(1.03 to 1.05)
Western Europe	1952 to 1956	1.02<br>(1.01 to 1.03)
Western Europe	1957 to 1961	1<br>(1 to 1)
Western Europe	1962 to 1966	0.98<br>(0.96 to 1)
Western Sub-Saharan Africa	1897 to 1901	0.61<br>(0.57 to 0.66)
Western Sub-Saharan Africa	1902 to 1906	0.66<br>(0.64 to 0.68)
Western Sub-Saharan Africa	1907 to 1911	0.71<br>(0.7 to 0.72)

Western Sub-Saharan Africa	1912 to 1916	0.76 (0.75 to 0.76)
Western Sub-Saharan Africa	1917 to 1921	0.8 (0.79 to 0.81)
Western Sub-Saharan Africa	1922 to 1926	0.84 (0.83 to 0.85)
Western Sub-Saharan Africa	1927 to 1931	0.89 (0.88 to 0.89)
Western Sub-Saharan Africa	1932 to 1936	0.92 (0.91 to 0.92)
Western Sub-Saharan Africa	1937 to 1941	0.94 (0.93 to 0.94)
Western Sub-Saharan Africa	1942 to 1946	0.96 (0.95 to 0.96)
Western Sub-Saharan Africa	1947 to 1951	0.98 (0.97 to 0.98)
Western Sub-Saharan Africa	1952 to 1956	0.99 (0.98 to 1)
Western Sub-Saharan Africa	1957 to 1961	1 (1 to 1)
Western Sub-Saharan Africa	1962 to 1966	1 (1 to 1.01)

Table S9 APC Model Analysis Results of Asthma Prevalence Among Adults Aged 55 and Above in the Global, 5 SDI Regions, and 21 GBD Regions

Asthma<br>Location	Age	Local drift (%/year)
Global	55 to 59	-2.37<br>(-2.47 to -2.28)
Global	60 to 64	-2.45<br>(-2.53 to -2.38)
Global	65 to 69	-2.57<br>(-2.64 to -2.5)
Global	70 to 74	-2.6<br>(-2.68 to -2.52)
Global	75 to 79	-2.59<br>(-2.68 to -2.5)
Global	80 to 84	-2.45<br>(-2.56 to -2.33)
Global	85 to 89	-2.02<br>(-2.19 to -1.85)
Global	90 to 94	-1.41<br>(-1.72 to -1.1)
Global	95+	-0.67<br>(-1.34 to 0.01)
High SDI	55 to 59	-1.79<br>(-1.92 to -1.67)
High SDI	60 to 64	-1.92<br>(-2.01 to -1.82)
High SDI	65 to 69	-2.16<br>(-2.26 to -2.07)
High SDI	70 to 74	-2.4<br>(-2.5 to -2.31)
High SDI	75 to 79	-2.6<br>(-2.72 to -2.49)
High SDI	80 to 84	-2.51<br>(-2.65 to -2.37)
High SDI	85 to 89	-1.99<br>(-2.19 to -1.79)
High SDI	90 to 94	-1.19<br>(-1.52 to -0.86)
High SDI	95+	-0.21<br>(-0.9 to 0.49)
High-middle SDI	55 to 59	-4.16<br>(-4.31 to -4)
High-middle SDI	60 to 64	-4.23<br>(-4.34 to -4.11)
High-middle SDI	65 to 69	-4.11<br>(-4.22 to -4)
High-middle SDI	70 to 74	-3.79<br>(-3.91 to -3.67)
High-middle SDI	75 to 79	-3.44<br>(-3.58 to -3.29)
High-middle SDI	80 to 84	-3<br>(-3.18 to -2.82)
High-middle SDI	85 to 89	-2.39<br>(-2.66 to -2.11)
High-middle SDI	90 to 94	-1.71<br>(-2.23 to -1.19)
High-middle SDI	95+	-0.79<br>(-2.05 to 0.49)
Middle SDI	55 to 59	-2.22<br>(-2.32 to -2.12)
Middle SDI	60 to 64	-2.45<br>(-2.53 to -2.37)
Middle SDI	65 to 69	-2.66<br>(-2.74 to -2.58)
Middle SDI	70 to 74	-2.65<br>(-2.73 to -2.56)

Middle SDI	75 to 79	-2.46<br>(-2.57 to -2.36)
Middle SDI	80 to 84	-2.18<br>(-2.32 to -2.04)
Middle SDI	85 to 89	-1.82<br>(-2.05 to -1.59)
Middle SDI	90 to 94	-1.5<br>(-1.94 to -1.06)
Middle SDI	95+	-1.3<br>(-2.35 to -0.25)
Low-middle SDI	55 to 59	-1.15<br>(-1.23 to -1.07)
Low-middle SDI	60 to 64	-1.2<br>(-1.26 to -1.13)
Low-middle SDI	65 to 69	-1.15<br>(-1.22 to -1.09)
Low-middle SDI	70 to 74	-1.05<br>(-1.12 to -0.98)
Low-middle SDI	75 to 79	-0.89<br>(-0.99 to -0.8)
Low-middle SDI	80 to 84	-0.76<br>(-0.89 to -0.63)
Low-middle SDI	85 to 89	-0.67<br>(-0.87 to -0.47)
Low-middle SDI	90 to 94	-0.53<br>(-0.9 to -0.16)
Low-middle SDI	95+	-0.42<br>(-1.27 to 0.44)
Low SDI	55 to 59	-1.28<br>(-1.31 to -1.25)
Low SDI	60 to 64	-1.28<br>(-1.3 to -1.25)
Low SDI	65 to 69	-1.21<br>(-1.23 to -1.18)
Low SDI	70 to 74	-1.07<br>(-1.1 to -1.04)
Low SDI	75 to 79	-0.9<br>(-0.94 to -0.86)
Low SDI	80 to 84	-0.81<br>(-0.87 to -0.74)
Low SDI	85 to 89	-0.76<br>(-0.87 to -0.66)
Low SDI	90 to 94	-0.64<br>(-0.85 to -0.43)
Low SDI	95+	-0.42<br>(-0.93 to 0.09)
Andean Latin America	55 to 59	-1.58<br>(-1.67 to -1.49)
Andean Latin America	60 to 64	-1.81<br>(-1.89 to -1.74)
Andean Latin America	65 to 69	-1.95<br>(-2.02 to -1.87)
Andean Latin America	70 to 74	-1.99<br>(-2.08 to -1.91)
Andean Latin America	75 to 79	-1.94<br>(-2.03 to -1.84)
Andean Latin America	80 to 84	-1.75<br>(-1.88 to -1.63)
Andean Latin America	85 to 89	-1.4<br>(-1.57 to -1.23)
Andean Latin America	90 to 94	-0.91<br>(-1.18 to -0.64)
Andean Latin America	95+	-0.33<br>(-0.88 to 0.22)
Australasia	55 to 59	-2.97<br>(-3.21 to -2.73)

Australasia	60 to 64	-2.91<br>(-3.1 to -2.72)
Australasia	65 to 69	-2.83<br>(-3.02 to -2.65)
Australasia	70 to 74	-2.74<br>(-2.94 to -2.54)
Australasia	75 to 79	-2.56<br>(-2.79 to -2.32)
Australasia	80 to 84	-2.19<br>(-2.47 to -1.91)
Australasia	85 to 89	-1.59<br>(-1.99 to -1.19)
Australasia	90 to 94	-0.89<br>(-1.55 to -0.23)
Australasia	95+	-0.22<br>(-1.63 to 1.21)
Caribbean	55 to 59	-1.54<br>(-1.59 to -1.49)
Caribbean	60 to 64	-1.5<br>(-1.54 to -1.46)
Caribbean	65 to 69	-1.49<br>(-1.53 to -1.44)
Caribbean	70 to 74	-1.5<br>(-1.55 to -1.45)
Caribbean	75 to 79	-1.5<br>(-1.56 to -1.44)
Caribbean	80 to 84	-1.51<br>(-1.59 to -1.42)
Caribbean	85 to 89	-1.41<br>(-1.53 to -1.28)
Caribbean	90 to 94	-1.17<br>(-1.39 to -0.96)
Caribbean	95+	-0.96<br>(-1.46 to -0.46)
Central Asia	55 to 59	-1.97<br>(-2.02 to -1.92)
Central Asia	60 to 64	-1.92<br>(-1.96 to -1.89)
Central Asia	65 to 69	-1.81<br>(-1.85 to -1.77)
Central Asia	70 to 74	-1.71<br>(-1.76 to -1.66)
Central Asia	75 to 79	-1.46<br>(-1.53 to -1.4)
Central Asia	80 to 84	-1.31<br>(-1.39 to -1.22)
Central Asia	85 to 89	-1.18<br>(-1.3 to -1.05)
Central Asia	90 to 94	-1.23<br>(-1.45 to -1)
Central Asia	95+	-1.21<br>(-1.76 to -0.66)
Central Europe	55 to 59	-2.76<br>(-2.87 to -2.66)
Central Europe	60 to 64	-3.06<br>(-3.14 to -2.99)
Central Europe	65 to 69	-3.4<br>(-3.47 to -3.33)
Central Europe	70 to 74	-3.6<br>(-3.68 to -3.53)
Central Europe	75 to 79	-3.67<br>(-3.77 to -3.58)
Central Europe	80 to 84	-3.5<br>(-3.62 to -3.38)
Central Europe	85 to 89	-3.41<br>(-3.6 to -3.22)

Central Europe	90 to 94	-3.26<br>(-3.64 to -2.89)
Central Europe	95+	-3.07<br>(-4.05 to -2.09)
Central Latin America	55 to 59	-3.36<br>(-3.53 to -3.19)
Central Latin America	60 to 64	-3.66<br>(-3.8 to -3.52)
Central Latin America	65 to 69	-3.97<br>(-4.11 to -3.83)
Central Latin America	70 to 74	-4.21<br>(-4.37 to -4.05)
Central Latin America	75 to 79	-4.34<br>(-4.52 to -4.15)
Central Latin America	80 to 84	-4.34<br>(-4.57 to -4.1)
Central Latin America	85 to 89	-4.16<br>(-4.47 to -3.84)
Central Latin America	90 to 94	-3.78<br>(-4.29 to -3.26)
Central Latin America	95+	-3.24<br>(-4.31 to -2.16)
Central Sub-Saharan Africa	55 to 59	-1.23<br>(-1.27 to -1.2)
Central Sub-Saharan Africa	60 to 64	-1.14<br>(-1.17 to -1.11)
Central Sub-Saharan Africa	65 to 69	-1.03<br>(-1.06 to -1.01)
Central Sub-Saharan Africa	70 to 74	-0.9<br>(-0.93 to -0.86)
Central Sub-Saharan Africa	75 to 79	-0.75<br>(-0.8 to -0.7)
Central Sub-Saharan Africa	80 to 84	-0.6<br>(-0.68 to -0.53)
Central Sub-Saharan Africa	85 to 89	-0.44<br>(-0.57 to -0.3)
Central Sub-Saharan Africa	90 to 94	-0.24<br>(-0.52 to 0.03)
Central Sub-Saharan Africa	95+	-0.08<br>(-0.7 to 0.54)
East Asia	55 to 59	-3.03<br>(-3.21 to -2.85)
East Asia	60 to 64	-3.47<br>(-3.6 to -3.33)
East Asia	65 to 69	-3.69<br>(-3.81 to -3.57)
East Asia	70 to 74	-3.63<br>(-3.75 to -3.5)
East Asia	75 to 79	-3.33<br>(-3.47 to -3.18)
East Asia	80 to 84	-2.93<br>(-3.13 to -2.72)
East Asia	85 to 89	-2.43<br>(-2.77 to -2.08)
East Asia	90 to 94	-1.94<br>(-2.69 to -1.18)
East Asia	95+	-1.47<br>(-3.65 to 0.76)
Eastern Europe	55 to 59	-5.67<br>(-5.79 to -5.54)
Eastern Europe	60 to 64	-5.73<br>(-5.83 to -5.64)
Eastern Europe	65 to 69	-5.72<br>(-5.81 to -5.63)
Eastern Europe	70 to 74	-5.63<br>(-5.74 to -5.51)

Eastern Europe	75 to 79	-5.46 (-5.6 to -5.32)
Eastern Europe	80 to 84	-5.34 (-5.52 to -5.16)
Eastern Europe	85 to 89	-5.12 (-5.41 to -4.83)
Eastern Europe	90 to 94	-4.91 (-5.47 to -4.33)
Eastern Europe	95+	-4.61 (-6.02 to -3.18)
Eastern Sub-Saharan Africa	55 to 59	-1.35 (-1.38 to -1.33)
Eastern Sub-Saharan Africa	60 to 64	-1.27 (-1.29 to -1.25)
Eastern Sub-Saharan Africa	65 to 69	-1.2 (-1.22 to -1.17)
Eastern Sub-Saharan Africa	70 to 74	-1.13 (-1.16 to -1.1)
Eastern Sub-Saharan Africa	75 to 79	-1.03 (-1.07 to -1)
Eastern Sub-Saharan Africa	80 to 84	-0.94 (-1 to -0.89)
Eastern Sub-Saharan Africa	85 to 89	-0.85 (-0.94 to -0.75)
Eastern Sub-Saharan Africa	90 to 94	-0.74 (-0.93 to -0.56)
Eastern Sub-Saharan Africa	95+	-0.63 (-1.1 to -0.17)
High-income Asia Pacific	55 to 59	-5.46 (-6.13 to -4.78)
High-income Asia Pacific	60 to 64	-5.55 (-6.03 to -5.07)
High-income Asia Pacific	65 to 69	-5.88 (-6.28 to -5.49)
High-income Asia Pacific	70 to 74	-6.39 (-6.76 to -6.01)
High-income Asia Pacific	75 to 79	-6.67 (-7.06 to -6.28)
High-income Asia Pacific	80 to 84	-6.48 (-6.91 to -6.06)
High-income Asia Pacific	85 to 89	-5.69 (-6.24 to -5.14)
High-income Asia Pacific	90 to 94	-4.54 (-5.41 to -3.65)
High-income Asia Pacific	95+	-3.35 (-5.2 to -1.46)
High-income North America	55 to 59	1.17 (0.98 to 1.36)
High-income North America	60 to 64	1.43 (1.27 to 1.58)
High-income North America	65 to 69	1.63 (1.48 to 1.78)
High-income North America	70 to 74	1.7 (1.54 to 1.86)
High-income North America	75 to 79	1.62 (1.43 to 1.82)
High-income North America	80 to 84	1.52 (1.28 to 1.76)
High-income North America	85 to 89	1.59 (1.24 to 1.93)
High-income North America	90 to 94	1.79 (1.24 to 2.35)
High-income North America	95+	2.09 (1.01 to 3.18)
North Africa and Middle East	55 to 59	-2.34 (-2.45 to -2.24)

North Africa and Middle East	60 to 64	-2.2<br>(-2.29 to -2.12)
North Africa and Middle East	65 to 69	-1.93<br>(-2.01 to -1.85)
North Africa and Middle East	70 to 74	-1.57<br>(-1.67 to -1.48)
North Africa and Middle East	75 to 79	-1.14<br>(-1.26 to -1.02)
North Africa and Middle East	80 to 84	-0.68<br>(-0.85 to -0.5)
North Africa and Middle East	85 to 89	-0.22<br>(-0.48 to 0.05)
North Africa and Middle East	90 to 94	0.19<br>(-0.26 to 0.65)
North Africa and Middle East	95+	0.73<br>(-0.21 to 1.68)
Oceania	55 to 59	-1.82<br>(-1.9 to -1.75)
Oceania	60 to 64	-1.84<br>(-1.91 to -1.78)
Oceania	65 to 69	-1.91<br>(-1.97 to -1.85)
Oceania	70 to 74	-1.94<br>(-2.01 to -1.87)
Oceania	75 to 79	-1.95<br>(-2.05 to -1.86)
Oceania	80 to 84	-1.85<br>(-1.99 to -1.7)
Oceania	85 to 89	-1.64<br>(-1.89 to -1.39)
Oceania	90 to 94	-1.51<br>(-2.03 to -0.99)
Oceania	95+	-1.72<br>(-2.91 to -0.52)
South Asia	55 to 59	-1.09<br>(-1.19 to -0.98)
South Asia	60 to 64	-1.17<br>(-1.26 to -1.08)
South Asia	65 to 69	-1.14<br>(-1.23 to -1.06)
South Asia	70 to 74	-1.04<br>(-1.14 to -0.94)
South Asia	75 to 79	-0.89<br>(-1.02 to -0.76)
South Asia	80 to 84	-0.79<br>(-0.97 to -0.61)
South Asia	85 to 89	-0.72<br>(-1.01 to -0.44)
South Asia	90 to 94	-0.59<br>(-1.14 to -0.05)
South Asia	95+	-0.38<br>(-1.7 to 0.96)
Southeast Asia	55 to 59	-2.07<br>(-2.12 to -2.02)
Southeast Asia	60 to 64	-1.95<br>(-1.99 to -1.9)
Southeast Asia	65 to 69	-1.8<br>(-1.84 to -1.76)
Southeast Asia	70 to 74	-1.68<br>(-1.73 to -1.63)
Southeast Asia	75 to 79	-1.54<br>(-1.6 to -1.48)
Southeast Asia	80 to 84	-1.34<br>(-1.43 to -1.26)
Southeast Asia	85 to 89	-1.03<br>(-1.16 to -0.9)

Southeast Asia	90 to 94	-0.59 (-0.84 to -0.35)
Southeast Asia	95+	-0.16 (-0.71 to 0.39)
Southern Latin America	55 to 59	-1.98 (-2.03 to -1.93)
Southern Latin America	60 to 64	-1.99 (-2.02 to -1.95)
Southern Latin America	65 to 69	-1.89 (-1.93 to -1.86)
Southern Latin America	70 to 74	-1.67 (-1.71 to -1.64)
Southern Latin America	75 to 79	-1.37 (-1.41 to -1.32)
Southern Latin America	80 to 84	-1.02 (-1.07 to -0.96)
Southern Latin America	85 to 89	-0.63 (-0.71 to -0.55)
Southern Latin America	90 to 94	-0.25 (-0.39 to -0.11)
Southern Latin America	95+	0.15 (-0.17 to 0.46)
Southern Sub-Saharan Africa	55 to 59	-1.71 (-1.77 to -1.64)
Southern Sub-Saharan Africa	60 to 64	-1.6 (-1.65 to -1.54)
Southern Sub-Saharan Africa	65 to 69	-1.5 (-1.56 to -1.45)
Southern Sub-Saharan Africa	70 to 74	-1.49 (-1.55 to -1.42)
Southern Sub-Saharan Africa	75 to 79	-1.47 (-1.55 to -1.39)
Southern Sub-Saharan Africa	80 to 84	-1.4 (-1.52 to -1.29)
Southern Sub-Saharan Africa	85 to 89	-1.27 (-1.44 to -1.1)
Southern Sub-Saharan Africa	90 to 94	-1.14 (-1.42 to -0.86)
Southern Sub-Saharan Africa	95+	-1.15 (-1.76 to -0.53)
Tropical Latin America	55 to 59	-3.19 (-3.38 to -3)
Tropical Latin America	60 to 64	-3.28 (-3.44 to -3.12)
Tropical Latin America	65 to 69	-3.29 (-3.45 to -3.13)
Tropical Latin America	70 to 74	-3.12 (-3.3 to -2.94)
Tropical Latin America	75 to 79	-2.8 (-3.02 to -2.58)
Tropical Latin America	80 to 84	-2.35 (-2.63 to -2.06)
Tropical Latin America	85 to 89	-1.86 (-2.28 to -1.44)
Tropical Latin America	90 to 94	-1.54 (-2.27 to -0.82)
Tropical Latin America	95+	-1.47 (-3.04 to 0.13)
Western Europe	55 to 59	-2.97 (-3.21 to -2.73)
Western Europe	60 to 64	-3.19 (-3.37 to -3)
Western Europe	65 to 69	-3.44 (-3.61 to -3.27)
Western Europe	70 to 74	-3.65 (-3.84 to -3.46)

Western Europe	75 to 79	-3.75<br>(-3.97 to -3.53)
Western Europe	80 to 84	-3.49<br>(-3.76 to -3.22)
Western Europe	85 to 89	-2.84<br>(-3.22 to -2.46)
Western Europe	90 to 94	-2.04<br>(-2.7 to -1.38)
Western Europe	95+	-1.17<br>(-2.64 to 0.32)
Western Sub-Saharan Africa	55 to 59	-0.85<br>(-0.89 to -0.82)
Western Sub-Saharan Africa	60 to 64	-0.88<br>(-0.91 to -0.85)
Western Sub-Saharan Africa	65 to 69	-0.98<br>(-1.01 to -0.95)
Western Sub-Saharan Africa	70 to 74	-1.08<br>(-1.12 to -1.05)
Western Sub-Saharan Africa	75 to 79	-1.02<br>(-1.06 to -0.97)
Western Sub-Saharan Africa	80 to 84	-0.86<br>(-0.92 to -0.79)
Western Sub-Saharan Africa	85 to 89	-0.55<br>(-0.65 to -0.45)
Western Sub-Saharan Africa	90 to 94	-0.17<br>(-0.35 to 0.02)
Western Sub-Saharan Africa	95+	0.15<br>(-0.28 to 0.57)

Asthma<br>Location	Age	Prevalence rate (per 100,000 population)
Global	55 to 59	3374.31<br>(3313.47 to 3436.27)
Global	60 to 64	3553.6<br>(3489.72 to 3618.66)
Global	65 to 69	3661.35<br>(3583.86 to 3740.52)
Global	70 to 74	3604.33<br>(3524.85 to 3685.61)
Global	75 to 79	3312.23<br>(3234.09 to 3392.25)
Global	80 to 84	2969.16<br>(2890.55 to 3049.92)
Global	85 to 89	2830.98<br>(2740.25 to 2924.71)
Global	90 to 94	2950.14<br>(2821.14 to 3085.04)
Global	95+	3357.62<br>(3129.79 to 3602.04)
High SDI	55 to 59	6133.55<br>(5985.2 to 6285.57)
High SDI	60 to 64	6186.72<br>(6036.51 to 6340.67)
High SDI	65 to 69	6067.05<br>(5893.35 to 6245.87)
High SDI	70 to 74	5682.89<br>(5513.54 to 5857.44)
High SDI	75 to 79	4988.66<br>(4830.89 to 5151.59)
High SDI	80 to 84	4299.29<br>(4150.07 to 4453.87)
High SDI	85 to 89	4069.74<br>(3907.26 to 4238.98)
High SDI	90 to 94	4297.17<br>(4080.64 to 4525.19)

High SDI	95+	4924.19<br>(4568.61 to 5307.46)
High-middle SDI	55 to 59	2358.83<br>(2284.9 to 2435.15)
High-middle SDI	60 to 64	2314.59<br>(2241.98 to 2389.55)
High-middle SDI	65 to 69	2239.58<br>(2159.89 to 2322.22)
High-middle SDI	70 to 74	2075.43<br>(1999.33 to 2154.43)
High-middle SDI	75 to 79	1795.5<br>(1725.83 to 1867.99)
High-middle SDI	80 to 84	1524.94<br>(1459.31 to 1593.52)
High-middle SDI	85 to 89	1374.15<br>(1302.59 to 1449.63)
High-middle SDI	90 to 94	1349.8<br>(1250.93 to 1456.49)
High-middle SDI	95+	1573.14<br>(1384.81 to 1787.07)
Middle SDI	55 to 59	2161.05<br>(2118.82 to 2204.13)
Middle SDI	60 to 64	2347.87<br>(2302.3 to 2394.34)
Middle SDI	65 to 69	2579.21<br>(2519.88 to 2639.93)
Middle SDI	70 to 74	2742.34<br>(2676.77 to 2809.52)
Middle SDI	75 to 79	2667.51<br>(2599.34 to 2737.46)
Middle SDI	80 to 84	2467.23<br>(2395.87 to 2540.72)
Middle SDI	85 to 89	2280.77<br>(2197.16 to 2367.57)
Middle SDI	90 to 94	2147.94<br>(2028.73 to 2274.16)
Middle SDI	95+	2051.39<br>(1849.46 to 2275.37)
Low-middle SDI	55 to 59	3432.62<br>(3383.21 to 3482.77)
Low-middle SDI	60 to 64	3990.64<br>(3934.04 to 4048.05)
Low-middle SDI	65 to 69	4421.64<br>(4346.06 to 4498.53)
Low-middle SDI	70 to 74	4651.65<br>(4567.62 to 4737.23)
Low-middle SDI	75 to 79	4580.41<br>(4489.73 to 4672.92)
Low-middle SDI	80 to 84	4347.11<br>(4245.8 to 4450.85)
Low-middle SDI	85 to 89	4217.76<br>(4087.69 to 4351.97)
Low-middle SDI	90 to 94	4210.71<br>(4011.01 to 4420.35)
Low-middle SDI	95+	4182.07<br>(3840.27 to 4554.29)
Low SDI	55 to 59	3936.81<br>(3912.72 to 3961.06)
Low SDI	60 to 64	4505.19<br>(4477.79 to 4532.76)
Low SDI	65 to 69	4713.43<br>(4678.77 to 4748.34)
Low SDI	70 to 74	4633.85<br>(4597.28 to 4670.7)
Low SDI	75 to 79	4425.11<br>(4386.03 to 4464.54)

Low SDI	80 to 84	4202.07 (4156.81 to 4247.83)
Low SDI	85 to 89	4039.61 (3978.35 to 4101.82)
Low SDI	90 to 94	3928.83 (3827.45 to 4032.88)
Low SDI	95+	3846.61 (3647.03 to 4057.12)
Andean Latin America	55 to 59	1216.56 (1195.76 to 1237.73)
Andean Latin America	60 to 64	1259.49 (1237.68 to 1281.69)
Andean Latin America	65 to 69	1276.02 (1249.33 to 1303.28)
Andean Latin America	70 to 74	1267.18 (1239.25 to 1295.75)
Andean Latin America	75 to 79	1253.87 (1224.32 to 1284.13)
Andean Latin America	80 to 84	1263.81 (1230.96 to 1297.53)
Andean Latin America	85 to 89	1350.07 (1309.35 to 1392.06)
Andean Latin America	90 to 94	1489.01 (1431.15 to 1549.2)
Andean Latin America	95+	1573.04 (1483.13 to 1668.4)
Australasia	55 to 59	6374.82 (6075.5 to 6688.88)
Australasia	60 to 64	5564.82 (5300.05 to 5842.81)
Australasia	65 to 69	4892.01 (4618.41 to 5181.82)
Australasia	70 to 74	4342.73 (4088.82 to 4612.41)
Australasia	75 to 79	3879.29 (3638.42 to 4136.11)
Australasia	80 to 84	3567.08 (3326.74 to 3824.78)
Australasia	85 to 89	3692.85 (3413.75 to 3994.76)
Australasia	90 to 94	4243.78 (3853.16 to 4674)
Australasia	95+	5091.58 (4414.9 to 5871.97)
Caribbean	55 to 59	3807.94 (3770.86 to 3845.38)
Caribbean	60 to 64	3604.22 (3568.48 to 3640.32)
Caribbean	65 to 69	3322.11 (3282.5 to 3362.2)
Caribbean	70 to 74	2947.06 (2909.34 to 2985.27)
Caribbean	75 to 79	2446.66 (2411.62 to 2482.21)
Caribbean	80 to 84	1949.91 (1916.63 to 1983.77)
Caribbean	85 to 89	1726.23 (1688.97 to 1764.31)
Caribbean	90 to 94	1700.99 (1650.31 to 1753.23)
Caribbean	95+	1817.92 (1741.32 to 1897.9)
Central Asia	55 to 59	3430.84 (3398.72 to 3463.25)
Central Asia	60 to 64	3791.81 (3756.8 to 3827.14)

Central Asia	65 to 69	3977.01 (3932.77 to 4021.75)
Central Asia	70 to 74	3912.57 (3866.5 to 3959.2)
Central Asia	75 to 79	3415.05 (3370.46 to 3460.24)
Central Asia	80 to 84	2716.02 (2672.66 to 2760.09)
Central Asia	85 to 89	2145.23 (2096.88 to 2194.69)
Central Asia	90 to 94	1741.26 (1677.22 to 1807.74)
Central Asia	95+	1383.16 (1286.46 to 1487.12)
Central Europe	55 to 59	5291.12 (5183.5 to 5400.96)
Central Europe	60 to 64	5285.58 (5177.61 to 5395.79)
Central Europe	65 to 69	5235.93 (5114.32 to 5360.43)
Central Europe	70 to 74	4943.67 (4825.55 to 5064.68)
Central Europe	75 to 79	4083.51 (3979.66 to 4190.08)
Central Europe	80 to 84	3047.81 (2959.87 to 3138.36)
Central Europe	85 to 89	2223.64 (2141.04 to 2309.44)
Central Europe	90 to 94	1652.85 (1554.47 to 1757.46)
Central Europe	95+	1250.03 (1103.96 to 1415.42)
Central Latin America	55 to 59	1605.87 (1551.72 to 1661.92)
Central Latin America	60 to 64	1498.14 (1446.82 to 1551.28)
Central Latin America	65 to 69	1359.35 (1304.14 to 1416.9)
Central Latin America	70 to 74	1177.98 (1127.39 to 1230.84)
Central Latin America	75 to 79	964.68 (919.53 to 1012.05)
Central Latin America	80 to 84	789.57 (747.51 to 833.99)
Central Latin America	85 to 89	735.45 (689.16 to 784.86)
Central Latin America	90 to 94	723.15 (664.3 to 787.23)
Central Latin America	95+	714.19 (634.91 to 803.38)
Central Sub-Saharan Africa	55 to 59	3178.15 (3159.96 to 3196.45)
Central Sub-Saharan Africa	60 to 64	3552.28 (3531.67 to 3573)
Central Sub-Saharan Africa	65 to 69	3721.82 (3695.19 to 3748.63)
Central Sub-Saharan Africa	70 to 74	3693.91 (3665.13 to 3722.92)
Central Sub-Saharan Africa	75 to 79	3476.92 (3445.74 to 3508.38)
Central Sub-Saharan Africa	80 to 84	3234.44 (3197.46 to 3271.85)
Central Sub-Saharan Africa	85 to 89	3238.29 (3184.21 to 3293.28)
Central Sub-Saharan Africa	90 to 94	3400.42 (3300.23 to 3503.64)

Central Sub-Saharan Africa	95+	3641.14 (3416.67 to 3880.35)
East Asia	55 to 59	1203.48 (1159.64 to 1248.97)
East Asia	60 to 64	1299.06 (1252.22 to 1347.64)
East Asia	65 to 69	1544.53 (1480.79 to 1611.01)
East Asia	70 to 74	1776.51 (1701.83 to 1854.46)
East Asia	75 to 79	1810.8 (1731.72 to 1893.5)
East Asia	80 to 84	1734.43 (1652.13 to 1820.82)
East Asia	85 to 89	1604.43 (1511.89 to 1702.63)
East Asia	90 to 94	1490.52 (1361.44 to 1631.85)
East Asia	95+	1497.12 (1249.11 to 1794.37)
Eastern Europe	55 to 59	2407.2 (2343.76 to 2472.36)
Eastern Europe	60 to 64	2010.84 (1957.01 to 2066.14)
Eastern Europe	65 to 69	1542.89 (1497.28 to 1589.88)
Eastern Europe	70 to 74	1083.31 (1049.39 to 1118.32)
Eastern Europe	75 to 79	681.76 (657.9 to 706.49)
Eastern Europe	80 to 84	399.04 (382.06 to 416.77)
Eastern Europe	85 to 89	251.79 (236.78 to 267.76)
Eastern Europe	90 to 94	183.07 (164.86 to 203.29)
Eastern Europe	95+	149.52 (118.96 to 187.93)
Eastern Sub-Saharan Africa	55 to 59	3630.71 (3613.14 to 3648.37)
Eastern Sub-Saharan Africa	60 to 64	4063.77 (4044.11 to 4083.52)
Eastern Sub-Saharan Africa	65 to 69	4101.56 (4077.29 to 4125.97)
Eastern Sub-Saharan Africa	70 to 74	3829.39 (3804.74 to 3854.2)
Eastern Sub-Saharan Africa	75 to 79	3489.9 (3464.28 to 3515.71)
Eastern Sub-Saharan Africa	80 to 84	3190.27 (3160.98 to 3219.84)
Eastern Sub-Saharan Africa	85 to 89	2976.61 (2937.1 to 3016.65)
Eastern Sub-Saharan Africa	90 to 94	2830.61 (2765.21 to 2897.55)
Eastern Sub-Saharan Africa	95+	2711.12 (2584.17 to 2844.3)
High-income Asia Pacific	55 to 59	2580.67 (2232.81 to 2982.73)
High-income Asia Pacific	60 to 64	2374.76 (2052.14 to 2748.12)
High-income Asia Pacific	65 to 69	2190.89 (1870.98 to 2565.5)
High-income Asia Pacific	70 to 74	1961.06 (1671.1 to 2301.35)
High-income Asia Pacific	75 to 79	1681.5 (1427.75 to 1980.34)

High-income Asia Pacific	80 to 84	1434.62 (1210.98 to 1699.56)
High-income Asia Pacific	85 to 89	1329.35 (1111.58 to 1589.78)
High-income Asia Pacific	90 to 94	1331.94 (1092.45 to 1623.93)
High-income Asia Pacific	95+	1380.57 (1088.96 to 1750.25)
High-income North America	55 to 59	8204.43 (7954.07 to 8462.67)
High-income North America	60 to 64	9474.61 (9184.58 to 9773.8)
High-income North America	65 to 69	10906.5 (10482.28 to 11347.89)
High-income North America	70 to 74	12341.6 (11829.1 to 12876.31)
High-income North America	75 to 79	13192.11 (12603.99 to 13807.68)
High-income North America	80 to 84	13683.38 (13008.28 to 14393.52)
High-income North America	85 to 89	14982.55 (14122.91 to 15894.51)
High-income North America	90 to 94	17358.1 (16077.75 to 18740.41)
High-income North America	95+	20974.52 (18633.27 to 23609.96)
North Africa and Middle East	55 to 59	3762.61 (3686.97 to 3839.8)
North Africa and Middle East	60 to 64	4072.04 (3990.43 to 4155.32)
North Africa and Middle East	65 to 69	4375.24 (4270.05 to 4483.02)
North Africa and Middle East	70 to 74	4610.14 (4493.77 to 4729.53)
North Africa and Middle East	75 to 79	4591.72 (4466.36 to 4720.59)
North Africa and Middle East	80 to 84	4482.58 (4342.76 to 4626.9)
North Africa and Middle East	85 to 89	4637.06 (4454.46 to 4827.16)
North Africa and Middle East	90 to 94	5192.8 (4892.33 to 5511.72)
North Africa and Middle East	95+	6608.74 (6049.49 to 7219.68)
Oceania	55 to 59	3836.91 (3782.9 to 3891.69)
Oceania	60 to 64	4307.11 (4246.08 to 4369.01)
Oceania	65 to 69	4992.13 (4906.69 to 5079.07)
Oceania	70 to 74	5508.82 (5409.23 to 5610.25)
Oceania	75 to 79	5124.08 (5020.85 to 5229.42)
Oceania	80 to 84	4316.16 (4209.2 to 4425.83)
Oceania	85 to 89	3858.5 (3724.38 to 3997.44)
Oceania	90 to 94	3695.02 (3479.14 to 3924.29)
Oceania	95+	3470.62 (3077.81 to 3913.56)
South Asia	55 to 59	3393.71 (3324.09 to 3464.79)
South Asia	60 to 64	3982.46 (3902.29 to 4064.29)

South Asia	65 to 69	4502.37 (4393.27 to 4614.18)
South Asia	70 to 74	4870.37 (4745.96 to 4998.03)
South Asia	75 to 79	4964.43 (4826.17 to 5106.65)
South Asia	80 to 84	4889.34 (4730.51 to 5053.51)
South Asia	85 to 89	4888.58 (4680.14 to 5106.32)
South Asia	90 to 94	4955.63 (4628.87 to 5305.46)
South Asia	95+	5045.82 (4431.95 to 5744.72)
Southeast Asia	55 to 59	3157.98 (3127.28 to 3188.97)
Southeast Asia	60 to 64	3603.63 (3568.88 to 3638.72)
Southeast Asia	65 to 69	3954.85 (3908.81 to 4001.42)
Southeast Asia	70 to 74	4115 (4064.47 to 4166.15)
Southeast Asia	75 to 79	3843.22 (3791.41 to 3895.73)
Southeast Asia	80 to 84	3397.01 (3342.88 to 3452.03)
Southeast Asia	85 to 89	3164.91 (3098.47 to 3232.78)
Southeast Asia	90 to 94	3131.95 (3031.21 to 3236.04)
Southeast Asia	95+	3174.93 (2998.74 to 3361.47)
Southern Latin America	55 to 59	6998.57 (6934.34 to 7063.39)
Southern Latin America	60 to 64	7938.81 (7867.18 to 8011.09)
Southern Latin America	65 to 69	8458.74 (8369.45 to 8548.99)
Southern Latin America	70 to 74	8521.45 (8428.36 to 8615.57)
Southern Latin America	75 to 79	8062.8 (7969.49 to 8157.19)
Southern Latin America	80 to 84	7434.7 (7340.06 to 7530.56)
Southern Latin America	85 to 89	7321.5 (7213.09 to 7431.53)
Southern Latin America	90 to 94	7707.76 (7557.08 to 7861.44)
Southern Latin America	95+	8336.69 (8087.51 to 8593.56)
Southern Sub-Saharan Africa	55 to 59	2273.29 (2244.42 to 2302.52)
Southern Sub-Saharan Africa	60 to 64	2316.43 (2286.4 to 2346.86)
Southern Sub-Saharan Africa	65 to 69	2311.19 (2275.25 to 2347.7)
Southern Sub-Saharan Africa	70 to 74	2211.31 (2174.42 to 2248.83)
Southern Sub-Saharan Africa	75 to 79	2020.75 (1983.02 to 2059.19)
Southern Sub-Saharan Africa	80 to 84	1871.11 (1830.28 to 1912.85)
Southern Sub-Saharan Africa	85 to 89	1948.96 (1894.66 to 2004.82)
Southern Sub-Saharan Africa	90 to 94	2116.34 (2024.46 to 2212.38)

Southern Sub-Saharan Africa	95+	2229.88<br>(2032.63 to 2446.28)
Tropical Latin America	55 to 59	2140.89<br>(2059.8 to 2225.17)
Tropical Latin America	60 to 64	1957.91<br>(1882.62 to 2036.21)
Tropical Latin America	65 to 69	1846.42<br>(1762.44 to 1934.41)
Tropical Latin America	70 to 74	1735.8<br>(1652.8 to 1822.97)
Tropical Latin America	75 to 79	1515.43<br>(1436.72 to 1598.46)
Tropical Latin America	80 to 84	1305.1<br>(1227.95 to 1387.09)
Tropical Latin America	85 to 89	1288.14<br>(1197.21 to 1385.98)
Tropical Latin America	90 to 94	1401.1<br>(1272.56 to 1542.62)
Tropical Latin America	95+	1573.81<br>(1373.68 to 1803.1)
Western Europe	55 to 59	6232.37<br>(5933.92 to 6545.83)
Western Europe	60 to 64	5778.65<br>(5499.33 to 6072.16)
Western Europe	65 to 69	5005.98<br>(4731.43 to 5296.46)
Western Europe	70 to 74	4040.72<br>(3810.41 to 4284.96)
Western Europe	75 to 79	3081.98<br>(2894.46 to 3281.66)
Western Europe	80 to 84	2321.45<br>(2165.19 to 2489)
Western Europe	85 to 89	1952.81<br>(1799.38 to 2119.32)
Western Europe	90 to 94	1876.56<br>(1685.27 to 2089.56)
Western Europe	95+	1980.72<br>(1675.43 to 2341.64)
Western Sub-Saharan Africa	55 to 59	3880.25<br>(3854.71 to 3905.95)
Western Sub-Saharan Africa	60 to 64	4878.59<br>(4847.08 to 4910.3)
Western Sub-Saharan Africa	65 to 69	4887.02<br>(4847.64 to 4926.72)
Western Sub-Saharan Africa	70 to 74	4362.78<br>(4324.27 to 4401.62)
Western Sub-Saharan Africa	75 to 79	4339.92<br>(4297.39 to 4382.87)
Western Sub-Saharan Africa	80 to 84	4672.65<br>(4618.89 to 4727.03)
Western Sub-Saharan Africa	85 to 89	4785.35<br>(4711.88 to 4859.96)
Western Sub-Saharan Africa	90 to 94	4701<br>(4584.48 to 4820.49)
Western Sub-Saharan Africa	95+	4611.89<br>(4395.91 to 4838.48)

Asthma<br>Location	Period	Prevalence rate ratio
Global	1992 to 1996	1.69<br>(1.65 to 1.72)
Global	1997 to 2001	1.4<br>(1.37 to 1.42)
Global	2002 to 2006	1.2<br>(1.18 to 1.22)

Global	2007 to 2011	1.1<br>(1.08 to 1.12)
Global	2012 to 2016	1<br>(1 to 1)
Global	2017 to 2021	0.96<br>(0.95 to 0.98)
High SDI	1992 to 1996	1.67<br>(1.63 to 1.71)
High SDI	1997 to 2001	1.29<br>(1.26 to 1.32)
High SDI	2002 to 2006	1.08<br>(1.05 to 1.1)
High SDI	2007 to 2011	1.04<br>(1.02 to 1.06)
High SDI	2012 to 2016	1<br>(1 to 1)
High SDI	2017 to 2021	0.97<br>(0.95 to 1)
High-middle SDI	1992 to 1996	2.07<br>(2 to 2.15)
High-middle SDI	1997 to 2001	1.7<br>(1.65 to 1.76)
High-middle SDI	2002 to 2006	1.38<br>(1.34 to 1.42)
High-middle SDI	2007 to 2011	1.18<br>(1.15 to 1.21)
High-middle SDI	2012 to 2016	1<br>(1 to 1)
High-middle SDI	2017 to 2021	0.97<br>(0.94 to 0.99)
Middle SDI	1992 to 1996	1.61<br>(1.56 to 1.65)
Middle SDI	1997 to 2001	1.46<br>(1.43 to 1.5)
Middle SDI	2002 to 2006	1.3<br>(1.27 to 1.32)
Middle SDI	2007 to 2011	1.16<br>(1.14 to 1.18)
Middle SDI	2012 to 2016	1<br>(1 to 1)
Middle SDI	2017 to 2021	0.95<br>(0.93 to 0.97)
Low-middle SDI	1992 to 1996	1.24<br>(1.21 to 1.27)
Low-middle SDI	1997 to 2001	1.14<br>(1.11 to 1.16)
Low-middle SDI	2002 to 2006	1.1<br>(1.08 to 1.12)
Low-middle SDI	2007 to 2011	1.05<br>(1.04 to 1.07)
Low-middle SDI	2012 to 2016	1<br>(1 to 1)
Low-middle SDI	2017 to 2021	0.99<br>(0.98 to 1.01)
Low SDI	1992 to 1996	1.24<br>(1.22 to 1.25)
Low SDI	1997 to 2001	1.17<br>(1.16 to 1.18)
Low SDI	2002 to 2006	1.12<br>(1.11 to 1.13)
Low SDI	2007 to 2011	1.07<br>(1.06 to 1.08)
Low SDI	2012 to 2016	1<br>(1 to 1)
Low SDI	2017 to 2021	0.99<br>(0.98 to 1)

Andean Latin America	1992 to 1996	1.38<br>(1.35 to 1.41)
Andean Latin America	1997 to 2001	1.3<br>(1.27 to 1.32)
Andean Latin America	2002 to 2006	1.19<br>(1.17 to 1.22)
Andean Latin America	2007 to 2011	1.1<br>(1.08 to 1.12)
Andean Latin America	2012 to 2016	1<br>(1 to 1)
Andean Latin America	2017 to 2021	0.94<br>(0.92 to 0.95)
Australasia	1992 to 1996	1.62<br>(1.54 to 1.7)
Australasia	1997 to 2001	1.47<br>(1.4 to 1.55)
Australasia	2002 to 2006	1.35<br>(1.29 to 1.41)
Australasia	2007 to 2011	1.1<br>(1.06 to 1.15)
Australasia	2012 to 2016	1<br>(1 to 1)
Australasia	2017 to 2021	0.98<br>(0.94 to 1.03)
Caribbean	1992 to 1996	1.35<br>(1.33 to 1.37)
Caribbean	1997 to 2001	1.29<br>(1.28 to 1.31)
Caribbean	2002 to 2006	1.21<br>(1.2 to 1.22)
Caribbean	2007 to 2011	1.12<br>(1.11 to 1.13)
Caribbean	2012 to 2016	1<br>(1 to 1)
Caribbean	2017 to 2021	0.98<br>(0.97 to 0.98)
Central Asia	1992 to 1996	1.42<br>(1.4 to 1.45)
Central Asia	1997 to 2001	1.35<br>(1.33 to 1.37)
Central Asia	2002 to 2006	1.21<br>(1.2 to 1.23)
Central Asia	2007 to 2011	1.1<br>(1.09 to 1.11)
Central Asia	2012 to 2016	1<br>(1 to 1)
Central Asia	2017 to 2021	1.01<br>(1 to 1.02)
Central Europe	1992 to 1996	2.13<br>(2.08 to 2.19)
Central Europe	1997 to 2001	1.75<br>(1.71 to 1.79)
Central Europe	2002 to 2006	1.36<br>(1.33 to 1.38)
Central Europe	2007 to 2011	1.12<br>(1.1 to 1.14)
Central Europe	2012 to 2016	1<br>(1 to 1)
Central Europe	2017 to 2021	0.94<br>(0.92 to 0.96)
Central Latin America	1992 to 1996	2.36<br>(2.27 to 2.45)
Central Latin America	1997 to 2001	1.96<br>(1.89 to 2.03)
Central Latin America	2002 to 2006	1.56<br>(1.51 to 1.62)

Central Latin America	2007 to 2011	1.26<br>(1.22 to 1.31)
Central Latin America	2012 to 2016	1<br>(1 to 1)
Central Latin America	2017 to 2021	0.89<br>(0.87 to 0.92)
Central Sub-Saharan Africa	1992 to 1996	1.15<br>(1.13 to 1.17)
Central Sub-Saharan Africa	1997 to 2001	1.12<br>(1.11 to 1.14)
Central Sub-Saharan Africa	2002 to 2006	1.09<br>(1.08 to 1.1)
Central Sub-Saharan Africa	2007 to 2011	1.06<br>(1.05 to 1.07)
Central Sub-Saharan Africa	2012 to 2016	1<br>(1 to 1)
Central Sub-Saharan Africa	2017 to 2021	0.96<br>(0.96 to 0.97)
East Asia	1992 to 1996	1.91<br>(1.81 to 2.01)
East Asia	1997 to 2001	1.67<br>(1.6 to 1.74)
East Asia	2002 to 2006	1.37<br>(1.32 to 1.42)
East Asia	2007 to 2011	1.18<br>(1.15 to 1.22)
East Asia	2012 to 2016	1<br>(1 to 1)
East Asia	2017 to 2021	0.93<br>(0.91 to 0.96)
Eastern Europe	1992 to 1996	3.16<br>(3.04 to 3.29)
Eastern Europe	1997 to 2001	2.56<br>(2.47 to 2.65)
Eastern Europe	2002 to 2006	1.87<br>(1.82 to 1.93)
Eastern Europe	2007 to 2011	1.39<br>(1.35 to 1.43)
Eastern Europe	2012 to 2016	1<br>(1 to 1)
Eastern Europe	2017 to 2021	0.85<br>(0.83 to 0.88)
Eastern Sub-Saharan Africa	1992 to 1996	1.25<br>(1.24 to 1.26)
Eastern Sub-Saharan Africa	1997 to 2001	1.21<br>(1.19 to 1.22)
Eastern Sub-Saharan Africa	2002 to 2006	1.14<br>(1.13 to 1.14)
Eastern Sub-Saharan Africa	2007 to 2011	1.07<br>(1.07 to 1.08)
Eastern Sub-Saharan Africa	2012 to 2016	1<br>(1 to 1)
Eastern Sub-Saharan Africa	2017 to 2021	0.99<br>(0.98 to 0.99)
High-income Asia Pacific	1992 to 1996	3.64<br>(3.34 to 3.97)
High-income Asia Pacific	1997 to 2001	2.66<br>(2.44 to 2.9)
High-income Asia Pacific	2002 to 2006	1.74<br>(1.6 to 1.9)
High-income Asia Pacific	2007 to 2011	1.3<br>(1.19 to 1.42)
High-income Asia Pacific	2012 to 2016	1<br>(1 to 1)
High-income Asia Pacific	2017 to 2021	0.88<br>(0.8 to 0.97)

High-income North America	1992 to 1996	0.83<br>(0.8 to 0.86)
High-income North America	1997 to 2001	0.63<br>(0.61 to 0.66)
High-income North America	2002 to 2006	0.63<br>(0.61 to 0.66)
High-income North America	2007 to 2011	0.84<br>(0.81 to 0.86)
High-income North America	2012 to 2016	1<br>(1 to 1)
High-income North America	2017 to 2021	1.04<br>(1.02 to 1.07)
North Africa and Middle East	1992 to 1996	1.31<br>(1.27 to 1.34)
North Africa and Middle East	1997 to 2001	1.21<br>(1.18 to 1.25)
North Africa and Middle East	2002 to 2006	1.14<br>(1.11 to 1.16)
North Africa and Middle East	2007 to 2011	1.07<br>(1.05 to 1.09)
North Africa and Middle East	2012 to 2016	1<br>(1 to 1)
North Africa and Middle East	2017 to 2021	1.03<br>(1.01 to 1.05)
Oceania	1992 to 1996	1.43<br>(1.38 to 1.47)
Oceania	1997 to 2001	1.36<br>(1.32 to 1.39)
Oceania	2002 to 2006	1.24<br>(1.22 to 1.27)
Oceania	2007 to 2011	1.12<br>(1.1 to 1.13)
Oceania	2012 to 2016	1<br>(1 to 1)
Oceania	2017 to 2021	0.92<br>(0.91 to 0.94)
South Asia	1992 to 1996	1.24<br>(1.19 to 1.28)
South Asia	1997 to 2001	1.13<br>(1.09 to 1.16)
South Asia	2002 to 2006	1.11<br>(1.08 to 1.14)
South Asia	2007 to 2011	1.07<br>(1.05 to 1.09)
South Asia	2012 to 2016	1<br>(1 to 1)
South Asia	2017 to 2021	0.98<br>(0.96 to 1)
Southeast Asia	1992 to 1996	1.37<br>(1.35 to 1.39)
Southeast Asia	1997 to 2001	1.25<br>(1.24 to 1.27)
Southeast Asia	2002 to 2006	1.17<br>(1.16 to 1.19)
Southeast Asia	2007 to 2011	1.11<br>(1.1 to 1.12)
Southeast Asia	2012 to 2016	1<br>(1 to 1)
Southeast Asia	2017 to 2021	0.97<br>(0.96 to 0.98)
Southern Latin America	1992 to 1996	1.32<br>(1.31 to 1.33)
Southern Latin America	1997 to 2001	1.28<br>(1.27 to 1.3)
Southern Latin America	2002 to 2006	1.18<br>(1.17 to 1.19)

Southern Latin America	2007 to 2011	1.08 (1.07 to 1.09)
Southern Latin America	2012 to 2016	1 (1 to 1)
Southern Latin America	2017 to 2021	1.02 (1.01 to 1.03)
Southern Sub-Saharan Africa	1992 to 1996	1.27 (1.25 to 1.3)
Southern Sub-Saharan Africa	1997 to 2001	1.31 (1.28 to 1.33)
Southern Sub-Saharan Africa	2002 to 2006	1.27 (1.25 to 1.29)
Southern Sub-Saharan Africa	2007 to 2011	1.17 (1.16 to 1.19)
Southern Sub-Saharan Africa	2012 to 2016	1 (1 to 1)
Southern Sub-Saharan Africa	2017 to 2021	0.92 (0.91 to 0.93)
Tropical Latin America	1992 to 1996	1.71 (1.63 to 1.8)
Tropical Latin America	1997 to 2001	1.66 (1.59 to 1.74)
Tropical Latin America	2002 to 2006	1.5 (1.44 to 1.56)
Tropical Latin America	2007 to 2011	1.15 (1.11 to 1.19)
Tropical Latin America	2012 to 2016	1 (1 to 1)
Tropical Latin America	2017 to 2021	0.98 (0.95 to 1.02)
Western Europe	1992 to 1996	2.07 (1.97 to 2.17)
Western Europe	1997 to 2001	1.59 (1.52 to 1.67)
Western Europe	2002 to 2006	1.36 (1.3 to 1.42)
Western Europe	2007 to 2011	1.21 (1.16 to 1.26)
Western Europe	2012 to 2016	1 (1 to 1)
Western Europe	2017 to 2021	0.94 (0.9 to 0.98)
Western Sub-Saharan Africa	1992 to 1996	1.23 (1.21 to 1.24)
Western Sub-Saharan Africa	1997 to 2001	1.13 (1.12 to 1.14)
Western Sub-Saharan Africa	2002 to 2006	1.07 (1.06 to 1.08)
Western Sub-Saharan Africa	2007 to 2011	1.04 (1.04 to 1.05)
Western Sub-Saharan Africa	2012 to 2016	1 (1 to 1)
Western Sub-Saharan Africa	2017 to 2021	1.02 (1.01 to 1.03)

Asthma<br>Location	Cohort	Prevalence rate ratio
Global	1897 to 1901	2.88 (2.29 to 3.62)
Global	1902 to 1906	3.03 (2.74 to 3.35)
Global	1907 to 1911	3.06 (2.89 to 3.24)
Global	1912 to 1916	2.97 (2.86 to 3.09)

Global	1917 to 1921	2.72<br>(2.64 to 2.81)
Global	1922 to 1926	2.45<br>(2.38 to 2.51)
Global	1927 to 1931	2.15<br>(2.1 to 2.2)
Global	1932 to 1936	1.86<br>(1.82 to 1.9)
Global	1937 to 1941	1.61<br>(1.58 to 1.64)
Global	1942 to 1946	1.44<br>(1.41 to 1.47)
Global	1947 to 1951	1.27<br>(1.25 to 1.3)
Global	1952 to 1956	1.11<br>(1.08 to 1.13)
Global	1957 to 1961	1<br>(1 to 1)
Global	1962 to 1966	0.89<br>(0.86 to 0.91)
High SDI	1897 to 1901	2.31<br>(1.83 to 2.91)
High SDI	1902 to 1906	2.54<br>(2.28 to 2.84)
High SDI	1907 to 1911	2.68<br>(2.51 to 2.86)
High SDI	1912 to 1916	2.67<br>(2.54 to 2.8)
High SDI	1917 to 1921	2.47<br>(2.37 to 2.57)
High SDI	1922 to 1926	2.18<br>(2.11 to 2.26)
High SDI	1927 to 1931	1.92<br>(1.86 to 1.98)
High SDI	1932 to 1936	1.65<br>(1.6 to 1.7)
High SDI	1937 to 1941	1.43<br>(1.39 to 1.47)
High SDI	1942 to 1946	1.3<br>(1.26 to 1.34)
High SDI	1947 to 1951	1.21<br>(1.17 to 1.25)
High SDI	1952 to 1956	1.1<br>(1.06 to 1.13)
High SDI	1957 to 1961	1<br>(1 to 1)
High SDI	1962 to 1966	0.91<br>(0.87 to 0.94)
High-middle SDI	1897 to 1901	4.86<br>(3.16 to 7.48)
High-middle SDI	1902 to 1906	5.29<br>(4.44 to 6.31)
High-middle SDI	1907 to 1911	5.27<br>(4.8 to 5.79)
High-middle SDI	1912 to 1916	5.01<br>(4.7 to 5.33)
High-middle SDI	1917 to 1921	4.61<br>(4.38 to 4.85)
High-middle SDI	1922 to 1926	4.04<br>(3.87 to 4.22)
High-middle SDI	1927 to 1931	3.45<br>(3.32 to 3.59)
High-middle SDI	1932 to 1936	2.9<br>(2.8 to 3.01)
High-middle SDI	1937 to 1941	2.34<br>(2.26 to 2.43)

High-middle SDI	1942 to 1946	1.95 (1.88 to 2.02)
High-middle SDI	1947 to 1951	1.54 (1.48 to 1.6)
High-middle SDI	1952 to 1956	1.21 (1.16 to 1.25)
High-middle SDI	1957 to 1961	1 (1 to 1)
High-middle SDI	1962 to 1966	0.83 (0.79 to 0.88)
Middle SDI	1897 to 1901	3.29 (2.3 to 4.7)
Middle SDI	1902 to 1906	3.06 (2.64 to 3.55)
Middle SDI	1907 to 1911	2.88 (2.67 to 3.11)
Middle SDI	1912 to 1916	2.74 (2.61 to 2.87)
Middle SDI	1917 to 1921	2.56 (2.47 to 2.65)
Middle SDI	1922 to 1926	2.34 (2.27 to 2.41)
Middle SDI	1927 to 1931	2.07 (2.02 to 2.13)
Middle SDI	1932 to 1936	1.82 (1.78 to 1.87)
Middle SDI	1937 to 1941	1.59 (1.55 to 1.63)
Middle SDI	1942 to 1946	1.38 (1.35 to 1.42)
Middle SDI	1947 to 1951	1.2 (1.17 to 1.23)
Middle SDI	1952 to 1956	1.07 (1.04 to 1.09)
Middle SDI	1957 to 1961	1 (1 to 1)
Middle SDI	1962 to 1966	0.9 (0.87 to 0.93)
Low-middle SDI	1897 to 1901	1.62 (1.21 to 2.15)
Low-middle SDI	1902 to 1906	1.59 (1.41 to 1.8)
Low-middle SDI	1907 to 1911	1.59 (1.49 to 1.7)
Low-middle SDI	1912 to 1916	1.54 (1.48 to 1.61)
Low-middle SDI	1917 to 1921	1.5 (1.45 to 1.54)
Low-middle SDI	1922 to 1926	1.45 (1.42 to 1.49)
Low-middle SDI	1927 to 1931	1.4 (1.37 to 1.43)
Low-middle SDI	1932 to 1936	1.34 (1.32 to 1.37)
Low-middle SDI	1937 to 1941	1.27 (1.25 to 1.3)
Low-middle SDI	1942 to 1946	1.19 (1.17 to 1.22)
Low-middle SDI	1947 to 1951	1.12 (1.1 to 1.14)
Low-middle SDI	1952 to 1956	1.05 (1.03 to 1.07)
Low-middle SDI	1957 to 1961	1 (1 to 1)
Low-middle SDI	1962 to 1966	0.96 (0.93 to 0.98)

Low SDI	1897 to 1901	1.63<br>(1.37 to 1.93)
Low SDI	1902 to 1906	1.65<br>(1.54 to 1.77)
Low SDI	1907 to 1911	1.66<br>(1.6 to 1.72)
Low SDI	1912 to 1916	1.6<br>(1.57 to 1.63)
Low SDI	1917 to 1921	1.53<br>(1.51 to 1.55)
Low SDI	1922 to 1926	1.48<br>(1.46 to 1.5)
Low SDI	1927 to 1931	1.43<br>(1.41 to 1.44)
Low SDI	1932 to 1936	1.37<br>(1.36 to 1.38)
Low SDI	1937 to 1941	1.3<br>(1.29 to 1.31)
Low SDI	1942 to 1946	1.22<br>(1.21 to 1.23)
Low SDI	1947 to 1951	1.13<br>(1.12 to 1.14)
Low SDI	1952 to 1956	1.06<br>(1.05 to 1.07)
Low SDI	1957 to 1961	1<br>(1 to 1)
Low SDI	1962 to 1966	0.94<br>(0.93 to 0.95)
Andean Latin America	1897 to 1901	2.08<br>(1.73 to 2.5)
Andean Latin America	1902 to 1906	2.16<br>(1.98 to 2.36)
Andean Latin America	1907 to 1911	2.21<br>(2.09 to 2.34)
Andean Latin America	1912 to 1916	2.19<br>(2.1 to 2.28)
Andean Latin America	1917 to 1921	2.07<br>(2 to 2.13)
Andean Latin America	1922 to 1926	1.9<br>(1.85 to 1.96)
Andean Latin America	1927 to 1931	1.73<br>(1.69 to 1.78)
Andean Latin America	1932 to 1936	1.58<br>(1.54 to 1.61)
Andean Latin America	1937 to 1941	1.41<br>(1.38 to 1.44)
Andean Latin America	1942 to 1946	1.27<br>(1.25 to 1.3)
Andean Latin America	1947 to 1951	1.16<br>(1.13 to 1.18)
Andean Latin America	1952 to 1956	1.07<br>(1.05 to 1.09)
Andean Latin America	1957 to 1961	1<br>(1 to 1)
Andean Latin America	1962 to 1966	0.95<br>(0.92 to 0.97)
Australasia	1897 to 1901	2.91<br>(1.81 to 4.69)
Australasia	1902 to 1906	3.03<br>(2.43 to 3.78)
Australasia	1907 to 1911	3.13<br>(2.74 to 3.57)
Australasia	1912 to 1916	3.12<br>(2.84 to 3.44)
Australasia	1917 to 1921	2.98<br>(2.75 to 3.23)

Australasia	1922 to 1926	2.73<br>(2.54 to 2.92)
Australasia	1927 to 1931	2.43<br>(2.27 to 2.59)
Australasia	1932 to 1936	2.11<br>(1.99 to 2.24)
Australasia	1937 to 1941	1.81<br>(1.71 to 1.92)
Australasia	1942 to 1946	1.58<br>(1.49 to 1.68)
Australasia	1947 to 1951	1.38<br>(1.3 to 1.46)
Australasia	1952 to 1956	1.18<br>(1.11 to 1.25)
Australasia	1957 to 1961	1<br>(1 to 1)
Australasia	1962 to 1966	0.86<br>(0.79 to 0.93)
Caribbean	1897 to 1901	2.19<br>(1.85 to 2.59)
Caribbean	1902 to 1906	2.1<br>(1.95 to 2.25)
Caribbean	1907 to 1911	2.06<br>(1.98 to 2.15)
Caribbean	1912 to 1916	1.99<br>(1.93 to 2.04)
Caribbean	1917 to 1921	1.83<br>(1.8 to 1.87)
Caribbean	1922 to 1926	1.7<br>(1.68 to 1.73)
Caribbean	1927 to 1931	1.58<br>(1.56 to 1.6)
Caribbean	1932 to 1936	1.47<br>(1.45 to 1.48)
Caribbean	1937 to 1941	1.36<br>(1.34 to 1.37)
Caribbean	1942 to 1946	1.26<br>(1.24 to 1.27)
Caribbean	1947 to 1951	1.17<br>(1.16 to 1.18)
Caribbean	1952 to 1956	1.09<br>(1.07 to 1.1)
Caribbean	1957 to 1961	1<br>(1 to 1)
Caribbean	1962 to 1966	0.92<br>(0.9 to 0.93)
Central Asia	1897 to 1901	2.5<br>(2.07 to 3.01)
Central Asia	1902 to 1906	2.37<br>(2.2 to 2.55)
Central Asia	1907 to 1911	2.21<br>(2.12 to 2.3)
Central Asia	1912 to 1916	2.04<br>(1.98 to 2.09)
Central Asia	1917 to 1921	1.95<br>(1.91 to 1.99)
Central Asia	1922 to 1926	1.86<br>(1.84 to 1.89)
Central Asia	1927 to 1931	1.72<br>(1.69 to 1.74)
Central Asia	1932 to 1936	1.63<br>(1.61 to 1.65)
Central Asia	1937 to 1941	1.45<br>(1.44 to 1.47)
Central Asia	1942 to 1946	1.36<br>(1.35 to 1.38)

Central Asia	1947 to 1951	1.2<br>(1.18 to 1.21)
Central Asia	1952 to 1956	1.1<br>(1.09 to 1.12)
Central Asia	1957 to 1961	1<br>(1 to 1)
Central Asia	1962 to 1966	0.89<br>(0.87 to 0.9)
Central Europe	1897 to 1901	6.76<br>(4.81 to 9.5)
Central Europe	1902 to 1906	5.93<br>(5.22 to 6.74)
Central Europe	1907 to 1911	5.25<br>(4.92 to 5.59)
Central Europe	1912 to 1916	4.39<br>(4.21 to 4.58)
Central Europe	1917 to 1921	3.75<br>(3.62 to 3.88)
Central Europe	1922 to 1926	3.1<br>(3.01 to 3.18)
Central Europe	1927 to 1931	2.63<br>(2.57 to 2.7)
Central Europe	1932 to 1936	2.2<br>(2.15 to 2.25)
Central Europe	1937 to 1941	1.79<br>(1.75 to 1.83)
Central Europe	1942 to 1946	1.46<br>(1.42 to 1.49)
Central Europe	1947 to 1951	1.27<br>(1.24 to 1.31)
Central Europe	1952 to 1956	1.13<br>(1.11 to 1.16)
Central Europe	1957 to 1961	1<br>(1 to 1)
Central Europe	1962 to 1966	0.86<br>(0.83 to 0.89)
Central Latin America	1897 to 1901	9.06<br>(6.26 to 13.11)
Central Latin America	1902 to 1906	8.2<br>(6.88 to 9.77)
Central Latin America	1907 to 1911	7.3<br>(6.57 to 8.1)
Central Latin America	1912 to 1916	6.13<br>(5.67 to 6.63)
Central Latin America	1917 to 1921	4.98<br>(4.67 to 5.3)
Central Latin America	1922 to 1926	3.99<br>(3.78 to 4.21)
Central Latin America	1927 to 1931	3.19<br>(3.04 to 3.35)
Central Latin America	1932 to 1936	2.55<br>(2.45 to 2.67)
Central Latin America	1937 to 1941	2.03<br>(1.95 to 2.11)
Central Latin America	1942 to 1946	1.65<br>(1.59 to 1.73)
Central Latin America	1947 to 1951	1.38<br>(1.32 to 1.44)
Central Latin America	1952 to 1956	1.17<br>(1.12 to 1.22)
Central Latin America	1957 to 1961	1<br>(1 to 1)
Central Latin America	1962 to 1966	0.86<br>(0.81 to 0.91)
Central Sub-Saharan Africa	1897 to 1901	1.46<br>(1.19 to 1.79)

Central Sub-Saharan Africa	1902 to 1906	1.46<br>(1.34 to 1.6)
Central Sub-Saharan Africa	1907 to 1911	1.48<br>(1.41 to 1.55)
Central Sub-Saharan Africa	1912 to 1916	1.48<br>(1.44 to 1.51)
Central Sub-Saharan Africa	1917 to 1921	1.45<br>(1.43 to 1.48)
Central Sub-Saharan Africa	1922 to 1926	1.42<br>(1.41 to 1.44)
Central Sub-Saharan Africa	1927 to 1931	1.38<br>(1.37 to 1.39)
Central Sub-Saharan Africa	1932 to 1936	1.33<br>(1.32 to 1.34)
Central Sub-Saharan Africa	1937 to 1941	1.27<br>(1.26 to 1.28)
Central Sub-Saharan Africa	1942 to 1946	1.21<br>(1.2 to 1.21)
Central Sub-Saharan Africa	1947 to 1951	1.14<br>(1.13 to 1.14)
Central Sub-Saharan Africa	1952 to 1956	1.06<br>(1.06 to 1.07)
Central Sub-Saharan Africa	1957 to 1961	1<br>(1 to 1)
Central Sub-Saharan Africa	1962 to 1966	0.93<br>(0.92 to 0.94)
East Asia	1897 to 1901	4.8<br>(2.23 to 10.31)
East Asia	1902 to 1906	4.68<br>(3.61 to 6.06)
East Asia	1907 to 1911	4.35<br>(3.86 to 4.9)
East Asia	1912 to 1916	4.06<br>(3.77 to 4.37)
East Asia	1917 to 1921	3.73<br>(3.53 to 3.95)
East Asia	1922 to 1926	3.32<br>(3.16 to 3.48)
East Asia	1927 to 1931	2.82<br>(2.69 to 2.95)
East Asia	1932 to 1936	2.34<br>(2.24 to 2.45)
East Asia	1937 to 1941	1.96<br>(1.88 to 2.04)
East Asia	1942 to 1946	1.62<br>(1.56 to 1.7)
East Asia	1947 to 1951	1.31<br>(1.26 to 1.37)
East Asia	1952 to 1956	1.11<br>(1.06 to 1.16)
East Asia	1957 to 1961	1<br>(1 to 1)
East Asia	1962 to 1966	0.92<br>(0.87 to 0.98)
Eastern Europe	1897 to 1901	24.32<br>(14.8 to 39.97)
Eastern Europe	1902 to 1906	19.83<br>(16.29 to 24.12)
Eastern Europe	1907 to 1911	16.12<br>(14.61 to 17.8)
Eastern Europe	1912 to 1916	12.73<br>(11.97 to 13.54)
Eastern Europe	1917 to 1921	9.88<br>(9.42 to 10.35)
Eastern Europe	1922 to 1926	7.42<br>(7.14 to 7.71)

Eastern Europe	1927 to 1931	5.71 (5.53 to 5.9)
Eastern Europe	1932 to 1936	4.38 (4.25 to 4.52)
Eastern Europe	1937 to 1941	3.2 (3.11 to 3.29)
Eastern Europe	1942 to 1946	2.42 (2.34 to 2.49)
Eastern Europe	1947 to 1951	1.74 (1.69 to 1.8)
Eastern Europe	1952 to 1956	1.34 (1.29 to 1.38)
Eastern Europe	1957 to 1961	1 (1 to 1)
Eastern Europe	1962 to 1966	0.74 (0.71 to 0.78)
Eastern Sub-Saharan Africa	1897 to 1901	1.78 (1.52 to 2.09)
Eastern Sub-Saharan Africa	1902 to 1906	1.74 (1.64 to 1.86)
Eastern Sub-Saharan Africa	1907 to 1911	1.7 (1.65 to 1.75)
Eastern Sub-Saharan Africa	1912 to 1916	1.65 (1.62 to 1.68)
Eastern Sub-Saharan Africa	1917 to 1921	1.58 (1.57 to 1.6)
Eastern Sub-Saharan Africa	1922 to 1926	1.52 (1.51 to 1.54)
Eastern Sub-Saharan Africa	1927 to 1931	1.45 (1.43 to 1.46)
Eastern Sub-Saharan Africa	1932 to 1936	1.38 (1.37 to 1.38)
Eastern Sub-Saharan Africa	1937 to 1941	1.3 (1.3 to 1.31)
Eastern Sub-Saharan Africa	1942 to 1946	1.22 (1.21 to 1.23)
Eastern Sub-Saharan Africa	1947 to 1951	1.14 (1.14 to 1.15)
Eastern Sub-Saharan Africa	1952 to 1956	1.07 (1.07 to 1.08)
Eastern Sub-Saharan Africa	1957 to 1961	1 (1 to 1)
Eastern Sub-Saharan Africa	1962 to 1966	0.92 (0.92 to 0.93)
High-income Asia Pacific	1897 to 1901	21.73 (11.2 to 42.16)
High-income Asia Pacific	1902 to 1906	20.15 (14.51 to 27.97)
High-income Asia Pacific	1907 to 1911	18.46 (14.7 to 23.17)
High-income Asia Pacific	1912 to 1916	15.8 (13.05 to 19.13)
High-income Asia Pacific	1917 to 1921	12.42 (10.41 to 14.83)
High-income Asia Pacific	1922 to 1926	9.1 (7.69 to 10.76)
High-income Asia Pacific	1927 to 1931	6.37 (5.41 to 7.49)
High-income Asia Pacific	1932 to 1936	4.35 (3.72 to 5.1)
High-income Asia Pacific	1937 to 1941	3.05 (2.61 to 3.56)
High-income Asia Pacific	1942 to 1946	2.3 (1.97 to 2.7)
High-income Asia Pacific	1947 to 1951	1.78 (1.51 to 2.1)

High-income Asia Pacific	1952 to 1956	1.38 (1.16 to 1.64)
High-income Asia Pacific	1957 to 1961	1 (1 to 1)
High-income Asia Pacific	1962 to 1966	0.74 (0.58 to 0.96)
High-income North America	1897 to 1901	0.36 (0.26 to 0.52)
High-income North America	1902 to 1906	0.42 (0.35 to 0.5)
High-income North America	1907 to 1911	0.47 (0.42 to 0.53)
High-income North America	1912 to 1916	0.52 (0.48 to 0.57)
High-income North America	1917 to 1921	0.57 (0.53 to 0.61)
High-income North America	1922 to 1926	0.61 (0.58 to 0.65)
High-income North America	1927 to 1931	0.66 (0.63 to 0.69)
High-income North America	1932 to 1936	0.71 (0.68 to 0.74)
High-income North America	1937 to 1941	0.77 (0.74 to 0.8)
High-income North America	1942 to 1946	0.86 (0.82 to 0.89)
High-income North America	1947 to 1951	0.92 (0.89 to 0.96)
High-income North America	1952 to 1956	0.96 (0.93 to 1)
High-income North America	1957 to 1961	1 (1 to 1)
High-income North America	1962 to 1966	1.05 (1 to 1.1)
North Africa and Middle East	1897 to 1901	1.56 (1.14 to 2.14)
North Africa and Middle East	1902 to 1906	1.77 (1.53 to 2.06)
North Africa and Middle East	1907 to 1911	1.87 (1.71 to 2.03)
North Africa and Middle East	1912 to 1916	1.9 (1.8 to 2.01)
North Africa and Middle East	1917 to 1921	1.92 (1.84 to 2)
North Africa and Middle East	1922 to 1926	1.92 (1.86 to 1.98)
North Africa and Middle East	1927 to 1931	1.86 (1.81 to 1.92)
North Africa and Middle East	1932 to 1936	1.75 (1.71 to 1.8)
North Africa and Middle East	1937 to 1941	1.59 (1.56 to 1.63)
North Africa and Middle East	1942 to 1946	1.45 (1.42 to 1.49)
North Africa and Middle East	1947 to 1951	1.3 (1.27 to 1.34)
North Africa and Middle East	1952 to 1956	1.15 (1.12 to 1.18)
North Africa and Middle East	1957 to 1961	1 (1 to 1)
North Africa and Middle East	1962 to 1966	0.89 (0.86 to 0.92)
Oceania	1897 to 1901	3.14 (2.09 to 4.71)
Oceania	1902 to 1906	2.63 (2.21 to 3.14)

Oceania	1907 to 1911	2.4<br>(2.21 to 2.61)
Oceania	1912 to 1916	2.28<br>(2.17 to 2.39)
Oceania	1917 to 1921	2.13<br>(2.07 to 2.2)
Oceania	1922 to 1926	1.96<br>(1.91 to 2.01)
Oceania	1927 to 1931	1.77<br>(1.74 to 1.81)
Oceania	1932 to 1936	1.59<br>(1.56 to 1.62)
Oceania	1937 to 1941	1.44<br>(1.42 to 1.47)
Oceania	1942 to 1946	1.31<br>(1.29 to 1.34)
Oceania	1947 to 1951	1.2<br>(1.18 to 1.22)
Oceania	1952 to 1956	1.09<br>(1.07 to 1.11)
Oceania	1957 to 1961	1<br>(1 to 1)
Oceania	1962 to 1966	0.91<br>(0.89 to 0.93)
South Asia	1897 to 1901	1.57<br>(1 to 2.46)
South Asia	1902 to 1906	1.59<br>(1.33 to 1.91)
South Asia	1907 to 1911	1.6<br>(1.46 to 1.75)
South Asia	1912 to 1916	1.55<br>(1.46 to 1.65)
South Asia	1917 to 1921	1.49<br>(1.43 to 1.56)
South Asia	1922 to 1926	1.44<br>(1.39 to 1.49)
South Asia	1927 to 1931	1.39<br>(1.35 to 1.43)
South Asia	1932 to 1936	1.33<br>(1.3 to 1.37)
South Asia	1937 to 1941	1.26<br>(1.23 to 1.3)
South Asia	1942 to 1946	1.18<br>(1.16 to 1.21)
South Asia	1947 to 1951	1.11<br>(1.08 to 1.14)
South Asia	1952 to 1956	1.05<br>(1.02 to 1.08)
South Asia	1957 to 1961	1<br>(1 to 1)
South Asia	1962 to 1966	0.97<br>(0.93 to 1)
Southeast Asia	1897 to 1901	2.01<br>(1.67 to 2.41)
Southeast Asia	1902 to 1906	2.04<br>(1.88 to 2.21)
Southeast Asia	1907 to 1911	2.11<br>(2.02 to 2.2)
Southeast Asia	1912 to 1916	2.09<br>(2.04 to 2.15)
Southeast Asia	1917 to 1921	2.02<br>(1.98 to 2.06)
Southeast Asia	1922 to 1926	1.91<br>(1.88 to 1.94)
Southeast Asia	1927 to 1931	1.77<br>(1.75 to 1.8)

Southeast Asia	1932 to 1936	1.64 (1.62 to 1.66)
Southeast Asia	1937 to 1941	1.5 (1.48 to 1.52)
Southeast Asia	1942 to 1946	1.37 (1.36 to 1.39)
Southeast Asia	1947 to 1951	1.25 (1.23 to 1.26)
Southeast Asia	1952 to 1956	1.12 (1.11 to 1.14)
Southeast Asia	1957 to 1961	1 (1 to 1)
Southeast Asia	1962 to 1966	0.89 (0.88 to 0.91)
Southern Latin America	1897 to 1901	1.78 (1.6 to 1.98)
Southern Latin America	1902 to 1906	1.87 (1.79 to 1.96)
Southern Latin America	1907 to 1911	1.93 (1.88 to 1.98)
Southern Latin America	1912 to 1916	1.94 (1.9 to 1.98)
Southern Latin America	1917 to 1921	1.91 (1.88 to 1.94)
Southern Latin America	1922 to 1926	1.85 (1.82 to 1.87)
Southern Latin America	1927 to 1931	1.76 (1.74 to 1.78)
Southern Latin America	1932 to 1936	1.64 (1.63 to 1.66)
Southern Latin America	1937 to 1941	1.5 (1.48 to 1.51)
Southern Latin America	1942 to 1946	1.36 (1.34 to 1.37)
Southern Latin America	1947 to 1951	1.22 (1.21 to 1.23)
Southern Latin America	1952 to 1956	1.1 (1.09 to 1.12)
Southern Latin America	1957 to 1961	1 (1 to 1)
Southern Latin America	1962 to 1966	0.91 (0.9 to 0.92)
Southern Sub-Saharan Africa	1897 to 1901	2.39 (1.95 to 2.93)
Southern Sub-Saharan Africa	1902 to 1906	2.2 (2.01 to 2.4)
Southern Sub-Saharan Africa	1907 to 1911	2.08 (1.97 to 2.19)
Southern Sub-Saharan Africa	1912 to 1916	1.98 (1.9 to 2.05)
Southern Sub-Saharan Africa	1917 to 1921	1.88 (1.83 to 1.93)
Southern Sub-Saharan Africa	1922 to 1926	1.77 (1.73 to 1.81)
Southern Sub-Saharan Africa	1927 to 1931	1.64 (1.6 to 1.67)
Southern Sub-Saharan Africa	1932 to 1936	1.51 (1.48 to 1.53)
Southern Sub-Saharan Africa	1937 to 1941	1.4 (1.38 to 1.42)
Southern Sub-Saharan Africa	1942 to 1946	1.31 (1.29 to 1.33)
Southern Sub-Saharan Africa	1947 to 1951	1.21 (1.19 to 1.23)
Southern Sub-Saharan Africa	1952 to 1956	1.11 (1.09 to 1.13)

Southern Sub-Saharan Africa	1957 to 1961	1<br>(1 to 1)
Southern Sub-Saharan Africa	1962 to 1966	0.92<br>(0.9 to 0.94)
Tropical Latin America	1897 to 1901	4.41<br>(2.57 to 7.57)
Tropical Latin America	1902 to 1906	3.98<br>(3.13 to 5.07)
Tropical Latin America	1907 to 1911	3.68<br>(3.21 to 4.23)
Tropical Latin America	1912 to 1916	3.49<br>(3.17 to 3.84)
Tropical Latin America	1917 to 1921	3.27<br>(3.04 to 3.52)
Tropical Latin America	1922 to 1926	2.99<br>(2.81 to 3.17)
Tropical Latin America	1927 to 1931	2.65<br>(2.51 to 2.8)
Tropical Latin America	1932 to 1936	2.29<br>(2.18 to 2.41)
Tropical Latin America	1937 to 1941	1.93<br>(1.84 to 2.01)
Tropical Latin America	1942 to 1946	1.62<br>(1.55 to 1.7)
Tropical Latin America	1947 to 1951	1.37<br>(1.31 to 1.43)
Tropical Latin America	1952 to 1956	1.16<br>(1.11 to 1.22)
Tropical Latin America	1957 to 1961	1<br>(1 to 1)
Tropical Latin America	1962 to 1966	0.86<br>(0.8 to 0.91)
Western Europe	1897 to 1901	4.58<br>(2.77 to 7.57)
Western Europe	1902 to 1906	4.69<br>(3.76 to 5.85)
Western Europe	1907 to 1911	4.66<br>(4.09 to 5.3)
Western Europe	1912 to 1916	4.44<br>(4.05 to 4.87)
Western Europe	1917 to 1921	3.99<br>(3.69 to 4.31)
Western Europe	1922 to 1926	3.37<br>(3.15 to 3.6)
Western Europe	1927 to 1931	2.82<br>(2.65 to 3)
Western Europe	1932 to 1936	2.3<br>(2.17 to 2.44)
Western Europe	1937 to 1941	1.85<br>(1.75 to 1.95)
Western Europe	1942 to 1946	1.57<br>(1.48 to 1.66)
Western Europe	1947 to 1951	1.37<br>(1.29 to 1.45)
Western Europe	1952 to 1956	1.17<br>(1.1 to 1.25)
Western Europe	1957 to 1961	1<br>(1 to 1)
Western Europe	1962 to 1966	0.87<br>(0.8 to 0.94)
Western Sub-Saharan Africa	1897 to 1901	1.37<br>(1.19 to 1.58)
Western Sub-Saharan Africa	1902 to 1906	1.4<br>(1.31 to 1.49)
Western Sub-Saharan Africa	1907 to 1911	1.42<br>(1.38 to 1.47)

Western Sub-Saharan Africa	1912 to 1916	1.44<br>(1.41 to 1.47)
Western Sub-Saharan Africa	1917 to 1921	1.44<br>(1.42 to 1.46)
Western Sub-Saharan Africa	1922 to 1926	1.41<br>(1.4 to 1.43)
Western Sub-Saharan Africa	1927 to 1931	1.32<br>(1.31 to 1.34)
Western Sub-Saharan Africa	1932 to 1936	1.24<br>(1.23 to 1.25)
Western Sub-Saharan Africa	1937 to 1941	1.18<br>(1.17 to 1.19)
Western Sub-Saharan Africa	1942 to 1946	1.14<br>(1.13 to 1.15)
Western Sub-Saharan Africa	1947 to 1951	1.07<br>(1.06 to 1.08)
Western Sub-Saharan Africa	1952 to 1956	1.04<br>(1.03 to 1.04)
Western Sub-Saharan Africa	1957 to 1961	1<br>(1 to 1)
Western Sub-Saharan Africa	1962 to 1966	0.95<br>(0.94 to 0.96)

Table S10 APC Model Analysis Results of ILD&PS Prevalence Among Adults Aged 55 and Above in the Global, 5 SDI Regions, and 21 GBD Regions

ILD&PS<br>Location	Age	Local drift (%/year)
Global	55 to 59	-0.08<br>(-0.16 to 0.01)
Global	60 to 64	0.19<br>(0.12 to 0.25)
Global	65 to 69	0.47<br>(0.41 to 0.53)
Global	70 to 74	0.75<br>(0.69 to 0.82)
Global	75 to 79	0.91<br>(0.84 to 0.98)
Global	80 to 84	1.19<br>(1.09 to 1.28)
Global	85 to 89	1.62<br>(1.47 to 1.77)
Global	90 to 94	1.92<br>(1.62 to 2.22)
Global	95+	1.95<br>(1.23 to 2.68)
High SDI	55 to 59	-0.05<br>(-0.18 to 0.08)
High SDI	60 to 64	0.21<br>(0.12 to 0.31)
High SDI	65 to 69	0.57<br>(0.49 to 0.66)
High SDI	70 to 74	0.97<br>(0.89 to 1.06)
High SDI	75 to 79	1.17<br>(1.08 to 1.26)
High SDI	80 to 84	1.52<br>(1.41 to 1.64)
High SDI	85 to 89	1.9<br>(1.72 to 2.08)
High SDI	90 to 94	2.06<br>(1.72 to 2.4)
High SDI	95+	1.96<br>(1.15 to 2.77)
High-middle SDI	55 to 59	0.05<br>(-0.09 to 0.19)
High-middle SDI	60 to 64	0.3<br>(0.2 to 0.41)
High-middle SDI	65 to 69	0.75<br>(0.65 to 0.84)
High-middle SDI	70 to 74	1.18<br>(1.08 to 1.29)
High-middle SDI	75 to 79	1.54<br>(1.41 to 1.68)
High-middle SDI	80 to 84	1.95<br>(1.77 to 2.14)
High-middle SDI	85 to 89	2.5<br>(2.18 to 2.82)
High-middle SDI	90 to 94	2.83<br>(2.16 to 3.51)
High-middle SDI	95+	3.02<br>(1.2 to 4.88)
Middle SDI	55 to 59	0.27<br>(0.21 to 0.34)
Middle SDI	60 to 64	0.49<br>(0.44 to 0.54)
Middle SDI	65 to 69	0.8<br>(0.75 to 0.85)
Middle SDI	70 to 74	1.18<br>(1.12 to 1.24)

Middle SDI	75 to 79	1.51<br>(1.44 to 1.58)
Middle SDI	80 to 84	1.79<br>(1.68 to 1.89)
Middle SDI	85 to 89	2.09<br>(1.91 to 2.26)
Middle SDI	90 to 94	2.38<br>(2.02 to 2.75)
Middle SDI	95+	2.68<br>(1.78 to 3.59)
Low-middle SDI	55 to 59	0.12<br>(0.08 to 0.16)
Low-middle SDI	60 to 64	0.26<br>(0.23 to 0.29)
Low-middle SDI	65 to 69	0.45<br>(0.42 to 0.48)
Low-middle SDI	70 to 74	0.62<br>(0.59 to 0.66)
Low-middle SDI	75 to 79	0.78<br>(0.74 to 0.82)
Low-middle SDI	80 to 84	0.91<br>(0.85 to 0.97)
Low-middle SDI	85 to 89	1.04<br>(0.93 to 1.14)
Low-middle SDI	90 to 94	1.25<br>(1.03 to 1.47)
Low-middle SDI	95+	1.51<br>(0.95 to 2.06)
Low SDI	55 to 59	-0.09<br>(-0.16 to -0.02)
Low SDI	60 to 64	0.1<br>(0.05 to 0.16)
Low SDI	65 to 69	0.32<br>(0.27 to 0.38)
Low SDI	70 to 74	0.43<br>(0.36 to 0.49)
Low SDI	75 to 79	0.44<br>(0.36 to 0.53)
Low SDI	80 to 84	0.41<br>(0.28 to 0.55)
Low SDI	85 to 89	0.33<br>(0.07 to 0.59)
Low SDI	90 to 94	0.47<br>(-0.14 to 1.09)
Low SDI	95+	0.73<br>(-0.88 to 2.35)
Andean Latin America	55 to 59	1.65<br>(1.53 to 1.78)
Andean Latin America	60 to 64	1.88<br>(1.78 to 1.97)
Andean Latin America	65 to 69	2.18<br>(2.09 to 2.26)
Andean Latin America	70 to 74	2.53<br>(2.44 to 2.61)
Andean Latin America	75 to 79	2.96<br>(2.86 to 3.06)
Andean Latin America	80 to 84	3.44<br>(3.32 to 3.57)
Andean Latin America	85 to 89	3.91<br>(3.73 to 4.1)
Andean Latin America	90 to 94	4.26<br>(3.95 to 4.57)
Andean Latin America	95+	4.57<br>(3.91 to 5.23)
Australasia	55 to 59	0.83<br>(0.64 to 1.02)

Australasia	60 to 64	1.09<br>(0.95 to 1.23)
Australasia	65 to 69	1.47<br>(1.35 to 1.59)
Australasia	70 to 74	1.92<br>(1.81 to 2.03)
Australasia	75 to 79	2.4<br>(2.28 to 2.52)
Australasia	80 to 84	2.83<br>(2.68 to 2.99)
Australasia	85 to 89	3.16<br>(2.9 to 3.41)
Australasia	90 to 94	3.29<br>(2.78 to 3.8)
Australasia	95+	3.19<br>(1.9 to 4.51)
Caribbean	55 to 59	0.85<br>(0.58 to 1.12)
Caribbean	60 to 64	1.07<br>(0.85 to 1.28)
Caribbean	65 to 69	1.25<br>(1.04 to 1.46)
Caribbean	70 to 74	1.43<br>(1.21 to 1.66)
Caribbean	75 to 79	1.62<br>(1.35 to 1.89)
Caribbean	80 to 84	1.83<br>(1.46 to 2.2)
Caribbean	85 to 89	2.1<br>(1.54 to 2.67)
Caribbean	90 to 94	2.38<br>(1.29 to 3.47)
Caribbean	95+	2.54<br>(-0.18 to 5.33)
Central Asia	55 to 59	-0.18<br>(-0.33 to -0.03)
Central Asia	60 to 64	0<br>(-0.12 to 0.12)
Central Asia	65 to 69	0.31<br>(0.19 to 0.43)
Central Asia	70 to 74	0.7<br>(0.55 to 0.84)
Central Asia	75 to 79	1.06<br>(0.88 to 1.24)
Central Asia	80 to 84	1.3<br>(1.06 to 1.53)
Central Asia	85 to 89	1.29<br>(0.95 to 1.64)
Central Asia	90 to 94	1.03<br>(0.42 to 1.64)
Central Asia	95+	0.84<br>(-0.63 to 2.33)
Central Europe	55 to 59	0.03<br>(-0.07 to 0.13)
Central Europe	60 to 64	0.24<br>(0.16 to 0.31)
Central Europe	65 to 69	0.39<br>(0.32 to 0.46)
Central Europe	70 to 74	0.55<br>(0.47 to 0.63)
Central Europe	75 to 79	0.65<br>(0.54 to 0.76)
Central Europe	80 to 84	0.72<br>(0.57 to 0.87)
Central Europe	85 to 89	0.97<br>(0.71 to 1.24)

Central Europe	90 to 94	1.35<br>(0.73 to 1.98)
Central Europe	95+	1.68<br>(-0.13 to 3.52)
Central Latin America	55 to 59	0.08<br>(-0.06 to 0.22)
Central Latin America	60 to 64	0.13<br>(0.01 to 0.24)
Central Latin America	65 to 69	0.27<br>(0.16 to 0.37)
Central Latin America	70 to 74	0.49<br>(0.38 to 0.6)
Central Latin America	75 to 79	0.74<br>(0.61 to 0.88)
Central Latin America	80 to 84	1.06<br>(0.88 to 1.24)
Central Latin America	85 to 89	1.53<br>(1.24 to 1.81)
Central Latin America	90 to 94	2.16<br>(1.57 to 2.75)
Central Latin America	95+	2.74<br>(1.21 to 4.3)
Central Sub-Saharan Africa	55 to 59	0.58<br>(0.31 to 0.85)
Central Sub-Saharan Africa	60 to 64	0.57<br>(0.35 to 0.78)
Central Sub-Saharan Africa	65 to 69	0.52<br>(0.3 to 0.75)
Central Sub-Saharan Africa	70 to 74	0.47<br>(0.19 to 0.74)
Central Sub-Saharan Africa	75 to 79	0.4<br>(0.03 to 0.78)
Central Sub-Saharan Africa	80 to 84	0.38<br>(-0.24 to 1.01)
Central Sub-Saharan Africa	85 to 89	0.38<br>(-0.94 to 1.73)
Central Sub-Saharan Africa	90 to 94	0.48<br>(-2.56 to 3.61)
Central Sub-Saharan Africa	95+	-0.29<br>(-6.42 to 6.24)
East Asia	55 to 59	0.36<br>(0.16 to 0.56)
East Asia	60 to 64	0.75<br>(0.59 to 0.91)
East Asia	65 to 69	1.22<br>(1.07 to 1.37)
East Asia	70 to 74	1.79<br>(1.62 to 1.97)
East Asia	75 to 79	2.33<br>(2.1 to 2.57)
East Asia	80 to 84	2.79<br>(2.42 to 3.15)
East Asia	85 to 89	3.1<br>(2.39 to 3.82)
East Asia	90 to 94	3.18<br>(1.43 to 4.96)
East Asia	95+	3.1<br>(-2.55 to 9.07)
Eastern Europe	55 to 59	-2.85<br>(-2.94 to -2.76)
Eastern Europe	60 to 64	-2.92<br>(-2.99 to -2.85)
Eastern Europe	65 to 69	-2.97<br>(-3.04 to -2.9)
Eastern Europe	70 to 74	-3.03<br>(-3.12 to -2.95)

Eastern Europe	75 to 79	-3.03<br>(-3.14 to -2.92)
Eastern Europe	80 to 84	-3.11<br>(-3.25 to -2.97)
Eastern Europe	85 to 89	-3.14<br>(-3.38 to -2.9)
Eastern Europe	90 to 94	-3.05<br>(-3.56 to -2.53)
Eastern Europe	95+	-2.92<br>(-4.29 to -1.54)
Eastern Sub-Saharan Africa	55 to 59	0.42<br>(0.25 to 0.59)
Eastern Sub-Saharan Africa	60 to 64	0.44<br>(0.31 to 0.58)
Eastern Sub-Saharan Africa	65 to 69	0.45<br>(0.31 to 0.59)
Eastern Sub-Saharan Africa	70 to 74	0.42<br>(0.26 to 0.59)
Eastern Sub-Saharan Africa	75 to 79	0.38<br>(0.16 to 0.6)
Eastern Sub-Saharan Africa	80 to 84	0.33<br>(-0.02 to 0.69)
Eastern Sub-Saharan Africa	85 to 89	0.21<br>(-0.49 to 0.92)
Eastern Sub-Saharan Africa	90 to 94	0.03<br>(-1.6 to 1.68)
Eastern Sub-Saharan Africa	95+	-0.47<br>(-4.67 to 3.92)
High-income Asia Pacific	55 to 59	-0.15<br>(-0.29 to 0)
High-income Asia Pacific	60 to 64	0.03<br>(-0.08 to 0.13)
High-income Asia Pacific	65 to 69	0.27<br>(0.18 to 0.35)
High-income Asia Pacific	70 to 74	0.57<br>(0.49 to 0.66)
High-income Asia Pacific	75 to 79	0.88<br>(0.79 to 0.98)
High-income Asia Pacific	80 to 84	1.17<br>(1.05 to 1.29)
High-income Asia Pacific	85 to 89	1.37<br>(1.17 to 1.58)
High-income Asia Pacific	90 to 94	1.41<br>(0.99 to 1.82)
High-income Asia Pacific	95+	1.27<br>(0.15 to 2.4)
High-income North America	55 to 59	-0.27<br>(-0.42 to -0.13)
High-income North America	60 to 64	-0.08<br>(-0.19 to 0.03)
High-income North America	65 to 69	0.13<br>(0.04 to 0.23)
High-income North America	70 to 74	0.4<br>(0.31 to 0.49)
High-income North America	75 to 79	0.74<br>(0.64 to 0.84)
High-income North America	80 to 84	1.16<br>(1.03 to 1.28)
High-income North America	85 to 89	1.57<br>(1.39 to 1.75)
High-income North America	90 to 94	1.96<br>(1.64 to 2.27)
High-income North America	95+	2.26<br>(1.56 to 2.97)
North Africa and Middle East	55 to 59	1.48<br>(1.4 to 1.55)

North Africa and Middle East	60 to 64	1.55 (1.49 to 1.61)
North Africa and Middle East	65 to 69	1.57 (1.51 to 1.63)
North Africa and Middle East	70 to 74	1.62 (1.54 to 1.69)
North Africa and Middle East	75 to 79	1.6 (1.5 to 1.7)
North Africa and Middle East	80 to 84	1.57 (1.42 to 1.72)
North Africa and Middle East	85 to 89	1.67 (1.42 to 1.92)
North Africa and Middle East	90 to 94	1.84 (1.35 to 2.33)
North Africa and Middle East	95+	1.91 (0.81 to 3.01)
Oceania	55 to 59	0.35 (-0.14 to 0.84)
Oceania	60 to 64	0.42 (0.01 to 0.82)
Oceania	65 to 69	0.4 (-0.02 to 0.81)
Oceania	70 to 74	0.39 (-0.11 to 0.9)
Oceania	75 to 79	0.46 (-0.25 to 1.18)
Oceania	80 to 84	0.7 (-0.49 to 1.9)
Oceania	85 to 89	0.91 (-1.67 to 3.55)
Oceania	90 to 94	0.91 (-6.05 to 8.38)
Oceania	95+	0.15 (-17.22 to 21.16)
South Asia	55 to 59	0.1 (0.06 to 0.13)
South Asia	60 to 64	0.19 (0.16 to 0.22)
South Asia	65 to 69	0.26 (0.24 to 0.29)
South Asia	70 to 74	0.34 (0.3 to 0.37)
South Asia	75 to 79	0.43 (0.38 to 0.47)
South Asia	80 to 84	0.56 (0.5 to 0.63)
South Asia	85 to 89	0.77 (0.65 to 0.89)
South Asia	90 to 94	1.01 (0.75 to 1.28)
South Asia	95+	1.23 (0.52 to 1.94)
Southeast Asia	55 to 59	0.83 (0.75 to 0.91)
Southeast Asia	60 to 64	0.89 (0.82 to 0.95)
Southeast Asia	65 to 69	0.93 (0.87 to 0.99)
Southeast Asia	70 to 74	0.99 (0.92 to 1.07)
Southeast Asia	75 to 79	1.11 (1.01 to 1.21)
Southeast Asia	80 to 84	1.33 (1.17 to 1.48)
Southeast Asia	85 to 89	1.63 (1.37 to 1.89)

Southeast Asia	90 to 94	1.85<br>(1.33 to 2.38)
Southeast Asia	95+	1.85<br>(0.64 to 3.07)
Southern Latin America	55 to 59	1.41<br>(1.31 to 1.51)
Southern Latin America	60 to 64	1.51<br>(1.43 to 1.58)
Southern Latin America	65 to 69	1.65<br>(1.58 to 1.72)
Southern Latin America	70 to 74	1.85<br>(1.78 to 1.92)
Southern Latin America	75 to 79	2.09<br>(2.01 to 2.17)
Southern Latin America	80 to 84	2.34<br>(2.23 to 2.45)
Southern Latin America	85 to 89	2.64<br>(2.46 to 2.81)
Southern Latin America	90 to 94	2.84<br>(2.51 to 3.18)
Southern Latin America	95+	2.87<br>(2.03 to 3.72)
Southern Sub-Saharan Africa	55 to 59	-0.59<br>(-0.75 to -0.43)
Southern Sub-Saharan Africa	60 to 64	-0.5<br>(-0.63 to -0.38)
Southern Sub-Saharan Africa	65 to 69	-0.43<br>(-0.55 to -0.31)
Southern Sub-Saharan Africa	70 to 74	-0.39<br>(-0.53 to -0.26)
Southern Sub-Saharan Africa	75 to 79	-0.38<br>(-0.54 to -0.21)
Southern Sub-Saharan Africa	80 to 84	-0.43<br>(-0.67 to -0.19)
Southern Sub-Saharan Africa	85 to 89	-0.58<br>(-0.96 to -0.19)
Southern Sub-Saharan Africa	90 to 94	-0.71<br>(-1.45 to 0.03)
Southern Sub-Saharan Africa	95+	-0.85<br>(-2.67 to 1)
Tropical Latin America	55 to 59	-1.36<br>(-1.49 to -1.23)
Tropical Latin America	60 to 64	-1.23<br>(-1.33 to -1.13)
Tropical Latin America	65 to 69	-0.97<br>(-1.07 to -0.88)
Tropical Latin America	70 to 74	-0.6<br>(-0.7 to -0.49)
Tropical Latin America	75 to 79	-0.17<br>(-0.29 to -0.05)
Tropical Latin America	80 to 84	0.24<br>(0.08 to 0.4)
Tropical Latin America	85 to 89	0.59<br>(0.34 to 0.84)
Tropical Latin America	90 to 94	0.96<br>(0.47 to 1.45)
Tropical Latin America	95+	1.45<br>(0.18 to 2.74)
Western Europe	55 to 59	0.42<br>(0.38 to 0.47)
Western Europe	60 to 64	0.68<br>(0.64 to 0.71)
Western Europe	65 to 69	1.01<br>(0.98 to 1.04)
Western Europe	70 to 74	1.43<br>(1.4 to 1.46)

Western Europe	75 to 79	1.71<br>(1.68 to 1.75)
Western Europe	80 to 84	2.12<br>(2.07 to 2.16)
Western Europe	85 to 89	2.47<br>(2.41 to 2.54)
Western Europe	90 to 94	2.6<br>(2.46 to 2.74)
Western Europe	95+	2.5<br>(2.14 to 2.87)
Western Sub-Saharan Africa	55 to 59	-0.35<br>(-0.5 to -0.19)
Western Sub-Saharan Africa	60 to 64	-0.43<br>(-0.56 to -0.3)
Western Sub-Saharan Africa	65 to 69	-0.5<br>(-0.63 to -0.37)
Western Sub-Saharan Africa	70 to 74	-0.47<br>(-0.62 to -0.31)
Western Sub-Saharan Africa	75 to 79	-0.39<br>(-0.6 to -0.18)
Western Sub-Saharan Africa	80 to 84	-0.2<br>(-0.54 to 0.14)
Western Sub-Saharan Africa	85 to 89	0.1<br>(-0.54 to 0.76)
Western Sub-Saharan Africa	90 to 94	0.39<br>(-1.01 to 1.81)
Western Sub-Saharan Africa	95+	0.91<br>(-2.59 to 4.54)

ILD&PS<br>Location	Age	Prevalence rate (per 100,000 population)
Global	55 to 59	114.31<br>(112.57 to 116.08)
Global	60 to 64	163.26<br>(160.87 to 165.68)
Global	65 to 69	224.53<br>(220.59 to 228.54)
Global	70 to 74	294.84<br>(289.49 to 300.28)
Global	75 to 79	365.96<br>(359.02 to 373.03)
Global	80 to 84	419.44<br>(410.84 to 428.21)
Global	85 to 89	440.96<br>(430.35 to 451.83)
Global	90 to 94	438.54<br>(423.88 to 453.7)
Global	95+	419.16<br>(394.75 to 445.07)
High SDI	55 to 59	209.91<br>(204.85 to 215.1)
High SDI	60 to 64	305.71<br>(298.69 to 312.89)
High SDI	65 to 69	437.22<br>(425.42 to 449.35)
High SDI	70 to 74	592.45<br>(576.12 to 609.24)
High SDI	75 to 79	739.61<br>(718.61 to 761.24)
High SDI	80 to 84	824.06<br>(799.41 to 849.46)
High SDI	85 to 89	819.33<br>(792.04 to 847.57)
High SDI	90 to 94	755.4<br>(723.22 to 789.01)

High SDI	95+	660.52 (614.85 to 709.58)
High-middle SDI	55 to 59	91.95 (89.69 to 94.27)
High-middle SDI	60 to 64	126.32 (123.3 to 129.41)
High-middle SDI	65 to 69	165.43 (160.69 to 170.31)
High-middle SDI	70 to 74	205.5 (199.4 to 211.79)
High-middle SDI	75 to 79	241.61 (234.07 to 249.39)
High-middle SDI	80 to 84	264.98 (255.9 to 274.38)
High-middle SDI	85 to 89	268.31 (257.07 to 280.05)
High-middle SDI	90 to 94	257.21 (240.84 to 274.69)
High-middle SDI	95+	249.57 (218.58 to 284.95)
Middle SDI	55 to 59	82.4 (81.48 to 83.34)
Middle SDI	60 to 64	115.73 (114.48 to 117)
Middle SDI	65 to 69	152.05 (150.01 to 154.11)
Middle SDI	70 to 74	188.1 (185.45 to 190.79)
Middle SDI	75 to 79	220.81 (217.48 to 224.2)
Middle SDI	80 to 84	241.18 (237.06 to 245.38)
Middle SDI	85 to 89	248.52 (243.07 to 254.1)
Middle SDI	90 to 94	265.67 (256.67 to 274.99)
Middle SDI	95+	306.82 (288.39 to 326.44)
Low-middle SDI	55 to 59	102.24 (101.6 to 102.88)
Low-middle SDI	60 to 64	145.53 (144.66 to 146.4)
Low-middle SDI	65 to 69	189.57 (188.18 to 190.96)
Low-middle SDI	70 to 74	227.72 (225.96 to 229.48)
Low-middle SDI	75 to 79	249.74 (247.65 to 251.84)
Low-middle SDI	80 to 84	237.87 (235.53 to 240.23)
Low-middle SDI	85 to 89	190.82 (188.16 to 193.53)
Low-middle SDI	90 to 94	161.66 (157.75 to 165.66)
Low-middle SDI	95+	155.53 (148.55 to 162.84)
Low SDI	55 to 59	69.75 (68.87 to 70.65)
Low SDI	60 to 64	96.84 (95.65 to 98.04)
Low SDI	65 to 69	122.08 (120.25 to 123.93)
Low SDI	70 to 74	140.15 (137.92 to 142.41)
Low SDI	75 to 79	146.79 (144.22 to 149.39)

Low SDI	80 to 84	132.9<br>(130.06 to 135.8)
Low SDI	85 to 89	95.53<br>(92.33 to 98.85)
Low SDI	90 to 94	73.22<br>(68.42 to 78.36)
Low SDI	95+	64.45<br>(55.36 to 75.04)
Andean Latin America	55 to 59	226.73<br>(222 to 231.55)
Andean Latin America	60 to 64	392.98<br>(385.42 to 400.69)
Andean Latin America	65 to 69	650.09<br>(634.51 to 666.06)
Andean Latin America	70 to 74	1053.86<br>(1027.61 to 1080.78)
Andean Latin America	75 to 79	1574.26<br>(1533.26 to 1616.36)
Andean Latin America	80 to 84	2112.59<br>(2053.97 to 2172.88)
Andean Latin America	85 to 89	2781.26<br>(2696.12 to 2869.08)
Andean Latin America	90 to 94	3625.24<br>(3492.4 to 3763.12)
Andean Latin America	95+	4772.14<br>(4536.59 to 5019.92)
Australasia	55 to 59	106.61<br>(103.04 to 110.31)
Australasia	60 to 64	183.39<br>(177.75 to 189.2)
Australasia	65 to 69	307.25<br>(295.95 to 318.98)
Australasia	70 to 74	489.6<br>(471.18 to 508.74)
Australasia	75 to 79	701.17<br>(674.01 to 729.42)
Australasia	80 to 84	846.82<br>(812.58 to 882.51)
Australasia	85 to 89	857.75<br>(819.86 to 897.4)
Australasia	90 to 94	772.09<br>(729.57 to 817.09)
Australasia	95+	673.61<br>(611.34 to 742.22)
Caribbean	55 to 59	52.55<br>(50.13 to 55.09)
Caribbean	60 to 64	70.53<br>(67.34 to 73.88)
Caribbean	65 to 69	90.8<br>(85.8 to 96.09)
Caribbean	70 to 74	113.38<br>(106.84 to 120.32)
Caribbean	75 to 79	126.16<br>(118.36 to 134.46)
Caribbean	80 to 84	127.77<br>(118.96 to 137.22)
Caribbean	85 to 89	123.61<br>(113.32 to 134.84)
Caribbean	90 to 94	122.54<br>(108.61 to 138.24)
Caribbean	95+	134.81<br>(113.12 to 160.67)
Central Asia	55 to 59	78.87<br>(76.66 to 81.14)
Central Asia	60 to 64	99.16<br>(96.47 to 101.91)

Central Asia	65 to 69	122.98<br>(118.82 to 127.27)
Central Asia	70 to 74	150.7<br>(145.4 to 156.2)
Central Asia	75 to 79	170.26<br>(163.87 to 176.89)
Central Asia	80 to 84	169.73<br>(162.4 to 177.4)
Central Asia	85 to 89	158.42<br>(149.32 to 168.07)
Central Asia	90 to 94	149.12<br>(135.8 to 163.75)
Central Asia	95+	140.37<br>(117.87 to 167.17)
Central Europe	55 to 59	100.21<br>(98.44 to 102.01)
Central Europe	60 to 64	119.28<br>(117.18 to 121.42)
Central Europe	65 to 69	136.4<br>(133.55 to 139.32)
Central Europe	70 to 74	146.91<br>(143.71 to 150.19)
Central Europe	75 to 79	143.72<br>(140.35 to 147.17)
Central Europe	80 to 84	126.12<br>(122.71 to 129.62)
Central Europe	85 to 89	98.09<br>(94.48 to 101.85)
Central Europe	90 to 94	74.31<br>(69.43 to 79.54)
Central Europe	95+	60.18<br>(51.75 to 69.98)
Central Latin America	55 to 59	108.88<br>(106.21 to 111.62)
Central Latin America	60 to 64	153.17<br>(149.58 to 156.85)
Central Latin America	65 to 69	206.9<br>(200.88 to 213.09)
Central Latin America	70 to 74	269.36<br>(261.2 to 277.78)
Central Latin America	75 to 79	325.73<br>(315.31 to 336.49)
Central Latin America	80 to 84	350.34<br>(338.04 to 363.09)
Central Latin America	85 to 89	344.01<br>(329.65 to 359)
Central Latin America	90 to 94	322.55<br>(303.97 to 342.27)
Central Latin America	95+	270.25<br>(245.56 to 297.44)
Central Sub-Saharan Africa	55 to 59	47.72<br>(45.58 to 49.97)
Central Sub-Saharan Africa	60 to 64	63.52<br>(60.71 to 66.46)
Central Sub-Saharan Africa	65 to 69	79.08<br>(74.65 to 83.77)
Central Sub-Saharan Africa	70 to 74	89.78<br>(84.36 to 95.56)
Central Sub-Saharan Africa	75 to 79	93.91<br>(87.52 to 100.76)
Central Sub-Saharan Africa	80 to 84	85.24<br>(77.87 to 93.31)
Central Sub-Saharan Africa	85 to 89	64.01<br>(55.11 to 74.36)
Central Sub-Saharan Africa	90 to 94	50.94<br>(37.29 to 69.58)

Central Sub-Saharan Africa	95+	50.88 (24.74 to 104.64)
East Asia	55 to 59	80.19 (77.47 to 82.99)
East Asia	60 to 64	112.84 (109.13 to 116.68)
East Asia	65 to 69	148.97 (142.97 to 155.22)
East Asia	70 to 74	182.81 (175.04 to 190.92)
East Asia	75 to 79	215.28 (205.5 to 225.53)
East Asia	80 to 84	237.33 (225.08 to 250.25)
East Asia	85 to 89	236.56 (220.31 to 254.01)
East Asia	90 to 94	220.34 (194 to 250.27)
East Asia	95+	206.75 (152.32 to 280.65)
Eastern Europe	55 to 59	50.59 (49.67 to 51.53)
Eastern Europe	60 to 64	48.13 (47.24 to 49.03)
Eastern Europe	65 to 69	42.51 (41.61 to 43.43)
Eastern Europe	70 to 74	34.99 (34.21 to 35.8)
Eastern Europe	75 to 79	26.57 (25.91 to 27.25)
Eastern Europe	80 to 84	18.55 (17.99 to 19.13)
Eastern Europe	85 to 89	12.03 (11.5 to 12.58)
Eastern Europe	90 to 94	8.16 (7.51 to 8.87)
Eastern Europe	95+	6.18 (5.07 to 7.55)
Eastern Sub-Saharan Africa	55 to 59	38.9 (37.75 to 40.09)
Eastern Sub-Saharan Africa	60 to 64	50.97 (49.49 to 52.5)
Eastern Sub-Saharan Africa	65 to 69	62.58 (60.31 to 64.93)
Eastern Sub-Saharan Africa	70 to 74	70.36 (67.63 to 73.21)
Eastern Sub-Saharan Africa	75 to 79	73.74 (70.56 to 77.06)
Eastern Sub-Saharan Africa	80 to 84	66.19 (62.64 to 69.93)
Eastern Sub-Saharan Africa	85 to 89	44.98 (41.09 to 49.23)
Eastern Sub-Saharan Africa	90 to 94	31.12 (25.73 to 37.65)
Eastern Sub-Saharan Africa	95+	24.03 (15.32 to 37.69)
High-income Asia Pacific	55 to 59	314.58 (306.08 to 323.31)
High-income Asia Pacific	60 to 64	498.1 (485.36 to 511.18)
High-income Asia Pacific	65 to 69	750.58 (728.78 to 773.04)
High-income Asia Pacific	70 to 74	1020.26 (990.24 to 1051.19)
High-income Asia Pacific	75 to 79	1237.64 (1200.29 to 1276.17)

High-income Asia Pacific	80 to 84	1297.74 (1256.66 to 1340.15)
High-income Asia Pacific	85 to 89	1163.58 (1122.77 to 1205.87)
High-income Asia Pacific	90 to 94	918.11 (876.81 to 961.35)
High-income Asia Pacific	95+	663.65 (615.36 to 715.74)
High-income North America	55 to 59	262.95 (256.07 to 270.01)
High-income North America	60 to 64	353.76 (344.97 to 362.78)
High-income North America	65 to 69	481.73 (467.27 to 496.65)
High-income North America	70 to 74	652.28 (632.18 to 673.02)
High-income North America	75 to 79	849.39 (822.47 to 877.19)
High-income North America	80 to 84	1006.16 (972.84 to 1040.62)
High-income North America	85 to 89	1069.95 (1031.17 to 1110.18)
High-income North America	90 to 94	1048.35 (1001.76 to 1097.11)
High-income North America	95+	982.04 (914.26 to 1054.85)
North Africa and Middle East	55 to 59	95.53 (94.41 to 96.67)
North Africa and Middle East	60 to 64	130.09 (128.58 to 131.61)
North Africa and Middle East	65 to 69	167.47 (164.99 to 169.99)
North Africa and Middle East	70 to 74	198.27 (195.1 to 201.49)
North Africa and Middle East	75 to 79	210.28 (206.54 to 214.09)
North Africa and Middle East	80 to 84	205.82 (201.45 to 210.29)
North Africa and Middle East	85 to 89	185.97 (180.44 to 191.68)
North Africa and Middle East	90 to 94	173.79 (164.94 to 183.12)
North Africa and Middle East	95+	180.55 (164.93 to 197.66)
Oceania	55 to 59	107.48 (98.83 to 116.89)
Oceania	60 to 64	143.37 (132.01 to 155.71)
Oceania	65 to 69	176.92 (159.21 to 196.59)
Oceania	70 to 74	187.45 (167 to 210.4)
Oceania	75 to 79	187.33 (164.23 to 213.69)
Oceania	80 to 84	186.4 (158.33 to 219.45)
Oceania	85 to 89	122.56 (93.06 to 161.42)
Oceania	90 to 94	67.36 (34.7 to 130.76)
Oceania	95+	46.06 (7.55 to 281.05)
South Asia	55 to 59	135.07 (134.19 to 135.94)
South Asia	60 to 64	190.82 (189.64 to 192.01)

South Asia	65 to 69	247.09 (245.21 to 248.97)
South Asia	70 to 74	295.47 (293.1 to 297.86)
South Asia	75 to 79	324.06 (321.24 to 326.9)
South Asia	80 to 84	308.59 (305.43 to 311.79)
South Asia	85 to 89	237.94 (234.39 to 241.54)
South Asia	90 to 94	187.25 (182.16 to 192.48)
South Asia	95+	168.08 (158.42 to 178.32)
Southeast Asia	55 to 59	46.96 (46.33 to 47.6)
Southeast Asia	60 to 64	67.93 (67.06 to 68.81)
Southeast Asia	65 to 69	89.55 (88.09 to 91.04)
Southeast Asia	70 to 74	104.1 (102.28 to 105.95)
Southeast Asia	75 to 79	107.82 (105.73 to 109.94)
Southeast Asia	80 to 84	100.82 (98.48 to 103.21)
Southeast Asia	85 to 89	87.42 (84.56 to 90.37)
Southeast Asia	90 to 94	80.27 (75.85 to 84.95)
Southeast Asia	95+	80.83 (72.63 to 89.97)
Southern Latin America	55 to 59	222.21 (218.31 to 226.18)
Southern Latin America	60 to 64	362.52 (356.6 to 368.54)
Southern Latin America	65 to 69	539.45 (528.72 to 550.4)
Southern Latin America	70 to 74	753.29 (737.82 to 769.09)
Southern Latin America	75 to 79	952.28 (931.84 to 973.17)
Southern Latin America	80 to 84	1047.2 (1022.98 to 1071.99)
Southern Latin America	85 to 89	1033.94 (1006.23 to 1062.42)
Southern Latin America	90 to 94	999.45 (962.83 to 1037.47)
Southern Latin America	95+	948.07 (889.57 to 1010.42)
Southern Sub-Saharan Africa	55 to 59	117.1 (113.82 to 120.48)
Southern Sub-Saharan Africa	60 to 64	154.73 (150.47 to 159.11)
Southern Sub-Saharan Africa	65 to 69	191.7 (185.33 to 198.29)
Southern Sub-Saharan Africa	70 to 74	217.7 (210.09 to 225.58)
Southern Sub-Saharan Africa	75 to 79	222.56 (214.11 to 231.34)
Southern Sub-Saharan Africa	80 to 84	207.07 (198.05 to 216.5)
Southern Sub-Saharan Africa	85 to 89	164.85 (155.06 to 175.26)
Southern Sub-Saharan Africa	90 to 94	129.51 (115.74 to 144.92)

Southern Sub-Saharan Africa	95+	107.4<br>(81.71 to 141.16)
Tropical Latin America	55 to 59	48.41<br>(47.23 to 49.62)
Tropical Latin America	60 to 64	62.44<br>(60.99 to 63.93)
Tropical Latin America	65 to 69	76.23<br>(74.07 to 78.45)
Tropical Latin America	70 to 74	90.78<br>(88.11 to 93.53)
Tropical Latin America	75 to 79	104.93<br>(101.7 to 108.28)
Tropical Latin America	80 to 84	113.87<br>(110.04 to 117.82)
Tropical Latin America	85 to 89	117.69<br>(113.06 to 122.5)
Tropical Latin America	90 to 94	117.57<br>(111.39 to 124.08)
Tropical Latin America	95+	112.05<br>(103.11 to 121.76)
Western Europe	55 to 59	140.57<br>(139.33 to 141.82)
Western Europe	60 to 64	205.32<br>(203.58 to 207.08)
Western Europe	65 to 69	290.05<br>(287.18 to 292.95)
Western Europe	70 to 74	388.21<br>(384.3 to 392.16)
Western Europe	75 to 79	469.54<br>(464.67 to 474.47)
Western Europe	80 to 84	502.71<br>(497.21 to 508.28)
Western Europe	85 to 89	470.26<br>(464.44 to 476.15)
Western Europe	90 to 94	405.97<br>(399.22 to 412.83)
Western Europe	95+	348.87<br>(338.55 to 359.51)
Western Sub-Saharan Africa	55 to 59	36.17<br>(35.15 to 37.23)
Western Sub-Saharan Africa	60 to 64	44.24<br>(42.99 to 45.52)
Western Sub-Saharan Africa	65 to 69	50.79<br>(49.03 to 52.62)
Western Sub-Saharan Africa	70 to 74	53.11<br>(51.14 to 55.16)
Western Sub-Saharan Africa	75 to 79	49.04<br>(47 to 51.17)
Western Sub-Saharan Africa	80 to 84	37.68<br>(35.67 to 39.81)
Western Sub-Saharan Africa	85 to 89	25.53<br>(23.37 to 27.9)
Western Sub-Saharan Africa	90 to 94	19.36<br>(16.3 to 23)
Western Sub-Saharan Africa	95+	17.27<br>(12.03 to 24.8)

ILD&PS<br>Location	Period	Prevalence rate ratio
Global	1992 to 1996	0.82<br>(0.8 to 0.83)
Global	1997 to 2001	0.85<br>(0.84 to 0.87)
Global	2002 to 2006	0.89<br>(0.88 to 0.9)

Global	2007 to 2011	0.96<br>(0.95 to 0.98)
Global	2012 to 2016	1<br>(1 to 1)
Global	2017 to 2021	1.03<br>(1.01 to 1.04)
High SDI	1992 to 1996	0.79<br>(0.77 to 0.8)
High SDI	1997 to 2001	0.85<br>(0.83 to 0.87)
High SDI	2002 to 2006	0.9<br>(0.88 to 0.91)
High SDI	2007 to 2011	0.96<br>(0.94 to 0.97)
High SDI	2012 to 2016	1<br>(1 to 1)
High SDI	2017 to 2021	1.05<br>(1.04 to 1.07)
High-middle SDI	1992 to 1996	0.72<br>(0.69 to 0.76)
High-middle SDI	1997 to 2001	0.76<br>(0.73 to 0.78)
High-middle SDI	2002 to 2006	0.81<br>(0.79 to 0.84)
High-middle SDI	2007 to 2011	0.95<br>(0.93 to 0.97)
High-middle SDI	2012 to 2016	1<br>(1 to 1)
High-middle SDI	2017 to 2021	1.02<br>(1 to 1.04)
Middle SDI	1992 to 1996	0.75<br>(0.73 to 0.77)
Middle SDI	1997 to 2001	0.77<br>(0.75 to 0.78)
Middle SDI	2002 to 2006	0.81<br>(0.8 to 0.82)
Middle SDI	2007 to 2011	0.94<br>(0.93 to 0.95)
Middle SDI	2012 to 2016	1<br>(1 to 1)
Middle SDI	2017 to 2021	1.03<br>(1.02 to 1.04)
Low-middle SDI	1992 to 1996	0.87<br>(0.86 to 0.88)
Low-middle SDI	1997 to 2001	0.88<br>(0.87 to 0.89)
Low-middle SDI	2002 to 2006	0.9<br>(0.89 to 0.9)
Low-middle SDI	2007 to 2011	0.95<br>(0.95 to 0.96)
Low-middle SDI	2012 to 2016	1<br>(1 to 1)
Low-middle SDI	2017 to 2021	1.04<br>(1.03 to 1.05)
Low SDI	1992 to 1996	0.95<br>(0.92 to 0.99)
Low SDI	1997 to 2001	0.94<br>(0.92 to 0.97)
Low SDI	2002 to 2006	0.94<br>(0.92 to 0.96)
Low SDI	2007 to 2011	0.97<br>(0.96 to 0.99)
Low SDI	2012 to 2016	1<br>(1 to 1)
Low SDI	2017 to 2021	1.04<br>(1.02 to 1.05)

Andean Latin America	1992 to 1996	0.54<br>(0.53 to 0.56)
Andean Latin America	1997 to 2001	0.59<br>(0.58 to 0.6)
Andean Latin America	2002 to 2006	0.69<br>(0.68 to 0.7)
Andean Latin America	2007 to 2011	0.83<br>(0.82 to 0.84)
Andean Latin America	2012 to 2016	1<br>(1 to 1)
Andean Latin America	2017 to 2021	1.09<br>(1.07 to 1.1)
Australasia	1992 to 1996	0.61<br>(0.59 to 0.64)
Australasia	1997 to 2001	0.7<br>(0.68 to 0.72)
Australasia	2002 to 2006	0.82<br>(0.8 to 0.84)
Australasia	2007 to 2011	0.91<br>(0.89 to 0.93)
Australasia	2012 to 2016	1<br>(1 to 1)
Australasia	2017 to 2021	1.06<br>(1.04 to 1.08)
Caribbean	1992 to 1996	0.7<br>(0.65 to 0.76)
Caribbean	1997 to 2001	0.76<br>(0.72 to 0.81)
Caribbean	2002 to 2006	0.85<br>(0.81 to 0.89)
Caribbean	2007 to 2011	0.94<br>(0.9 to 0.98)
Caribbean	2012 to 2016	1<br>(1 to 1)
Caribbean	2017 to 2021	1.05<br>(1.01 to 1.09)
Central Asia	1992 to 1996	0.96<br>(0.92 to 1)
Central Asia	1997 to 2001	0.87<br>(0.84 to 0.91)
Central Asia	2002 to 2006	0.83<br>(0.81 to 0.86)
Central Asia	2007 to 2011	0.88<br>(0.86 to 0.9)
Central Asia	2012 to 2016	1<br>(1 to 1)
Central Asia	2017 to 2021	1.14<br>(1.11 to 1.17)
Central Europe	1992 to 1996	0.88<br>(0.85 to 0.92)
Central Europe	1997 to 2001	0.86<br>(0.83 to 0.88)
Central Europe	2002 to 2006	0.89<br>(0.86 to 0.91)
Central Europe	2007 to 2011	0.95<br>(0.93 to 0.96)
Central Europe	2012 to 2016	1<br>(1 to 1)
Central Europe	2017 to 2021	1.02<br>(1 to 1.04)
Central Latin America	1992 to 1996	0.83<br>(0.8 to 0.86)
Central Latin America	1997 to 2001	0.86<br>(0.84 to 0.89)
Central Latin America	2002 to 2006	0.9<br>(0.88 to 0.93)

Central Latin America	2007 to 2011	0.94<br>(0.92 to 0.96)
Central Latin America	2012 to 2016	1<br>(1 to 1)
Central Latin America	2017 to 2021	1.05<br>(1.03 to 1.08)
Central Sub-Saharan Africa	1992 to 1996	0.96<br>(0.82 to 1.12)
Central Sub-Saharan Africa	1997 to 2001	0.93<br>(0.82 to 1.05)
Central Sub-Saharan Africa	2002 to 2006	0.92<br>(0.84 to 1)
Central Sub-Saharan Africa	2007 to 2011	0.95<br>(0.89 to 1.01)
Central Sub-Saharan Africa	2012 to 2016	1<br>(1 to 1)
Central Sub-Saharan Africa	2017 to 2021	1.05<br>(0.99 to 1.11)
East Asia	1992 to 1996	0.66<br>(0.59 to 0.75)
East Asia	1997 to 2001	0.66<br>(0.6 to 0.72)
East Asia	2002 to 2006	0.69<br>(0.65 to 0.74)
East Asia	2007 to 2011	0.93<br>(0.89 to 0.97)
East Asia	2012 to 2016	1<br>(1 to 1)
East Asia	2017 to 2021	1.01<br>(0.97 to 1.05)
Eastern Europe	1992 to 1996	1.89<br>(1.83 to 1.96)
Eastern Europe	1997 to 2001	1.67<br>(1.63 to 1.72)
Eastern Europe	2002 to 2006	1.39<br>(1.36 to 1.43)
Eastern Europe	2007 to 2011	1.16<br>(1.14 to 1.19)
Eastern Europe	2012 to 2016	1<br>(1 to 1)
Eastern Europe	2017 to 2021	0.92<br>(0.9 to 0.93)
Eastern Sub-Saharan Africa	1992 to 1996	0.96<br>(0.87 to 1.05)
Eastern Sub-Saharan Africa	1997 to 2001	0.96<br>(0.89 to 1.04)
Eastern Sub-Saharan Africa	2002 to 2006	0.97<br>(0.91 to 1.02)
Eastern Sub-Saharan Africa	2007 to 2011	0.98<br>(0.95 to 1.02)
Eastern Sub-Saharan Africa	2012 to 2016	1<br>(1 to 1)
Eastern Sub-Saharan Africa	2017 to 2021	1.02<br>(0.99 to 1.06)
High-income Asia Pacific	1992 to 1996	0.84<br>(0.81 to 0.86)
High-income Asia Pacific	1997 to 2001	0.88<br>(0.86 to 0.9)
High-income Asia Pacific	2002 to 2006	0.93<br>(0.91 to 0.95)
High-income Asia Pacific	2007 to 2011	0.98<br>(0.97 to 1)
High-income Asia Pacific	2012 to 2016	1<br>(1 to 1)
High-income Asia Pacific	2017 to 2021	1<br>(0.99 to 1.02)

High-income North America	1992 to 1996	0.86<br>(0.84 to 0.88)
High-income North America	1997 to 2001	0.94<br>(0.92 to 0.96)
High-income North America	2002 to 2006	0.95<br>(0.93 to 0.97)
High-income North America	2007 to 2011	0.96<br>(0.94 to 0.98)
High-income North America	2012 to 2016	1<br>(1 to 1)
High-income North America	2017 to 2021	1.11<br>(1.09 to 1.13)
North Africa and Middle East	1992 to 1996	0.71<br>(0.69 to 0.73)
North Africa and Middle East	1997 to 2001	0.76<br>(0.74 to 0.78)
North Africa and Middle East	2002 to 2006	0.84<br>(0.83 to 0.86)
North Africa and Middle East	2007 to 2011	0.93<br>(0.92 to 0.95)
North Africa and Middle East	2012 to 2016	1<br>(1 to 1)
North Africa and Middle East	2017 to 2021	1.04<br>(1.03 to 1.06)
Oceania	1992 to 1996	0.92<br>(0.61 to 1.38)
Oceania	1997 to 2001	0.94<br>(0.68 to 1.29)
Oceania	2002 to 2006	0.94<br>(0.75 to 1.17)
Oceania	2007 to 2011	0.96<br>(0.84 to 1.1)
Oceania	2012 to 2016	1<br>(1 to 1)
Oceania	2017 to 2021	1.05<br>(0.92 to 1.2)
South Asia	1992 to 1996	0.91<br>(0.89 to 0.92)
South Asia	1997 to 2001	0.91<br>(0.9 to 0.93)
South Asia	2002 to 2006	0.92<br>(0.91 to 0.93)
South Asia	2007 to 2011	0.97<br>(0.96 to 0.98)
South Asia	2012 to 2016	1<br>(1 to 1)
South Asia	2017 to 2021	1.02<br>(1.02 to 1.03)
Southeast Asia	1992 to 1996	0.79<br>(0.76 to 0.81)
Southeast Asia	1997 to 2001	0.83<br>(0.81 to 0.85)
Southeast Asia	2002 to 2006	0.87<br>(0.86 to 0.89)
Southeast Asia	2007 to 2011	0.93<br>(0.92 to 0.95)
Southeast Asia	2012 to 2016	1<br>(1 to 1)
Southeast Asia	2017 to 2021	1.07<br>(1.06 to 1.08)
Southern Latin America	1992 to 1996	0.63<br>(0.62 to 0.65)
Southern Latin America	1997 to 2001	0.73<br>(0.72 to 0.75)
Southern Latin America	2002 to 2006	0.82<br>(0.81 to 0.83)

Southern Latin America	2007 to 2011	0.91<br>(0.89 to 0.92)
Southern Latin America	2012 to 2016	1<br>(1 to 1)
Southern Latin America	2017 to 2021	1.08<br>(1.06 to 1.09)
Southern Sub-Saharan Africa	1992 to 1996	1.07<br>(1.02 to 1.13)
Southern Sub-Saharan Africa	1997 to 2001	1.11<br>(1.06 to 1.15)
Southern Sub-Saharan Africa	2002 to 2006	1.11<br>(1.07 to 1.14)
Southern Sub-Saharan Africa	2007 to 2011	1.07<br>(1.04 to 1.1)
Southern Sub-Saharan Africa	2012 to 2016	1<br>(1 to 1)
Southern Sub-Saharan Africa	2017 to 2021	0.96<br>(0.93 to 0.98)
Tropical Latin America	1992 to 1996	1.07<br>(1.04 to 1.11)
Tropical Latin America	1997 to 2001	1.06<br>(1.03 to 1.09)
Tropical Latin America	2002 to 2006	1.03<br>(1 to 1.05)
Tropical Latin America	2007 to 2011	0.97<br>(0.95 to 0.99)
Tropical Latin America	2012 to 2016	1<br>(1 to 1)
Tropical Latin America	2017 to 2021	1.07<br>(1.05 to 1.09)
Western Europe	1992 to 1996	0.69<br>(0.69 to 0.7)
Western Europe	1997 to 2001	0.76<br>(0.75 to 0.77)
Western Europe	2002 to 2006	0.85<br>(0.85 to 0.86)
Western Europe	2007 to 2011	0.95<br>(0.94 to 0.95)
Western Europe	2012 to 2016	1<br>(1 to 1)
Western Europe	2017 to 2021	1.03<br>(1.02 to 1.03)
Western Sub-Saharan Africa	1992 to 1996	1.06<br>(0.97 to 1.15)
Western Sub-Saharan Africa	1997 to 2001	1.01<br>(0.95 to 1.08)
Western Sub-Saharan Africa	2002 to 2006	0.99<br>(0.94 to 1.04)
Western Sub-Saharan Africa	2007 to 2011	0.99<br>(0.96 to 1.03)
Western Sub-Saharan Africa	2012 to 2016	1<br>(1 to 1)
Western Sub-Saharan Africa	2017 to 2021	1.01<br>(0.98 to 1.05)

ILD&PS<br>Location	Cohort	Prevalence rate ratio
Global	1897 to 1901	0.56<br>(0.44 to 0.71)
Global	1902 to 1906	0.58<br>(0.53 to 0.64)
Global	1907 to 1911	0.64<br>(0.61 to 0.67)
Global	1912 to 1916	0.72<br>(0.7 to 0.75)

Global	1917 to 1921	0.81<br>(0.79 to 0.84)
Global	1922 to 1926	0.87<br>(0.85 to 0.89)
Global	1927 to 1931	0.92<br>(0.9 to 0.94)
Global	1932 to 1936	0.96<br>(0.94 to 0.98)
Global	1937 to 1941	0.98<br>(0.97 to 1)
Global	1942 to 1946	1.03<br>(1.01 to 1.05)
Global	1947 to 1951	1.05<br>(1.03 to 1.07)
Global	1952 to 1956	1.01<br>(0.99 to 1.03)
Global	1957 to 1961	1<br>(1 to 1)
Global	1962 to 1966	0.98<br>(0.96 to 1.01)
High SDI	1897 to 1901	0.52<br>(0.4 to 0.69)
High SDI	1902 to 1906	0.55<br>(0.49 to 0.61)
High SDI	1907 to 1911	0.59<br>(0.56 to 0.63)
High SDI	1912 to 1916	0.67<br>(0.64 to 0.7)
High SDI	1917 to 1921	0.78<br>(0.75 to 0.8)
High SDI	1922 to 1926	0.82<br>(0.79 to 0.84)
High SDI	1927 to 1931	0.89<br>(0.86 to 0.92)
High SDI	1932 to 1936	0.95<br>(0.92 to 0.98)
High SDI	1937 to 1941	0.99<br>(0.96 to 1.01)
High SDI	1942 to 1946	1.03<br>(1 to 1.06)
High SDI	1947 to 1951	1.04<br>(1.02 to 1.07)
High SDI	1952 to 1956	1.02<br>(0.99 to 1.05)
High SDI	1957 to 1961	1<br>(1 to 1)
High SDI	1962 to 1966	0.99<br>(0.95 to 1.03)
High-middle SDI	1897 to 1901	0.38<br>(0.21 to 0.7)
High-middle SDI	1902 to 1906	0.43<br>(0.35 to 0.54)
High-middle SDI	1907 to 1911	0.49<br>(0.45 to 0.55)
High-middle SDI	1912 to 1916	0.59<br>(0.55 to 0.62)
High-middle SDI	1917 to 1921	0.69<br>(0.66 to 0.73)
High-middle SDI	1922 to 1926	0.79<br>(0.76 to 0.82)
High-middle SDI	1927 to 1931	0.84<br>(0.81 to 0.87)
High-middle SDI	1932 to 1936	0.92<br>(0.9 to 0.95)
High-middle SDI	1937 to 1941	0.96<br>(0.93 to 0.99)

High-middle SDI	1942 to 1946	1.03<br>(1 to 1.06)
High-middle SDI	1947 to 1951	1.04<br>(1.01 to 1.07)
High-middle SDI	1952 to 1956	1<br>(0.97 to 1.03)
High-middle SDI	1957 to 1961	1<br>(1 to 1)
High-middle SDI	1962 to 1966	1<br>(0.96 to 1.04)
Middle SDI	1897 to 1901	0.38<br>(0.28 to 0.52)
Middle SDI	1902 to 1906	0.45<br>(0.4 to 0.51)
Middle SDI	1907 to 1911	0.52<br>(0.49 to 0.55)
Middle SDI	1912 to 1916	0.6<br>(0.58 to 0.62)
Middle SDI	1917 to 1921	0.67<br>(0.65 to 0.69)
Middle SDI	1922 to 1926	0.74<br>(0.73 to 0.76)
Middle SDI	1927 to 1931	0.81<br>(0.8 to 0.83)
Middle SDI	1932 to 1936	0.88<br>(0.86 to 0.89)
Middle SDI	1937 to 1941	0.93<br>(0.92 to 0.94)
Middle SDI	1942 to 1946	0.97<br>(0.96 to 0.99)
Middle SDI	1947 to 1951	0.99<br>(0.98 to 1.01)
Middle SDI	1952 to 1956	0.99<br>(0.97 to 1)
Middle SDI	1957 to 1961	1<br>(1 to 1)
Middle SDI	1962 to 1966	1.01<br>(0.99 to 1.02)
Low-middle SDI	1897 to 1901	0.59<br>(0.49 to 0.71)
Low-middle SDI	1902 to 1906	0.65<br>(0.61 to 0.7)
Low-middle SDI	1907 to 1911	0.72<br>(0.7 to 0.75)
Low-middle SDI	1912 to 1916	0.77<br>(0.76 to 0.79)
Low-middle SDI	1917 to 1921	0.82<br>(0.8 to 0.83)
Low-middle SDI	1922 to 1926	0.86<br>(0.85 to 0.87)
Low-middle SDI	1927 to 1931	0.9<br>(0.89 to 0.91)
Low-middle SDI	1932 to 1936	0.94<br>(0.93 to 0.95)
Low-middle SDI	1937 to 1941	0.97<br>(0.96 to 0.97)
Low-middle SDI	1942 to 1946	0.99<br>(0.98 to 1)
Low-middle SDI	1947 to 1951	1.01<br>(1 to 1.01)
Low-middle SDI	1952 to 1956	1.01<br>(1 to 1.02)
Low-middle SDI	1957 to 1961	1<br>(1 to 1)
Low-middle SDI	1962 to 1966	1<br>(0.99 to 1.01)

Low SDI	1897 to 1901	0.77 (0.45 to 1.33)
Low SDI	1902 to 1906	0.83 (0.67 to 1.01)
Low SDI	1907 to 1911	0.91 (0.83 to 0.99)
Low SDI	1912 to 1916	0.91 (0.87 to 0.95)
Low SDI	1917 to 1921	0.92 (0.89 to 0.94)
Low SDI	1922 to 1926	0.93 (0.91 to 0.96)
Low SDI	1927 to 1931	0.96 (0.94 to 0.97)
Low SDI	1932 to 1936	0.98 (0.97 to 1)
Low SDI	1937 to 1941	1.01 (0.99 to 1.02)
Low SDI	1942 to 1946	1.02 (1 to 1.03)
Low SDI	1947 to 1951	1.04 (1.02 to 1.05)
Low SDI	1952 to 1956	1.03 (1.02 to 1.05)
Low SDI	1957 to 1961	1 (1 to 1)
Low SDI	1962 to 1966	0.99 (0.97 to 1.01)
Andean Latin America	1897 to 1901	0.15 (0.12 to 0.19)
Andean Latin America	1902 to 1906	0.19 (0.17 to 0.21)
Andean Latin America	1907 to 1911	0.24 (0.23 to 0.26)
Andean Latin America	1912 to 1916	0.3 (0.29 to 0.32)
Andean Latin America	1917 to 1921	0.38 (0.36 to 0.39)
Andean Latin America	1922 to 1926	0.46 (0.44 to 0.47)
Andean Latin America	1927 to 1931	0.54 (0.53 to 0.56)
Andean Latin America	1932 to 1936	0.63 (0.61 to 0.65)
Andean Latin America	1937 to 1941	0.7 (0.69 to 0.72)
Andean Latin America	1942 to 1946	0.78 (0.76 to 0.8)
Andean Latin America	1947 to 1951	0.86 (0.84 to 0.88)
Andean Latin America	1952 to 1956	0.94 (0.91 to 0.96)
Andean Latin America	1957 to 1961	1 (1 to 1)
Andean Latin America	1962 to 1966	1.06 (1.03 to 1.1)
Australasia	1897 to 1901	0.27 (0.18 to 0.41)
Australasia	1902 to 1906	0.3 (0.26 to 0.36)
Australasia	1907 to 1911	0.35 (0.32 to 0.38)
Australasia	1912 to 1916	0.42 (0.39 to 0.44)
Australasia	1917 to 1921	0.49 (0.47 to 0.52)

Australasia	1922 to 1926	0.58<br>(0.56 to 0.61)
Australasia	1927 to 1931	0.67<br>(0.65 to 0.7)
Australasia	1932 to 1936	0.76<br>(0.73 to 0.79)
Australasia	1937 to 1941	0.83<br>(0.8 to 0.87)
Australasia	1942 to 1946	0.89<br>(0.86 to 0.93)
Australasia	1947 to 1951	0.94<br>(0.91 to 0.98)
Australasia	1952 to 1956	0.97<br>(0.94 to 1.01)
Australasia	1957 to 1961	1<br>(1 to 1)
Australasia	1962 to 1966	1.04<br>(0.98 to 1.1)
Caribbean	1897 to 1901	0.35<br>(0.14 to 0.87)
Caribbean	1902 to 1906	0.39<br>(0.28 to 0.56)
Caribbean	1907 to 1911	0.45<br>(0.38 to 0.54)
Caribbean	1912 to 1916	0.52<br>(0.47 to 0.59)
Caribbean	1917 to 1921	0.58<br>(0.53 to 0.64)
Caribbean	1922 to 1926	0.65<br>(0.6 to 0.7)
Caribbean	1927 to 1931	0.71<br>(0.66 to 0.76)
Caribbean	1932 to 1936	0.77<br>(0.72 to 0.82)
Caribbean	1937 to 1941	0.82<br>(0.78 to 0.87)
Caribbean	1942 to 1946	0.87<br>(0.82 to 0.92)
Caribbean	1947 to 1951	0.93<br>(0.88 to 0.98)
Caribbean	1952 to 1956	0.96<br>(0.91 to 1.02)
Caribbean	1957 to 1961	1<br>(1 to 1)
Caribbean	1962 to 1966	1.01<br>(0.94 to 1.09)
Central Asia	1897 to 1901	0.68<br>(0.42 to 1.12)
Central Asia	1902 to 1906	0.73<br>(0.6 to 0.88)
Central Asia	1907 to 1911	0.74<br>(0.66 to 0.83)
Central Asia	1912 to 1916	0.75<br>(0.7 to 0.81)
Central Asia	1917 to 1921	0.8<br>(0.75 to 0.85)
Central Asia	1922 to 1926	0.86<br>(0.82 to 0.91)
Central Asia	1927 to 1931	0.94<br>(0.9 to 0.97)
Central Asia	1932 to 1936	1<br>(0.97 to 1.04)
Central Asia	1937 to 1941	1.01<br>(0.98 to 1.05)
Central Asia	1942 to 1946	1.03<br>(1 to 1.07)

Central Asia	1947 to 1951	1.04<br>(1 to 1.08)
Central Asia	1952 to 1956	1.02<br>(0.99 to 1.06)
Central Asia	1957 to 1961	1<br>(1 to 1)
Central Asia	1962 to 1966	0.97<br>(0.93 to 1.02)
Central Europe	1897 to 1901	0.59<br>(0.32 to 1.08)
Central Europe	1902 to 1906	0.64<br>(0.52 to 0.79)
Central Europe	1907 to 1911	0.73<br>(0.67 to 0.8)
Central Europe	1912 to 1916	0.82<br>(0.78 to 0.86)
Central Europe	1917 to 1921	0.85<br>(0.81 to 0.88)
Central Europe	1922 to 1926	0.87<br>(0.85 to 0.9)
Central Europe	1927 to 1931	0.92<br>(0.9 to 0.94)
Central Europe	1932 to 1936	0.95<br>(0.93 to 0.97)
Central Europe	1937 to 1941	0.97<br>(0.95 to 0.99)
Central Europe	1942 to 1946	0.99<br>(0.97 to 1.01)
Central Europe	1947 to 1951	1.01<br>(0.99 to 1.03)
Central Europe	1952 to 1956	1.01<br>(0.99 to 1.04)
Central Europe	1957 to 1961	1<br>(1 to 1)
Central Europe	1962 to 1966	0.97<br>(0.94 to 1)
Central Latin America	1897 to 1901	0.46<br>(0.27 to 0.76)
Central Latin America	1902 to 1906	0.54<br>(0.44 to 0.65)
Central Latin America	1907 to 1911	0.65<br>(0.59 to 0.71)
Central Latin America	1912 to 1916	0.75<br>(0.71 to 0.8)
Central Latin America	1917 to 1921	0.83<br>(0.79 to 0.87)
Central Latin America	1922 to 1926	0.88<br>(0.85 to 0.92)
Central Latin America	1927 to 1931	0.92<br>(0.89 to 0.96)
Central Latin America	1932 to 1936	0.96<br>(0.93 to 1)
Central Latin America	1937 to 1941	0.98<br>(0.95 to 1.01)
Central Latin America	1942 to 1946	0.99<br>(0.96 to 1.02)
Central Latin America	1947 to 1951	0.99<br>(0.97 to 1.02)
Central Latin America	1952 to 1956	0.99<br>(0.97 to 1.02)
Central Latin America	1957 to 1961	1<br>(1 to 1)
Central Latin America	1962 to 1966	1.01<br>(0.97 to 1.05)
Central Sub-Saharan Africa	1897 to 1901	0.99<br>(0.12 to 7.96)

Central Sub-Saharan Africa	1902 to 1906	0.75<br>(0.27 to 2.1)
Central Sub-Saharan Africa	1907 to 1911	0.79<br>(0.51 to 1.23)
Central Sub-Saharan Africa	1912 to 1916	0.81<br>(0.66 to 0.99)
Central Sub-Saharan Africa	1917 to 1921	0.83<br>(0.73 to 0.93)
Central Sub-Saharan Africa	1922 to 1926	0.84<br>(0.77 to 0.91)
Central Sub-Saharan Africa	1927 to 1931	0.85<br>(0.79 to 0.92)
Central Sub-Saharan Africa	1932 to 1936	0.87<br>(0.82 to 0.93)
Central Sub-Saharan Africa	1937 to 1941	0.89<br>(0.84 to 0.94)
Central Sub-Saharan Africa	1942 to 1946	0.91<br>(0.86 to 0.97)
Central Sub-Saharan Africa	1947 to 1951	0.94<br>(0.89 to 1)
Central Sub-Saharan Africa	1952 to 1956	0.97<br>(0.92 to 1.03)
Central Sub-Saharan Africa	1957 to 1961	1<br>(1 to 1)
Central Sub-Saharan Africa	1962 to 1966	1.03<br>(0.95 to 1.1)
East Asia	1897 to 1901	0.3<br>(0.04 to 2.05)
East Asia	1902 to 1906	0.34<br>(0.19 to 0.61)
East Asia	1907 to 1911	0.39<br>(0.31 to 0.49)
East Asia	1912 to 1916	0.46<br>(0.41 to 0.52)
East Asia	1917 to 1921	0.54<br>(0.5 to 0.58)
East Asia	1922 to 1926	0.63<br>(0.6 to 0.67)
East Asia	1927 to 1931	0.73<br>(0.69 to 0.77)
East Asia	1932 to 1936	0.82<br>(0.79 to 0.86)
East Asia	1937 to 1941	0.9<br>(0.86 to 0.94)
East Asia	1942 to 1946	0.95<br>(0.91 to 0.99)
East Asia	1947 to 1951	0.99<br>(0.95 to 1.03)
East Asia	1952 to 1956	0.99<br>(0.95 to 1.03)
East Asia	1957 to 1961	1<br>(1 to 1)
East Asia	1962 to 1966	0.99<br>(0.93 to 1.04)
Eastern Europe	1897 to 1901	6.03<br>(3.74 to 9.72)
Eastern Europe	1902 to 1906	5.23<br>(4.39 to 6.24)
Eastern Europe	1907 to 1911	4.65<br>(4.29 to 5.04)
Eastern Europe	1912 to 1916	3.95<br>(3.76 to 4.14)
Eastern Europe	1917 to 1921	3.34<br>(3.23 to 3.47)
Eastern Europe	1922 to 1926	2.89<br>(2.8 to 2.97)

Eastern Europe	1927 to 1931	2.44<br>(2.38 to 2.5)
Eastern Europe	1932 to 1936	2.09<br>(2.05 to 2.14)
Eastern Europe	1937 to 1941	1.79<br>(1.75 to 1.82)
Eastern Europe	1942 to 1946	1.56<br>(1.53 to 1.6)
Eastern Europe	1947 to 1951	1.32<br>(1.29 to 1.35)
Eastern Europe	1952 to 1956	1.15<br>(1.12 to 1.17)
Eastern Europe	1957 to 1961	1<br>(1 to 1)
Eastern Europe	1962 to 1966	0.87<br>(0.85 to 0.9)
Eastern Sub-Saharan Africa	1897 to 1901	1<br>(0.23 to 4.3)
Eastern Sub-Saharan Africa	1902 to 1906	0.88<br>(0.51 to 1.52)
Eastern Sub-Saharan Africa	1907 to 1911	0.86<br>(0.68 to 1.08)
Eastern Sub-Saharan Africa	1912 to 1916	0.84<br>(0.75 to 0.95)
Eastern Sub-Saharan Africa	1917 to 1921	0.85<br>(0.79 to 0.92)
Eastern Sub-Saharan Africa	1922 to 1926	0.87<br>(0.82 to 0.92)
Eastern Sub-Saharan Africa	1927 to 1931	0.88<br>(0.84 to 0.92)
Eastern Sub-Saharan Africa	1932 to 1936	0.9<br>(0.86 to 0.94)
Eastern Sub-Saharan Africa	1937 to 1941	0.92<br>(0.88 to 0.95)
Eastern Sub-Saharan Africa	1942 to 1946	0.94<br>(0.9 to 0.97)
Eastern Sub-Saharan Africa	1947 to 1951	0.96<br>(0.93 to 1)
Eastern Sub-Saharan Africa	1952 to 1956	0.98<br>(0.95 to 1.02)
Eastern Sub-Saharan Africa	1957 to 1961	1<br>(1 to 1)
Eastern Sub-Saharan Africa	1962 to 1966	1.02<br>(0.97 to 1.06)
High-income Asia Pacific	1897 to 1901	0.65<br>(0.45 to 0.95)
High-income Asia Pacific	1902 to 1906	0.68<br>(0.59 to 0.78)
High-income Asia Pacific	1907 to 1911	0.72<br>(0.67 to 0.77)
High-income Asia Pacific	1912 to 1916	0.77<br>(0.74 to 0.81)
High-income Asia Pacific	1917 to 1921	0.83<br>(0.8 to 0.86)
High-income Asia Pacific	1922 to 1926	0.89<br>(0.86 to 0.92)
High-income Asia Pacific	1927 to 1931	0.96<br>(0.93 to 1)
High-income Asia Pacific	1932 to 1936	1<br>(0.97 to 1.03)
High-income Asia Pacific	1937 to 1941	1.02<br>(0.99 to 1.05)
High-income Asia Pacific	1942 to 1946	1.03<br>(1 to 1.06)
High-income Asia Pacific	1947 to 1951	1.04<br>(1.01 to 1.07)

High-income Asia Pacific	1952 to 1956	1.03<br>(1 to 1.06)
High-income Asia Pacific	1957 to 1961	1<br>(1 to 1)
High-income Asia Pacific	1962 to 1966	0.99<br>(0.94 to 1.03)
High-income North America	1897 to 1901	0.54<br>(0.43 to 0.67)
High-income North America	1902 to 1906	0.61<br>(0.55 to 0.68)
High-income North America	1907 to 1911	0.69<br>(0.65 to 0.73)
High-income North America	1912 to 1916	0.78<br>(0.74 to 0.81)
High-income North America	1917 to 1921	0.86<br>(0.83 to 0.9)
High-income North America	1922 to 1926	0.93<br>(0.9 to 0.97)
High-income North America	1927 to 1931	0.98<br>(0.95 to 1.02)
High-income North America	1932 to 1936	1.02<br>(0.99 to 1.05)
High-income North America	1937 to 1941	1.04<br>(1.01 to 1.07)
High-income North America	1942 to 1946	1.04<br>(1.01 to 1.07)
High-income North America	1947 to 1951	1.04<br>(1 to 1.07)
High-income North America	1952 to 1956	1.02<br>(0.99 to 1.05)
High-income North America	1957 to 1961	1<br>(1 to 1)
High-income North America	1962 to 1966	0.97<br>(0.93 to 1.01)
North Africa and Middle East	1897 to 1901	0.37<br>(0.26 to 0.53)
North Africa and Middle East	1902 to 1906	0.4<br>(0.34 to 0.47)
North Africa and Middle East	1907 to 1911	0.45<br>(0.41 to 0.49)
North Africa and Middle East	1912 to 1916	0.5<br>(0.48 to 0.53)
North Africa and Middle East	1917 to 1921	0.54<br>(0.52 to 0.56)
North Africa and Middle East	1922 to 1926	0.58<br>(0.57 to 0.6)
North Africa and Middle East	1927 to 1931	0.64<br>(0.63 to 0.65)
North Africa and Middle East	1932 to 1936	0.68<br>(0.67 to 0.7)
North Africa and Middle East	1937 to 1941	0.74<br>(0.73 to 0.75)
North Africa and Middle East	1942 to 1946	0.81<br>(0.79 to 0.82)
North Africa and Middle East	1947 to 1951	0.87<br>(0.86 to 0.89)
North Africa and Middle East	1952 to 1956	0.93<br>(0.92 to 0.95)
North Africa and Middle East	1957 to 1961	1<br>(1 to 1)
North Africa and Middle East	1962 to 1966	1.07<br>(1.05 to 1.09)
Oceania	1897 to 1901	0.9<br>(0 to 571.19)
Oceania	1902 to 1906	0.74<br>(0.07 to 8.38)

Oceania	1907 to 1911	0.74<br>(0.31 to 1.75)
Oceania	1912 to 1916	0.77<br>(0.52 to 1.13)
Oceania	1917 to 1921	0.83<br>(0.66 to 1.04)
Oceania	1922 to 1926	0.88<br>(0.74 to 1.03)
Oceania	1927 to 1931	0.9<br>(0.79 to 1.04)
Oceania	1932 to 1936	0.91<br>(0.81 to 1.02)
Oceania	1937 to 1941	0.92<br>(0.83 to 1.03)
Oceania	1942 to 1946	0.94<br>(0.84 to 1.05)
Oceania	1947 to 1951	0.98<br>(0.88 to 1.08)
Oceania	1952 to 1956	0.99<br>(0.89 to 1.1)
Oceania	1957 to 1961	1<br>(1 to 1)
Oceania	1962 to 1966	1<br>(0.88 to 1.14)
South Asia	1897 to 1901	0.68<br>(0.54 to 0.86)
South Asia	1902 to 1906	0.73<br>(0.67 to 0.8)
South Asia	1907 to 1911	0.79<br>(0.76 to 0.82)
South Asia	1912 to 1916	0.84<br>(0.82 to 0.86)
South Asia	1917 to 1921	0.88<br>(0.87 to 0.9)
South Asia	1922 to 1926	0.91<br>(0.9 to 0.92)
South Asia	1927 to 1931	0.94<br>(0.93 to 0.95)
South Asia	1932 to 1936	0.96<br>(0.95 to 0.96)
South Asia	1937 to 1941	0.97<br>(0.96 to 0.98)
South Asia	1942 to 1946	0.98<br>(0.98 to 0.99)
South Asia	1947 to 1951	1<br>(0.99 to 1)
South Asia	1952 to 1956	1<br>(0.99 to 1.01)
South Asia	1957 to 1961	1<br>(1 to 1)
South Asia	1962 to 1966	0.99<br>(0.98 to 1)
Southeast Asia	1897 to 1901	0.47<br>(0.31 to 0.7)
Southeast Asia	1902 to 1906	0.49<br>(0.41 to 0.58)
Southeast Asia	1907 to 1911	0.54<br>(0.49 to 0.58)
Southeast Asia	1912 to 1916	0.6<br>(0.57 to 0.63)
Southeast Asia	1917 to 1921	0.66<br>(0.64 to 0.69)
Southeast Asia	1922 to 1926	0.72<br>(0.7 to 0.74)
Southeast Asia	1927 to 1931	0.76<br>(0.75 to 0.78)

Southeast Asia	1932 to 1936	0.8 (0.79 to 0.82)
Southeast Asia	1937 to 1941	0.84 (0.83 to 0.86)
Southeast Asia	1942 to 1946	0.88 (0.87 to 0.9)
Southeast Asia	1947 to 1951	0.92 (0.91 to 0.94)
Southeast Asia	1952 to 1956	0.96 (0.95 to 0.98)
Southeast Asia	1957 to 1961	1 (1 to 1)
Southeast Asia	1962 to 1966	1.03 (1.01 to 1.05)
Southern Latin America	1897 to 1901	0.28 (0.21 to 0.36)
Southern Latin America	1902 to 1906	0.31 (0.28 to 0.34)
Southern Latin America	1907 to 1911	0.36 (0.34 to 0.38)
Southern Latin America	1912 to 1916	0.42 (0.41 to 0.44)
Southern Latin America	1917 to 1921	0.48 (0.47 to 0.5)
Southern Latin America	1922 to 1926	0.55 (0.53 to 0.56)
Southern Latin America	1927 to 1931	0.62 (0.6 to 0.63)
Southern Latin America	1932 to 1936	0.69 (0.67 to 0.7)
Southern Latin America	1937 to 1941	0.75 (0.73 to 0.76)
Southern Latin America	1942 to 1946	0.81 (0.8 to 0.83)
Southern Latin America	1947 to 1951	0.87 (0.85 to 0.89)
Southern Latin America	1952 to 1956	0.94 (0.92 to 0.95)
Southern Latin America	1957 to 1961	1 (1 to 1)
Southern Latin America	1962 to 1966	1.06 (1.03 to 1.09)
Southern Sub-Saharan Africa	1897 to 1901	1.45 (0.78 to 2.69)
Southern Sub-Saharan Africa	1902 to 1906	1.38 (1.08 to 1.75)
Southern Sub-Saharan Africa	1907 to 1911	1.32 (1.17 to 1.5)
Southern Sub-Saharan Africa	1912 to 1916	1.25 (1.15 to 1.35)
Southern Sub-Saharan Africa	1917 to 1921	1.2 (1.14 to 1.27)
Southern Sub-Saharan Africa	1922 to 1926	1.18 (1.13 to 1.24)
Southern Sub-Saharan Africa	1927 to 1931	1.15 (1.11 to 1.2)
Southern Sub-Saharan Africa	1932 to 1936	1.14 (1.09 to 1.18)
Southern Sub-Saharan Africa	1937 to 1941	1.11 (1.08 to 1.15)
Southern Sub-Saharan Africa	1942 to 1946	1.09 (1.06 to 1.13)
Southern Sub-Saharan Africa	1947 to 1951	1.07 (1.03 to 1.1)
Southern Sub-Saharan Africa	1952 to 1956	1.03 (1 to 1.07)

Southern Sub-Saharan Africa	1957 to 1961	1<br>(1 to 1)
Southern Sub-Saharan Africa	1962 to 1966	0.96<br>(0.92 to 1.01)
Tropical Latin America	1897 to 1901	0.92<br>(0.6 to 1.41)
Tropical Latin America	1902 to 1906	1.06<br>(0.9 to 1.24)
Tropical Latin America	1907 to 1911	1.16<br>(1.07 to 1.27)
Tropical Latin America	1912 to 1916	1.24<br>(1.17 to 1.31)
Tropical Latin America	1917 to 1921	1.29<br>(1.24 to 1.35)
Tropical Latin America	1922 to 1926	1.33<br>(1.28 to 1.38)
Tropical Latin America	1927 to 1931	1.35<br>(1.31 to 1.4)
Tropical Latin America	1932 to 1936	1.35<br>(1.31 to 1.39)
Tropical Latin America	1937 to 1941	1.31<br>(1.27 to 1.35)
Tropical Latin America	1942 to 1946	1.23<br>(1.2 to 1.27)
Tropical Latin America	1947 to 1951	1.15<br>(1.11 to 1.18)
Tropical Latin America	1952 to 1956	1.07<br>(1.04 to 1.1)
Tropical Latin America	1957 to 1961	1<br>(1 to 1)
Tropical Latin America	1962 to 1966	0.93<br>(0.9 to 0.97)
Western Europe	1897 to 1901	0.38<br>(0.34 to 0.43)
Western Europe	1902 to 1906	0.41<br>(0.39 to 0.43)
Western Europe	1907 to 1911	0.46<br>(0.45 to 0.47)
Western Europe	1912 to 1916	0.53<br>(0.52 to 0.54)
Western Europe	1917 to 1921	0.62<br>(0.62 to 0.63)
Western Europe	1922 to 1926	0.69<br>(0.68 to 0.69)
Western Europe	1927 to 1931	0.77<br>(0.76 to 0.78)
Western Europe	1932 to 1936	0.84<br>(0.84 to 0.85)
Western Europe	1937 to 1941	0.89<br>(0.89 to 0.9)
Western Europe	1942 to 1946	0.95<br>(0.94 to 0.96)
Western Europe	1947 to 1951	0.99<br>(0.98 to 1)
Western Europe	1952 to 1956	0.99<br>(0.98 to 1)
Western Europe	1957 to 1961	1<br>(1 to 1)
Western Europe	1962 to 1966	1<br>(0.99 to 1.02)
Western Sub-Saharan Africa	1897 to 1901	0.9<br>(0.27 to 2.94)
Western Sub-Saharan Africa	1902 to 1906	1.04<br>(0.65 to 1.65)
Western Sub-Saharan Africa	1907 to 1911	1.09<br>(0.88 to 1.34)

Western Sub-Saharan Africa	1912 to 1916	1.14 (1.02 to 1.27)
Western Sub-Saharan Africa	1917 to 1921	1.15 (1.08 to 1.24)
Western Sub-Saharan Africa	1922 to 1926	1.14 (1.09 to 1.21)
Western Sub-Saharan Africa	1927 to 1931	1.15 (1.1 to 1.2)
Western Sub-Saharan Africa	1932 to 1936	1.12 (1.08 to 1.16)
Western Sub-Saharan Africa	1937 to 1941	1.08 (1.04 to 1.12)
Western Sub-Saharan Africa	1942 to 1946	1.05 (1.01 to 1.09)
Western Sub-Saharan Africa	1947 to 1951	1.03 (1 to 1.07)
Western Sub-Saharan Africa	1952 to 1956	1.02 (0.98 to 1.05)
Western Sub-Saharan Africa	1957 to 1961	1 (1 to 1)
Western Sub-Saharan Africa	1962 to 1966	0.99 (0.94 to 1.03)

Table S11 *Net Drift* in the Prevalence of CRDs, COPD, Asthma, and ILD & PS in 11 Example Countries (1990-2021)

Cause Location	CRDs Net Drift (%/year)	COPD Net Drift (%/year)	Asthma Net Drift (%/year)	ILD&PS Net Drift (%/year)
Germany	-0.66 (-0.79 to -0.52)	0.12 (0.08 to 0.16)	-3.81 (-4.07 to -3.55)	1.57 (1.36 to 1.79)
Brazil	-0.54 (-0.58 to -0.5)	-0.09 (-0.12 to -0.06)	-2.64 (-2.87 to -2.42)	-0.18 (-0.34 to -0.03)
Afghanistan	-0.21 (-0.29 to -0.14)	0.43 (0.38 to 0.49)	-1.26 (-1.34 to -1.17)	1.16 (-0.69 to 3.05)
Japan	-2.77 (-2.9 to -2.64)	-0.66 (-0.72 to -0.61)	-6.15 (-6.41 to -5.88)	0.69 (0.56 to 0.82)
Saudi Arabia	0.85 (0.78 to 0.91)	1.37 (1.32 to 1.42)	-0.68 (-0.82 to -0.54)	2.44 (2.02 to 2.86)
China	-0.38 (-0.53 to -0.23)	0.08 (-0.07 to 0.22)	-3.09 (-3.35 to -2.83)	2.08 (1.46 to 2.7)
Burundi	-0.18 (-0.3 to -0.07)	0.39 (0.26 to 0.52)	-1.14 (-1.34 to -0.93)	0.43 (-2.54 to 3.5)
India	0.05 (0.02 to 0.08)	0.27 (0.25 to 0.29)	-0.76 (-0.93 to -0.58)	0.49 (0.38 to 0.59)
Russia	-1.46 (-1.52 to -1.4)	-0.34 (-0.44 to -0.25)	-5.62 (-5.84 to -5.41)	-1.77 (-1.99 to -1.54)
United Kingdom	-0.5 (-0.6 to -0.39)	0.24 (0.21 to 0.26)	-1.7 (-1.96 to -1.43)	1.61 (1.54 to 1.68)
United States	0.87 (0.74 to 0.99)	0.56 (0.49 to 0.64)	1.95 (1.76 to 2.15)	0.75 (0.65 to 0.85)

Table S12 APC Model Analysis Results of CRDs Prevalence Among Adults Aged 55 and Above in 11 Example Countries

CRDs<br>Location	Age	Local drift (%/year)
Germany	55 to 59	-1.58<br>(-1.81 to -1.34)
Germany	60 to 64	-1.36<br>(-1.55 to -1.18)
Germany	65 to 69	-1.17<br>(-1.33 to -1)
Germany	70 to 74	-0.98<br>(-1.14 to -0.82)
Germany	75 to 79	-0.75<br>(-0.93 to -0.58)
Germany	80 to 84	-0.44<br>(-0.63 to -0.25)
Germany	85 to 89	-0.1<br>(-0.33 to 0.14)
Germany	90 to 94	0.19<br>(-0.2 to 0.57)
Germany	95+	0.45<br>(-0.36 to 1.26)
Brazil	55 to 59	-1.32<br>(-1.37 to -1.26)
Brazil	60 to 64	-1.13<br>(-1.17 to -1.09)
Brazil	65 to 69	-0.92<br>(-0.96 to -0.88)
Brazil	70 to 74	-0.69<br>(-0.74 to -0.65)
Brazil	75 to 79	-0.47<br>(-0.52 to -0.43)
Brazil	80 to 84	-0.27<br>(-0.33 to -0.22)
Brazil	85 to 89	-0.11<br>(-0.19 to -0.04)
Brazil	90 to 94	-0.02<br>(-0.15 to 0.11)
Brazil	95+	0.02<br>(-0.28 to 0.32)
Afghanistan	55 to 59	-0.97<br>(-1.03 to -0.91)
Afghanistan	60 to 64	-0.79<br>(-0.84 to -0.75)
Afghanistan	65 to 69	-0.64<br>(-0.68 to -0.6)
Afghanistan	70 to 74	-0.48<br>(-0.53 to -0.43)
Afghanistan	75 to 79	-0.28<br>(-0.34 to -0.22)
Afghanistan	80 to 84	-0.04<br>(-0.12 to 0.04)
Afghanistan	85 to 89	0.23<br>(0.09 to 0.36)
Afghanistan	90 to 94	0.48<br>(0.23 to 0.74)
Afghanistan	95+	0.68<br>(0.05 to 1.32)
Japan	55 to 59	-3.74<br>(-4.07 to -3.4)
Japan	60 to 64	-3.6<br>(-3.83 to -3.37)
Japan	65 to 69	-3.5<br>(-3.68 to -3.33)
Japan	70 to 74	-3.41<br>(-3.57 to -3.25)

Japan	75 to 79	-3.18<br>(-3.34 to -3.02)
Japan	80 to 84	-2.73<br>(-2.91 to -2.56)
Japan	85 to 89	-2.07<br>(-2.3 to -1.84)
Japan	90 to 94	-1.33<br>(-1.71 to -0.96)
Japan	95+	-0.68<br>(-1.49 to 0.13)
Saudi Arabia	55 to 59	0.02<br>(-0.04 to 0.07)
Saudi Arabia	60 to 64	0.19<br>(0.14 to 0.24)
Saudi Arabia	65 to 69	0.37<br>(0.32 to 0.42)
Saudi Arabia	70 to 74	0.56<br>(0.51 to 0.62)
Saudi Arabia	75 to 79	0.78<br>(0.72 to 0.85)
Saudi Arabia	80 to 84	1.04<br>(0.96 to 1.13)
Saudi Arabia	85 to 89	1.34<br>(1.21 to 1.47)
Saudi Arabia	90 to 94	1.6<br>(1.37 to 1.82)
Saudi Arabia	95+	1.81<br>(1.36 to 2.26)
China	55 to 59	-1.41<br>(-1.52 to -1.3)
China	60 to 64	-1.29<br>(-1.37 to -1.21)
China	65 to 69	-1.09<br>(-1.17 to -1.02)
China	70 to 74	-0.83<br>(-0.91 to -0.76)
China	75 to 79	-0.51<br>(-0.59 to -0.42)
China	80 to 84	-0.14<br>(-0.26 to -0.02)
China	85 to 89	0.27<br>(0.07 to 0.47)
China	90 to 94	0.72<br>(0.27 to 1.18)
China	95+	1.14<br>(-0.27 to 2.58)
Burundi	55 to 59	-1.01<br>(-1.07 to -0.94)
Burundi	60 to 64	-0.82<br>(-0.87 to -0.76)
Burundi	65 to 69	-0.6<br>(-0.65 to -0.54)
Burundi	70 to 74	-0.37<br>(-0.44 to -0.31)
Burundi	75 to 79	-0.16<br>(-0.24 to -0.08)
Burundi	80 to 84	0.04<br>(-0.06 to 0.15)
Burundi	85 to 89	0.25<br>(0.07 to 0.43)
Burundi	90 to 94	0.38<br>(0.01 to 0.76)
Burundi	95+	0.62<br>(-0.38 to 1.62)
India	55 to 59	-0.33<br>(-0.35 to -0.3)

India	60 to 64	-0.3<br>(-0.32 to -0.28)
India	65 to 69	-0.21<br>(-0.23 to -0.19)
India	70 to 74	-0.1<br>(-0.12 to -0.08)
India	75 to 79	0.03<br>(0 to 0.05)
India	80 to 84	0.16<br>(0.13 to 0.2)
India	85 to 89	0.29<br>(0.24 to 0.35)
India	90 to 94	0.42<br>(0.31 to 0.52)
India	95+	0.52<br>(0.27 to 0.76)
Russia	55 to 59	-2.93<br>(-3 to -2.86)
Russia	60 to 64	-2.64<br>(-2.7 to -2.59)
Russia	65 to 69	-2.27<br>(-2.32 to -2.22)
Russia	70 to 74	-1.84<br>(-1.9 to -1.79)
Russia	75 to 79	-1.38<br>(-1.44 to -1.32)
Russia	80 to 84	-0.99<br>(-1.06 to -0.91)
Russia	85 to 89	-0.64<br>(-0.75 to -0.53)
Russia	90 to 94	-0.38<br>(-0.58 to -0.18)
Russia	95+	-0.15<br>(-0.65 to 0.35)
United Kingdom	55 to 59	-1.79<br>(-1.97 to -1.61)
United Kingdom	60 to 64	-1.6<br>(-1.74 to -1.46)
United Kingdom	65 to 69	-1.25<br>(-1.37 to -1.13)
United Kingdom	70 to 74	-0.86<br>(-0.98 to -0.75)
United Kingdom	75 to 79	-0.46<br>(-0.59 to -0.34)
United Kingdom	80 to 84	-0.06<br>(-0.21 to 0.09)
United Kingdom	85 to 89	0.27<br>(0.08 to 0.46)
United Kingdom	90 to 94	0.52<br>(0.21 to 0.84)
United Kingdom	95+	0.73<br>(0.06 to 1.41)
United States	55 to 59	0.94<br>(0.73 to 1.14)
United States	60 to 64	0.9<br>(0.74 to 1.06)
United States	65 to 69	0.84<br>(0.7 to 0.98)
United States	70 to 74	0.77<br>(0.63 to 0.91)
United States	75 to 79	0.75<br>(0.6 to 0.9)
United States	80 to 84	0.8<br>(0.61 to 0.98)
United States	85 to 89	0.88<br>(0.63 to 1.13)

United States	90 to 94	0.99<br>(0.58 to 1.39)
United States	95+	1.11<br>(0.3 to 1.93)

CRDs<br>Location	Age	Prevalence rate (per 100,000 population)
Germany	55 to 59	10197.45<br>(9712.55 to 10706.55)
Germany	60 to 64	11447.83<br>(10912.44 to 12009.49)
Germany	65 to 69	12797.66<br>(12115.71 to 13518)
Germany	70 to 74	14287.29<br>(13513.47 to 15105.43)
Germany	75 to 79	16324.15<br>(15421.52 to 17279.6)
Germany	80 to 84	19810.57<br>(18681.36 to 21008.04)
Germany	85 to 89	25391.51<br>(23843.44 to 27040.08)
Germany	90 to 94	32280.53<br>(30016.71 to 34715.08)
Germany	95+	40146.01<br>(36417.96 to 44255.71)
Brazil	55 to 59	7053.78<br>(6981.7 to 7126.61)
Brazil	60 to 64	8439.51<br>(8355.24 to 8524.63)
Brazil	65 to 69	10542.88<br>(10415.98 to 10671.32)
Brazil	70 to 74	13433.01<br>(13266.29 to 13601.82)
Brazil	75 to 79	16888.99<br>(16670.78 to 17110.05)
Brazil	80 to 84	21016.01<br>(20727.92 to 21308.11)
Brazil	85 to 89	26147.53<br>(25751.12 to 26550.03)
Brazil	90 to 94	31550.56<br>(30976.61 to 32135.15)
Brazil	95+	36850.5<br>(35964.46 to 37758.37)
Afghanistan	55 to 59	14433.97<br>(14288.09 to 14581.34)
Afghanistan	60 to 64	16296.51<br>(16134.49 to 16460.15)
Afghanistan	65 to 69	18085.88<br>(17903.06 to 18270.57)
Afghanistan	70 to 74	19995.8<br>(19760.78 to 20233.61)
Afghanistan	75 to 79	21940.21<br>(21656.53 to 22227.6)
Afghanistan	80 to 84	24238.78<br>(23865.94 to 24617.45)
Afghanistan	85 to 89	27199.38<br>(26627.1 to 27783.96)
Afghanistan	90 to 94	30348.62<br>(29261.91 to 31475.69)
Afghanistan	95+	34105.77<br>(31651.08 to 36750.84)
Japan	55 to 59	5221.57<br>(4874.56 to 5593.28)
Japan	60 to 64	5751.38<br>(5372.43 to 6157.05)

Japan	65 to 69	6418.85<br>(5962.5 to 6910.13)
Japan	70 to 74	7161.76<br>(6650.4 to 7712.45)
Japan	75 to 79	7918.32<br>(7347.64 to 8533.32)
Japan	80 to 84	8859.95<br>(8211.1 to 9560.08)
Japan	85 to 89	10151.17<br>(9384.35 to 10980.64)
Japan	90 to 94	11521.4<br>(10591.75 to 12532.65)
Japan	95+	12887.75<br>(11705.2 to 14189.78)
Saudi Arabia	55 to 59	6714.64<br>(6651 to 6778.89)
Saudi Arabia	60 to 64	8562.5<br>(8480.89 to 8644.89)
Saudi Arabia	65 to 69	10868<br>(10735 to 11002.64)
Saudi Arabia	70 to 74	13839.22<br>(13655.91 to 14025)
Saudi Arabia	75 to 79	17433.14<br>(17174.33 to 17695.85)
Saudi Arabia	80 to 84	22219.36<br>(21846.05 to 22599.05)
Saudi Arabia	85 to 89	29231.07<br>(28639.17 to 29835.2)
Saudi Arabia	90 to 94	38161.37<br>(37071.66 to 39283.11)
Saudi Arabia	95+	48952.1<br>(46380.08 to 51666.75)
China	55 to 59	5876.49<br>(5755.25 to 6000.28)
China	60 to 64	7599.16<br>(7447.13 to 7754.3)
China	65 to 69	10679.09<br>(10428.51 to 10935.69)
China	70 to 74	14498.32<br>(14150.67 to 14854.51)
China	75 to 79	18298.2<br>(17844.99 to 18762.93)
China	80 to 84	21633.83<br>(21061.42 to 22221.81)
China	85 to 89	24589.88<br>(23830.87 to 25373.06)
China	90 to 94	26375.55<br>(25212.26 to 27592.52)
China	95+	28666<br>(26206.48 to 31356.35)
Burundi	55 to 59	8429.92<br>(8331.72 to 8529.28)
Burundi	60 to 64	9917.94<br>(9803.47 to 10033.74)
Burundi	65 to 69	10985.1<br>(10825.28 to 11147.28)
Burundi	70 to 74	11919.19<br>(11733.77 to 12107.54)
Burundi	75 to 79	13051.85<br>(12830.1 to 13277.43)
Burundi	80 to 84	14265.75<br>(13981.21 to 14556.07)
Burundi	85 to 89	15011.94<br>(14606.07 to 15429.09)
Burundi	90 to 94	15012.62<br>(14321.67 to 15736.92)

Burundi	95+	14611.33 (13219.73 to 16149.41)
India	55 to 59	9514.51 (9466.43 to 9562.84)
India	60 to 64	12246.12 (12186.33 to 12306.21)
India	65 to 69	16844.49 (16745.85 to 16943.72)
India	70 to 74	22085.27 (21950.92 to 22220.45)
India	75 to 79	27368.8 (27192.57 to 27546.16)
India	80 to 84	32015.11 (31787.18 to 32244.67)
India	85 to 89	37336.79 (37015.78 to 37660.59)
India	90 to 94	43035.09 (42512.92 to 43563.68)
India	95+	49940.03 (48890.37 to 51012.23)
Russia	55 to 59	7044.81 (6942.46 to 7148.67)
Russia	60 to 64	7663.96 (7553.52 to 7776.01)
Russia	65 to 69	8148.97 (8014.47 to 8285.72)
Russia	70 to 74	8624.13 (8478.35 to 8772.41)
Russia	75 to 79	9154.26 (8992.94 to 9318.47)
Russia	80 to 84	9948.7 (9761.74 to 10139.25)
Russia	85 to 89	11071.93 (10830.02 to 11319.25)
Russia	90 to 94	12415.8 (12048.04 to 12794.79)
Russia	95+	13925.15 (13135.37 to 14762.43)
United Kingdom	55 to 59	15355.75 (14802.47 to 15929.7)
United Kingdom	60 to 64	16085.37 (15510.97 to 16681.05)
United Kingdom	65 to 69	17731.66 (17007.8 to 18486.34)
United Kingdom	70 to 74	20101.37 (19268.32 to 20970.43)
United Kingdom	75 to 79	23096.81 (22116.48 to 24120.59)
United Kingdom	80 to 84	26800.19 (25620.27 to 28034.45)
United Kingdom	85 to 89	32191.26 (30674.05 to 33783.53)
United Kingdom	90 to 94	38306.59 (36217.48 to 40516.2)
United Kingdom	95+	45133.74 (41795.16 to 48739.01)
United States	55 to 59	15439.6 (14903.76 to 15994.69)
United States	60 to 64	19888.5 (19213.96 to 20586.73)
United States	65 to 69	27911.43 (26756.92 to 29115.75)
United States	70 to 74	36999.76 (35412.29 to 38658.39)
United States	75 to 79	46310.47 (44246.98 to 48470.18)

United States	80 to 84	54198.1 (51655.37 to 56866)
United States	85 to 89	60378.55 (57273.33 to 63652.14)
United States	90 to 94	64577.4 (60562.59 to 68858.35)
United States	95+	68164.64 (61953.2 to 74998.85)

CRDs Location	Period	Prevalence rate ratio
Germany	1992 to 1996	1.28 (1.23 to 1.32)
Germany	1997 to 2001	1.09 (1.05 to 1.13)
Germany	2002 to 2006	1.09 (1.05 to 1.13)
Germany	2007 to 2011	1.06 (1.03 to 1.1)
Germany	2012 to 2016	1 (1 to 1)
Germany	2017 to 2021	1.07 (1.04 to 1.11)
Brazil	1992 to 1996	1.14 (1.13 to 1.15)
Brazil	1997 to 2001	1.13 (1.12 to 1.14)
Brazil	2002 to 2006	1.09 (1.08 to 1.1)
Brazil	2007 to 2011	1.02 (1.01 to 1.03)
Brazil	2012 to 2016	1 (1 to 1)
Brazil	2017 to 2021	1.03 (1.02 to 1.03)
Afghanistan	1992 to 1996	1.06 (1.05 to 1.07)
Afghanistan	1997 to 2001	1.04 (1.03 to 1.05)
Afghanistan	2002 to 2006	1.02 (1.01 to 1.03)
Afghanistan	2007 to 2011	1 (1 to 1)
Afghanistan	2012 to 2016	0.99 (0.98 to 1)
Afghanistan	2017 to 2021	1.02 (1.01 to 1.03)
Japan	1992 to 1996	1.91 (1.84 to 1.98)
Japan	1997 to 2001	1.53 (1.48 to 1.59)
Japan	2002 to 2006	1.21 (1.17 to 1.25)
Japan	2007 to 2011	1.07 (1.04 to 1.11)
Japan	2012 to 2016	1 (1 to 1)
Japan	2017 to 2021	0.95 (0.91 to 0.98)
Saudi Arabia	1992 to 1996	0.86 (0.85 to 0.88)
Saudi Arabia	1997 to 2001	0.87 (0.86 to 0.88)
Saudi Arabia	2002 to 2006	0.91 (0.9 to 0.92)

Saudi Arabia	2007 to 2011	0.96<br>(0.95 to 0.97)
Saudi Arabia	2012 to 2016	1<br>(1 to 1)
Saudi Arabia	2017 to 2021	1.06<br>(1.05 to 1.07)
China	1992 to 1996	1.1<br>(1.06 to 1.13)
China	1997 to 2001	1.05<br>(1.03 to 1.08)
China	2002 to 2006	1.01<br>(0.99 to 1.03)
China	2007 to 2011	1<br>(0.98 to 1.01)
China	2012 to 2016	1<br>(1 to 1)
China	2017 to 2021	0.99<br>(0.98 to 1.01)
Burundi	1992 to 1996	1.09<br>(1.07 to 1.12)
Burundi	1997 to 2001	1.06<br>(1.04 to 1.08)
Burundi	2002 to 2006	1.02<br>(1 to 1.04)
Burundi	2007 to 2011	1<br>(0.98 to 1.01)
Burundi	2012 to 2016	1<br>(1 to 1)
Burundi	2017 to 2021	1.07<br>(1.05 to 1.08)
India	1992 to 1996	1.01<br>(1 to 1.01)
India	1997 to 2001	0.98<br>(0.98 to 0.99)
India	2002 to 2006	0.99<br>(0.98 to 0.99)
India	2007 to 2011	1<br>(0.99 to 1)
India	2012 to 2016	1<br>(1 to 1)
India	2017 to 2021	1.01<br>(1.01 to 1.01)
Russia	1992 to 1996	1.46<br>(1.44 to 1.48)
Russia	1997 to 2001	1.3<br>(1.28 to 1.31)
Russia	2002 to 2006	1.15<br>(1.14 to 1.17)
Russia	2007 to 2011	1.06<br>(1.05 to 1.07)
Russia	2012 to 2016	1<br>(1 to 1)
Russia	2017 to 2021	1.04<br>(1.02 to 1.05)
United Kingdom	1992 to 1996	1.19<br>(1.16 to 1.22)
United Kingdom	1997 to 2001	1.05<br>(1.02 to 1.08)
United Kingdom	2002 to 2006	1.02<br>(0.99 to 1.05)
United Kingdom	2007 to 2011	1.02<br>(1 to 1.05)
United Kingdom	2012 to 2016	1<br>(1 to 1)
United Kingdom	2017 to 2021	1.03<br>(1 to 1.05)

United States	1992 to 1996	0.85 (0.82 to 0.87)
United States	1997 to 2001	0.82 (0.8 to 0.85)
United States	2002 to 2006	0.88 (0.85 to 0.9)
United States	2007 to 2011	0.95 (0.93 to 0.98)
United States	2012 to 2016	1 (1 to 1)
United States	2017 to 2021	1 (0.98 to 1.03)

CRDs Location	Cohort	Prevalence rate ratio
Germany	1897 to 1901	1.35 (1.03 to 1.78)
Germany	1902 to 1906	1.42 (1.25 to 1.62)
Germany	1907 to 1911	1.46 (1.34 to 1.59)
Germany	1912 to 1916	1.5 (1.4 to 1.61)
Germany	1917 to 1921	1.52 (1.42 to 1.63)
Germany	1922 to 1926	1.51 (1.42 to 1.61)
Germany	1927 to 1931	1.48 (1.4 to 1.57)
Germany	1932 to 1936	1.43 (1.35 to 1.51)
Germany	1937 to 1941	1.34 (1.27 to 1.42)
Germany	1942 to 1946	1.26 (1.2 to 1.33)
Germany	1947 to 1951	1.19 (1.13 to 1.26)
Germany	1952 to 1956	1.11 (1.05 to 1.18)
Germany	1957 to 1961	1 (1 to 1)
Germany	1962 to 1966	0.9 (0.83 to 0.97)
Brazil	1897 to 1901	1.37 (1.24 to 1.52)
Brazil	1902 to 1906	1.37 (1.31 to 1.43)
Brazil	1907 to 1911	1.37 (1.34 to 1.41)
Brazil	1912 to 1916	1.37 (1.35 to 1.4)
Brazil	1917 to 1921	1.38 (1.35 to 1.4)
Brazil	1922 to 1926	1.37 (1.35 to 1.39)
Brazil	1927 to 1931	1.36 (1.34 to 1.38)
Brazil	1932 to 1936	1.33 (1.31 to 1.34)
Brazil	1937 to 1941	1.28 (1.26 to 1.29)
Brazil	1942 to 1946	1.22 (1.21 to 1.24)
Brazil	1947 to 1951	1.16 (1.14 to 1.17)

Brazil	1952 to 1956	1.08<br>(1.06 to 1.09)
Brazil	1957 to 1961	1<br>(1 to 1)
Brazil	1962 to 1966	0.92<br>(0.91 to 0.94)
Afghanistan	1897 to 1901	1<br>(0.81 to 1.24)
Afghanistan	1902 to 1906	1.04<br>(0.96 to 1.13)
Afghanistan	1907 to 1911	1.09<br>(1.04 to 1.14)
Afghanistan	1912 to 1916	1.13<br>(1.1 to 1.16)
Afghanistan	1917 to 1921	1.17<br>(1.14 to 1.19)
Afghanistan	1922 to 1926	1.18<br>(1.16 to 1.2)
Afghanistan	1927 to 1931	1.17<br>(1.16 to 1.19)
Afghanistan	1932 to 1936	1.15<br>(1.14 to 1.17)
Afghanistan	1937 to 1941	1.13<br>(1.11 to 1.14)
Afghanistan	1942 to 1946	1.09<br>(1.08 to 1.1)
Afghanistan	1947 to 1951	1.04<br>(1.03 to 1.06)
Afghanistan	1952 to 1956	1<br>(1 to 1)
Afghanistan	1957 to 1961	0.95<br>(0.93 to 0.96)
Afghanistan	1962 to 1966	0.88<br>(0.86 to 0.9)
Japan	1897 to 1901	4.2<br>(3.17 to 5.56)
Japan	1902 to 1906	4.25<br>(3.69 to 4.89)
Japan	1907 to 1911	4.26<br>(3.86 to 4.71)
Japan	1912 to 1916	4.17<br>(3.82 to 4.54)
Japan	1917 to 1921	3.91<br>(3.61 to 4.23)
Japan	1922 to 1926	3.49<br>(3.23 to 3.77)
Japan	1927 to 1931	3.03<br>(2.81 to 3.27)
Japan	1932 to 1936	2.54<br>(2.36 to 2.73)
Japan	1937 to 1941	2.1<br>(1.96 to 2.26)
Japan	1942 to 1946	1.77<br>(1.64 to 1.9)
Japan	1947 to 1951	1.49<br>(1.38 to 1.6)
Japan	1952 to 1956	1.24<br>(1.15 to 1.34)
Japan	1957 to 1961	1<br>(1 to 1)
Japan	1962 to 1966	0.81<br>(0.71 to 0.91)
Saudi Arabia	1897 to 1901	0.56<br>(0.49 to 0.65)
Saudi Arabia	1902 to 1906	0.62<br>(0.58 to 0.67)

Saudi Arabia	1907 to 1911	0.69<br>(0.66 to 0.72)
Saudi Arabia	1912 to 1916	0.75<br>(0.73 to 0.78)
Saudi Arabia	1917 to 1921	0.82<br>(0.8 to 0.84)
Saudi Arabia	1922 to 1926	0.88<br>(0.86 to 0.89)
Saudi Arabia	1927 to 1931	0.92<br>(0.91 to 0.94)
Saudi Arabia	1932 to 1936	0.96<br>(0.94 to 0.97)
Saudi Arabia	1937 to 1941	0.98<br>(0.97 to 0.99)
Saudi Arabia	1942 to 1946	1<br>(0.99 to 1.01)
Saudi Arabia	1947 to 1951	1.01<br>(1 to 1.02)
Saudi Arabia	1952 to 1956	1.01<br>(1 to 1.02)
Saudi Arabia	1957 to 1961	1<br>(1 to 1)
Saudi Arabia	1962 to 1966	0.99<br>(0.97 to 1)
China	1897 to 1901	1.08<br>(0.67 to 1.76)
China	1902 to 1906	1.19<br>(1.02 to 1.39)
China	1907 to 1911	1.29<br>(1.2 to 1.38)
China	1912 to 1916	1.36<br>(1.3 to 1.42)
China	1917 to 1921	1.41<br>(1.37 to 1.46)
China	1922 to 1926	1.44<br>(1.4 to 1.48)
China	1927 to 1931	1.42<br>(1.38 to 1.46)
China	1932 to 1936	1.38<br>(1.34 to 1.41)
China	1937 to 1941	1.32<br>(1.29 to 1.35)
China	1942 to 1946	1.25<br>(1.22 to 1.28)
China	1947 to 1951	1.16<br>(1.14 to 1.19)
China	1952 to 1956	1.08<br>(1.05 to 1.1)
China	1957 to 1961	1<br>(1 to 1)
China	1962 to 1966	0.93<br>(0.9 to 0.97)
Burundi	1897 to 1901	1.05<br>(0.75 to 1.47)
Burundi	1902 to 1906	1.13<br>(1 to 1.28)
Burundi	1907 to 1911	1.15<br>(1.09 to 1.22)
Burundi	1912 to 1916	1.19<br>(1.15 to 1.23)
Burundi	1917 to 1921	1.22<br>(1.19 to 1.25)
Burundi	1922 to 1926	1.23<br>(1.21 to 1.26)
Burundi	1927 to 1931	1.23<br>(1.21 to 1.26)

Burundi	1932 to 1936	1.23<br>(1.21 to 1.25)
Burundi	1937 to 1941	1.2<br>(1.19 to 1.22)
Burundi	1942 to 1946	1.17<br>(1.15 to 1.19)
Burundi	1947 to 1951	1.12<br>(1.1 to 1.14)
Burundi	1952 to 1956	1.06<br>(1.05 to 1.08)
Burundi	1957 to 1961	1<br>(1 to 1)
Burundi	1962 to 1966	0.94<br>(0.92 to 0.95)
India	1897 to 1901	0.94<br>(0.86 to 1.02)
India	1902 to 1906	0.97<br>(0.93 to 1)
India	1907 to 1911	1<br>(0.98 to 1.01)
India	1912 to 1916	1.03<br>(1.01 to 1.04)
India	1917 to 1921	1.05<br>(1.04 to 1.06)
India	1922 to 1926	1.06<br>(1.05 to 1.07)
India	1927 to 1931	1.07<br>(1.06 to 1.08)
India	1932 to 1936	1.07<br>(1.07 to 1.08)
India	1937 to 1941	1.07<br>(1.06 to 1.08)
India	1942 to 1946	1.05<br>(1.05 to 1.06)
India	1947 to 1951	1.04<br>(1.03 to 1.04)
India	1952 to 1956	1.02<br>(1.01 to 1.02)
India	1957 to 1961	1<br>(1 to 1)
India	1962 to 1966	0.99<br>(0.98 to 1)
Russia	1897 to 1901	2.25<br>(1.9 to 2.66)
Russia	1902 to 1906	2.28<br>(2.13 to 2.44)
Russia	1907 to 1911	2.3<br>(2.21 to 2.38)
Russia	1912 to 1916	2.27<br>(2.21 to 2.33)
Russia	1917 to 1921	2.24<br>(2.19 to 2.29)
Russia	1922 to 1926	2.16<br>(2.12 to 2.2)
Russia	1927 to 1931	2.07<br>(2.04 to 2.11)
Russia	1932 to 1936	1.95<br>(1.92 to 1.98)
Russia	1937 to 1941	1.76<br>(1.73 to 1.79)
Russia	1942 to 1946	1.58<br>(1.55 to 1.6)
Russia	1947 to 1951	1.36<br>(1.33 to 1.38)
Russia	1952 to 1956	1.18<br>(1.16 to 1.2)

Russia	1957 to 1961	1<br>(1 to 1)
Russia	1962 to 1966	0.84<br>(0.82 to 0.86)
United Kingdom	1897 to 1901	1.27<br>(1.01 to 1.6)
United Kingdom	1902 to 1906	1.34<br>(1.21 to 1.49)
United Kingdom	1907 to 1911	1.4<br>(1.31 to 1.5)
United Kingdom	1912 to 1916	1.46<br>(1.38 to 1.54)
United Kingdom	1917 to 1921	1.5<br>(1.43 to 1.58)
United Kingdom	1922 to 1926	1.52<br>(1.46 to 1.6)
United Kingdom	1927 to 1931	1.53<br>(1.46 to 1.59)
United Kingdom	1932 to 1936	1.49<br>(1.43 to 1.56)
United Kingdom	1937 to 1941	1.43<br>(1.37 to 1.49)
United Kingdom	1942 to 1946	1.33<br>(1.28 to 1.39)
United Kingdom	1947 to 1951	1.23<br>(1.18 to 1.28)
United Kingdom	1952 to 1956	1.12<br>(1.07 to 1.17)
United Kingdom	1957 to 1961	1<br>(1 to 1)
United Kingdom	1962 to 1966	0.92<br>(0.87 to 0.98)
United States	1897 to 1901	0.57<br>(0.43 to 0.74)
United States	1902 to 1906	0.61<br>(0.53 to 0.69)
United States	1907 to 1911	0.64<br>(0.59 to 0.7)
United States	1912 to 1916	0.68<br>(0.64 to 0.72)
United States	1917 to 1921	0.72<br>(0.68 to 0.76)
United States	1922 to 1926	0.75<br>(0.71 to 0.79)
United States	1927 to 1931	0.77<br>(0.74 to 0.81)
United States	1932 to 1936	0.8<br>(0.76 to 0.84)
United States	1937 to 1941	0.83<br>(0.8 to 0.87)
United States	1942 to 1946	0.87<br>(0.83 to 0.9)
United States	1947 to 1951	0.91<br>(0.87 to 0.94)
United States	1952 to 1956	0.95<br>(0.91 to 0.99)
United States	1957 to 1961	1<br>(1 to 1)
United States	1962 to 1966	1.05<br>(0.99 to 1.11)

Table S13 APC Model Analysis Results of COPD Prevalence Among Adults Aged 55 and Above in 11 Example Countries

COPD<br>Location	Age	Local drift (%/year)
Germany	55 to 59	-0.3<br>(-0.39 to -0.21)
Germany	60 to 64	-0.18<br>(-0.24 to -0.11)
Germany	65 to 69	-0.05<br>(-0.11 to 0.01)
Germany	70 to 74	0.06<br>(0.01 to 0.12)
Germany	75 to 79	0.15<br>(0.09 to 0.2)
Germany	80 to 84	0.23<br>(0.18 to 0.29)
Germany	85 to 89	0.31<br>(0.24 to 0.38)
Germany	90 to 94	0.37<br>(0.26 to 0.48)
Germany	95+	0.41<br>(0.17 to 0.64)
Brazil	55 to 59	-0.38<br>(-0.42 to -0.33)
Brazil	60 to 64	-0.26<br>(-0.29 to -0.22)
Brazil	65 to 69	-0.18<br>(-0.21 to -0.15)
Brazil	70 to 74	-0.12<br>(-0.16 to -0.09)
Brazil	75 to 79	-0.08<br>(-0.11 to -0.04)
Brazil	80 to 84	-0.02<br>(-0.06 to 0.02)
Brazil	85 to 89	0.02<br>(-0.03 to 0.08)
Brazil	90 to 94	0.06<br>(-0.03 to 0.15)
Brazil	95+	0.08<br>(-0.12 to 0.29)
Afghanistan	55 to 59	-0.17<br>(-0.23 to -0.11)
Afghanistan	60 to 64	-0.02<br>(-0.06 to 0.03)
Afghanistan	65 to 69	0.14<br>(0.1 to 0.17)
Afghanistan	70 to 74	0.28<br>(0.24 to 0.32)
Afghanistan	75 to 79	0.42<br>(0.37 to 0.47)
Afghanistan	80 to 84	0.57<br>(0.51 to 0.63)
Afghanistan	85 to 89	0.74<br>(0.64 to 0.83)
Afghanistan	90 to 94	0.9<br>(0.73 to 1.08)
Afghanistan	95+	1.04<br>(0.6 to 1.48)
Japan	55 to 59	-1.21<br>(-1.37 to -1.04)
Japan	60 to 64	-1.07<br>(-1.18 to -0.96)
Japan	65 to 69	-0.96<br>(-1.05 to -0.88)
Japan	70 to 74	-0.82<br>(-0.89 to -0.75)

Japan	75 to 79	-0.67<br>(-0.74 to -0.6)
Japan	80 to 84	-0.52<br>(-0.59 to -0.45)
Japan	85 to 89	-0.38<br>(-0.47 to -0.29)
Japan	90 to 94	-0.24<br>(-0.38 to -0.1)
Japan	95+	-0.1<br>(-0.4 to 0.21)
Saudi Arabia	55 to 59	0.76<br>(0.71 to 0.81)
Saudi Arabia	60 to 64	0.88<br>(0.83 to 0.92)
Saudi Arabia	65 to 69	1.02<br>(0.98 to 1.06)
Saudi Arabia	70 to 74	1.18<br>(1.13 to 1.23)
Saudi Arabia	75 to 79	1.35<br>(1.29 to 1.4)
Saudi Arabia	80 to 84	1.53<br>(1.46 to 1.6)
Saudi Arabia	85 to 89	1.73<br>(1.62 to 1.83)
Saudi Arabia	90 to 94	1.9<br>(1.72 to 2.07)
Saudi Arabia	95+	2.04<br>(1.69 to 2.38)
China	55 to 59	-0.98<br>(-1.09 to -0.87)
China	60 to 64	-0.78<br>(-0.86 to -0.7)
China	65 to 69	-0.56<br>(-0.63 to -0.49)
China	70 to 74	-0.3<br>(-0.38 to -0.23)
China	75 to 79	-0.02<br>(-0.11 to 0.06)
China	80 to 84	0.28<br>(0.17 to 0.4)
China	85 to 89	0.64<br>(0.45 to 0.84)
China	90 to 94	1.08<br>(0.64 to 1.52)
China	95+	1.53<br>(0.16 to 2.91)
Burundi	55 to 59	0.04<br>(-0.05 to 0.14)
Burundi	60 to 64	0.12<br>(0.05 to 0.2)
Burundi	65 to 69	0.2<br>(0.13 to 0.28)
Burundi	70 to 74	0.29<br>(0.2 to 0.37)
Burundi	75 to 79	0.37<br>(0.27 to 0.46)
Burundi	80 to 84	0.46<br>(0.33 to 0.59)
Burundi	85 to 89	0.57<br>(0.36 to 0.79)
Burundi	90 to 94	0.65<br>(0.22 to 1.09)
Burundi	95+	0.84<br>(-0.31 to 2.01)
India	55 to 59	-0.07<br>(-0.09 to -0.05)

India	60 to 64	0<br>(-0.02 to 0.01)
India	65 to 69	0.08<br>(0.06 to 0.09)
India	70 to 74	0.15<br>(0.14 to 0.17)
India	75 to 79	0.24<br>(0.22 to 0.26)
India	80 to 84	0.35<br>(0.33 to 0.38)
India	85 to 89	0.48<br>(0.44 to 0.51)
India	90 to 94	0.59<br>(0.53 to 0.65)
India	95+	0.65<br>(0.5 to 0.8)
Russia	55 to 59	-0.32<br>(-0.45 to -0.18)
Russia	60 to 64	-0.3<br>(-0.4 to -0.2)
Russia	65 to 69	-0.32<br>(-0.41 to -0.23)
Russia	70 to 74	-0.33<br>(-0.43 to -0.24)
Russia	75 to 79	-0.38<br>(-0.48 to -0.28)
Russia	80 to 84	-0.41<br>(-0.52 to -0.29)
Russia	85 to 89	-0.41<br>(-0.57 to -0.25)
Russia	90 to 94	-0.35<br>(-0.64 to -0.05)
Russia	95+	-0.22<br>(-0.94 to 0.51)
United Kingdom	55 to 59	-0.01<br>(-0.07 to 0.04)
United Kingdom	60 to 64	0.04<br>(0 to 0.08)
United Kingdom	65 to 69	0.12<br>(0.09 to 0.15)
United Kingdom	70 to 74	0.21<br>(0.18 to 0.24)
United Kingdom	75 to 79	0.28<br>(0.25 to 0.31)
United Kingdom	80 to 84	0.34<br>(0.3 to 0.37)
United Kingdom	85 to 89	0.36<br>(0.32 to 0.4)
United Kingdom	90 to 94	0.36<br>(0.3 to 0.43)
United Kingdom	95+	0.37<br>(0.23 to 0.5)
United States	55 to 59	0.23<br>(0.08 to 0.38)
United States	60 to 64	0.31<br>(0.21 to 0.42)
United States	65 to 69	0.41<br>(0.32 to 0.5)
United States	70 to 74	0.49<br>(0.41 to 0.58)
United States	75 to 79	0.58<br>(0.5 to 0.67)
United States	80 to 84	0.67<br>(0.57 to 0.77)
United States	85 to 89	0.74<br>(0.6 to 0.88)

United States	90 to 94	0.79<br>(0.56 to 1.01)
United States	95+	0.82<br>(0.37 to 1.27)

COPD<br>Location	Age	Prevalence rate (per 100,000 population)
Germany	55 to 59	5309.8<br>(5212.11 to 5409.32)
Germany	60 to 64	7118.79<br>(6994.29 to 7245.5)
Germany	65 to 69	9609.82<br>(9415.73 to 9807.91)
Germany	70 to 74	12969.27<br>(12705.42 to 13238.6)
Germany	75 to 79	17569.5<br>(17207.9 to 17938.7)
Germany	80 to 84	24448.58<br>(23934.02 to 24974.21)
Germany	85 to 89	34168.87<br>(33415.79 to 34938.93)
Germany	90 to 94	45487.15<br>(44382.08 to 46619.73)
Germany	95+	58053.44<br>(56298.16 to 59863.45)
Brazil	55 to 59	4949.98<br>(4910.34 to 4989.95)
Brazil	60 to 64	6712.07<br>(6660.52 to 6764.02)
Brazil	65 to 69	9351.94<br>(9264.68 to 9440.02)
Brazil	70 to 74	13116.87<br>(12990.87 to 13244.1)
Brazil	75 to 79	17957.05<br>(17778.23 to 18137.67)
Brazil	80 to 84	23800.24<br>(23550.89 to 24052.24)
Brazil	85 to 89	30703.7<br>(30353.34 to 31058.12)
Brazil	90 to 94	37771.69<br>(37267.31 to 38282.89)
Brazil	95+	44597.78<br>(43832 to 45376.93)
Afghanistan	55 to 59	5737.49<br>(5666.13 to 5809.75)
Afghanistan	60 to 64	7360.11<br>(7269.58 to 7451.76)
Afghanistan	65 to 69	9415.09<br>(9287.06 to 9544.88)
Afghanistan	70 to 74	12222.73<br>(12053.27 to 12394.56)
Afghanistan	75 to 79	16023.93<br>(15793.9 to 16257.31)
Afghanistan	80 to 84	20758.28<br>(20437.52 to 21084.06)
Afghanistan	85 to 89	26018.29<br>(25541.68 to 26503.79)
Afghanistan	90 to 94	31136.65<br>(30299.88 to 31996.54)
Afghanistan	95+	36799.07<br>(34956.28 to 38739)
Japan	55 to 59	2242.08<br>(2172.64 to 2313.74)
Japan	60 to 64	3207.77<br>(3111.33 to 3307.2)

Japan	65 to 69	4524.87<br>(4375.67 to 4679.16)
Japan	70 to 74	6424.34<br>(6212.11 to 6643.83)
Japan	75 to 79	9165.75<br>(8860.63 to 9481.37)
Japan	80 to 84	12985.05<br>(12547.2 to 13438.17)
Japan	85 to 89	17754.41<br>(17141.8 to 18388.91)
Japan	90 to 94	22686.78<br>(21867.65 to 23536.59)
Japan	95+	27568.33<br>(26479.8 to 28701.6)
Saudi Arabia	55 to 59	3985.18<br>(3950.96 to 4019.7)
Saudi Arabia	60 to 64	5689.74<br>(5641.52 to 5738.37)
Saudi Arabia	65 to 69	7867.06<br>(7780.74 to 7954.34)
Saudi Arabia	70 to 74	10932.86<br>(10803.65 to 11063.62)
Saudi Arabia	75 to 79	15360.4<br>(15160.59 to 15562.84)
Saudi Arabia	80 to 84	21753.12<br>(21441.24 to 22069.54)
Saudi Arabia	85 to 89	30795.4<br>(30279.83 to 31319.76)
Saudi Arabia	90 to 94	42185.57<br>(41229.9 to 43163.39)
Saudi Arabia	95+	56140.04<br>(53882.79 to 58491.85)
China	55 to 59	4744.93<br>(4648.7 to 4843.15)
China	60 to 64	6438.12<br>(6312.2 to 6566.56)
China	65 to 69	9471.63<br>(9253.39 to 9695.02)
China	70 to 74	13447.19<br>(13130.26 to 13771.77)
China	75 to 79	17834.74<br>(17400.9 to 18279.4)
China	80 to 84	22024.72<br>(21454.67 to 22609.92)
China	85 to 89	26032.65<br>(25255.65 to 26833.55)
China	90 to 94	28724.86<br>(27523.84 to 29978.3)
China	95+	32065.83<br>(29502.68 to 34851.66)
Burundi	55 to 59	4124.3<br>(4056.56 to 4193.16)
Burundi	60 to 64	5445.77<br>(5358.72 to 5534.24)
Burundi	65 to 69	6926.13<br>(6785.59 to 7069.58)
Burundi	70 to 74	8776.88<br>(8589.22 to 8968.65)
Burundi	75 to 79	11036.01<br>(10784.66 to 11293.22)
Burundi	80 to 84	13436.58<br>(13091.86 to 13790.38)
Burundi	85 to 89	15096.56<br>(14599.9 to 15610.12)
Burundi	90 to 94	15543.55<br>(14714.82 to 16418.95)

Burundi	95+	15324.81 (13672.24 to 17177.13)
India	55 to 59	6009.19 (5987.16 to 6031.3)
India	60 to 64	8143.54 (8114.96 to 8172.22)
India	65 to 69	12449.92 (12397.73 to 12502.33)
India	70 to 74	17771.53 (17694.6 to 17848.79)
India	75 to 79	23624.57 (23517.33 to 23732.3)
India	80 to 84	29092.57 (28949.06 to 29236.79)
India	85 to 89	35390.79 (35186.54 to 35596.22)
India	90 to 94	42109.41 (41780.71 to 42440.69)
India	95+	50347.69 (49690.71 to 51013.35)
Russia	55 to 59	4724.3 (4607.55 to 4844)
Russia	60 to 64	6248.31 (6098.12 to 6402.2)
Russia	65 to 69	8217.93 (7985.55 to 8457.07)
Russia	70 to 74	10642.05 (10335.92 to 10957.25)
Russia	75 to 79	13362.72 (12966.75 to 13770.78)
Russia	80 to 84	16413.11 (15902.36 to 16940.27)
Russia	85 to 89	19632.17 (18945 to 20344.26)
Russia	90 to 94	22935.26 (21899.61 to 24019.89)
Russia	95+	26184.17 (24040.78 to 28518.66)
United Kingdom	55 to 59	6267.12 (6200.16 to 6334.81)
United Kingdom	60 to 64	8654.72 (8566.12 to 8744.24)
United Kingdom	65 to 69	13040.17 (12887.01 to 13195.15)
United Kingdom	70 to 74	19077.37 (18851.76 to 19305.69)
United Kingdom	75 to 79	26384.79 (26067.91 to 26705.53)
United Kingdom	80 to 84	34628.67 (34203.42 to 35059.21)
United Kingdom	85 to 89	44582.63 (44013.04 to 45159.6)
United Kingdom	90 to 94	54701.67 (53936.85 to 55477.35)
United Kingdom	95+	65228.17 (64091.4 to 66385.1)
United States	55 to 59	6873.32 (6695.43 to 7055.93)
United States	60 to 64	9895.09 (9651.66 to 10144.66)
United States	65 to 69	16367.34 (15890.36 to 16858.64)
United States	70 to 74	23939.86 (23226.11 to 24675.56)
United States	75 to 79	32214.93 (31229.41 to 33231.55)

United States	80 to 84	39317.86<br>(38072.83 to 40603.6)
United States	85 to 89	44383.59<br>(42886.6 to 45932.82)
United States	90 to 94	47111.18<br>(45278.34 to 49018.21)
United States	95+	48604.92<br>(45975.46 to 51384.76)

COPD<br>Location	Period	Prevalence rate ratio
Germany	1992 to 1996	1.04<br>(1.03 to 1.05)
Germany	1997 to 2001	1.02<br>(1.01 to 1.03)
Germany	2002 to 2006	1.01<br>(1 to 1.02)
Germany	2007 to 2011	1.01<br>(1 to 1.02)
Germany	2012 to 2016	1<br>(1 to 1)
Germany	2017 to 2021	1.1<br>(1.09 to 1.11)
Brazil	1992 to 1996	1.03<br>(1.02 to 1.03)
Brazil	1997 to 2001	1.03<br>(1.03 to 1.04)
Brazil	2002 to 2006	1.03<br>(1.02 to 1.04)
Brazil	2007 to 2011	1.01<br>(1 to 1.01)
Brazil	2012 to 2016	1<br>(1 to 1)
Brazil	2017 to 2021	1.02<br>(1.01 to 1.02)
Afghanistan	1992 to 1996	0.94<br>(0.92 to 0.95)
Afghanistan	1997 to 2001	0.95<br>(0.94 to 0.96)
Afghanistan	2002 to 2006	0.96<br>(0.95 to 0.97)
Afghanistan	2007 to 2011	0.97<br>(0.96 to 0.98)
Afghanistan	2012 to 2016	1<br>(1 to 1)
Afghanistan	2017 to 2021	1.05<br>(1.04 to 1.06)
Japan	1992 to 1996	1.12<br>(1.1 to 1.13)
Japan	1997 to 2001	1.07<br>(1.06 to 1.09)
Japan	2002 to 2006	1.04<br>(1.03 to 1.05)
Japan	2007 to 2011	1.01<br>(1 to 1.02)
Japan	2012 to 2016	1<br>(1 to 1)
Japan	2017 to 2021	0.93<br>(0.92 to 0.94)
Saudi Arabia	1992 to 1996	0.77<br>(0.76 to 0.78)
Saudi Arabia	1997 to 2001	0.8<br>(0.79 to 0.81)
Saudi Arabia	2002 to 2006	0.86<br>(0.85 to 0.87)

Saudi Arabia	2007 to 2011	0.92<br>(0.92 to 0.93)
Saudi Arabia	2012 to 2016	1<br>(1 to 1)
Saudi Arabia	2017 to 2021	1.07<br>(1.06 to 1.08)
China	1992 to 1996	0.99<br>(0.96 to 1.02)
China	1997 to 2001	0.97<br>(0.95 to 1)
China	2002 to 2006	0.97<br>(0.95 to 0.99)
China	2007 to 2011	0.97<br>(0.96 to 0.99)
China	2012 to 2016	1<br>(1 to 1)
China	2017 to 2021	1<br>(0.98 to 1.01)
Burundi	1992 to 1996	0.96<br>(0.93 to 0.99)
Burundi	1997 to 2001	0.97<br>(0.94 to 0.99)
Burundi	2002 to 2006	0.96<br>(0.95 to 0.99)
Burundi	2007 to 2011	0.97<br>(0.95 to 0.98)
Burundi	2012 to 2016	1<br>(1 to 1)
Burundi	2017 to 2021	1.08<br>(1.06 to 1.09)
India	1992 to 1996	0.95<br>(0.94 to 0.95)
India	1997 to 2001	0.96<br>(0.95 to 0.96)
India	2002 to 2006	0.97<br>(0.97 to 0.97)
India	2007 to 2011	0.99<br>(0.98 to 0.99)
India	2012 to 2016	1<br>(1 to 1)
India	2017 to 2021	1.01<br>(1.01 to 1.01)
Russia	1992 to 1996	1.09<br>(1.07 to 1.12)
Russia	1997 to 2001	1.07<br>(1.05 to 1.09)
Russia	2002 to 2006	1.07<br>(1.05 to 1.09)
Russia	2007 to 2011	1.03<br>(1.01 to 1.05)
Russia	2012 to 2016	1<br>(1 to 1)
Russia	2017 to 2021	1.01<br>(1 to 1.03)
United Kingdom	1992 to 1996	0.94<br>(0.94 to 0.95)
United Kingdom	1997 to 2001	0.96<br>(0.96 to 0.97)
United Kingdom	2002 to 2006	0.98<br>(0.98 to 0.99)
United Kingdom	2007 to 2011	0.99<br>(0.99 to 1)
United Kingdom	2012 to 2016	1<br>(1 to 1)
United Kingdom	2017 to 2021	1<br>(0.99 to 1)

United States	1992 to 1996	0.87<br>(0.85 to 0.88)
United States	1997 to 2001	0.91<br>(0.9 to 0.93)
United States	2002 to 2006	1<br>(0.98 to 1.01)
United States	2007 to 2011	1.01<br>(0.99 to 1.02)
United States	2012 to 2016	1<br>(1 to 1)
United States	2017 to 2021	1<br>(0.98 to 1.02)

COPD<br>Location	Cohort	Prevalence rate ratio
Germany	1897 to 1901	0.93<br>(0.86 to 1)
Germany	1902 to 1906	0.95<br>(0.91 to 0.99)
Germany	1907 to 1911	0.97<br>(0.94 to 1)
Germany	1912 to 1916	0.99<br>(0.97 to 1.02)
Germany	1917 to 1921	1.01<br>(0.99 to 1.04)
Germany	1922 to 1926	1.02<br>(1 to 1.05)
Germany	1927 to 1931	1.04<br>(1.02 to 1.06)
Germany	1932 to 1936	1.05<br>(1.03 to 1.07)
Germany	1937 to 1941	1.05<br>(1.03 to 1.07)
Germany	1942 to 1946	1.05<br>(1.03 to 1.07)
Germany	1947 to 1951	1.04<br>(1.02 to 1.06)
Germany	1952 to 1956	1.03<br>(1 to 1.05)
Germany	1957 to 1961	1<br>(1 to 1)
Germany	1962 to 1966	0.98<br>(0.95 to 1)
Brazil	1897 to 1901	1.05<br>(0.98 to 1.13)
Brazil	1902 to 1906	1.06<br>(1.03 to 1.09)
Brazil	1907 to 1911	1.07<br>(1.05 to 1.09)
Brazil	1912 to 1916	1.07<br>(1.05 to 1.08)
Brazil	1917 to 1921	1.07<br>(1.06 to 1.09)
Brazil	1922 to 1926	1.08<br>(1.06 to 1.09)
Brazil	1927 to 1931	1.08<br>(1.06 to 1.09)
Brazil	1932 to 1936	1.07<br>(1.06 to 1.08)
Brazil	1937 to 1941	1.06<br>(1.05 to 1.07)
Brazil	1942 to 1946	1.05<br>(1.04 to 1.06)
Brazil	1947 to 1951	1.04<br>(1.03 to 1.05)

Brazil	1952 to 1956	1.03<br>(1.02 to 1.04)
Brazil	1957 to 1961	1<br>(1 to 1)
Brazil	1962 to 1966	0.97<br>(0.95 to 0.98)
Afghanistan	1897 to 1901	0.74<br>(0.64 to 0.86)
Afghanistan	1902 to 1906	0.79<br>(0.74 to 0.83)
Afghanistan	1907 to 1911	0.84<br>(0.81 to 0.86)
Afghanistan	1912 to 1916	0.88<br>(0.86 to 0.9)
Afghanistan	1917 to 1921	0.92<br>(0.91 to 0.94)
Afghanistan	1922 to 1926	0.96<br>(0.94 to 0.97)
Afghanistan	1927 to 1931	0.98<br>(0.97 to 1)
Afghanistan	1932 to 1936	1.01<br>(0.99 to 1.02)
Afghanistan	1937 to 1941	1.02<br>(1 to 1.03)
Afghanistan	1942 to 1946	1.03<br>(1.01 to 1.04)
Afghanistan	1947 to 1951	1.03<br>(1.01 to 1.04)
Afghanistan	1952 to 1956	1.02<br>(1 to 1.03)
Afghanistan	1957 to 1961	1<br>(1 to 1)
Afghanistan	1962 to 1966	0.98<br>(0.96 to 1)
Japan	1897 to 1901	1.41<br>(1.27 to 1.57)
Japan	1902 to 1906	1.43<br>(1.35 to 1.51)
Japan	1907 to 1911	1.44<br>(1.38 to 1.5)
Japan	1912 to 1916	1.43<br>(1.38 to 1.49)
Japan	1917 to 1921	1.41<br>(1.36 to 1.46)
Japan	1922 to 1926	1.38<br>(1.33 to 1.43)
Japan	1927 to 1931	1.35<br>(1.31 to 1.4)
Japan	1932 to 1936	1.31<br>(1.27 to 1.35)
Japan	1937 to 1941	1.25<br>(1.21 to 1.29)
Japan	1942 to 1946	1.19<br>(1.15 to 1.23)
Japan	1947 to 1951	1.13<br>(1.09 to 1.17)
Japan	1952 to 1956	1.07<br>(1.03 to 1.11)
Japan	1957 to 1961	1<br>(1 to 1)
Japan	1962 to 1966	0.92<br>(0.87 to 0.97)
Saudi Arabia	1897 to 1901	0.42<br>(0.38 to 0.47)
Saudi Arabia	1902 to 1906	0.47<br>(0.45 to 0.5)

Saudi Arabia	1907 to 1911	0.52<br>(0.51 to 0.54)
Saudi Arabia	1912 to 1916	0.58<br>(0.57 to 0.6)
Saudi Arabia	1917 to 1921	0.64<br>(0.63 to 0.65)
Saudi Arabia	1922 to 1926	0.7<br>(0.69 to 0.71)
Saudi Arabia	1927 to 1931	0.75<br>(0.74 to 0.76)
Saudi Arabia	1932 to 1936	0.8<br>(0.79 to 0.81)
Saudi Arabia	1937 to 1941	0.85<br>(0.84 to 0.86)
Saudi Arabia	1942 to 1946	0.9<br>(0.89 to 0.91)
Saudi Arabia	1947 to 1951	0.94<br>(0.93 to 0.95)
Saudi Arabia	1952 to 1956	0.97<br>(0.96 to 0.98)
Saudi Arabia	1957 to 1961	1<br>(1 to 1)
Saudi Arabia	1962 to 1966	1.03<br>(1.02 to 1.05)
China	1897 to 1901	0.83<br>(0.52 to 1.32)
China	1902 to 1906	0.93<br>(0.8 to 1.08)
China	1907 to 1911	1.03<br>(0.97 to 1.1)
China	1912 to 1916	1.11<br>(1.07 to 1.16)
China	1917 to 1921	1.17<br>(1.13 to 1.21)
China	1922 to 1926	1.21<br>(1.17 to 1.24)
China	1927 to 1931	1.22<br>(1.19 to 1.25)
China	1932 to 1936	1.21<br>(1.18 to 1.24)
China	1937 to 1941	1.2<br>(1.17 to 1.22)
China	1942 to 1946	1.17<br>(1.14 to 1.2)
China	1947 to 1951	1.12<br>(1.09 to 1.14)
China	1952 to 1956	1.06<br>(1.04 to 1.09)
China	1957 to 1961	1<br>(1 to 1)
China	1962 to 1966	0.94<br>(0.91 to 0.97)
Burundi	1897 to 1901	0.75<br>(0.51 to 1.1)
Burundi	1902 to 1906	0.81<br>(0.7 to 0.94)
Burundi	1907 to 1911	0.84<br>(0.78 to 0.9)
Burundi	1912 to 1916	0.88<br>(0.84 to 0.92)
Burundi	1917 to 1921	0.91<br>(0.88 to 0.94)
Burundi	1922 to 1926	0.93<br>(0.91 to 0.96)
Burundi	1927 to 1931	0.95<br>(0.93 to 0.98)

Burundi	1932 to 1936	0.97<br>(0.95 to 0.99)
Burundi	1937 to 1941	0.98<br>(0.96 to 1)
Burundi	1942 to 1946	0.99<br>(0.97 to 1.01)
Burundi	1947 to 1951	1<br>(0.98 to 1.02)
Burundi	1952 to 1956	1<br>(0.98 to 1.02)
Burundi	1957 to 1961	1<br>(1 to 1)
Burundi	1962 to 1966	0.99<br>(0.97 to 1.02)
India	1897 to 1901	0.83<br>(0.79 to 0.88)
India	1902 to 1906	0.86<br>(0.84 to 0.87)
India	1907 to 1911	0.89<br>(0.88 to 0.9)
India	1912 to 1916	0.92<br>(0.91 to 0.93)
India	1917 to 1921	0.95<br>(0.95 to 0.96)
India	1922 to 1926	0.97<br>(0.97 to 0.98)
India	1927 to 1931	0.99<br>(0.98 to 0.99)
India	1932 to 1936	1<br>(1 to 1)
India	1937 to 1941	1.01<br>(1 to 1.01)
India	1942 to 1946	1.01<br>(1.01 to 1.01)
India	1947 to 1951	1.01<br>(1.01 to 1.01)
India	1952 to 1956	1.01<br>(1 to 1.01)
India	1957 to 1961	1<br>(1 to 1)
India	1962 to 1966	0.99<br>(0.98 to 1)
Russia	1897 to 1901	1.18<br>(0.92 to 1.5)
Russia	1902 to 1906	1.19<br>(1.07 to 1.31)
Russia	1907 to 1911	1.19<br>(1.13 to 1.26)
Russia	1912 to 1916	1.17<br>(1.13 to 1.22)
Russia	1917 to 1921	1.15<br>(1.11 to 1.19)
Russia	1922 to 1926	1.11<br>(1.08 to 1.15)
Russia	1927 to 1931	1.1<br>(1.07 to 1.13)
Russia	1932 to 1936	1.08<br>(1.05 to 1.11)
Russia	1937 to 1941	1.06<br>(1.03 to 1.09)
Russia	1942 to 1946	1.04<br>(1.01 to 1.07)
Russia	1947 to 1951	1.03<br>(1 to 1.06)
Russia	1952 to 1956	1.02<br>(0.99 to 1.05)

Russia	1957 to 1961	1<br>(1 to 1)
Russia	1962 to 1966	0.97<br>(0.93 to 1.01)
United Kingdom	1897 to 1901	0.87<br>(0.83 to 0.91)
United Kingdom	1902 to 1906	0.89<br>(0.87 to 0.91)
United Kingdom	1907 to 1911	0.9<br>(0.89 to 0.92)
United Kingdom	1912 to 1916	0.92<br>(0.91 to 0.93)
United Kingdom	1917 to 1921	0.94<br>(0.93 to 0.95)
United Kingdom	1922 to 1926	0.96<br>(0.94 to 0.97)
United Kingdom	1927 to 1931	0.97<br>(0.96 to 0.98)
United Kingdom	1932 to 1936	0.99<br>(0.98 to 1)
United Kingdom	1937 to 1941	1<br>(0.99 to 1.01)
United Kingdom	1942 to 1946	1<br>(0.99 to 1.02)
United Kingdom	1947 to 1951	1<br>(0.99 to 1.02)
United Kingdom	1952 to 1956	1<br>(0.99 to 1.02)
United Kingdom	1957 to 1961	1<br>(1 to 1)
United Kingdom	1962 to 1966	1<br>(0.98 to 1.02)
United States	1897 to 1901	0.71<br>(0.61 to 0.82)
United States	1902 to 1906	0.74<br>(0.69 to 0.8)
United States	1907 to 1911	0.77<br>(0.73 to 0.81)
United States	1912 to 1916	0.8<br>(0.77 to 0.83)
United States	1917 to 1921	0.84<br>(0.81 to 0.87)
United States	1922 to 1926	0.87<br>(0.84 to 0.9)
United States	1927 to 1931	0.9<br>(0.87 to 0.93)
United States	1932 to 1936	0.92<br>(0.89 to 0.95)
United States	1937 to 1941	0.95<br>(0.92 to 0.98)
United States	1942 to 1946	0.97<br>(0.94 to 1)
United States	1947 to 1951	0.98<br>(0.95 to 1.01)
United States	1952 to 1956	0.99<br>(0.96 to 1.02)
United States	1957 to 1961	1<br>(1 to 1)
United States	1962 to 1966	1.01<br>(0.96 to 1.05)

Table S14 APC Model Analysis Results of Asthma Prevalence Among Adults Aged 55 and Above in 11 Example Countries

Asthma<br>Location	Age	Local drift (%/year)
Germany	55 to 59	-2.53<br>(-2.79 to -2.26)
Germany	60 to 64	-2.86<br>(-3.06 to -2.65)
Germany	65 to 69	-3.34<br>(-3.55 to -3.14)
Germany	70 to 74	-3.88<br>(-4.11 to -3.66)
Germany	75 to 79	-4.3<br>(-4.57 to -4.03)
Germany	80 to 84	-4.54<br>(-4.87 to -4.21)
Germany	85 to 89	-4.47<br>(-4.93 to -4)
Germany	90 to 94	-4.13<br>(-4.95 to -3.32)
Germany	95+	-3.44<br>(-5.24 to -1.62)
Brazil	55 to 59	-3.25<br>(-3.45 to -3.06)
Brazil	60 to 64	-3.35<br>(-3.51 to -3.19)
Brazil	65 to 69	-3.36<br>(-3.52 to -3.2)
Brazil	70 to 74	-3.19<br>(-3.38 to -3.01)
Brazil	75 to 79	-2.87<br>(-3.09 to -2.64)
Brazil	80 to 84	-2.41<br>(-2.7 to -2.11)
Brazil	85 to 89	-1.91<br>(-2.34 to -1.47)
Brazil	90 to 94	-1.58<br>(-2.33 to -0.84)
Brazil	95+	-1.52<br>(-3.16 to 0.15)
Afghanistan	55 to 59	-1.49<br>(-1.53 to -1.44)
Afghanistan	60 to 64	-1.41<br>(-1.45 to -1.37)
Afghanistan	65 to 69	-1.41<br>(-1.45 to -1.37)
Afghanistan	70 to 74	-1.43<br>(-1.47 to -1.38)
Afghanistan	75 to 79	-1.39<br>(-1.44 to -1.33)
Afghanistan	80 to 84	-1.29<br>(-1.37 to -1.2)
Afghanistan	85 to 89	-1.12<br>(-1.26 to -0.97)
Afghanistan	90 to 94	-0.9<br>(-1.19 to -0.62)
Afghanistan	95+	-0.69<br>(-1.44 to 0.06)
Japan	55 to 59	-5.49<br>(-6.12 to -4.84)
Japan	60 to 64	-5.7<br>(-6.14 to -5.25)
Japan	65 to 69	-6.2<br>(-6.55 to -5.85)
Japan	70 to 74	-6.87<br>(-7.2 to -6.54)

Japan	75 to 79	-7.28<br>(-7.62 to -6.94)
Japan	80 to 84	-7.12<br>(-7.49 to -6.75)
Japan	85 to 89	-6.27<br>(-6.73 to -5.8)
Japan	90 to 94	-4.96<br>(-5.68 to -4.24)
Japan	95+	-3.63<br>(-5.13 to -2.1)
Saudi Arabia	55 to 59	-1.15<br>(-1.23 to -1.06)
Saudi Arabia	60 to 64	-1.1<br>(-1.17 to -1.02)
Saudi Arabia	65 to 69	-1.05<br>(-1.13 to -0.97)
Saudi Arabia	70 to 74	-1.02<br>(-1.12 to -0.92)
Saudi Arabia	75 to 79	-0.93<br>(-1.06 to -0.81)
Saudi Arabia	80 to 84	-0.74<br>(-0.92 to -0.56)
Saudi Arabia	85 to 89	-0.39<br>(-0.67 to -0.11)
Saudi Arabia	90 to 94	0.08<br>(-0.42 to 0.58)
Saudi Arabia	95+	0.59<br>(-0.45 to 1.63)
China	55 to 59	-3.13<br>(-3.32 to -2.94)
China	60 to 64	-3.59<br>(-3.73 to -3.44)
China	65 to 69	-3.8<br>(-3.93 to -3.67)
China	70 to 74	-3.76<br>(-3.9 to -3.63)
China	75 to 79	-3.47<br>(-3.62 to -3.32)
China	80 to 84	-3.03<br>(-3.25 to -2.82)
China	85 to 89	-2.54<br>(-2.9 to -2.17)
China	90 to 94	-2.1<br>(-2.9 to -1.29)
China	95+	-1.72<br>(-4.16 to 0.78)
Burundi	55 to 59	-1.88<br>(-1.97 to -1.8)
Burundi	60 to 64	-1.72<br>(-1.79 to -1.64)
Burundi	65 to 69	-1.52<br>(-1.6 to -1.44)
Burundi	70 to 74	-1.33<br>(-1.43 to -1.24)
Burundi	75 to 79	-1.15<br>(-1.28 to -1.03)
Burundi	80 to 84	-0.97<br>(-1.15 to -0.78)
Burundi	85 to 89	-0.75<br>(-1.07 to -0.42)
Burundi	90 to 94	-0.55<br>(-1.23 to 0.14)
Burundi	95+	-0.31<br>(-2.12 to 1.53)
India	55 to 59	-0.75<br>(-0.87 to -0.63)

India	60 to 64	-0.88<br>(-0.98 to -0.79)
India	65 to 69	-0.9<br>(-0.99 to -0.81)
India	70 to 74	-0.83<br>(-0.94 to -0.73)
India	75 to 79	-0.74<br>(-0.88 to -0.6)
India	80 to 84	-0.72<br>(-0.91 to -0.53)
India	85 to 89	-0.73<br>(-1.03 to -0.43)
India	90 to 94	-0.7<br>(-1.29 to -0.11)
India	95+	-0.56<br>(-2 to 0.9)
Russia	55 to 59	-6.35<br>(-6.49 to -6.21)
Russia	60 to 64	-6.3<br>(-6.4 to -6.2)
Russia	65 to 69	-6.18<br>(-6.28 to -6.08)
Russia	70 to 74	-6<br>(-6.12 to -5.87)
Russia	75 to 79	-5.71<br>(-5.86 to -5.55)
Russia	80 to 84	-5.5<br>(-5.7 to -5.3)
Russia	85 to 89	-5.19<br>(-5.52 to -4.85)
Russia	90 to 94	-4.82<br>(-5.51 to -4.13)
Russia	95+	-4.37<br>(-6.17 to -2.54)
United Kingdom	55 to 59	-2.94<br>(-3.22 to -2.67)
United Kingdom	60 to 64	-2.95<br>(-3.17 to -2.72)
United Kingdom	65 to 69	-2.82<br>(-3.03 to -2.6)
United Kingdom	70 to 74	-2.59<br>(-2.82 to -2.36)
United Kingdom	75 to 79	-2.21<br>(-2.48 to -1.93)
United Kingdom	80 to 84	-1.58<br>(-1.93 to -1.24)
United Kingdom	85 to 89	-0.72<br>(-1.22 to -0.22)
United Kingdom	90 to 94	0.26<br>(-0.6 to 1.13)
United Kingdom	95+	1.21<br>(-0.72 to 3.18)
United States	55 to 59	1.37<br>(1.15 to 1.58)
United States	60 to 64	1.66<br>(1.49 to 1.84)
United States	65 to 69	1.94<br>(1.77 to 2.11)
United States	70 to 74	2.09<br>(1.9 to 2.27)
United States	75 to 79	2.05<br>(1.83 to 2.27)
United States	80 to 84	1.94<br>(1.66 to 2.22)
United States	85 to 89	1.96<br>(1.56 to 2.37)

United States	90 to 94	2.11 (1.47 to 2.77)
United States	95+	2.36 (1.11 to 3.63)

Asthma Location	Age	Prevalence rate (per 100,000 population)
Germany	55 to 59	5144.24 (4867.94 to 5436.22)
Germany	60 to 64	4858.54 (4594.2 to 5138.09)
Germany	65 to 69	4194.59 (3937.55 to 4468.42)
Germany	70 to 74	3241.06 (3032.86 to 3463.56)
Germany	75 to 79	2269.64 (2111.38 to 2439.77)
Germany	80 to 84	1498.87 (1379.43 to 1628.65)
Germany	85 to 89	1084.05 (976.86 to 1203.01)
Germany	90 to 94	893.43 (770.51 to 1035.97)
Germany	95+	816.22 (640.18 to 1040.68)
Brazil	55 to 59	2135.71 (2053.08 to 2221.68)
Brazil	60 to 64	1946.34 (1869.87 to 2025.93)
Brazil	65 to 69	1825.8 (1741.05 to 1914.67)
Brazil	70 to 74	1706.35 (1623.07 to 1793.89)
Brazil	75 to 79	1482.71 (1404.09 to 1565.74)
Brazil	80 to 84	1272.75 (1195.92 to 1354.52)
Brazil	85 to 89	1253.88 (1163.48 to 1351.3)
Brazil	90 to 94	1362.48 (1234.79 to 1503.38)
Brazil	95+	1530.54 (1331.08 to 1759.9)
Afghanistan	55 to 59	8371.29 (8280.92 to 8462.65)
Afghanistan	60 to 64	8763.21 (8668.28 to 8859.18)
Afghanistan	65 to 69	8792.38 (8687.21 to 8898.83)
Afghanistan	70 to 74	8426.1 (8320.84 to 8532.69)
Afghanistan	75 to 79	7468.45 (7366.22 to 7572.09)
Afghanistan	80 to 84	6292.36 (6187.69 to 6398.8)
Afghanistan	85 to 89	5488.11 (5356.42 to 5623.05)
Afghanistan	90 to 94	5050.73 (4831.12 to 5280.31)
Afghanistan	95+	4863.35 (4419.45 to 5351.84)
Japan	55 to 59	2712.83 (2363.05 to 3114.38)
Japan	60 to 64	2431.14 (2115.57 to 2793.78)

Japan	65 to 69	2154.28 (1857.02 to 2499.14)
Japan	70 to 74	1844.03 (1586.62 to 2143.2)
Japan	75 to 79	1499.89 (1286.47 to 1748.73)
Japan	80 to 84	1224 (1044.68 to 1434.1)
Japan	85 to 89	1111.54 (941.81 to 1311.86)
Japan	90 to 94	1110.31 (927.3 to 1329.43)
Japan	95+	1150.84 (933.91 to 1418.15)
Saudi Arabia	55 to 59	2571.62 (2532.25 to 2611.61)
Saudi Arabia	60 to 64	2745.97 (2702.49 to 2790.15)
Saudi Arabia	65 to 69	3005.93 (2945.89 to 3067.19)
Saudi Arabia	70 to 74	3243.1 (3172.12 to 3315.66)
Saudi Arabia	75 to 79	3183.06 (3100.98 to 3267.33)
Saudi Arabia	80 to 84	2989.93 (2894.87 to 3088.12)
Saudi Arabia	85 to 89	3098.11 (2968.35 to 3233.54)
Saudi Arabia	90 to 94	3473.12 (3250.2 to 3711.32)
Saudi Arabia	95+	3931.69 (3445.5 to 4486.49)
China	55 to 59	1090.29 (1048.02 to 1134.25)
China	60 to 64	1167.99 (1123.18 to 1214.59)
China	65 to 69	1389.19 (1328.31 to 1452.86)
China	70 to 74	1602.44 (1530.98 to 1677.23)
China	75 to 79	1641.48 (1565.59 to 1721.05)
China	80 to 84	1573.25 (1494.43 to 1656.22)
China	85 to 89	1438.54 (1351.02 to 1531.73)
China	90 to 94	1298.27 (1178.47 to 1430.25)
China	95+	1197.01 (974.59 to 1470.18)
Burundi	55 to 59	4441.65 (4369.59 to 4514.89)
Burundi	60 to 64	4776.65 (4698.86 to 4855.73)
Burundi	65 to 69	4646.2 (4551.53 to 4742.83)
Burundi	70 to 74	4192.59 (4099.04 to 4288.28)
Burundi	75 to 79	3710.1 (3615.07 to 3807.63)
Burundi	80 to 84	3269.8 (3163.25 to 3379.94)
Burundi	85 to 89	2930.35 (2789.08 to 3078.77)
Burundi	90 to 94	2693.09 (2460.1 to 2948.15)

Burundi	95+	2505.01 (2058.65 to 3048.16)
India	55 to 59	3613.27 (3535.29 to 3692.98)
India	60 to 64	4330.96 (4239.57 to 4424.32)
India	65 to 69	5002.17 (4874.85 to 5132.8)
India	70 to 74	5527.2 (5378.89 to 5679.59)
India	75 to 79	5739.08 (5571.3 to 5911.91)
India	80 to 84	5733.94 (5538.79 to 5935.98)
India	85 to 89	5783.63 (5525.99 to 6053.28)
India	90 to 94	5884.88 (5480.93 to 6318.59)
India	95+	5977.83 (5224.6 to 6839.67)
Russia	55 to 59	2331.53 (2263.7 to 2401.39)
Russia	60 to 64	1932.99 (1875.85 to 1991.87)
Russia	65 to 69	1446.78 (1399.71 to 1495.44)
Russia	70 to 74	982.89 (948.93 to 1018.06)
Russia	75 to 79	598.31 (575.07 to 622.48)
Russia	80 to 84	337.87 (321.81 to 354.72)
Russia	85 to 89	204.43 (190.65 to 219.21)
Russia	90 to 94	143.1 (126.75 to 161.56)
Russia	95+	113.33 (85.51 to 150.2)
United Kingdom	55 to 59	9408.33 (8870.87 to 9978.36)
United Kingdom	60 to 64	8274.72 (7797.82 to 8780.78)
United Kingdom	65 to 69	6999.83 (6537.58 to 7494.77)
United Kingdom	70 to 74	5747.13 (5351.16 to 6172.4)
United Kingdom	75 to 79	4652.1 (4308.85 to 5022.69)
United Kingdom	80 to 84	3848.13 (3534.15 to 4190.01)
United Kingdom	85 to 89	3679.11 (3332.71 to 4061.51)
United Kingdom	90 to 94	4081.55 (3595.44 to 4633.38)
United Kingdom	95+	4928.71 (4074.05 to 5962.66)
United States	55 to 59	8663.36 (8366.59 to 8970.65)
United States	60 to 64	10089.34 (9742.8 to 10448.21)
United States	65 to 69	11704.04 (11191.14 to 12240.45)
United States	70 to 74	13351.49 (12725.33 to 14008.46)
United States	75 to 79	14396.14 (13670.02 to 15160.83)

United States	80 to 84	15065.85<br>(14222.49 to 15959.22)
United States	85 to 89	16637.3<br>(15550.22 to 17800.37)
United States	90 to 94	19430<br>(17791.75 to 21219.1)
United States	95+	23689.85<br>(20664.21 to 27158.5)

Asthma<br>Location	Period	Prevalence rate ratio
Germany	1992 to 1996	2.37<br>(2.23 to 2.52)
Germany	1997 to 2001	1.65<br>(1.56 to 1.75)
Germany	2002 to 2006	1.61<br>(1.53 to 1.7)
Germany	2007 to 2011	1.4<br>(1.33 to 1.47)
Germany	2012 to 2016	1<br>(1 to 1)
Germany	2017 to 2021	0.84<br>(0.8 to 0.89)
Brazil	1992 to 1996	1.73<br>(1.64 to 1.82)
Brazil	1997 to 2001	1.68<br>(1.61 to 1.77)
Brazil	2002 to 2006	1.52<br>(1.46 to 1.58)
Brazil	2007 to 2011	1.15<br>(1.11 to 1.2)
Brazil	2012 to 2016	1<br>(1 to 1)
Brazil	2017 to 2021	0.98<br>(0.94 to 1.02)
Afghanistan	1992 to 1996	1.31<br>(1.29 to 1.34)
Afghanistan	1997 to 2001	1.25<br>(1.23 to 1.27)
Afghanistan	2002 to 2006	1.18<br>(1.17 to 1.2)
Afghanistan	2007 to 2011	1.1<br>(1.09 to 1.11)
Afghanistan	2012 to 2016	1<br>(1 to 1)
Afghanistan	2017 to 2021	0.98<br>(0.97 to 0.99)
Japan	1992 to 1996	3.99<br>(3.7 to 4.3)
Japan	1997 to 2001	2.9<br>(2.69 to 3.12)
Japan	2002 to 2006	1.83<br>(1.69 to 1.97)
Japan	2007 to 2011	1.32<br>(1.22 to 1.43)
Japan	2012 to 2016	1<br>(1 to 1)
Japan	2017 to 2021	0.87<br>(0.8 to 0.95)
Saudi Arabia	1992 to 1996	1.16<br>(1.13 to 1.2)
Saudi Arabia	1997 to 2001	1.11<br>(1.08 to 1.14)
Saudi Arabia	2002 to 2006	1.1<br>(1.08 to 1.13)

Saudi Arabia	2007 to 2011	1.07 (1.06 to 1.09)
Saudi Arabia	2012 to 2016	1 (1 to 1)
Saudi Arabia	2017 to 2021	0.98 (0.96 to 1)
China	1992 to 1996	1.97 (1.86 to 2.09)
China	1997 to 2001	1.71 (1.63 to 1.8)
China	2002 to 2006	1.38 (1.33 to 1.44)
China	2007 to 2011	1.19 (1.15 to 1.23)
China	2012 to 2016	1 (1 to 1)
China	2017 to 2021	0.93 (0.91 to 0.97)
Burundi	1992 to 1996	1.32 (1.26 to 1.38)
Burundi	1997 to 2001	1.24 (1.19 to 1.28)
Burundi	2002 to 2006	1.14 (1.1 to 1.17)
Burundi	2007 to 2011	1.06 (1.04 to 1.08)
Burundi	2012 to 2016	1 (1 to 1)
Burundi	2017 to 2021	1.02 (1 to 1.04)
India	1992 to 1996	1.21 (1.17 to 1.26)
India	1997 to 2001	1.09 (1.06 to 1.13)
India	2002 to 2006	1.08 (1.05 to 1.11)
India	2007 to 2011	1.06 (1.04 to 1.08)
India	2012 to 2016	1 (1 to 1)
India	2017 to 2021	0.98 (0.96 to 1)
Russia	1992 to 1996	3.37 (3.21 to 3.53)
Russia	1997 to 2001	2.66 (2.56 to 2.77)
Russia	2002 to 2006	1.9 (1.84 to 1.96)
Russia	2007 to 2011	1.4 (1.36 to 1.44)
Russia	2012 to 2016	1 (1 to 1)
Russia	2017 to 2021	0.85 (0.82 to 0.88)
United Kingdom	1992 to 1996	1.63 (1.53 to 1.73)
United Kingdom	1997 to 2001	1.24 (1.17 to 1.32)
United Kingdom	2002 to 2006	1.14 (1.08 to 1.21)
United Kingdom	2007 to 2011	1.12 (1.07 to 1.18)
United Kingdom	2012 to 2016	1 (1 to 1)
United Kingdom	2017 to 2021	1.02 (0.97 to 1.08)

United States	1992 to 1996	0.78 (0.75 to 0.82)
United States	1997 to 2001	0.59 (0.57 to 0.62)
United States	2002 to 2006	0.6 (0.57 to 0.62)
United States	2007 to 2011	0.82 (0.79 to 0.84)
United States	2012 to 2016	1 (1 to 1)
United States	2017 to 2021	1.05 (1.02 to 1.09)

Asthma Location	Cohort	Prevalence rate ratio
Germany	1897 to 1901	7.88 (4.22 to 14.68)
Germany	1902 to 1906	7.45 (5.65 to 9.8)
Germany	1907 to 1911	6.47 (5.54 to 7.56)
Germany	1912 to 1916	5.4 (4.83 to 6.03)
Germany	1917 to 1921	4.26 (3.87 to 4.68)
Germany	1922 to 1926	3.35 (3.1 to 3.62)
Germany	1927 to 1931	2.64 (2.46 to 2.84)
Germany	1932 to 1936	2.1 (1.97 to 2.24)
Germany	1937 to 1941	1.7 (1.6 to 1.81)
Germany	1942 to 1946	1.44 (1.35 to 1.53)
Germany	1947 to 1951	1.26 (1.18 to 1.35)
Germany	1952 to 1956	1.13 (1.06 to 1.21)
Germany	1957 to 1961	1 (1 to 1)
Germany	1962 to 1966	0.88 (0.8 to 0.97)
Brazil	1897 to 1901	4.58 (2.6 to 8.06)
Brazil	1902 to 1906	4.11 (3.21 to 5.27)
Brazil	1907 to 1911	3.79 (3.29 to 4.37)
Brazil	1912 to 1916	3.59 (3.26 to 3.96)
Brazil	1917 to 1921	3.36 (3.12 to 3.63)
Brazil	1922 to 1926	3.06 (2.88 to 3.26)
Brazil	1927 to 1931	2.71 (2.57 to 2.86)
Brazil	1932 to 1936	2.33 (2.22 to 2.45)
Brazil	1937 to 1941	1.95 (1.86 to 2.04)
Brazil	1942 to 1946	1.64 (1.56 to 1.72)
Brazil	1947 to 1951	1.38 (1.31 to 1.44)

Brazil	1952 to 1956	1.16<br>(1.11 to 1.22)
Brazil	1957 to 1961	1<br>(1 to 1)
Brazil	1962 to 1966	0.85<br>(0.8 to 0.91)
Afghanistan	1897 to 1901	1.97<br>(1.53 to 2.53)
Afghanistan	1902 to 1906	1.92<br>(1.75 to 2.12)
Afghanistan	1907 to 1911	1.88<br>(1.79 to 1.97)
Afghanistan	1912 to 1916	1.82<br>(1.77 to 1.88)
Afghanistan	1917 to 1921	1.75<br>(1.71 to 1.78)
Afghanistan	1922 to 1926	1.65<br>(1.62 to 1.67)
Afghanistan	1927 to 1931	1.53<br>(1.51 to 1.55)
Afghanistan	1932 to 1936	1.43<br>(1.41 to 1.44)
Afghanistan	1937 to 1941	1.33<br>(1.31 to 1.35)
Afghanistan	1942 to 1946	1.24<br>(1.22 to 1.25)
Afghanistan	1947 to 1951	1.15<br>(1.13 to 1.16)
Afghanistan	1952 to 1956	1.08<br>(1.06 to 1.09)
Afghanistan	1957 to 1961	1<br>(1 to 1)
Afghanistan	1962 to 1966	0.91<br>(0.89 to 0.92)
Japan	1897 to 1901	26.47<br>(15.43 to 45.41)
Japan	1902 to 1906	24.43<br>(18.49 to 32.29)
Japan	1907 to 1911	22.33<br>(18.25 to 27.32)
Japan	1912 to 1916	18.9<br>(15.88 to 22.5)
Japan	1917 to 1921	14.52<br>(12.33 to 17.09)
Japan	1922 to 1926	10.26<br>(8.78 to 12.01)
Japan	1927 to 1931	6.91<br>(5.93 to 8.05)
Japan	1932 to 1936	4.54<br>(3.92 to 5.27)
Japan	1937 to 1941	3.09<br>(2.67 to 3.58)
Japan	1942 to 1946	2.3<br>(1.98 to 2.67)
Japan	1947 to 1951	1.78<br>(1.52 to 2.07)
Japan	1952 to 1956	1.37<br>(1.17 to 1.61)
Japan	1957 to 1961	1<br>(1 to 1)
Japan	1962 to 1966	0.74<br>(0.58 to 0.95)
Saudi Arabia	1897 to 1901	1.27<br>(0.9 to 1.78)
Saudi Arabia	1902 to 1906	1.36<br>(1.16 to 1.6)

Saudi Arabia	1907 to 1911	1.45 (1.33 to 1.58)
Saudi Arabia	1912 to 1916	1.5 (1.41 to 1.58)
Saudi Arabia	1917 to 1921	1.5 (1.44 to 1.56)
Saudi Arabia	1922 to 1926	1.46 (1.42 to 1.51)
Saudi Arabia	1927 to 1931	1.39 (1.36 to 1.43)
Saudi Arabia	1932 to 1936	1.32 (1.29 to 1.35)
Saudi Arabia	1937 to 1941	1.25 (1.23 to 1.28)
Saudi Arabia	1942 to 1946	1.19 (1.17 to 1.22)
Saudi Arabia	1947 to 1951	1.13 (1.11 to 1.16)
Saudi Arabia	1952 to 1956	1.07 (1.05 to 1.09)
Saudi Arabia	1957 to 1961	1 (1 to 1)
Saudi Arabia	1962 to 1966	0.94 (0.92 to 0.96)
China	1897 to 1901	5.39 (2.28 to 12.74)
China	1902 to 1906	5.1 (3.87 to 6.72)
China	1907 to 1911	4.66 (4.11 to 5.28)
China	1912 to 1916	4.3 (3.98 to 4.64)
China	1917 to 1921	3.94 (3.71 to 4.18)
China	1922 to 1926	3.48 (3.31 to 3.67)
China	1927 to 1931	2.94 (2.8 to 3.09)
China	1932 to 1936	2.44 (2.33 to 2.55)
China	1937 to 1941	2.02 (1.93 to 2.11)
China	1942 to 1946	1.65 (1.58 to 1.73)
China	1947 to 1951	1.34 (1.28 to 1.4)
China	1952 to 1956	1.13 (1.08 to 1.19)
China	1957 to 1961	1 (1 to 1)
China	1962 to 1966	0.92 (0.87 to 0.98)
Burundi	1897 to 1901	1.85 (1 to 3.44)
Burundi	1902 to 1906	1.88 (1.5 to 2.35)
Burundi	1907 to 1911	1.85 (1.66 to 2.05)
Burundi	1912 to 1916	1.83 (1.73 to 1.94)
Burundi	1917 to 1921	1.79 (1.72 to 1.86)
Burundi	1922 to 1926	1.71 (1.66 to 1.77)
Burundi	1927 to 1931	1.63 (1.59 to 1.67)

Burundi	1932 to 1936	1.54<br>(1.5 to 1.57)
Burundi	1937 to 1941	1.44<br>(1.41 to 1.47)
Burundi	1942 to 1946	1.33<br>(1.31 to 1.36)
Burundi	1947 to 1951	1.22<br>(1.2 to 1.25)
Burundi	1952 to 1956	1.11<br>(1.09 to 1.13)
Burundi	1957 to 1961	1<br>(1 to 1)
Burundi	1962 to 1966	0.9<br>(0.87 to 0.92)
India	1897 to 1901	1.5<br>(0.92 to 2.45)
India	1902 to 1906	1.51<br>(1.24 to 1.83)
India	1907 to 1911	1.48<br>(1.34 to 1.63)
India	1912 to 1916	1.43<br>(1.34 to 1.53)
India	1917 to 1921	1.37<br>(1.3 to 1.43)
India	1922 to 1926	1.31<br>(1.27 to 1.36)
India	1927 to 1931	1.28<br>(1.24 to 1.32)
India	1932 to 1936	1.24<br>(1.2 to 1.27)
India	1937 to 1941	1.19<br>(1.16 to 1.22)
India	1942 to 1946	1.13<br>(1.1 to 1.16)
India	1947 to 1951	1.07<br>(1.04 to 1.1)
India	1952 to 1956	1.03<br>(1 to 1.05)
India	1957 to 1961	1<br>(1 to 1)
India	1962 to 1966	0.99<br>(0.95 to 1.02)
Russia	1897 to 1901	27.25<br>(14.42 to 51.5)
Russia	1902 to 1906	22.77<br>(17.94 to 28.9)
Russia	1907 to 1911	18.96<br>(16.9 to 21.28)
Russia	1912 to 1916	15.1<br>(14.09 to 16.18)
Russia	1917 to 1921	11.79<br>(11.19 to 12.43)
Russia	1922 to 1926	8.85<br>(8.48 to 9.23)
Russia	1927 to 1931	6.71<br>(6.47 to 6.96)
Russia	1932 to 1936	5.07<br>(4.9 to 5.24)
Russia	1937 to 1941	3.66<br>(3.54 to 3.77)
Russia	1942 to 1946	2.71<br>(2.62 to 2.8)
Russia	1947 to 1951	1.87<br>(1.8 to 1.94)
Russia	1952 to 1956	1.39<br>(1.34 to 1.44)

Russia	1957 to 1961	1<br>(1 to 1)
Russia	1962 to 1966	0.71<br>(0.67 to 0.75)
United Kingdom	1897 to 1901	1.97<br>(1.03 to 3.76)
United Kingdom	1902 to 1906	2.25<br>(1.69 to 2.99)
United Kingdom	1907 to 1911	2.52<br>(2.14 to 2.98)
United Kingdom	1912 to 1916	2.7<br>(2.4 to 3.04)
United Kingdom	1917 to 1921	2.74<br>(2.49 to 3.01)
United Kingdom	1922 to 1926	2.62<br>(2.41 to 2.85)
United Kingdom	1927 to 1931	2.4<br>(2.22 to 2.59)
United Kingdom	1932 to 1936	2.12<br>(1.98 to 2.27)
United Kingdom	1937 to 1941	1.83<br>(1.71 to 1.96)
United Kingdom	1942 to 1946	1.59<br>(1.49 to 1.71)
United Kingdom	1947 to 1951	1.38<br>(1.29 to 1.48)
United Kingdom	1952 to 1956	1.18<br>(1.09 to 1.26)
United Kingdom	1957 to 1961	1<br>(1 to 1)
United Kingdom	1962 to 1966	0.88<br>(0.8 to 0.97)
United States	1897 to 1901	0.31<br>(0.2 to 0.46)
United States	1902 to 1906	0.35<br>(0.29 to 0.44)
United States	1907 to 1911	0.41<br>(0.36 to 0.46)
United States	1912 to 1916	0.46<br>(0.42 to 0.5)
United States	1917 to 1921	0.5<br>(0.47 to 0.54)
United States	1922 to 1926	0.55<br>(0.52 to 0.58)
United States	1927 to 1931	0.6<br>(0.57 to 0.64)
United States	1932 to 1936	0.66<br>(0.63 to 0.7)
United States	1937 to 1941	0.74<br>(0.71 to 0.77)
United States	1942 to 1946	0.84<br>(0.8 to 0.88)
United States	1947 to 1951	0.91<br>(0.87 to 0.95)
United States	1952 to 1956	0.95<br>(0.91 to 1)
United States	1957 to 1961	1<br>(1 to 1)
United States	1962 to 1966	1.06<br>(1 to 1.12)

Table S15 APC Model Analysis Results of ILD&PS Prevalence Among Adults Aged 55 and Above in 11 Example Countries

ILD&PS Location	Age	Local drift (%/year)
Germany	55 to 59	0.03 (-0.18 to 0.23)
Germany	60 to 64	0.57 (0.42 to 0.72)
Germany	65 to 69	1.05 (0.91 to 1.18)
Germany	70 to 74	1.53 (1.39 to 1.67)
Germany	75 to 79	1.88 (1.71 to 2.04)
Germany	80 to 84	2.11 (1.9 to 2.32)
Germany	85 to 89	2.21 (1.88 to 2.54)
Germany	90 to 94	2.17 (1.48 to 2.86)
Germany	95+	2.13 (0.28 to 4.01)
Brazil	55 to 59	-1.39 (-1.53 to -1.26)
Brazil	60 to 64	-1.27 (-1.37 to -1.16)
Brazil	65 to 69	-1.01 (-1.11 to -0.92)
Brazil	70 to 74	-0.64 (-0.74 to -0.53)
Brazil	75 to 79	-0.21 (-0.34 to -0.09)
Brazil	80 to 84	0.19 (0.03 to 0.36)
Brazil	85 to 89	0.54 (0.29 to 0.8)
Brazil	90 to 94	0.9 (0.4 to 1.4)
Brazil	95+	1.37 (0.05 to 2.71)
Afghanistan	55 to 59	0.99 (0.4 to 1.59)
Afghanistan	60 to 64	1.08 (0.6 to 1.56)
Afghanistan	65 to 69	1.1 (0.65 to 1.54)
Afghanistan	70 to 74	1.06 (0.54 to 1.59)
Afghanistan	75 to 79	1.02 (0.31 to 1.73)
Afghanistan	80 to 84	1 (-0.1 to 2.12)
Afghanistan	85 to 89	1.04 (-1.03 to 3.15)
Afghanistan	90 to 94	0.97 (-3.44 to 5.57)
Afghanistan	95+	2.52 (-15.07 to 23.75)
Japan	55 to 59	-0.28 (-0.45 to -0.11)
Japan	60 to 64	-0.09 (-0.21 to 0.02)
Japan	65 to 69	0.14 (0.05 to 0.22)
Japan	70 to 74	0.47 (0.38 to 0.55)

Japan	75 to 79	0.83<br>(0.73 to 0.92)
Japan	80 to 84	1.14<br>(1.01 to 1.27)
Japan	85 to 89	1.32<br>(1.12 to 1.52)
Japan	90 to 94	1.33<br>(0.91 to 1.75)
Japan	95+	1.2<br>(0.08 to 2.33)
Saudi Arabia	55 to 59	2.32<br>(2.1 to 2.55)
Saudi Arabia	60 to 64	2.23<br>(2.04 to 2.42)
Saudi Arabia	65 to 69	2.18<br>(1.99 to 2.37)
Saudi Arabia	70 to 74	2.21<br>(1.98 to 2.44)
Saudi Arabia	75 to 79	2.3<br>(2 to 2.6)
Saudi Arabia	80 to 84	2.45<br>(2 to 2.89)
Saudi Arabia	85 to 89	2.67<br>(1.91 to 3.44)
Saudi Arabia	90 to 94	2.84<br>(1.33 to 4.37)
Saudi Arabia	95+	2.95<br>(-0.38 to 6.38)
China	55 to 59	0.32<br>(0.12 to 0.53)
China	60 to 64	0.72<br>(0.56 to 0.88)
China	65 to 69	1.21<br>(1.05 to 1.36)
China	70 to 74	1.79<br>(1.61 to 1.97)
China	75 to 79	2.34<br>(2.1 to 2.58)
China	80 to 84	2.79<br>(2.41 to 3.17)
China	85 to 89	3.09<br>(2.36 to 3.83)
China	90 to 94	3.13<br>(1.3 to 4.98)
China	95+	2.93<br>(-3.07 to 9.3)
Burundi	55 to 59	0.08<br>(-0.9 to 1.07)
Burundi	60 to 64	0.13<br>(-0.65 to 0.92)
Burundi	65 to 69	0.16<br>(-0.65 to 0.96)
Burundi	70 to 74	0.13<br>(-0.82 to 1.08)
Burundi	75 to 79	0.04<br>(-1.17 to 1.26)
Burundi	80 to 84	0.08<br>(-1.77 to 1.97)
Burundi	85 to 89	0.85<br>(-3.34 to 5.23)
Burundi	90 to 94	2.64<br>(-10.46 to 17.65)
Burundi	95+	0.23<br>(-19.75 to 25.17)
India	55 to 59	0.14<br>(0.09 to 0.18)

India	60 to 64	0.22<br>(0.18 to 0.25)
India	65 to 69	0.28<br>(0.24 to 0.31)
India	70 to 74	0.33<br>(0.29 to 0.37)
India	75 to 79	0.4<br>(0.34 to 0.45)
India	80 to 84	0.5<br>(0.42 to 0.58)
India	85 to 89	0.69<br>(0.54 to 0.84)
India	90 to 94	0.9<br>(0.55 to 1.25)
India	95+	1.08<br>(0.14 to 2.03)
Russia	55 to 59	-1.72<br>(-1.84 to -1.61)
Russia	60 to 64	-1.75<br>(-1.84 to -1.66)
Russia	65 to 69	-1.76<br>(-1.85 to -1.67)
Russia	70 to 74	-1.73<br>(-1.84 to -1.61)
Russia	75 to 79	-1.74<br>(-1.89 to -1.6)
Russia	80 to 84	-1.8<br>(-2 to -1.6)
Russia	85 to 89	-1.83<br>(-2.17 to -1.49)
Russia	90 to 94	-1.83<br>(-2.55 to -1.11)
Russia	95+	-1.78<br>(-3.72 to 0.2)
United Kingdom	55 to 59	0.49<br>(0.4 to 0.57)
United Kingdom	60 to 64	0.61<br>(0.54 to 0.67)
United Kingdom	65 to 69	0.86<br>(0.81 to 0.92)
United Kingdom	70 to 74	1.27<br>(1.22 to 1.32)
United Kingdom	75 to 79	1.71<br>(1.65 to 1.76)
United Kingdom	80 to 84	2.09<br>(2.02 to 2.17)
United Kingdom	85 to 89	2.37<br>(2.26 to 2.49)
United Kingdom	90 to 94	2.5<br>(2.28 to 2.72)
United Kingdom	95+	2.44<br>(1.89 to 2.99)
United States	55 to 59	-0.35<br>(-0.51 to -0.2)
United States	60 to 64	-0.18<br>(-0.29 to -0.06)
United States	65 to 69	0.02<br>(-0.08 to 0.12)
United States	70 to 74	0.28<br>(0.18 to 0.37)
United States	75 to 79	0.61<br>(0.51 to 0.72)
United States	80 to 84	1.04<br>(0.92 to 1.17)
United States	85 to 89	1.48<br>(1.3 to 1.67)

United States	90 to 94	1.89<br>(1.56 to 2.22)
United States	95+	2.21<br>(1.49 to 2.94)

ILD&PS<br>Location	Age	Prevalence rate (per 100,000 population)
Germany	55 to 59	119.91<br>(115.49 to 124.51)
Germany	60 to 64	167.05<br>(161.04 to 173.3)
Germany	65 to 69	225.38<br>(216.02 to 235.14)
Germany	70 to 74	287.49<br>(275.32 to 300.2)
Germany	75 to 79	327.76<br>(313.42 to 342.75)
Germany	80 to 84	329.78<br>(314.33 to 345.98)
Germany	85 to 89	286.59<br>(270.71 to 303.41)
Germany	90 to 94	227.22<br>(208.74 to 247.34)
Germany	95+	184.82<br>(156.85 to 217.79)
Brazil	55 to 59	48.59<br>(47.38 to 49.83)
Brazil	60 to 64	62.58<br>(61.09 to 64.11)
Brazil	65 to 69	76.3<br>(74.1 to 78.57)
Brazil	70 to 74	90.68<br>(87.97 to 93.49)
Brazil	75 to 79	104.59<br>(101.29 to 107.99)
Brazil	80 to 84	113.32<br>(109.45 to 117.34)
Brazil	85 to 89	117.04<br>(112.35 to 121.92)
Brazil	90 to 94	116.82<br>(110.57 to 123.42)
Brazil	95+	111.44<br>(102.35 to 121.33)
Afghanistan	55 to 59	60.75<br>(53.7 to 68.73)
Afghanistan	60 to 64	76.64<br>(67.78 to 86.64)
Afghanistan	65 to 69	92.46<br>(80.47 to 106.24)
Afghanistan	70 to 74	103.63<br>(89.65 to 119.79)
Afghanistan	75 to 79	105.46<br>(90.04 to 123.53)
Afghanistan	80 to 84	96.58<br>(79.62 to 117.16)
Afghanistan	85 to 89	75.36<br>(55.63 to 102.08)
Afghanistan	90 to 94	69.12<br>(37.66 to 126.88)
Afghanistan	95+	40.49<br>(5.62 to 291.59)
Japan	55 to 59	336.27<br>(325.7 to 347.18)
Japan	60 to 64	537.76<br>(521.72 to 554.31)

Japan	65 to 69	809.12<br>(782.26 to 836.9)
Japan	70 to 74	1092.54<br>(1055.94 to 1130.41)
Japan	75 to 79	1309.46<br>(1264.7 to 1355.82)
Japan	80 to 84	1352.19<br>(1304.14 to 1402.01)
Japan	85 to 89	1192.44<br>(1146.25 to 1240.48)
Japan	90 to 94	923.39<br>(878.9 to 970.13)
Japan	95+	654.38<br>(605.15 to 707.62)
Saudi Arabia	55 to 59	259.72<br>(251.1 to 268.63)
Saudi Arabia	60 to 64	421.23<br>(407.43 to 435.5)
Saudi Arabia	65 to 69	617.95<br>(591.11 to 646)
Saudi Arabia	70 to 74	824.24<br>(784.79 to 865.67)
Saudi Arabia	75 to 79	978.45<br>(924.56 to 1035.48)
Saudi Arabia	80 to 84	1055.58<br>(985.84 to 1130.25)
Saudi Arabia	85 to 89	1040.54<br>(947.15 to 1143.15)
Saudi Arabia	90 to 94	1029.19<br>(875.71 to 1209.56)
Saudi Arabia	95+	1107.42<br>(774.7 to 1583.03)
China	55 to 59	80.83<br>(78.02 to 83.74)
China	60 to 64	113.29<br>(109.46 to 117.26)
China	65 to 69	149.13<br>(142.96 to 155.56)
China	70 to 74	182.7<br>(174.73 to 191.04)
China	75 to 79	215.06<br>(205.01 to 225.59)
China	80 to 84	237.43<br>(224.83 to 250.73)
China	85 to 89	236.68<br>(219.92 to 254.71)
China	90 to 94	219.6<br>(192.37 to 250.69)
China	95+	202.92<br>(146.27 to 281.5)
Burundi	55 to 59	40.55<br>(34.29 to 47.94)
Burundi	60 to 64	53.02<br>(44.93 to 62.57)
Burundi	65 to 69	64.76<br>(52.45 to 79.95)
Burundi	70 to 74	72.07<br>(57.47 to 90.38)
Burundi	75 to 79	74.13<br>(57.64 to 95.35)
Burundi	80 to 84	64.38<br>(46.85 to 88.47)
Burundi	85 to 89	44.09<br>(25.99 to 74.79)
Burundi	90 to 94	37.3<br>(13.1 to 106.17)

Burundi	95+	28.24<br>(1.47 to 542.6)
India	55 to 59	138.38<br>(137.24 to 139.54)
India	60 to 64	196.23<br>(194.67 to 197.8)
India	65 to 69	255.42<br>(252.95 to 257.92)
India	70 to 74	306.86<br>(303.72 to 310.03)
India	75 to 79	338.53<br>(334.77 to 342.33)
India	80 to 84	323.92<br>(319.69 to 328.21)
India	85 to 89	250.28<br>(245.55 to 255.11)
India	90 to 94	197.07<br>(190.31 to 204.08)
India	95+	176.27<br>(163.59 to 189.94)
Russia	55 to 59	46.41<br>(45.39 to 47.45)
Russia	60 to 64	45.82<br>(44.79 to 46.87)
Russia	65 to 69	42.7<br>(41.57 to 43.86)
Russia	70 to 74	37.16<br>(36.12 to 38.23)
Russia	75 to 79	30.05<br>(29.11 to 31.03)
Russia	80 to 84	22.71<br>(21.84 to 23.6)
Russia	85 to 89	16.27<br>(15.37 to 17.21)
Russia	90 to 94	12.26<br>(11.06 to 13.58)
Russia	95+	10.24<br>(7.96 to 13.16)
United Kingdom	55 to 59	206.13<br>(202.83 to 209.49)
United Kingdom	60 to 64	312.48<br>(307.74 to 317.29)
United Kingdom	65 to 69	450.06<br>(442.08 to 458.18)
United Kingdom	70 to 74	606.56<br>(595.62 to 617.71)
United Kingdom	75 to 79	741.16<br>(727.39 to 755.19)
United Kingdom	80 to 84	808.91<br>(793.12 to 825.02)
United Kingdom	85 to 89	777.16<br>(760.21 to 794.48)
United Kingdom	90 to 94	701.59<br>(681.7 to 722.06)
United Kingdom	95+	646.73<br>(616.21 to 678.75)
United States	55 to 59	266.08<br>(258.8 to 273.56)
United States	60 to 64	355.84<br>(346.59 to 365.33)
United States	65 to 69	480.65<br>(465.56 to 496.22)
United States	70 to 74	644.91<br>(624.14 to 666.37)
United States	75 to 79	833.93<br>(806.32 to 862.48)

United States	80 to 84	984.16<br>(950.13 to 1019.41)
United States	85 to 89	1041.65<br>(1002.23 to 1082.61)
United States	90 to 94	1014.36<br>(967.26 to 1063.75)
United States	95+	942.88<br>(874.85 to 1016.2)

ILD&PS<br>Location	Period	Prevalence rate ratio
Germany	1992 to 1996	0.74<br>(0.7 to 0.77)
Germany	1997 to 2001	0.74<br>(0.71 to 0.77)
Germany	2002 to 2006	0.81<br>(0.78 to 0.84)
Germany	2007 to 2011	0.9<br>(0.88 to 0.93)
Germany	2012 to 2016	1<br>(1 to 1)
Germany	2017 to 2021	1.04<br>(1.01 to 1.07)
Brazil	1992 to 1996	1.08<br>(1.04 to 1.12)
Brazil	1997 to 2001	1.07<br>(1.04 to 1.1)
Brazil	2002 to 2006	1.03<br>(1.01 to 1.06)
Brazil	2007 to 2011	0.97<br>(0.95 to 0.99)
Brazil	2012 to 2016	1<br>(1 to 1)
Brazil	2017 to 2021	1.07<br>(1.05 to 1.09)
Afghanistan	1992 to 1996	0.81<br>(0.56 to 1.18)
Afghanistan	1997 to 2001	0.8<br>(0.6 to 1.07)
Afghanistan	2002 to 2006	0.84<br>(0.68 to 1.04)
Afghanistan	2007 to 2011	0.94<br>(0.82 to 1.07)
Afghanistan	2012 to 2016	1<br>(1 to 1)
Afghanistan	2017 to 2021	1.04<br>(0.91 to 1.19)
Japan	1992 to 1996	0.86<br>(0.83 to 0.88)
Japan	1997 to 2001	0.89<br>(0.87 to 0.92)
Japan	2002 to 2006	0.94<br>(0.92 to 0.96)
Japan	2007 to 2011	0.99<br>(0.97 to 1.01)
Japan	2012 to 2016	1<br>(1 to 1)
Japan	2017 to 2021	1.01<br>(0.99 to 1.03)
Saudi Arabia	1992 to 1996	0.59<br>(0.54 to 0.65)
Saudi Arabia	1997 to 2001	0.67<br>(0.62 to 0.72)
Saudi Arabia	2002 to 2006	0.77<br>(0.73 to 0.81)

Saudi Arabia	2007 to 2011	0.89<br>(0.86 to 0.93)
Saudi Arabia	2012 to 2016	1<br>(1 to 1)
Saudi Arabia	2017 to 2021	1.05<br>(1.01 to 1.09)
China	1992 to 1996	0.67<br>(0.59 to 0.76)
China	1997 to 2001	0.66<br>(0.6 to 0.73)
China	2002 to 2006	0.69<br>(0.65 to 0.74)
China	2007 to 2011	0.93<br>(0.89 to 0.97)
China	2012 to 2016	1<br>(1 to 1)
China	2017 to 2021	1.01<br>(0.97 to 1.05)
Burundi	1992 to 1996	0.93<br>(0.5 to 1.71)
Burundi	1997 to 2001	0.93<br>(0.58 to 1.51)
Burundi	2002 to 2006	0.94<br>(0.67 to 1.34)
Burundi	2007 to 2011	0.97<br>(0.77 to 1.22)
Burundi	2012 to 2016	1<br>(1 to 1)
Burundi	2017 to 2021	1.03<br>(0.83 to 1.28)
India	1992 to 1996	0.91<br>(0.89 to 0.93)
India	1997 to 2001	0.91<br>(0.9 to 0.93)
India	2002 to 2006	0.92<br>(0.91 to 0.94)
India	2007 to 2011	0.97<br>(0.97 to 0.98)
India	2012 to 2016	1<br>(1 to 1)
India	2017 to 2021	1.01<br>(1 to 1.02)
Russia	1992 to 1996	1.43<br>(1.36 to 1.5)
Russia	1997 to 2001	1.35<br>(1.3 to 1.4)
Russia	2002 to 2006	1.21<br>(1.18 to 1.25)
Russia	2007 to 2011	1.09<br>(1.07 to 1.12)
Russia	2012 to 2016	1<br>(1 to 1)
Russia	2017 to 2021	0.94<br>(0.91 to 0.96)
United Kingdom	1992 to 1996	0.74<br>(0.73 to 0.75)
United Kingdom	1997 to 2001	0.77<br>(0.76 to 0.78)
United Kingdom	2002 to 2006	0.83<br>(0.82 to 0.84)
United Kingdom	2007 to 2011	0.93<br>(0.92 to 0.94)
United Kingdom	2012 to 2016	1<br>(1 to 1)
United Kingdom	2017 to 2021	1.08<br>(1.07 to 1.09)

United States	1992 to 1996	0.88<br>(0.86 to 0.9)
United States	1997 to 2001	0.96<br>(0.94 to 0.98)
United States	2002 to 2006	0.96<br>(0.94 to 0.98)
United States	2007 to 2011	0.96<br>(0.95 to 0.98)
United States	2012 to 2016	1<br>(1 to 1)
United States	2017 to 2021	1.12<br>(1.1 to 1.14)

ILD&PS<br>Location	Cohort	Prevalence rate ratio
Germany	1897 to 1901	0.41<br>(0.22 to 0.76)
Germany	1902 to 1906	0.46<br>(0.37 to 0.58)
Germany	1907 to 1911	0.51<br>(0.46 to 0.57)
Germany	1912 to 1916	0.56<br>(0.52 to 0.61)
Germany	1917 to 1921	0.63<br>(0.59 to 0.67)
Germany	1922 to 1926	0.7<br>(0.66 to 0.73)
Germany	1927 to 1931	0.79<br>(0.75 to 0.83)
Germany	1932 to 1936	0.87<br>(0.83 to 0.91)
Germany	1937 to 1941	0.94<br>(0.9 to 0.98)
Germany	1942 to 1946	0.99<br>(0.95 to 1.03)
Germany	1947 to 1951	1.02<br>(0.98 to 1.07)
Germany	1952 to 1956	1.02<br>(0.98 to 1.07)
Germany	1957 to 1961	1<br>(1 to 1)
Germany	1962 to 1966	0.94<br>(0.88 to 1)
Brazil	1897 to 1901	0.95<br>(0.61 to 1.48)
Brazil	1902 to 1906	1.09<br>(0.92 to 1.28)
Brazil	1907 to 1911	1.19<br>(1.09 to 1.29)
Brazil	1912 to 1916	1.26<br>(1.19 to 1.33)
Brazil	1917 to 1921	1.31<br>(1.26 to 1.37)
Brazil	1922 to 1926	1.35<br>(1.3 to 1.4)
Brazil	1927 to 1931	1.37<br>(1.32 to 1.42)
Brazil	1932 to 1936	1.36<br>(1.32 to 1.4)
Brazil	1937 to 1941	1.32<br>(1.28 to 1.36)
Brazil	1942 to 1946	1.24<br>(1.2 to 1.28)
Brazil	1947 to 1951	1.15<br>(1.12 to 1.19)

Brazil	1952 to 1956	1.07<br>(1.04 to 1.1)
Brazil	1957 to 1961	1<br>(1 to 1)
Brazil	1962 to 1966	0.93<br>(0.89 to 0.97)
Afghanistan	1897 to 1901	0.32<br>(0 to 213.86)
Afghanistan	1902 to 1906	0.58<br>(0.13 to 2.54)
Afghanistan	1907 to 1911	0.59<br>(0.3 to 1.17)
Afghanistan	1912 to 1916	0.63<br>(0.43 to 0.91)
Afghanistan	1917 to 1921	0.66<br>(0.52 to 0.85)
Afghanistan	1922 to 1926	0.7<br>(0.58 to 0.84)
Afghanistan	1927 to 1931	0.73<br>(0.62 to 0.86)
Afghanistan	1932 to 1936	0.77<br>(0.66 to 0.89)
Afghanistan	1937 to 1941	0.81<br>(0.71 to 0.93)
Afghanistan	1942 to 1946	0.86<br>(0.74 to 0.99)
Afghanistan	1947 to 1951	0.9<br>(0.78 to 1.04)
Afghanistan	1952 to 1956	0.96<br>(0.83 to 1.1)
Afghanistan	1957 to 1961	1<br>(1 to 1)
Afghanistan	1962 to 1966	1.03<br>(0.85 to 1.25)
Japan	1897 to 1901	0.68<br>(0.46 to 0.99)
Japan	1902 to 1906	0.71<br>(0.62 to 0.82)
Japan	1907 to 1911	0.75<br>(0.69 to 0.8)
Japan	1912 to 1916	0.79<br>(0.75 to 0.83)
Japan	1917 to 1921	0.85<br>(0.81 to 0.89)
Japan	1922 to 1926	0.91<br>(0.88 to 0.95)
Japan	1927 to 1931	0.98<br>(0.95 to 1.02)
Japan	1932 to 1936	1.02<br>(0.99 to 1.06)
Japan	1937 to 1941	1.04<br>(1 to 1.08)
Japan	1942 to 1946	1.04<br>(1.01 to 1.08)
Japan	1947 to 1951	1.04<br>(1 to 1.07)
Japan	1952 to 1956	1.02<br>(0.99 to 1.06)
Japan	1957 to 1961	1<br>(1 to 1)
Japan	1962 to 1966	0.97<br>(0.92 to 1.03)
Saudi Arabia	1897 to 1901	0.22<br>(0.07 to 0.66)
Saudi Arabia	1902 to 1906	0.26<br>(0.16 to 0.42)

Saudi Arabia	1907 to 1911	0.3<br>(0.23 to 0.38)
Saudi Arabia	1912 to 1916	0.35<br>(0.3 to 0.4)
Saudi Arabia	1917 to 1921	0.4<br>(0.36 to 0.44)
Saudi Arabia	1922 to 1926	0.45<br>(0.42 to 0.49)
Saudi Arabia	1927 to 1931	0.52<br>(0.49 to 0.55)
Saudi Arabia	1932 to 1936	0.58<br>(0.55 to 0.61)
Saudi Arabia	1937 to 1941	0.64<br>(0.61 to 0.67)
Saudi Arabia	1942 to 1946	0.71<br>(0.68 to 0.74)
Saudi Arabia	1947 to 1951	0.79<br>(0.76 to 0.83)
Saudi Arabia	1952 to 1956	0.89<br>(0.85 to 0.93)
Saudi Arabia	1957 to 1961	1<br>(1 to 1)
Saudi Arabia	1962 to 1966	1.13<br>(1.08 to 1.19)
China	1897 to 1901	0.31<br>(0.04 to 2.46)
China	1902 to 1906	0.35<br>(0.19 to 0.63)
China	1907 to 1911	0.39<br>(0.31 to 0.5)
China	1912 to 1916	0.46<br>(0.41 to 0.52)
China	1917 to 1921	0.54<br>(0.5 to 0.58)
China	1922 to 1926	0.64<br>(0.6 to 0.68)
China	1927 to 1931	0.74<br>(0.7 to 0.77)
China	1932 to 1936	0.83<br>(0.79 to 0.87)
China	1937 to 1941	0.9<br>(0.86 to 0.94)
China	1942 to 1946	0.96<br>(0.92 to 1)
China	1947 to 1951	0.99<br>(0.95 to 1.04)
China	1952 to 1956	0.99<br>(0.95 to 1.03)
China	1957 to 1961	1<br>(1 to 1)
China	1962 to 1966	0.98<br>(0.93 to 1.04)
Burundi	1897 to 1901	1.48<br>(0 to 1418.9)
Burundi	1902 to 1906	0.46<br>(0 to 48.97)
Burundi	1907 to 1911	0.73<br>(0.18 to 3.01)
Burundi	1912 to 1916	0.94<br>(0.52 to 1.73)
Burundi	1917 to 1921	0.98<br>(0.66 to 1.44)
Burundi	1922 to 1926	0.97<br>(0.71 to 1.32)
Burundi	1927 to 1931	0.96<br>(0.74 to 1.26)

Burundi	1932 to 1936	0.97<br>(0.77 to 1.23)
Burundi	1937 to 1941	0.98<br>(0.79 to 1.21)
Burundi	1942 to 1946	0.98<br>(0.79 to 1.22)
Burundi	1947 to 1951	1<br>(0.8 to 1.23)
Burundi	1952 to 1956	1<br>(0.82 to 1.23)
Burundi	1957 to 1961	1<br>(1 to 1)
Burundi	1962 to 1966	0.99<br>(0.76 to 1.3)
India	1897 to 1901	0.7<br>(0.51 to 0.97)
India	1902 to 1906	0.75<br>(0.66 to 0.84)
India	1907 to 1911	0.79<br>(0.76 to 0.83)
India	1912 to 1916	0.85<br>(0.82 to 0.87)
India	1917 to 1921	0.88<br>(0.87 to 0.9)
India	1922 to 1926	0.91<br>(0.9 to 0.92)
India	1927 to 1931	0.93<br>(0.92 to 0.94)
India	1932 to 1936	0.95<br>(0.94 to 0.96)
India	1937 to 1941	0.96<br>(0.95 to 0.97)
India	1942 to 1946	0.98<br>(0.97 to 0.99)
India	1947 to 1951	0.99<br>(0.98 to 1)
India	1952 to 1956	1<br>(0.99 to 1.01)
India	1957 to 1961	1<br>(1 to 1)
India	1962 to 1966	1<br>(0.98 to 1.01)
Russia	1897 to 1901	2.86<br>(1.46 to 5.62)
Russia	1902 to 1906	2.64<br>(2.07 to 3.36)
Russia	1907 to 1911	2.46<br>(2.2 to 2.75)
Russia	1912 to 1916	2.24<br>(2.1 to 2.39)
Russia	1917 to 1921	2.03<br>(1.93 to 2.13)
Russia	1922 to 1926	1.82<br>(1.75 to 1.89)
Russia	1927 to 1931	1.69<br>(1.64 to 1.74)
Russia	1932 to 1936	1.56<br>(1.52 to 1.61)
Russia	1937 to 1941	1.41<br>(1.38 to 1.45)
Russia	1942 to 1946	1.3<br>(1.26 to 1.33)
Russia	1947 to 1951	1.18<br>(1.15 to 1.22)
Russia	1952 to 1956	1.09<br>(1.06 to 1.12)

Russia	1957 to 1961	1<br>(1 to 1)
Russia	1962 to 1966	0.91<br>(0.88 to 0.95)
United Kingdom	1897 to 1901	0.39<br>(0.32 to 0.46)
United Kingdom	1902 to 1906	0.42<br>(0.39 to 0.45)
United Kingdom	1907 to 1911	0.48<br>(0.46 to 0.49)
United Kingdom	1912 to 1916	0.54<br>(0.53 to 0.56)
United Kingdom	1917 to 1921	0.62<br>(0.6 to 0.63)
United Kingdom	1922 to 1926	0.7<br>(0.68 to 0.71)
United Kingdom	1927 to 1931	0.78<br>(0.76 to 0.79)
United Kingdom	1932 to 1936	0.85<br>(0.83 to 0.86)
United Kingdom	1937 to 1941	0.9<br>(0.89 to 0.92)
United Kingdom	1942 to 1946	0.94<br>(0.92 to 0.95)
United Kingdom	1947 to 1951	0.96<br>(0.94 to 0.97)
United Kingdom	1952 to 1956	0.97<br>(0.95 to 0.99)
United Kingdom	1957 to 1961	1<br>(1 to 1)
United Kingdom	1962 to 1966	1.03<br>(1 to 1.05)
United States	1897 to 1901	0.56<br>(0.44 to 0.71)
United States	1902 to 1906	0.64<br>(0.58 to 0.71)
United States	1907 to 1911	0.72<br>(0.68 to 0.77)
United States	1912 to 1916	0.81<br>(0.77 to 0.85)
United States	1917 to 1921	0.9<br>(0.86 to 0.93)
United States	1922 to 1926	0.97<br>(0.93 to 1)
United States	1927 to 1931	1.02<br>(0.98 to 1.05)
United States	1932 to 1936	1.04<br>(1.01 to 1.08)
United States	1937 to 1941	1.06<br>(1.02 to 1.09)
United States	1942 to 1946	1.05<br>(1.02 to 1.08)
United States	1947 to 1951	1.04<br>(1.01 to 1.08)
United States	1952 to 1956	1.03<br>(0.99 to 1.06)
United States	1957 to 1961	1<br>(1 to 1)
United States	1962 to 1966	0.96<br>(0.92 to 1.01)

Table S16 *Net Drift* in the Incidence of CRDs, COPD, Asthma, and ILD & PS in 11 Example Countries (1990-2021)

Cause<br>Location	CRDs<br>Net Drift (%/year)	COPD<br>Net Drift (%/year)	Asthma<br>Net Drift (%/year)	ILD&PS<br>Net Drift (%/year)
Germany	-0.28<br>(-0.47 to -0.09)	0.17<br>(0.05 to 0.3)	-2.33<br>(-2.62 to -2.04)	1.8<br>(1.53 to 2.07)
Brazil	-0.68<br>(-0.76 to -0.59)	-0.26<br>(-0.32 to -0.21)	-1.87<br>(-2.13 to -1.61)	0.99<br>(0.7 to 1.28)
Afghanistan	-0.06<br>(-0.22 to 0.1)	0.4<br>(0.19 to 0.62)	-0.64<br>(-0.9 to -0.38)	-0.06<br>(-2.37 to 2.3)
Japan	-2.09<br>(-2.34 to -1.85)	-0.34<br>(-0.42 to -0.26)	-4.69<br>(-5.02 to -4.36)	1.43<br>(1.04 to 1.83)
Saudi Arabia	0.46<br>(0.3 to 0.61)	1.02<br>(0.83 to 1.21)	-0.61<br>(-0.88 to -0.33)	1.23<br>(0.45 to 2.02)
China	-1.19<br>(-1.44 to -0.93)	-0.83<br>(-1.02 to -0.65)	-2.43<br>(-3.1 to -1.76)	2.63<br>(2.29 to 2.98)
Burundi	-0.27<br>(-0.64 to 0.1)	-0.11<br>(-0.57 to 0.36)	-0.45<br>(-1.07 to 0.17)	-0.53<br>(-4.03 to 3.1)
India	-0.47<br>(-0.53 to -0.42)	-0.26<br>(-0.28 to -0.23)	-1.03<br>(-1.21 to -0.86)	0.37<br>(0.26 to 0.48)
Russia	-1.35<br>(-1.5 to -1.19)	-0.42<br>(-0.63 to -0.21)	-4.11<br>(-4.38 to -3.85)	-2.05<br>(-2.59 to -1.51)
United Kingdom	0.35<br>(0.25 to 0.45)	0.26<br>(0.23 to 0.29)	0.98<br>(0.77 to 1.2)	2.58<br>(2.4 to 2.76)
United States	0.85<br>(0.61 to 1.1)	0.52<br>(0.31 to 0.74)	1.25<br>(0.88 to 1.63)	1.56<br>(1.3 to 1.82)

Table S17 APC Model Analysis Results of CRDs Incidence Among Adults Aged 55 and Above in 11 Example Countries

CRDs<br>Location	Age	Local drift (%/year)
Germany	55 to 59	-1.36<br>(-1.7 to -1.02)
Germany	60 to 64	-1.08<br>(-1.34 to -0.81)
Germany	65 to 69	-0.74<br>(-0.98 to -0.5)
Germany	70 to 74	-0.41<br>(-0.64 to -0.18)
Germany	75 to 79	-0.13<br>(-0.37 to 0.11)
Germany	80 to 84	0.13<br>(-0.12 to 0.38)
Germany	85 to 89	0.25<br>(-0.07 to 0.57)
Germany	90 to 94	0.27<br>(-0.27 to 0.81)
Germany	95+	0.29<br>(-0.9 to 1.51)
Brazil	55 to 59	-1.56<br>(-1.66 to -1.46)
Brazil	60 to 64	-1.35<br>(-1.43 to -1.27)
Brazil	65 to 69	-1.07<br>(-1.14 to -1)
Brazil	70 to 74	-0.75<br>(-0.83 to -0.68)
Brazil	75 to 79	-0.49<br>(-0.58 to -0.41)
Brazil	80 to 84	-0.33<br>(-0.43 to -0.22)
Brazil	85 to 89	-0.25<br>(-0.4 to -0.1)
Brazil	90 to 94	-0.25<br>(-0.51 to 0.01)
Brazil	95+	-0.29<br>(-0.9 to 0.32)
Afghanistan	55 to 59	-0.65<br>(-0.79 to -0.52)
Afghanistan	60 to 64	-0.51<br>(-0.62 to -0.4)
Afghanistan	65 to 69	-0.37<br>(-0.47 to -0.27)
Afghanistan	70 to 74	-0.18<br>(-0.29 to -0.07)
Afghanistan	75 to 79	0.04<br>(-0.1 to 0.18)
Afghanistan	80 to 84	0.22<br>(0.03 to 0.41)
Afghanistan	85 to 89	0.3<br>(0.01 to 0.59)
Afghanistan	90 to 94	0.27<br>(-0.26 to 0.8)
Afghanistan	95+	0.19<br>(-1.1 to 1.5)
Japan	55 to 59	-3.85<br>(-4.42 to -3.27)
Japan	60 to 64	-3.84<br>(-4.23 to -3.45)
Japan	65 to 69	-3.74<br>(-4.03 to -3.44)
Japan	70 to 74	-3.3<br>(-3.57 to -3.03)

Japan	75 to 79	-2.53<br>(-2.81 to -2.25)
Japan	80 to 84	-1.51<br>(-1.82 to -1.19)
Japan	85 to 89	-0.47<br>(-0.89 to -0.04)
Japan	90 to 94	0.35<br>(-0.38 to 1.09)
Japan	95+	0.85<br>(-0.79 to 2.53)
Saudi Arabia	55 to 59	-0.15<br>(-0.29 to -0.02)
Saudi Arabia	60 to 64	0.04<br>(-0.08 to 0.15)
Saudi Arabia	65 to 69	0.2<br>(0.08 to 0.31)
Saudi Arabia	70 to 74	0.35<br>(0.22 to 0.49)
Saudi Arabia	75 to 79	0.53<br>(0.36 to 0.69)
Saudi Arabia	80 to 84	0.69<br>(0.47 to 0.9)
Saudi Arabia	85 to 89	0.8<br>(0.49 to 1.11)
Saudi Arabia	90 to 94	0.81<br>(0.29 to 1.33)
Saudi Arabia	95+	0.75<br>(-0.28 to 1.8)
China	55 to 59	-1.62<br>(-1.86 to -1.38)
China	60 to 64	-1.72<br>(-1.89 to -1.55)
China	65 to 69	-1.59<br>(-1.73 to -1.44)
China	70 to 74	-1.3<br>(-1.45 to -1.15)
China	75 to 79	-0.99<br>(-1.17 to -0.81)
China	80 to 84	-0.78<br>(-1.02 to -0.54)
China	85 to 89	-0.73<br>(-1.12 to -0.33)
China	90 to 94	-0.9<br>(-1.71 to -0.09)
China	95+	-1.31<br>(-3.5 to 0.92)
Burundi	55 to 59	-0.59<br>(-0.82 to -0.37)
Burundi	60 to 64	-0.47<br>(-0.66 to -0.29)
Burundi	65 to 69	-0.34<br>(-0.54 to -0.14)
Burundi	70 to 74	-0.25<br>(-0.48 to -0.02)
Burundi	75 to 79	-0.2<br>(-0.47 to 0.07)
Burundi	80 to 84	-0.18<br>(-0.54 to 0.19)
Burundi	85 to 89	-0.17<br>(-0.77 to 0.44)
Burundi	90 to 94	-0.23<br>(-1.42 to 0.97)
Burundi	95+	-0.12<br>(-3.21 to 3.06)
India	55 to 59	-0.46<br>(-0.52 to -0.4)

India	60 to 64	-0.45<br>(-0.49 to -0.4)
India	65 to 69	-0.39<br>(-0.43 to -0.35)
India	70 to 74	-0.36<br>(-0.41 to -0.32)
India	75 to 79	-0.41<br>(-0.46 to -0.35)
India	80 to 84	-0.5<br>(-0.57 to -0.43)
India	85 to 89	-0.59<br>(-0.7 to -0.49)
India	90 to 94	-0.62<br>(-0.82 to -0.43)
India	95+	-0.59<br>(-1.04 to -0.13)
Russia	55 to 59	-3.61<br>(-3.75 to -3.46)
Russia	60 to 64	-3.03<br>(-3.13 to -2.92)
Russia	65 to 69	-2.32<br>(-2.42 to -2.22)
Russia	70 to 74	-1.63<br>(-1.75 to -1.51)
Russia	75 to 79	-1.05<br>(-1.2 to -0.9)
Russia	80 to 84	-0.66<br>(-0.84 to -0.49)
Russia	85 to 89	-0.34<br>(-0.61 to -0.08)
Russia	90 to 94	-0.05<br>(-0.56 to 0.46)
Russia	95+	0.22<br>(-1.06 to 1.52)
United Kingdom	55 to 59	-0.89<br>(-1.07 to -0.7)
United Kingdom	60 to 64	-0.48<br>(-0.61 to -0.34)
United Kingdom	65 to 69	-0.04<br>(-0.15 to 0.08)
United Kingdom	70 to 74	0.3<br>(0.19 to 0.41)
United Kingdom	75 to 79	0.52<br>(0.41 to 0.64)
United Kingdom	80 to 84	0.7<br>(0.56 to 0.83)
United Kingdom	85 to 89	0.79<br>(0.61 to 0.97)
United Kingdom	90 to 94	0.9<br>(0.61 to 1.2)
United Kingdom	95+	1.06<br>(0.39 to 1.73)
United States	55 to 59	0.92<br>(0.62 to 1.22)
United States	60 to 64	1.01<br>(0.79 to 1.23)
United States	65 to 69	0.91<br>(0.72 to 1.11)
United States	70 to 74	0.79<br>(0.58 to 1)
United States	75 to 79	0.8<br>(0.55 to 1.06)
United States	80 to 84	0.88<br>(0.54 to 1.22)
United States	85 to 89	0.88<br>(0.38 to 1.38)

United States	90 to 94	0.82<br>(-0.01 to 1.66)
United States	95+	0.74<br>(-0.91 to 2.41)

CRDs<br>Location	Age	Prevalence rate (per 100,000 population)
Germany	55 to 59	566.96<br>(528 to 608.8)
Germany	60 to 64	637.11<br>(594.4 to 682.88)
Germany	65 to 69	725.79<br>(670.05 to 786.17)
Germany	70 to 74	860.25<br>(793.43 to 932.7)
Germany	75 to 79	1110.95<br>(1023.73 to 1205.6)
Germany	80 to 84	1607.98<br>(1479.55 to 1747.56)
Germany	85 to 89	2023.45<br>(1851.05 to 2211.92)
Germany	90 to 94	2292.43<br>(2066.58 to 2542.96)
Germany	95+	2434.3<br>(2105.69 to 2814.19)
Brazil	55 to 59	556.62<br>(546.11 to 567.33)
Brazil	60 to 64	672.25<br>(659.94 to 684.8)
Brazil	65 to 69	892.24<br>(872.72 to 912.2)
Brazil	70 to 74	1093.86<br>(1069.15 to 1119.13)
Brazil	75 to 79	1254.1<br>(1224.4 to 1284.52)
Brazil	80 to 84	1541.2<br>(1502.43 to 1580.96)
Brazil	85 to 89	1804.72<br>(1753.93 to 1856.98)
Brazil	90 to 94	1982.27<br>(1913.18 to 2053.87)
Brazil	95+	2117.53<br>(2015.45 to 2224.77)
Afghanistan	55 to 59	1069.9<br>(1038.21 to 1102.57)
Afghanistan	60 to 64	1156.88<br>(1122.45 to 1192.36)
Afghanistan	65 to 69	1291.73<br>(1249.14 to 1335.78)
Afghanistan	70 to 74	1446.46<br>(1397.22 to 1497.44)
Afghanistan	75 to 79	1594.71<br>(1537.1 to 1654.46)
Afghanistan	80 to 84	1904.19<br>(1827.62 to 1983.97)
Afghanistan	85 to 89	2267.84<br>(2153.52 to 2388.24)
Afghanistan	90 to 94	2606.17<br>(2400.92 to 2828.96)
Afghanistan	95+	2980.34<br>(2536.59 to 3501.72)
Japan	55 to 59	400.28<br>(355.35 to 450.88)
Japan	60 to 64	458.85<br>(408.15 to 515.85)

Japan	65 to 69	513.99 (452.76 to 583.51)
Japan	70 to 74	558.67 (491.89 to 634.52)
Japan	75 to 79	620.17 (545.48 to 705.08)
Japan	80 to 84	774.46 (680.21 to 881.76)
Japan	85 to 89	925.77 (809.7 to 1058.48)
Japan	90 to 94	1022.2 (884.62 to 1181.17)
Japan	95+	1117.77 (944.24 to 1323.21)
Saudi Arabia	55 to 59	582.54 (569.2 to 596.18)
Saudi Arabia	60 to 64	692.43 (676.32 to 708.92)
Saudi Arabia	65 to 69	864.65 (838.93 to 891.16)
Saudi Arabia	70 to 74	1074.43 (1039.78 to 1110.24)
Saudi Arabia	75 to 79	1316.71 (1268.94 to 1366.29)
Saudi Arabia	80 to 84	1786.15 (1713.88 to 1861.48)
Saudi Arabia	85 to 89	2359.09 (2244.7 to 2479.32)
Saudi Arabia	90 to 94	2887.77 (2688.86 to 3101.4)
Saudi Arabia	95+	3415.37 (2980.94 to 3913.11)
China	55 to 59	478.29 (456.89 to 500.69)
China	60 to 64	748.81 (716.92 to 782.11)
China	65 to 69	1113.69 (1058.89 to 1171.33)
China	70 to 74	1373.87 (1304.59 to 1446.84)
China	75 to 79	1504.99 (1426.11 to 1588.24)
China	80 to 84	1781.96 (1682.67 to 1887.1)
China	85 to 89	1981.67 (1853.43 to 2118.78)
China	90 to 94	2149.65 (1954.37 to 2364.44)
China	95+	2372.66 (1972.02 to 2854.71)
Burundi	55 to 59	761.29 (731.57 to 792.22)
Burundi	60 to 64	822.77 (790.27 to 856.61)
Burundi	65 to 69	823.97 (782.26 to 867.89)
Burundi	70 to 74	954.43 (903.01 to 1008.78)
Burundi	75 to 79	1171.19 (1103.4 to 1243.16)
Burundi	80 to 84	1337.37 (1247.59 to 1433.61)
Burundi	85 to 89	1389.58 (1264.68 to 1526.8)
Burundi	90 to 94	1383.04 (1178.19 to 1623.51)

Burundi	95+	1368.44<br>(981.18 to 1908.55)
India	55 to 59	823.02<br>(814.36 to 831.78)
India	60 to 64	1278.03<br>(1265.42 to 1290.76)
India	65 to 69	1780.13<br>(1759.14 to 1801.38)
India	70 to 74	2120.97<br>(2094.8 to 2147.48)
India	75 to 79	2333.84<br>(2302.98 to 2365.1)
India	80 to 84	2814.39<br>(2773.49 to 2855.9)
India	85 to 89	3255.21<br>(3198.19 to 3313.24)
India	90 to 94	3609.49<br>(3520.05 to 3701.2)
India	95+	4072.88<br>(3899.86 to 4253.58)
Russia	55 to 59	564.04<br>(547.86 to 580.69)
Russia	60 to 64	542.93<br>(527.37 to 558.96)
Russia	65 to 69	513.06<br>(496.15 to 530.54)
Russia	70 to 74	490.84<br>(474.07 to 508.2)
Russia	75 to 79	473.73<br>(456.51 to 491.61)
Russia	80 to 84	499.38<br>(479.65 to 519.92)
Russia	85 to 89	538.97<br>(513.31 to 565.93)
Russia	90 to 94	580.23<br>(540.78 to 622.56)
Russia	95+	634.32<br>(548.61 to 733.41)
United Kingdom	55 to 59	675.1<br>(651.14 to 699.95)
United Kingdom	60 to 64	930.56<br>(899.13 to 963.09)
United Kingdom	65 to 69	1251.47<br>(1202.96 to 1301.94)
United Kingdom	70 to 74	1523.02<br>(1462.94 to 1585.56)
United Kingdom	75 to 79	1718.82<br>(1648.96 to 1791.64)
United Kingdom	80 to 84	2187.76<br>(2096.17 to 2283.35)
United Kingdom	85 to 89	2675.61<br>(2556.51 to 2800.26)
United Kingdom	90 to 94	3054.58<br>(2897.7 to 3219.96)
United Kingdom	95+	3464.11<br>(3221.82 to 3724.62)
United States	55 to 59	1054.6<br>(1003.28 to 1108.54)
United States	60 to 64	1740.89<br>(1661.96 to 1823.57)
United States	65 to 69	2410.13<br>(2275.33 to 2552.92)
United States	70 to 74	2655.8<br>(2498.71 to 2822.77)
United States	75 to 79	2591.77<br>(2428.16 to 2766.41)

United States	80 to 84	2306.18 (2144.42 to 2480.15)
United States	85 to 89	2106.4 (1931.13 to 2297.59)
United States	90 to 94	2068.7 (1839.38 to 2326.6)
United States	95+	2265.8 (1877.07 to 2735.03)

CRDs<br>Location	Period	Prevalence rate ratio
Germany	1992 to 1996	1.15 (1.1 to 1.21)
Germany	1997 to 2001	1.05 (1 to 1.11)
Germany	2002 to 2006	1.02 (0.98 to 1.07)
Germany	2007 to 2011	1 (0.96 to 1.05)
Germany	2012 to 2016	1 (1 to 1)
Germany	2017 to 2021	1.08 (1.04 to 1.13)
Brazil	1992 to 1996	1.18 (1.16 to 1.21)
Brazil	1997 to 2001	1.16 (1.14 to 1.18)
Brazil	2002 to 2006	1.11 (1.09 to 1.12)
Brazil	2007 to 2011	1.02 (1 to 1.03)
Brazil	2012 to 2016	1 (1 to 1)
Brazil	2017 to 2021	1.04 (1.02 to 1.05)
Afghanistan	1992 to 1996	1.04 (1 to 1.08)
Afghanistan	1997 to 2001	1.03 (1 to 1.06)
Afghanistan	2002 to 2006	1.02 (1 to 1.05)
Afghanistan	2007 to 2011	1 (0.98 to 1.03)
Afghanistan	2012 to 2016	1 (1 to 1)
Afghanistan	2017 to 2021	1.04 (1.01 to 1.06)
Japan	1992 to 1996	1.7 (1.6 to 1.82)
Japan	1997 to 2001	1.39 (1.31 to 1.48)
Japan	2002 to 2006	1.13 (1.06 to 1.2)
Japan	2007 to 2011	1.02 (0.97 to 1.08)
Japan	2012 to 2016	1 (1 to 1)
Japan	2017 to 2021	1.01 (0.95 to 1.07)
Saudi Arabia	1992 to 1996	0.94 (0.9 to 0.97)
Saudi Arabia	1997 to 2001	0.93 (0.9 to 0.96)
Saudi Arabia	2002 to 2006	0.95 (0.92 to 0.98)

Saudi Arabia	2007 to 2011	0.98<br>(0.95 to 1)
Saudi Arabia	2012 to 2016	1<br>(1 to 1)
Saudi Arabia	2017 to 2021	1.04<br>(1.02 to 1.07)
China	1992 to 1996	1.29<br>(1.22 to 1.37)
China	1997 to 2001	1.21<br>(1.15 to 1.27)
China	2002 to 2006	1.11<br>(1.06 to 1.15)
China	2007 to 2011	1.04<br>(1 to 1.07)
China	2012 to 2016	1<br>(1 to 1)
China	2017 to 2021	0.97<br>(0.94 to 1)
Burundi	1992 to 1996	1.09<br>(1 to 1.17)
Burundi	1997 to 2001	1.07<br>(1 to 1.14)
Burundi	2002 to 2006	1.04<br>(0.98 to 1.1)
Burundi	2007 to 2011	1.01<br>(0.96 to 1.05)
Burundi	2012 to 2016	1<br>(1 to 1)
Burundi	2017 to 2021	1.03<br>(0.99 to 1.08)
India	1992 to 1996	1.11<br>(1.09 to 1.12)
India	1997 to 2001	1.07<br>(1.06 to 1.08)
India	2002 to 2006	1.06<br>(1.05 to 1.07)
India	2007 to 2011	1.04<br>(1.03 to 1.05)
India	2012 to 2016	1<br>(1 to 1)
India	2017 to 2021	0.98<br>(0.97 to 0.99)
Russia	1992 to 1996	1.47<br>(1.42 to 1.52)
Russia	1997 to 2001	1.29<br>(1.25 to 1.34)
Russia	2002 to 2006	1.14<br>(1.11 to 1.17)
Russia	2007 to 2011	1.04<br>(1.02 to 1.07)
Russia	2012 to 2016	1<br>(1 to 1)
Russia	2017 to 2021	1.09<br>(1.06 to 1.11)
United Kingdom	1992 to 1996	0.96<br>(0.93 to 0.98)
United Kingdom	1997 to 2001	0.94<br>(0.92 to 0.96)
United Kingdom	2002 to 2006	0.95<br>(0.93 to 0.97)
United Kingdom	2007 to 2011	0.97<br>(0.95 to 0.99)
United Kingdom	2012 to 2016	1<br>(1 to 1)
United Kingdom	2017 to 2021	1.04<br>(1.01 to 1.06)

United States	1992 to 1996	0.84 (0.8 to 0.89)
United States	1997 to 2001	0.83 (0.79 to 0.88)
United States	2002 to 2006	0.9 (0.86 to 0.94)
United States	2007 to 2011	0.96 (0.92 to 1)
United States	2012 to 2016	1 (1 to 1)
United States	2017 to 2021	1.01 (0.97 to 1.05)

CRDs Location	Cohort	Prevalence rate ratio
Germany	1897 to 1901	1.2 (0.8 to 1.8)
Germany	1902 to 1906	1.23 (1.02 to 1.48)
Germany	1907 to 1911	1.24 (1.1 to 1.4)
Germany	1912 to 1916	1.25 (1.13 to 1.38)
Germany	1917 to 1921	1.28 (1.16 to 1.41)
Germany	1922 to 1926	1.29 (1.18 to 1.41)
Germany	1927 to 1931	1.31 (1.2 to 1.43)
Germany	1932 to 1936	1.31 (1.21 to 1.42)
Germany	1937 to 1941	1.29 (1.19 to 1.39)
Germany	1942 to 1946	1.22 (1.13 to 1.33)
Germany	1947 to 1951	1.17 (1.07 to 1.27)
Germany	1952 to 1956	1.09 (1 to 1.19)
Germany	1957 to 1961	1 (1 to 1)
Germany	1962 to 1966	0.91 (0.81 to 1.02)
Brazil	1897 to 1901	1.55 (1.26 to 1.91)
Brazil	1902 to 1906	1.52 (1.39 to 1.66)
Brazil	1907 to 1911	1.49 (1.42 to 1.57)
Brazil	1912 to 1916	1.47 (1.42 to 1.53)
Brazil	1917 to 1921	1.45 (1.41 to 1.5)
Brazil	1922 to 1926	1.44 (1.4 to 1.48)
Brazil	1927 to 1931	1.42 (1.39 to 1.46)
Brazil	1932 to 1936	1.4 (1.36 to 1.43)
Brazil	1937 to 1941	1.35 (1.32 to 1.38)
Brazil	1942 to 1946	1.28 (1.25 to 1.31)
Brazil	1947 to 1951	1.19 (1.16 to 1.21)

Brazil	1952 to 1956	1.09<br>(1.06 to 1.11)
Brazil	1957 to 1961	1<br>(1 to 1)
Brazil	1962 to 1966	0.91<br>(0.89 to 0.94)
Afghanistan	1897 to 1901	1.06<br>(0.69 to 1.64)
Afghanistan	1902 to 1906	1.06<br>(0.89 to 1.27)
Afghanistan	1907 to 1911	1.07<br>(0.97 to 1.17)
Afghanistan	1912 to 1916	1.07<br>(1 to 1.14)
Afghanistan	1917 to 1921	1.09<br>(1.04 to 1.14)
Afghanistan	1922 to 1926	1.12<br>(1.07 to 1.16)
Afghanistan	1927 to 1931	1.13<br>(1.09 to 1.18)
Afghanistan	1932 to 1936	1.13<br>(1.1 to 1.17)
Afghanistan	1937 to 1941	1.12<br>(1.08 to 1.16)
Afghanistan	1942 to 1946	1.1<br>(1.06 to 1.14)
Afghanistan	1947 to 1951	1.07<br>(1.03 to 1.11)
Afghanistan	1952 to 1956	1.04<br>(1 to 1.07)
Afghanistan	1957 to 1961	1<br>(1 to 1)
Afghanistan	1962 to 1966	0.95<br>(0.91 to 1)
Japan	1897 to 1901	2.69<br>(1.53 to 4.73)
Japan	1902 to 1906	2.88<br>(2.21 to 3.75)
Japan	1907 to 1911	3.07<br>(2.56 to 3.66)
Japan	1912 to 1916	3.22<br>(2.77 to 3.74)
Japan	1917 to 1921	3.33<br>(2.89 to 3.82)
Japan	1922 to 1926	3.28<br>(2.87 to 3.75)
Japan	1927 to 1931	3.1<br>(2.73 to 3.53)
Japan	1932 to 1936	2.67<br>(2.35 to 3.02)
Japan	1937 to 1941	2.18<br>(1.93 to 2.47)
Japan	1942 to 1946	1.78<br>(1.57 to 2.03)
Japan	1947 to 1951	1.47<br>(1.29 to 1.67)
Japan	1952 to 1956	1.22<br>(1.06 to 1.39)
Japan	1957 to 1961	1<br>(1 to 1)
Japan	1962 to 1966	0.81<br>(0.66 to 1.01)
Saudi Arabia	1897 to 1901	0.78<br>(0.55 to 1.09)
Saudi Arabia	1902 to 1906	0.8<br>(0.68 to 0.95)

Saudi Arabia	1907 to 1911	0.82<br>(0.74 to 0.91)
Saudi Arabia	1912 to 1916	0.85<br>(0.8 to 0.91)
Saudi Arabia	1917 to 1921	0.89<br>(0.85 to 0.94)
Saudi Arabia	1922 to 1926	0.94<br>(0.9 to 0.98)
Saudi Arabia	1927 to 1931	0.97<br>(0.94 to 1.01)
Saudi Arabia	1932 to 1936	0.99<br>(0.96 to 1.03)
Saudi Arabia	1937 to 1941	1.01<br>(0.98 to 1.04)
Saudi Arabia	1942 to 1946	1.02<br>(0.99 to 1.05)
Saudi Arabia	1947 to 1951	1.03<br>(1 to 1.06)
Saudi Arabia	1952 to 1956	1.02<br>(0.99 to 1.05)
Saudi Arabia	1957 to 1961	1<br>(1 to 1)
Saudi Arabia	1962 to 1966	0.97<br>(0.94 to 1.01)
China	1897 to 1901	2.32<br>(1.08 to 4.98)
China	1902 to 1906	2.05<br>(1.56 to 2.69)
China	1907 to 1911	1.87<br>(1.63 to 2.14)
China	1912 to 1916	1.75<br>(1.6 to 1.91)
China	1917 to 1921	1.69<br>(1.58 to 1.81)
China	1922 to 1926	1.66<br>(1.56 to 1.76)
China	1927 to 1931	1.61<br>(1.53 to 1.71)
China	1932 to 1936	1.53<br>(1.45 to 1.61)
China	1937 to 1941	1.42<br>(1.35 to 1.5)
China	1942 to 1946	1.32<br>(1.26 to 1.39)
China	1947 to 1951	1.2<br>(1.14 to 1.26)
China	1952 to 1956	1.08<br>(1.03 to 1.14)
China	1957 to 1961	1<br>(1 to 1)
China	1962 to 1966	0.97<br>(0.9 to 1.05)
Burundi	1897 to 1901	1.16<br>(0.4 to 3.35)
Burundi	1902 to 1906	1.21<br>(0.81 to 1.79)
Burundi	1907 to 1911	1.17<br>(0.96 to 1.43)
Burundi	1912 to 1916	1.17<br>(1.04 to 1.31)
Burundi	1917 to 1921	1.16<br>(1.06 to 1.26)
Burundi	1922 to 1926	1.14<br>(1.06 to 1.23)
Burundi	1927 to 1931	1.13<br>(1.06 to 1.21)

Burundi	1932 to 1936	1.13<br>(1.07 to 1.19)
Burundi	1937 to 1941	1.11<br>(1.06 to 1.17)
Burundi	1942 to 1946	1.09<br>(1.04 to 1.15)
Burundi	1947 to 1951	1.07<br>(1.02 to 1.13)
Burundi	1952 to 1956	1.04<br>(0.99 to 1.1)
Burundi	1957 to 1961	1<br>(1 to 1)
Burundi	1962 to 1966	0.96<br>(0.9 to 1.02)
India	1897 to 1901	1.35<br>(1.15 to 1.57)
India	1902 to 1906	1.31<br>(1.23 to 1.4)
India	1907 to 1911	1.28<br>(1.24 to 1.33)
India	1912 to 1916	1.24<br>(1.21 to 1.27)
India	1917 to 1921	1.2<br>(1.18 to 1.23)
India	1922 to 1926	1.16<br>(1.14 to 1.18)
India	1927 to 1931	1.13<br>(1.11 to 1.14)
India	1932 to 1936	1.11<br>(1.1 to 1.13)
India	1937 to 1941	1.1<br>(1.09 to 1.12)
India	1942 to 1946	1.08<br>(1.07 to 1.09)
India	1947 to 1951	1.05<br>(1.04 to 1.06)
India	1952 to 1956	1.02<br>(1.01 to 1.04)
India	1957 to 1961	1<br>(1 to 1)
India	1962 to 1966	0.99<br>(0.97 to 1)
Russia	1897 to 1901	2.17<br>(1.4 to 3.35)
Russia	1902 to 1906	2.24<br>(1.89 to 2.66)
Russia	1907 to 1911	2.35<br>(2.15 to 2.57)
Russia	1912 to 1916	2.36<br>(2.22 to 2.5)
Russia	1917 to 1921	2.34<br>(2.23 to 2.46)
Russia	1922 to 1926	2.28<br>(2.19 to 2.38)
Russia	1927 to 1931	2.24<br>(2.16 to 2.33)
Russia	1932 to 1936	2.16<br>(2.09 to 2.23)
Russia	1937 to 1941	1.97<br>(1.91 to 2.04)
Russia	1942 to 1946	1.78<br>(1.72 to 1.84)
Russia	1947 to 1951	1.5<br>(1.45 to 1.56)
Russia	1952 to 1956	1.25<br>(1.21 to 1.3)

Russia	1957 to 1961	1<br>(1 to 1)
Russia	1962 to 1966	0.8<br>(0.76 to 0.84)
United Kingdom	1897 to 1901	0.81<br>(0.65 to 1.01)
United Kingdom	1902 to 1906	0.87<br>(0.79 to 0.96)
United Kingdom	1907 to 1911	0.93<br>(0.87 to 0.99)
United Kingdom	1912 to 1916	0.98<br>(0.93 to 1.03)
United Kingdom	1917 to 1921	1.02<br>(0.97 to 1.07)
United Kingdom	1922 to 1926	1.06<br>(1.01 to 1.1)
United Kingdom	1927 to 1931	1.1<br>(1.05 to 1.14)
United Kingdom	1932 to 1936	1.13<br>(1.09 to 1.18)
United Kingdom	1937 to 1941	1.16<br>(1.11 to 1.21)
United Kingdom	1942 to 1946	1.15<br>(1.11 to 1.2)
United Kingdom	1947 to 1951	1.13<br>(1.09 to 1.18)
United Kingdom	1952 to 1956	1.08<br>(1.04 to 1.13)
United Kingdom	1957 to 1961	1<br>(1 to 1)
United Kingdom	1962 to 1966	0.93<br>(0.88 to 0.99)
United States	1897 to 1901	0.6<br>(0.35 to 1.04)
United States	1902 to 1906	0.63<br>(0.48 to 0.82)
United States	1907 to 1911	0.64<br>(0.55 to 0.76)
United States	1912 to 1916	0.66<br>(0.59 to 0.74)
United States	1917 to 1921	0.69<br>(0.63 to 0.75)
United States	1922 to 1926	0.73<br>(0.68 to 0.79)
United States	1927 to 1931	0.77<br>(0.72 to 0.82)
United States	1932 to 1936	0.79<br>(0.74 to 0.84)
United States	1937 to 1941	0.82<br>(0.77 to 0.87)
United States	1942 to 1946	0.85<br>(0.8 to 0.9)
United States	1947 to 1951	0.9<br>(0.85 to 0.96)
United States	1952 to 1956	0.96<br>(0.91 to 1.02)
United States	1957 to 1961	1<br>(1 to 1)
United States	1962 to 1966	1.01<br>(0.93 to 1.1)

Table S18 APC Model Analysis Results of COPD Incidence Among Adults Aged 55 and Above in 11 Example Countries

COPD Location	Age	Local drift (%/year)
Germany	55 to 59	0.05 (-0.22 to 0.32)
Germany	60 to 64	0.1 (-0.11 to 0.31)
Germany	65 to 69	0.08 (-0.09 to 0.26)
Germany	70 to 74	0.1 (-0.07 to 0.26)
Germany	75 to 79	0.15 (-0.01 to 0.31)
Germany	80 to 84	0.25 (0.08 to 0.42)
Germany	85 to 89	0.3 (0.09 to 0.52)
Germany	90 to 94	0.29 (-0.07 to 0.66)
Germany	95+	0.24 (-0.57 to 1.06)
Brazil	55 to 59	-0.26 (-0.33 to -0.18)
Brazil	60 to 64	-0.25 (-0.31 to -0.19)
Brazil	65 to 69	-0.27 (-0.33 to -0.22)
Brazil	70 to 74	-0.29 (-0.34 to -0.24)
Brazil	75 to 79	-0.29 (-0.34 to -0.23)
Brazil	80 to 84	-0.26 (-0.33 to -0.2)
Brazil	85 to 89	-0.24 (-0.34 to -0.14)
Brazil	90 to 94	-0.23 (-0.4 to -0.06)
Brazil	95+	-0.23 (-0.64 to 0.18)
Afghanistan	55 to 59	-0.06 (-0.28 to 0.17)
Afghanistan	60 to 64	0.15 (-0.03 to 0.33)
Afghanistan	65 to 69	0.29 (0.15 to 0.44)
Afghanistan	70 to 74	0.39 (0.24 to 0.54)
Afghanistan	75 to 79	0.47 (0.29 to 0.64)
Afghanistan	80 to 84	0.54 (0.3 to 0.77)
Afghanistan	85 to 89	0.57 (0.21 to 0.94)
Afghanistan	90 to 94	0.57 (-0.12 to 1.27)
Afghanistan	95+	0.57 (-1.15 to 2.32)
Japan	55 to 59	-1.2 (-1.41 to -0.99)
Japan	60 to 64	-1 (-1.14 to -0.86)
Japan	65 to 69	-0.87 (-0.98 to -0.77)
Japan	70 to 74	-0.65 (-0.74 to -0.56)

Japan	75 to 79	-0.38<br>(-0.48 to -0.29)
Japan	80 to 84	-0.1<br>(-0.2 to 0)
Japan	85 to 89	0.17<br>(0.03 to 0.3)
Japan	90 to 94	0.42<br>(0.18 to 0.65)
Japan	95+	0.62<br>(0.07 to 1.16)
Saudi Arabia	55 to 59	0.75<br>(0.56 to 0.94)
Saudi Arabia	60 to 64	0.93<br>(0.76 to 1.09)
Saudi Arabia	65 to 69	1.03<br>(0.86 to 1.19)
Saudi Arabia	70 to 74	1.06<br>(0.88 to 1.24)
Saudi Arabia	75 to 79	1.08<br>(0.87 to 1.29)
Saudi Arabia	80 to 84	1.11<br>(0.85 to 1.37)
Saudi Arabia	85 to 89	1.11<br>(0.73 to 1.49)
Saudi Arabia	90 to 94	1.04<br>(0.4 to 1.69)
Saudi Arabia	95+	0.92<br>(-0.37 to 2.22)
China	55 to 59	-0.85<br>(-1.04 to -0.66)
China	60 to 64	-0.97<br>(-1.11 to -0.83)
China	65 to 69	-0.89<br>(-1 to -0.77)
China	70 to 74	-0.75<br>(-0.86 to -0.63)
China	75 to 79	-0.65<br>(-0.79 to -0.52)
China	80 to 84	-0.64<br>(-0.82 to -0.46)
China	85 to 89	-0.7<br>(-0.99 to -0.41)
China	90 to 94	-0.94<br>(-1.53 to -0.35)
China	95+	-1.39<br>(-2.98 to 0.22)
Burundi	55 to 59	0.09<br>(-0.26 to 0.43)
Burundi	60 to 64	0.04<br>(-0.25 to 0.32)
Burundi	65 to 69	-0.01<br>(-0.28 to 0.27)
Burundi	70 to 74	-0.07<br>(-0.36 to 0.22)
Burundi	75 to 79	-0.12<br>(-0.45 to 0.22)
Burundi	80 to 84	-0.15<br>(-0.59 to 0.29)
Burundi	85 to 89	-0.17<br>(-0.9 to 0.57)
Burundi	90 to 94	-0.25<br>(-1.74 to 1.25)
Burundi	95+	-0.32<br>(-4.19 to 3.7)
India	55 to 59	-0.12<br>(-0.15 to -0.09)

India	60 to 64	-0.17<br>(-0.19 to -0.14)
India	65 to 69	-0.16<br>(-0.18 to -0.14)
India	70 to 74	-0.18<br>(-0.2 to -0.16)
India	75 to 79	-0.21<br>(-0.24 to -0.19)
India	80 to 84	-0.28<br>(-0.31 to -0.24)
India	85 to 89	-0.36<br>(-0.4 to -0.31)
India	90 to 94	-0.43<br>(-0.52 to -0.33)
India	95+	-0.48<br>(-0.69 to -0.26)
Russia	55 to 59	-0.64<br>(-0.88 to -0.4)
Russia	60 to 64	-0.72<br>(-0.9 to -0.54)
Russia	65 to 69	-0.76<br>(-0.92 to -0.6)
Russia	70 to 74	-0.69<br>(-0.87 to -0.52)
Russia	75 to 79	-0.63<br>(-0.82 to -0.43)
Russia	80 to 84	-0.49<br>(-0.72 to -0.26)
Russia	85 to 89	-0.23<br>(-0.58 to 0.11)
Russia	90 to 94	0.15<br>(-0.52 to 0.82)
Russia	95+	0.56<br>(-1.15 to 2.3)
United Kingdom	55 to 59	0.12<br>(0.05 to 0.19)
United Kingdom	60 to 64	0.18<br>(0.13 to 0.23)
United Kingdom	65 to 69	0.25<br>(0.21 to 0.29)
United Kingdom	70 to 74	0.29<br>(0.25 to 0.32)
United Kingdom	75 to 79	0.28<br>(0.25 to 0.32)
United Kingdom	80 to 84	0.27<br>(0.23 to 0.31)
United Kingdom	85 to 89	0.25<br>(0.19 to 0.3)
United Kingdom	90 to 94	0.28<br>(0.18 to 0.38)
United Kingdom	95+	0.39<br>(0.16 to 0.62)
United States	55 to 59	0.56<br>(0.3 to 0.83)
United States	60 to 64	0.65<br>(0.47 to 0.83)
United States	65 to 69	0.64<br>(0.49 to 0.79)
United States	70 to 74	0.67<br>(0.52 to 0.82)
United States	75 to 79	0.76<br>(0.57 to 0.95)
United States	80 to 84	0.76<br>(0.5 to 1.02)
United States	85 to 89	0.55<br>(0.13 to 0.97)

United States	90 to 94	0.13<br>(-0.62 to 0.88)
United States	95+	-0.35<br>(-1.92 to 1.25)

COPD<br>Location	Age	Prevalence rate (per 100,000 population)
Germany	55 to 59	373.91<br>(354.06 to 394.87)
Germany	60 to 64	488.28<br>(463.44 to 514.44)
Germany	65 to 69	694.34<br>(653.65 to 737.57)
Germany	70 to 74	946.67<br>(890.79 to 1006.06)
Germany	75 to 79	1340.53<br>(1260.76 to 1425.34)
Germany	80 to 84	2016.64<br>(1894.61 to 2146.53)
Germany	85 to 89	2505.04<br>(2345.39 to 2675.55)
Germany	90 to 94	2783.5<br>(2583.24 to 2999.29)
Germany	95+	2890.22<br>(2612.44 to 3197.54)
Brazil	55 to 59	370.92<br>(365.91 to 375.99)
Brazil	60 to 64	509.97<br>(503.37 to 516.65)
Brazil	65 to 69	777.8<br>(765.67 to 790.12)
Brazil	70 to 74	1080.62<br>(1063.26 to 1098.27)
Brazil	75 to 79	1370.94<br>(1348 to 1394.27)
Brazil	80 to 84	1728.33<br>(1697.84 to 1759.38)
Brazil	85 to 89	1964.67<br>(1926.28 to 2003.83)
Brazil	90 to 94	2087.66<br>(2037.79 to 2138.74)
Brazil	95+	2162.51<br>(2091.41 to 2236.03)
Afghanistan	55 to 59	378.87<br>(360.57 to 398.1)
Afghanistan	60 to 64	451.57<br>(429.88 to 474.36)
Afghanistan	65 to 69	634.98<br>(601.47 to 670.36)
Afghanistan	70 to 74	903.3<br>(855.01 to 954.31)
Afghanistan	75 to 79	1222.48<br>(1155.16 to 1293.71)
Afghanistan	80 to 84	1557.9<br>(1466.16 to 1655.38)
Afghanistan	85 to 89	1781.61<br>(1657 to 1915.59)
Afghanistan	90 to 94	1971.71<br>(1767.89 to 2199.02)
Afghanistan	95+	2209.66<br>(1789.9 to 2727.88)
Japan	55 to 59	199.99<br>(192.14 to 208.15)
Japan	60 to 64	270.2<br>(259.84 to 280.97)

Japan	65 to 69	386.85<br>(370.59 to 403.83)
Japan	70 to 74	572.42<br>(548.35 to 597.54)
Japan	75 to 79	838.54<br>(803.05 to 875.6)
Japan	80 to 84	1189.75<br>(1138.74 to 1243.05)
Japan	85 to 89	1417.08<br>(1354.46 to 1482.61)
Japan	90 to 94	1495.02<br>(1424.32 to 1569.22)
Japan	95+	1524.18<br>(1440.77 to 1612.41)
Saudi Arabia	55 to 59	302.3<br>(292.76 to 312.14)
Saudi Arabia	60 to 64	378.16<br>(366.11 to 390.6)
Saudi Arabia	65 to 69	518.37<br>(497.08 to 540.57)
Saudi Arabia	70 to 74	769.91<br>(736.18 to 805.18)
Saudi Arabia	75 to 79	1139.22<br>(1084.87 to 1196.29)
Saudi Arabia	80 to 84	1656.31<br>(1570.07 to 1747.29)
Saudi Arabia	85 to 89	2148.82<br>(2016.99 to 2289.27)
Saudi Arabia	90 to 94	2581.14<br>(2361.51 to 2821.19)
Saudi Arabia	95+	2978.41<br>(2516.49 to 3525.11)
China	55 to 59	352.76<br>(340.31 to 365.67)
China	60 to 64	593.55<br>(573.85 to 613.93)
China	65 to 69	936.05<br>(899.8 to 973.75)
China	70 to 74	1236.48<br>(1187.37 to 1287.61)
China	75 to 79	1433.95<br>(1374.83 to 1495.61)
China	80 to 84	1797.17<br>(1718.91 to 1879)
China	85 to 89	2059.56<br>(1956.79 to 2167.72)
China	90 to 94	2270.56<br>(2114.65 to 2437.96)
China	95+	2529.37<br>(2209.39 to 2895.69)
Burundi	55 to 59	309.57<br>(291.12 to 329.19)
Burundi	60 to 64	369.66<br>(347.75 to 392.96)
Burundi	65 to 69	492.75<br>(456.32 to 532.09)
Burundi	70 to 74	687.35<br>(634.65 to 744.43)
Burundi	75 to 79	910.91<br>(837.54 to 990.71)
Burundi	80 to 84	1065<br>(969.24 to 1170.21)
Burundi	85 to 89	1074.85<br>(951.27 to 1214.47)
Burundi	90 to 94	1011.63<br>(825.35 to 1239.96)

Burundi	95+	931.26 (605.66 to 1431.9)
India	55 to 59	399.04 (396.75 to 401.34)
India	60 to 64	799.64 (795.57 to 803.74)
India	65 to 69	1272.95 (1265.19 to 1280.75)
India	70 to 74	1604.41 (1594.24 to 1614.66)
India	75 to 79	1822.2 (1809.95 to 1834.53)
India	80 to 84	2257.89 (2241.47 to 2274.43)
India	85 to 89	2583.53 (2561.37 to 2605.89)
India	90 to 94	2835.48 (2801.73 to 2869.63)
India	95+	3229.95 (3164.75 to 3296.5)
Russia	55 to 59	321.46 (307.17 to 336.41)
Russia	60 to 64	397.65 (380.29 to 415.8)
Russia	65 to 69	524.57 (497.86 to 552.72)
Russia	70 to 74	638.91 (605.73 to 673.9)
Russia	75 to 79	736.4 (696.74 to 778.31)
Russia	80 to 84	833.55 (785.92 to 884.07)
Russia	85 to 89	891.77 (832.67 to 955.07)
Russia	90 to 94	960.32 (873.69 to 1055.54)
Russia	95+	1044.28 (864.86 to 1260.92)
United Kingdom	55 to 59	405.25 (400.62 to 409.92)
United Kingdom	60 to 64	708.95 (702.01 to 715.96)
United Kingdom	65 to 69	1161.36 (1150.7 to 1172.12)
United Kingdom	70 to 74	1526.32 (1510.01 to 1542.8)
United Kingdom	75 to 79	1767.85 (1748.2 to 1787.73)
United Kingdom	80 to 84	2168.42 (2143.24 to 2193.9)
United Kingdom	85 to 89	2394.45 (2363.93 to 2425.36)
United Kingdom	90 to 94	2450.48 (2411.87 to 2489.7)
United Kingdom	95+	2519.36 (2459.47 to 2580.71)
United States	55 to 59	440.6 (421.35 to 460.73)
United States	60 to 64	1023.41 (984.7 to 1063.65)
United States	65 to 69	1587.91 (1514.71 to 1664.66)
United States	70 to 74	1805.05 (1717.89 to 1896.64)
United States	75 to 79	1813.63 (1721.63 to 1910.55)

United States	80 to 84	1486.32<br>(1403.41 to 1574.13)
United States	85 to 89	1074.73<br>(1001.3 to 1153.54)
United States	90 to 94	791.31<br>(712.03 to 879.42)
United States	95+	688.45<br>(569.57 to 832.15)

COPD<br>Location	Period	Prevalence rate ratio
Germany	1992 to 1996	1.01<br>(0.98 to 1.05)
Germany	1997 to 2001	1<br>(0.96 to 1.03)
Germany	2002 to 2006	0.99<br>(0.96 to 1.03)
Germany	2007 to 2011	0.99<br>(0.96 to 1.02)
Germany	2012 to 2016	1<br>(1 to 1)
Germany	2017 to 2021	1.07<br>(1.04 to 1.1)
Brazil	1992 to 1996	1.07<br>(1.05 to 1.08)
Brazil	1997 to 2001	1.06<br>(1.05 to 1.08)
Brazil	2002 to 2006	1.04<br>(1.03 to 1.05)
Brazil	2007 to 2011	1.01<br>(1 to 1.02)
Brazil	2012 to 2016	1<br>(1 to 1)
Brazil	2017 to 2021	1.01<br>(1.01 to 1.02)
Afghanistan	1992 to 1996	0.95<br>(0.9 to 0.99)
Afghanistan	1997 to 2001	0.96<br>(0.92 to 1)
Afghanistan	2002 to 2006	0.97<br>(0.94 to 1.01)
Afghanistan	2007 to 2011	0.97<br>(0.94 to 1)
Afghanistan	2012 to 2016	1<br>(1 to 1)
Afghanistan	2017 to 2021	1.06<br>(1.03 to 1.1)
Japan	1992 to 1996	1.07<br>(1.05 to 1.1)
Japan	1997 to 2001	1.04<br>(1.02 to 1.06)
Japan	2002 to 2006	1.01<br>(0.99 to 1.03)
Japan	2007 to 2011	1<br>(0.98 to 1.02)
Japan	2012 to 2016	1<br>(1 to 1)
Japan	2017 to 2021	0.98<br>(0.96 to 0.99)
Saudi Arabia	1992 to 1996	0.82<br>(0.79 to 0.86)
Saudi Arabia	1997 to 2001	0.85<br>(0.82 to 0.89)
Saudi Arabia	2002 to 2006	0.9<br>(0.86 to 0.93)

Saudi Arabia	2007 to 2011	0.94<br>(0.91 to 0.98)
Saudi Arabia	2012 to 2016	1<br>(1 to 1)
Saudi Arabia	2017 to 2021	1.06<br>(1.02 to 1.09)
China	1992 to 1996	1.18<br>(1.13 to 1.23)
China	1997 to 2001	1.13<br>(1.09 to 1.18)
China	2002 to 2006	1.07<br>(1.04 to 1.11)
China	2007 to 2011	1.02<br>(0.99 to 1.04)
China	2012 to 2016	1<br>(1 to 1)
China	2017 to 2021	0.96<br>(0.94 to 0.98)
Burundi	1992 to 1996	1.04<br>(0.94 to 1.15)
Burundi	1997 to 2001	1.04<br>(0.95 to 1.14)
Burundi	2002 to 2006	1.03<br>(0.95 to 1.1)
Burundi	2007 to 2011	1<br>(0.95 to 1.07)
Burundi	2012 to 2016	1<br>(1 to 1)
Burundi	2017 to 2021	1.03<br>(0.97 to 1.09)
India	1992 to 1996	1.05<br>(1.04 to 1.06)
India	1997 to 2001	1.04<br>(1.04 to 1.05)
India	2002 to 2006	1.03<br>(1.02 to 1.03)
India	2007 to 2011	1.01<br>(1.01 to 1.02)
India	2012 to 2016	1<br>(1 to 1)
India	2017 to 2021	0.98<br>(0.98 to 0.99)
Russia	1992 to 1996	1.15<br>(1.09 to 1.2)
Russia	1997 to 2001	1.07<br>(1.03 to 1.12)
Russia	2002 to 2006	1.04<br>(1 to 1.09)
Russia	2007 to 2011	1.01<br>(0.98 to 1.05)
Russia	2012 to 2016	1<br>(1 to 1)
Russia	2017 to 2021	1.04<br>(1 to 1.08)
United Kingdom	1992 to 1996	0.94<br>(0.94 to 0.95)
United Kingdom	1997 to 2001	0.97<br>(0.96 to 0.97)
United Kingdom	2002 to 2006	0.99<br>(0.98 to 0.99)
United Kingdom	2007 to 2011	1<br>(1 to 1)
United Kingdom	2012 to 2016	1.01<br>(1 to 1.01)
United Kingdom	2017 to 2021	1.01<br>(1 to 1.01)

United States	1992 to 1996	0.86<br>(0.82 to 0.9)
United States	1997 to 2001	0.93<br>(0.89 to 0.97)
United States	2002 to 2006	1.01<br>(0.98 to 1.05)
United States	2007 to 2011	1.01<br>(0.98 to 1.05)
United States	2012 to 2016	1<br>(1 to 1)
United States	2017 to 2021	0.99<br>(0.96 to 1.02)

COPD<br>Location	Cohort	Prevalence rate ratio
Germany	1897 to 1901	0.9<br>(0.68 to 1.19)
Germany	1902 to 1906	0.91<br>(0.8 to 1.03)
Germany	1907 to 1911	0.91<br>(0.84 to 1)
Germany	1912 to 1916	0.92<br>(0.86 to 0.99)
Germany	1917 to 1921	0.94<br>(0.88 to 1.01)
Germany	1922 to 1926	0.96<br>(0.9 to 1.02)
Germany	1927 to 1931	0.97<br>(0.91 to 1.04)
Germany	1932 to 1936	0.98<br>(0.92 to 1.04)
Germany	1937 to 1941	0.98<br>(0.92 to 1.04)
Germany	1942 to 1946	0.98<br>(0.92 to 1.04)
Germany	1947 to 1951	0.99<br>(0.93 to 1.05)
Germany	1952 to 1956	1<br>(0.94 to 1.06)
Germany	1957 to 1961	1<br>(1 to 1)
Germany	1962 to 1966	0.98<br>(0.9 to 1.07)
Brazil	1897 to 1901	1.16<br>(1.01 to 1.33)
Brazil	1902 to 1906	1.15<br>(1.08 to 1.22)
Brazil	1907 to 1911	1.13<br>(1.1 to 1.17)
Brazil	1912 to 1916	1.12<br>(1.09 to 1.15)
Brazil	1917 to 1921	1.11<br>(1.09 to 1.13)
Brazil	1922 to 1926	1.1<br>(1.08 to 1.12)
Brazil	1927 to 1931	1.08<br>(1.06 to 1.1)
Brazil	1932 to 1936	1.07<br>(1.05 to 1.08)
Brazil	1937 to 1941	1.05<br>(1.03 to 1.07)
Brazil	1942 to 1946	1.03<br>(1.02 to 1.05)
Brazil	1947 to 1951	1.02<br>(1.01 to 1.04)

Brazil	1952 to 1956	1.01<br>(1 to 1.03)
Brazil	1957 to 1961	1<br>(1 to 1)
Brazil	1962 to 1966	0.98<br>(0.96 to 1)
Afghanistan	1897 to 1901	0.8<br>(0.45 to 1.42)
Afghanistan	1902 to 1906	0.82<br>(0.65 to 1.03)
Afghanistan	1907 to 1911	0.84<br>(0.74 to 0.95)
Afghanistan	1912 to 1916	0.86<br>(0.79 to 0.94)
Afghanistan	1917 to 1921	0.89<br>(0.83 to 0.96)
Afghanistan	1922 to 1926	0.92<br>(0.86 to 0.98)
Afghanistan	1927 to 1931	0.94<br>(0.89 to 1)
Afghanistan	1932 to 1936	0.97<br>(0.91 to 1.02)
Afghanistan	1937 to 1941	0.99<br>(0.94 to 1.04)
Afghanistan	1942 to 1946	1<br>(0.95 to 1.05)
Afghanistan	1947 to 1951	1.01<br>(0.95 to 1.06)
Afghanistan	1952 to 1956	1.01<br>(0.96 to 1.07)
Afghanistan	1957 to 1961	1<br>(1 to 1)
Afghanistan	1962 to 1966	0.97<br>(0.89 to 1.04)
Japan	1897 to 1901	1.12<br>(0.93 to 1.35)
Japan	1902 to 1906	1.17<br>(1.08 to 1.28)
Japan	1907 to 1911	1.23<br>(1.16 to 1.3)
Japan	1912 to 1916	1.27<br>(1.21 to 1.34)
Japan	1917 to 1921	1.3<br>(1.24 to 1.36)
Japan	1922 to 1926	1.3<br>(1.24 to 1.36)
Japan	1927 to 1931	1.3<br>(1.25 to 1.36)
Japan	1932 to 1936	1.28<br>(1.22 to 1.34)
Japan	1937 to 1941	1.24<br>(1.19 to 1.29)
Japan	1942 to 1946	1.17<br>(1.13 to 1.23)
Japan	1947 to 1951	1.11<br>(1.06 to 1.16)
Japan	1952 to 1956	1.06<br>(1.01 to 1.1)
Japan	1957 to 1961	1<br>(1 to 1)
Japan	1962 to 1966	0.9<br>(0.84 to 0.97)
Saudi Arabia	1897 to 1901	0.57<br>(0.38 to 0.87)
Saudi Arabia	1902 to 1906	0.59<br>(0.48 to 0.73)

Saudi Arabia	1907 to 1911	0.61<br>(0.54 to 0.69)
Saudi Arabia	1912 to 1916	0.64<br>(0.59 to 0.69)
Saudi Arabia	1917 to 1921	0.68<br>(0.63 to 0.73)
Saudi Arabia	1922 to 1926	0.72<br>(0.68 to 0.76)
Saudi Arabia	1927 to 1931	0.76<br>(0.72 to 0.8)
Saudi Arabia	1932 to 1936	0.8<br>(0.76 to 0.84)
Saudi Arabia	1937 to 1941	0.84<br>(0.81 to 0.88)
Saudi Arabia	1942 to 1946	0.89<br>(0.85 to 0.93)
Saudi Arabia	1947 to 1951	0.94<br>(0.9 to 0.98)
Saudi Arabia	1952 to 1956	0.98<br>(0.94 to 1.02)
Saudi Arabia	1957 to 1961	1<br>(1 to 1)
Saudi Arabia	1962 to 1966	1.01<br>(0.96 to 1.06)
China	1897 to 1901	1.94<br>(1.11 to 3.36)
China	1902 to 1906	1.68<br>(1.38 to 2.06)
China	1907 to 1911	1.53<br>(1.38 to 1.69)
China	1912 to 1916	1.44<br>(1.35 to 1.54)
China	1917 to 1921	1.39<br>(1.31 to 1.46)
China	1922 to 1926	1.35<br>(1.29 to 1.41)
China	1927 to 1931	1.32<br>(1.26 to 1.37)
China	1932 to 1936	1.27<br>(1.22 to 1.32)
China	1937 to 1941	1.22<br>(1.17 to 1.27)
China	1942 to 1946	1.18<br>(1.13 to 1.23)
China	1947 to 1951	1.12<br>(1.07 to 1.16)
China	1952 to 1956	1.05<br>(1.01 to 1.09)
China	1957 to 1961	1<br>(1 to 1)
China	1962 to 1966	1.01<br>(0.95 to 1.07)
Burundi	1897 to 1901	1.09<br>(0.29 to 4.14)
Burundi	1902 to 1906	1.07<br>(0.65 to 1.75)
Burundi	1907 to 1911	1.03<br>(0.81 to 1.32)
Burundi	1912 to 1916	1.03<br>(0.89 to 1.19)
Burundi	1917 to 1921	1.01<br>(0.91 to 1.13)
Burundi	1922 to 1926	1<br>(0.91 to 1.11)
Burundi	1927 to 1931	1<br>(0.91 to 1.09)

Burundi	1932 to 1936	0.99<br>(0.91 to 1.08)
Burundi	1937 to 1941	0.99<br>(0.91 to 1.07)
Burundi	1942 to 1946	0.98<br>(0.91 to 1.06)
Burundi	1947 to 1951	0.99<br>(0.92 to 1.07)
Burundi	1952 to 1956	1<br>(0.92 to 1.07)
Burundi	1957 to 1961	1<br>(1 to 1)
Burundi	1962 to 1966	1.01<br>(0.91 to 1.11)
India	1897 to 1901	1.2<br>(1.12 to 1.29)
India	1902 to 1906	1.17<br>(1.13 to 1.21)
India	1907 to 1911	1.14<br>(1.12 to 1.16)
India	1912 to 1916	1.11<br>(1.1 to 1.12)
India	1917 to 1921	1.08<br>(1.07 to 1.09)
India	1922 to 1926	1.07<br>(1.06 to 1.08)
India	1927 to 1931	1.05<br>(1.05 to 1.06)
India	1932 to 1936	1.04<br>(1.04 to 1.05)
India	1937 to 1941	1.03<br>(1.03 to 1.04)
India	1942 to 1946	1.03<br>(1.02 to 1.03)
India	1947 to 1951	1.02<br>(1.01 to 1.03)
India	1952 to 1956	1.01<br>(1 to 1.02)
India	1957 to 1961	1<br>(1 to 1)
India	1962 to 1966	1.01<br>(1 to 1.02)
Russia	1897 to 1901	1.1<br>(0.61 to 1.96)
Russia	1902 to 1906	1.16<br>(0.93 to 1.46)
Russia	1907 to 1911	1.26<br>(1.12 to 1.42)
Russia	1912 to 1916	1.3<br>(1.2 to 1.4)
Russia	1917 to 1921	1.29<br>(1.2 to 1.38)
Russia	1922 to 1926	1.25<br>(1.17 to 1.33)
Russia	1927 to 1931	1.24<br>(1.17 to 1.31)
Russia	1932 to 1936	1.2<br>(1.14 to 1.27)
Russia	1937 to 1941	1.14<br>(1.08 to 1.2)
Russia	1942 to 1946	1.1<br>(1.04 to 1.16)
Russia	1947 to 1951	1.06<br>(1.01 to 1.12)
Russia	1952 to 1956	1.03<br>(0.97 to 1.08)

Russia	1957 to 1961	1<br>(1 to 1)
Russia	1962 to 1966	0.97<br>(0.89 to 1.04)
United Kingdom	1897 to 1901	0.84<br>(0.78 to 0.91)
United Kingdom	1902 to 1906	0.87<br>(0.85 to 0.9)
United Kingdom	1907 to 1911	0.9<br>(0.88 to 0.92)
United Kingdom	1912 to 1916	0.91<br>(0.9 to 0.92)
United Kingdom	1917 to 1921	0.92<br>(0.91 to 0.93)
United Kingdom	1922 to 1926	0.93<br>(0.92 to 0.94)
United Kingdom	1927 to 1931	0.94<br>(0.93 to 0.95)
United Kingdom	1932 to 1936	0.96<br>(0.95 to 0.97)
United Kingdom	1937 to 1941	0.97<br>(0.96 to 0.98)
United Kingdom	1942 to 1946	0.99<br>(0.97 to 1)
United Kingdom	1947 to 1951	1<br>(0.99 to 1.01)
United Kingdom	1952 to 1956	1<br>(1 to 1)
United Kingdom	1957 to 1961	1<br>(0.99 to 1.02)
United Kingdom	1962 to 1966	1<br>(0.98 to 1.03)
United States	1897 to 1901	0.85<br>(0.5 to 1.43)
United States	1902 to 1906	0.82<br>(0.64 to 1.05)
United States	1907 to 1911	0.77<br>(0.67 to 0.88)
United States	1912 to 1916	0.74<br>(0.67 to 0.8)
United States	1917 to 1921	0.75<br>(0.7 to 0.8)
United States	1922 to 1926	0.8<br>(0.75 to 0.85)
United States	1927 to 1931	0.83<br>(0.79 to 0.88)
United States	1932 to 1936	0.85<br>(0.81 to 0.89)
United States	1937 to 1941	0.88<br>(0.84 to 0.93)
United States	1942 to 1946	0.92<br>(0.87 to 0.96)
United States	1947 to 1951	0.94<br>(0.9 to 0.99)
United States	1952 to 1956	0.97<br>(0.93 to 1.02)
United States	1957 to 1961	1<br>(1 to 1)
United States	1962 to 1966	1.01<br>(0.94 to 1.09)

Table S19 APC Model Analysis Results of Asthma Incidence Among Adults Aged 55 and Above in 11 Example Countries

Asthma Location	Age	Local drift (%/year)
Germany	55 to 59	-3.79 (-4.1 to -3.47)
Germany	60 to 64	-3.69 (-3.95 to -3.44)
Germany	65 to 69	-3.29 (-3.58 to -3)
Germany	70 to 74	-2.73 (-3.07 to -2.39)
Germany	75 to 79	-2.26 (-2.67 to -1.85)
Germany	80 to 84	-1.83 (-2.28 to -1.37)
Germany	85 to 89	-1.48 (-2.03 to -0.92)
Germany	90 to 94	-1.22 (-2.07 to -0.37)
Germany	95+	-0.78 (-2.52 to 0.99)
Brazil	55 to 59	-3.85 (-4.1 to -3.6)
Brazil	60 to 64	-3.57 (-3.77 to -3.37)
Brazil	65 to 69	-3.02 (-3.22 to -2.81)
Brazil	70 to 74	-2.25 (-2.49 to -2.01)
Brazil	75 to 79	-1.49 (-1.8 to -1.19)
Brazil	80 to 84	-0.94 (-1.33 to -0.56)
Brazil	85 to 89	-0.66 (-1.18 to -0.14)
Brazil	90 to 94	-0.67 (-1.52 to 0.19)
Brazil	95+	-0.87 (-2.73 to 1.04)
Afghanistan	55 to 59	-0.91 (-1.08 to -0.74)
Afghanistan	60 to 64	-0.94 (-1.08 to -0.8)
Afghanistan	65 to 69	-0.97 (-1.11 to -0.83)
Afghanistan	70 to 74	-0.87 (-1.04 to -0.71)
Afghanistan	75 to 79	-0.64 (-0.87 to -0.41)
Afghanistan	80 to 84	-0.42 (-0.74 to -0.1)
Afghanistan	85 to 89	-0.32 (-0.79 to 0.16)
Afghanistan	90 to 94	-0.31 (-1.15 to 0.53)
Afghanistan	95+	-0.38 (-2.36 to 1.65)
Japan	55 to 59	-6.22 (-6.97 to -5.45)
Japan	60 to 64	-6.72 (-7.24 to -6.21)
Japan	65 to 69	-7.2 (-7.61 to -6.8)
Japan	70 to 74	-7.17 (-7.55 to -6.78)

Japan	75 to 79	-6.25 (-6.66 to -5.84)
Japan	80 to 84	-4.36 (-4.81 to -3.91)
Japan	85 to 89	-2.09 (-2.68 to -1.5)
Japan	90 to 94	-0.25 (-1.22 to 0.73)
Japan	95+	0.74 (-1.37 to 2.89)
Saudi Arabia	55 to 59	-1.17 (-1.37 to -0.97)
Saudi Arabia	60 to 64	-1.06 (-1.23 to -0.89)
Saudi Arabia	65 to 69	-0.99 (-1.17 to -0.8)
Saudi Arabia	70 to 74	-0.88 (-1.11 to -0.64)
Saudi Arabia	75 to 79	-0.69 (-0.99 to -0.38)
Saudi Arabia	80 to 84	-0.46 (-0.86 to -0.06)
Saudi Arabia	85 to 89	-0.21 (-0.78 to 0.37)
Saudi Arabia	90 to 94	-0.01 (-0.95 to 0.93)
Saudi Arabia	95+	0.14 (-1.69 to 2.01)
China	55 to 59	-3.67 (-4.13 to -3.21)
China	60 to 64	-3.88 (-4.21 to -3.55)
China	65 to 69	-3.78 (-4.06 to -3.49)
China	70 to 74	-3.2 (-3.51 to -2.89)
China	75 to 79	-2.31 (-2.71 to -1.92)
China	80 to 84	-1.58 (-2.16 to -1)
China	85 to 89	-1.17 (-2.16 to -0.18)
China	90 to 94	-1.09 (-3.19 to 1.06)
China	95+	-1.21 (-7.26 to 5.23)
Burundi	55 to 59	-1.05 (-1.34 to -0.76)
Burundi	60 to 64	-0.87 (-1.13 to -0.62)
Burundi	65 to 69	-0.67 (-0.98 to -0.37)
Burundi	70 to 74	-0.51 (-0.89 to -0.13)
Burundi	75 to 79	-0.39 (-0.86 to 0.09)
Burundi	80 to 84	-0.31 (-0.96 to 0.35)
Burundi	85 to 89	-0.25 (-1.31 to 0.81)
Burundi	90 to 94	-0.13 (-2.17 to 1.95)
Burundi	95+	0.02 (-5.03 to 5.35)
India	55 to 59	-0.85 (-0.98 to -0.72)

India	60 to 64	-0.9<br>(-1.01 to -0.79)
India	65 to 69	-0.87<br>(-0.97 to -0.76)
India	70 to 74	-0.86<br>(-0.99 to -0.73)
India	75 to 79	-1<br>(-1.16 to -0.84)
India	80 to 84	-1.21<br>(-1.42 to -1)
India	85 to 89	-1.35<br>(-1.66 to -1.04)
India	90 to 94	-1.28<br>(-1.85 to -0.71)
India	95+	-1.02<br>(-2.35 to 0.33)
Russia	55 to 59	-6.41<br>(-6.55 to -6.26)
Russia	60 to 64	-5.68<br>(-5.79 to -5.57)
Russia	65 to 69	-4.92<br>(-5.05 to -4.79)
Russia	70 to 74	-4.3<br>(-4.48 to -4.12)
Russia	75 to 79	-3.76<br>(-4.02 to -3.51)
Russia	80 to 84	-3.39<br>(-3.75 to -3.03)
Russia	85 to 89	-3.11<br>(-3.62 to -2.6)
Russia	90 to 94	-2.98<br>(-3.85 to -2.11)
Russia	95+	-2.94<br>(-4.93 to -0.91)
United Kingdom	55 to 59	-2.83<br>(-3.12 to -2.53)
United Kingdom	60 to 64	-1.96<br>(-2.2 to -1.72)
United Kingdom	65 to 69	-0.63<br>(-0.88 to -0.38)
United Kingdom	70 to 74	0.71<br>(0.43 to 0.99)
United Kingdom	75 to 79	1.78<br>(1.46 to 2.11)
United Kingdom	80 to 84	2.52<br>(2.16 to 2.87)
United Kingdom	85 to 89	2.78<br>(2.36 to 3.2)
United Kingdom	90 to 94	2.74<br>(2.12 to 3.36)
United Kingdom	95+	2.67<br>(1.4 to 3.95)
United States	55 to 59	1.2<br>(0.76 to 1.63)
United States	60 to 64	1.67<br>(1.32 to 2.02)
United States	65 to 69	1.68<br>(1.32 to 2.03)
United States	70 to 74	1.28<br>(0.87 to 1.68)
United States	75 to 79	0.83<br>(0.34 to 1.33)
United States	80 to 84	0.75<br>(0.15 to 1.35)
United States	85 to 89	1.01<br>(0.23 to 1.8)

United States	90 to 94	1.46<br>(0.27 to 2.65)
United States	95+	1.93<br>(-0.28 to 4.18)

Asthma<br>Location	Age	Prevalence rate (per 100,000 population)
Germany	55 to 59	176.26<br>(164.33 to 189.06)
Germany	60 to 64	149.51<br>(139.37 to 160.38)
Germany	65 to 69	96.52<br>(88.96 to 104.73)
Germany	70 to 74	62.32<br>(57.02 to 68.11)
Germany	75 to 79	40.59<br>(36.69 to 44.91)
Germany	80 to 84	43.02<br>(38.61 to 47.94)
Germany	85 to 89	65.18<br>(57.93 to 73.33)
Germany	90 to 94	85.52<br>(73.85 to 99.04)
Germany	95+	102.85<br>(82.99 to 127.46)
Brazil	55 to 59	175.25<br>(166.44 to 184.52)
Brazil	60 to 64	167.26<br>(158.9 to 176.05)
Brazil	65 to 69	164.91<br>(155.25 to 175.17)
Brazil	70 to 74	141.01<br>(132.24 to 150.36)
Brazil	75 to 79	104.83<br>(97.53 to 112.68)
Brazil	80 to 84	113.25<br>(104.55 to 122.66)
Brazil	85 to 89	155.46<br>(142.09 to 170.09)
Brazil	90 to 94	193.76<br>(172.93 to 217.1)
Brazil	95+	227.49<br>(194.18 to 266.52)
Afghanistan	55 to 59	690.46<br>(664.63 to 717.29)
Afghanistan	60 to 64	705.17<br>(678.56 to 732.83)
Afghanistan	65 to 69	670.69<br>(642.44 to 700.19)
Afghanistan	70 to 74	592.11<br>(565.62 to 619.84)
Afghanistan	75 to 79	479.24<br>(455.01 to 504.77)
Afghanistan	80 to 84	504.38<br>(474.06 to 536.64)
Afghanistan	85 to 89	655.11<br>(604.21 to 710.29)
Afghanistan	90 to 94	800.21<br>(702.17 to 911.93)
Afghanistan	95+	940.54<br>(729.07 to 1213.36)
Japan	55 to 59	229.93<br>(203.53 to 259.76)
Japan	60 to 64	212.82<br>(188.8 to 239.89)

Japan	65 to 69	180.81 (160.39 to 203.83)
Japan	70 to 74	138.13 (121.09 to 157.57)
Japan	75 to 79	103.8 (90.39 to 119.2)
Japan	80 to 84	112.1 (97.33 to 129.12)
Japan	85 to 89	146.46 (126.6 to 169.43)
Japan	90 to 94	174.77 (148.68 to 205.44)
Japan	95+	204.04 (167.62 to 248.38)
Saudi Arabia	55 to 59	255.84 (246.98 to 265.03)
Saudi Arabia	60 to 64	284.85 (274.74 to 295.33)
Saudi Arabia	65 to 69	319.63 (305.36 to 334.57)
Saudi Arabia	70 to 74	309.88 (294.45 to 326.13)
Saudi Arabia	75 to 79	263.7 (247.63 to 280.82)
Saudi Arabia	80 to 84	299.84 (278.31 to 323.03)
Saudi Arabia	85 to 89	411.73 (376.15 to 450.67)
Saudi Arabia	90 to 94	519.44 (455.37 to 592.53)
Saudi Arabia	95+	626.66 (488.19 to 804.41)
China	55 to 59	113.73 (103.64 to 124.8)
China	60 to 64	148.44 (135.55 to 162.56)
China	65 to 69	188.69 (170.29 to 209.08)
China	70 to 74	188.67 (169.78 to 209.65)
China	75 to 79	169.56 (151.77 to 189.44)
China	80 to 84	157.08 (138.96 to 177.55)
China	85 to 89	149.81 (128.54 to 174.6)
China	90 to 94	148.61 (117.05 to 188.68)
China	95+	153.75 (93.33 to 253.26)
Burundi	55 to 59	446.18 (423.17 to 470.44)
Burundi	60 to 64	449.61 (425.87 to 474.67)
Burundi	65 to 69	344.39 (320.38 to 370.19)
Burundi	70 to 74	304.18 (280.29 to 330.1)
Burundi	75 to 79	320.97 (292.51 to 352.2)
Burundi	80 to 84	347.33 (309.78 to 389.44)
Burundi	85 to 89	385.81 (328.87 to 452.62)
Burundi	90 to 94	428.71 (327.72 to 560.83)

Burundi	95+	458.64<br>(265.93 to 791.01)
India	55 to 59	405.95<br>(395.99 to 416.16)
India	60 to 64	464.45<br>(453.15 to 476.03)
India	65 to 69	506.68<br>(491.82 to 521.98)
India	70 to 74	526.16<br>(509.71 to 543.14)
India	75 to 79	528.69<br>(510.44 to 547.59)
India	80 to 84	589.08<br>(565.82 to 613.29)
India	85 to 89	698.86<br>(664.56 to 734.94)
India	90 to 94	790.88<br>(734.92 to 851.11)
India	95+	865.55<br>(760.34 to 985.32)
Russia	55 to 59	232.68<br>(225.45 to 240.15)
Russia	60 to 64	174.55<br>(169.06 to 180.22)
Russia	65 to 69	105.28<br>(101.49 to 109.2)
Russia	70 to 74	57.73<br>(55.4 to 60.17)
Russia	75 to 79	24.47<br>(23.17 to 25.83)
Russia	80 to 84	14.08<br>(13.07 to 15.16)
Russia	85 to 89	17.53<br>(15.99 to 19.23)
Russia	90 to 94	19.52<br>(16.99 to 22.42)
Russia	95+	20.52<br>(15.38 to 27.37)
United Kingdom	55 to 59	231.35<br>(217.58 to 245.99)
United Kingdom	60 to 64	210.08<br>(197.5 to 223.46)
United Kingdom	65 to 69	154.11<br>(143.23 to 165.83)
United Kingdom	70 to 74	122.99<br>(113.7 to 133.05)
United Kingdom	75 to 79	110.49<br>(101.4 to 120.4)
United Kingdom	80 to 84	189.94<br>(174.31 to 206.97)
United Kingdom	85 to 89	398.55<br>(364.71 to 435.53)
United Kingdom	90 to 94	671.93<br>(607.13 to 743.64)
United Kingdom	95+	1004.17<br>(879.5 to 1146.53)
United States	55 to 59	587.57<br>(547.73 to 630.31)
United States	60 to 64	649.28<br>(604.99 to 696.83)
United States	65 to 69	700.44<br>(639.19 to 767.56)
United States	70 to 74	682.82<br>(617.72 to 754.77)
United States	75 to 79	582.51<br>(520.16 to 652.33)

United States	80 to 84	653.92 (576.57 to 741.66)
United States	85 to 89	919.23 (799.61 to 1056.75)
United States	90 to 94	1214.5 (1022.08 to 1443.15)
United States	95+	1560.58 (1211.85 to 2009.65)

Asthma Location	Period	Prevalence rate ratio
Germany	1992 to 1996	1.84 (1.71 to 1.97)
Germany	1997 to 2001	1.41 (1.31 to 1.51)
Germany	2002 to 2006	1.27 (1.18 to 1.36)
Germany	2007 to 2011	1.13 (1.06 to 1.21)
Germany	2012 to 2016	1 (1 to 1)
Germany	2017 to 2021	1.01 (0.94 to 1.08)
Brazil	1992 to 1996	1.54 (1.45 to 1.64)
Brazil	1997 to 2001	1.48 (1.4 to 1.57)
Brazil	2002 to 2006	1.35 (1.28 to 1.42)
Brazil	2007 to 2011	1.09 (1.04 to 1.15)
Brazil	2012 to 2016	1 (1 to 1)
Brazil	2017 to 2021	1.05 (1 to 1.1)
Afghanistan	1992 to 1996	1.15 (1.08 to 1.21)
Afghanistan	1997 to 2001	1.12 (1.07 to 1.18)
Afghanistan	2002 to 2006	1.1 (1.06 to 1.14)
Afghanistan	2007 to 2011	1.05 (1.02 to 1.09)
Afghanistan	2012 to 2016	1 (1 to 1)
Afghanistan	2017 to 2021	0.99 (0.95 to 1.02)
Japan	1992 to 1996	2.69 (2.47 to 2.92)
Japan	1997 to 2001	2 (1.84 to 2.18)
Japan	2002 to 2006	1.32 (1.21 to 1.43)
Japan	2007 to 2011	1 (1 to 1)
Japan	2012 to 2016	0.87 (0.79 to 0.95)
Japan	2017 to 2021	0.87 (0.79 to 0.96)
Saudi Arabia	1992 to 1996	1.16 (1.09 to 1.23)
Saudi Arabia	1997 to 2001	1.1 (1.04 to 1.16)
Saudi Arabia	2002 to 2006	1.08 (1.03 to 1.13)

Saudi Arabia	2007 to 2011	1.05<br>(1.01 to 1.1)
Saudi Arabia	2012 to 2016	1<br>(1 to 1)
Saudi Arabia	2017 to 2021	1<br>(0.96 to 1.04)
China	1992 to 1996	1.73<br>(1.5 to 2)
China	1997 to 2001	1.52<br>(1.35 to 1.71)
China	2002 to 2006	1.27<br>(1.16 to 1.4)
China	2007 to 2011	1.14<br>(1.06 to 1.23)
China	2012 to 2016	1<br>(1 to 1)
China	2017 to 2021	0.96<br>(0.89 to 1.03)
Burundi	1992 to 1996	1.13<br>(0.99 to 1.29)
Burundi	1997 to 2001	1.1<br>(0.98 to 1.22)
Burundi	2002 to 2006	1.06<br>(0.97 to 1.16)
Burundi	2007 to 2011	1.02<br>(0.95 to 1.09)
Burundi	2012 to 2016	1<br>(1 to 1)
Burundi	2017 to 2021	1.03<br>(0.96 to 1.1)
India	1992 to 1996	1.26<br>(1.21 to 1.3)
India	1997 to 2001	1.15<br>(1.11 to 1.19)
India	2002 to 2006	1.14<br>(1.11 to 1.17)
India	2007 to 2011	1.11<br>(1.09 to 1.14)
India	2012 to 2016	1<br>(1 to 1)
India	2017 to 2021	0.95<br>(0.93 to 0.98)
Russia	1992 to 1996	2.58<br>(2.43 to 2.73)
Russia	1997 to 2001	2.17<br>(2.06 to 2.28)
Russia	2002 to 2006	1.65<br>(1.59 to 1.72)
Russia	2007 to 2011	1.29<br>(1.24 to 1.33)
Russia	2012 to 2016	1<br>(1 to 1)
Russia	2017 to 2021	0.99<br>(0.95 to 1.03)
United Kingdom	1992 to 1996	0.93<br>(0.88 to 0.98)
United Kingdom	1997 to 2001	0.82<br>(0.77 to 0.86)
United Kingdom	2002 to 2006	0.82<br>(0.78 to 0.86)
United Kingdom	2007 to 2011	0.89<br>(0.84 to 0.93)
United Kingdom	2012 to 2016	1<br>(1 to 1)
United Kingdom	2017 to 2021	1.14<br>(1.08 to 1.19)

United States	1992 to 1996	0.88 (0.81 to 0.96)
United States	1997 to 2001	0.7 (0.64 to 0.77)
United States	2002 to 2006	0.71 (0.66 to 0.77)
United States	2007 to 2011	0.86 (0.8 to 0.92)
United States	2012 to 2016	1 (1 to 1)
United States	2017 to 2021	1.06 (0.99 to 1.13)

Asthma Location	Cohort	Prevalence rate ratio
Germany	1897 to 1901	3.59 (1.99 to 6.45)
Germany	1902 to 1906	3.66 (2.77 to 4.84)
Germany	1907 to 1911	3.6 (3.02 to 4.3)
Germany	1912 to 1916	3.53 (3.05 to 4.07)
Germany	1917 to 1921	3.28 (2.86 to 3.75)
Germany	1922 to 1926	2.92 (2.62 to 3.26)
Germany	1927 to 1931	2.74 (2.49 to 3.01)
Germany	1932 to 1936	2.55 (2.35 to 2.77)
Germany	1937 to 1941	2.18 (2.02 to 2.35)
Germany	1942 to 1946	1.78 (1.64 to 1.93)
Germany	1947 to 1951	1.48 (1.36 to 1.62)
Germany	1952 to 1956	1.22 (1.12 to 1.34)
Germany	1957 to 1961	1 (1 to 1)
Germany	1962 to 1966	0.83 (0.74 to 0.93)
Brazil	1897 to 1901	3.33 (1.76 to 6.31)
Brazil	1902 to 1906	3.1 (2.34 to 4.12)
Brazil	1907 to 1911	2.92 (2.47 to 3.46)
Brazil	1912 to 1916	2.78 (2.46 to 3.15)
Brazil	1917 to 1921	2.72 (2.46 to 3.01)
Brazil	1922 to 1926	2.69 (2.47 to 2.92)
Brazil	1927 to 1931	2.59 (2.41 to 2.78)
Brazil	1932 to 1936	2.42 (2.27 to 2.58)
Brazil	1937 to 1941	2.16 (2.03 to 2.29)
Brazil	1942 to 1946	1.86 (1.75 to 1.97)
Brazil	1947 to 1951	1.52 (1.42 to 1.61)

Brazil	1952 to 1956	1.21<br>(1.14 to 1.29)
Brazil	1957 to 1961	1<br>(1 to 1)
Brazil	1962 to 1966	0.83<br>(0.76 to 0.9)
Afghanistan	1897 to 1901	1.45<br>(0.74 to 2.83)
Afghanistan	1902 to 1906	1.44<br>(1.09 to 1.89)
Afghanistan	1907 to 1911	1.4<br>(1.2 to 1.63)
Afghanistan	1912 to 1916	1.34<br>(1.21 to 1.49)
Afghanistan	1917 to 1921	1.33<br>(1.23 to 1.43)
Afghanistan	1922 to 1926	1.34<br>(1.26 to 1.42)
Afghanistan	1927 to 1931	1.32<br>(1.26 to 1.39)
Afghanistan	1932 to 1936	1.26<br>(1.21 to 1.32)
Afghanistan	1937 to 1941	1.2<br>(1.15 to 1.25)
Afghanistan	1942 to 1946	1.14<br>(1.1 to 1.19)
Afghanistan	1947 to 1951	1.09<br>(1.04 to 1.14)
Afghanistan	1952 to 1956	1.04<br>(0.99 to 1.08)
Afghanistan	1957 to 1961	1<br>(1 to 1)
Afghanistan	1962 to 1966	0.95<br>(0.9 to 1.01)
Japan	1897 to 1901	6.81<br>(3.32 to 13.99)
Japan	1902 to 1906	7.08<br>(5.05 to 9.92)
Japan	1907 to 1911	7.56<br>(6.08 to 9.4)
Japan	1912 to 1916	8.12<br>(6.84 to 9.65)
Japan	1917 to 1921	8.48<br>(7.27 to 9.88)
Japan	1922 to 1926	7.8<br>(6.77 to 8.98)
Japan	1927 to 1931	6.31<br>(5.54 to 7.19)
Japan	1932 to 1936	4.27<br>(3.76 to 4.85)
Japan	1937 to 1941	2.69<br>(2.37 to 3.05)
Japan	1942 to 1946	1.81<br>(1.59 to 2.07)
Japan	1947 to 1951	1.34<br>(1.16 to 1.54)
Japan	1952 to 1956	1<br>(1 to 1)
Japan	1957 to 1961	0.72<br>(0.59 to 0.87)
Japan	1962 to 1966	0.53<br>(0.4 to 0.69)
Saudi Arabia	1897 to 1901	1.34<br>(0.73 to 2.47)
Saudi Arabia	1902 to 1906	1.38<br>(1.02 to 1.87)

Saudi Arabia	1907 to 1911	1.39<br>(1.16 to 1.66)
Saudi Arabia	1912 to 1916	1.4<br>(1.23 to 1.58)
Saudi Arabia	1917 to 1921	1.4<br>(1.27 to 1.54)
Saudi Arabia	1922 to 1926	1.4<br>(1.3 to 1.51)
Saudi Arabia	1927 to 1931	1.37<br>(1.29 to 1.45)
Saudi Arabia	1932 to 1936	1.31<br>(1.24 to 1.38)
Saudi Arabia	1937 to 1941	1.24<br>(1.19 to 1.3)
Saudi Arabia	1942 to 1946	1.19<br>(1.14 to 1.25)
Saudi Arabia	1947 to 1951	1.13<br>(1.08 to 1.18)
Saudi Arabia	1952 to 1956	1.06<br>(1.02 to 1.11)
Saudi Arabia	1957 to 1961	1<br>(1 to 1)
Saudi Arabia	1962 to 1966	0.93<br>(0.88 to 0.98)
China	1897 to 1901	4.19<br>(0.48 to 36.25)
China	1902 to 1906	4.02<br>(1.95 to 8.25)
China	1907 to 1911	3.7<br>(2.65 to 5.17)
China	1912 to 1916	3.34<br>(2.72 to 4.09)
China	1917 to 1921	3.19<br>(2.75 to 3.7)
China	1922 to 1926	3.21<br>(2.84 to 3.63)
China	1927 to 1931	3.01<br>(2.69 to 3.36)
China	1932 to 1936	2.6<br>(2.34 to 2.89)
China	1937 to 1941	2.19<br>(1.97 to 2.42)
China	1942 to 1946	1.82<br>(1.64 to 2.02)
China	1947 to 1951	1.44<br>(1.29 to 1.6)
China	1952 to 1956	1.16<br>(1.04 to 1.29)
China	1957 to 1961	1<br>(1 to 1)
China	1962 to 1966	0.88<br>(0.76 to 1.03)
Burundi	1897 to 1901	1.27<br>(0.22 to 7.28)
Burundi	1902 to 1906	1.29<br>(0.66 to 2.54)
Burundi	1907 to 1911	1.32<br>(0.94 to 1.86)
Burundi	1912 to 1916	1.32<br>(1.07 to 1.62)
Burundi	1917 to 1921	1.3<br>(1.12 to 1.51)
Burundi	1922 to 1926	1.28<br>(1.14 to 1.44)
Burundi	1927 to 1931	1.26<br>(1.15 to 1.39)

Burundi	1932 to 1936	1.25<br>(1.15 to 1.35)
Burundi	1937 to 1941	1.22<br>(1.14 to 1.3)
Burundi	1942 to 1946	1.17<br>(1.09 to 1.26)
Burundi	1947 to 1951	1.12<br>(1.05 to 1.21)
Burundi	1952 to 1956	1.07<br>(1 to 1.14)
Burundi	1957 to 1961	1<br>(1 to 1)
Burundi	1962 to 1966	0.93<br>(0.86 to 1.02)
India	1897 to 1901	1.78<br>(1.13 to 2.81)
India	1902 to 1906	1.73<br>(1.44 to 2.1)
India	1907 to 1911	1.68<br>(1.52 to 1.85)
India	1912 to 1916	1.61<br>(1.5 to 1.72)
India	1917 to 1921	1.5<br>(1.43 to 1.58)
India	1922 to 1926	1.37<br>(1.31 to 1.43)
India	1927 to 1931	1.27<br>(1.22 to 1.31)
India	1932 to 1936	1.23<br>(1.19 to 1.27)
India	1937 to 1941	1.2<br>(1.17 to 1.24)
India	1942 to 1946	1.15<br>(1.12 to 1.19)
India	1947 to 1951	1.07<br>(1.04 to 1.11)
India	1952 to 1956	1.02<br>(0.99 to 1.05)
India	1957 to 1961	1<br>(1 to 1)
India	1962 to 1966	0.98<br>(0.94 to 1.02)
Russia	1897 to 1901	13.11<br>(6.58 to 26.12)
Russia	1902 to 1906	11.16<br>(8.37 to 14.89)
Russia	1907 to 1911	9.58<br>(8.15 to 11.27)
Russia	1912 to 1916	8.18<br>(7.3 to 9.17)
Russia	1917 to 1921	7.29<br>(6.7 to 7.93)
Russia	1922 to 1926	6.15<br>(5.81 to 6.52)
Russia	1927 to 1931	5.16<br>(4.95 to 5.39)
Russia	1932 to 1936	4.32<br>(4.17 to 4.49)
Russia	1937 to 1941	3.47<br>(3.35 to 3.58)
Russia	1942 to 1946	2.78<br>(2.68 to 2.89)
Russia	1947 to 1951	2<br>(1.92 to 2.08)
Russia	1952 to 1956	1.47<br>(1.41 to 1.53)

Russia	1957 to 1961	1<br>(1 to 1)
Russia	1962 to 1966	0.67<br>(0.63 to 0.71)
United Kingdom	1897 to 1901	0.64<br>(0.42 to 0.97)
United Kingdom	1902 to 1906	0.73<br>(0.6 to 0.9)
United Kingdom	1907 to 1911	0.84<br>(0.73 to 0.96)
United Kingdom	1912 to 1916	0.94<br>(0.84 to 1.06)
United Kingdom	1917 to 1921	1.08<br>(0.97 to 1.19)
United Kingdom	1922 to 1926	1.24<br>(1.13 to 1.36)
United Kingdom	1927 to 1931	1.45<br>(1.34 to 1.58)
United Kingdom	1932 to 1936	1.64<br>(1.52 to 1.76)
United Kingdom	1937 to 1941	1.69<br>(1.58 to 1.82)
United Kingdom	1942 to 1946	1.62<br>(1.51 to 1.74)
United Kingdom	1947 to 1951	1.48<br>(1.37 to 1.59)
United Kingdom	1952 to 1956	1.25<br>(1.16 to 1.35)
United Kingdom	1957 to 1961	1<br>(1 to 1)
United Kingdom	1962 to 1966	0.86<br>(0.78 to 0.95)
United States	1897 to 1901	0.42<br>(0.2 to 0.86)
United States	1902 to 1906	0.47<br>(0.32 to 0.69)
United States	1907 to 1911	0.53<br>(0.42 to 0.68)
United States	1912 to 1916	0.59<br>(0.49 to 0.72)
United States	1917 to 1921	0.64<br>(0.55 to 0.75)
United States	1922 to 1926	0.66<br>(0.58 to 0.75)
United States	1927 to 1931	0.68<br>(0.6 to 0.76)
United States	1932 to 1936	0.7<br>(0.63 to 0.77)
United States	1937 to 1941	0.73<br>(0.67 to 0.81)
United States	1942 to 1946	0.8<br>(0.73 to 0.89)
United States	1947 to 1951	0.92<br>(0.84 to 1.01)
United States	1952 to 1956	1.01<br>(0.92 to 1.09)
United States	1957 to 1961	1<br>(1 to 1)
United States	1962 to 1966	0.96<br>(0.85 to 1.08)

Table S20 APC Model Analysis Results of ILD&PS Incidence Among Adults Aged 55 and Above in 11 Example Countries

ILD&PS Location	Age	Local drift (%/year)
Germany	55 to 59	0.29 (-0.03 to 0.61)
Germany	60 to 64	1.08 (0.83 to 1.32)
Germany	65 to 69	1.86 (1.63 to 2.09)
Germany	70 to 74	2.51 (2.26 to 2.76)
Germany	75 to 79	2.66 (2.34 to 2.97)
Germany	80 to 84	2.36 (1.98 to 2.74)
Germany	85 to 89	1.83 (1.33 to 2.34)
Germany	90 to 94	1.27 (0.41 to 2.14)
Germany	95+	1.06 (-0.85 to 3.01)
Brazil	55 to 59	-0.32 (-0.69 to 0.05)
Brazil	60 to 64	0.03 (-0.27 to 0.32)
Brazil	65 to 69	0.48 (0.2 to 0.75)
Brazil	70 to 74	0.91 (0.63 to 1.2)
Brazil	75 to 79	1.23 (0.91 to 1.54)
Brazil	80 to 84	1.4 (1.02 to 1.78)
Brazil	85 to 89	1.51 (0.99 to 2.04)
Brazil	90 to 94	1.62 (0.72 to 2.53)
Brazil	95+	1.7 (-0.4 to 3.85)
Afghanistan	55 to 59	0.95 (-1.21 to 3.16)
Afghanistan	60 to 64	0.67 (-1.07 to 2.44)
Afghanistan	65 to 69	0.29 (-1.23 to 1.84)
Afghanistan	70 to 74	0.08 (-1.53 to 1.72)
Afghanistan	75 to 79	-0.1 (-2 to 1.84)
Afghanistan	80 to 84	-0.18 (-2.65 to 2.35)
Afghanistan	85 to 89	-0.33 (-3.86 to 3.33)
Afghanistan	90 to 94	-0.53 (-6.83 to 6.18)
Afghanistan	95+	-1.44 (-19.37 to 20.48)
Japan	55 to 59	0.08 (-0.3 to 0.46)
Japan	60 to 64	0.4 (0.14 to 0.67)
Japan	65 to 69	0.88 (0.65 to 1.1)
Japan	70 to 74	1.53 (1.28 to 1.79)

Japan	75 to 79	2.06<br>(1.72 to 2.4)
Japan	80 to 84	2.26<br>(1.79 to 2.73)
Japan	85 to 89	2.04<br>(1.32 to 2.77)
Japan	90 to 94	1.57<br>(0.25 to 2.91)
Japan	95+	1.14<br>(-1.98 to 4.36)
Saudi Arabia	55 to 59	2.03<br>(1.29 to 2.77)
Saudi Arabia	60 to 64	1.85<br>(1.22 to 2.5)
Saudi Arabia	65 to 69	1.62<br>(0.96 to 2.28)
Saudi Arabia	70 to 74	1.33<br>(0.56 to 2.11)
Saudi Arabia	75 to 79	1.09<br>(0.13 to 2.05)
Saudi Arabia	80 to 84	0.98<br>(-0.21 to 2.2)
Saudi Arabia	85 to 89	0.95<br>(-0.68 to 2.61)
Saudi Arabia	90 to 94	0.85<br>(-1.72 to 3.49)
Saudi Arabia	95+	0.52<br>(-4.12 to 5.38)
China	55 to 59	1.77<br>(1.61 to 1.93)
China	60 to 64	2.33<br>(2.2 to 2.46)
China	65 to 69	2.83<br>(2.69 to 2.97)
China	70 to 74	3.22<br>(3.05 to 3.4)
China	75 to 79	3.31<br>(3.08 to 3.54)
China	80 to 84	3.03<br>(2.71 to 3.35)
China	85 to 89	2.53<br>(2.01 to 3.05)
China	90 to 94	2.01<br>(0.94 to 3.09)
China	95+	1.62<br>(-1.47 to 4.81)
Burundi	55 to 59	0.33<br>(-2.79 to 3.55)
Burundi	60 to 64	0.4<br>(-2.14 to 3.02)
Burundi	65 to 69	0.31<br>(-2.2 to 2.89)
Burundi	70 to 74	0.01<br>(-2.7 to 2.81)
Burundi	75 to 79	-0.11<br>(-3.3 to 3.19)
Burundi	80 to 84	-0.36<br>(-4.49 to 3.96)
Burundi	85 to 89	-1.04<br>(-7.34 to 5.69)
Burundi	90 to 94	-1.74<br>(-15.18 to 13.83)
Burundi	95+	-3.1<br>(-22.73 to 21.51)
India	55 to 59	0.11<br>(0.01 to 0.21)

India	60 to 64	0.23<br>(0.16 to 0.31)
India	65 to 69	0.3<br>(0.23 to 0.38)
India	70 to 74	0.33<br>(0.25 to 0.41)
India	75 to 79	0.34<br>(0.24 to 0.43)
India	80 to 84	0.36<br>(0.23 to 0.49)
India	85 to 89	0.44<br>(0.24 to 0.63)
India	90 to 94	0.56<br>(0.19 to 0.93)
India	95+	0.72<br>(-0.15 to 1.59)
Russia	55 to 59	-2.35<br>(-2.94 to -1.76)
Russia	60 to 64	-2.24<br>(-2.71 to -1.78)
Russia	65 to 69	-2.13<br>(-2.62 to -1.62)
Russia	70 to 74	-1.99<br>(-2.6 to -1.37)
Russia	75 to 79	-2.02<br>(-2.72 to -1.32)
Russia	80 to 84	-2.07<br>(-2.82 to -1.31)
Russia	85 to 89	-2.03<br>(-2.98 to -1.06)
Russia	90 to 94	-1.92<br>(-3.5 to -0.31)
Russia	95+	-1.81<br>(-5.37 to 1.89)
United Kingdom	55 to 59	0.73<br>(0.45 to 1.01)
United Kingdom	60 to 64	1.15<br>(0.94 to 1.36)
United Kingdom	65 to 69	1.78<br>(1.6 to 1.97)
United Kingdom	70 to 74	2.45<br>(2.27 to 2.63)
United Kingdom	75 to 79	2.96<br>(2.75 to 3.16)
United Kingdom	80 to 84	3.3<br>(3.06 to 3.55)
United Kingdom	85 to 89	3.47<br>(3.14 to 3.8)
United Kingdom	90 to 94	3.47<br>(2.92 to 4.03)
United Kingdom	95+	3.34<br>(2.1 to 4.59)
United States	55 to 59	0.29<br>(-0.1 to 0.69)
United States	60 to 64	0.43<br>(0.14 to 0.73)
United States	65 to 69	0.69<br>(0.43 to 0.95)
United States	70 to 74	1.12<br>(0.87 to 1.37)
United States	75 to 79	1.64<br>(1.36 to 1.92)
United States	80 to 84	2.11<br>(1.76 to 2.47)
United States	85 to 89	2.42<br>(1.91 to 2.94)

United States	90 to 94	2.6<br>(1.74 to 3.46)
United States	95+	2.61<br>(0.84 to 4.4)

ILD&PS<br>Location	Age	Prevalence rate (per 100,000 population)
Germany	55 to 59	9.96<br>(9.38 to 10.58)
Germany	60 to 64	13.81<br>(13.04 to 14.62)
Germany	65 to 69	17.68<br>(16.51 to 18.92)
Germany	70 to 74	20.98<br>(19.56 to 22.5)
Germany	75 to 79	20.38<br>(18.92 to 21.94)
Germany	80 to 84	20.1<br>(18.52 to 21.81)
Germany	85 to 89	24.91<br>(22.67 to 27.37)
Germany	90 to 94	29.49<br>(26.03 to 33.4)
Germany	95+	34.95<br>(28.71 to 42.56)
Brazil	55 to 59	5.2<br>(4.85 to 5.56)
Brazil	60 to 64	7.1<br>(6.65 to 7.57)
Brazil	65 to 69	9.24<br>(8.54 to 10.01)
Brazil	70 to 74	13.07<br>(12.04 to 14.19)
Brazil	75 to 79	17.31<br>(15.88 to 18.87)
Brazil	80 to 84	23.85<br>(21.76 to 26.14)
Brazil	85 to 89	34.85<br>(31.53 to 38.53)
Brazil	90 to 94	47.28<br>(42.01 to 53.2)
Brazil	95+	59.97<br>(51.49 to 69.84)
Afghanistan	55 to 59	4.37<br>(2.75 to 6.94)
Afghanistan	60 to 64	5.53<br>(3.49 to 8.76)
Afghanistan	65 to 69	6.61<br>(3.94 to 11.09)
Afghanistan	70 to 74	8.61<br>(5.07 to 14.64)
Afghanistan	75 to 79	10.35<br>(5.93 to 18.07)
Afghanistan	80 to 84	13.5<br>(7.37 to 24.72)
Afghanistan	85 to 89	19.24<br>(9.45 to 39.19)
Afghanistan	90 to 94	22.28<br>(7.73 to 64.2)
Afghanistan	95+	10.58<br>(0.6 to 187.47)
Japan	55 to 59	36.38<br>(33.93 to 39.02)
Japan	60 to 64	57.03<br>(53.37 to 60.95)

Japan	65 to 69	72.63<br>(67.36 to 78.32)
Japan	70 to 74	79.06<br>(73.16 to 85.43)
Japan	75 to 79	70.07<br>(64.54 to 76.08)
Japan	80 to 84	59.49<br>(54.31 to 65.16)
Japan	85 to 89	59.45<br>(53.49 to 66.09)
Japan	90 to 94	58.84<br>(51.13 to 67.72)
Japan	95+	58.13<br>(46.89 to 72.08)
Saudi Arabia	55 to 59	24.55<br>(21.94 to 27.47)
Saudi Arabia	60 to 64	34.67<br>(30.94 to 38.85)
Saudi Arabia	65 to 69	43.95<br>(37.73 to 51.19)
Saudi Arabia	70 to 74	54.57<br>(46.02 to 64.7)
Saudi Arabia	75 to 79	61.35<br>(50.21 to 74.98)
Saudi Arabia	80 to 84	86.25<br>(68.78 to 108.16)
Saudi Arabia	85 to 89	137.98<br>(106.09 to 179.46)
Saudi Arabia	90 to 94	194.78<br>(135.95 to 279.06)
Saudi Arabia	95+	266.31<br>(140.38 to 505.21)
China	55 to 59	7.35<br>(7.17 to 7.53)
China	60 to 64	9.6<br>(9.36 to 9.84)
China	65 to 69	10.76<br>(10.42 to 11.11)
China	70 to 74	11.75<br>(11.34 to 12.17)
China	75 to 79	13.03<br>(12.53 to 13.55)
China	80 to 84	17.25<br>(16.48 to 18.05)
China	85 to 89	25.03<br>(23.62 to 26.52)
China	90 to 94	32.2<br>(29.37 to 35.29)
China	95+	38.21<br>(31.44 to 46.44)
Burundi	55 to 59	3.88<br>(2.25 to 6.71)
Burundi	60 to 64	4.89<br>(2.85 to 8.39)
Burundi	65 to 69	5.86<br>(2.92 to 11.74)
Burundi	70 to 74	7.9<br>(3.8 to 16.39)
Burundi	75 to 79	9.68<br>(4.45 to 21.06)
Burundi	80 to 84	12.13<br>(5.06 to 29.05)
Burundi	85 to 89	13.54<br>(4.39 to 41.8)
Burundi	90 to 94	5.35<br>(0.4 to 70.79)

Burundi	95+	19.67<br>(0.9 to 430.34)
India	55 to 59	15.61<br>(15.33 to 15.89)
India	60 to 64	21.54<br>(21.17 to 21.91)
India	65 to 69	27.22<br>(26.65 to 27.79)
India	70 to 74	35.7<br>(34.93 to 36.49)
India	75 to 79	44.25<br>(43.23 to 45.29)
India	80 to 84	53.7<br>(52.33 to 55.1)
India	85 to 89	67.48<br>(65.42 to 69.61)
India	90 to 94	82.31<br>(78.82 to 85.95)
India	95+	98.56<br>(91.51 to 106.16)
Russia	55 to 59	1.7<br>(1.51 to 1.92)
Russia	60 to 64	1.51<br>(1.34 to 1.7)
Russia	65 to 69	1.18<br>(1.03 to 1.37)
Russia	70 to 74	0.95<br>(0.82 to 1.12)
Russia	75 to 79	0.84<br>(0.71 to 1)
Russia	80 to 84	1.14<br>(0.95 to 1.36)
Russia	85 to 89	1.71<br>(1.4 to 2.09)
Russia	90 to 94	2.17<br>(1.66 to 2.84)
Russia	95+	2.55<br>(1.56 to 4.17)
United Kingdom	55 to 59	19.83<br>(18.82 to 20.89)
United Kingdom	60 to 64	30.33<br>(28.87 to 31.86)
United Kingdom	65 to 69	42.24<br>(39.85 to 44.76)
United Kingdom	70 to 74	56.7<br>(53.42 to 60.17)
United Kingdom	75 to 79	67.28<br>(63.24 to 71.58)
United Kingdom	80 to 84	84.88<br>(79.53 to 90.58)
United Kingdom	85 to 89	120.84<br>(112.7 to 129.57)
United Kingdom	90 to 94	163.93<br>(151.15 to 177.78)
United Kingdom	95+	217.1<br>(194.25 to 242.64)
United States	55 to 59	20.39<br>(18.98 to 21.9)
United States	60 to 64	31.22<br>(29.23 to 33.35)
United States	65 to 69	46<br>(42.45 to 49.86)
United States	70 to 74	66.11<br>(60.87 to 71.79)
United States	75 to 79	81.24<br>(74.58 to 88.51)

United States	80 to 84	88.66<br>(80.97 to 97.09)
United States	85 to 89	98.72<br>(89.32 to 109.1)
United States	90 to 94	110.96<br>(98.27 to 125.3)
United States	95+	126.19<br>(105.42 to 151.05)

ILD&PS<br>Location	Period	Prevalence rate ratio
Germany	1992 to 1996	0.74<br>(0.7 to 0.79)
Germany	1997 to 2001	0.73<br>(0.69 to 0.78)
Germany	2002 to 2006	0.76<br>(0.72 to 0.8)
Germany	2007 to 2011	0.86<br>(0.82 to 0.9)
Germany	2012 to 2016	1<br>(1 to 1)
Germany	2017 to 2021	1.13<br>(1.08 to 1.18)
Brazil	1992 to 1996	0.82<br>(0.76 to 0.88)
Brazil	1997 to 2001	0.89<br>(0.83 to 0.94)
Brazil	2002 to 2006	0.94<br>(0.89 to 0.99)
Brazil	2007 to 2011	0.95<br>(0.9 to 1)
Brazil	2012 to 2016	1<br>(1 to 1)
Brazil	2017 to 2021	1.07<br>(1.02 to 1.12)
Afghanistan	1992 to 1996	0.98<br>(0.59 to 1.63)
Afghanistan	1997 to 2001	0.99<br>(0.63 to 1.56)
Afghanistan	2002 to 2006	1.02<br>(0.69 to 1.51)
Afghanistan	2007 to 2011	1.03<br>(0.74 to 1.45)
Afghanistan	2012 to 2016	1<br>(1 to 1)
Afghanistan	2017 to 2021	0.95<br>(0.67 to 1.35)
Japan	1992 to 1996	0.73<br>(0.67 to 0.79)
Japan	1997 to 2001	0.81<br>(0.76 to 0.87)
Japan	2002 to 2006	0.89<br>(0.84 to 0.94)
Japan	2007 to 2011	0.96<br>(0.92 to 1.01)
Japan	2012 to 2016	1<br>(1 to 1)
Japan	2017 to 2021	1.04<br>(0.99 to 1.09)
Saudi Arabia	1992 to 1996	0.74<br>(0.62 to 0.89)
Saudi Arabia	1997 to 2001	0.82<br>(0.69 to 0.96)
Saudi Arabia	2002 to 2006	0.88<br>(0.76 to 1.02)

Saudi Arabia	2007 to 2011	0.95<br>(0.84 to 1.08)
Saudi Arabia	2012 to 2016	1<br>(1 to 1)
Saudi Arabia	2017 to 2021	0.99<br>(0.88 to 1.11)
China	1992 to 1996	0.6<br>(0.56 to 0.64)
China	1997 to 2001	0.58<br>(0.55 to 0.61)
China	2002 to 2006	0.61<br>(0.58 to 0.63)
China	2007 to 2011	0.93<br>(0.9 to 0.96)
China	2012 to 2016	1<br>(1 to 1)
China	2017 to 2021	0.98<br>(0.96 to 1.01)
Burundi	1992 to 1996	1.11<br>(0.5 to 2.46)
Burundi	1997 to 2001	1.1<br>(0.53 to 2.29)
Burundi	2002 to 2006	1.08<br>(0.57 to 2.05)
Burundi	2007 to 2011	1.03<br>(0.59 to 1.78)
Burundi	2012 to 2016	1<br>(1 to 1)
Burundi	2017 to 2021	0.99<br>(0.59 to 1.66)
India	1992 to 1996	0.94<br>(0.92 to 0.96)
India	1997 to 2001	0.94<br>(0.92 to 0.96)
India	2002 to 2006	0.95<br>(0.93 to 0.97)
India	2007 to 2011	0.98<br>(0.96 to 0.99)
India	2012 to 2016	1<br>(1 to 1)
India	2017 to 2021	1.02<br>(1.01 to 1.04)
Russia	1992 to 1996	1.61<br>(1.42 to 1.84)
Russia	1997 to 2001	1.46<br>(1.28 to 1.66)
Russia	2002 to 2006	1.24<br>(1.09 to 1.4)
Russia	2007 to 2011	1.07<br>(0.95 to 1.2)
Russia	2012 to 2016	1<br>(1 to 1)
Russia	2017 to 2021	1.01<br>(0.9 to 1.13)
United Kingdom	1992 to 1996	0.6<br>(0.58 to 0.63)
United Kingdom	1997 to 2001	0.67<br>(0.64 to 0.7)
United Kingdom	2002 to 2006	0.76<br>(0.73 to 0.79)
United Kingdom	2007 to 2011	0.9<br>(0.87 to 0.93)
United Kingdom	2012 to 2016	1<br>(1 to 1)
United Kingdom	2017 to 2021	1.12<br>(1.08 to 1.15)

United States	1992 to 1996	0.71<br>(0.67 to 0.76)
United States	1997 to 2001	0.86<br>(0.81 to 0.91)
United States	2002 to 2006	0.9<br>(0.85 to 0.95)
United States	2007 to 2011	0.95<br>(0.9 to 1)
United States	2012 to 2016	1<br>(1 to 1)
United States	2017 to 2021	1.1<br>(1.05 to 1.15)

ILD&PS<br>Location	Cohort	Prevalence rate ratio
Germany	1897 to 1901	0.42<br>(0.22 to 0.79)
Germany	1902 to 1906	0.46<br>(0.35 to 0.61)
Germany	1907 to 1911	0.48<br>(0.41 to 0.57)
Germany	1912 to 1916	0.5<br>(0.44 to 0.57)
Germany	1917 to 1921	0.52<br>(0.47 to 0.58)
Germany	1922 to 1926	0.56<br>(0.51 to 0.61)
Germany	1927 to 1931	0.65<br>(0.6 to 0.71)
Germany	1932 to 1936	0.77<br>(0.71 to 0.82)
Germany	1937 to 1941	0.87<br>(0.82 to 0.93)
Germany	1942 to 1946	0.96<br>(0.9 to 1.03)
Germany	1947 to 1951	1.02<br>(0.95 to 1.1)
Germany	1952 to 1956	1.02<br>(0.96 to 1.1)
Germany	1957 to 1961	1<br>(1 to 1)
Germany	1962 to 1966	0.94<br>(0.86 to 1.04)
Brazil	1897 to 1901	0.56<br>(0.28 to 1.14)
Brazil	1902 to 1906	0.61<br>(0.45 to 0.83)
Brazil	1907 to 1911	0.67<br>(0.57 to 0.8)
Brazil	1912 to 1916	0.74<br>(0.65 to 0.84)
Brazil	1917 to 1921	0.8<br>(0.71 to 0.89)
Brazil	1922 to 1926	0.86<br>(0.77 to 0.95)
Brazil	1927 to 1931	0.92<br>(0.84 to 1.01)
Brazil	1932 to 1936	0.98<br>(0.9 to 1.07)
Brazil	1937 to 1941	1.04<br>(0.96 to 1.13)
Brazil	1942 to 1946	1.07<br>(0.99 to 1.16)
Brazil	1947 to 1951	1.06<br>(0.98 to 1.15)

Brazil	1952 to 1956	1.03<br>(0.95 to 1.12)
Brazil	1957 to 1961	1<br>(1 to 1)
Brazil	1962 to 1966	0.97<br>(0.87 to 1.08)
Afghanistan	1897 to 1901	1.31<br>(0 to 1215.56)
Afghanistan	1902 to 1906	1<br>(0.12 to 8.52)
Afghanistan	1907 to 1911	0.93<br>(0.28 to 3.06)
Afghanistan	1912 to 1916	0.88<br>(0.37 to 2.12)
Afghanistan	1917 to 1921	0.88<br>(0.43 to 1.8)
Afghanistan	1922 to 1926	0.86<br>(0.46 to 1.59)
Afghanistan	1927 to 1931	0.87<br>(0.49 to 1.54)
Afghanistan	1932 to 1936	0.84<br>(0.49 to 1.44)
Afghanistan	1937 to 1941	0.85<br>(0.51 to 1.42)
Afghanistan	1942 to 1946	0.86<br>(0.51 to 1.45)
Afghanistan	1947 to 1951	0.88<br>(0.52 to 1.49)
Afghanistan	1952 to 1956	0.93<br>(0.55 to 1.6)
Afghanistan	1957 to 1961	1<br>(1 to 1)
Afghanistan	1962 to 1966	1.07<br>(0.52 to 2.2)
Japan	1897 to 1901	0.5<br>(0.17 to 1.44)
Japan	1902 to 1906	0.53<br>(0.34 to 0.82)
Japan	1907 to 1911	0.55<br>(0.44 to 0.7)
Japan	1912 to 1916	0.57<br>(0.49 to 0.67)
Japan	1917 to 1921	0.61<br>(0.54 to 0.69)
Japan	1922 to 1926	0.68<br>(0.61 to 0.75)
Japan	1927 to 1931	0.8<br>(0.73 to 0.87)
Japan	1932 to 1936	0.9<br>(0.83 to 0.97)
Japan	1937 to 1941	0.96<br>(0.89 to 1.04)
Japan	1942 to 1946	0.99<br>(0.91 to 1.07)
Japan	1947 to 1951	1.01<br>(0.93 to 1.08)
Japan	1952 to 1956	1.01<br>(0.93 to 1.09)
Japan	1957 to 1961	1<br>(1 to 1)
Japan	1962 to 1966	0.98<br>(0.86 to 1.11)
Saudi Arabia	1897 to 1901	0.52<br>(0.11 to 2.42)
Saudi Arabia	1902 to 1906	0.49<br>(0.22 to 1.13)

Saudi Arabia	1907 to 1911	0.5<br>(0.3 to 0.83)
Saudi Arabia	1912 to 1916	0.52<br>(0.36 to 0.76)
Saudi Arabia	1917 to 1921	0.56<br>(0.41 to 0.75)
Saudi Arabia	1922 to 1926	0.58<br>(0.45 to 0.74)
Saudi Arabia	1927 to 1931	0.6<br>(0.49 to 0.73)
Saudi Arabia	1932 to 1936	0.63<br>(0.53 to 0.76)
Saudi Arabia	1937 to 1941	0.68<br>(0.57 to 0.8)
Saudi Arabia	1942 to 1946	0.73<br>(0.62 to 0.86)
Saudi Arabia	1947 to 1951	0.8<br>(0.69 to 0.93)
Saudi Arabia	1952 to 1956	0.9<br>(0.78 to 1.03)
Saudi Arabia	1957 to 1961	1<br>(1 to 1)
Saudi Arabia	1962 to 1966	1.11<br>(0.93 to 1.32)
China	1897 to 1901	0.26<br>(0.09 to 0.75)
China	1902 to 1906	0.28<br>(0.2 to 0.4)
China	1907 to 1911	0.3<br>(0.25 to 0.35)
China	1912 to 1916	0.32<br>(0.29 to 0.36)
China	1917 to 1921	0.35<br>(0.32 to 0.37)
China	1922 to 1926	0.39<br>(0.37 to 0.42)
China	1927 to 1931	0.47<br>(0.45 to 0.49)
China	1932 to 1936	0.56<br>(0.54 to 0.58)
China	1937 to 1941	0.66<br>(0.64 to 0.69)
China	1942 to 1946	0.76<br>(0.74 to 0.79)
China	1947 to 1951	0.86<br>(0.83 to 0.89)
China	1952 to 1956	0.93<br>(0.9 to 0.96)
China	1957 to 1961	1<br>(1 to 1)
China	1962 to 1966	1.03<br>(0.99 to 1.07)
Burundi	1897 to 1901	2.17<br>(0 to 2227.1)
Burundi	1902 to 1906	1.47<br>(0.01 to 196.08)
Burundi	1907 to 1911	1.29<br>(0.16 to 10.58)
Burundi	1912 to 1916	1.02<br>(0.26 to 4.05)
Burundi	1917 to 1921	0.95<br>(0.32 to 2.77)
Burundi	1922 to 1926	0.98<br>(0.39 to 2.45)
Burundi	1927 to 1931	0.95<br>(0.41 to 2.19)

Burundi	1932 to 1936	0.93<br>(0.43 to 1.97)
Burundi	1937 to 1941	0.92<br>(0.46 to 1.85)
Burundi	1942 to 1946	0.95<br>(0.47 to 1.93)
Burundi	1947 to 1951	0.98<br>(0.49 to 1.97)
Burundi	1952 to 1956	1.01<br>(0.52 to 1.97)
Burundi	1957 to 1961	1<br>(1 to 1)
Burundi	1962 to 1966	1<br>(0.42 to 2.37)
India	1897 to 1901	0.77<br>(0.57 to 1.03)
India	1902 to 1906	0.81<br>(0.72 to 0.92)
India	1907 to 1911	0.85<br>(0.8 to 0.9)
India	1912 to 1916	0.88<br>(0.84 to 0.92)
India	1917 to 1921	0.9<br>(0.87 to 0.93)
India	1922 to 1926	0.92<br>(0.89 to 0.95)
India	1927 to 1931	0.94<br>(0.91 to 0.96)
India	1932 to 1936	0.95<br>(0.93 to 0.97)
India	1937 to 1941	0.96<br>(0.94 to 0.98)
India	1942 to 1946	0.98<br>(0.96 to 1)
India	1947 to 1951	1<br>(0.98 to 1.02)
India	1952 to 1956	1<br>(0.98 to 1.02)
India	1957 to 1961	1<br>(1 to 1)
India	1962 to 1966	0.99<br>(0.96 to 1.02)
Russia	1897 to 1901	3.38<br>(0.98 to 11.65)
Russia	1902 to 1906	3.07<br>(1.79 to 5.25)
Russia	1907 to 1911	2.91<br>(2.11 to 4.01)
Russia	1912 to 1916	2.68<br>(2.1 to 3.43)
Russia	1917 to 1921	2.41<br>(1.93 to 3.02)
Russia	1922 to 1926	2.1<br>(1.73 to 2.55)
Russia	1927 to 1931	1.94<br>(1.65 to 2.28)
Russia	1932 to 1936	1.78<br>(1.54 to 2.06)
Russia	1937 to 1941	1.58<br>(1.38 to 1.8)
Russia	1942 to 1946	1.43<br>(1.23 to 1.65)
Russia	1947 to 1951	1.28<br>(1.1 to 1.49)
Russia	1952 to 1956	1.14<br>(0.98 to 1.32)

Russia	1957 to 1961	1<br>(1 to 1)
Russia	1962 to 1966	0.87<br>(0.71 to 1.06)
United Kingdom	1897 to 1901	0.24<br>(0.16 to 0.36)
United Kingdom	1902 to 1906	0.27<br>(0.23 to 0.33)
United Kingdom	1907 to 1911	0.32<br>(0.29 to 0.36)
United Kingdom	1912 to 1916	0.38<br>(0.35 to 0.41)
United Kingdom	1917 to 1921	0.45<br>(0.42 to 0.49)
United Kingdom	1922 to 1926	0.54<br>(0.5 to 0.57)
United Kingdom	1927 to 1931	0.64<br>(0.6 to 0.68)
United Kingdom	1932 to 1936	0.75<br>(0.7 to 0.79)
United Kingdom	1937 to 1941	0.84<br>(0.8 to 0.9)
United Kingdom	1942 to 1946	0.92<br>(0.87 to 0.98)
United Kingdom	1947 to 1951	0.98<br>(0.93 to 1.04)
United Kingdom	1952 to 1956	0.99<br>(0.93 to 1.05)
United Kingdom	1957 to 1961	1<br>(1 to 1)
United Kingdom	1962 to 1966	1.04<br>(0.95 to 1.13)
United States	1897 to 1901	0.38<br>(0.22 to 0.69)
United States	1902 to 1906	0.43<br>(0.33 to 0.57)
United States	1907 to 1911	0.49<br>(0.41 to 0.58)
United States	1912 to 1916	0.56<br>(0.49 to 0.63)
United States	1917 to 1921	0.64<br>(0.58 to 0.71)
United States	1922 to 1926	0.73<br>(0.66 to 0.8)
United States	1927 to 1931	0.81<br>(0.74 to 0.89)
United States	1932 to 1936	0.88<br>(0.81 to 0.96)
United States	1937 to 1941	0.94<br>(0.86 to 1.02)
United States	1942 to 1946	0.96<br>(0.88 to 1.04)
United States	1947 to 1951	0.97<br>(0.89 to 1.05)
United States	1952 to 1956	0.98<br>(0.9 to 1.06)
United States	1957 to 1961	1<br>(1 to 1)
United States	1962 to 1966	1.01<br>(0.89 to 1.13)

Table S21 Global Burden Projection of Chronic Respiratory Diseases in Adults Aged 55 and Above, 2022-2031

Global Burden Projection of Chronic Respiratory Diseases in Adults Aged 55 and Above, 2022-2031				
Cause	Measure	Rate/100,000		
		2022 (95%UI)	2031 (95%UI)	% change
CRDs				
	Prevalence	14501.76 (14260.15 to 14743.36)	13984.24 (11966.21 to 16002.27)	-0.036
	Incidence	1197.49 (1170.18 to 1224.80)	1177.89 (983.96 to 1371.82)	-0.016
	Deaths	258.59 (252.59 to 264.59)	226.19 (179.76 to 272.62)	-0.125
COPD				
	Prevalence	10845.26 (10716.96 to 10973.56)	10502.51 (9238.78 to 11766.25)	-0.032
	Incidence	860.51 (848.80 to 872.23)	865.36 (752.63 to 978.09)	0.006
	Deaths	223.05 (217.78 to 228.32)	198.28 (156.52 to 240.05)	-0.111
Asthma				
	Prevalence	4149.11 (4042.06 to 4256.16)	4124.99 (3228.85 to 5021.14)	-0.006
	Incidence	330.75 (319.19 to 342.30)	330.29 (253.22 to 407.37)	-0.001
	Deaths	21.81 (20.99 to 22.62)	16.46 (10.54 to 22.38)	-0.245
ILD & PS				
	Prevalence	214.89 (209.54 to 220.25)	202.62 (160.56 to 244.67)	-0.057
	Incidence	18.21 (17.61 to 18.82)	16.91 (13.16 to 20.65)	-0.072
	Deaths	10.81 (10.52 to 11.10)	9.19 (6.95 to 11.43)	-0.150

CRDs = Chronic Respiratory Diseases; COPD = Chronic Obstructive Pulmonary Disease; ILD & PS = Interstitial lung disease and pulmonary sarcoidosis.

Table S22 Burden of DALYs Attributed to Risk Factors for CRDs, COPD, and Asthma

Cause	Risk factor	DALY_YEAR/1000 (95% UI)			DALY_RATE/100,000 (95% UI)		
		Male	Female	Both	Male	Female	Both
CRDs	Environmental/occupational risks	24551.48 (20776.73 to 28739.52)	17607.02 (14702.23 to 20920.62)	42158.5 (36518.29 to 48745.99)	3509.77 (2970.15 to 4108.47)	2238.74 (1869.4 to 2660.07)	2837.07 (2457.51 to 3280.38)
	Air pollution	18483.7 (14604.67 to 22667.71)	14580.43 (11729.99 to 17875.5)	33064.13 (26736.14 to 39601.45)	2642.35 (2087.82 to 3240.48)	1853.91 (1491.48 to 2272.88)	2225.06 (1799.22 to 2664.99)
	Particulate matter pollution	16179.68 (12570.34 to 20605.3)	12961.1 (10017.35 to 16211.28)	29140.78 (23325.21 to 36295.63)	2312.98 (1797 to 2945.64)	1648.01 (1273.71 to 2061.28)	1961.04 (1569.68 to 2442.53)
	Ambient particulate matter pollution	9241.88 (6762.31 to 11452.99)	6461.18 (4475.46 to 8348.67)	15703.06 (11283.31 to 19331.56)	1321.18 (966.71 to 1637.27)	821.54 (569.06 to 1061.54)	1056.74 (759.31 to 1300.92)
	Household air pollution from solid fuels	6935.22 (4140.65 to 12170.46)	6498.23 (3879.86 to 10587.67)	13433.44 (8082.08 to 22299.04)	991.43 (591.93 to 1739.84)	826.25 (493.33 to 1346.23)	904.01 (543.89 to 1500.62)
	Ambient ozone pollution	4610.6 (928.7 to 7954.12)	3382.01 (764.71 to 5752.86)	7992.61 (1749.61 to 13654.4)	659.11 (132.76 to 1137.09)	430.02 (97.23 to 731.48)	537.87 (117.74 to 918.88)
	Non-optimal temperature	3049 (2102.34 to 4204.38)	2141.83 (1455.4 to 3049.21)	5190.83 (3632.34 to 7146.95)	435.87 (300.54 to 601.04)	272.34 (185.05 to 387.71)	349.32 (244.44 to 480.96)
	High temperature	369.13 (-61.82 to 1006.46)	252.92 (-53.15 to 686.99)	622.05 (-114.23 to 1708.73)	52.77 (-8.84 to 143.88)	32.16 (-6.76 to 87.35)	41.86 (-7.69 to 114.99)
	Low temperature	2704.78 (2106.13 to 3321.58)	1905.45 (1458.94 to 2434.66)	4610.23 (3706.14 to 5639.76)	386.66 (301.08 to 474.84)	242.28 (185.51 to 309.57)	310.25 (249.41 to 379.53)
	Occupational risks	8661.24 (6875.24 to 10769.49)	3689.69 (2777.19 to 4784.16)	12350.94 (10465.18 to 14710.79)	1238.17 (982.85 to 1539.56)	469.15 (353.12 to 608.31)	831.16 (704.26 to 989.97)
	Occupational carcinogens	227.91 (194.16 to 262.47)	19.24 (12.29 to 28.47)	247.14 (212.49 to 285.04)	32.58 (27.76 to 37.52)	2.45 (1.56 to 3.62)	16.63 (14.3 to 19.18)
	Occupational asthmagens	442.56 (331.24 to 686.96)	208.24 (150.34 to 286.54)	650.8 (520.25 to 902.27)	63.27 (47.35 to 98.21)	26.48 (19.12 to 36.43)	43.8 (35.01 to 60.72)
	Occupational particulate matter, gases, and fumes	7990.78 (6233.81 to 10066.37)	3462.21 (2557.95 to 4500.02)	11452.99 (9523.46 to 13762.1)	1142.33 (891.16 to 1439.04)	440.22 (325.25 to 572.18)	770.73 (640.89 to 926.13)
	Behavioral risks	22495.11 (17336.38 to 27037.48)	7034.69 (4263.94 to 9858.57)	29529.8 (21926.3 to 36414.55)	3215.8 (2478.33 to 3865.16)	894.47 (542.16 to 1253.52)	1987.22 (1475.54 to 2450.53)
	Tobacco	22495.11 (17336.38 to 27037.48)	7034.69 (4263.94 to 9858.57)	29529.8 (21926.3 to 36414.55)	3215.8 (2478.33 to 3865.16)	894.47 (542.16 to 1253.52)	1987.22 (1475.54 to 2450.53)
	Smoking	21424.75 (16835.05 to 25631.7)	4823.51 (3395.91 to 6473.58)	26248.27 (20450.45 to 31516.61)	3062.79 (2406.66 to 3664.2)	613.31 (431.79 to 823.12)	1766.39 (1376.22 to 2120.92)
	Secondhand smoke	2401.81 (961.59 to 3893.73)	2561.98 (984.15 to 4121.81)	4963.79 (1978.97 to 7984.1)	343.35 (137.46 to 556.63)	325.76 (125.14 to 524.09)	334.04 (133.18 to 537.29)
	Metabolic risks	617.79 (271.88 to 979.13)	913.48 (394.32 to 1460.42)	1531.26 (664.36 to 2410.51)	88.32 (38.87 to 139.97)	116.15 (50.14 to 185.69)	103.05 (44.71 to 162.22)
	High body-mass index	617.79 (271.88 to 979.13)	913.48 (394.32 to 1460.42)	1531.26 (664.36 to 2410.51)	88.32 (38.87 to 139.97)	116.15 (50.14 to 185.69)	103.05 (44.71 to 162.22)
COPD							
	Environmental/occupational risks	23808.18 (20063.75 to 27945.03)	17365.77 (14523.75 to 20703.75)	41173.94 (35678.21 to 47728.44)	3403.51 (2868.23 to 3994.9)	2208.07 (1846.71 to 2632.5)	2770.82 (2400.98 to 3211.9)
	Air pollution	18483.7 (14604.67 to 22667.71)	14580.43 (11729.99 to 17875.5)	33064.13 (26736.14 to 39601.45)	2642.35 (2087.82 to 3240.48)	1853.91 (1491.48 to 2272.88)	2225.06 (1799.22 to 2664.99)
	Particulate matter pollution	16179.68 (12570.34 to 20605.3)	12961.1 (10017.35 to 16211.28)	29140.78 (23325.21 to 36295.63)	2312.98 (1797 to 2945.64)	1648.01 (1273.71 to 2061.28)	1961.04 (1569.68 to 2442.53)
	Ambient particulate matter pollution	9241.88 (6762.31 to 11452.99)	6461.18 (4475.46 to 8348.67)	15703.06 (11283.31 to 19331.56)	1321.18 (966.71 to 1637.27)	821.54 (569.06 to 1061.54)	1056.74 (759.31 to 1300.92)
	Household air pollution from solid fuels	6935.22 (4140.65 to 12170.46)	6498.23 (3879.86 to 10587.67)	13433.44 (8082.08 to 22299.04)	991.43 (591.93 to 1739.84)	826.25 (493.33 to 1346.23)	904.01 (543.89 to 1500.62)
	Ambient ozone pollution	4610.6 (928.7 to 7954.12)	3382.01 (764.71 to 5752.86)	7992.61 (1749.61 to 13654.4)	659.11 (132.76 to 1137.09)	430.02 (97.23 to 731.48)	537.87 (117.74 to 918.88)
	Non-optimal temperature	3049 (2102.34 to 4204.38)	2141.83 (1455.4 to 3049.21)	5190.83 (3632.34 to 7146.95)	435.87 (300.54 to 601.04)	272.34 (185.05 to 387.71)	349.32 (244.44 to 480.96)
	High temperature	369.13 (-61.82 to 1006.46)	252.92 (-53.15 to 686.99)	622.05 (-114.23 to 1708.73)	52.77 (-8.84 to 143.88)	32.16 (-6.76 to 87.35)	41.86 (-7.69 to 114.99)
	Low temperature	2704.78 (2106.13 to 3321.58)	1905.45 (1458.94 to 2434.66)	4610.23 (3706.14 to 5639.76)	386.66 (301.08 to 474.84)	242.28 (185.51 to 309.57)	310.25 (249.41 to 379.53)
	Occupational risks	7917.94 (6152.04 to 9992.17)	3448.44 (2544.47 to 4486.52)	11366.38 (9443.07 to 13676.91)	1131.91 (879.47 to 1428.44)	438.47 (323.53 to 570.46)	764.9 (635.47 to 920.39)
	Occupational particulate matter, gases, and fumes	7917.94 (6152.04 to 9992.17)	3448.44 (2544.47 to 4486.52)	11366.38 (9443.07 to 13676.91)	1131.91 (879.47 to 1428.44)	438.47 (323.53 to 570.46)	764.9 (635.47 to 920.39)

Behavioral risks	21846.53 (17180.91 to 26003.37)	6843.47 (4242.99 to 9416.8)	28690 (21736.45 to 34950.68)	3123.08 (2456.11 to 3717.33)	870.15 (539.5 to 1197.35)	1930.7 (1462.76 to 2352.02)
Tobacco	21846.53 (17180.91 to 26003.37)	6843.47 (4242.99 to 9416.8)	28690 (21736.45 to 34950.68)	3123.08 (2456.11 to 3717.33)	870.15 (539.5 to 1197.35)	1930.7 (1462.76 to 2352.02)
Smoking	20776.17 (16705.2 to 24572.94)	4632.29 (3370.97 to 6082.01)	25408.47 (20333.22 to 30084.81)	2970.07 (2388.1 to 3512.84)	589 (428.62 to 773.33)	1709.87 (1368.33 to 2024.57)
Secondhand smoke	2401.81 (961.59 to 3893.73)	2561.98 (984.15 to 4121.81)	4963.79 (1978.97 to 7984.1)	343.35 (137.46 to 556.63)	325.76 (125.14 to 524.09)	334.04 (133.18 to 537.29)
Asthma						
Environmental/occupational risks	442.56 (331.24 to 686.96)	208.24 (150.34 to 286.54)	650.8 (520.25 to 902.27)	63.27 (47.35 to 98.21)	26.48 (19.12 to 36.43)	43.8 (35.01 to 60.72)
Occupational risks	442.56 (331.24 to 686.96)	208.24 (150.34 to 286.54)	650.8 (520.25 to 902.27)	63.27 (47.35 to 98.21)	26.48 (19.12 to 36.43)	43.8 (35.01 to 60.72)
Occupational asthmagens	442.56 (331.24 to 686.96)	208.24 (150.34 to 286.54)	650.8 (520.25 to 902.27)	63.27 (47.35 to 98.21)	26.48 (19.12 to 36.43)	43.8 (35.01 to 60.72)
Behavioral risks	648.58 (74.88 to 1246.43)	191.22 (20.18 to 406.51)	839.8 (94.02 to 1635.94)	92.72 (10.7 to 178.18)	24.31 (2.57 to 51.69)	56.51 (6.33 to 110.09)
Tobacco	648.58 (74.88 to 1246.43)	191.22 (20.18 to 406.51)	839.8 (94.02 to 1635.94)	92.72 (10.7 to 178.18)	24.31 (2.57 to 51.69)	56.51 (6.33 to 110.09)
Smoking	648.58 (74.88 to 1246.43)	191.22 (20.18 to 406.51)	839.8 (94.02 to 1635.94)	92.72 (10.7 to 178.18)	24.31 (2.57 to 51.69)	56.51 (6.33 to 110.09)
Metabolic risks	617.79 (271.88 to 979.13)	913.48 (394.32 to 1460.42)	1531.26 (664.36 to 2410.51)	88.32 (38.87 to 139.97)	116.15 (50.14 to 185.69)	103.05 (44.71 to 162.22)
High body-mass index	617.79 (271.88 to 979.13)	913.48 (394.32 to 1460.42)	1531.26 (664.36 to 2410.51)	88.32 (38.87 to 139.97)	116.15 (50.14 to 185.69)	103.05 (44.71 to 162.22)

Table S23 Burden of Mortality Attributed to Risk Factors for CRDs, COPD, and Asthma

Cause	Risk factor	Deaths_NUM/1000 (95% UI)			Deaths_RATE/100,000 (95% UI)		
		Male	Female	Both	Male	Female	Both
CRDs	Environmental/occupational risks	1268.06 (1056.4 to 1492.11)	915.24 (746.74 to 1118.42)	2183.3 (1883.27 to 2539.78)	181.28 (151.02 to 213.31)	116.37 (94.95 to 142.21)	146.93 (126.74 to 170.92)
	Air pollution	957.12 (748.08 to 1175.22)	755.04 (597.91 to 946.83)	1712.16 (1367.28 to 2084.13)	136.83 (106.94 to 168)	96 (76.02 to 120.39)	115.22 (92.01 to 140.25)
	Particulate matter pollution	821.2 (628.52 to 1062.86)	652.49 (489.44 to 836.44)	1473.7 (1165.33 to 1840.92)	117.4 (89.85 to 151.94)	82.96 (62.23 to 106.35)	99.17 (78.42 to 123.89)
	Ambient particulate matter pollution	481.51 (351.51 to 598.65)	330.9 (226.1 to 430.45)	812.42 (590.85 to 1000.65)	68.84 (50.25 to 85.58)	42.07 (28.75 to 54.73)	54.67 (39.76 to 67.34)
	Household air pollution from solid fuels	339.56 (196.59 to 614.36)	321.5 (188.43 to 531.5)	661.06 (389.74 to 1128.19)	48.54 (28.1 to 87.83)	40.88 (23.96 to 67.58)	44.49 (26.23 to 75.92)
	Ambient ozone pollution	264.05 (53.77 to 454.49)	207.13 (46.31 to 354.15)	471.18 (103.42 to 806.06)	37.75 (7.69 to 64.97)	26.34 (5.89 to 45.03)	31.71 (6.96 to 54.24)
	Non-optimal temperature	185.04 (130.03 to 251.47)	140.36 (97.11 to 195.71)	325.39 (233.01 to 440.36)	26.45 (18.59 to 35.95)	17.85 (12.35 to 24.89)	21.9 (15.68 to 29.63)
	High temperature	20.48 (-4.03 to 56.47)	15.13 (-3.32 to 41.87)	35.62 (-7.37 to 98.34)	2.93 (-0.58 to 8.07)	1.92 (-0.42 to 5.32)	2.4 (-0.5 to 6.62)
	Low temperature	165.97 (131.31 to 202.1)	126.24 (97.1 to 159.37)	292.2 (235.56 to 354.97)	23.73 (18.77 to 28.89)	16.05 (12.35 to 20.26)	19.66 (15.85 to 23.89)
	Occupational risks	425.63 (325.95 to 541.94)	175.79 (120.54 to 246.31)	601.43 (491.07 to 734.25)	60.85 (46.6 to 77.47)	22.35 (15.33 to 31.32)	40.47 (33.05 to 49.41)
	Occupational carcinogens	11.04 (9.44 to 12.67)	0.87 (0.49 to 1.36)	11.9 (10.22 to 13.67)	1.58 (1.35 to 1.81)	0.11 (0.06 to 0.17)	0.8 (0.69 to 0.92)
	Occupational asthmagens	13.45 (10.04 to 22.9)	5.56 (3.87 to 8.14)	19.01 (14.8 to 28.94)	1.92 (1.43 to 3.27)	0.71 (0.49 to 1.04)	1.28 (1 to 1.95)
	Occupational particulate matter, gases, and fumes	401.15 (305.28 to 515.56)	169.36 (114.71 to 239.2)	570.51 (457.13 to 697.61)	57.35 (43.64 to 73.7)	21.53 (14.59 to 30.41)	38.39 (30.76 to 46.95)
	Behavioral risks	1146.47 (884.96 to 1395.86)	345.51 (205.39 to 495.6)	1491.98 (1108.28 to 1873.48)	163.89 (126.51 to 199.55)	43.93 (26.12 to 63.02)	100.4 (74.58 to 126.08)
	Tobacco	1146.47 (884.96 to 1395.86)	345.51 (205.39 to 495.6)	1491.98 (1108.28 to 1873.48)	163.89 (126.51 to 199.55)	43.93 (26.12 to 63.02)	100.4 (74.58 to 126.08)
	Smoking	1090.6 (852.81 to 1320.42)	233.16 (160.67 to 319.39)	1323.76 (1022.69 to 1608.52)	155.91 (121.91 to 188.76)	29.65 (20.43 to 40.61)	89.08 (68.82 to 108.25)
	Secondhand smoke	126.11 (49.54 to 209.74)	130.1 (49.96 to 213.75)	256.2 (102.13 to 419.02)	18.03 (7.08 to 29.98)	16.54 (6.35 to 27.18)	17.24 (6.87 to 28.2)
	Metabolic risks	20.05 (8.58 to 33.03)	29.19 (12.35 to 48.08)	49.24 (20.24 to 79.77)	2.87 (1.23 to 4.72)	3.71 (1.57 to 6.11)	3.31 (1.36 to 5.37)
	High body-mass index	20.05 (8.58 to 33.03)	29.19 (12.35 to 48.08)	49.24 (20.24 to 79.77)	2.87 (1.23 to 4.72)	3.71 (1.57 to 6.11)	3.31 (1.36 to 5.37)
COPD							
	Environmental/occupational risks	1240.01 (1023.85 to 1464.13)	908.17 (739.41 to 1110.52)	2148.18 (1846 to 2506.14)	177.27 (146.36 to 209.31)	115.47 (94.02 to 141.2)	144.56 (124.23 to 168.65)
	Air pollution	957.12 (748.08 to 1175.22)	755.04 (597.91 to 946.83)	1712.16 (1367.28 to 2084.13)	136.83 (106.94 to 168)	96 (76.02 to 120.39)	115.22 (92.01 to 140.25)
	Particulate matter pollution	821.2 (628.52 to 1062.86)	652.49 (489.44 to 836.44)	1473.7 (1165.33 to 1840.92)	117.4 (89.85 to 151.94)	82.96 (62.23 to 106.35)	99.17 (78.42 to 123.89)
	Ambient particulate matter pollution	481.51 (351.51 to 598.65)	330.9 (226.1 to 430.45)	812.42 (590.85 to 1000.65)	68.84 (50.25 to 85.58)	42.07 (28.75 to 54.73)	54.67 (39.76 to 67.34)
	Household air pollution from solid fuels	339.56 (196.59 to 614.36)	321.5 (188.43 to 531.5)	661.06 (389.74 to 1128.19)	48.54 (28.1 to 87.83)	40.88 (23.96 to 67.58)	44.49 (26.23 to 75.92)
	Ambient ozone pollution	264.05 (53.77 to 454.49)	207.13 (46.31 to 354.15)	471.18 (103.42 to 806.06)	37.75 (7.69 to 64.97)	26.34 (5.89 to 45.03)	31.71 (6.96 to 54.24)
	Non-optimal temperature	185.04 (130.03 to 251.47)	140.36 (97.11 to 195.71)	325.39 (233.01 to 440.36)	26.45 (18.59 to 35.95)	17.85 (12.35 to 24.89)	21.9 (15.68 to 29.63)
	High temperature	20.48 (-4.03 to 56.47)	15.13 (-3.32 to 41.87)	35.62 (-7.37 to 98.34)	2.93 (-0.58 to 8.07)	1.92 (-0.42 to 5.32)	2.4 (-0.5 to 6.62)
	Low temperature	165.97 (131.31 to 202.1)	126.24 (97.1 to 159.37)	292.2 (235.56 to 354.97)	23.73 (18.77 to 28.89)	16.05 (12.35 to 20.26)	19.66 (15.85 to 23.89)
	Occupational risks	397.58 (301.98 to 511.93)	168.72 (113.98 to 238.65)	566.31 (453.26 to 693.37)	56.84 (43.17 to 73.18)	21.45 (14.49 to 30.35)	38.11 (30.5 to 46.66)
	Occupational particulate matter, gases, and fumes	397.58 (301.98 to 511.93)	168.72 (113.98 to 238.65)	566.31 (453.26 to 693.37)	56.84 (43.17 to 73.18)	21.45 (14.49 to 30.35)	38.11 (30.5 to 46.66)

Behavioral risks	1122.89 (872.34 to 1359.5)	339.95 (205.68 to 484.53)	1462.84 (1103.69 to 1814.25)	160.52 (124.71 to 194.35)	43.22 (26.15 to 61.61)	98.44 (74.27 to 122.09)
Tobacco	1122.89 (872.34 to 1359.5)	339.95 (205.68 to 484.53)	1462.84 (1103.69 to 1814.25)	160.52 (124.71 to 194.35)	43.22 (26.15 to 61.61)	98.44 (74.27 to 122.09)
Smoking	1067.02 (840.34 to 1281.58)	227.6 (160.3 to 310.32)	1294.62 (1020.48 to 1548.38)	152.54 (120.13 to 183.21)	28.94 (20.38 to 39.46)	87.12 (68.67 to 104.2)
Secondhand smoke	126.11 (49.54 to 209.74)	130.1 (49.96 to 213.75)	256.2 (102.13 to 419.02)	18.03 (7.08 to 29.98)	16.54 (6.35 to 27.18)	17.24 (6.87 to 28.2)
Asthma						
Environmental/occupational risks	13.45 (10.04 to 22.9)	5.56 (3.87 to 8.14)	19.01 (14.8 to 28.94)	1.92 (1.43 to 3.27)	0.71 (0.49 to 1.04)	1.28 (1 to 1.95)
Occupational risks	13.45 (10.04 to 22.9)	5.56 (3.87 to 8.14)	19.01 (14.8 to 28.94)	1.92 (1.43 to 3.27)	0.71 (0.49 to 1.04)	1.28 (1 to 1.95)
Occupational asthmagens	13.45 (10.04 to 22.9)	5.56 (3.87 to 8.14)	19.01 (14.8 to 28.94)	1.92 (1.43 to 3.27)	0.71 (0.49 to 1.04)	1.28 (1 to 1.95)
Behavioral risks	23.58 (2.75 to 47.28)	5.56 (0.55 to 12.33)	29.14 (3.31 to 59.82)	3.37 (0.39 to 6.76)	0.71 (0.07 to 1.57)	1.96 (0.22 to 4.03)
Tobacco	23.58 (2.75 to 47.28)	5.56 (0.55 to 12.33)	29.14 (3.31 to 59.82)	3.37 (0.39 to 6.76)	0.71 (0.07 to 1.57)	1.96 (0.22 to 4.03)
Smoking	23.58 (2.75 to 47.28)	5.56 (0.55 to 12.33)	29.14 (3.31 to 59.82)	3.37 (0.39 to 6.76)	0.71 (0.07 to 1.57)	1.96 (0.22 to 4.03)
Metabolic risks	20.05 (8.58 to 33.03)	29.19 (12.35 to 48.08)	49.24 (20.24 to 79.77)	2.87 (1.23 to 4.72)	3.71 (1.57 to 6.11)	3.31 (1.36 to 5.37)
High body-mass index	20.05 (8.58 to 33.03)	29.19 (12.35 to 48.08)	49.24 (20.24 to 79.77)	2.87 (1.23 to 4.72)	3.71 (1.57 to 6.11)	3.31 (1.36 to 5.37)

Table S24 Decomposition Analysis of CRDs-Related Burden Globally and by SDI Region, 1990–2021

Prevalence

**location**	**cause**	**overall difference**	**Aging**	**Population**	**Epidemiological change**	**Percent change of aging**	**Percent change of population**	**Percent change of epidemiological change**
Global	CRDs	87035454.64	58769212.16	168108217.44	-139841974.96	67.52	193.15	-160.67
High SDI	CRDs	11272078.33	18330963.27	25508198.42	-32567083.36	162.62	226.30	-288.92
High-middle SDI	CRDs	7236245.86	17887073.70	15707564.87	-26358392.71	247.19	217.07	-364.26
Middle SDI	CRDs	27824117.47	19602409.75	38925246.50	-30703538.78	70.45	139.90	-110.35
Low-middle SDI	CRDs	19495548.21	6617312.19	39171893.94	-26293657.92	33.94	200.93	-134.87
Low SDI	CRDs	21199249.59	-1171981.35	36059244.17	-13688013.24	-5.53	170.10	-64.57

Incidence

**location**	**cause**	**overall difference**	**Aging**	**Population**	**Epidemiological change**	**Percent change of aging**	**Percent change of population**	**Percent change of epidemiological change**
Global	CRDs	5402632.01	-601011.55	20827689.95	-14824046.38	-11.12	385.51	-274.39
High SDI	CRDs	1135772.23	240355.03	2261713.36	-1366296.16	21.16	199.13	-120.30
High-middle SDI	CRDs	-237734.96	505042.69	1671391.32	-2414168.97	-212.44	-703.05	1015.49
Middle SDI	CRDs	465899.87	-271708.28	5422821.50	-4685213.35	-58.32	1163.95	-1005.63
Low-middle SDI	CRDs	756029.66	-755253.70	5806371.87	-4295088.50	-99.90	768.01	-568.11
Low SDI	CRDs	3282632.83	-680217.01	6103182.93	-2140333.09	-20.72	185.92	-65.20

DALYs (Disability-Adjusted Life Years)

**location**	**cause**	**overall difference**	**Aging**	**Population**	**Epidemiological change**	**Percent change of aging**	**Percent change of population**	**Percent change of epidemiological change**
Global	CRDs	23611960.40	33599219.40	38887239.48	-48874498.47	142.30	164.69	-206.99
High SDI	CRDs	3441902.49	4417807.96	2949561.59	-3925467.05	128.35	85.70	-114.05
High-middle SDI	CRDs	-1760261.33	8834058.72	3735103.96	-14329424.00	-501.86	-212.19	814.05
Middle SDI	CRDs	5015474.45	19147343.37	11835326.20	-25967195.12	381.77	235.98	-517.74
Low-middle SDI	CRDs	12597446.08	7787450.76	12619929.84	-7809934.52	61.82	100.18	-62.00
Low SDI	CRDs	4311267.89	20749.08	7846016.17	-3555497.37	0.48	181.99	-82.47

Deaths

location	cause	overall difference	Aging	Population	Epidemiological change	Percent change of aging	Percent change of population	Percent change of epidemiological change
Global	CRDs	1423137.11	1806346.14	1481311.02	-1864520.04	126.93	104.09	-131.01
High SDI	CRDs	218304.28	241267.22	102440.31	-125403.25	110.52	46.93	-57.44
High-middle SDI	CRDs	11157.98	515407.26	164345.79	-668595.07	4619.18	1472.90	-5992.08
Middle SDI	CRDs	383802.98	1050592.24	487992.17	-1154781.43	273.73	127.15	-300.88
Low-middle SDI	CRDs	648095.88	383940.35	454632.98	-190477.45	59.24	70.15	-29.39
Low SDI	CRDs	161311.36	11980.09	226013.53	-76682.27	7.43	140.11	-47.54

Table S25 Decomposition Analysis of COPD-Related Burden Globally and by SDI Region, 1990–2021

Prevalence

location	cause	overall difference	Aging	Population	Epidemiological change	Percent change of aging	Percent change of population	Percent change of epidemiological change
Global	COPD	112842590.75	57478951.97	58679010.29	-3315371.51	50.94	52.00	-2.94
High SDI	COPD	23761789.86	15955525.29	8595291.52	-789026.95	67.15	36.17	-3.32
High-middle SDI	COPD	21083124.61	16260440.43	7009880.03	-2187195.85	77.13	33.25	-10.37
Middle SDI	COPD	38058542.18	24183609.55	14903428.11	-1028495.48	63.54	39.16	-2.70
Low-middle SDI	COPD	22449905.10	9057506.24	12931741.68	460657.18	40.35	57.60	2.05
Low SDI	COPD	7414386.44	390284.45	6718584.76	305517.23	5.26	90.62	4.12

Incidence

location	cause	overall difference	Aging	Population	Epidemiological change	Percent change of aging	Percent change of population	Percent change of epidemiological change
Global	COPD	8840580.23	4557662.15	4672092.48	-389174.40	51.55	52.85	-4.40
High SDI	COPD	1732313.90	1039124.30	608894.89	84294.71	59.98	35.15	4.87
High-middle SDI	COPD	1589757.58	1302444.64	556917.68	-269604.75	81.93	35.03	-16.96
Middle SDI	COPD	3096999.65	2131129.10	1267667.18	-301796.63	68.81	40.93	-9.74
Low-middle SDI	COPD	1838923.97	784554.86	1098587.58	-44218.48	42.66	59.74	-2.40
Low SDI	COPD	577091.82	30453.66	538535.22	8102.94	5.28	93.32	1.40

DALYs (Disability-Adjusted Life Years)

location	cause	overall difference	Aging	Population	Epidemiological change	Percent change of aging	Percent change of population	Percent change of epidemiological change
Global	COPD	22922404.63	29657918.83	27412467.61	-34147981.81	129.38	119.59	-148.97
High SDI	COPD	3579671.20	3662130.62	1833038.88	-1915498.29	102.30	51.21	-53.51
High-middle SDI	COPD	-692878.30	8224529.23	3042281.43	-11959688.96	-1187.01	-439.08	1726.09
Middle SDI	COPD	5242177.17	17788324.85	9254578.59	-21800726.27	339.33	176.54	-415.87
Low-middle SDI	COPD	11600066.56	6425146.87	8208434.10	-3033514.42	55.39	70.76	-26.15
Low SDI	COPD	3182723.28	157131.14	4002094.07	-976501.93	4.94	125.74	-30.68

Deaths

location	cause	overall difference	Aging	Population	Epidemiological change	Percent change of aging	Percent change of population	Percent change of epidemiological change
Global	COPD	4248608.18	4005936.58	4022161.22	-3779489.62	94.29	94.67	-88.96
High SDI	COPD	463061.53	393205.30	232721.19	-162864.96	84.91	50.26	-35.17
High-middle SDI	COPD	183582.91	1322391.50	546961.56	-1685770.16	720.32	297.94	-918.26
Middle SDI	COPD	1502634.07	3042839.57	1676874.79	-3217080.28	202.50	111.60	-214.10
Low-middle SDI	COPD	2072746.95	917231.55	1225914.85	-70399.45	44.25	59.14	-3.40
Low SDI	COPD	553018.37	11889.44	588351.90	-47222.97	2.15	106.39	-8.54

Table S26 Decomposition Analysis of Asthma-Related Burden Globally and by SDI Region, 1990–2021

Prevalence

location	cause	overall difference	Aging	Population	Epidemiological change	Percent change of aging	Percent change of population	Percent change of epidemiological change
Global	Asthma	-26821701.17	4480506.04	112106340.76	-143408547.97	-16.70	-417.97	534.67
High SDI	Asthma	-13421608.07	3874894.97	17618840.67	-34915343.71	-28.87	-131.27	260.14
High-middle SDI	Asthma	-14550526.64	2368625.07	8957328.33	-25876480.04	-16.28	-61.56	177.84
Middle SDI	Asthma	-10172749.66	-3854932.48	24368479.96	-30686297.14	37.89	-239.55	301.65
Low-middle SDI	Asthma	-2531726.20	-2063068.61	26689420.43	-27158078.03	81.49	-1054.20	1072.71
Low SDI	Asthma	13925635.16	-1550878.37	29590252.81	-14113739.28	-11.14	212.49	-101.35

Incidence

location	cause	overall difference	Aging	Population	Epidemiological change	Percent change of aging	Percent change of population	Percent change of epidemiological change
Global	Asthma	-3691452.61	-5265749.86	16035381.08	-14461083.84	142.65	-434.39	391.75
High SDI	Asthma	-690513.33	-836463.64	1627522.37	-1481572.05	121.14	-235.70	214.56
High-middle SDI	Asthma	-1861766.62	-818950.16	1103633.59	-2146450.05	43.99	-59.28	115.29
Middle SDI	Asthma	-2698477.35	-2441701.14	4129191.61	-4385967.83	90.48	-153.02	162.53
Low-middle SDI	Asthma	-1128995.69	-1557365.20	4681880.19	-4253510.68	137.94	-414.69	376.75
Low SDI	Asthma	2693835.09	-711305.75	5553500.15	-2148359.32	-26.40	206.16	-79.75

DALYs (Disability-Adjusted Life Years)

location	cause	overall difference	Aging	Population	Epidemiological change	Percent change of aging	Percent change of population	Percent change of epidemiological change
Global	Asthma	-1439189.80	2966644.95	9091014.68	-13496849.43	-206.13	-631.68	937.81
High SDI	Asthma	-1132131.01	325299.67	849736.62	-2307167.29	-28.73	-75.06	203.79
High-middle SDI	Asthma	-1120385.72	378657.25	526329.13	-2025372.10	-33.80	-46.98	180.77
Middle SDI	Asthma	-570746.35	1061992.79	2055151.05	-3687890.19	-186.07	-360.08	646.15
Low-middle SDI	Asthma	553023.24	1217697.70	3562505.39	-4227179.85	220.19	644.19	-764.38
Low SDI	Asthma	835802.68	-68920.82	3127256.43	-2222532.94	-8.25	374.16	-265.92

Deaths

location	cause	overall difference	Aging	Population	Epidemiological change	Percent change of aging	Percent change of population	Percent change of epidemiological change
Global	Asthma	61815.70	163058.93	166583.03	-267826.26	263.78	269.48	-433.27
High SDI	Asthma	-23196.85	15536.09	7493.40	-46226.34	-66.98	-32.30	199.28
High-middle SDI	Asthma	-14647.64	18988.30	7240.42	-40876.36	-129.63	-49.43	279.06
Middle SDI	Asthma	18217.66	72125.70	40117.69	-94025.74	395.91	220.21	-516.12
Low-middle SDI	Asthma	62508.93	65018.73	88512.12	-91021.91	104.02	141.60	-145.61
Low SDI	Asthma	18970.43	2052.33	56945.88	-40027.77	10.82	300.18	-211.00

Table S27 Decomposition Analysis of ILD & PS-Related Burden Globally and by SDI Region, 1990–2021

Prevalence

location	cause	overall difference	Aging	Population	Epidemiological change	Percent change of aging	Percent change of population	Percent change of epidemiological change
Global	ILD&PS	2419182.46	1070368.99	1141365.76	207447.70	44.25	47.18	8.58
High SDI	ILD&PS	1036401.82	471421.34	296313.75	268666.74	45.49	28.59	25.92
High-middle SDI	ILD&PS	352889.70	215387.95	103651.73	33850.03	61.04	29.37	9.59
Middle SDI	ILD&PS	577313.20	307521.13	199058.71	70733.36	53.27	34.48	12.25
Low-middle SDI	ILD&PS	363587.49	132544.49	194947.05	36095.95	36.45	53.62	9.93
Low SDI	ILD&PS	88005.52	3550.19	81925.80	2529.53	4.03	93.09	2.87

Incidence

location	cause	overall difference	Aging	Population	Epidemiological change	Percent change of aging	Percent change of population	Percent change of epidemiological change
Global	ILD&PS	232825.93	88238.93	99517.18	45069.83	37.90	42.74	19.36
High SDI	ILD&PS	90008.88	33016.73	22601.58	34390.57	36.68	25.11	38.21
High-middle SDI	ILD&PS	30385.73	15128.94	7900.45	7356.35	49.79	26.00	24.21
Middle SDI	ILD&PS	58946.73	28661.94	19285.09	10999.70	48.62	32.72	18.66
Low-middle SDI	ILD&PS	42811.35	15818.12	23123.28	3869.94	36.95	54.01	9.04
Low SDI	ILD&PS	10596.07	551.92	9771.71	272.45	5.21	92.22	2.57

DALYs (Disability-Adjusted Life Years)

location	cause	overall difference	Aging	Population	Epidemiological change	Percent change of aging	Percent change of population	Percent change of epidemiological change
Global	ILD&PS	2541122.06	969053.65	995347.02	576721.39	38.13	39.17	22.70
High SDI	ILD&PS	994724.96	360724.73	201055.16	432945.06	36.26	20.21	43.52
High-middle SDI	ILD&PS	247997.34	156539.28	70599.81	20858.26	63.12	28.47	8.41
Middle SDI	ILD&PS	542200.36	269476.74	173198.81	99524.80	49.70	31.94	18.36
Low-middle SDI	ILD&PS	589948.82	214771.44	302503.46	72673.93	36.41	51.28	12.32
Low SDI	ILD&PS	165183.66	5053.37	158178.16	1952.14	3.06	95.76	1.18

Deaths

location	cause	overall difference	Aging	Population	Epidemiological change	Percent change of aging	Percent change of population	Percent change of epidemiological change
Global	ILD&PS	133255.14	50880.72	42209.76	40164.65	38.18	31.68	30.14
High SDI	ILD&PS	61668.33	22114.56	9848.62	29705.15	35.86	15.97	48.17
High-middle SDI	ILD&PS	14613.15	8277.59	2940.49	3395.07	56.64	20.12	23.23
Middle SDI	ILD&PS	24349.14	12916.40	6634.96	4797.78	53.05	27.25	19.70
Low-middle SDI	ILD&PS	25964.94	9806.30	11864.72	4293.92	37.77	45.70	16.54
Low SDI	ILD&PS	6605.89	306.00	5799.00	500.89	4.63	87.79	7.58

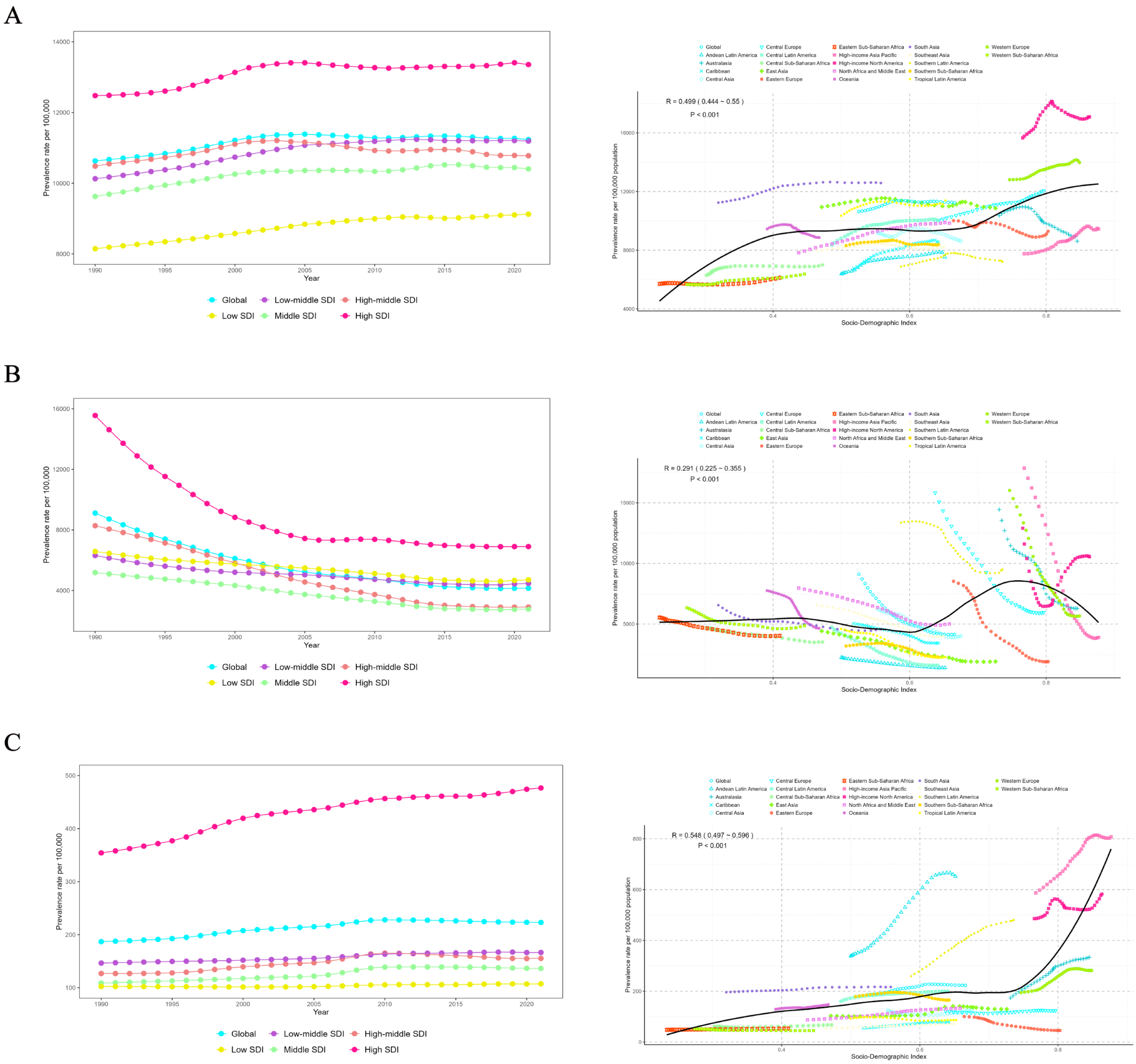

Fig.S1 Trends in Disease Burden with Changes in SDI at the Global Level, 5 SDI Regions, and 21 GBD Regions

A: Trends in Chronic Obstructive Pulmonary Disease Prevalence and SDI Across Global Regions

B: Trends in Asthma Prevalence and SDI Across Global Regions

C: Trends in Interstitial Lung Disease and Pulmonary Sarcoidosis Prevalence and SDI Across Global Regions

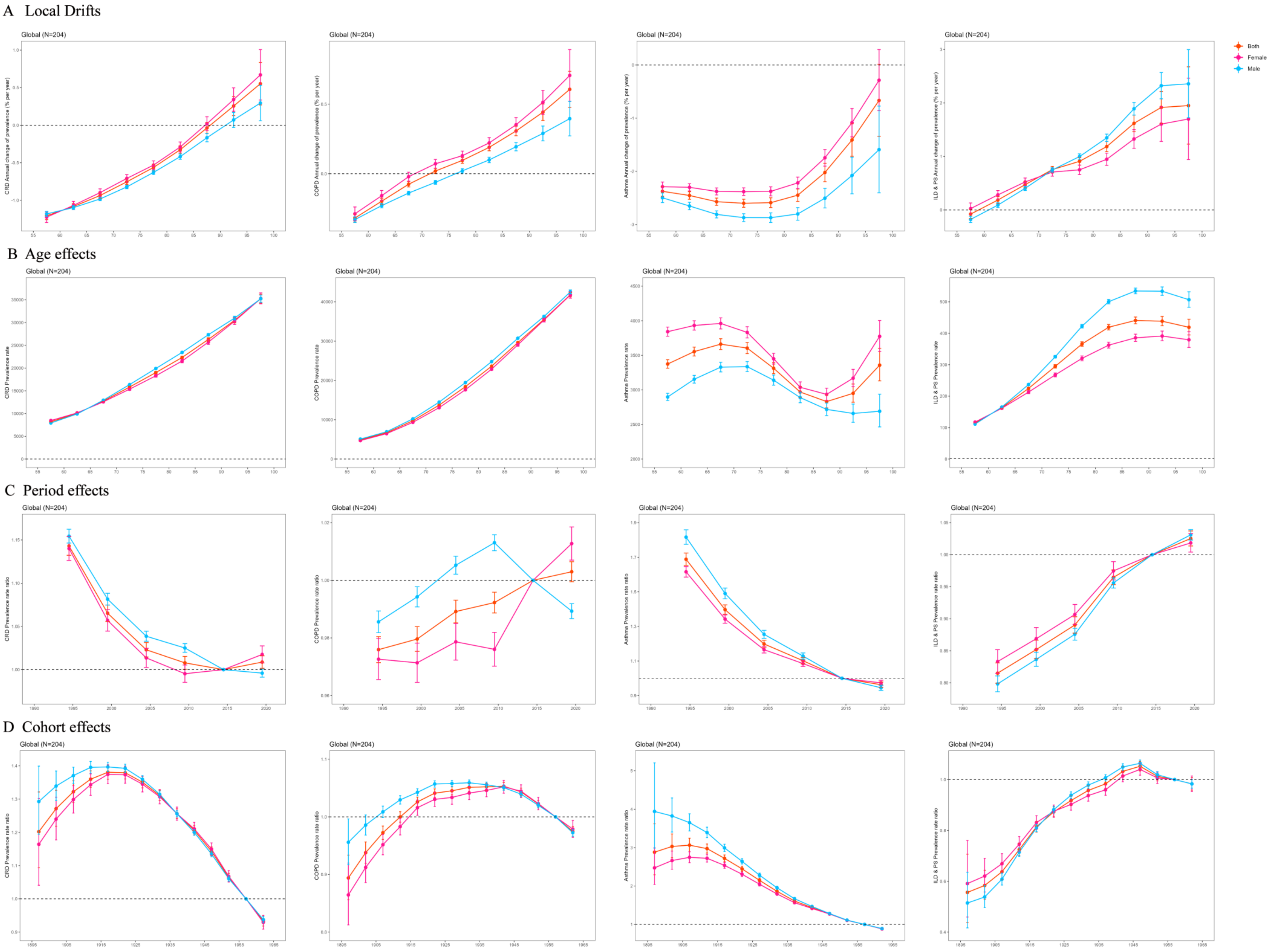

Fig.S2 APC Model Analysis Figures for the Prevalence of CRDs, COPD, Asthma, and ILD & PS at the Global Level

A: Local Drift Figures for the Prevalence of CRDs, COPD, Asthma, and ILD & PS at the Global Level

B: Age Effect Figures for the Prevalence of CRDs, COPD, Asthma, and ILD & PS at the Global Level

C: Period Effect Figures for the Prevalence of CRDs, COPD, Asthma, and ILD & PS at the Global Level

D: Cohort Effect Figures for the Prevalence of CRDs, COPD, Asthma, and ILD & PS at the Global Level

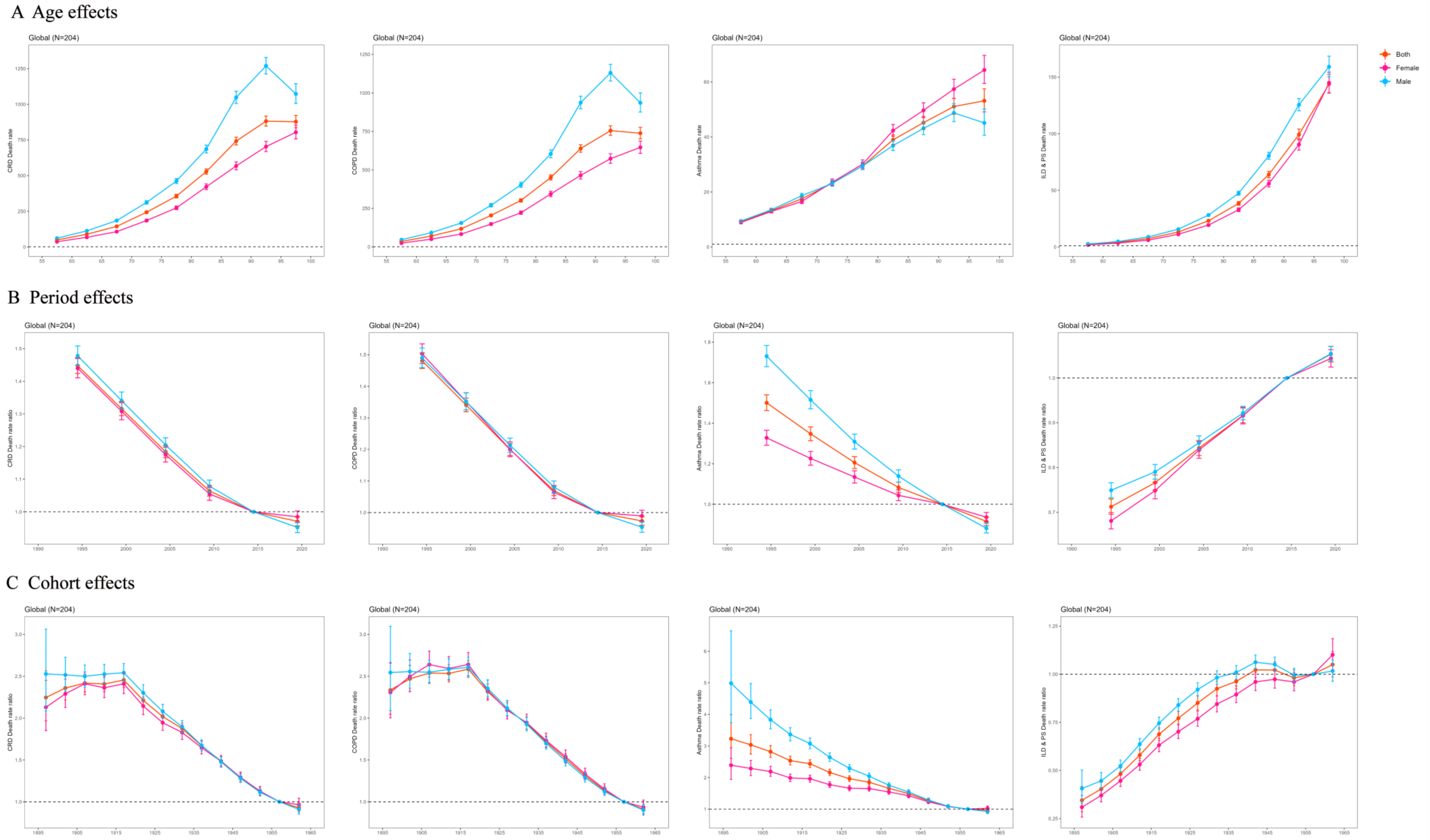

Fig.S3 APC Model Analysis Figures for the Mortality of CRDs, COPD, Asthma, and ILD & PS at the Global Level

A: Age Effect Figures for the Mortality of CRDs, COPD, Asthma, and ILD & PS at the Global Level

B: Period Effect Figures for the Mortality of CRDs, COPD, Asthma, and ILD & PS at the Global Level

C: Cohort Effect Figures for the Mortality of CRDs, COPD, Asthma, and ILD & PS at the Global Level

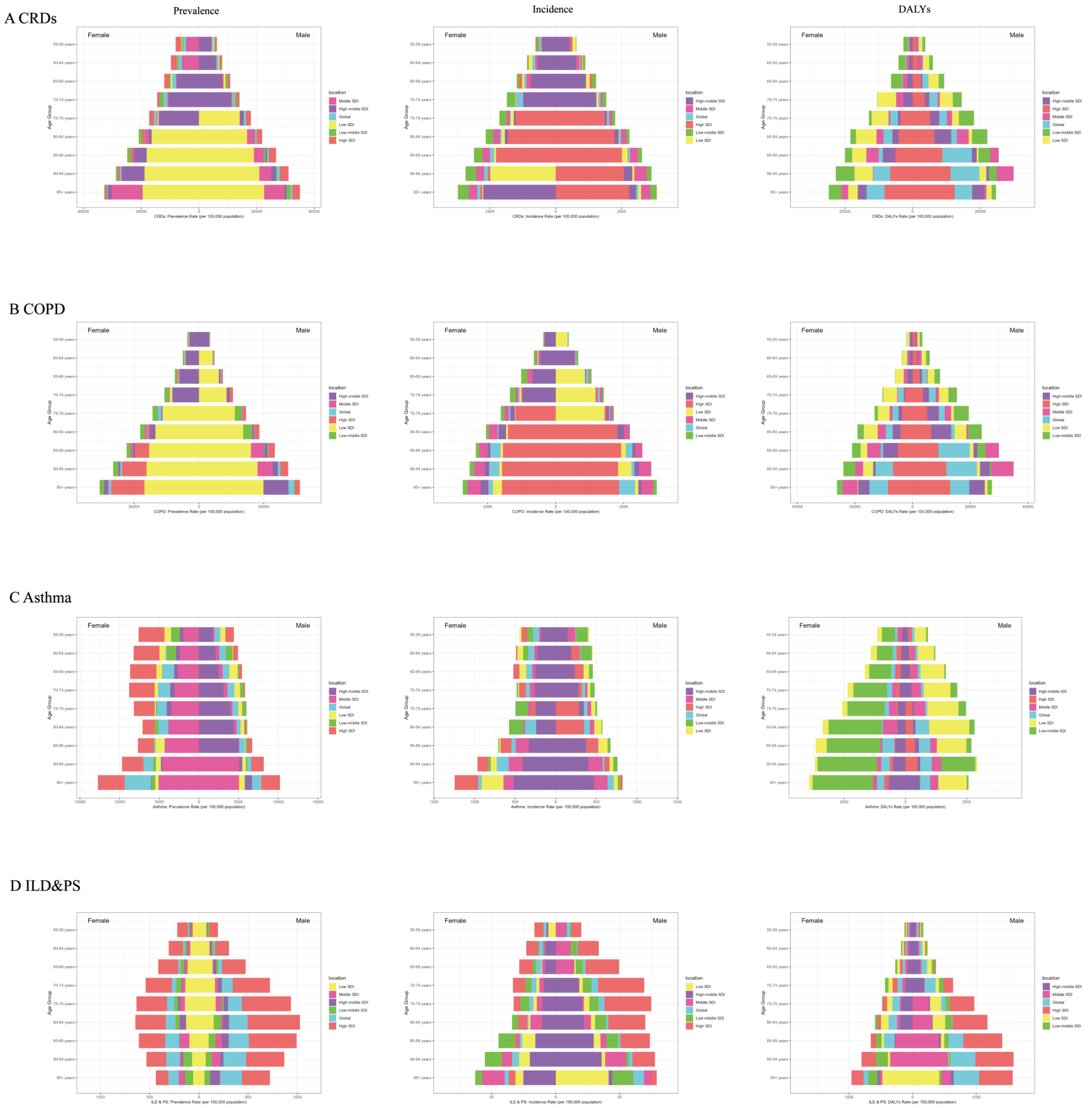

Fig.S4 Age and Sex Structure Figures for the Prevalence, Incidence, and DALYs of CRDs, COPD, Asthma, and ILD & PS at the Global Level

A: Age and Sex Structure Figures for CRDs

B: Age and Sex Structure Figures for COPD

C: Age and Sex Structure Figures for Asthma

D: Age and Sex Structure Figures for ILD & PS

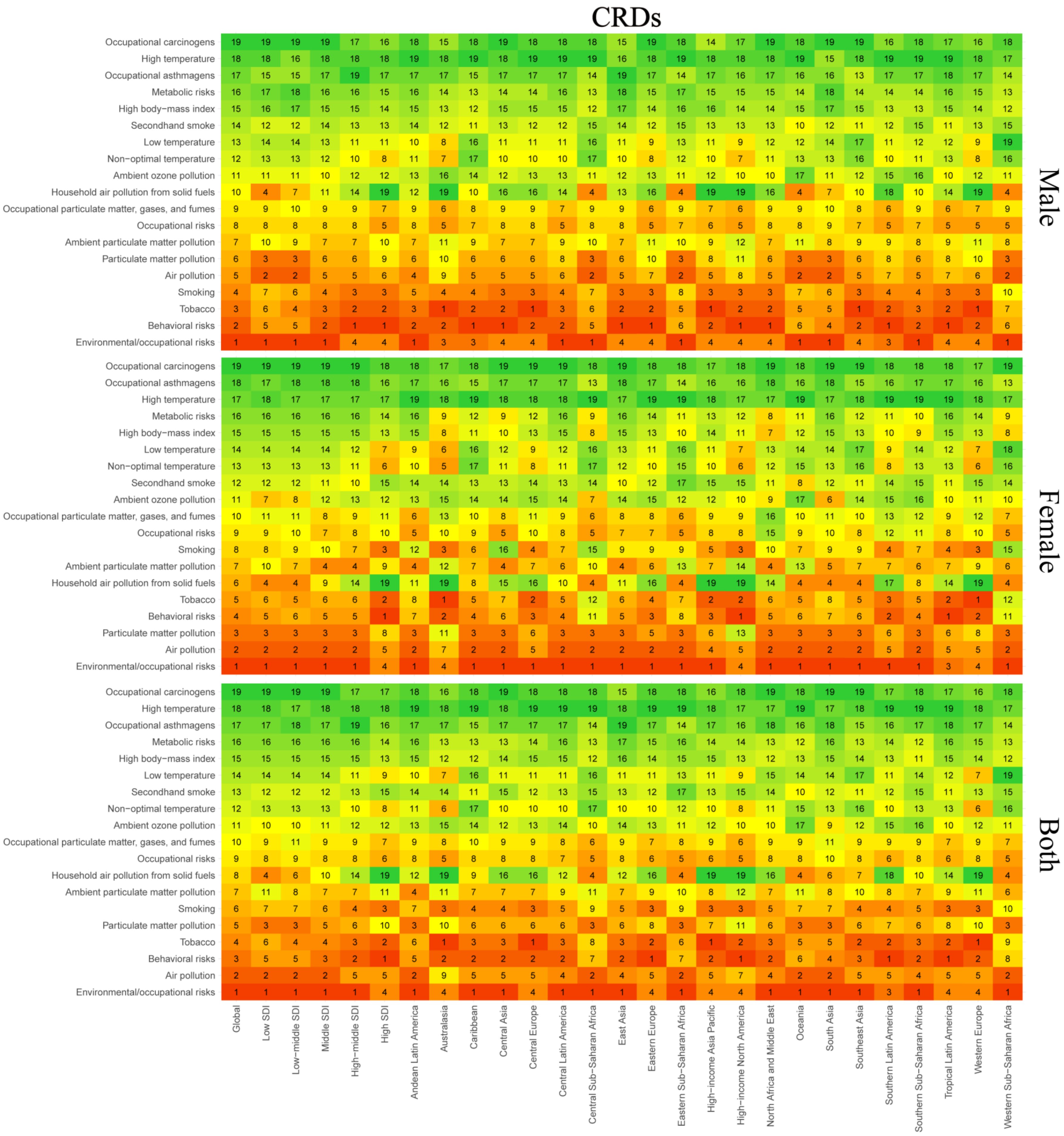

Fig.S5 Risk Factor Figures for the DALYs Rate of CRDs at the Global Level, 5 SDI Regions, and 21 GBD Regions

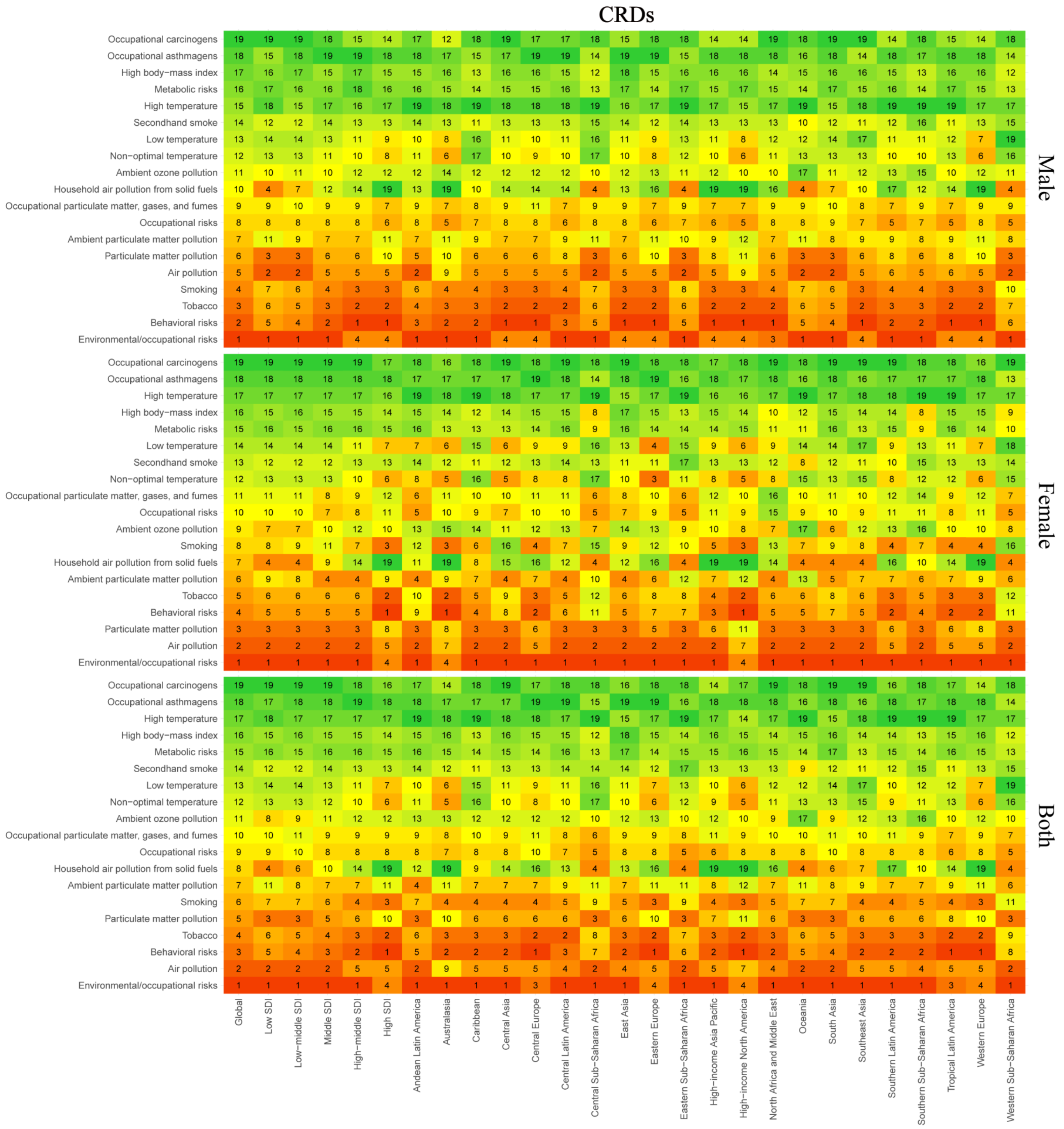

Fig.S6 Risk Factor Figures for the Mortality Rate of CRDs at the Global Level, 5 SDI Regions, and 21 GBD Regions

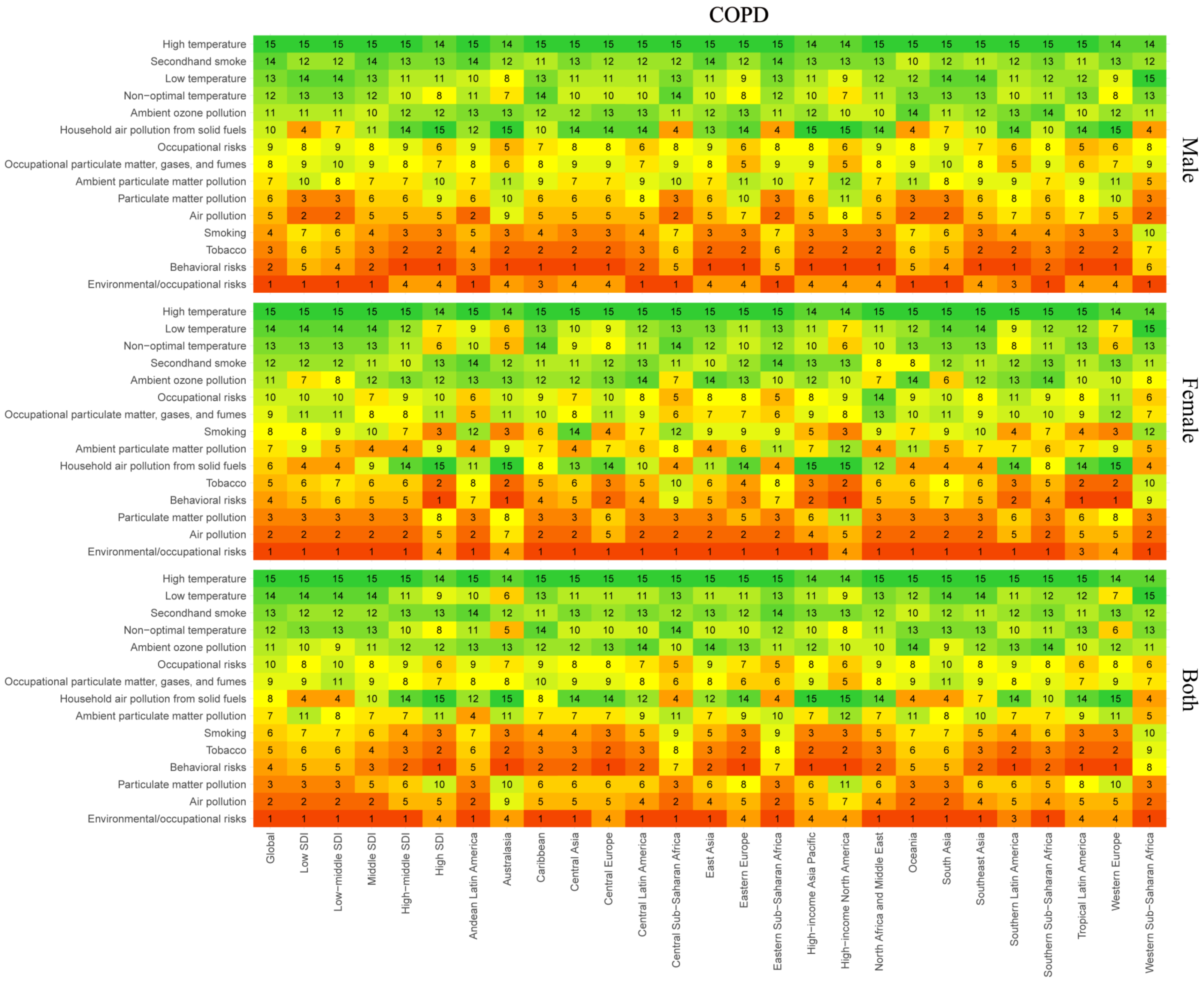

Fig.S7 Risk Factor Figures for the DALYs Rate of COPD at the Global Level, 5 SDI Regions, and 21 GBD Regions

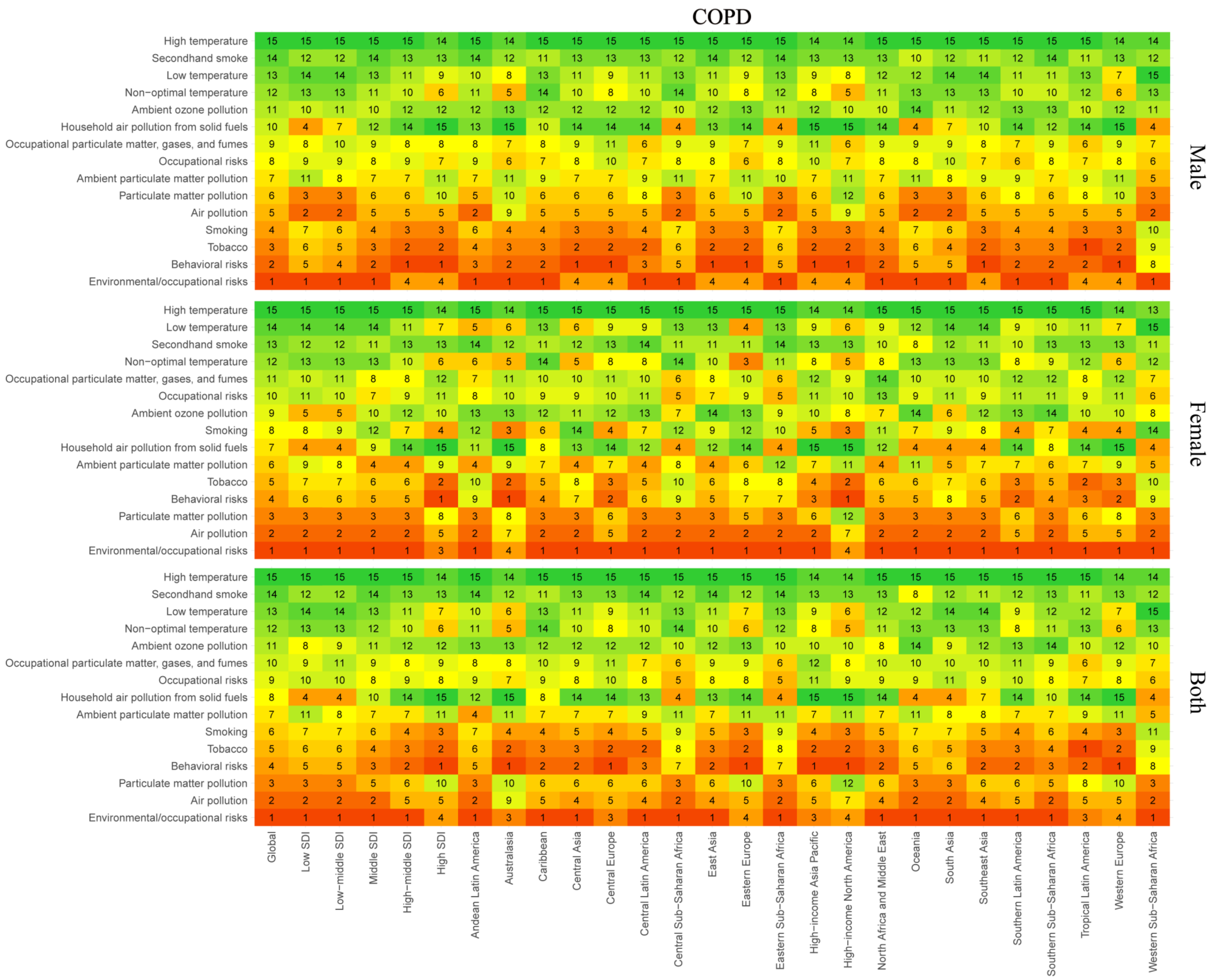

Fig.S8 Risk Factor Figures for the Mortality Rate of COPD at the Global Level, 5 SDI Regions, and 21 GBD Regions

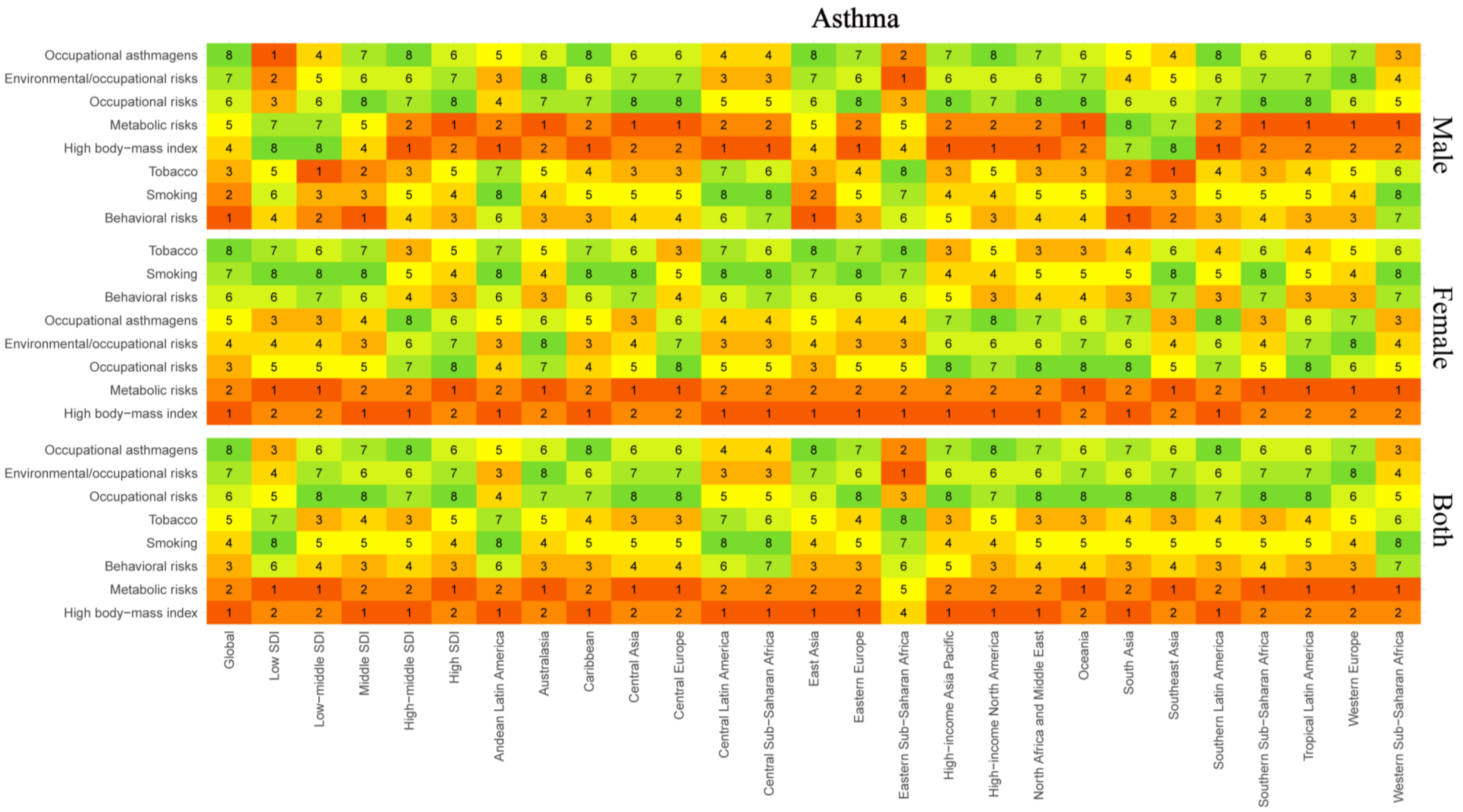

Fig.S9 Risk Factor Figures for the DALYs Rate of Asthma at the Global Level, 5 SDI Regions, and 21 GBD Regions

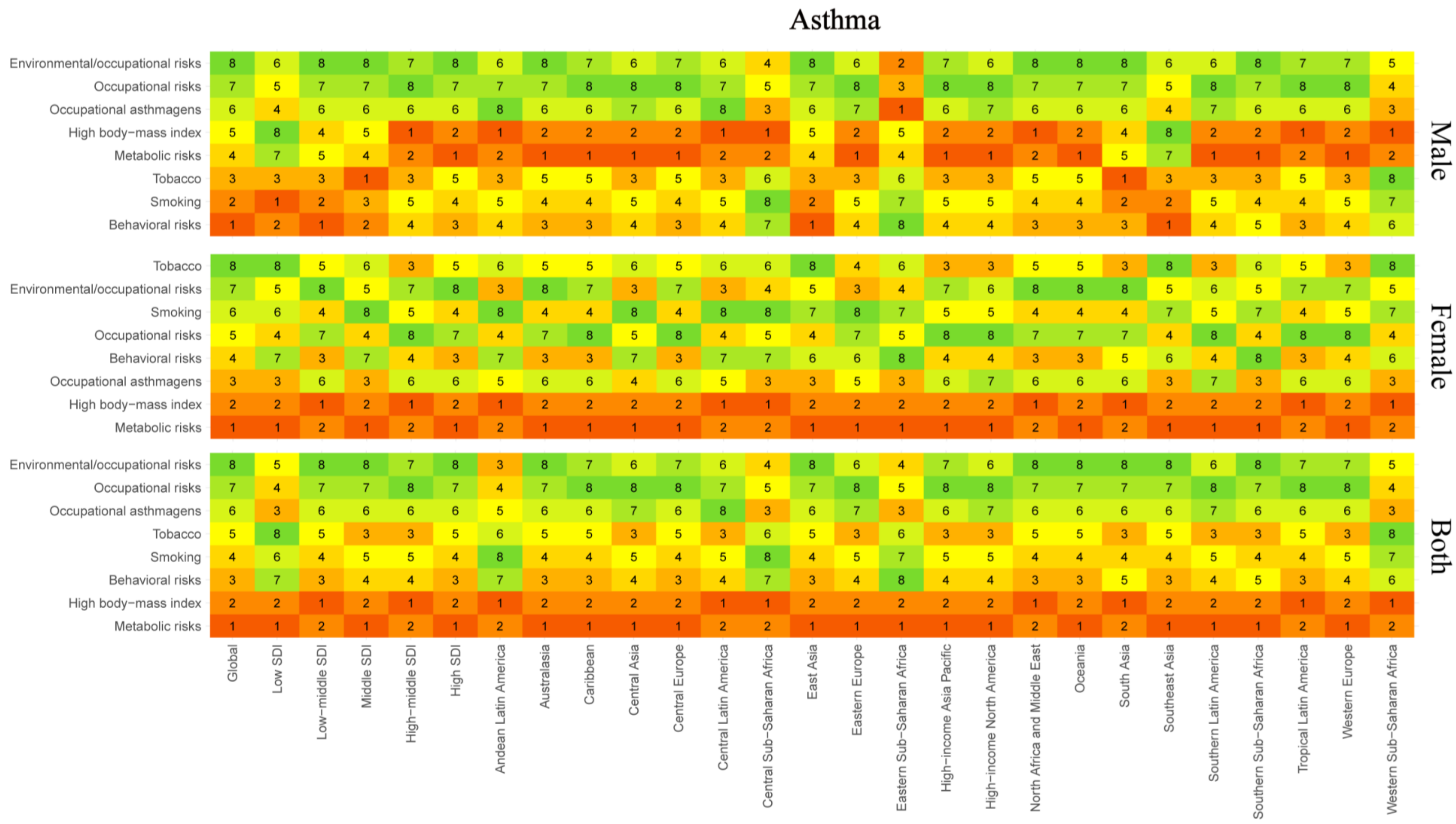

Fig.S10 Risk Factor Figures for the Mortality Rate of Asthma at the Global Level, 5 SDI Regions, and 21 GBD Regions

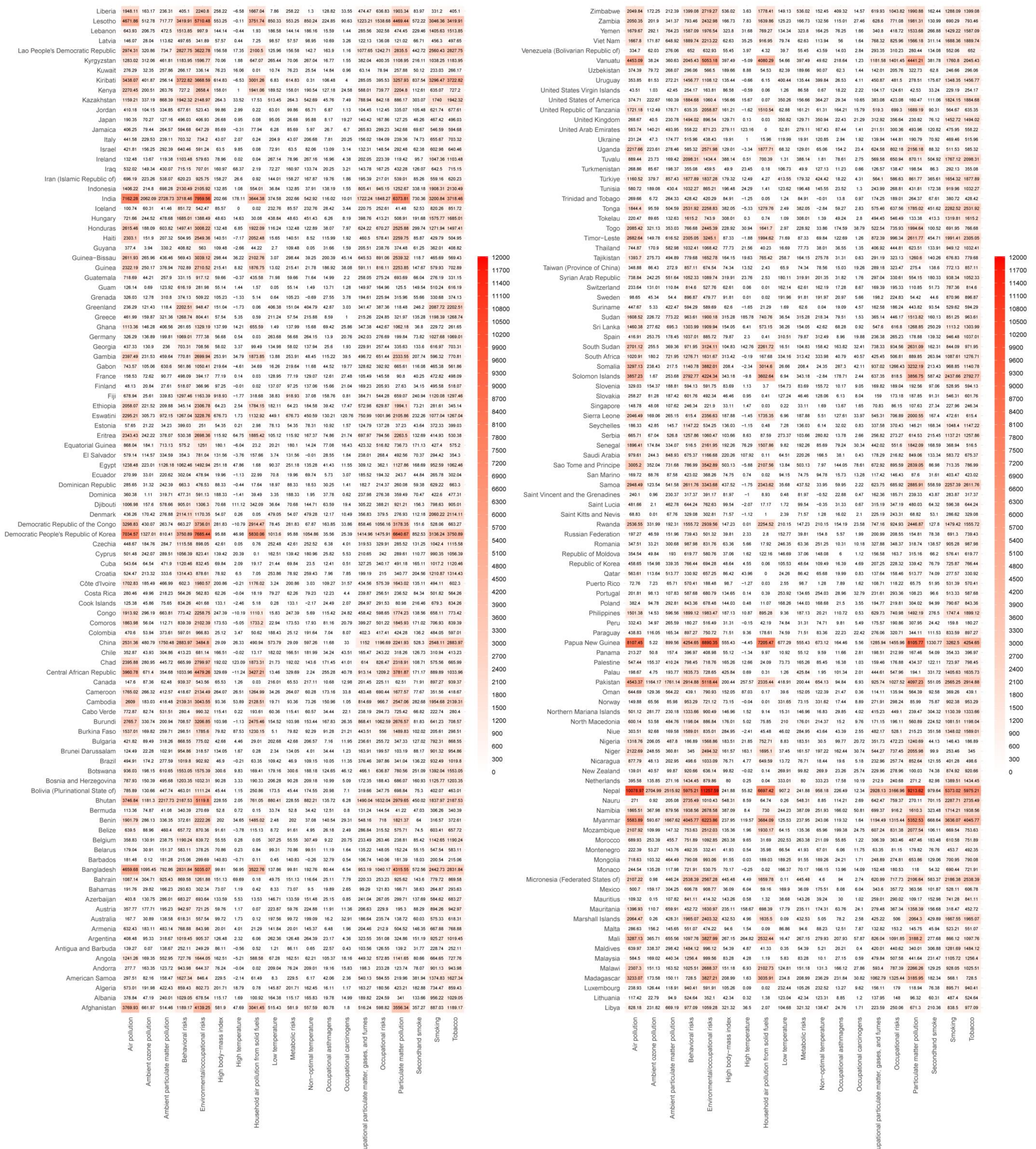

Fig.S11 Risk Factor Figures for the DALYs Rate of CRDs in 204 Countries Globally

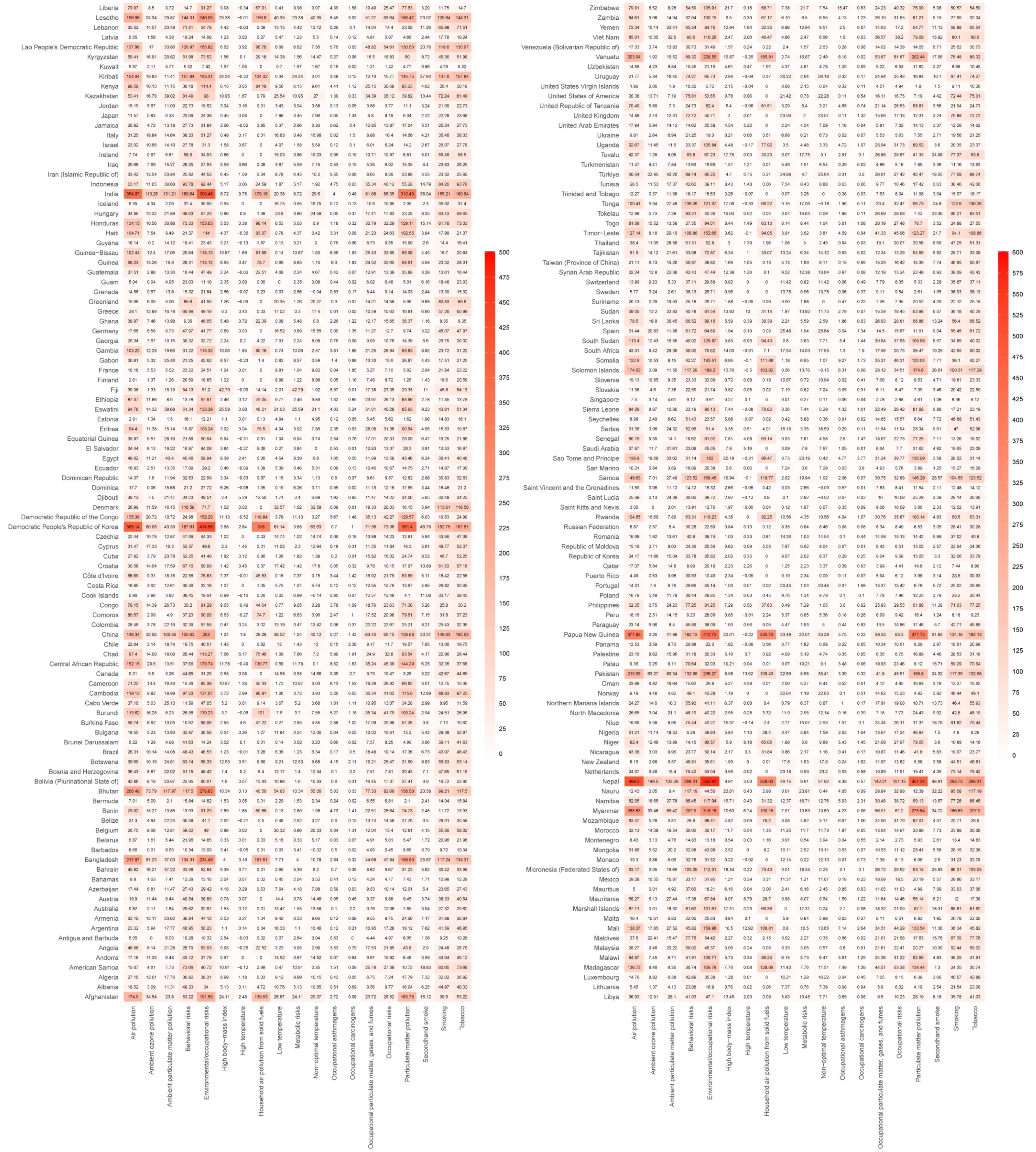

Fig.S12 Risk Factor Figures for the Mortality Rate of CRDs in 204 Countries Globally

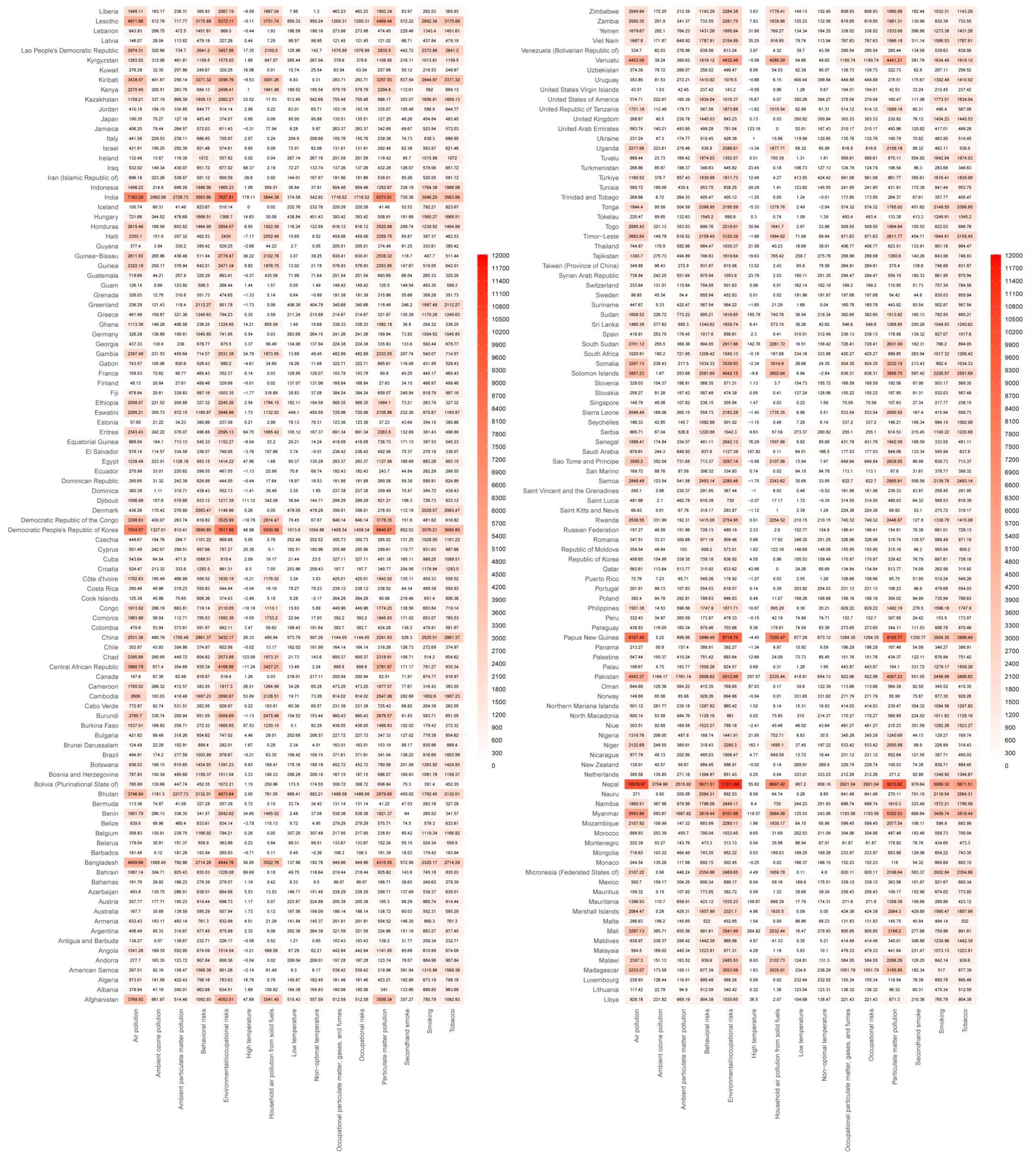

Fig.S13 Risk Factor Figures for the DALYs Rate of COPD in 204 Countries Globally

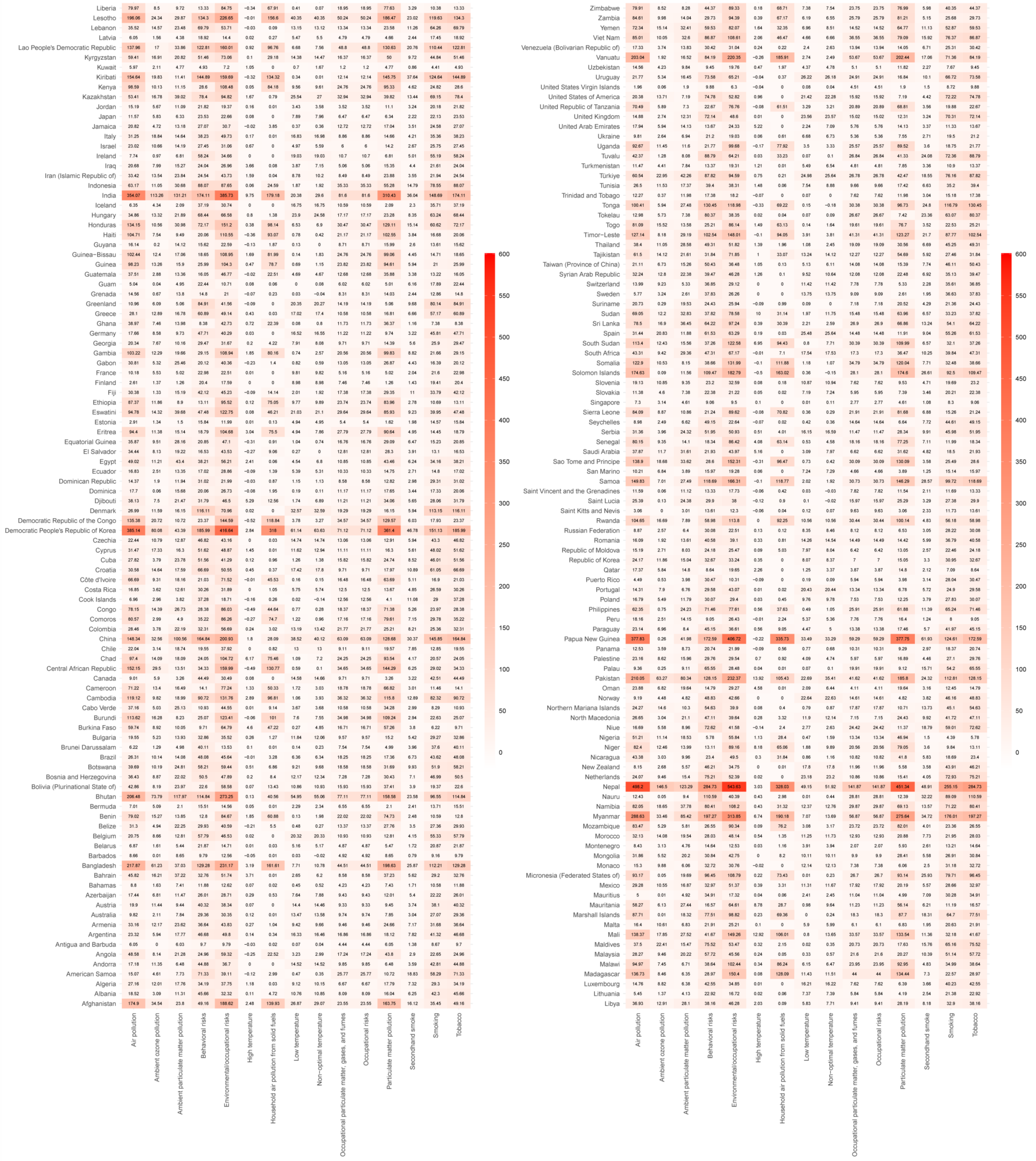

Fig.S14 Risk Factor Figures for the Mortality Rate of COPD in 204 Countries Globally

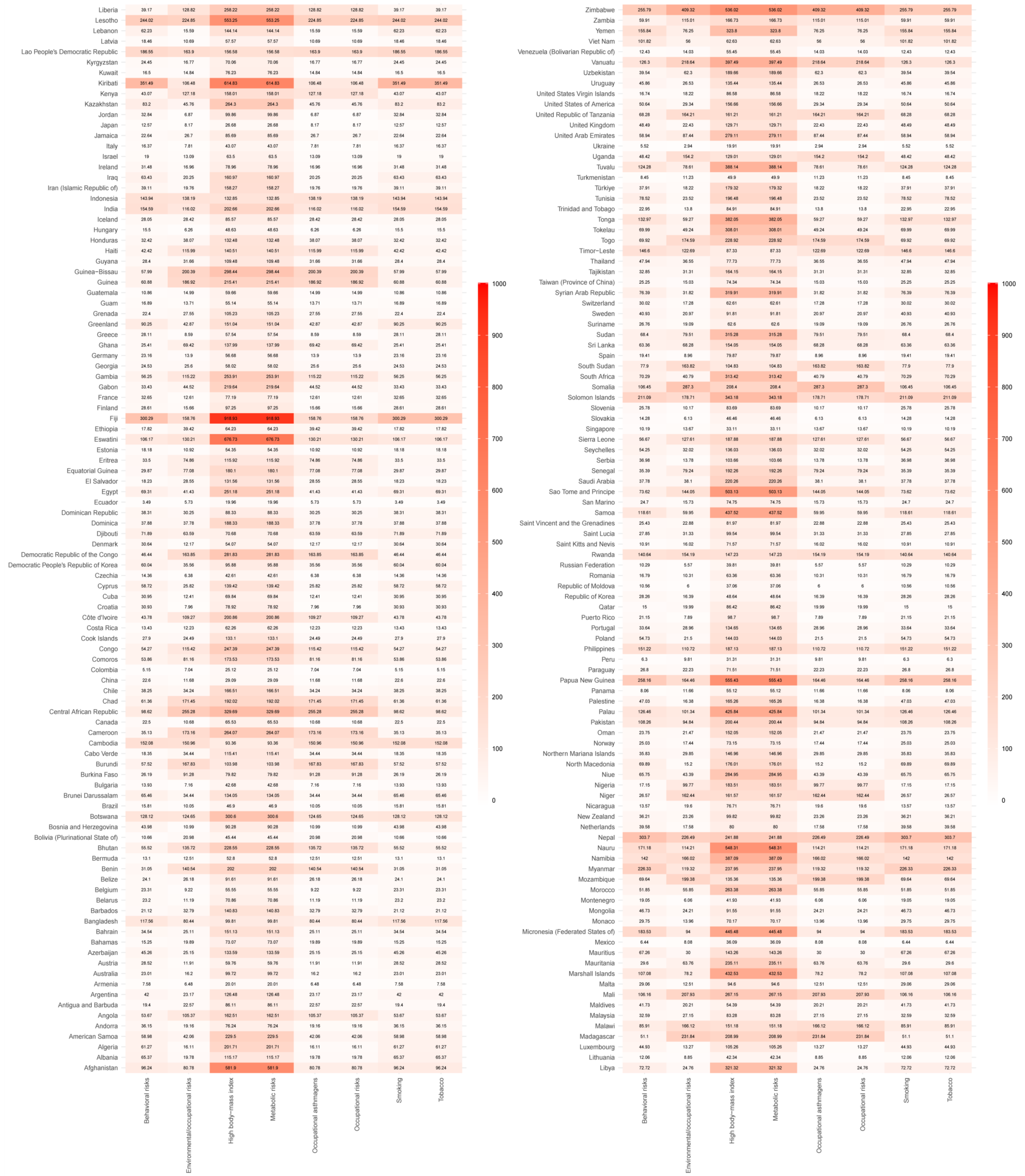

Fig.S15 Risk Factor Figures for the DALYs Rate of Asthma in 204 Countries Globally

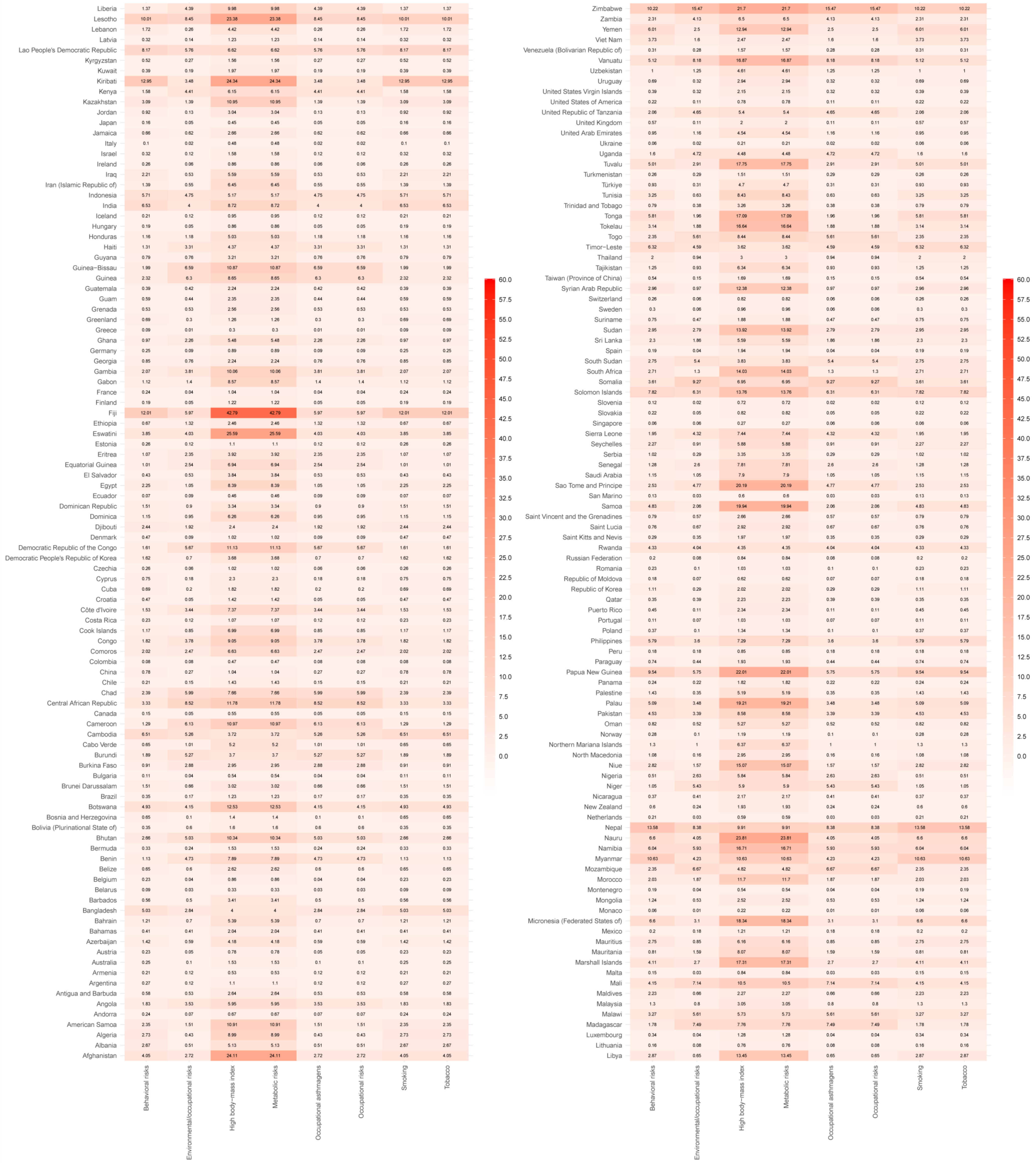

Fig.S16 Risk Factor Figures for the Mortality Rate of Asthma in 204 Countries Globally

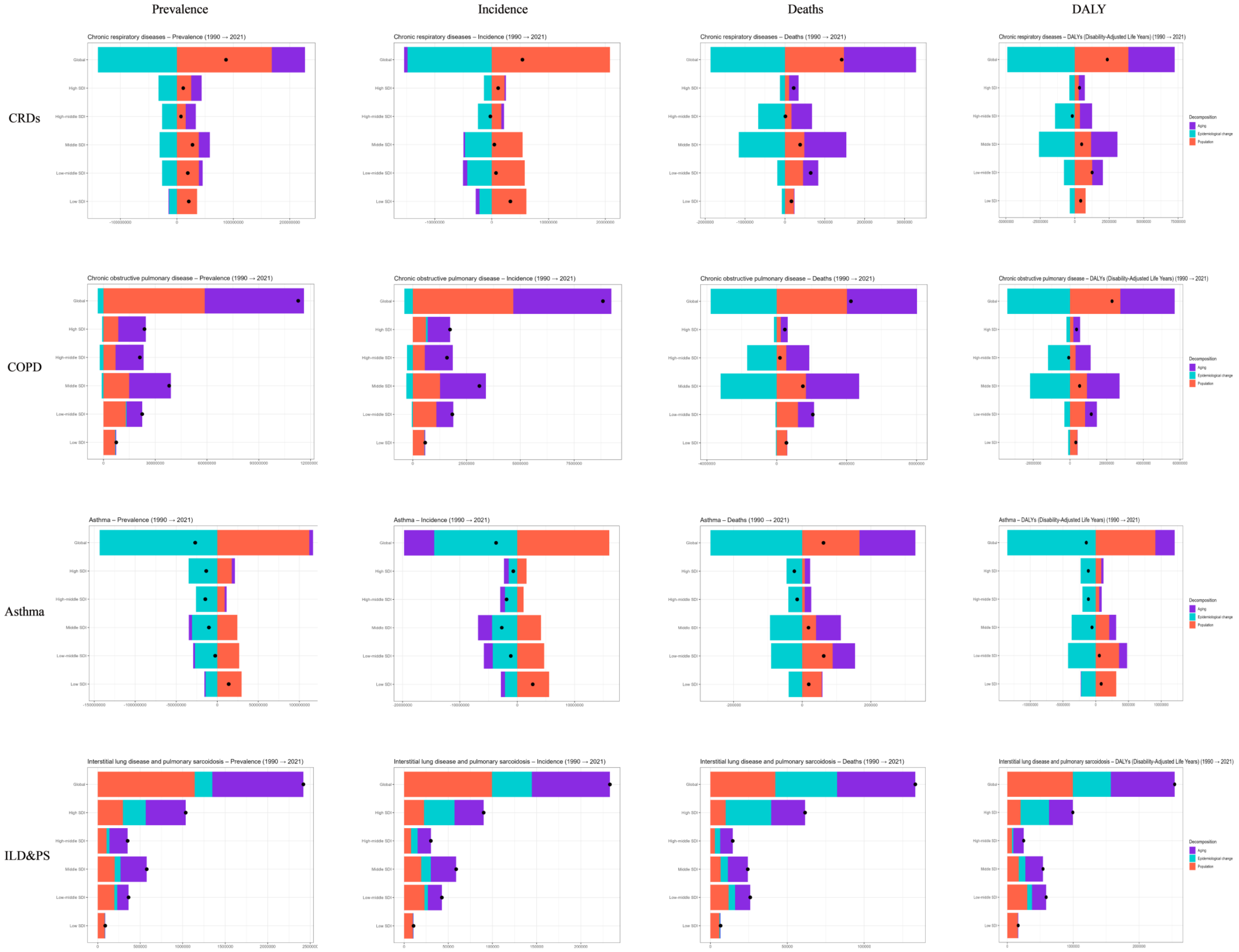

Fig.S17 Decomposition Analysis of CRD-Related Burden Globally and by SDI Region, 1990–2021
